# Supplementary figures and images for: Phylogenetic analysis of the MCL1 BH3 binding groove and rBH3 sequence motifs in the p53 and INK4 protein families
Source: PLoS One. 2023 Jan 25;18(1):e0277726. doi: 10.1371/journal.pone.0277726 (PMC9876281; doi:10.1371/journal.pone.0277726)

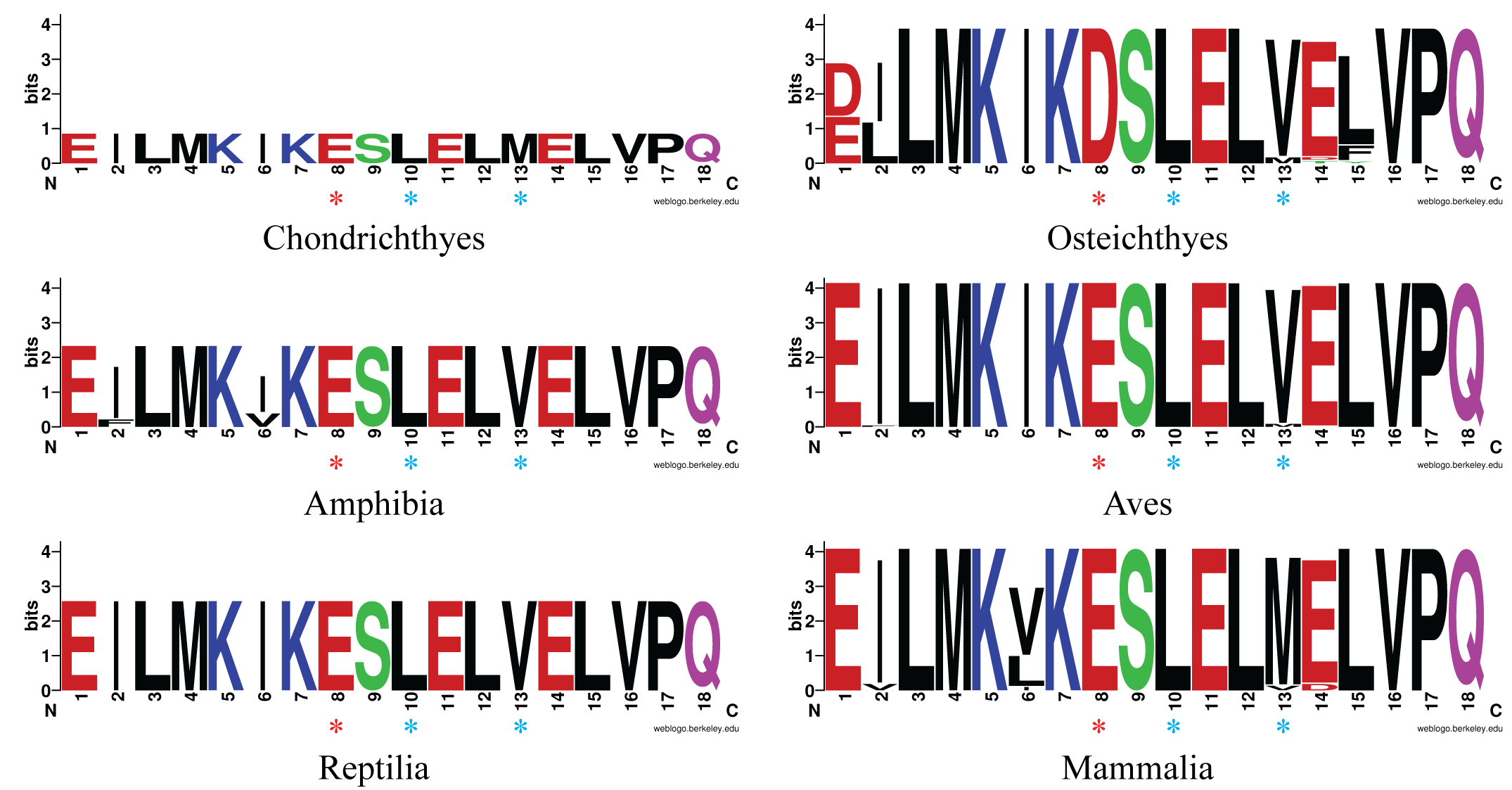

Supplement: S1 Fig — The p73 sequences used to construct the phylogenetic tree were aligned using Clustal Omega and the alignment was analyzed for conservation using sequence logos. The residues are colored based on their chemical properties, with polar residues (G, S, T, Y, C, Q, N) colored in green, basic residues (K, R, H) colored in blue, acidic (D, E) colored in red, and hydrophobic residues (A, V, L, I, P, W, F, M) in black. The three residues known to be important for binding (two hydrophobic residues and one acidic residue) are indicated by blue and red asterisks respectively. The rBH3 and surrounding sequence of the tetramerization domain are strongly conserved in the analyzed sequences (217 total sequences, 4 chondrichthyan, 34 osteichthyan, 7 amphibian, 97 avian, 8 reptilian, and 67 mammalian). (TIF) [file pone.0277726.s001.tif]

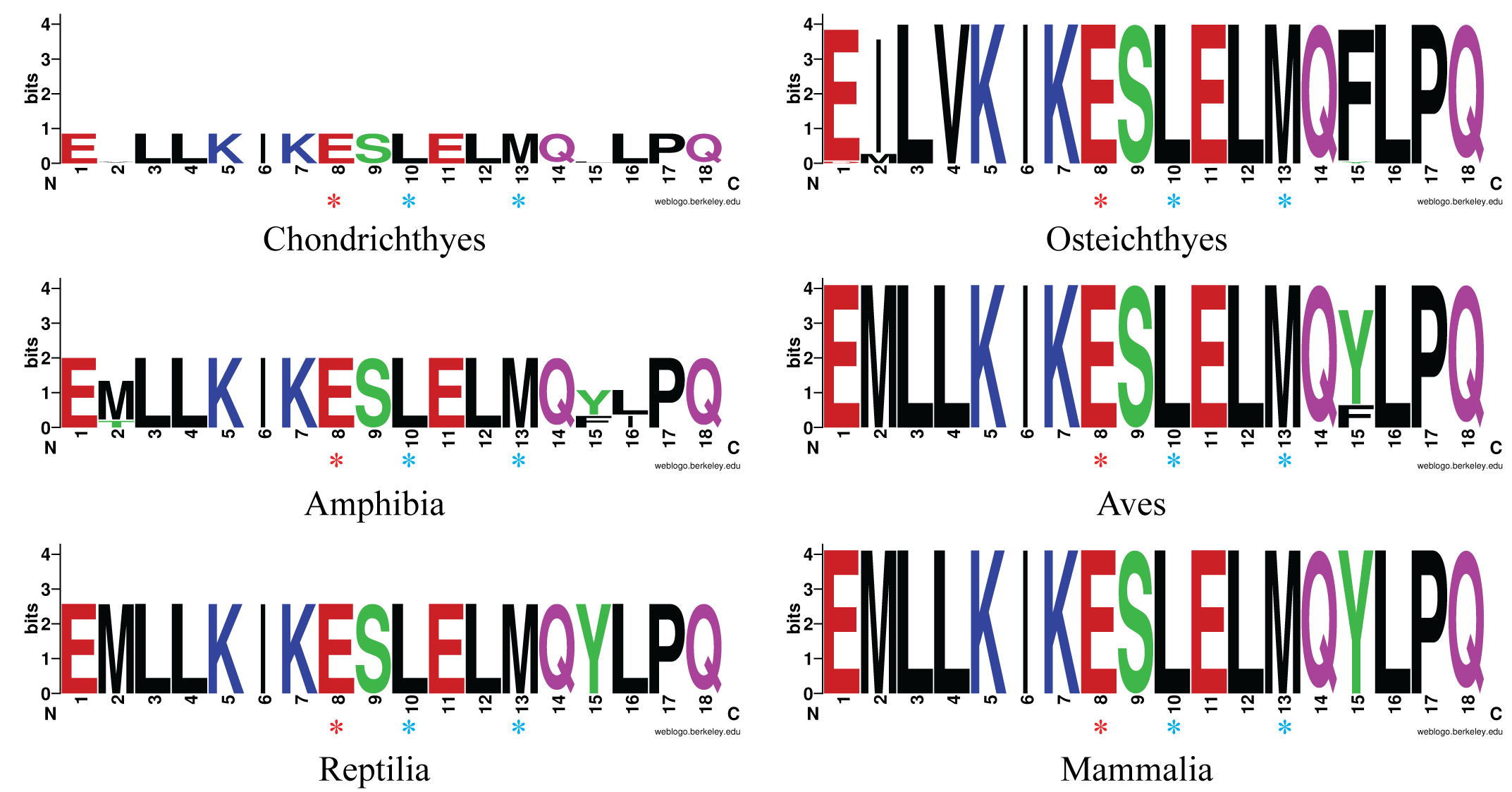

Supplement: S2 Fig — The p63 sequences used in the phylogenetic analysis were aligned using Clustal Omega and the sequence surrounding the putative rBH3 was analyzed using sequence logos. The residues are colored based on their chemical properties, with polar residues (G, S, T, Y, C, Q, N) colored in green, basic residues (K, R, H) colored in blue, acidic (D, E) colored in red, and hydrophobic residues (A, V, L, I, P, W, F, M) in black. Red and blue asterisks represent the conserved acidic and hydrophobic residues known to be important for binding, respectively. The rBH3 motif is conserved throughout all jawed vertebrate classes analyzed (4 chondrichthyan, 46 osteichthyan, 6 amphibian, 70 avian, 8 reptilian, and 79 mammalian, 213 total). The area surrounding the rBH3 motif, which is a part of the p63 tetramerization domain, is also well conserved. (TIF) [file pone.0277726.s002.tif]

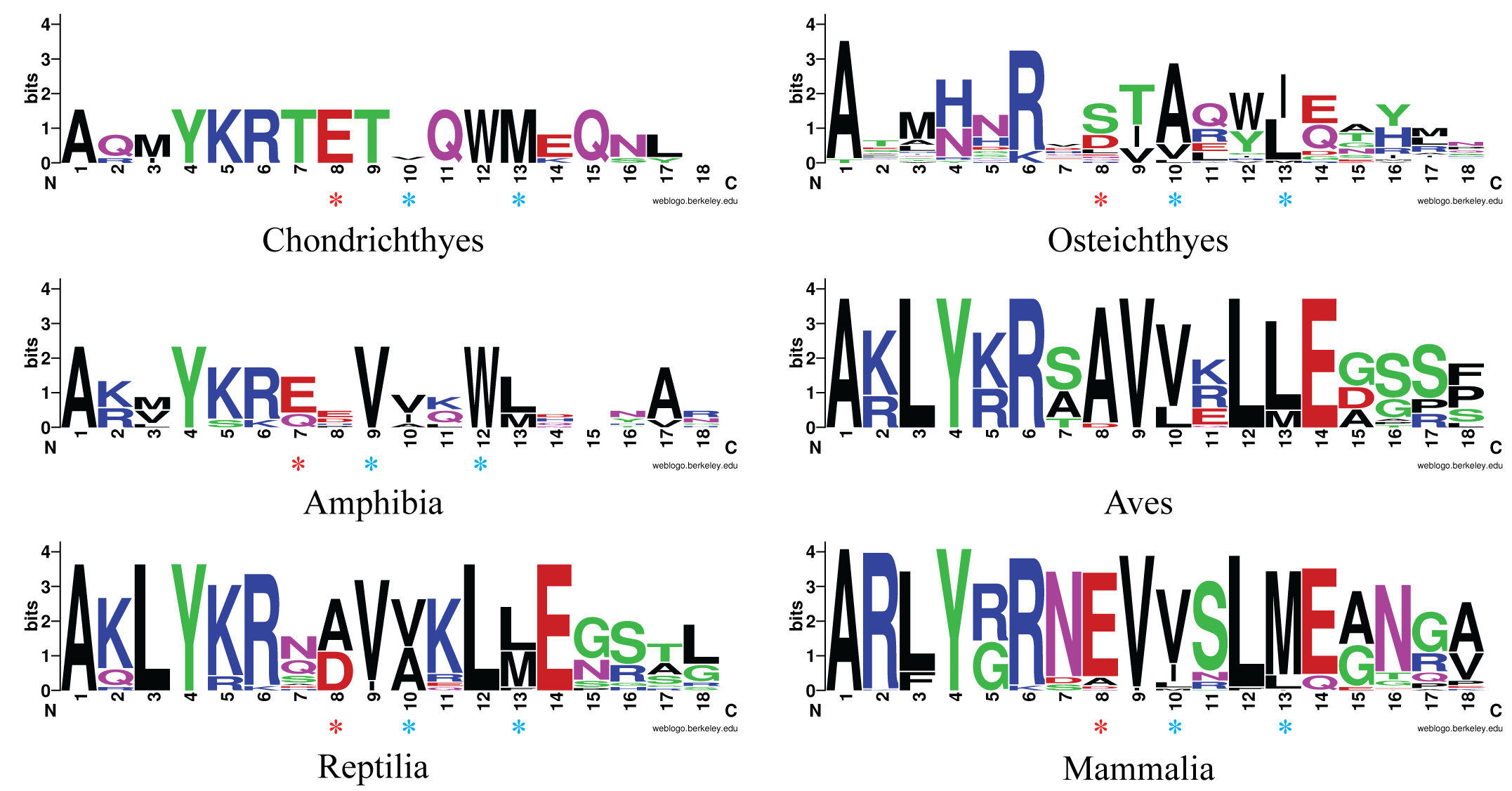

Supplement: S3 Fig — The p18 sequences (150 total sequences, 5 chondrichthyan, 26 osteichthyan, 7 amphibian, 24 avian, 21 reptilian, and 67 mammalian). used to construct the INK4 family phylogenetic tree were aligned using Clustal Omega and the conservation of the rBH3 and surrounding sequence was visualized using sequence logos. The residues are colored based on their chemical properties, with polar residues (G, S, T, Y, C, Q, N) colored in green, basic residues (K, R, H) colored in blue, acidic (D, E) colored in red, and hydrophobic residues (A, V, L, I, P, W, F, M) in black. The three residues known to be important for binding (two hydrophobic residues and one acidic residue) are indicated by blue and red asterisks respectively. The rBH3 motif is conserved in the chondrichthyan, amphibian and mammalian sequences examined, but only partially conserved in the osteichthyan and reptilian sequences. It is lost in avians. Both reptiles and avian sequences have a BH3-like motif at this locus. (TIF) [file pone.0277726.s003.tif]

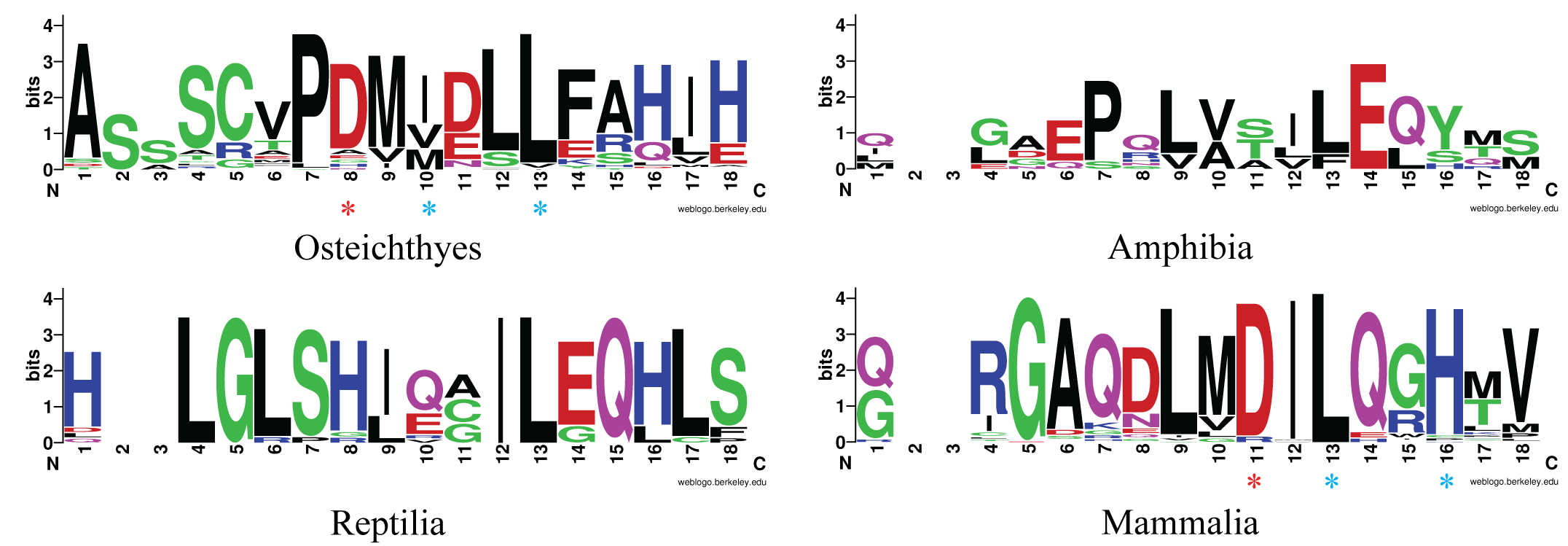

Supplement: S4 Fig — The p19 sequences (188 total sequences, 0 chondrichthyan, 77 osteichthyan, 10 amphibian, 0 avian, 17 reptilian, and 84 mammalian) used in the phylogenetic analysis of the INK4 family were aligned and the sequence area aligning with the rBH3 in human p18 was visualized using sequence logos. The residues are colored based on their chemical properties, with polar residues (G, S, T, Y, C, Q, N) colored in green, basic residues (K, R, H) colored in blue, acidic (D, E) colored in red, and hydrophobic residues (A, V, L, I, P, W, F, M) in black. The three residues known to be important for binding (two hydrophobic residues and one acidic residue) are indicated by blue and red asterisks respectively. There were no chondrichthyan or avian p19 sequences identified. The rBH3 motif was observed and conserved in both osteichthyan and mammalian sequences, but not in the amphibian or reptilian sequences. However, amphibians and reptilians both contain a BH3-like motif in this sequence location. (TIF) [file pone.0277726.s004.tif]
