# Supplementary material for: Phylogenetic analysis of the MCL1 BH3 binding groove and rBH3 sequence motifs in the p53 and INK4 protein families
Source: PLoS One. 2023 Jan 25;18(1):e0277726. doi: 10.1371/journal.pone.0277726 (PMC9876281; doi:10.1371/journal.pone.0277726)
Supplement: S1 File — A total of 151 p53 sequences were used to generate the p53 family phylogenetic tree. (DOCX) [file pone.0277726.s005.docx]

**>NP_000537.3 cellular tumor antigen p53 isoform a [Homo sapiens]**

MEEPQSDPSVEPPLSQETFSDLWKLLPENNVLSPLPSQAMDDLMLSPDDIEQWFTEDPGPDEAPRMPEAAPPVAPAPAAPTPAAPAPAPSWPLSSSVPSQKTYQGSYGFRLGFLHSGTAKSVTCTYSPALNKMFCQLAKTCPVQLWVDSTPPPGTRVRAMAIYKQSQHMTEVVRRCPHHERCSDSDGLAPPQHLIRVEGNLRVEYLDDRNTFRHSVVVPYEPPEVGSDCTTIHYNYMCNSSCMGGMNRRPILTIITLEDSSGNLLGRNSFEVRVCACPGRDRRTEEENLRKKGEPHHELPPGSTKRALPNNTSSSPQPKKKPLDGEYFTLQIRGRERFEMFRELNEALELKDAQAGKEPGGSRAHSSHLKSKKGQSTSRHKKLMFKTEGPDSD

>XP_018868681.2 cellular tumor antigen p53 isoform X1 [Gorilla gorilla gorilla]

MEPCISSQTAFRVTAMEEPQSDPSVEPPLSQETFSDLWKLLPENNVLSPLPSQAMDDLMLSPDDIEQWFTEDPGPDEAPRMPEAAPPVAPAPAAPTPAAPAPAPSWPLSSSVPSQKTYQGSYGFRLGFLHSGTAKSVTCTYSPALNKMFCQLAKTCPVQLWVDSTPPPGTRVRAMAIYKQSQHMTEVVRRCPHHERCSDSDGLAPPQHLIRVEGNLRVEYLDDRNTFRHSVVVPYEPPEVGSDCTTIHYNYMCNSSCMGGMNRRPILTIITLEDSSGNLLGRNSFEVRVCACPGRDRRTEEENLRKKGEPHHELPPGSTKRALPNNTSSSPQPKKKPLDGEYFTLQIRGRERFEMFRELNEALELKDAQAGKEPGGSRAHSSHLKSKKGQSTSRHKKLMFKTEGPDSD

>XP_003810114.2 cellular tumor antigen p53 [Pan paniscus]

MEEPQSDPSVEPPLSQETFSDLWKLLPENNVLSPLPSQAMDDLMLSPDDIEQWFTEDPGPDEAPRMPEAAPRVAPAPAAPTPAAPAPAPSWPLSSSVPSQKTYQGSYGFRLGFLHSGTAKSVTCTYSPALNKMFCQLAKTCPVQLWVDSTPPPGTRVRAMAIYKQSQHMTEVVRRCPHHERCSDSDGLAPPQHLIRVEGNLRVEYLDDRNTFRHSVVVPYEPPEVGSDCTTIHYNYMCNSSCMGGMNRRPILTIITLEDSSGNLLGRNSFEVRVCACPGRDRRTEEENLRKKGEPHHELPPGSTKRALPNNTSSSPQPKKKPLDGEYFTLQIRGRERFEMFRELNEALELKDAQAGKEPGGSRAHSSHLKSKKGQSTSRHKKLMFKTEGPDSD

>XP_030656345.1 cellular tumor antigen p53 [Nomascus leucogenys]

MEEPQSDPSVEPPLSQETFSDLWKLLPENNVLSPLPSQAMDDLMLSPEDIAQWFTEDPGPHEAPRMSEAAPPMAPAPAAPTLAAPAPAPSWPLSSSVPSQKTYQGSYGFRLGFLHSGTAKSVTCTYSPALNKMFCQLAKTCPVQLWVDSTPPPGTRVRAMAIYKQSQHMTEVVRRCPHHERCSDSDGLAPPQHLIRVEGNLRVEYLDDRNTFRHSVVVPYEPPEVGSDCTTIHYNYMCNSSCMGGMNRRPILTIITLEDSSGNLLGRNSFEVRVCACPGRDRRTEEENFHKKGEPHHELPPGSTKRALPNNTSSSPQPKKKPLDGEYFTLQIRGRERFEMFRELNEALELKDAQAGKEPGGSRAHSSHLKSKKGQSTSRHKKLMFKTEGPDSD

>XP_002827020.2 cellular tumor antigen p53 [Pongo abelii]

MEEPQSDPSVEPPLSQETFSDLWKLLPENNVLSPLPSQAVDDLLLSPDDIAQWFIEDPGPDEAPRMSEAASPVDPAPAAPIPAAPAPAPSWPLSSSVPSQKTYQGSYGFRLGFLHSGTAKSVTCTYSPALNKMFCQLAKTCPVQLWVDSTPPPGTRVRAMAIYKQSQHMTEVVRRCPHHERCSDSDGLAPPQHLIRVEGNLRVEYLDDRNTFRHSVVVPYEPPEVGSDCTTIHYNYMCNSSCMGGMNRRPILTIITLEDSSGNLLGRNSFEVRVCACPGRDRRTEEENFRKKGEPHHELPPGSTKRALPNNTSSSPQPKKKPLDGEYFTLQIRGRERFEMFRELNEALELKDAQAGKEPGGSRAHSSHLKSKKGQSTSRHKKLMFKTEGPDSD

>XP_031995228.1 cellular tumor antigen p53 [Hylobates moloch]

MEEPQSDPSVEPPLSQETFSDLWKLLPENNVLSPLPSQAMDDLMLSPEDIAQWFTEDPGPHEAPRMSEAAPPMAPAPGAPTLAAPAPAPSWPLSSSVPSQKTYQGSYGFRLGFLHSGTAKSVTCTYSPALNKMFCQLAKTCPVQLWVDSTPPPGTRVRAMAIYKQSQHMTEVVRRCPHHERCSDSDGLAPPQHLIRVEGNLRVEYLDDRNTFRHSVVVPYEPPEVGSDCTTIHYNYMCNSSCMGGMNRRPILTIITLEDSSGNLLGRNSFEVRVCACPGRDRRTEEENFHKKGEPHHELPPGSTKRALPNNTSSSPQPKKKPLDGEYFTLQIRGRERFEMFRELNEALELKDAQAGKEPGGSRAHSSHLKSKKGQSTSRHKKLMFKTEGPDSD

>XP_010360689.1 cellular tumor antigen p53 [Rhinopithecus roxellana]

MEEPQSDPSIEPPLSQETFSDLWKLLPENNVLSPLPSQAVDDLMLSPDDLAQWLTEDPGPDEAPRMSEAAPPMAPTPAAPTPAAPAPAPSWPLSSSVPSQKTYHGSYGFRLGFLHSGTAKSVTCTYSPDLNKMFCQLAKTCPVQLWVDSTPPPGSRVRAMAIYKQSQHMTEVVRRCPHHERCSDSDGLAPPQHLIRVEGNLRVEYSDDRNTFRHSVVVPYEPPEVGSDCTTIHYNYMCNSSCMGGMNRRPILTIITLEDSSGNLLGRNSFEVRVCACPGRDRRTEEENYRKKGEPCHELPPGSTKRALPNNTSSSPQPKKKPLDGEYFTLQIRGRERFEMFRELNEALELKDAQAGKEPAGSRAHSSHLKSKKGQSTSRHKKLMFKTEGPDSD

>XP_016786959.2 cellular tumor antigen p53 isoform X1 [Pan troglodytes]

METVSGSIGKAGPPPPHPNPSPLVETCGKRKFHGTDFLLLSFRLPENNVLSPLPSQAMDDLMLSPDDIEQWFTEDPGPDEAPRMPEAAPPVAPAPAAPTPAAPAPAPSWPLSSSVPSQKTYQGSYGFRLGFLHSGTAKSVTCTYSPALNKMFCQLAKTCPVQLWVDSTPPPGTRVRAMAIYKQSQHMTEVVRRCPHHERCSDSDGLAPPQHLIRVEGNLRVEYLDDRNTFRHSVVVPYEPPEVGSDCTTIHYNYMCNSSCMGGMNRRPILTIITLEDSSGNLLGRNSFEVRVCACPGRDRRTEEENLRKKGEPHHELPPGSTKRALPNNTSSSPQPKKKPLDGEYFTLQIRGRERFEMFRELNEALELKDAQAGKEPGGSRAHSSHLKSKKGQSTSRHKKLMFKTEGPDSD

>XP_033040608.1 cellular tumor antigen p53 isoform X1 [Trachypithecus francoisi]

MEEPQSDPSIEPPLSQETFSDLWKLLPENNVLSPLPSQAVDDLMLSPDDLAQWLTEDPGPDEAPRMSEAAPPMAPTPAAPTPAAPAPAPSWPLSSSVPSQKTYHGSYGFRLGFLHSGTAKSVTCTYSPDLNKMFCQLAKTCPVQLWVDSTPPPGSRVRAMAIYKQSQHMTEVVRRCPHHERCSDSDGLAPPQHLIRVEGNLRVEYSDDRNTFRHSVVVPYEPPEVGSDCTTIHYNYMCNSSCMGGMNRRPILTIITLEDSSGNLLGRNSFEVRVCACPGRDRRTEEENFRKKGEPCHELPPGSTKRALPNNTSSSPQPKKKPMDGEYFTLQIRGRERFEMFRELNEALELKDAQAGKEPAGSRAHSSHLKSKKGQSTSRHKKLMFKTEGPDSD

>XP_023049082.1 cellular tumor antigen p53 isoform X1 [Piliocolobus tephrosceles]

MEEPQSDPSIEPPLSQETFSDLWKLLPENNVLSPLPSQAVDDLMLSPDDLAQWLTEDPSPDEAPRMSEAAPPMAPTPAAPTPAAPAPAPSWPLSSSVPSQKTYHGSYGFRLGFLHSGTAKSVTCTYSPDLNKMFCQLAKTCPVQLWVDSAPPPGSRVRAMAIYKQSQHMTEVVRRCPHHERCSDSDGLAPPQHLIRVEGNLRVEYLDDRNTFRHSVVVPYEPPEVGSDCTTIHYNYMCNSSCMGGMNRRPILTIITLEDSSGNLLGRNSFEVRVCACPGRDRRTEEENFRKKGEPCHELPPGSTKRALPNNTSSSPQPKKKPLDGEYFTLQIRGRERFEMFRELNEALELKDAQAGKEPAGSRAHSSHLKSKKGQSTSRHKKLMFKTEGPDSD

>XP_003912321.1 cellular tumor antigen p53 [Papio anubis]

MEEPQSDPSIEPPLSQETFSDLWKLLPENNVLSPLPSQAVDDLMLSPDDLAQWLTEDPGPDEAPRMSEATPPMAPTPAAPTPAAPAPAPSWPLSSSVPSQKTYHGSYGFRLGFLHSGTAKSVTCTYSPDLNKMFCQLAKTCPVQLWVDSTPPPGSRVRAMAIYKQSQHMTEVVRRCPHHERCSDSDGLAPPQHLIRVEGNLRVEYSDDRNTFRHSVVVPYEPPEVGSDCTTIHYNYMCNSSCMGGMNRRPILTIITLEDSSGNLLGRNSFEVRVCACPGRDRRTEEENFRKKGEPCHELPPGSTKRALPNNTSSSPQPKKKPLDGEYFTLQIRGRERFEMFRELNEALELKDAQAGKEPAGSRAHSSHLKSKKGQSTSRHKKFMFKTEGPDSD

>NP_001040616.1 cellular tumor antigen p53 [Macaca mulatta]

MEEPQSDPSIEPPLSQETFSDLWKLLPENNVLSPLPSQAVDDLMLSPDDLAQWLTEDPGPDEAPRMSEAAPPMAPTPAAPTPAAPAPAPSWPLSSSVPSQKTYHGSYGFRLGFLHSGTAKSVTCTYSPDLNKMFCQLAKTCPVQLWVDSTPPPGSRVRAMAIYKQSQHMTEVVRRCPHHERCSDSDGLAPPQHLIRVEGNLRVEYSDDRNTFRHSVVVPYEPPEVGSDCTTIHYNYMCNSSCMGGMNRRPILTIITLEDSSGNLLGRNSFEVRVCACPGRDRRTEEENFRKKGEPCHQLPPGSTKRALPNNTSSSPQPKKKPLDGEYFTLQIRGRERFEMFRELNEALELKDAQAGKEPAGSRAHSSHLKSKKGQSTSRHKKFMFKTEGPDSD

>AAB91535.1 P53 [Macaca fascicularis]

MEEPQSDPSIEPPLSQETFSDLWKLLPENHVLSPLPSQAVDDLMLSPDDLAQWLTEDPGPDEAPRMSEAAPPMAPTPAAPTPAAPAPAPSWPLSSSVPSQKTYHGSYGFRLGFLHSGTAKSVTCTYSPDLNKMFCQLAKTCPVQLWVDSTPPPGSRVRAMAIYKQSQHMTEVVRRCPHHERCSDSDGLAPPQHLIRVEGNLRVEYSDDRNTFRHSVVVPYEPPEVGSDCTTIHYNYMCNSSCMGGMNRRPILTIITLEDSSGNLLGRNSFEVRVCACPGRDRRTEEENFRKKGEPCHQLPPGSTKRALPNNTSSSPQPKKKPLDGEYFTLQIRGRERFEMFRELNEALELKDAQAGKEPAGSRAHSSHLKSKKGQSTSRHKKFMFKTEGPDSD

>XP_025219300.1 cellular tumor antigen p53 isoform X1 [Theropithecus gelada]

MEEPQSDPSIEPPLSQETFSDLWKLLPENNVLSPLPSQAVDDLMLSPDDLAQWLTEDPGPDEAPRMSEATPPMAPTPAAPTPAAPAPAPSWPLSSSVPSQKTYHGSYGFRLGFLHSGTAKSVTCTYSPDLNKMFCQLAKTCPVQLWVDSTPPPGSRVRAMAIYKQSQHMTEVVRRCPHHERCSDSDGLAPPQHLIRVEGNLRVEYSDDRNTFRHSVVVPYEPPEVGSDCTTIHYNYMCNSSCMGGMNRRPILTIITLEDSSSGNLLGRNSFEVRVCACPGRDRRTEEENFRKKGEPCHELPPGSTKRALPNNTSSSPQPKKKPLDGEYFTLQIRGRERFEMFRELNEALELKDAQAGKEPAGSRAHSSHLKSKKGQSTSRHKKFMFKTEGPDSD

>XP_002747994.1 cellular tumor antigen p53 isoform X1 [Callithrix jacchus]

MEEPQSDLSIEPPLSQETFSDLWKLLPENNILSSSLSQPVDDLMLSPDDIDIAQWLSQDPVPDEAPTVSEAPPAMAQAPAAPTLVAPTPAPSWPLSSSVPSQKTYHGDYGFRLGFLHSGTAKSVTCTYSPALNKMFCQLAKTCPVQLWVDSTPPRGTRVRAMAIYKQSQHMTEVVRRCPHHERCSDSDGLAPPQHLIRVEGNLHVEYLDDKNTFRHSVVVPYEPPEVGSDCTTIHYNYMCNSSCMGGMNRRPILTIITLEDSSGNLLGRNSFEVRVCACPGRDRRTEEENFRKKGEPCLDLPPGSTKRAMPNSTSSSPQPKKKPLDGEYFTLQIHGRERFEMFRELNEALELKDAQAGKEPGGSRAHSNHLKSKKGQCTSRHKKLMVKREGPDSD

>NP_001274298.1 cellular tumor antigen p53 [Tupaia chinensis]

MEEPQSDPSVEPPLSQETFSDLWKLLPENNVLSPLPSQAMDDLMLSPDDIEQWFTEDPGPDEAPRMPEAAPPVAPAPAAPTPAAPAPAPSWPLSSSVPSQKTYQGSYGFRLGFLHSGTAKSVTCTYSPDLNKLFCQLAKTCPVQLWVDSAPPPGTRVRAMAIYKQSQYVTEVVRRCPHHERCSDSDGLAPPQHLIRVEGNLHAEYSDDRNTFRHSVVVPYEPPEVGSDCTTIHYNYMCNSSCMGGMNRRPILTIITLEDSSGKLLGRNSFEVRICACPGRDRRTEEENFRKKGESCPKLPTGSIKRALPTGSSSSPQPKKKPLDEEYFTLQIRGRERFEMLREINEALELKDAMAGKESAGSRAHSSHLKSKKGQSTSRHRKLMFKTEGPDSD

>XP_003929235.1 cellular tumor antigen p53 isoform X2 [Saimiri boliviensis boliviensis]

MEEPQSDLSIEPPLSQETFSDLWKLLPENNILSSSLSQPVDDLMLSPDDIAQWFSQDPVPDESPTVSEAPPAVAQAPAAPTSAAPTPAPSWPLSSSVPSQKTYHGDYGFRLGFLHSGTAKSVTCTYSPALNKMFCQLAKTCPVQLWVDSTPPRGTRVRAMAIYKQSQHMTEVVRRCPHHERCSDSDGLAPPQHLIRVEGNLHVEYLDDKNTFRHSVVVPYEPPEVGSDCTTIHYNYMCNSSCMGGMNRRPILTIITLEDSSGNLLGRNSFEVRVCACPGRDRRTEEENFRKKGEPCLDLPPGSTKRAMPNSTSSSPQPKKKPLDGEYFTLQIRGRERFEMFRELNEALELKDAQAGKEPGGSRAHSSHLKSKKGQCTSRHKKLMVKREGPDSD

>XP_017387083.1 cellular tumor antigen p53 isoform X1 [Cebus imitator]

MEEPHSDLSIEPPLSQETFSDLWKLLPENNILSPSLSQPVDDLMLSPDDIDIAQWFSQDPVPDEDSTVSEAPPTVAQAPAAPTSAAPTPAPSWPLSSSVPSQKTYRGDYGFRLGFLHSGTAKSVTCTYSPDLNKMFCQLAKTCPVQLWVDSTPPRGTRVRAMAIYKQSQHMTEVVRRCPHHERCSDSDGLAPPQHLIRVEGNLHVEYLDDKNTFRHSVVVPYEPPEVGSDCTTIHYNYMCNSSCMGGMNRRPILTIITLEDSSGNLLGRNSFEVRVCACPGRDRRTEEENFRKKGEPCLDLPPGSTKRAMPNSTSSSPQPKKKPLDGEYFTLQIRGRERFEMFRELNEALELKDAQAGKEPGGSRAHSSHLKSKKGQCTSRHKKLMVKREGPDSD

>XP_008008383.2 cellular tumor antigen p53 isoform X1 [Chlorocebus sabaeus]

METVSRPMGGADPLSPPQPQPPSRGLWEAKKIHGTDFLLLSFRLPENNVLSPLPSQAVDDLMLSPDDLAQWLTEDPGPDEAPRMSEAAPPMAPTPAAPTPAAPAPAPSWPLSSSVPSQKTYHGSYGFRLGFLHSGTAKSVTCTYSPDLNKMFCQLAKTCPVQLWVDSTPPPGSRVRAMAIYKQSQHMTEVVRRCPHHERCSDSDGLAPPQHLIRVEGNLRVEYSDDRNTFRHSVVVPYEPPEVGSDCTTIHYNYMCNSSCMGGMNRRPILTIITLEDSSGNLLGRNSFEVRVCACPGRDRRTEEENFRKKGEPCHELPPGSTKRALPNNTSSSPQPKKKPLDGEYFTLQIRGRERFEMFRELNEALELKDAQAGKEPAGSRAHSSHLKSKKGQSTSRHKKFMFKTEGPDSD

>XP_008060532.1 cellular tumor antigen p53 [Carlito syrichta]

MEEPQSDLSIEPLSQETFSDLWKLLPENNVLSPSLSPPVDDLILSTEDIANWFSEGPDEALRTAPAPVAPTPAASTQAAPAPGTPWPLSSSVPSQKTYHGNYGFRLGFLHSGTAKSVTCTYSPALNKMFCQLAKTCPVQLWVDSTPPPGTRVRAMAIYKQSQYMTEVVRRCPHHERCSDSDGLAPPQHLIRVEGNLRVEYLDDKTTFRHSVVVPYEPPEVGSDCTTIHYNYMCNSSCMGGMNRRPILTIITLEDSSGNLLGRNSFEVRVCACPGRDRRTEEENFRKKGEPCSELPPGSTKRALPTSTSSPSQPKKKPLDGEYFTLQIRGRERFEMFRELNEALELKDAQAGKEPGGSRAHTSHLKSKKGQSTSRHKKLMFKREGPDSD

>KAF7473436.1 cellular tumor antigen p53 [Marmota monax]

MPELPLTVHHELGDRAWSVLPRSVTRSPEVGRPDCILNAAMEEPQSDLSIEPPLSQETFSDLWNLLPENNVLSPVLSPPMDDLLLSSEDVENWFDKGPDEALQMSAAPAPKAPTPAASTLAAPSPATSWPLSSSVPSQNTYPGVYGFRLGFLHSGTAKSVTCTYSPSLNKLFCQLAKTCPVQLWVDSTPPPGTRVRAMAIYKKSQHMTEVVRRCPHHERCSDSDGLAPPQHLIRVEGNLRAEYLDDRNTFRHSVVVPYEPPEVGSECTTIHYNYMCNSSCMGGMNRRPILTIITLEDSSGNLLGRNSFEVRVCACPGRDRRTEEENFRKRGEPCPEPPPRSTKRALPNGTSSSPQPKKKPLDGEYFTLKIRGRARFEMFQELNEALELKDAQAEKEPGESRPHPSYLKSKKGQSTSRHKKIIFKREGPDSD

>XP_005332876.1 cellular tumor antigen p53 [Ictidomys tridecemlineatus]

MEEPQSDLSIEPPLSQETFSDLWNLLPENNVLSPVLSPPMDDLLLSSEDVENWFDKGPDEALQMSAAPAPKAPTPAASTLAAPAPATSWPLSSSVPSQNTYPGVYGFRLGFIHSGTAKSVTCTYSPSLNKLFCQLAKTCPVQLWVDSTPPPGTRVRAMAIYKKSQHMTEVVRRCPHHERCSDSDGLAPPQHLIRVEGNLRAEYLDDRNTFRHSVVVPYEPPEVGSECTTIHYNYMCNSSCMGGMNRRPILTIITLEDSSGNLLGRNSFEVRVCACPGRDRRTEEENFRKRGEPCPEPPPGSTKRALPTGTSSSPQPKKKPLDGEYFTLKIRGRARFEMFQELNEALELKDAQAEKEPGESRPHPSYLKSKKGQSTSRHKKIIFKREGPDSD

>XP_012631512.1 cellular tumor antigen p53 [Microcebus murinus]

MEEPQSDLSIEAPLSQETFSDLWKLLPENNVLSSSLSPPVDDLMLSAEDFAGWFTEGPDEAARMSENPEPAAPVPATPTPVASTPTWPLSSSVPSQKTYPGNYGFRLGFLHSGTAKSVTCTYSPALNKMFCQLAKTCPVQLWVDSTPPPGSRIRAMAIYKQSQHMTEVVRRCPHHERCSDSDGLAPSQHLIRVERNLRVEYLDDRNTFRHSVVVPYEPPEVGSDCTTIHYNYMCNSSCMGGMNRRPIVTIITLEDSNGIVLGRNSFEVRVCACPGRDRRTEEENFRKKGEPCSGSAKRALPTNTSSSPPPKKKPLDGEYFTLQIRGRERFEMFRMLNEALEFKDAQTGKEPGESRAHSSHLKSKKGQSTSRHKKLMFKREGPDSD

>XP_026246322.1 cellular tumor antigen p53 [Urocitellus parryii]

MEEPQSDLSIEPPLSQETFSDLWNLLPENNVLSPVLSPPMDDLLLSSEDVENWFDKGPDEALQMSAAPAPKAPTPAASTLAAPAPATSWPLSSSVPSQNTYPGVYGFRLGFIHSGTAKSVTCTYSPSLNKLFCQLAKTCPVQLWVDSTPPPGTRVRAMAIYKKSQHMTEVVRRCPHHERCSDSDGLAPPQHLIRVEGNLRAEYLDDRNTFRHSVVVPYEPPEVGSECTTIHYNYMCNSSCMGGMNRRPILTIITLEDSSGNLLGRNSFEVRVCACPGRDRRTEEENFRKRGEPCPEPPPGSTKRALPTGTSSSPQPKKKPLDGEYFTLKIRGRARFEMFQELNEALELKDAQAEKEPGESRPHPSYLKSKKGQSTSRYKKIIFKREGPDSD

>AGC92786.1 p53 [Microtus oeconomus]

MEEPQSDLSIEPPLSQETFSDLWNLLPPNNVLSTSLSVDAMEDLFLSQDVANWLEEPNEGPQMSAAASTAEDPVTEAPAPVTPAPVTSWPLSSSVPSQKTYQGEYGFRLGFLHSGTAKSVTCTYSPSLNKLFCQLAKTCPVQLWVSSTPPPGTRVRAMAIYKKSQHMTEVVRRCPHHERCSDGDGLAPPQHLIRVEGNLRAEYLDDRQTFRHSVVVPYEPPEVGSDCTTIHYNYMCNSSCMGGMNRRPILTIITLEDPSGNLLGRNSFEVRVCACPGRDRRTEEENFRKKGEPRPELPLGSTKRVLPTNTSSSPQPKKKPLDGEYFTLKIRGRERFKMFSELNEALELKDAQDANGSGDSRAHSSYLKSKKGQSTSRHKKLMIKREGPDSD

>XP_032136321.1 cellular tumor antigen p53 isoform X1 [Sapajus apella]

METVSGSMRRAGPPTPPQPQPPSRDLWEAKKKSTDSLLLSFRLPENNILSPSLSQPVDDLMLSPDDIDIAQWFSQDPVPDEDPTVSEAPPTVAQAPAAPTSAAPTPAPSWPLSSSVPSQKTYRGDYGFRLGFLHSGTAKSVTCTYSPDLNKMFCQLAKTCPVQLWVDSTPPRGTRVRAMAIYKQSQHMTEVVRRCPHHERCSDSDGLAPPQHLIRVEGNLHVEYLDDKNTFRHSVVVPYEPPEVGSDCTTIHYNYMCNSSCMGGMNRRPILTIITLEDSSGNLLGRNSFEVRVCACPGRDRRTEEENFRKKGEPCLDLPPGSTKRAMPNSTSSSPQPKKKPLDGEYFTLQIRGRERFEMFRELNEALELKDAQAGKEPGGSRAHSSHLKSKKGQCTSRHKKLMVKREGPDSD

**>NP_035770.2 cellular tumor antigen p53 isoform a [Mus musculus]**

MTAMEESQSDISLELPLSQETFSGLWKLLPPEDILPSPHCMDDLLLPQDVEEFFEGPSEALRVSGAPAAQDPVTETPGPVAPAPATPWPLSSFVPSQKTYQGNYGFHLGFLQSGTAKSVMCTYSPPLNKLFCQLAKTCPVQLWVSATPPAGSRVRAMAIYKKSQHMTEVVRRCPHHERCSDGDGLAPPQHLIRVEGNLYPEYLEDRQTFRHSVVVPYEPPEAGSEYTTIHYKYMCNSSCMGGMNRRPILTIITLEDSSGNLLGRDSFEVRVCACPGRDRRTEEENFRKKEVLCPELPPGSAKRALPTCTSASPPQKKKPLDGEYFTLKIRGRKRFEMFRELNEALELKDAHATEESGDSRAHSSYLKTKKGQSTSRHKKTMVKKVGPDSD

>prf||1001197A antigen p53,tumor [Murid betaherpesvirus 1]

MTAMEESQSDISLELPLSQETFSGLWKLLPPEDILPSPHCMDDLLLPQDVEEFFEGPSEALRVSGAPAAQDPVTETPGPVAPAPATPWPLSSFVPSQKTYQGNYGFHLGFLQSGTAKSVMCTYSPPLNKLFCQLAKTCPVQLWVSATPPAGSRVRAMAIHKKSQHMTGVVRRCPHHERCSDGDGLAPPQHLIRVEGNLYPEYLEDRQTFRHSVVVPYEPPEAGSEYTTIHYKYICNSSCMGGMNRRPILTIITLEDSSGNLLGRDSFEVRVCACPGRDRRTEEENFRKKEVLCPELPPGSAKRALPTCTSASPPQKKKPLDGEYFTLKIRGRKRFEMFRELNEALELKDAHATEESGDSRAHSSYLKTKKGQSTSRHKKTMVKKVGPDSD

>XP_021032869.1 cellular tumor antigen p53 isoform X1 [Mus caroli]

MTAMEESQSDISLELPLSQETFSGLWKLLPPEDILPSPHSMDDLLLSQDVEEFFEGPSEALQVSRAPAAQDPVTETPGPVAPAPATPWPLSSFVPSQKTYQGNYGFHLGFLQSGTAKSVMCTYSPPLNKLFCQLAKTCPVQLWVSSTPPAGSRVRAMAIYKKSQHMTEVVRRCPHHERCSDGDGLAPPQHLIRVEGNLYAEYVEDRQTFRHSVVVPYEPPEVGSEYTTIHYKYMCNSSCMGGMNRRPILTIITLEDSSGNLLGRDSFEVRVCACPGRDRRTEEENFRKKEIPCPELPPGSAKRALPTCTSASPPQKKKPLDGEYFTLKIRGRERFEMFRELNEALELKDARATEESGDSRAHSSYLKTKKGQSTSRHKKTMVKKVGPDSD

>XP_021068233.2 cellular tumor antigen p53 isoform X1 [Mus pahari]

MTAMEDSQSDISLELPLSQETFSGLWKLLPPEVNLPTSVPSPNSMDELFLSPDVAELFEGPNEALHVLAAPAAQDPVTETPGPMAPAPATPWPLSSFVPSQKTYQGNYGFRLGFLQSGTAKSVTCTYSPPLNKLFCQLAKTCPVQLWVNATPPAGSRVRAMAIYKKSQHMTEVVRRCPHHERCSDGDGLAPPQHLIRVEGNLYAEYLEDRQTFRHSVVVPYEPPEVGSEYTAIHYKFMCNSSCMGGMNRRPILTIITLEDSSGNLLGRDSFEVRVCACPGRDRRTEEENFRKKEDPCPELPPGSAKRALPTCTSASPQQKKKPLDGECFILKIRGRERFEMFRELNEALELKDARAAEESGDSRAHSSYLKTKRGQSTSRHKKTMVKKVGPDSD

>XP_034361430.1 cellular tumor antigen p53 [Arvicanthis niloticus]

MTTMEDSQSDISIELPLSQETFSGFWKSIPSQDILSPSVLSPNPIEDLFLPQNIAELLEDPDEALQVAAAPVAQDTVTEAPALVAPTAATQWPLSSFVPSQKTYQGNYGFRLGFLQSGTAKSVMCTYSPPLNKLFCQLAKTCPVQLWVSATPPPGSRVRAMAIYKKSQHMTEVVRRCPHHERCSDGDGLAPPQHLIRVEGNLYAEYLDDRQTFRHSVVVPYEPPEVGSDYTTIHYKYMCNSSCMGGMNRRPILTIITLEDSCGNLLGRDSFEVRVCACPGRDRRTEEENFRKKEEHCPEPPPGSTKRALPTSTSSSPQQKKKPLDGEYFTLKIRGRERFEMFRELNEALELKDARAAEESGDSRAHSSYLKTKKGQSTSRHKKPMIKKMGPDSD

>XP_028635882.1 cellular tumor antigen p53 isoform X1 [Grammomys surdaster]

MTAMEDSQSDISIELPLSQETFSGLWKSILPQEDILSAAVLSPNPMEDLFLSQNVAELLEDPDEALQMAAAPPTQDPVTEAPAPVAPAPATQWPLSSSVPSQKTYQGNYGFRLGFLQSGTAKSVMCTYSPPLNKLFCQLAKTCPVQLWVSATPPAGSRVRAMAIYKKSQHMTEVVRRCPHHERCSDGDGLAPPQHLIRVEGNLYAEYLDDRQTFRHSVVVPYEPPEVGSDYTTIHYKYMCNSSCMGGMNRRPILTIITLEDSSGNLLGRDSFEVRVCACPGRDRRTEEENFRKKEEHCPELPPGSAKRALPTSTSSSPQQKKKPLDGEYFTLKIRGRSRFEMFRELNEALELKDARAAEESGDSRAHSSYLKTKKGQSTSRHKKPMIKKVGPDSD

>XP_032768227.1 cellular tumor antigen p53 [Rattus rattus]

MEDSQSDMSIELPLSQETFSCLWKLLPPDDILPTTATGSPNPMEDLFLPQDVAELLEGPEEALQVSAPAAQEPGTEAPAPVAPASATPWPLSSSVPSQKTYQGNYGFHLGFLQSGTAKSVMCTYSISLNKLFCQLAKTCPVQLWVTSTPPPGTRVRAMAIYKKSQHMTEVVRRCPHHERCSDGDGLAPPQHLIRVEGNPYAEYLDDKQTFRHSVVVPYEPPEVGSDYTTIHYKYMCNSSCMGGMNRRPILTIITLEDSSGNLLGRDSFEVRVCACPGRDRRTEEENFRKKEEHCPELPPGSAKRALPTSTSSSPQQKKKPLDGEYFTLKIRGRERFEMFRELNEALELKDAHAAEESGDSRAHSSYPKTKKGQSTSRHKKPMIKKVGPDSD

>NP_112251.2 cellular tumor antigen p53 [Rattus norvegicus]

MEDSQSDMSIELPLSQETFSCLWKLLPPDDILPTTATGSPNSMEDLFLPQDVAELLEGPEEALQVSAPAAQEPGTEAPAPVAPASATPWPLSSSVPSQKTYQGNYGFHLGFLQSGTAKSVMCTYSISLNKLFCQLAKTCPVQLWVTSTPPPGTRVRAMAIYKKSQHMTEVVRRCPHHERCSDGDGLAPPQHLIRVEGNPYAEYLDDRQTFRHSVVVPYEPPEVGSDYTTIHYKYMCNSSCMGGMNRRPILTIITLEDSSGNLLGRDSFEVRVCACPGRDRRTEEENFRKKEEHCPELPPGSAKRALPTSTSSSPQQKKKPLDGEYFTLKIRGRERFEMFRELNEALELKDARAAEESGDSRAHSSYPKTKKGQSTSRHKKPMIKKVGPDSD

>XP_031207308.1 cellular tumor antigen p53 isoform X1 [Mastomys coucha]

MVLLSEMTAMEYSDGIDLPLSQETFQRLWKLLPPEAVLSEASSNSMDNMFLSPDVVNLLEGPEEALQVSAAPAAQDPVTETPAPAAPAPATPWPLSSFVPSQKTYQGSYGFHLGFLQSGTAKSVMCTYSPSLNKLFCQLAKTCPVQLWVSDTPPAGSRVRAMAIYKKSQHMTEVVRRCPHHERCTDGDGLAPPQHLIRVEGNLNAEYLDDKQTFRHSVVVPYEPPEVGSDYTTIHYKYMCNSSCMGGMNRRPILTIITLEDSSGNLLGRDSFEVRICACPGRDRRTEEENFRKKEEPCPELPLGSAKRALPTGTSASPQQKKKRLDGEYFTLKIRGRERFEMFRELNEALELKDARAAEELGDSRAHSSYLKTKRGQSSSHHKKPMVKKVEPDSD

>XP_038184659.1 cellular tumor antigen p53 [Arvicola amphibius]

MEEPQSDLSIEPPLSQETFSDLWNLLPPNNVLSTSLSVDAMEDLFLSQDVANWLEEPSEGPQMSAATSTAEDPVTEAPAPVTPAPTTSWPLSSSVPSQKTYQGEYGFRLGFLHSGTAKSVTCTYSPSLNKLFCQLAKTCPVQLWVSSTPPPGTRVRAMAI

YKKSQHMTEVVRRCPHHERCSDGDGLAPPQHLIRVEGNLRAEYLDDRQTFRHSVVVPYEPPEVGSDCTTIHYNYMCNSSCMGGMNRRPILTIITLEDPSGNLLGRNSFEVRVCACPGRDRRTEEENFRKKGEPRPELPLGSTKRVLPTNTSSSPQPKKKPLDGEYFTLKIRGRERFKMFSELNEALELKDAQDANGSGDSRAHSSYLKSKKGQSTSRHKKLMIKREGPDSD

>XP_005349834.1 cellular tumor antigen p53 [Microtus ochrogaster]

MEEPQSDLSIEPPLSQETFSDLWNLLPPNNVLSTSLSVDAMEDLFLSQDVANWLEEPNEGPQMSAAASTAEDPVTEAPAPVTPAPVTSWPLSSSVPSQKTYQGEYGFRLGFLHSGTAKSVTCTYSPSLNKLFCQLAKTCPVQLWVSSTPPPGTRVRAMAIYKKSQHMTEVVRRCPHHERCSDGDGLAPPQHLIRVEGNLRAEYLDDRQTFRHSVVVPYEPPEVGSDCTTIHYNYMCNSSCMGGMNRRPILTIITLEDPSGNLLGRNSFEVRVCACPGRDRRTEEENFRKKGEPRPELPVGSTKRVLPTNTSSPQPKKKPLDGEYFTLKIRGRERFKMFSELNEALELKDAQDANGSGDSRAHSSYLKSKKGQSTSRHKKLMIKREGPDSD

>NP_001306217.1 cellular tumor antigen p53 [Nannospalax galili]

MEEQQSDLSIEPPLSQETFSDLWKLLPQNNVLSTPLSPNSMEDLLLSPEDVANWLDDPDEALQVPAAAITGDPVTETSAPVAPPPATPWPLSSSVPSQKTYQGSYGFRLGFLHSGTAKSVTCTYSPPLNKLFCQLAKTCPVQLWVDSTPPPGTRVRAMAIYKKSQHMTEVVKRCPHHERCSDSDGLAPPQHLIRVEGNLRAEYLDDKHTFRHSVVVPYEPPEVGSDCTTIHYNYMCNSSCMGGMNRRPILTIITLEDSSGNLLGRNSFEVRVCACPGRDRRTEEENFRKKGELCPELPPGSTKRALPTGTSSSPQPKKKPLDGEYFTLKIRGRERFEMFRELNEALELKDTQAEKDSGESRAHSSYLKSKKGQSTSRHKKLMIKREGPDSD

>XP_028725282.1 cellular tumor antigen p53 [Peromyscus leucopus]

MEEPQSDLSVEPPLSQETFSDLWKLLPQNNVLSTSLSSNSMEDLFLSQDVANWLEGPDETLQVSAATSAAEDPVPEAPLPVAPAPATPWPLSSSVPSQKTYQGKYGFRLGFLHSGTAKSVTCTYSPPLNKLFCQLAKTCPVQLWVSSTPPPGTRVRAMAIYKKSQHMTEVVRRCPHHERCSDGDGLAPPQHLIRVEGNLRAEYLDDRQTFRHSVVVPYEPPEVGSDCTTIHYNYMCNSSCMGGMNRRPILTIITLEDPSGNLLGRNSFEVRICACPGRDRRTEEENFRKKGEPCPELPPGSAKRALPTSTSSSPQPKKKTLDGEYFTLKIHGRERFEMFRKLNEALEFKDAHTAKGSGDSRAHSSYLKSKKEQSTSRHKKLMIKREGPDSD

>AQW35081.1 p53 [Eospalax fontanierii baileyi]

MEEPQSDLSIEPPLSQETFSDLWKLLPQNNVLSTSLSPNSMEDLLLSAEDVANWLDDPDDALRMPAAPVTEDPVTEASAPVAPPPATPWPLSSSVPSQKTYQGNYGFRLGFLHSGTAKSVTCTYSPCLNKLFCQLAKTCPVQLWVDSTPPPGTRVRAMAIYKKSQHMTEVVRRCPHHERCSDSDGLAPPQHLIRVEGNLRAEYLDDKHTFRHSVVVPYEPPEVGSDCTTIHYNYMCNSSCMGGMNRRPILTIITLEDSSGNLLGRNSFEVRVCACPGRDRRTEEENFRKKGESCPELPPGSTKRALPTDTSSSPQPKKKPLLDGEYFTLKIRGRERFEMFRELNEALELKDAQAEKESGESRAHSSYLKSKKGQSTSRHKKLMIKREGPDSD

>XP_036053163.1 cellular tumor antigen p53 isoform X1 [Onychomys torridus]

MATLQLFASILAGVAGIGTFPSHVLILKVLCWSPVPPAWLQAATGTCAPPASGSGTSFPAVTRSPEDPESCTEEPVVNRVVGRPSLTKARSSTFCVSSAGHQSLADLLGTAMEEPQSDLSIEPPLSQETFSDLWKLLPQNNVLSTSLTSNSMEDLFLSQDVANWLEDPDEALQVSAATSAAQDPVPEAPVPVAPAPATPWPLSSSVPSQKTYQGKYGFRLGFLHSGTAKSVTCTYSPPLNKLFCQLAKTCPVQLWVTSTPPPGTRVRAMAVYKKSQHMTEVVRRCPHHERCSDGDGLAPPQHLIRVEGNLRAEYLDDRQTFRHSVVVPYEPPEVGSDCSTIHYNYMCNSSCMGGMNRRPILTIITLEDPSGNLLGRNSFEVRICACPGRDRRTEEENFRKKGEPCPELPPGSAKRALPTNTSSSPQPKKKTLDGDGGYFTLKIHGRERFEMFRKLNEALEFKDAHAAKGSGDSRAHSSYLKSKKEQSTSRHKKLMIKREGPDSD

>NP_001230905.1 cellular tumor antigen p53 [Cricetulus griseus]

MEEPQSDLSIELPLSQETFSDLWKLLPPNNVLSTLPSSDSIEELFLSENVTGWLEDSGGALQGVAAAAASTAEDPVTETPAPVASAPATPWPLSSSVPSYKTYQGDYGFRLGFLHSGTAKSVTCTYSPSLNKLFCQLAKTCPVQLWVNSTPPPGTRVRAMAIYKKLQYMTEVVRRCPHHERSSEGDSLAPPQHLIRVEGNLHAEYLDDKQTFRHSVVVPYEPPEVGSDCTTIHYNYMCNSSCMGGMNRRPILTIITLEDPSGNLLGRNSFEVRICACPGRDRRTEEKNFQKKGEPCPELPPKSAKRALPTNTSSSPPPKKKTLDGEYFTLKIRGHERFKMFQELNEALELKDAQASKGSEDNGAHSSYLKSKKGQSASRLKKLMIKREGPDSD

>NP_001268590.1 cellular tumor antigen p53 [Mesocricetus auratus]

MEEPQSDLSIELPLSQETFSDLWKLLPPNNVLSTLPSSDSIEELFLSENVAGWLEDPGEALQGSAAAAAPAAPAAEDPVAETPAPVASAPATPWPLSSSVPSYKTYQGDYGFRLGFLHSGTAKSVTCTYSPSLNKLFCQLAKTCPVQLWVSSTPPPGTRVRAMAIYKKLQYMTEVVRRCPHHERSSESDGLAPPQHLIRVEGNMHAEYLDDKQTFRHSVVVPYEPPEVGSDCTTIHYNYMCNSSCMGGMNRRPILTIITLEDPSGNLLGRNSFEVRICACPGRDRRTEEKNFQKKGEPCPELPPKSAKRALPTNTSSSPQPKRKTLDGEYFTLKIRGQERFKMFQELNEALELKDAQALKASEDSGAHSSYLKSKKGQSASRLKKLMIKREGPDSD

>NP_001075873.1 cellular tumor antigen p53 [Oryctolagus cuniculus]

MEESQSDLSLEPPLSQETFSDLWKLLPENNLLTTSLNPPVDDLLSAEDVANWLNEDPEEGLRVPAAPAPEAPAPAAPALAAPAPATSWPLSSSVPSQKTYHGNYGFRLGFLHSGTAKSVTCTYSPCLNKLFCQLAKTCPVQLWVDSTPPPGTRVRAMAIYKKSQHMTEVVRRCPHHERCSDSDGLAPPQHLIRVEGNLRAEYLDDRNTFRHSVVVPYEPPEVGSDCTTIHYNYMCNSSCMGGMNRRPILTIITLEDSSGNLLGRNSFEVRVCACPGRDRRTEEENFRKKGEPCPELPPGSSKRALPTTTTDSSPQTKKKPLDGEYFILKIRGRERFEMFRELNEALELKDAQAEKEPGGSRAHSSYLKAKKGQSTSRHKKPMFKREGPDSD

>XP_041520530.1 cellular tumor antigen p53 isoform X1 [Microtus oregoni]

MEEPQSDLSIEPPLSQETFSDLWNLLPPNNVLSTSLSVDAMEDLFLSQDVANWLEEPNEGPQMSAAASTAEDPVTEAPAPVTPAPVTSWPLSSSVPSQKTYQGEYGFRLGFLHSGTAKSVTCTYSPSLNKLFCQLAKTCPVQLWVSSTPPPGTRVRAMAIYKKSQHMTEVVRRCPHHERCSDGDGLAPPQHLIRVEGNLRAEYLDDRQTFRHSVVVPYEPPEVGSDCTTIHYNYMCNSSCMGGMNRRPILTIITLEDPSGNLLGRNSFEVRVCACPGRDRRTEEENFRKKGEPRPELPLGSSKRVLPTNISSSPQPKKKPLDGEYFTLKIRGRERFKMFSELNEALELKDAQDANGSGDSRAHSSLQPCPRRKAQAAASCHTGSLLPSVFLQLPEVQEGPVNLPS

>XP_004638481.1 cellular tumor antigen p53 [Octodon degus]

MEEPQSDLSLEPPLSQETFSDLWKLLPDNNVLSSSLPSTMDDLLLPDVVNWLEENPDEDIQVSAAPVPQPPTPVAPAPAAPVPATSWPLSSSVPSHKPYHGNYGFRLGFLQSGTAKSVTCTYSPDLNKLFCQLAKTCPVQVWVESPPPPGTRVRALAIYKKSQHMTEVVRRCPHHERCSDSDGLAPPQHLIRVEGNLHAEYLDDRTTFRHSVVVPYEPPEVGSDCTTIHYNYMCNSSCMGGMNRRPILTIITLEDSSGKLLGRNSFEVRVCACPGRDRRTEEENFRKKGGSCSEPTPGSIKRALPPTTSSSPQPKKKPLDGEYFTLKVRGRERFEMFRELNEALELKDAQTEKEQGESRPHSSYLKSKKGQSTSCHKKLMCKREGPDSD

>XP_022451328.1 cellular tumor antigen p53 [Delphinapterus leucas]

MEESQAELGVEPPLSQETFSDLWKLLPENNLLSSELSPAVDDLLLSPEDVANWLDERPDEAPQMPEPPAPAAPTPAAPAPATSWPLSSFVPSQKTYPGSYGFHLGFLHSGTAKSVTCTYSPALNKLFCQLAKTCPVQLWVSSPPPPGTRVRAMAIYKKSEYMTEVVRRCPHHERCSDYSDGLAPPQHLIRVEGNLRAEYLDDRNTFRHSVVVPYEPPEVGSDCTTIHYNFMCNSSCMGGMNRRPILTIITLEDSNGNLLGRNSFEVRVCACPGRDRRTEEENFHKKGQSCPELPTGSAKRALPTGTSSSPPQKKKPLDGEYFTLQIRGRERFEMFRELNEALELKDAQAGKEPGESRAHSSHLKSKKGQSPSRHKKLMFKREGPDSD

**>NP_001081567.1 cellular tumor antigen p53 [Xenopus laevis]**

MEPSSETGMDPPLSQETFEDLWSLLPDPLQTVTCRLDNLSEFPDYPLAADMTVLQEGLMGNAVPTVTSCAVPSTDDYAGKYGLQLDFQQNGTAKSVTCTYSPELNKLFCQLAKTCPLLVRVESPPPRGSILRATAVYKKSEHVAEVVKRCPHHERSVEPGEDAAPPSHLMRVEGNLQAYYMEDVNSGRHSVCVPYEGPQVGTECTTVLYNYMCNSSCMGGMNRRPILTIITLETPQGLLLGRRCFEVRVCACPGRDRRTEEDNYTKKRGLKPSGKRELAHPPSSEPPLPKKRLVVVDDDEEIFTLRIKGRSRYEMIKKLNDALELQESLDQQKVTIKCRKCRDEIKPKKGKKLLVKDEQPDSE

>NP_001001903.1 cellular tumor antigen p53 [Xenopus tropicalis]

MEPSSETGMEPPLSQETFEDLWSLLPDPLQTGTGQMENFAEFSEYPLAPDMTVLQEGLMGNTVPTVTSSAVPSTEDYAGSYGLKLEFQQNGTAKSVTCTYSTDLNKLFCQLAKTCPLLVRVERPPPLGSILRATAVYKKSEHVAEVVKRCPHHERSVEPGDDPAPPSHLMRVEGNSKAYYMEDVGTGRHSVCVPYEGPQVGTECTTVLYNYMCNSSCMGGMNRRPILTIITLESPEGLLLGRRCFEVRVCACPGRDRRTEEDNCTKKRGLKPNGKRELSHPPSSDPPLPKKRLVEEDDEETFTLLIKGRSRYEMIKKLNDALELQESLDQQKLSIKCRKCRDEIKPKKGKKLLVKDELQDSE

>XP_040203199.1 cellular tumor antigen p53 [Rana temporaria]

MDPPSDNGMEPPLSQETFEDLWSLLPAMQTVNCPEVGFQDLDYPLPLPPTMTTLQEGGITSTFTPPSSTAVPSTEDYPGNHLLQLEFEQNGTAKSVTCTYSPDLNKLFCQSAKTCPVLIHVQAPPPSGSVLRAVAVYKKSEHMAEVVKRCPHHERSMEPGDDYAPRSHLIRVEGNSMAQYAEDVNGRHSVCLPYEEPQVGSQCTTILLNYMCNSSCMGGMNRRPIMTIITLESKEGVLLGRRCFEVRVCACPGRDRRTEEENFTKKKELKGSGKRAISNPQTLEVLIPKKRLVTEEEVFTLQIRGRERYDMLKKINDALECQDNKAEPQKLTLKCRKCRDEIKPKKGKKLLVKDELDSE

>XP_040271379.1 cellular tumor antigen p53-like [Bufo bufo]

MVLEAFVVGGGSAVHAGSWGIPNTRTTAALPPGTEYSFLPPMDPPSEGAMEPPLSQETFEDLWSLLPADNIAGVSCAEENLLDFEYPLPPVMSALQEGGVSSTFVPAPHSAVPSTEDYPGLHGLSLEFQQNGTAKSVTCTYSPVLNKLFCQSAKTCPVLIRVPNAPPGGSVLRAVAVYKKSEHVAEVVRRCPHHERSLEPGDDSAPRNHLIRVEGNSRACYAEDTNGRHSVCVSYEEPQVGCEFTVLLLNYMCNSSCMGGMNRRAIMTIITLESKEGYLLGRRCFEVRICACPGRDRRTEEENCLKKELKTSLKRAVQGSPVSDAALPKKKKDLNEEETFTLQVKGRERYEMLKKINDALELQDKVDQQILTLKCRKCRDDIKPKKGKKLLVKDEQDSE

>XP_030043586.1 cellular tumor antigen p53 isoform X1 [Microcaecilia unicolor]

MDLYLENADTYFFRDLFSSCLENASPDTCYCSSSDPPLACCCPPSLIMMSETVSENGLEENFSQESFSDLWNCLPNLSPTLPENENDFWLMDQFPLQTHVEATLDPDLLPQEEGLLVQELEAVSQHVVLLSPEANSVATSSIVPSTEDYPGVLDFRLAFQQSGTAKSVTCTYSPDLNKLFCQLSKTCPVQIKVKETPPAGALIRATAVYKKSEHVAEVVKRCPHHERTAEPMEDTASRNHLIRVEGNQLARYTENANTHRHSVDVPYEAPQVGSDFTTVLFNYMCNSSCMGGMNRRPILTIITLETKEGQLLGRRSFEVRICACPGRDRKTEEANSQRKVGKCGSSKRVIQDVSQAATPPENRKKRVTSSDEEIFTLQVRGRQRYEFIKTIHDALELQDYIPQQQMEKIKQQKQLKSRKERDRIAPKKGKKLLVKDEAPDSD

>XP_029436808.1 cellular tumor antigen p53 isoform X1 [Rhinatrema bivittatum]

MRVEGAQPIEGAFFSLSCFLIGRPDVMGVPTDRFRAPDKNDSCAGGLFSLWTAMDLYPEDADTHPFGDLFPPCLETVFSEEGHCSTPELSLACCCPEGHPPALAMMSVSENGLEEPLSQESFSTLWNLLPNLPASLENGNNELWQMETEQFLTDTALYPEVPDHELGGCLLPEAFSLEANSVTTSSTVLSTEDYPGEHNFRLAFQQSGTAKSVTCTYSPELNKLFCQLSKTCPIQIKVQDMPPAGALIRATAVYKKSEHVAEVVKRCPHHERSTDPMDDAAPPSHLIRVEGNQLAKYSENISTRRHSVYVPFEAPQVGSDCTTVLYNYMCNSSCMGGMNRRPILTIVTLESKEGQLLGRRSFEVRICACPGRDRKSEEENCQKKTMGKSGSSKRAIREVSQAVACTESRKKQVTSSDDEIFTLQVRGRDRYEFLKKINDAFECQDYVSQQELEKIKQQKQLVKSRRERDTVAPKKGKRLLQKDETQDSD

>XP_033781307.1 cellular tumor antigen p53 isoform X1 [Geotrypetes seraphini]

MDLYPEDANTHTFRDLFSSCLENASLDMWYFSTTEPPLACRCPNCCPLSLLTMSEAVSETGLDEAFSQESFSDLWNGLPNLSSPLLGHGNDLWSDLEPFSLHVSMVAPQPLIEEESMLVQELDSAPPSEVPLSPEANSVATSSIVPSTEDYPGDHEFLLNFQQSGTAKSVTCTYSPELNKLFCQLTKTCPVQIKVKGTPPAGALIRATAVYKKSEHVAEVVKRCPHHERSTESVEDTAPRNHLIRVEGNHLARYTENANTHRHSVAVPYEAPQVGSDFTTVLYNYMCNSSCMGGMNRRPILTLITLETKEGQLLGRRSFEVRICACPGRDRKMEEGNSQRKIGKSGNSKRAILDVSQAPVHPEKKRVTSSDEEIFTLQVRGRQRYELIKTIHDALELQDYIPQQQMEKIKQQKQLKTRRERDMVAPKKGKKLLVKDEAPDSD

>AEI91929.1 P53 [Cynops orientalis]

MVESEAAMDSLPLSQETFSQLWGSLPETLLPTTDDFFWQQDFDCTLGMDPHNQLQQEDVSSTLNNDLLRAYEVALPEALPLEPPAATSAVPSTEDYPGTYNFRLAFQQSGTAKSVTCTYSPDLNKLFCQLAKTCPVKMTVSTPPPPGAFVRATAVYKKSQHIAEVVKRCPHHERSGDPSDGTAPSNHLIRVEGNYKAEYKESGATRRHSVLVPYETPQVGSDCTTVLYNYMCNSSCMGGMNRRPILTIITLETNDGQLLGRCCFEVRVCACPGRDRKTEEENFHNKKKPSKNAVNTGTKRTIKDASLTETDVENPKRKAHEEDEIYTLQIRGRERYEMLKKLNDALELKDMIPQSDQEKIKQKFQKPRKERENPAPKNGKKLLVKDESQDSD

>XP_006112198.1 cellular tumor antigen p53 isoform X1 [Pelodiscus sinensis]

MEPILDTGLDLPLSQESFSDLWKTLSPLAEEYLVKPAEPVSQELFTLPDMSLGLSGSADSSLLLPQAGHSYESWDLPCLALEPPPTSSTVPSTEDYAGEHDFQLAFQQSGTAKSVTCTYSPDLNKLFCQLAKTCPVQIKVSSQPPPGSVIRATAVYKKSEHVAEVVRRCPHHERSADYSDGVAPAQHLIRIEGNHQAYYRDDENTKRQSVTVPYETPQVGSDCITVLYNFMCNSSCMGGMNRRPILAIITLESKNGQLLGRRCFEVRVCACPGRDRRTEEENHRKKLSCRALSGSVVLKESKAKRTLQATMEASENPKKRTVSSDKEVFCLEVHGRENYEMLKKINNALEVAAARPQGELETQRNPKAWLKTRKERGDGPLPQSGKKLLVKEEDSE

>XP_038242058.1 cellular tumor antigen p53 isoform X1 [Dermochelys coriacea]

MHPGPPGNPQLVHVDAKCLGETGVEISAEGKARMESMLDPGLEPPLSQESFSDWWSMMLQPTNVDPTALENQELFSLPDPDLGLSDSADPSLLLPQAGGSDEGWELPGPAPEPPPTSSTVPSTEDYAGEHGFELTFQQSGTAKSVTCTYSPELNKLFCQLAKTCPVQIKMASQPPPGSIVRATAVYKKSEHVAEVVRRCPHHERCLEYSDGVAPARHLIRIEGNQQAHYHDDENTKRQSVTVPYEMPQVGSDCTTVLYNFMCNSSCMGGMNRRPILAIITLEGKHGQLLGRRCFEVRVCACPGRDRRTEEENFHKKLTGRVLSGSGTLKGARAKRALQTTMETAENPKKRVVSPEKEVFLLEVHGRKRYMMLKEINDALEMASAKQQEEPESHRNPTPTRLLKTRKEPGDGLLPRSGKRLLVKEEDSE

>XP_028654218.1 cellular tumor antigen p53 isoform X1 [Erpetoichthys calabaricus]

MTEVGSDTLEPLSQESFQDLWNMVTIPDQVTGESEWLDNALITGDMDTLQHGYISSSDELFSAVPFEAITSVPLGLASNDTGAPSASTVPSTKDYPGDVGFKLRFQQSSTAKSVTCTYSPVLNKLFCQLAKTCPVQMTVDKPPPPGTLIRATAVYKKSEHVADVVRRCPHHEQTAENNEGPAPRSHLIRVEGNHQAEYLDDMNTKRQSVTVPYETPQLGSDCITVLYNYMCNSSCMGGMNRRPIITIVTLETKEGLLIGRRCFEVRVCACPGRDRKSEEENCHKQQENATCKSSSGTKRNIKDVTQPISHPESSKKNKSSSDEEVFILQVRGRERYEMLKRINDSLELKDVIPHTDLEKYRQKVILKNSNRKDREKVEPKEGKKLLVKEEKSDSD

>QCO84576.1 tumor protein suppressor 53 [Latimeria menadoensis]

MLGNSPARVECEPGGSGQGSRGIRVLPEARIGETVSFSCSCGNGVLPEATMTDPMSESGLEPLSQESFADLWNLLPTSMSNNVDLLPEETWQAELDDLRLPGEMGAPLQTMTVSSNFGAFLESELEAGLASAAPPPPAPPAPAGESIPVTSSVPSTEDYAGDHNFQLIFQQSSTAKSVTCTYSFTLNKLFCQLAKTCPVQIKVDAAPPPGALIRTIAIYKKSEHIAEVVRRCPHHERCADLNDGLAPASHLIRVEGNRLAQYIEDRNTQRQCVIIPYECPQVGSECSTVLYNFMCNSSCMGGMNRRPILTIITLETKEGRLLGRRCFEVRVCACPGRDRKTEEENSHKQQEKANPKTAIKRALREVSCSDSGPYKKRAVNPEEELYSLQVRGRERYEMFKKLNNALELQDSVSAAETEKHKTKPSKNASRKELEPKKGKKLLVKEEGSGGGGGGGGGNDSD

>ABI33869.1 tumor suppressor p53 [Ambystoma mexicanum]

MDSLPLSQQTFSELWRSLPETLLQTTDDFWPAELCYNLDMEQPPQLQQEDVSSTLSCDLLEAYHGHVPGTLPPETLAAATSAVPSTEDYPGAYDFKLVFQQSGTAKSVTCTYSPDLNKLFCQLAKTCPVQMTVSSPPPPGAILRATAVYKKSQHVAEVVKRCPHHERTVDPNDDSVPVPADHLIRVEGNFRAEYKMNSTTHRHSVVVPYETPQLGSGCTTVLYNYMCNSSCMGGMNRRPILTIITLETSDEQLLGRRCFEVRVCACPGRDRKTEEENFHNKKKPTKPSGGSSSKRTIRDVTATEPDTESPKRKASEEETFTLQIHGRERYEMFKKLNEALELKDMIPQTDLEKIKQQKSQKTKKERDPPAPKNGKKLLVKDETQDSD

>XP_037743179.1 cellular tumor antigen p53 [Chelonia mydas]

MEPMLDPGLEPPLSQESFSDWWSMMLQPTNVDPTAPENQELFSLPDPDLGLGLSDSADPSLLLPQAGGSDEGWEFPGPAPEPPPTSSTVPSTEDYAGEHGFELTFQQSGTAKSVTCTYSPELNKLFCQLAKTCPVQIKMASQPPPGSIVRATAVYKKSEHVAEVVRRCPHHERCMEYSDGVAPARHLIRIEGNQQAHYHDDENTKRQSVTVPYETPQVGSDCTTVLYNFMCNSSCMGGMNRRPILAIITLEGKHGQLLGRRCFEVRVCACPGRDRRTEEENFQKKLTGRVLSGAGTLKGARAKRALQASMETAENPKKRVVSPEKEVFLLEVRGRKRYMMLKEINDALEMVAAKQQGEPESHRNPTPSRLLKTRKEPGDGLLPRSGKRLLVKEEDSE

>XP_018586898.1 cellular tumor antigen p53 isoform X1 [Scleropages formosus]

MATPTLPTALGDSPAHGRFFIEKTHTEGRKKRKNKNSEIGVGPPGTGTRRPDDSLATPVCPSCFWKSVSNSITDNMAEEDMPSLPFSQESFQELWKDLMPAPSGDMDWQNMNSDLQLLSDTTFDEGLFEIPSAVPPQASVSPPECGAPPSSTVPTTTDYPGDLGFQLRFQQSSTAKSVTCTYSPDLNKLYCQLAKTCPVQMVVNQKPPLGSVLRATAVYKKSEHVADVVRRCPHHERTAENNEGPAPRGHLIRVEGNQLAQYMEDANTRRQSVVVPYEAPEVGAECTTVLYSYMCNSSCMGGMNRRPILTIITLETGDGQLLGRRSFEVRVCACPGRDRKTEEENFRKQQEKNVPKAPSGGTKRALKEPSQPASHPEGSKKTKCVSSGEEEIYTLHVRGRERYEMFKKINESLELGDLVPLADQEKYRQKGHSKNKRDKERDAPEPKRGKKLLVKEEKSDSD

>XP_027712839.1 cellular tumor antigen p53 [Vombatus ursinus]

MEESLSDLEPPLSQETFSDIWNLQWNNMPDDLSNIDDIQFASADDINWLRNEEENPGFQVPAPPVNLPTSSTVPSRKDYPGEYGFKLGFLHSGTAKSVTCTYSPELNKLFCQLAKTCPVQLWVTSPPPAGSRVRAMAIYKKSEHMTEVVKRCPHHERCTEHIDALAPPQHLIRVEGNLRAEYLNDATTMRQSVSIPYESPEVGCDCTTIHYNYMCNSSCMGGMNRRPILTIITLEDSSGQLLGRRSFEVRVCACPGRDRRTEEENFRKKGGHNPQPSSESNKRALPTTPSTPKAKKKLVDGEYFTLQIRGRHRYELFRELNEALELKDAHSGKEAEGSCPHRSQLKSKKGDSTPSQGKRLLVKSEVPDSD

>XP_039602918.1 cellular tumor antigen p53 isoform X1 [Polypterus senegalus]

MTEVGSDTLEPLSQESFQDLWNMVAIPDQVTGDSEWLDNALITDDMDTLQHGYISSSNELFSGVPFEAINPVPSGLASNDTGAPSASVVPSTKDYPGDVGFKLRFQQSSTAKSVTCTYSPVLNKLFCQLAKTCPVQMTVDKPPPPGTLIRATAVYKKSEHVADVVRRCPHHEQTTENNEGPAPRSHLIRVEGNHQAEYWDDLNTKRQSVTVPYETPQLGSDCITVLYNYMCNSSCMGGMNRRPIITIVTLETKEGLLIGRRCFEVRVCACPGRDRKSEEENCHKQQENASCKSSSGTKRNIKDVTQPISHPESSKKNKSSSDEEVFILQVRGRERYEMLKRINDSLELKDVIPHTDLEKYRQKLNLKNSNRKDREKVEPKEGKKLLVKEEKSDSD

>XP_035289590.1 cellular tumor antigen p53 isoform X1 [Anguilla anguilla]

MTEQEGTGALEYSLSQDETFQALWNTVTVPTEDEGWGSLDNVSVPDALNCDHLQFQQYLVGSPLQDGFDEKLFDVPATPAPPQPSGSALDSGAPPASTVPTTTDYPGEFGFLLRFQKSSTAKSVTCTYSPELNKLFCQLAKTCPVQMQVTHTPPPGAVLRATAVYKKSEHVAEVVRRCPHHERTSENNDDSTPRSHLIRVEGSQRAWYTEDSNTLRHSVLVPYEPPQLGSECTTVLYNFMCNSSCMGGMNRRPILTIITLETQEGQVLGRRSFEVRVCACPGRDRKTEEDNHRKQQEKSGTKTGGGAKRTFKESSLPSSQPEDSKKTKTTSSNEEEIFTLQVRGKKRFEMLKMINDSLELKDLVPAADQDKYRQKLHSKSTSKREREREGVEPKKGKRLLVKEEKSDSD

>XP_036622872.1 cellular tumor antigen p53 isoform X1 [Trichosurus vulpecula]

MEESLSDLEPPLSQETFSDIWNLQWNNLPDDLSNIDDIPFASPDNMSWLGNEEESPGLPVSAPPVNLPTSSTVPSQKDYPGEYGFKLGFLQSGTAKSVTCTYSPELNKLFCQLAKTCPVQLWVTSPPPAGARVRAMAIYKKSEHMTEVVKRCPHHERCTEHIDALAPPQHLIRVEGNLRAEYLSDATTMRQSVSVPYESPEVGCDCTTIHYNYMCNSSCMGGMNRRPILTIITLEDSSGQLLGRRSFEVRVCACPGRDRRTEEENFRKKGGHNLQPPSESNKRGMALPTAPGSTPKTKKKLMDGEYFTLQIRGRQRYELLREINEALELKDAHSGKEPEGSCPHRSQLKSKKGDSTPCQGKRLLVKSEVPDSD

>XP_020822126.1 cellular tumor antigen p53 [Phascolarctos cinereus]

MEESLSDLEPPLSQETFSDIWNLQWNNMPDDLSNIDDIQFASPDDINWLGNEEENPGLQVPAPPVNLPTSSTVPSRKDYPGEYGFRLGFLHSGTAKSVTCTYSPELNKLFCQLAKTCPVQLWVTSPPPAGARVRAMAIYKKSEHMTEVVKRCPHHERCTEHIDALAPPQHLIRVEGNLRAEYLNDATTMRQSVSIPYESPEVGCDCTTIHYNYMCNSSCMGGMNRRPILTIITLEDSSGQLLGRRSFEVRVCACPGRDRRTEEENFRKKGGHNPQPSSESNKRALPTTPSSTPKAKKKLMDGEYFTLQIRGRHRYEVFRELNEALELKDAHSGKEAEGSCPHRSQLKSKKGDSTPSQGKRLLVKSEVPDSD

>MBN3310676.1 P53 protein [Amia calva]

PDSLPPVSSTVPSTTDYPGELGFQLVFQQSGTAKSVTCTYSPDLNKLFCQLAKTCPVQVLVRHPPPPGAILRATAVYKKSEHVADVVRRCPHHERTPDNNEGPAPPGHLIRVEGNQLAQYMEDGHTHRQSVLVPYETPQLGSECTTVLYNFMCNSSCMGGMNRRPILTIITLESKEGQLLGRRCFEVRVCACPGRDRKTEEENFRKVQEKSSAKPAGAAKRTIKEVTQAAPRPEVSKKTKSSPSTEEEVFTLQVRGRERFEMLKKINESLELKDLVPATELDKYRQKLHSKSGGRKDRERDGVEPKKGKRLLVKEEKTDSE

>XP_024074382.1 cellular tumor antigen p53 [Terrapene carolina triunguis]

MEPMLDPGLEPPLSQESFSDFWSNIWYPTNADSTATESQRLSSLPDPDPDLALGLALGLSGSGDPSLLLPQAGGSDGGWELPGPAPEPPPTSSTVPSTEDYAGEHGFELVFQQSGTAKSVTCTYSPELNKLYCQLAKTCPVQIKTASQPPPGSVVRATAVYKKSEHVAEVVRRCPHHERCEDNRDGVAPARHLIRIEGNQQAHYYDDENTKRQSVMVPYETPQVGSDCTTVLYNFMCNSSCMGGMNRRPILAIITLEGRHGQLLGRRCFEVRVCACPGRDRRTEEENFRKKLAGRVLSGAGALKGGRAKRALQATMETAENPKKRVVSTEKEVFLLEVRGRKRYMMLKEINDALEMVAAKQQGEPESHRNPMPSRLLKTRKESGDELLPQSGKKLLVKEEDSE

>XP_039355958.1 cellular tumor antigen p53 isoform X1 [Mauremys reevesii]

MGRRASWGERWRHRSRWTCGHVAKTAAQCILAPGNPQLVHVDARCSGETGVQISAEGTAAMEPMLDPGLEPMSDPGLEPPLSQESFFDLWNMMELRTRKDSTTQDCQGLSSLPDPELSLALGLSDSADPSLLLPQAGGSDGGWELPDPAPEPPPTSATVPSTEDYAGEHGFELAFQQSGTAKSVTCTYSPALNKLYCQLAKTCPVQIRTASQPPAGAVIRATAVYKKSEHVAEVVRRCPHHERCEEYRDGVAPARHLIRIEGNQQAHYYDDENTKRQSVTVPYETPQVGSDCTTVLYNFMCNSSCMGGMNRRPILAIITLEGRHGQLLGRRCFEVRVCACPGRDRRTEEENSCKKLSGRVLNGAGAHKGVRAKRALQATMETAENPKKLVVSSEKEVFFLEVHGRKRYMMLKEINDALEMAAAKQQGEPESHRNATPSRLLKTRKESADGMLPRSGKKLLVKEEDSE

>XP_030403970.1 cellular tumor antigen p53 [Gopherus evgoodei]

MRTKFREKITSLHLLLQIPPVIRPTSVVALLATASPWELVFSCYSSMPPKSLFQSPRFPGSSPPPYGDAGLMGPAVTWARQARNACWPPGNPQLVHVDTKCPGETGVQISAEGTAGMEPMLDPGLEPMLDPGLEPPLSQESFSDFWSMMARRTAQDSTTQDSQGLFSLPDPDLSLALGLSDSADPSLLLPQAGGSDGGWGLPDPAPEPPPTSATVPSTEDYAGEHGFELAFQQSGTAKSVTCTYSPQLNKLYCQLARTCPVQIRTASQPPAGSVIRATAVYKKSEHVAEVVRRCPHHERCEEYRDGVAPARHLIRIEGNQQAHYYDDENTKRQSVTVPYEMPQLGSDCTTVLYNFMCNSSCMGGMNRRPILAIITLEGRHGQLLGRRCFEVRVCACPGRDRRTEEENFCKKLAGRVLNGAGAHKGGRAKRALQATMEAAENPKKLVVSSEKEVFLLEVHGRKRYMMLKEINDALEMAAAKQQGEPESHRNATPSRLLKTRKESADGVLPRSGKKLLVKEEDSE

>XP_031821303.1 cellular tumor antigen p53 isoform X1 [Sarcophilus harrisii]

MSSHTLGRAPLRLTQPGVDSTFAFGGGILSLPSPLAECHKTAFLRSTMEESLSDLEPPLSQETFSDLWKLQWNNLPDDLSNIDDIQFASPDSINWLENEEENPGFQVSLPPVNLPTSSTVPSQKDYPGDYGFRLGFLQSGTAKSVTCTYSPELNKLFCQLAKTCPVQLWVTSPPPAGARVRAMAIYKKSEHMTEVVKRCPHHERCTEHKDALAPPQHLIRVEGNLRAEYLSDLTTKRQSVSVPYESPEVGCDYTTVHYNYMCNSSCMGGMNRRPILTIITLEDSSGQLLGRRSFEVRVCACPGRDRRTEEENFRKKGGHNPQPSSESNKRALPTTPSSTPKTKKKLMDGEYFTLQIRGRHRYELLRELNEALELKEAHSGKDPEGNCPHRSQLKSKRGDSTPCQGKRLLVKSEVADSD

>XP_027035235.1 cellular tumor antigen p53 isoform X1 [Tachysurus fulvidraco]

MEGNRERESKMTESSDSQEFAELWLKNLMGVEESGVPDDNSWENEEQIPEDLQDVLLGDILQPQSSSSPPTSTVPVTSDYPGLHNFTLHFQKSSTAKSVTCTYSPELNKLFCQLAKTCPVLMAVSFSPPHGSVLRATAVYKRSEHVADVVRRCPHHERSNDNNEGPAPPGHLLRVEGNSRAVYHEDLNTQRHSVVVPYEPPQVGSECTTVLYNYMCNSSCMGGMNRRPILTIITLETQDGQLLGRRTFEVRVCACPGRDRKSEENNFRKQQESKTSGKTLTKRSIKDPPSHPEDSKKSKNTSSDDEIYTLQVHGRERYEFLKKINDGLELSDLVPPADQEKYRQKLLSKACRKERDGAAAEPKRGKKRLVKEEKSDSD

>XP_005279396.2 cellular tumor antigen p53 [Chrysemys picta bellii]

MRRQASGAPCRDGGSGLTGPAVTWPKQPRNASWPPGNPQLVHVDAKCPGETGVEISAEGTAGMEPMLDPGLEPPLSQESFSDFWSNIWYPTNADSTATESQRLSSLPDPDPDLALGLALGLSGSGDPSLLLSQAGGSDGGWELPGPAPEPPPTSSTVPSTEDYAGEHGFELVFQQSGTAKSVTCTYSLELNKLYCQLAKTCPVQIKTASQPPPGSVVRATAVYKKSEHVAEVVRRCPHHERCEEYRDGVAPARHLIRIEGNQQAHYYDDENTKRQSVTVPYETPQVGSDCTTVLYNFMCNSSCMGGMNRRPILAIITLEGRHGQLLGRRCFEVRVCACPGRDRRTEEDNFRKKLAGRVLSGAGALKGGRAKRALQATMETAENPKKRVVSAEKEVFLLEVHGRKRYMMLKEINDALEMVAAKQQGEPESHRNPTPSRLLKTRKESGDELLPQSGKKLLVKEEDSE

>QGQ62196.1 tumor protein 53 [Ameiurus nebulosus]

MEGDEEDRERDTMMAEHPVSEEFAELWLQNLIVRDNSSWGNEGLIPDDLQDVPCELLLSDMLQPQSSSSPPTSTVPVTSDYPGLHDFTLHFQESSTAKSVTCTYSPGLNKLFCQLAKTCPVLMAVSSSPPPGSVLRATAVYKRSEHVAEVVRRCPHHERSNDSSDGPAPPGHLLRVEGNSRAVYQEDGNTQRHSVVVPYEPPQVGSQCTTVLYNYMCNSSCMGGMNRRPILTIITLETQNGHLLGRRTFEVRVCACPGRDRKTEESNFKKQQEPKTSGKTVTKRSMKDPPSHPEASKKSKNNSSDDEIYTLQVRGKERYEFLKRINDGLELSELVPPADQEKYRQKLLSKSCRKERDGAAAEPKRGKKRLVKEEKSDSD

>XP_036379425.1 cellular tumor antigen p53 [Megalops cyprinoides]

MNEQEEAGRLAFDNQETFQDLWNSVSVPGGDQNWNSLESYLLQGHLQEEFDEQLFDVPATPAPQPSVSALDSGAPPSSTVPTTTDYPGVLGFQLRFQQSSTAKSVTCTYSPDLNKLYCQLAKTCPVQMLVDSPPPPGAQLRATAVYKKSEHVAEVVRRCPHHERTSENNDGSTPPSHLIRVEGSQRTQYVEDSNTLRHSVLVPYEPPQLGSECTTVLYNFMCNSSCMGGMNRRPILTIITLETQEGQVLGRRCFEVRVCACPGRDRKTEEDNFRKLQEKTADKASTGTKRSFKETSQSAPRPEGSKKTKSSSSNEEEIFTLQIRGKERYEMLKKINESLELKDLVPAADLDKYRQKLHSKNNSKREKERDGVEPKRGKKLLVKEEKNSDSD

>XP_034771409.1 cellular tumor antigen p53 [Acipenser ruthenus]

MADPVSDSLEPLSQESFQELWNIVGTRGNEFWQENDDIFPELDGLETDAIGGSSSSAEVLLGGPTLVQQHPLPYVSETATTASTVPSTTDYPGELGFELRFQQSSTAKSVTCTYSPGLNKLFCQLAKTCPVQMHVRVPPPPGAVVRATAVYKKSEHVAEVVRRCPHHERTPENNEGPVPPGHLIRIEGNQLAQYVEDQRTRRQSVLVPYESPQVGSECTTVLYNYMCNSSCMGGMNRRPILTIVTLETSDGRLLGRRCFEVRVCACPGRDLKSEEENSRKQLEKAGSKASAGSTKRSIKEVTLPAPGSSKKSRTSSEEEVFTLQIHGRERYEMLKRINESLELKDLVPQSEQEKYREKLHKNGRKEREGLAPKRGKKLLIKEDKSDSD

>XP_036436707.1 cellular tumor antigen p53 isoform X1 [Colossoma macropomum]

MEPQAAMEEPSESQEFAELWLQNLMGQTPENSPWGNEEFPEDLQSMLDQPLFGEVSAEPPQPSTSPPAPTVPVATDYPGAHGFTLRFQQSGTAKSVTCTFSPELNKLYCQLAKTCPVQMEVNSPPPPGAVLRATAVYKKAEHVAEVVRRCPHHERTPENNDGLTPPGHLLRVEGNPRAQYQEDDNTLRHSVLVPYEPPQVGSEYTTVLYNYMCNSSCMGGMNRRPILTIVTLETQDGQLLGRRSFEVRVCACPGRDRKTEESNFRKLQEAKNTNKTPATNKRSLKEPTSRPEASKKAKTSSSSDEEIYTLQVRGRERYELLKKINDGLELSDVVPPADAEKYRQKLHSKSSRKERDGAPEPKRGKKLLVKEEKSDSD

>XP_030647985.1 cellular tumor antigen p53 [Chanos chanos]

MAEQSESQEDFAELWKLITQPNGEETSYDDYLQDGFDAELFEDVSSQPPQPSMSTPPPDGGSPPSSTVPTTTDYPGTHGFHLRFQQTSTAKSVTCTYSSELNKLYCQLAKTCPVQMVVDVLPPAGAMLRATAVYKKSEHVAEVVRRCPHHERTPENNEGPAPPGHLIRVEGNARAVYQEDCHTRRQSVLVPYEPPQLGSECTTVLYNYMCNSSCMGGMNRRPILTIITLETQEGALLGRRCFEVRVCACPGRDRRTEEGNYRKLLESKNSSKTSTSTKRIPRPEVSKRAKCSSSSDPEIFTLQVRGRDRYEMLKKINDSLELNDLVPPADREKYRQKLVSKPKKERDGSAPAPKKGKKLMMKSEKSDSD

>ARH02602.1 TP53 [Notamacropus eugenii]

MEESLSDLEPPLSQETFPDLWNLQWNNLPDDLSNIDDKYFAPPDSGTWLGNEEESLSVQVPVPPVSLPTSSTVPSRKDYPGDYGFRLGFLQSGTAKSVTCTYSPELNKLFCQLAKTCPVQLWVTSPPPAGARVRAMAIYKKSEHMTEVVKRCPHHERCTEHIDALAPPQHLIRVEGNLRAEYLNDATTMRQSVSIPYESPEVGCDCTTIHYNYMCNSSCMGGMNRRPFILTIITLEDSSGQLLGRRSFEVRVCACPGRDRRTEEENFRKKGGHNPQPPSESNKRALPTTPSSTPKGKKKLMDGEYFTLQIRGRHRYELLREINEALELKDAHSGKEPEGSCHHHSQLKSKKGDSTPCQGKRLLVKSEVADSD

>XP_037389493.1 cellular tumor antigen p53 isoform X1 [Pygocentrus nattereri]

MQACRDPIGHRARVTCGLAPPAGEFPAELDGNAGISWSGESGVFVGEDQLGGLSGEARSVGLVSSSPLPMEPQATMEEPSESQEFAELWLQNLMGQTPEDSPWVNEEFPEDLQSMLVQPLFDEVSAELPQPSTSPPASTVPVATDYPGAHAFTLRFQQSGTAKSVTCTFSPELNKLFCQLAKTCPVQMEVSSPPPPGAVLRATAVYKKAEHVAEVVRRCPHHERTPENNDGLTPPAHLLRVEGNPRAQYHEDDNTLRHSVLVPYEAPQVGSEYTTVLYNYMCNSSCMGGMNRRPILTIITLETQDGQLLGRRSFEVRVCACPGRDRKTEESSFRKQQEAKNTNKTPATNKRSLKEPTSRPEASKKAKTSGSSDEEIYTLQVRGRERYEFLKKINDGLELSDVVPPADAEKYRQKLHSKSSRKERDGAPEPKRGKKLLVKEEKSDSD

>XP_032650427.1 cellular tumor antigen p53 [Chelonoidis abingdonii]

MIWFDLYNPGHKPALKSSLQAASSPSLHYAKSLALHEAAPLCAAPWGARQHPLPLCPPPGQPDDMEEQDGDAGLMGPAVTWPRHPRNASWLPGNPQLVHVDAKCPGETGVQISAERTAGMEPMLDPGLEPMLDPGLEPPLSQESFSDLWSMMARRTAQDSNTQDCPGLYSLPDPDLSLDLGLSDSADPSLLLPQAGGSDGGWGLPDPAPEPPPTSATVPSTEDYAGEHGFELAFQQSGTAKSVTCTYSPQLNKLYCQLAKTCPVQIRTASQPPAGSIIRATAVYKKSEHVAEVVRRCPHHERCEEYRDGVAPARHLIRIEGNQQAHYYDDENTKRQSVTVPYETPQLGSDCTTVLYNFMCNSSCMGGMNRRPILAIITLEGRHRQLLGRRCFEVRVCACPGRDRRTEEENFCKKLAGRVLNGAGAHKGGGAKRALQATMETAENPKKLVVSSEKEVFLLEVHGRKRYMMLKEINDALEMAAAKQLGEPESHRNATPSRLLKTRKESADGVLPRSGKKLLVKEEDSE

>XP_037347933.1 cellular tumor antigen p53 [Talpa occidentalis]

MEESQSELGVGTPLSQETFSDLWNLLHGNNVLPQCPEVEELLMTSDWLDDDPNEASRIPAASAPVAPVPATLAPAASWPLSSSVPCQKPYPGSYGFRLDFLQSGTAKSVTCTYSPDLNKLFCQLAKTCPVQLWVSSPPPSGTRVRAMAIYKKSEYMTEVVRRCPHHERTSDNDGLAPPQHLIRVEGNLHAMYLDDENTFRHSVVVPYELPEVGSDCTTIHYNFMCNSSCMGGMNRRPIVTIITLEDSNGNLLGRSSFEVRVCACPGRDRRTEEENFRKRRELCAQLPSGSTKRAMPTSTSSSPQQKKKRLDAEYFTLQIRGRERYEMFQMLNDALELKDAQAGKEPGGSRAHSSHLKSKKGQSTSRHKKPMFKTEGPDSD

>XP_030825916.1 cellular tumor antigen p53 isoform X1 [Camarhynchus parvulus]

MAEHLQLLEGEGFLDLWNMLPNNIGSMPEDLLEWELSPPEPPGVGEGRALPPPPAEPPPPLPSAVVPSTEDYGGEHDFRVEFRETGTAKSVTCTYSPALNKLFCRLAKPCPVQVRVGVPPPPGALLRALAVYKKAEHVAEVVRRCPHHERAGGDSEGNAPAQHLIRVEGNPQARYQDDETTKRHSVAVPYEPPEVGSECTTVLYNFMCNSSCMGGMNRRPILTILTLETPGGQILGRRCFEVRVCACPGRDRRHEEETQRKRGGAKGGAKRALSPPAEAPESSKKRAQEPDDDIFLLRVRGRKRYEMFCKVNYALERTYDGEAAPQKSKGRRPRGEGRVPRGKKLLLKAESGESD

>XP_041094191.1 cellular tumor antigen p53-like [Polyodon spathula]

MADPVSDSLEPLSQESFQELWSIIGTRGDEIWEQNVNLFPELESLQNEAICSSSNVEVLFESPAPVQQHPLPSVSETATTASTVPSTTDYPGELGFELRFQQSSTAKSVTCTYSPSLNKLFCQLAKTCPVQMHVSAPPPPGAVVRATAVYKKSEHVAEVVKRCPHHERTPENNEGHVPPGHLIRIEGNQLARYQEDQRTRRQSVLVPYEPPQLGSTCTTVLYNYMCNSSCIGGMNRRPILTIITLETSDGRLLGRRCFEVRVCACPGRDLKSDEENSRKQLEKAGSKASAGGTKRSFKEVSLPAPGSSKRNRASAGSDEDTFTLQVHGRERYEMLRRINESLELKDLVPQSEQEKYRERLHRNGSRKERGGLAPKRGKKLLIKEDKSNSD

>QBM00765.1 tumor antigen p53 [Megalobrama amblycephala]

MAENPESQEFADLWERNLISAPEGGSCWDISDEYLPNSFDPSFFNLLTEQPQPSTSPPTSTVPIATDYPGEHGFKLQFPQSGTAKSVTCTYSPDLNKLFCQLAKTCPVQMVVDIAPPQGSLLRATAIYKKSEHVAEVVRRCPHHERTPDTDGLAPAAHLIRVEGNLRATYKEDDSTSRHSVVVPYEAPQLGAGFTTVLYNYMCNSSCMGGMNRRPILTIITLETQDGQMLGRRSFEVRVCACPGRDRKTEESNFRKDQETKTPSKNPSTTKRSIVKESSSSASRPEGSKKAKMSVSSDEEIFSLQVRGRERFEMLKKINDSLELSDVVPPSDVDKYRQKLVSKNKKERDGQTPEPKRGKKLMVKDEKSDSD

>XP_006738950.1 cellular tumor antigen p53 [Leptonychotes weddellii]

MQDPQSELTIDPPLSQETFSELWNLLPENNVLSSELSPAVDELLLSEGVANWLDTGSDDAPRMSAAPAPAGLGPATSWPLSSFVPSPKTYPGTYGFRLGFLHSGTAKSVTCTYSPSLNKLFCQLAKTCPVQLWVSSPPPPDTCVRAMAIYKKSEFVTEVVRRCPHHERCPDSSDGLAPPQHLIRVEGNLRAKYLDDRNTFRHSVVVPYEPPEVGSDCTTVHYNYMCNSSCMGGMNRRPILTIITLEDSSGNVLGRSSFEVRVCACPGRDRRTEEENFRKKGEPCPEPPPGSTKRALTPSTSSSPPQKKKPLDGEYFTLQIRGRERFNMFRELNEALELKDALSGKEPGGSRAHSSHLKAKKGQSTSRHKKLMFKREGPDSD

>NP_001277773.2 cellular tumor antigen p53 [Bubalus bubalis]

MEESQAELNVEPPLSQETFSDLWNLLPENNLLSSELSAPVDDLLPYTDVATWLDECPNEVPQMPEPSAPAAPPPATPAPATSWPLSSFVPSQKTYPGNYGFRLGFLHSGTAKSVTCTYSPSLNKLFCQLAKTCPVQLWVDSPPPPGTRVRAMAIYKKLEHMTEVVRRCPHHERSSDYSDGLAPPQHLIRVEGNLRAEYLDDRNTFRHSVVVPYESPEIDSECTTIHYNFMCNSSCMGGMNRRPILTIITLEDSCGNLLGRNSFEVRVCACPGRDRRTEEENFRKKGQSCPEPPPGSTKRALPTNTSSSPQQKKKRLDEEYFTLQIRGLKRYEMFRELNDALELKDALDGREPGESRAHSSHLKSKKRPSPSCHKKPMLKREGPDSD

>MBV97997.1 Cellular tumor antigen p53 [Eschrichtius robustus]

MFGVPLLSSLAFLHSPSPISFGSYDKRPFANLSRQVWLSGQISGHTASQGAVMEESQAELGVEPPLSQETFSDLWKLLPENNLLSSELSPAVDDLLLSPEDVANWLDERPDEAPQMPEPTAPAAPAPAAPAPATSWPLSSFVPSQKTYPGSYGFRLGFLHSGTAKSVTCTYSPALNKLFCQLAKTCPVQLWVSSPPPPGTRVRAMAIYKKSEYMTEVVRRCPHHERCSDYSDGLAPPQHLIRVEGNLRAEYLDDRNTFRHSVVVPYEPPEVGSDCTTIHYNFMCNSSCMGGMNRRPILTIITLEDSSGNLLGRNSFEVRVCACPGRDRRTEEENFRKKGQSGPEPPPGSAKRALPTSTSSSPPQKKKPLDGEYFTLQIRGRERFEMFRELNEALELKDAQAGKEPGESRAHSSHLKSKKGQSPSRHKKLMFKREGPDSD

>XP_034845405.1 cellular tumor antigen p53 [Mirounga leonina]

MQDPQSELTIDPPLSQETFSELWNLLPENNVLSSELSPAVDDLLLSEGVANWLDTGSDDAPRMSAAPAPAGLGPATSWPLSSFVPSPKTYPGTYGFRLGFLHSGTAKSVTCTYSPSLNKLFCQLAKTCPVQLWVSSPPPPDTCVRAMAIYKKSEFVTEVVRRCPHHERCPDSSDGLAPPQHLIRVEGNLRAKYLDDRNTFRHSVVVPYEPPEVGSDCTTVHYNYMCNSSCMGGMNRRPILTIITLEDSSGNVLGRSSFEVRVCACPGRDRRTEEENFRKKGEPCPEPPPGSTKRALTPSTSSSPPQKKKPLDGEYFTLQIRGRERFNMFRELNEALELKDALSGKEPGGSRAHSSHLKAKKGQSTSRHKKLMFKREGPDSD

>XP_036692565.1 cellular tumor antigen p53 [Balaenoptera musculus]

MEESQAELGVEPPLSQETFSDLWKLLPENNLLSSELSPAVDDLLLSPEDVANWLDERPDEAPQMPEPTAPAAPAPAAPAPATSWPLSSFVPSQKTYPGSYGFRLGFLHSGTAKSVTCTYSPALNKLFCQLAKTCPVQLWVSSPPPPGTRVRAMAIYKKSEYMTEVVRRCPHHERCSDYSDGLAPPQHLIRVEGNLRAEYLDDRNTFRHSVVVPYEPPEVGSDCTTIHYNFMCNSSCMGGMNRRPILTIITLEDSGGNLLGRNSFEVRVCACPGRDRRTEEENFHKKGQSGPEPPPGSAKRALPTSTSSSPPQKKKPLDGEYFTLQIRGRERFEMFRELNEALELKDAQAGKEPGESRAHSSHLKSKKGQSPSRHKKLMFKREGPDSD

**>KYO28679.1 cellular tumor antigen p53 [Alligator mississippiensis]**

MRQMESIMDPDLDPPLSQPFLDFWNVLDNNVRSIPKEQAELWDPQDLVLGLPDLGDLPLLEELEGAPVAGLGREAPPPDALPTSSIVPSTEDYPGAHGFEVAFQPSGTAKSVTCTYSPVLNKLFCQLARSCPVQVRVTQAPPPGALIRAGAVYKKAEHVAEVVRRCPHHERSAEHSDGVAPAQHLIRVEGNPQAQYCHDETTKRHSVTVPYTPPEVGSDSTTVLYNFMCNSSCMGGMNRRPILAILTLETKSGQLLGRCCFEVRICACPGRDRRTEEENLRNKAATAGGGAKRALKVPADDLPNPKKRVPNSSTEIFTLQIRGRERYEMFKKLNEGLEALDGQEARAEDPGIRSPKPLLKARRAKGLALVSCKKLLVKDESQDSD

>XP_025050964.1 cellular tumor antigen p53 isoform X1 [Alligator sinensis]

MLGLGKPRRMLLLALALPLPLLLLLPVPVPPPPALASRLDNNVRSIPKEQAELWDPQDLVLGLPDLGDLPLLEELEGAPVAGLGREAPPPGALPTSSIVPSTEDYPGAHGFEVAFQPSGTAKSVTCTYSPVLNKLFCQLAQSCPVQVRVAQAPPPGAMIRAGAVYKKAEHVAEVVRRCPHHERSAEHSDGVAPAQHLIRVEGNPQAQYCHDETTKRHSVTVPYTPPEVGSDSTTVLYNFMCNSSCMGGMNRRPILAILTLETKSGQLLGRRCFEVRICACPGRDRKTEEENLRNKAATTGGGAKRALKVPTDDLPNPKKRVPNPSTEIFTLQIRGHERYEMFKKLNEGLEALDGQEARAEDPGIRSPKPLLKARRAKGLALVSCKKLLVKDESQDSD

>XP_032940146.1 cellular tumor antigen p53 [Catharus ustulatus]

MAEDLEPLLEGEGFLDWWKTLPDNIGSIPEETLEWEDLTTLGVSPEGPPPEPPRQVEPPAPPPAPVVPSTEDYGGEHDFRLEFRETGTAKSVTCTYSPVLNKLYCRLAKPCPVQVRVGVPPPPGALVRAVAVYKKSEHVAEVVRRCPHHERCGGGKDGNAPAQHLIWVEGNPKARYQDDETTKRHSVAVPYEPPQVGSECTTVLYNFMCNSSCMGGMNRRPILAILTLEGPGGQTLGRRCFEVRVCACPGRDRRLEEENQRKRGGAKGGAKRALPPPAEAPEGSKKKVLEPDNEIFCLQVRGRKRFEMLQMINEALEAKERGETAPKKTKGRRPRGEGPVPRGKKLLVKVESGDSD

>XP_036125049.1 cellular tumor antigen p53 isoform X1 [Molossus molossus]

MDEPQSEMDAPLSQETFLDLWKLLRENNDLTSDGSPPDELFQSPDLVNCLDNGPDEASSVAATPARATVTPAPATSWPLSSFVPSQKTYPGSYGFRLGFLNSGTAKSVTCTYSPTLNKLFCQLAKTCPVQLWVSSPPPFGTRVRAMAIYKKSEYMTEVVRRCPHHERCSDYSDGLAPPQHLIRVEGNLRAEYSDDKNTFRHSVVVPYEPPEVGSDCTTIHYNFMCNSSCMGGMNRRPILTIITLEDSSGNLLGRNSFEVRVCACPGRDRRTEEENSRKKEEPCPKQPHGSTKRALPTNTDSSPPPKMPPDGEYFTLQIRGRERYEMFRELNEALELKDAQAGKDPGGSKTRSSHLKSKKGQSTSRHKKPMFKREGPDSD

>XP_007166447.1 cellular tumor antigen p53 isoform X1 [Balaenoptera acutorostrata scammoni]

MEESQAELGVEPPLSQESFSDLWKLLPENNLLSSELSPAVDDLLLSPEDVANWLDERPDEAPQMPEPTAPAAPAPAAPAPATSWPLSSFVPSQKTYPGSYGFRLGFLHSGTAKSVTCTYSPALNKLFCQLAKTCPVQLWVSSPPPPGTRVRAMAIYKKSEYMTEVVRRCPHHERCSDYSDGLAPPQHLIRVEGNLRAEYLDDRHTFRHSVVVPYEPPEVGSDCTTIHYNFMCNSSCMGGMNRRPILTIITLEDSSGNLLGRNSFEVRVCACPGRDRRTEEENFRKKGQSGPEPPPGSAKRALPTSTSSSPPQKKKPLDGEYFTLQIRGRERFEMFRELNEALELKDAQAGRELGESRAHSSHLKSKKGQSPSRHKKLMFKREGPDSD

>XP_038027745.1 cellular tumor antigen p53 isoform X1 [Anas platyrhynchos]

MAEELEPLLEPPEIFLELWNMLPDNMHSLSPPDDPLAVQDLCPLEPSEPPPGPPPSTEPPPAAPPEPPRASPSSMVPSTEDYGGHYDFQLGFQETGTAKSVTCTYSPVLNKLYCRLAKPCPVQVRVGAAPPPGAVLRAVAVYKKSEHVAEVVRRCPHHERNGEGTDGLAPAQHLIRVEGNPQARYHDDETTKRHSVAVPYEPPEVGSDCTTVLYSFMCNSSCMGGMNRRPILAIITLEGPGGQLLGRRCFEVRVCACPGRDRKIEEENFRKRGGAGGGAKRALSPPTKAPETPKKRVLNPDNEIFCLQVRGRRRYEMLKEINDALQMAEEGAAPRPSKGHRPRGEGPLPRSGKKLLLKGEPPDSD

>XP_033278862.1 cellular tumor antigen p53 isoform X1 [Orcinus orca]

MCPLGHTASQGAVMEESQAELGVEPPLSQETFSDLWKLLPENNLLSSELSPAVDDLLLSPEDVANWLDERPDEAPQMPEPPAPAAPTPAAPAPATSWPLSSFVPSQKTYPGSYGFRLGFLHSGTAKSVTCTYSPPLNKLFCQLAKTCPVQLWVSSPPPPGTRVRAMAIYKKSEYMTEVVRRCPHHERCSDYSDGLAPPQHLIRVEGNLRAEYLDDRNTFRHSVVVPYEPPEVGSDCTTIHYNFMCNSSCMGGMNRRPILTIITLEDSNGNLLGRNSFEVRVCACPGRDRRTEEENFRKKGQSCPELPTGSAKRALPTGTSSSPPQKKKPLDGEYFTLQIRGRERFEMFRELNEALELKDAQAGKEPGESRAHSSHLKSKKGPSPSRHKKLMFKREGPDSD

>XP_012661495.1 cellular tumor antigen p53 [Otolemur garnettii]

MEETQSDLTIEPPLSQETFSDLWKLLPENNVLSSSLSPPVDDLMLSPDIVNWFDEGPDEALRTLEDPAPVASTTAALTPAASAPVTGWPLSSCVPSQTTYPGSYGFRLGFLNSGTAKSVTCTYSPVLNKMFCQLAKTCPVQLWFDSTPPPGSRIRAMAIYKQSQHMTEVVRRCPHHERCSESDGLAPPQHLIRVEGNLRVEYLDDKNTFRHSVVVPYEPPEVGSDCTTIHYNYMCNSSCMGGMNRRPILTIITLEDSSGNLLGRNSFEVRVCACPGRDRRTEEENSRKKGETCSEPSLGSTKRSLPTSTSSSPQPKKKLLEGEYFTLKIRGRERYEMFRELNEALEIKDAQAEKESDGNRAHSSQPKSKKEQSTSRHKKLMFKREGPDSD

>XP_037665508.1 cellular tumor antigen p53 [Choloepus didactylus]

MEEPQSDLSIEPPLSQETFSDLWKLLPENNVLSPSPSAVDDLFLPEDVASWLNNPEEPVGTPAAAALATTPVPAAPAPATPWPLSSSVPSQKEYPGVYNFQLGFLRSGTAKSVTCTYSPALNKLFCQLAKTCPVQLWVTSPPPPGARVRAMAIYKKSEHMTEVVRRCPHHERCSDYSDSLAPPQHLIRVEGNPRAEYWDDSNTFRHSVVVPYEMPEVGSDCTTIHYNFMCNSSCMGGMNRRPILTIITLEDSNGNLLGRGSFEVRICACPGRDRRTEEENFHKKGEPCHEPPAGTTKRALATTTGSSPPAKKKPLDGEYFTLQIRGHERFKMFRELNEALELKDAQAGKEPEGSRAHSSHLKSKKGQSTSRHKKLMFKREGPDSD

>XP_032947085.1 cellular tumor antigen p53 [Rhinolophus ferrumequinum]

MEVPQSELSVDPPLSQETFSDLWKLLPENNVLTPDVSLADLVNWLDGGPNEDPNVPATPGPAAATPAPATSPAPANSWPLSSFVPSQKTYPGNYGFQLGFLNSGTAKSVTCTYSPTLNKLFCQLAKTCPVQLWVSSPPPVGTRVRAMAIYKKSEYMTEVVRRCPHHERCSDYSDGLAPPQHLIRVEGNLHAEYLDDKHTFRHSVVVPYEPPEVGSDCTTIHYNFMCNSSCMGGMNRRPILTIITLEDSSGNLLGRNSFEVRVCACPGRDRRTEEENFRKKGEPCPKQPPGSSKRALPTNTSSSTPPKKPLDGEYFTLQIRGRERFEMFRELNEALELKDAQAGKESEGSRAHSSHLKSKKGQSTSRHKKLLFKREGPDSD

>XP_033704460.1 cellular tumor antigen p53 isoform X1 [Tursiops truncatus]

MCPLGHTASQGAVMEELQAELGVEPPLSQETFSDLWKLLPENNLLSSELSPAVDDLLLSPEDVANWLDERPDEAPQMPEPPAPAAPTPAAPAPATSWPLSSFVPSQKTYPGSYGFRLGFLHSGTAKSVTCTYSPPLNKLFCQLAKTCPVQLLVSSPPPPGTRVRAMAIYKKSEYMTEVVRRCPHHERCSDYSDGLAPPQHLIRVEGNLRAEYLDDRHTFRHSVVVPYEPPEVGSDCTTIHYNFMCNSSCMGGMNRRPILTIITLEDSNGNLLGRNSFEVRVCACPGRDRRTEEENFRKKGQSCPELPTGSAKRALPTSTSSSPPQKKKPLDGEYFTLQIRGRERFEMFRELNEALELKDAQAGKEPGESRAHSSHLKSKKGPSPSRHKKLMFKREGPDSD

>XP_032472646.1 cellular tumor antigen p53 [Phocoena sinus]

MEESQAELGVEPPLSQETFSDLWKLLPENNLLSSELSPAVDDLLLSPEDVANWLDERPDEAPQMPEPPAPAAPTPAAPAPATSWPLSSFVPSQKTYPGSYGFHLGFLHSGTAKSVTCTYSPALNKLFCQLAKTCPVQLWVSSPPPPGTRVRAMAIYKKSEYMTEVVRRCPHHERCSDYSDGLAPPQHLIRVEGNLRAEYLDDRNTFRHSVVVPYEPPEVGSDCTTIHYNFMCNSSCMGGMNRRPILTIITLEDSNGNLLGRNSFEVRVCACPGRDRRTEEENFHKKGQSCPELPTGSAKRALPTGTSSSPPQKKKPLDGEYFTLQIRGRERFEMLRELNEALELKDAQAGKEPGESRAHSSHLKSKKGQSPSRHKKLMFKREGPDSD

>XP_026986728.1 cellular tumor antigen p53 isoform X1 [Lagenorhynchus obliquidens]

MCPLGHTASQGAVMEELQAELGVEPPLSQETFSDLWKLLPENNLLSSELSPAVDDLLLSPEDVANWLDERPDEAPQMPEPPAPAAPTPAAPAPATSWPLSSFVPSQKTYPGSYGFRLGFLHSGTAKSVTCTYSPPLNKLFCQLAKTCPVQLLVSSPPPPGTRVRAMAIYKKSEYMTEVVRRCPHHERCSDYSDGLAPPQHLIRVEGNLRAEYLDDRHTFRHSVVVPYEPPEVGSDCTTIHYNFMCNSSCMGGMNRRPILTIITLEDSNGNLLGRNSFEVRVCACPGRDRRTEEENFRKKGQSCPELPTGSAKRVLPTSTSSSPPQKKKPLDGEYFTLQIRGRERFEMFRELNEALELKDAQAGKEPGESRAHSSHLKSKKGQSPSRHKKLMFKREGPDSD

>XP_028355209.1 cellular tumor antigen p53 [Physeter catodon]

MEESQAELGVEPPLSQETFSDLWKLLPENNLLSSELSPAVDDLLLSPEDVANWLDERPDEAPQMPESPAPAAPAPAAPAPATSWPLSSFVPSQKTYPGSYEFRLGFLHSGTAKSVTCTYSPALNKLFCQLAKTCPVQLWVSSPPPPGTRVRAMAIYKKSEYMTEVVRRCPHHERCSDYSDGLAPPQHLIRVEGNLRAEYLDDRNTFRHSVVVPYEPPEVGSDCTTIHYNFMCNSSCMGGMNRRPILTIITLEDSNGNLLGRNSFEVRVCACPGRDRRTEEENFRKKGQSCPEPPPGSAKRALPTSTSSSPPQKKKPLDGEYFTLQIRGRERFEMFRELNEALELKDAQAGKEPGESRAHSSHLKSKKGQSPSRHKKLMFKREGPDSD

>XP_025861192.1 cellular tumor antigen p53 [Vulpes vulpes]

MQEPQSELNIDPPLSQETFSELWNLLPENNVLSSELCPAVDELLLPESVVNWLDEDSDDAPRMPATSAPTAPGPAPSWPLSSFVPSPKTYPGTYGFRLGFLHSGTAKSVTWTYSPLLNKLFCQLAKTCPVQLWVSSPPPPNTCVRAMAIYKKSEFVTEVVRRCPHHERCSDSSDGLAPPQHLIRVEGNLRAKYLDDRNTFRHSVVVPYEPPEVGSDYTTIHYNYMCNSSCMGGMNRRPILTIITLEDSSGNVLGRNSFEVRVCACPGRDRRTEEENFHKKGEPCPEPPPGSTKRALPPSTSSSPPQKKKPLDGEYFTLQIRGRERYEMFRNLNEALELKDAQSGKEPGGSRAHSSHLKAKKGQSTSRHKKLMFKREGPDSD

>XP_021550167.1 cellular tumor antigen p53 [Neomonachus schauinslandi]

MQDPQSELTIDPPLSQETFSELWNLLPENNVLSSELSPAVDELLLSEGVANWLDTGSDDAPRMSAAPAPAGVGPATSWPLSSFVPSPKTYPGTYGFRLGFLHSGTAKSVTCTYSPSLNKLFCQLAKTCPVQLWVSSPPPPDTCVRAMAIYKKSEFVTEVVRRCPHHERCPDSSDGLAPPQHLIRVEGNLRAKYLDDRNTFRHSVVVPYEPPEVGSDCTTVHYNYMCNSSCMGGMNRRPILTIITLEDSSGNVLGRSSFEVRVCACPGRDRRTEEENFRKKGEPCPEPPPGSTKRALTPSTSSSPPQKKKPLDGDYFTLQIRGRERFNMFRELNEALELKDALSGKEPGGSRAHSSHLKAKKGQSTSRHKKLMFKREGPDSD

>NP_001189334.1 cellular tumor antigen p53 [Equus caballus]

MEETQTELGIEPPLSQETFSDLWKLLPENNVLSPDLSPAVNNLLLSPDVVNWLDEGPDEAPRMPAAPAPLAPAPATSWPLSSFVPSQKTYPGCYGFRLGFLNSGTAKSVTCTYSPTLNKLFCQLAKTCPVQLLVSSPPPPGTRVRAMAIYKKSEFMTEVVRRCPHHERCSDSSDGLAPPQHLIRVEGNLRAEYLEDRNTFRHSVVVPYEPPEVGSDCTTIHYNFMCNSSCMGGMNRRPILTIITLEDSSGNLLGRNSFEVRVCACPGRDRRTEEENFRKKEEPCPEPPPRSTKRVLSSNTSSSPPQKKKPLDGEYFTLQIRGRERFEMFRELNEALELKDAQTGKEPGGSKAHSSHLKSKKGQSTSSHKKLIFKREGPDSD

>XP_024604188.1 cellular tumor antigen p53 [Neophocaena asiaeorientalis asiaeorientalis]

MEESQAELGVEPPLSQETFSDLWKLLPENNLLSSELSPAVDDLLLSPEDVANWLDERPDEAPQMPEPPAPAAPTPATPAPATSWPLSSFVPSQKTYPGSYGFHLGFLHSGTAKSVTCTYSPALNKLFCQLAKTCPVQLWVSSPPPPGTRVRAMAIYKKSEYMTEVVRRCPHHERCSDYSDGLAPPQHLIRVEGNLRAEYLDDRNTFRHSVVVPYEPPEVGSDCTTIHYNFMCNSSCMGGMNRRPILTIITLEDSNGNLLGRNSFEVRVCACPGRDRRTEEENFHKKGQSCPELPTGSAKRALPTGTSSSPPQKKKPLDGEYFTLQIRGRERFEMLRELNEALELKDAQAGKEPGESRAHSSHLKSKKGQSPSRHKKLMFKREGPDSD

>XP_006218839.1 cellular tumor antigen p53 [Vicugna pacos]

MEESQSELGVEPPLSQETFSDLWKLLPENNLLSSEFSPAVDELLLSPEVTNWLDENPDEAPRMQEPPVPTASAPATSWPLSSFVPSQKTYPGSYGFRLGFLHSGTAKSVTCTYSPPLNKLFCQLAKTCPVQLWVSSLPPPGTRVRAMAIYKKSEYMTEVVRRCPHHERSSDYSDGLAPPQHLIRVEGNLRAKYLDDRNTFRHSVVVPYEPPEVGSDCTTIHYNFMCNSSCMGGMNRRPILTIITLEDASGNLLGRNSFEVRVCACPGRDRRTEEENFRKKGQPCPELPPGSTKRALPTSSSSSPPQKKKPLDGEYFTLQIRGRERFEMFRELNEALELKDAQAGKEPGENKAHSSHLKSKKGQSPSRHKKLMFKREGPDSD

>NP_001376147.1 cellular tumor antigen p53 [Canis lupus familiaris]

MQEPQSELNIDPPLSQETFSELWNLLPENNVLSSELCPAVDELLLPESVVNWLDEDSDDAPRMPATSAPTAPGPAPSWPLSSSVPSPKTYPGTYGFRLGFLHSGTAKSVTWTYSPLLNKLFCQLAKTCPVQLWVSSPPPPNTCVRAMAIYKKSEFVTEVVRRCPHHERCSDSSDGLAPPQHLIRVEGNLRAKYLDDRNTFRHSVVVPYEPPEVGSDYTTIHYNYMCNSSCMGGMNRRPILTIITLEDSSGNVLGRNSFEVRVCACPGRDRRTEEENFHKKGEPCPEPPPGSTKRALPPSTSSSPPQKKKPLDGEYFTLQIRGRERYEMFRNLNEALELKDAQSGKEPGGSRAHSSHLKAKKGQSTSRHKKLMFKREGPDSD

**>NP_990595.1 cellular tumor antigen p53 [Gallus gallus]**

MAEEMEPLLEPTEVFMDLWSMLPYSMQQLPLPEDHSNWQELSPLEPSDPPPPPPPPPLPLAAAAPPPLNPPTPPRAAPSPVVPSTEDYGGDFDFRVGFVEAGTAKSVTCTYSPVLNKVYCRLAKPCPVQVRVGVAPPPGSSLRAVAVYKKSEHVAEVVRRCPHHERCGGGTDGLAPAQHLIRVEGNPQARYHDDETTKRHSVVVPYEPPEVGSDCTTVLYNFMCNSSCMGGMNRRPILTILTLEGPGGQLLGRRCFEVRVCACPGRDRKIEEENFRKRGGAGGVAKRAMSPPTEAPEPPKKRVLNPDNEIFYLQVRGRRRYEMLKEINEALQLAEGGSAPRPSKGRRVKVEGPQPSCGKKLLQKGSD

>XP_030330235.1 cellular tumor antigen p53 isoform X1 [Strigops habroptila]

MGSYGVLWGAMGLRCPQFSVVLHKLFCRLAKPCPVQVWVGVPPPPGALLRAVAVYKKSEHVAEVVRRCPHHERCGGGSDGLAPPQHLIRVEGNPQARYHDDETTKRHSVTVPYEPPEVGSDCTTVLYNFMCNSSCMGGMNRRPILAILTLEGPAGQVLGRRCFEVRVCACPGRDRRIEEENHRRRGGAGGVAKRGEGRGHTVYALPPPVEAPESSKKRVLNPDNEEFSLLVRGRHRYEMLKKINDALEAYEGGAAPERPVKGRRPRGEGLLPRSGKKLLLKGAGPDSD

>XP_016010258.1 cellular tumor antigen p53 isoform X1 [Rousettus aegyptiacus]

MDMPQSELNMEAPLSQETFSDLWKLLPQNNVLPPDISLPVDEFLLPPDLVNWLDEDRNESPRGPTTSTTIAATPAPAISWPLSSFVPSQKTYPGCYDFRLGFLNSGTAKSVTCTYSPTLNKLFCQLAKTCPVQLWVSSPPPLGARVRAMAIYKKSEHMTEVVRRCPHHERCSDYSDGLAPPQHLIRVEGNLRAEYLDDKNTFRHSVVVPYEPPEVGSDCTTIHYNFMCNSSCMGGMNRRPILTIITLEDSSGNLLGRNIFEVRVCACPGRDRRTEEENFQNKGKPCPKQPPGSTKRALPTNTTSSSPSPKMPLDEEYFTLQIRGRKNFEMLRELNEALELKDAQAGKEPRGSRAHSSHLKSKKGQSTSCHKKLMLKTEGPDSD

>NIG58109.1 cellular tumor antigen p53 isoform 1 [Pontoporia blainvillei]

MEESQAELGVEPPLSQETFSDLWKLLPENNLLSSELSPAVDDLLLSPEDVANWLDERPDEAPQMPETPAPAAPTPAAPAPATSWPLSSFVPSQKTYPGSYGFRLGFLHSGTAKSVTCTYSPALNKLFCQLAKTCPVQLWVSSPPPPGTRVRAMAIYKKSEYMTEVVRRCPHHERCSDYSDGLAPPQHLIRVEGNLRAEYLDDRNTFRHSVVVPYEPPEVGSDCTTIHYNFMCNSSCMGGMNRRPILTIITLEDPNGNLLGRNSFEVRVCACPGRDRRTEEENFRKKGQSCPELPAGSAKRALPTGTSSSPPQKKKPLDGEYFTLQIRGRERFEMFRELNEALELKDAQVGKEPGESRAHSRPFKAQVQEEGPRASQSDDVISPPSLSSQPPEV

>XP_039703172.1 cellular tumor antigen p53 isoform X1 [Pteropus giganteus]

MDIPQSELNMEPPLSQETFSDLWKLLPQNNVLPPDISLPEDEFLLPSVLVNWLDEDQNESPRVPAAATPAPATSWPLSSFVPSQKTYPGSYDFRLGFLNSGTAKSVTCTYSPTLNKLFCQLAKTCPVQLWVSSPPPLGTRVRAMAIYKKSEYMTEVVRRCPHHERCSDYSDGLAPPQHLIRVEGNLRAEYLDDKHTFRHSVVVPYEPPEVGSDCTTIHYNFMCNSSCMGGMNRRPILTIITLEDSSGNLLGRNSFEVRVCACPGRDRRTEEENFRKKGEPCPKKPPGSTKRALPTDTTSSSPSPKMPLDEEYFTLQIRGRKNFEILRELNEALELKDAQAGKEPRGSRAHSSHLKSKKGQSTSCHKKLMFKREGPDSD

>XP_030717425.1 cellular tumor antigen p53 isoform X1 [Globicephala melas]

MCPLGHTASQGAVMEELQAELGVEPPLSQETFSDLWKLLPENNLLSSELSPAVDDLLLSPEDVANWLDERPDEAPQMPEPPAPAAPTPAAPAPATSWPLSSFVPSQKTYPGSYGFRLGFLHSGTAKSVTCTYSPPLNKLFCQLAKTCPVQLLVSSPPPPGTRVRAMAIYKKSEYMTEVVRRCPHHERCSDYSDGLAPPQHLIRVEGNLRAEYLDDRNTFRHSVVVPYEPPEVGSDCTTIHYNFMCNSSCMGGMNRRPILTIITLEDSNGNLLGRNSFEVRVCACPGRDRRTEEENFRKKGQSCPELPTGSAKRALPTSTSSSPPQKKKPLDGEYFTLQIRGRERFEMFRELNEALELKDAQAGKEPGESRAHSRPFKAQVQEEGPRASQSDDVISPPPLSSQPPEV

>XP_030151376.1 cellular tumor antigen p53 isoform X1 [Lynx canadensis]

MQEPPLELTIEPPLSQETFSELWNLLPENNVLSSELSSAMNELPLSEDVANWLDEAPDDASGMSAVPAPAAPAPATPAPAISWPLSSFVPSQKTYPGAYGFHLGFLQSGTAKSVTCTYSPPLNKLFCQLAKTCPVQLWVRSPPPPGTCVRAMAIYKKSEFMTEVVRRCPHHERCPDSSDGLAPPQHLIRVEGNLHAKYLDDRNTFRHSVVVPYEPPEVGSDCTTIHYNFMCNSSCMGGMNRRPIITIITLEDSNGKLLGRNSFEVRVCACPGRDRRTEEENFRKKGEPCPEPPPGSTKRALPPSTSSTPPQKKKPLDGEYFTLQIRGRERFEMFRELNEALELKDAQSGKEPGGSRAHSSSHLKAKKGQSTSRHKKPMLKREGLDSD

>NP_001009294.1 cellular tumor antigen p53 [Felis catus]

MQEPPLELTIEPPLSQETFSELWNLLPENNVLSSELSSAMNELPLSEDVANWLDEAPDDASGMSAVPAPAAPAPATPAPAISWPLSSFVPSQKTYPGAYGFHLGFLQSGTAKSVTCTYSPPLNKLFCQLAKTCPVQLWVRSPPPPGTCVRAMAIYKKSEFMTEVVRRCPHHERCPDSSDGLAPPQHLIRVEGNLHAKYLDDRNTFRHSVVVPYEPPEVGSDCTTIHYNFMCNSSCMGGMNRRPIITIITLEDSNGKLLGRNSFEVRVCACPGRDRRTEEENFRKKGEPCPEPPPGSTKRALPPSTSSTPPQKKKPLDGEYFTLQIRGRERFEMFRELNEALELKDAQSGKEPGGSRAHSSHLKAKKGQSTSRHKKPMLKREGLDSD

>XP_011357166.1 cellular tumor antigen p53 isoform X1 [Pteropus vampyrus]

MDIPQSELNMEPPLSQETFSDLWKLLPQNNVLPPDISLPEDEFLLPSVLVNWLDEDQNESPRVPAAATPAPATSWPLSSFVPSQKTYPGSYDFRLGFLNSGTAKSVTCTYSPTLNKLFCQLAKTCPVQLWVSSPPPLGTRVRAMAIYKKSEYMTEVVRRCPHHERCSDYSDGLAPPQHLIRVEGNLRAEYLDDKHTFRHSVVVPYEPPEVGSDCTTIHYNFMCNSSCMGGMNRRPILTIITLEDSSGNLLGRNSFEVRVCACPGRDRRTEEENFRKKGEPCPKKPPGSTKRALPTDTTSSSPSPKMPLDEEYFTLQIRGRKNFEILRELKEALELKDAQAGKEPRGSRAHSSHLKSKKGQSTSCHKKLMFKREGPDSD

>XP_039087428.1 cellular tumor antigen p53 isoform X1 [Hyaena hyaena]

MQEPPLELNIEPPLSQETFSELWNLLPENNVLSSELSLEVNELPLSEDVANWLDEAPDDASRMSAAPAPTAPAPATPAPAISWPLSSFVPSQKTYPGAYGFRLGFLQSGTAKSVTCTYSPSLNKLFCQLAKTCPVQLWVSSPPPPGTCVRAMAIYKKSEFMTEVVRRCPHHERCPDSSDGLAPPQHLIRVEGNMHAKYLDDRNTFRHSVVVPYEPPEVGSDCTTIHYNFMCNSSCMGGMNRRPIITIITLEDSNGKLLGRNSFEVRVCACPGRDRRTEEENFRKKGEPCPEPPPGSTKRALPPSTSSTPPQKKKPLDGEYFTLQIRGRDRFEMFREMNEALELKDALNGREPGGSRAHSSHLKAKKGQSTSRHKKPMFKREGLDSD

>XP_006922397.1 cellular tumor antigen p53 [Pteropus alecto]

MDIPQSELNMEPPLSQETFSDLWKLLPQNNVLPPDISLPENEFLLPSVLVNWLDEDQNESPRVPAAATPAPATSWPLSSFVPSQKTYPGSYDFRLGFLNSGTAKSVTCTYSPTLNKLFCQLAKTCPVQLWVSSPPPLGTRVRAMAIYKKSEYMTEVVRRCPHHERCSDYSDGLAPPQHLIRVEGNLRAEYLDDKHTFRHSVVVPYEPPEVGSDCTTIHYNFMCNSSCMGGMNRRPILTIITLEDSSGNLLGRNSFEVRVCACPGRDRRTEEENFHKKGEPCPKKPPGSTKRALPTDTTSSSPSPKMPLDEEYFTLQIRGRKNFEILRELNEALELKDAQAGKEPRGSRAHSSHLKSKKGQSTSCHKKLTLKREGPDSN

>XP_010613193.1 cellular tumor antigen p53 [Fukomys damarensis]

MEEPQSDLSIEPPLSQETFSDLWKLLPENNVLSSSLSSPMDDLLLSPEDVVNWLGGNPDEDIQVSAPVPEPPTPVAPTTAAPTPASSWPLSSSVPSHKTYHGNYGFRLGFLQSGTAKSVTCTYSPVLNKLFCQLAKTCPVQVWVESPPPPGTRVRAMAIYKKSQHMTEVVRRCPHHERCSDTDGLAPPQHLIRVEGNLRAEYLDDRTTFRHSVVVPYDLPEVGSDCTTIHYNYMCNSSCMGGMNRRPILTIITLEDSSGNLLGRNSFEVRVCACPGRDRRTEEENFHKKGGSCPEPTPGSIKRALPTNTSSSPQTKKKPLDGEYFTLKIRGRERFEMFRELNEALELKDAQTEKEPGESRPHSSYLKSKKGQSTSSHKKLMFKREGPDSD

>NP_001297199.1 cellular tumor antigen p53 [Heterocephalus glaber]

MEEPQSDLSIEPPLSQETFSDLWKLLPENNVLSSSLSSPMDDLLLSPEDVVNWLGGNPDEDVQVSAAPVPEPPTPVAPAPAAPAPATSWPLSSSVPSHKTYQGNYGFHLGFLQSGTAKSVTCTYSPVLNKLFCQLAKTCPVQVWVESPPPPGTRVRAMAIYKKSQHMTEVVRRCPHHERCSDSDGLAPPQHLIRVEGNLRAEYLDDRTTFRHSVVVPYDLPEVGSDCTTIHYNYMCNSSCMGGMNRRPILTIITLEDSSGNLLGRNSFEVRVCACPGRDRRTEEENFHKKGGSCPEPTPGSIKRALPTGTNSSPQPKKKPLDGEYFTLKIRGRERFEMFRELNEALELKDAQTEKEPGESRPHSSYLKSKKGQSTSCHKKLMFKKEGPDSD

**>NP_001118164.1 cellular tumor antigen p53 [Oncorhynchus mykiss]**

MADLAENVSLPLSQESFEDLWKMNLNLVAVQPPETESWVGYDNFMMEAPLQVEFDPSLFEVSATEPAPQPSISTLDTGSPPTSTVPTTSDYPGALGFQLRFLQSSTAKSVTCTYSPDLNKLFCQLAKTCPVQIVVDHPPPPGAVVRALAIYKKLSDVADVVRRCPHHQSTSENNEGPAPRGHLVRVEGNQRSEYMEDGNTLRHSVLVPYEPPQVGSECTTVLYNFMCNSSCMGGMNRRPILTIITLETQEGQLLGRRSFEVRVCACPGRDRKTEEINLKKQQETTLETKTKPAQGIKRAMKEASLPAPQPGASKKTKSSPAVSDDEIYTLQIRGKEKYEMLKKFNDSLELSELVPVADADKYRQKCLTKRVAKRDFGVGPKKRKKLLVKEEKSDSD

>XP_031663410.1 cellular tumor antigen p53 isoform X1 [Oncorhynchus kisutch]

MTVPGWLFFPACCFEVLENNIMADLAENVSLPLSQESFEDLWKMNLNLMAVQPPVTESWVGYDNFMMEAPLQVEFDPSLFEVSATEPAPQPSISTLDTGSPPTSTVPTTSDYPGALGFQLRFLQSSTAKSVTCTYSPDLNKLFCQLAKTCPVQIVVDHPPPPGAVVRALAIYKKLSDVADVVRRCPHHQSTSENNEGPAPRGHLVRVEGNQRSEYMEDGNTLRQSVLVPYEPPQVGSECTTVLYNFMCNSSCMGGMNRRPILTIITLETQDGQLLGRRSFEVRVCACPGRDRKTEEINLKKQQETILETKTKPAQGIKRAMKETSLPAPQPEASKKTKSSPAVSDDEIYTLQIRGKEKYEMLKKFNDSLELSELVPVADADKYRQKRLTKRVAKRDFGVGPKKGKKLLVKEEKSDSD

>XP_029525966.1 cellular tumor antigen p53 isoform X1 [Oncorhynchus nerka]

MTKKMACSPVTRHVSDFNLRENKVSNPLTETSFSTTFPGVTDDSPAWLFFPACCFEVLENNIMADLAENVSLPLSQESFEDLWKMNLNLMAVQPPVTESWVGYDNFMMEAPLQVEFDPSLFEVSATEPAPQPSISTLDTGSPPTSTVPTTSDYPGALGFQLRFLQSSTAKSVTCTYSPDLNKLFCQLAKTCPVQIVVDHPPPPGAVVRALAIYKKLSDVADVVRRCPHHQSTSENNEGPAPRGHLVRVEGNQRSEYMEDRNTLRQSVLVPYEPPQVGSECTTVLYNFMCNSSCMGGMNRRPILTIITLETQEGQLLGRRSFEVRVCACPGRDRKTEEINLKKQQETTLETKTKPAQGIKRAMKEASLPAPRPEASKKTKSSPAVSDDEIYTLQIRGKEKYEMLKKFNDSLELSELVPVADAEKYRQKRLTKRVAKRDFGVGPKKGKKLLVKEEKSDSD

>XP_024250651.1 cellular tumor antigen p53 [Oncorhynchus tshawytscha]

MADLAENVSLPLSQESFEDLWKMNLNLMEVQPPVTESWVGYDNFMMEAPLQVEFDPSLFEVSATEPAPQPSISTLDTGSPPTSTVPTTSDYPGALGFQLRFLQSSTAKSVTCTYSPDLNKLFCQLAKTCPVQIVVDHPPPPGAVVRALAIYKKLSDVADVVRRCPHHQSTSENNEGPAPRGHLVRVEGNQRSEYMEDGNTLRQSVLVPYEPPQVGSECTTVLYNFMCNSSCMGGMNRRPILTIITLETQEGQLLGRRSFEVRVCACPGRDRKTEEINLKKQQETILETKTKPAQGIKRAMKEASLPAARPEASKKTKSSPAVSDDEIYTLQIRGKEKYEMLKKFNDSLELSELVPVADADKYRQKRLTKRVAKRDFGVGLKKGKKLLVKEEKSDSD

>XP_035637161.1 cellular tumor antigen p53 isoform X1 [Oncorhynchus keta]

MGVIQQGRFPGWLFFPACCFGVLENNIMADMAENVSLPLSQESFEDLWKMNLNLMAVQPPVTESWVGYDNLMMEAPLQVEFDPSLFEVSATEPAPQPSISTLDTGSPPTSTVPTTSDYPGALGFQLRFLQSSTAKSVTCTYSPDLNKLFCQLAKTCPVQIVVDHPPPPGAVVRALAIYKKLSDVADVVRRCPHHQSTSENNEGPVPRGHLVRVEGNQRSEYMEDRNTLRQSVLVPYEPPQVGSECTTVLYNFMCNSSCMGGMNRRPILTIITLETQEGQLLGRRSFEVRVCACPGRDRKTEEINLKKQQETTLETKTKPAQGIKRAMKEASLPAPRPEASKKTKSSPAVSDDEIYTLQIRGKEKYEMLKKFNDSLELSELVPVADAEKYRQKHLTKRVAKRDFGVGPKKGKKLLVKEEKSDSD

>XP_029557503.1 cellular tumor antigen p53 [Salmo trutta]

MADLVENVSLPLSQESFEDLWKMNLNLMEVQPPVTDAWEGYDNFMMETPLQEEFDPSLFEVSATEPAPQPSISTLDTGSPPTSTVPTTSDYPGALGFQLRFLQSSTAKSVTCTYSPDLNKLFCQLAKTCPVQIVVDHPPPPGAVVRALAIYKKLSDVADVVRRCPHHQSTSENNEGPAPRGHLVRVEGNQRAEYMEDGNTLRQSVLVPYEPPQVGSECTTVLYNFMCNSSCMGGMNRRPILTIITLETQEGQLLGRRSFEVRVCACPGRDRKTEEINLKKQQETTLETKTKPAQGIKRSMKAASLQAPRPEASKKTKSSPAVSDDEIYTLQIRGKEKYEMLKKFNDSLELSELVPVADADKYRQKRLTKRVAKREIGVGPKKGKKLLVKEEKSDSD

>XP_023823892.1 cellular tumor antigen p53 [Salvelinus alpinus]

MADLAENVSLPLSQESFEDLWKMNLNLLAVPSPVTSAWGEYGNFMMETPLQGEFDPSLFEVSATEPAPQPSISTLDTGSPPTSTVPTTSDYPGALGFQLRFLQSSTAKSVTCTYSPDLNKLFCQLAKTCPVQIVVDHPPPPGAVVRALAIYKKLSDVADVVRRCPHHQSTSENNEGHAPRGHLVRVEGNQRAEYMEDGNTLRQSVLVPYEPPQVGSECTTVLYNFMCNSSCMGGMNRRPILNIITLETQEGQLLGRRSFEVRVCACPGRDRKTEEINLKKQQETTLETKTKPAQGIKRSMKEASLPAPRPEASKKTKSSPAVSDDEIYTLQIRGKEKYEMLKKFNDSLELSELVPVADADKYRQKRLTKRVAKREMGVGPKKGKKLLVKEEKSDSD

>XP_038861878.1 cellular tumor antigen p53 isoform X1 [Salvelinus namaycush]

MADLAENVSLPLSQESFEDLWKMNLNLLAVPSPVTSAWVDYGNFMMETPLQGEFDPSLFEVSATEPAPQPSISTLDTGSPPTSTVPTTSDYPGALGFQLRFLQSSTAKSVTCTYSPDLNKLFCQLAKTCPVQIVVDHPPPPGAVVRALAIYKKLSDVADVVRRCPHHQSTSENNEGPAPRGHLVRVEGNQRAEYMEDGNTLRQSVLVPYEPPQVGSECTTVLYNFMCNSSCMGGMNRRPILTIITLETQEGQLLGRRSFEVRVCACPGRDRKTEEINLKKQQETTLETKPAQGIKRSMKEASLPAPRPEASKKTKSSPAVSDDEIYTLQIRGKEKYEMLKKFNDSLELSELVPVADADRYRQKRLTKRVAKREMGVGPKKGKKLLVKEEKSDSD

>ACH73252.1 tumor suppressing protein p53 [Coregonus lavaretus]

MADLVENVSLPLSQESFEDLWKMNLNLMEVQPPVTEAWVEYDNFMMEAPLQGEFDQSLFEVSAPQPSISTLDTGSPPTSTVPTTSDYPGALGFQLRFLQSSTAKSVTCTYSPDLNKLFCQLAKTCPVQIVVDHPPPPGAVVRALAVYKKLSDVADVVRRCPHHQSTSENNEGPAPRGHLVRVEGNQRAEYMEDGNTLRQSVLVPYEPPQVGSECTTVLYNFMCNSSCMGGMNRRPILTIITLETQEGQLLGRRSFEVRVCACPGRDRKTEEINLKKQQETILETKTKPAQGTKRSVKEASLPAPRPEVSKKTKSSSPAVSDDEIYTLQIRGKEKYEMLKKLNDCLELSELVPAADADKYRQKRLTKRVAKRELGVGPKKGKKLLVKEERSDSD

>XP_010866226.1 cellular tumor antigen p53 isoform X1 [Esox lucius]

MAELSENVSLPLSQESFEDLWNMNLNLMGGQPPTISEPWSGYNDFIMDTPLQGEFDASQYCVPAPQPSISTRDTCSSPTSSVPTTSDYPGTHGFQLRFLQSSTAKSVTCTYSSCLNKLFCQLAKTCPIQMVVDHPPPPGAVVRALAIYKKLSDVADVVRRCPHHQSTSENNEGPAPRGHLIRVEGNQRAEYIEDRSTQRQSVVVPYEPPQVGSECTTVLYNFMCNSSCMGGMNRRPILTIITLETLDGQLLGRCSFEVRVCACPGRDRKTEEINLKKQQEVALETKSKPAQGTKRPVKEASLLVPRPEVSKKTKVSSPVVSDDEIYTLQIRGKERYEMLKKINDGLELSDLVPTADADKYRQKQDPLTKRVAKQDFRVGPKKGKKLLVKEERSDSD

>XP_026854214.2 cellular tumor antigen p53 isoform X1 [Electrophorus electricus]

MNGRVHHLGEDHISVRKSFLELLYPLSCTNELETEKEGLMEEPTESQEFADLWSQNLMVPKPDDSSWGNDNEFTLEDLHDMLEQPLFSEVQMEPPQPSTSPPASTVPVTTDYPGAYDFTLRFQQSSTAKSVTCTYSPDLNKLFCQLAKTCPVQMEVNSLPPLGAVLRATAVYKKSEHVAEVVRRCPHHEKTQETNNGLAPPGHLLRVEGNSRAIYQEDDNTLRHSVLVPYEPPQVGSECTTVLYNYMCNSSCMGGMNRRPILTIITLETQDGQLLGRRSFEVRVCACPGRDRKIEESNFKKQQEARPASKTPGPAKRSESLHVCVSLSEEACVRLSLSTSGNPVHIGCADMPPHPEASKKAKTSSTSNEELYTLQVRGKERYEMLKKINDGLELNDMVPPADVEKYRQKLLTRSRKEKDGAAPEPKRGKRLMVKEEKSDSD

>ANP93608.1 tumor antigen p53 [Ctenopharyngodon idella]

MAENPESQEFADLWERNLISAPEGGSCWDISDEYLPNSFDPSLFNLPTEQPQPSTSPPTSTVPIATDYPGEHGFKLQFPQSGTAKSVTCTYSPDLNKLFCQLAKTCPVQMVVDVAPPQGSVLRATAIYKKSEHVAEVVRRCPHHERTPDTDGLAPAAHLIRVEGNLRATYKEDDVTSRHSVVVPYEAPQLGAGFTTVLYNYMCNSSCMGGMNRRPILTIITLETQDGQMLGRRSFEVRVCACPGRDRKTEESNFRKDQETKTPSKTPSTTKRSIVKESSSSTSRPEGSKKAKMSVSSDEEIFSLQVRGRERFEMLKKINDSLELSDVVPPSDVDKYRQKLVSKNKKERDGQTPEPKRGKKLMVKDEKSDSD

>KAA0716929.1 Cellular tumor antigen p53 [Triplophysa tibetana]

MAENTESQDFAELWSQNLMSETIETPECVFWETSLNCDEYLHSSFDRNFFDNVPTEQPQPSTSPPTSTVPVSTDYPGEHGFRLAFPQSGTAKSVTCTYSLELNKLFCQLAKTCPVQMVVNTAPPQGSVLRAIAIYKKSEHVAEVVRRCPHHERTPDTDVLAPPAHLIRVEGNMRAVYKQDDVAYRHSVMVPYEPPQLGAECTTILYNFMCNSSCMGGMNRRPILTIITLETQDGQLLGRRSFEVRVCACPGRDRKTEEGNFRKEQESKTSDKTPSTTKRSFKESFSSNPRAEGSKKAKLNNSSDEEMFTLQVRGKERYEMLKKINDSLELSDVVPPSDVDKYRQKLVSKSKKEKDGQTPEPKRGKKLMVKEEKSDSD

>XP_023680464.1 cellular tumor antigen p53 isoform X1 [Paramormyrops kingsleyae]

MDSDAAPSSLFSSDLVFEPCCRNVFGVCNIIDDRFALPLCPSCFCKQMSAADREAGDNMAEQDDNLQFSQTFQDLWNLMPAPNESENWEDIGHLIDASLFEATPPQPSVSPPECGAPPSSTVPTTTDYPGSLDFQLRFTQSSTTKSVTSTYSSILNKLYCQLAKTCPVQMLVSQPPPPGSILRATAVYKKSEHVAEVVRRCPHHERIPENNEGPAPKGHLIRVEGSQRAQYIDDSNTLRHSVVVPYEPPQVGSECTTILYNYMCNSSCMGGMNRRPILTIITLESPDGRLLGRRSFEVRVCACPGRDRKTEEENNSKLQEKSTSKTSGGTKRALKEPSQTGPRPEPSKKTKSSSSTDEETYTLQIRGKERYEMLKMINQSLELGDLVPLADQEKYRQKLQSKVGKREKEREAPEPKRGKKLLVKEEKSDSD

>XP_022519420.1 cellular tumor antigen p53 isoform X1 [Astyanax mexicanus]

MESLAAMEEPREPSESQEFAELWLQNLMVEQPENSSWVNDEYLPEDLQGVFNEPLFEVASDSPQPSTSPPTPTIPVATDYPGVHGFTLRFQQSGTAKSVTSTYSPDLNKLYCQLAKTCPVQMEVNSPPPLGAMLRATAVYKKSQHIAEVVRRCPHHERAAENNDGLAPPGHLLRVEGNPHALYQEDDNTHRHSVLVPYEHPQVGLESTTILYNYMCNSSCMGGMNRRPIMTIITLETQEGQLLGRRSFEVRVCACPGRDRKTEECNFKKQQEPKTTSSSKTPASNKRSLKEAPSRPDCSKKAKTGSSSDEELYTLQVRGRERFEFLKKINDGLELSDLVPPADAEKYRQKLLCKSSRKEKEAAAPEPKRGKKLLVKEEKSNSE

>ARJ54144.1 P53 [Gobiocypris rarus]

MAENPESQEFADLWERNLISAPEGSPWDINDEYPPFDPSIFNLISEQSQPSTSPPTSTVPIATDYPGDYGFKLQFPQSGTAKSVTCTYSPDLNKLFCQLAKTCPVQMVVDFAPPQGSVLRATAIYKKSEHVAEVVRRCPHHERTPDTDGLAPPAHLIRVEGNLRAIYKVDDITSRHSVMVLYEAPQLGAGFTTVLYNYMCNSSCMGGMNRRPILTIITLETQDGQMLGRRSFEVRVCACPGRDRKTEESNFRKDQETKTMSKTPSTTKRGMIKESTMSRPEGSKRAKLSASSDEEIYTLQVKGKERFDMLKKINDSLELTDVVPPCDVEKYRQKLLSKSKKERDGQTPEPKRGKKLMVKDEKSDSD

>XP_026066397.1 cellular tumor antigen p53 isoform X1 [Carassius auratus]

MAESQEFADLWEKNLISTPEAGTCWELINDEQYLPSPFDPNIFDNVQTEQPQPSTSPPTASVPVATDYPGEHGFKLGFPQSGTAKSVTCTYSSDLNKLFCQLAKTCPVQMMVDVAPPQGSVVRATAIYKKSEHVAEVVRRCPHHERTPDGDGLAPAAHLIRVEGNSRALYREDEVNLRHSVVVPYEAPQLGAEFTTVLFNFMCNSSCMGGMNRRPILTIITLETHDGQLLGRGSFEVRVCACPGRDRKTEESNFRKDQETKTSGKTPSSNKRSLTKESTSSVPRPEGSKKAKLSGSSDEEMYNLQVRGKERYEILKMINDSLELSDVVPPSEIDRYRQKILAKGKKEKDGQTPEPKRGKKLLVKDEKSDSD

>XP_028839744.1 cellular tumor antigen p53 isoform X1 [Denticeps clupeoides]

MNLDGDERYLSTTHFSPRPPRPRCCCCCAGSSLWAAMDEPGDSQEFADLWNLNLIELPTENGDWDDVFGRTGLEFPVPAAGFAQDGGSPPGSTVPTTSDYPGDHSFHLSFPHSSCAKSVTCTYSPDLNKLFCQLAKTCPVQMVVEQPPPPGAMVRATAVYKKSDHVAEVVKRCPHHERTPENNDGAAPPSHLIRVEGNQRALYHEDANTFRQSVVVPYEPPQLGSEWTTVLYNFMCNSSCMGGMNRRPILTIITLETNEGLVLGRRSFEVRVCACPGRDRKTEESNFRKQQETSAASQVSVSSSKRSAKEAPSSDVSAAKKAKSSSSTEEEVFTLQVHGRERYLMLKKINDSLELNDLVPPADVDKYRQRLNPKSSNKRDKEPKRGKRLLLKEEKSDSDSVNE

**>XP_005165158.1 cellular tumor antigen p53 isoform X1 [Danio rerio]**

MAQNDSQEFAELWEKNLISIQPPGGGSCWDIINDEEYLPGSFDPNFFENVLEEQPQPSTLPPTSTVPETSDYPGDHGFRLRFPQSGTAKSVTCTYSPDLNKLFCQLAKTCPVQMVVDVAPPQGSVVRATAIYKKSEHVAEVVRRCPHHERTPDGDNLAPAGHLIRVEGNQRANYREDNITLRHSVFVPYEAPQLGAEWTTVLLNYMCNSSCMGGMNRRPILTIITLETQEGQLLGRRSFEVRVCACPGRDRKTEESNFKKDQETKTMAKTTTGTKRKSSSATLRPEGSKKAKGSSSDEEIFTLQVRGRERYEILKKLNDSLELSDVVPASDAEKYRQKFMTKNKKENRESSEPKQGKKLMVKDEGRSDSD

>RXN32067.1 cellular tumor antigen p53-like isoform X1 [Labeo rohita]

MLAPPIPTIMPALLERPKLSNAMARALHKHIMRERERKRQEETKEQVHLCDCCILILGLFIMKMGVKLQGLQEEKHQLFLQLKKVLHEEEKRRRKEQSDMTTLTSATYQPNMAIHTGQHLLSMQAGQVSHGRPGALLGERSKQLFQSPVIPTRHFQSQPGFSAGGSEHGQYSGAQPSHSPYGVSQPQHTSPFASSQPVPANYASGSQLRGASAFQAMQYLPHQQQGYPVHSHFTSQPGYIPSAGIPLQKQLEHANQQSGFTDSSPLRPMHPQALHVSAAGLLPTPSIAVQIPPGKSGLPYAHPPRPASPGAFTHGTPSQQAHAATFQSSSQPTPRHTYLSHSQPGQSFENLKAMAESQEFADLWERNLISTPEGGPCWDLINDEQYLPSSFDPNIFDNVLAEQPQPSTSPPTASVPIATDYPGEHGFKLGFPQSGTAKSVTCTYSSDLNKLYCQLAKTCPVQMVVNTAPPQGSVIRATAIYKKSEHVAEVVRRCPHHERTPDGDGLAPAAHLIRVEGNSRALYREDDVTARHSVVVPYEAPQLGAEFTTILYNYMCNSSCMGGMNRRPILTIITLETQDGQLLGRRSFEVRVCACPGRDRKTEESNRKDQETKTLGKTPSTTKRSMTKESTTSTPRPEGSKKAKLGTSSDEEIYTLQVRGKERYEMLKKINDSLELSDVVPPSEMDRYRQKL

>KAG1958547.1 cellular tumor antigen p53 [Pimephales promelas]

MDDCCPNKTQYKIRPVPLSISTVVSPLMLLLLREIYETAVFLLKLLSVSNRQDSTKFCLPINFANLKQDMAENPESQEFADLWERNLISAPEGGPCWDLSDEYLTSSLDPSFFSLLTEPPQPSTSPPTSTVPIATDYPGDHGFKLQFPQSGTAKSVTCTYSPELNKLFCQLAKTCPVQMVVDVAPPQGSLLRATAIYKKSEHVADVVRRCPHHERALDTDGLAPPAHLIRVEGNLRANYKEDDITSRHSVVVPYESPQLGAEFITILYNYMCNSSCMGGMNRRPILTIITLETQDGQILGRRSFEVRVCACPGRDRKTEESNFRKDQETKTVSKTPSTTKRSLMKESSSSTSRPEGSKKAKLSASSDEEIYTLQVRGKERFEMLKKINDGLELSDVVPPCDVDKYRQKILSKTKKEKDGQTPEPKRGKKLMVKDEKSDSD

>XP_012690246.2 cellular tumor antigen p53-like isoform X1 [Clupea harengus]

MNDQGDSPDFEDLWNSIVPPVADVPWETSYLPESFDEQVFEQLQVSEPYIPTLGGGSPPCSAVPITTDYPGEHDFQLSFPQSSYAKSVTCTYSTDLNKLYCQLAKTCPVHMVVGQPPPPGSVLRATAVYKKSEHVAEVVRRCPHHERTPGNNDGPVPPSHLIRVEGNPRADYQQDLNIGRQSVVVPYEQPQLGCGHTTTLYNYMCNSSCMGGMNRRAILMIVTLETQEGLVLGRRSFEVRVCACPGRDRKTEETNFRKIQETKSSVKATATAAPTTKRSLKEAPQAAAQPEGSKKEEIFKLEVRGRERYEMLKKINDSLELNDLVPSSDIEKYRQKHSSKGGNKRERDGCTMEPKRGKRRLVKEEKSDSD

>NP_001187005.1 cellular tumor antigen p53 [Ictalurus punctatus]

MEGNGERDTMMVEPPDSQEFAELWLRNLIVRDNSLWGKEEEIPDDLQEVPCDVLLSDMLQPQSSSSPPTSTVPVTSDYPGLLNFTLHFQESSGTKSVTCTYSPDLNKLFCQLAKTCPVLMAVSSSPPPGSVLRATAVYKRSEHVAEVVRRCPHHERSNDSSDGPAPPGHLLRVEGNSRAVYQEDGNTQAHSVVVPYEPPQVGSQSTTVLYNYMCNSSCMGGMNRRPILTIITLETQDGHLLGRRTFEVRVCACPGRDRKTEESNFKKQQEPKTSGKTLTKRSMKDPPSHPEASKKSKNSSSDDEIYTLQVRGKERYEFLKKINDGLELSDVVPPADQEKYRQKLLSKTCRKERDGAAGEPKRGKKRLVKEEKCDSD

>KAF5891138.1 cellular tumor antigen p53 isoform X1 [Clarias magur]

MDTAIPSTSDFGDFDVFDDEAESSLFALLSPDCVCAYPARMEGLGEGVTMMPEPTGSQEFAEIWQQILSNPDDSPWANYQDIPEDLPDVTLLEDVLEPQSSSSPPTSTVPVTSDYPGLHDFTLHFQESSTAKSVTCTYSPDLNKLFCQLAKTCPVLMAVRSSPPPGCVLRATAVYKRSEHVAEVVRRCPHHERSNDNNEGPAPPGHLLRVEGNTRALYQEDRNTQRHSVVVPYEPPQVGSSCTTVLYNYMCNSSCMGGMNRRPILTIITLETQDGQLLGRRTFEVRVCACPGRDRKTEEINFRKLQEPKNSGKTVTKRSLKDPSSHPEASKKSKSSSDDEIYTLQVRGRERYEFLKRINDGLELSDLVPPADQEKYRQKLLSKTSKKERDGAATEPKRGKKRLVKEEKSDSD

**>AEW46988.1 tumor protein p53 [Callorhinchus milii]**

MEDELLEEPLSQETFGDLWNQLDFPPIRAEETLPWPNVDPGWGDSALEELNRVWLVTGETSGGYTEPLGPAQVQPPAEPPVLTTSAVPSITDYAGPHNFQLLFQQFSTAKSVTNTYSTSLTKLFCQIAKTCPIQVKVSSPPPPGSVIRATAVYKKSEHVAEVVKRCPHHERCPDFNDGLAPPSHLIRVEANSLARYHDDEHSKRQSVVLPYEEPQVGSECSTVLYNYMCNSSCMGGMNRRPILTIITLETPDGRLVGRRCFEVRVCACPGRDRRYEEENQRKQCEKLTVKRSIKEVSQPTVSTEPSKRKLSSDTEVFTLQVTGRERYETLKQINESLEVQELVPASVVQACRQQHKLRLKAAHKKESSASEPKKGRKLPLKDEVDSE

>XP_041034951.1 cellular tumor antigen p53 isoform X1 [Carcharodon carcharias]

MSESQLDEPLSQETFRELWNQLEVPSANVGLENELQIWDNEFSGLELAMEELDNNPLEFPVLPDNPLPYPSSSQAGPATDIHVAAPCTVLATTEYPGPHEFQLQFQQSSTAKSVTCTYSPSLNKLFCQLAKTCPVQVVVASVPPTGTLLRATAVYKKPEHVAEVVKRCPHHERGSETDGPAPPSHLIRVEANSRARYAEDEHTKRQSVIVPYESPQVGSDYTTVLYNFMCNSSCMGGMNRRPILSILTLETPDGHLLGRRCFEVRVCACPGRDRKSEEENLKRQQENSMVKSGGSATKRTIKEVSQATTSPDSRKKKALSDDEVFTLQVRGRERYELMKKLNEALEISELIPTGVIEAYKQQQKHRLKASHKKEKESTEIKNGKKLLVKDERDSD

>XP_041947523.1 cellular tumor antigen p53 isoform X1 [Alosa sapidissima]

MNDQGNSQDFEDLWNSMVQPTADVNWETSYLSESHFDEQVFEQLHVSEPYLSAPDGGGAPPCSTVPTTTDYPGVHGFQLRFPKSSFAKSVTCTYSTDLNKLYCQLAKTCHIQMVVETLPPPGSMLRATAVYKKSEHVAEVVRRCPHHERTPENNDGQPPPSHLIRVEGNLHAVYQQDPNTGRQSVVVPYEQPQLGCEYTTTLYNYMCNSSCMGGMNRRPILTIVTLETQEGVVLGRRCFEVRVCACPGRDRKTEETNFRKIQEAKPSVKTTATPKRSLKETPQAAAHPEGSKKAKSGSSTEEEIFHLQVRGRERYEMLKKINDGLELNDLVPPSDAEKYRQRHSSKGGNKRERDGHTMEPKRGKKPLVKGEKSDSD
