# Supplementary material for: Phylogenetic analysis of the MCL1 BH3 binding groove and rBH3 sequence motifs in the p53 and INK4 protein families
Source: PLoS One. 2023 Jan 25;18(1):e0277726. doi: 10.1371/journal.pone.0277726 (PMC9876281; doi:10.1371/journal.pone.0277726)
Supplement: S2 File — A total of 213 p63 sequences were used to generate the p53 family phylogenetic tree. (DOCX) [file pone.0277726.s006.docx]

**>NP_001120731.1 tumor protein 63 isoform a [Mus musculus]**

MNFETSRCATLQYCPDPYIQRFIETPAHFSWKESYYRSAMSQSTQTSEFLSPEVFQHIWDFLEQPICSVQPIELNFVDEPSENGATNKIEISMDCIRMQDSDLSDPMWPQYTNLGLLNSMDQQIQNGSSSTSPYNTDHAQNSVTAPSPYAQPSSTFDALSPSPAIPSNTDYPGPHSFDVSFQQSSTAKSATWTYSTELKKLYCQIAKTCPIQIKVMTPPPQGAVIRAMPVYKKAEHVTEVVKRCPNHELSREFNEGQIAPPSHLIRVEGNSHAQYVEDPITGRQSVLVPYEPPQVGTEFTTVLYNFMCNSSCVGGMNRRPILIIVTLETRDGQVLGRRCFEARICACPGRDRKADEDSIRKQQVSDSAKNGDGTKRPFRQNTHGIQMTSIKKRRSPDDELLYLPVRGRETYEMLLKIKESLELMQYLPQHTIETYRQQQQQQHQHLLQKQTSMQSQSSYGNSSPPLNKMNSMNKLPSVSQLINPQQRNALTPTTMPEGMGANIPMMGTHMPMAGDMNGLSPTQALPPPLSMPSTSHCTPPPPYPTDCSIVSFLARLGCSSCLDYFTTQGLTTIYQIEHYSMDDLASLKIPEQFRHAIWKGILDHRQLHDFSSPPHLLRTPSGASTVSVGSSETRGERVIDAVRFTLRQTISFPPRDEWNDFNFDMDSRRNKQQRIKEEGE

>XP_021065267.1 tumor protein 63 isoform X1 [Mus pahari]

MNFETSRCATLQYCPDPYIQRFIETPAHFSWKESYYRSAMSQSTQTSEFLSPEVFQHIWDFLEQPICSVQPIDLNFVDEPSENGATNKIEISMDCIRMQDSDLSDPMWPQYTNLGLLNSMDQQIQNGSSSTSPYNTDHAQNSVTAPSPYAQPSSTFDALSPSPAIPSNTDYPGPHSFDVSFQQSSTAKSATWTYSTELKKLYCQIAKTCPIQIKVMTPPPQGAVIRAMPVYKKAEHVTEVVKRCPNHELSREFNEGQIAPPSHLIRVEGNSHAQYVEDPITGRQSVLVPYEPPQVGTEFTTVLYNFMCNSSCVGGMNRRPILIIVTLETRDGQVLGRRCFEARICACPGRDRKADEDSIRKQQVSDSAKNGDGTKRPFRQNTHGIQMTSIKKRRSPDDELLYLPVRGRETYEMLLKIKESLELMQYLPQHTIETYRQQQQQQHQHLLQKQTSMQSQSSYGNSSPPLNKMNSMNKLPSVSQLINPQQRNALTPTTMPEGMGANIPMMGTHMPMAGDMNGLSPTQALPPPLSMPSTSHCTPPPPYPTDCSIVSFLARLGCSSCLDYFTTQGLTTIYQIEHYSMDDLASLKIPEQFRHAIWKGILDHRQLHDFSSPPHLLRTPSGASTVSVGSSETRGERVIDAVRFTLRQTISFPPRDEWNDFNFDMDSRRNKQQRIKEEGE

>NP_062094.1 tumor protein 63 isoform a [Rattus norvegicus]

MNFETSRCATLQYCPDPYIQRFIETPSHFSWKESYYRSAMSQSTQTSEFLSPEVFQHIWDFLEQPICSVQPIDLNFVDEPSENGATNKIEISMDCIRMQDSDLSDPMWPQYTNLGLLNGMDQQIQNGSSSTSPYNTDHAQNSVTAPSPYAQPSSTFDALSPSPAIPSNTDYPGPHSFDVSFQQSSTAKSATWTYSTELKKLYCQIAKTCPIQIKVMTPPPQGAVIRAMPVYKKAEHVTEVVKRCPNHELSREFNEGQIAPPSHLIRVEGNSHAQYVEDPITGRQSVLVPYEPPQVGTEFTTVLYNFMCNSSCVGGMNRRPILIIVTLETRDGQVLGRRCFEARICACPGRDRKADEDSIRKQQVSDSAKNGDGTKRPFRQNTHGIQMTSIKKRRSPDDELLYLPVRGRETYEMLLKIKESLELMQYLPQHTIETYRQQQQQQHQHLLQKQTSMQSQSSYGNSSPPLNKMNSMNKLPSVSQLINPQQRNALTPTTMPEGMGANIPMMGTHMPMAGDMNGLSPTQALPPPLSMPSTSHCTPPPPYPTDCSIVSFLARLGCSSCLDYFTTQGLTTIYQIEHYSMDDLASLKIPEQFRHAIWKGILDHRQLHDFSSPPHLLRTPSGASTVSVGSSETRGERVIDAVRFTLRQTISFPPRDEWNDFNFDMDSRRNKQQRIKEEGE

>XP_031219412.1 tumor protein 63 isoform X6 [Mastomys coucha]

MNFETSRCATLQYCPDPYIQRFIETPAHFSWKESYYRSAMSQSTQTSEFLSPEVFQHIWDFLEQPICSVQPIDLNFVDEPSENGATNKIEISMDCIRMQDSDLSDPMWPQYTNLGLLNSMDQQIQNGSSSTSPYNTDHAQNSVTAPSPYAQPSSTFDALSPSPAIPSNTDYPGPHSFDVSFQQSSTAKSATWTYSTELKKLYCQIAKTCPIQIKVMTPPPQGAVIRAMPVYKKAEHVTEVVKRCPNHELSREFNEGQIAPPSHLIRVEGNSHAQYVEDPITGRQSVLVPYEPPQVGTEFTTVLYNFMCNSSCVGGMNRRPILIIVTLETRDGQVLGRRCFEARICACPGRDRKADEDSIRKQQVSDSAKNGDGTKRPFRQNTHGIQMTSIKKRRSPDDELLYLPVRGRETYEMLLKIKESLELMQYLPQHTIETYRQQQQQQHQHLLQKQTSMQSQSSYGNSSPPLNKMNSMNKLPSVSQLINPQQRNALTPTTMPEGMGGNLPMMGTHMPMAGDMNGLSPTQALPPPLSMPSTSHCTPPPPYPTDCSIVSFLARLGCSSCLDYFTTQGLTTIYQIEHYSMDDLASLKIPEQFRHAIWKGILDHRQLHDFSSPPHLLRTPSGASTVSVGSSETRGERVIDAVRFTLRQTISFPPRDEWNDFNFDMDSRRNKQQRIKEEGE

>XP_036059905.1 tumor protein 63 isoform X1 [Onychomys torridus]

MNFETPRCATLQYCPDPYIQRFVETPAHFSWKESYYRSAMSQSTQTSEFLSPEVFQHIWDFLEQPICSVQPIDLNFVDEPSENGATNKIEISMDCIRMQDSDLSDPMWPQYTNLGLLNSMDQQIQNGSSSTSPYNTDHAQNSVTAPSPYAQPSSTFDALSPSPAIPSNTDYPGPHSFDVSFQQSSTAKSATWTYSTELKKLYCQIAKTCPIQIKVMTPPPQGAVIRAMPVYKKAEHVTEVVKRCPNHELSREFNEGQIAPPSHLIRVEGNSHAQYVEDPITGRQSVLVPYEPPQVGTEFTTVLYNFMCNSSCVGGMNRRPILIIVTLETRDGQVLGRRCFEARICACPGRDRKADEDSIRKQQVSDNAKNGDGTKRPFRQNTHGIQMTSIKKRRSPDDELLYLPVRGRETYEMLLKIKESLELMQYLPQHTIETYRQQQQQQHQHLLQKQTSMQSQSSYGNSSPPLNKMNSMNKLPSVSQLINPQQRNALTPTTMPEGMGANIPMMGTHMPMAGDMNGLSPTQALPPPLSMPSTSHCTPPPPYPTDCSIVSFLARLGCSSCLDYFTTQGLTTIYQIEHYSMDDLASLKIPEQFRHAIWKGILDHRQLHDFSSPPHLLRTPSGASTVSVGSSETRGERVIDAVRFTLRQTISFPPRDEWNDFNFDMDSRRNKQQRIKEEGE

>XP_032755130.1 tumor protein 63 isoform X1 [Rattus rattus]

MNFETSRCATLQYCPDPYIQRFIETPSHFSWKESYYRSAMSQSTQTSEFLSPEVFQHIWDFLEQPICSVQPIDLNFVDEPSENGATNKIEISMDCIRMQDSDLSDPMWPQYTNLGLLNGMDQQIQNGSSSTSPYNTDHAQNSVTAPSPYAQPSSTFDALSPSPAIPSNTDYPGPHSFDVSFQQSSTAKSATWTYSTELKKLYCQIAKTCPIQIKVMTPPPQGAVIRAMPVYKKAEHVTEVVKRCPNHELSREFNEGQIAPPSHLIRVEGNSHAQYVEDPITGRQSVLVPYEPPQVGTEFTTVLYNFMCNSSCVGGMNRRPILIIVTLETRDGQVLGRRCFEARICACPGRDRKADEDSIRKQQVSDSAKNGDGTKRPFRQNTHGIQMTSIKKRRSPDDELLYLPVRGRETYEMLLKIKESLELMQYLPQHTIETYRQQQQQQHQHLLQKQTSMQSQSSYGNSSPPLNKMNSMNKLPSVSQLINPQQRNALTPTTMPEGMGGNIPMMGTHMPMAGDMNGLSPTQALPPPLSMPSTSHCTPPPPYPTDCSIVSFLARLGCSSCLDYFTTQGLTTIYQIEHYSMDDLASLKIPEQFRHAIWKGILDHRQLHDFSSPPHLLRTPSGASTVSVGSSETRGERVIDAVRFTLRQTISFPPRDEWNDFNFDMDSRRNKQQRIKEEGE

>XP_035300559.1 tumor protein 63 isoform X1 [Cricetulus griseus]

MNFETSRCATLQYCPDPYIQRFVETPAHFSWKESYYRSAMSQSSQTSEFLSPEVFQHIWDFLEQPICSVQPIDLNFVEEPSENGATNKIEISMDCIRMQDSDLTDPMWPQYTNLGLLNSMDQQIQNGSSSTSPYNTDHAQNSVTAPSPYAQPSSTFDALSPSPAIPSNTDYPGPHSFDVSFQQSSTAKSATWTYSTELKKLYCQIAKTCPIQIKVMTPPPQGAVIRAMPVYKKAEHVTEVVKRCPNHELSREFNEGQIAPPSHLIRVEGNSHAQYVEDPITGRQSVLVPYEPPQVGTEFTTVLYNFMCNSSCVGGMNRRPILIIVTLETRDGQVLGRRCFEARICACPGRDRKADEDSIRKQQVSDSAKNGDGTKRPFRQNTHGIQMTSIKKRRSPDDELLYLPVRGRETYEMLLKIKESLELMQYLPQHTIETYRQQQQQQHQHLLQKQTSMQSQSSYGNSSPPLNKMNSMNKLPSVSQLINPQQRNALTPTTMPEGMGANIPMMGTHMPMAGDMNGLSPTQALPPPLSMPSTSHCTPPPPYPTDCSIVSFLARLGCSSCLDYFTTQGLTTIYQIEHYSMDDLASLKIPEQFRHAIWKGILDHRQLHDFSSPPHLLRTPSGASTVSVGSSETRGERVIDAVRFTLRQTISFPPRDEWNDFNFDMDSRRNKQQRIKEEGE

>XP_020009534.1 tumor protein 63 isoform X1 [Castor canadensis]

MNFETSRCATLQYCPDPYIQRFVETPAHFSWKESYYRSTMSQSTQTSEFLSPEVFQHIWDFLEQPICSVQPIDLNFVDEPSENGATNKIEISMDCIRMQDSDLSDPMWPQYTNLGLLNSMDQQIQNGSSSTSPYNTDHAQNSVTAPSPYAQPSSTFDALSPSPAIPSNTDYPGPHSFDVSFQQSSTAKSATWTYSTELKKLYCQIAKTCPIQIKVMTPPPQGAVIRAMPVYKKAEHVTEVVKRCPNHELSREFNEGQIAPPSHLIRVEGNSHAQYVEDPITGRQSVLVPYEPPQVGTEFTTVLYNFMCNSSCVGGMNRRPILIIVTLETRDGQVLGRRCFEARICACPGRDRKADEDSIRKQQVSDSTKNGDGTKRPFRQNTHGIQMTSIKKRRSPDDELLYLPVRGRETYEMLLKIKESLELMQYLPQHTIETYRQQQQQQHQHLLQKQTSMQSQSSYGNSSPPLNKMNSMNKLPSVSQLINPQQRNALTPTTMPDGMGANIPMMGTHMPMAGDMNGLSPTQALPPPLSMPSTSHCTPPPPYPTDCSIVSFLARLGCSSCLDYFTTQGLTTIYQIEHYSMDDLASLKIPEQFRHAIWKGILDHRQLHDFSSPPHLLRTPSGASTVSVGSSETRGERVIDAVRFTLRQTISFPPRDEWNDFNFDMDARRNKQQRIKEEGE

>XP_028639733.1 tumor protein 63 isoform X1 [Grammomys surdaster]

MNFETSRCATLQYCPDPYIQRFIETPAHFSWKESYYRSAMSQSTQPSEFLSPEVFQHIWDFLEQPICSVQPIDLNFVDEPSENGATNKIEISMDCIRMQDSDLSDPMWPQYTNLGLLNSMDQQIQNGSSSTSPYNTDHAQNSVTAPSPYAQPSSTFDALSPSPAIPSNTDYPGPHSFDVSFQQSSTAKSATWTYSTELKKLYCQIAKTCPIQIKVMTPPPQGAVIRAMPVYKKAEHVTEVVKRCPNHELSREFNEGQIAPPSHLIRVEGNSHAQYVEDPITGRQSVLVPYEPPQVGTEFTTVLYNFMCNSSCVGGMNRRPILIIVTLETRDGQVLGRRCFEARICACPGRDRKADEDSIRKQQVSDNAKNGDGTKRPFRQNTHGIQMTSIKKRRSPDDELLYLPVRGRETYEMLLKIKESLELMQYLPQHTIETYRQQQQQQHQHLLQKQTSMQSQSSYGTSSPPLNKMNSMNKLPSVSQLINPQQRNALTPTTMPEGMGANIPMMGTHMPMAGDMNGLSPTQALPPPLSMPSTSHCTPPPPYPTDCSIVSFLARLGCSSCLDYFTTQGLTTIYQIEHYSMDDLASLKIPEQFRHAIWKGILDHRQLHDFSSPPHLLRTPSGASTVSVGSSETRGERVIDAVRFTLRQTISFPPRDEWNDFNFDMDSRRNKQQRIKEEGE

>XP_040858785.1 tumor protein 63 isoform X1 [Ochotona curzoniae]

MNFETSRCATLQYCPDPYIQRFVETPAHFSWKESYYRSAMSQSTQPSEFLSPEVFQHIWDFLEQPICSVQPIDLNFVDEPSENGATNKIEISMDCIRMQDSDLSDPMWPQYTNLGLLNSMDQQIQNGSSSTSPYNTDHAQNSVTAPSPYAQPSSTFDALSPSPAIPSNTDYPGPHSFDVSFQQSSTAKSATWTYSTELKKLYCQIAKTCPIQIKVMTPPPQGAVIRAMPVYKKAEHVTEVVKRCPNHELSREFNEGQIAPPSHLIRVEGNSHAQYVEDPITGRQSVLVPYEPPQVGTEFTTVLYNFMCNSSCVGGMNRRPILIIVTLETRDGQVLGRRCFEARICACPGRDRKADEDSIRKQQVSDSAKNGDGTKRPFRQNTHGIQMTSIKKRRSPDDELLYLPVRGRETYEMLLKIKESLELMQYLPQHTIETYRQQQQQQHQHLLQKQTSIQSQSSYGNSSPPLNKMNSMNKLPSVSQLINPQQRNALTPTTIPDGMGANIPMMGTHMPMAGDMNGLSPTQALPPPLSMPSTSHCTPPPPYPTDCSIVSFLARLGCSSCLDYFTTQGLTTIYQIEHYSMDDLATLKIPEQFRHAIWKGILDHRQLHDFSSPPHLLRTPSGASTVSVGSSETRGERVIDAVRFTLRQTISFPPRDEWNDFNFDMDARRNKQQRIKEEGE

>XP_008069725.1 tumor protein 63 isoform X1 [Carlito syrichta]

MNFETSRCATLQYCPDPYIQRFVETPAHFSWKESYYRSTMSQSTQTSEFLSPEVFQHIWDFLEQPICSVQPIDLNFVDEPSENGPTNKIEISMDCIRMQDSDLSDPMWPQYTNLGLLNSMDQQIQNGSSSTSPYNTDHAQNSVTAPSPYAQPSSTFDALSPSPAIPSNTDYPGPHSFDVSFQQSSTAKSATWTYSTELKKLYCQIAKTCPIQIKVMTPPPQGAVIRAMPVYKKAEHVTEVVKRCPNHELSREFNEGQIAPPSHLIRVEGNSHAQYVEDPITGRQSVLVPYEPPQVGTEFTTVLYNFMCNSSCVGGMNRRPILIIVTLETRDGQVLGRRCFEARICACPGRDRKADEDSIRKQQVSDSTKNGDGTKRPFRQNTHGIQMTSIKKRRSPDDELLYLPVRGRETYEMLLKIKESLELMQYLPQHTIETYRQQQQQQHQHLLQKQTSMQSQSSYGNSSPPLNKMNSMNKLPSVSQLINPQQRNALTPTTIPDGMGANIPMMGTHMPMAGDMNGLSPTQALPPPLSMPSTSHCTPPPPYPTDCSIVSFLARLGCSSCLDYFTTQGLTTIYQIEHYSMDDLASLKIPEQFRHAIWKGILDHRQLHDFSSPPHLLRTPSGASTVSVGSSETRGERVIDAVRFTLRQTISFPPRDEWNDFNFDMDARRNKQQRIKEEGE

>XP_025142353.1 tumor protein 63 isoform X1 [Bubalus bubalis]

MNFETSRCATLQYCPDPYIQRFVETPAHFSWKESYYRSTMSQSTQTSEFLSPEVFQHIWDFLEQPICSVQPIDLNFVDEPSENGATNKIEISMDCIRMQDSDLGDPMWPQYTNLGLLNSMDQQIQNGSSSTSPYNTDHAQNSVTAPSPYAQPSSTFDALSPSPAIPSNTDYPGPHSFDVSFQQSSTAKSATWTYSTELKKLYCQIAKTCPIQIKVMTPPPQGAVIRAMPVYKKAEHVTEVVKRCPNHELSREFNEGQIAPPSHLIRVEGNSHAQYVEDPITGRQSVLVPYEPPQVGTEFTTVLYNFMCNSSCVGGMNRRPILIIVTLETRDGQVLGRRCFEARICACPGRDRKADEDSIRKQQVSDSTKNGDGTKRPFRQNTHGIQMTSIKKRRSPDDELLYLPVRGRETYEMLLKIKESLELMQYLPQHTIETYRQQQQQQHQHLLQKQTSMQSQSSYGNSSPPLNKMNSMNKLPSVSQLINPQQRNALTPTTIPDGMGANIPMMGTHMPMAGDMNGLSPTQALPPPLSMPSTSHCTPPPPYPTDCSLVSFLARLGCSSCLDYFTTQGLTTIYQIEHYSMDDLASLKIPEQFRHAIWKGILDHRQLHDFSSPPHLLRTPSGASTVSVGSSETRGERVIDAVRFTLRQTISFPPRDEWNDFNFDMDSRRNKQQRIKEEGE

>XP_040606948.1 tumor protein 63 isoform X1 [Mesocricetus auratus]

MNFETSRCATLQYRPDPYIQRFVETPAHFSWKESYYRSAMSQSSQTSEFLSPEVFQHIWDFLEQPICSVQPIDLNFVEEPSENGATNKIEISMDCIRMQDSDLSDPMWPQYTNLGLLNSMDQQIQNGSSSTSPYNTDHAQNSVTAPSPYAQPSSTFDALSPSPAIPSNTDYPGPHSFDVSFQQSSTAKSATWTYSTELKKLYCQIAKTCPIQIKVMTPPPQGAVIRAMPVYKKAEHVTEVVKRCPNHELSREFNEGQIAPPSHLIRVEGNSHAQYVEDPITGRQSVLVPYEPPQVGTEFTTVLYNFMCNSSCVGGMNRRPILIIVTLETRDGQVLGRRCFEARICACPGRDRKADEDSIRKQQVSDSAKNGDGTKRPFRQNTHGIQMTSIKKRRSPDDELLYLPVRGRETYEMLLKIKESLELMQYLPQHTIETYRQQQQQQHQHLLQKQTSMQSQSSYGNSSPPLNKMNSMNKLPSVSQLINPQQRNALTPTTMPEGMGANIPMMGTHMPMTGDMNGLSPTQALPPPLSMPSTSHCTPPPPYPTDCSIVSFLARLGCSSCLDYFTTQGLTTIYQIEHYSMDDLASLKIPEQFRHAIWKGILDHRQLHDFSSPPHLLRTPSGASTVSVGSSETRGERVIDAVRFTLRQTISFPPRDEWNDFNFDMDSRRNKQQRIKEEGE

>XP_020728485.1 tumor protein 63 isoform X1 [Odocoileus virginianus texanus]

MNFETSRCATLQYCPDPYIQRFVETPAHFSWKESYYRSTMSQSTQTSEFLSPEVFQHIWDFLEQPICSVQPIDLNFVDEPSENGATNKIEISMDCIRMQDSDLSDPMWPQYTNLGLLNSMDQQIQNGSSSTSPYNTDHAQNSVTAPSPYAQPSSTFDALSPSPAIPSNTDYPGPHSFDVSFQQSSTAKSATWTYSTELKKLYCQIAKTCPIQIKVMTPPPQGAVIRAMPVYKKAEHVTEVVKRCPNHELSREFNEGQIAPPSHLIRVEGNSHAQYVEDPITGRQSVLVPYEPPQVGTEFTTVLYNFMCNSSCVGGMNRRPILIIVTLETRDGQVLGRRCFEARICACPGRDRKADEDSIRKQQVSDSTKNGDGTKRPFRQNTHGIQMTSIKKRRSPDDELLYLPVRGRETYEMLLKIKESLELMQYLPQHTIETYRQQQQQQHQHLLQKQTSMQSQSSYGNSSPPLNKMNSMNKLPSVSQLINPQQRNALTPTTIPDGMGANIPMMGTHMPMAGDMNGLSPTQALPPPLSMPSTSHCTPPPPYPTDCSLVSFLARLGCSSCLDYFTTQGLTTIYQIEHYSMDDLASLKIPEQFRHVIWKGILDHRQLHDFSSPPHLLRTPSGASTVSVGSSETRGERVIDAVRFTLRQTISFPPRDEWNDFNFDMDARRNKQQRIKEEGE

>XP_028017903.1 tumor protein 63 isoform X1 [Balaenoptera acutorostrata scammoni]

MNFETSRCATLQYCPDPYIQRFVETPAHFSWKESYYRSTMSQSTQTSEFLSPEVFQHIWDFLEQPICSVQPIDLNFVDEPSENGATNKIEISMDCIRMQDSELSDPMWPQYTNLGLLNSMDQQIQNGSSSTSPYNTDHAQNSVTAPSPYAQPSSTFDALSPSPAIPSNTDYPGPHSFDVSFQQSSTAKSATWTYSTELKKLYCQIAKTCPIQIKVMTPPPQGAVIRAMPVYKKAEHVTEVVKRCPNHELSREFNEGQIAPPSHLIRVEGNSHAQYVEDPITGRQSVLVPYEPPQVGTEFTTVLYNFMCNSSCVGGMNRRPILIIVTLETRDGQVLGRRCFEARICACPGRDRKADEDSIRKQQVSDSTKNGDGTKRPFRQNTHGIQMTSIKKRRSPDDELLYLPVRGRETYEMLLKIKESLELMQYLPQHTIETYRQQQQQQHQHLLQKQTSMQSQSSYGNSSPPLNKMNSMNKLPSVSQLINPQQRNALTPTTIPDGMGANIPMMGTHMPMAGDMNGLSPTQALPPPLSMPSTSHCTPPPPYPTDCSLVSFLARLGCSSCLDYFTTQGLTTIYQIEHYSMDDLASLKIPEQFRHAIWKGILDHRQLHDFSSPPHLLRTPSGASTVSVGSSETRGERVIDAVRFTLRQTISFPPRDEWNDFNFDMDARRNKQQRIKEEGE

>XP_012600634.1 tumor protein 63 isoform X1 [Microcebus murinus]

MNFETSRCATLQYCPDPYIQRFVETPAHFSWKESYYRSTMSQSTQTSEFLSPEVFQHIWDFLEQPICSVQPIDLNFVDEPSENGATNKIEISMDCIHMQDSDLSDPMWPQYTNLGLLNSMDQQIQNGSSSTSPYNTDHAQNSVTAPSPYAQPSSTFDALSPSPAIPSNTDYPGPHSFDVSFQQSSTAKSATWTYSTELKKLYCQIAKTCPIQIKVMTPPPQGAVIRAMPVYKKAEHVTEVVKRCPNHELSREFNEGQIAPPSHLIRVEGNSHAQYVEDPITGRQSVLVPYEPPQVGTEFTTVLYNFMCNSSCVGGMNRRPILIIVTLETRDGQVLGRRCFEARICACPGRDRKADEDSIRKQQVSDSTKNGDGTKRPFRQNTHGIQMTSIKKRRSPDDELLYLPVRGRETYEMLLKIKESLELMQYLPQHTIETYRQQQQQQHQHLLQKQTSMQSQSSYGNSSPPLNKMNSMNKLPSVSQLINPQQRNALTPTTIPDGMGANIPMMGTHMPMAGDMNGLSPTQALPPPLSMPSTSHCTPPPPYPTDCSIVSFLARLGCSSCLDYFTTQGLTTIYQIEHYSMDDLASLKIPEQFRHAIWKGILDHRQLHDFSSPPHLLRTPSGASTVSVGSSETRGERVIDAVRFTLRQTISFPPRDEWNDFNFDMDTRRNKQQRIKEEGE

>XP_004003090.1 tumor protein 63 isoform X1 [Ovis aries]

MNFETSRCATLQYCPDPYIQRFVETPAHFSWKESYYRSTMSQSTQTNEFLSPEVFQHIWDFLEQPICSVQPIDLNFVDEPSENGATNKIEISMDCIRMQDSDLSDPMWPQYTNLGLLNSMDQQIQNGSSSTSPYNTDHAQNSVTAPSPYAQPSSTFDALSPSPAIPSNTDYPGPHSFDVSFQQSSTAKSATWTYSTELKKLYCQIAKTCPIQIKVMTPPPQGAVIRAMPVYKKAEHVTEVVKRCPNHELSREFNEGQIAPPSHLIRVEGNSHAQYVEDPITGRQSVLVPYEPPQVGTEFTTVLYNFMCNSSCVGGMNRRPILIIVTLETRDGQVLGRRCFEARICACPGRDRKADEDSIRKQQVSDSTKNGDGTKRPFRQNTHGIQMTSIKKRRSPDDELLYLPVRGRETYEMLLKIKESLELMQYLPQHTIETYRQQQQQQHQHLLQKQTSMQSQSSYGNSSPPLNKMNSMNKLPSVSQLINPQQRNALTPTTIPDGMGANIPMMGTHMPMAGDMNGLSPTQALPPPLSMPSTSHCTPPPPYPTDCSLVSFLARLGCSSCLDYFTTQGLTTIYQIEHYSMDDLASLKIPEQFRHAIWKGILDHRQLHDFSSPPHLLRTPSGASTVSVGSSETRGERVIDAVRFTLRQTISFPPRDEWNDFNFDMDARRNKQQRIKEEGE

>NP_001178266.1 tumor protein 63 [Bos taurus]

MNFETSRCATLQYCPDPYIQRFVETPAHFSWKESYYRSTMSQSTQTSEFLSPEVFQHIWDFLEQPICSVQPIDLNFVDEPSENGATNKIEISMDCIRMQDSDLGDPMWPQYTNLGLLNSMDQQIQNGSSSTSPYNTDHAQNSVTAPSPYAQPSSTFDALSPSPAIPSNTDYPGPHSFDVSFQQSSTAKSATWTYSTELKKLYCQIAKTCPIQIKVMTPPPQGAVIRAMPVYKKAEHVTEVVKRCPNHELSREFNEGQIAPPSHLIRVEGNSHAQYVEDPITGRQSVLVPYEPPQVGTEFTTVLYNFMCNSSCVGGMNRRPILIIVTLETRDGQVLGRRCFEARICACPGRDRKADEDSIRKQQVSDSTKNGDGTKRPFRQNTHGIQMTSIKKRRSPDDELLYLPVRGRETYEMLLKIKESLELMQYLPQHTIETYRQQQQQQHQHLLQKQTSMQSQSSYGNSSPPLNKMNSMNKLPSVSQLINPQQRNALTPTTIPDGMGANIPMMGTHMPMAGDMNGLSPTQALPPPLSMPSTSHCTPPPPYPTDCSLVSFLARLGCSSCLDYFTTQGLTTIYQIEHYSMDDLASLKIPEQFRHAIWKGILDHRQLHDFSSPPHLLRTPSGASTVSVGSSETRGERVIDAVRFTLRQTISFPPRDEWNDFNFDMDARRNKQQRIKEEGE

>XP_026245117.1 tumor protein 63 isoform X6 [Urocitellus parryii]

MNFETSRCATLQYCPDPYIQRFVETPAHFSWKESYYRSTMSQSTQTSEFLSPEVFQHIWDFLEQPICSVQPIDLNFVDEPSENGATNKIEISMDCIRMQDSDLSDPMWPQYTNLGLLNSMDQQIQNGSSSTSPYNTDHAQNSVTAPSPYAQPSSTFDALSPSPAIPSNTDYPGPHSFDVSFQQSSTAKSATWTYSTELKKLYCQIAKTCPIQIKVMTPPPQGAVIRAMPVYKKAEHVTEVVKRCPNHELSREFNEGQIAPPSHLIRVEGNSHAQYVEDPITGRQSVLVPYEPPQVGTEFTTVLYNFMCNSSCVGGMNRRPILIIVTLETRDGQVLGRRCFEARICACPGRDRKADEDSIRKQQVSDSTKNGDGTKRPFRQNTHGIQMTSIKKRRSPDDELLYLPVRGRETYEMLLKIKESLELMQYLPQHTIETYRQQQQQQHQHLLQKQTSMQSQSSYGNSSPPLNKMNNMNKLPSVSQLINPQQRNALTPTTIPDGMGANIPMMSTHMPMAGDMNGLSPTQALPPPLSMPSTSHCTPPPPYPTDCSIVSFLARLGCSSCLDYFTTQGLTTIYQIEHYSMDDLASLKIPEQFRHAIWKGILDHRQLHDFSSPPHLLRTPSGASTVSVGSSETRGERVIDAVRFTLRQTISFPPRDEWNDFNFDMDARRNKQQRIKEEGE

>XP_022425353.1 tumor protein 63 isoform X1 [Delphinapterus leucas]

MNFETSRCATVQYCPDPYIQRFVETPAHFSWKESYYRSTMSQSTQTSEFLSPEVFQHIWDFLEQPICSVQPIDLNFVDEPSENGATNKIEISMDCIRMQDSDLSDPMWPQYTNLGLLNSMDQQIQNGSSSTSPYNTDHAQNSVTAPSPYAQPSSTFDALSPSPAIPSNTDYPGPHSFDVSFQQSSTAKSATWTYSTELKKLYCQIAKTCPIQIKVMTPPPQGAVIRAMPVYKKAEHVTEVVKRCPNHELSREFNEGQIAPPSHLIRVEGNSHAQYVEDPITGRQSVLVPYEPPQVGTEFTTVLYNFMCNSSCVGGMNRRPILIIVTLETRDGQVLGRRCFEARICACPGRDRKADEDSIRKQQVSDSTKNGDGTKRPFRQNTHGIQMTSIKKRRSPDDELLYLPVRGRETYEMLLKIKESLELMQYLPQHTIETYRQQQQQQHQHLLQKQTSMQSQSSYGNSSPPLNKMNSMNKLPSVSQLINPQQRNTLTPTTIPDGMGANIPMMGTHMPMAGDMNGLSPTQALPPPLSMPSTSHCTPPPPYPTDCSLVSFLARLGCSSCLDYFTTQGLTTIYQIEHYSMDDLASLKIPEQFRHAIWKGILDHRQLHDFSSPPHLLRTPSGASTVSVGSSETRGERVIDAVRFTLRQTISFPPRDEWNDFNFDMDARRNKQQRIKEEGE

>XP_006925897.1 tumor protein 63 isoform X1 [Pteropus alecto]

MNFETSRCATLQYCPDPYIQRFVETPAHFSWKESYYRSTMSQSTQTSEFLSPEVFQHIWDFLEQPICSVQPIDLNFVDEPSENGATNKIEISMDCIRMQNSDLSDPMWPQYTNLGLLNSMDQQIQNGSSSTSPYNTDHAQNSVTAPSPYAQPSSTFDALSPSPAIPSNTDYPGPHSFDVSFQQSSTAKSATWTYSTELKKLYCQIAKTCPIQIKVMTPPPQGAVIRAMPVYKKAEHVTEVVKRCPNHELSREFNEGQVAPPSHLIRVEGNSHAQYVEDPITGRQSVLVPYEPPQVGTEFTTVLYNFMCNSSCVGGMNRRPILIIVTLETRDGQVLGRRCFEARICACPGRDRKADEDSIRKQQVSDSTKNGDGTKRPFRQNTHGIQMTSIKKRRSPDDELLYLPVRGRETYEMLLKIKESLELMQYLPQHTIETYRQQQQQQHQHLLQKQTSMQSQSSYGNSSPPLNKMNSMNKLPSVSQLINPQQRNALTPTTIPDGMGANIPMMGTHMPMAGDMNGLSPTQALPPPLSMPSTSHCTPPPPYPTDCSLVSFLARLGCSSCLDYFTTQGLTTIYQIEHYSMDDLASLKIPEQFRHAIWKGILDHRQLHDFSSPPHLLRTPSGTSTVSVGSSETRGERVIDAVRFTLRQTISFPPRDEWNDFNFDMDARRNKQQRIKEEGE

>XP_004834847.1 tumor protein 63 isoform X1 [Heterocephalus glaber]

MNFETSRCATLQYCPDPYIQRFVETPAHFSWKESYYRSTMSQSTQTSEFLSPEVFQHIWDFLEQPICSVQPIDLNFVDESSENGATNKIEISMDCIRMQDSDLSDPMWPQYTNLGLLNSMDQQIQNGSSSTSPYNTEHAQNSVTAPSPYAQPSSTFDALSPSPAIPSNTDYPGPHSFDVSFQQSSTAKSATWTYSTELKKLYCQIAKTCPIQIKVMTPPPQGAVIRAMPVYKKAEHVTEVVKRCPNHELSREFNEGQIAPPSHLIRVEGNSHAQYVEDPITGRQSVLVPYEPPQVGTEFTTVLYNFMCNSSCVGGMNRRPILIIVTLETRDGQVLGRRCFEARICACPGRDRKADEDSIRKQQVSDSTKNGDGTKRPFRQNTHGIQMTSIKKRRSPDDELLYLPVRGRETYEMLLKIKESLELMQYLPQHTIETYRQQQQQQHQHLLQKQTSMQSQASYGNSSPPLNKMNSMNKLPSVSQLINPQQRNALTPTTIPDGMGANIPMMGTHMPMAGDMNGLSPTQALPPPLSMPSTSHCTPPPPYPTDCSIVSFLARLGCSSCLDYFTTQGLTTIYQIEHYSMDDLASLKIPEQFRHAIWKGILDHRQLHDFSSPPHLLRTPSGASTVSVGSSETRGERVIDAVRFTLRQTISFPPRDEWNDFNFDMDARRNKQQRIKEEGE

>XP_029795499.1 tumor protein 63 isoform X1 [Suricata suricatta]

MNCETSRCATLQYCPDPYIQRFVETPAHFSWKESYYRSSMSQSTQTSEFLSPEVFQHIWDFLEQPICSVQPIDLNFVDEPSENGARNKIEISMDCIRMQDSDLSDPMWPQYTNLGLLNSMDQQIQNGSSSTSPYNTDHAQNSVTAPSPYAQPSSTFDALSPSPAIPSNTDYPGPHSFDVSFQQSSTAKSATWTYSTELKKLYCQIAKTCPIQIKVMTPPPQGAVIRAMPVYKKAEHVTEVVKRCPNHELSREFNEGQIAPPSHLIRVEGNSHAQYVEDPITGRQSVLVPYEPPQVGTEFTTVLYNFMCNSSCVGGMNRRPILIIVTLETRDGQVLGRRCFEARICACPGRDRKADEDSIRKQQVSDSAKNGDGTKRPFRQNTHGIQMTSIKKRRSPDDELLYLPVRGRETYEMLLKIKESLELMQYLPQHTIETYRQQQQQQHQHLLQKQTSMQSQSSYGNSSPPLNKMNSMNKLPSVSQLINPQQRNALTPTTIPDGMGANIPMMGTHMPMAGDMNGLSPTQALPPPLSMPSTSHCTPPPPYPTDCSLVSFLARLGCSSCLDYFTTQGLTTIYQIEHYSMDDLASLKIPEQFRHAIWKGILDHRQLHDFSSPPHLLRTPSGASTVSVGSSETRGERVIDAVRFTLRQTISFPPRDEWNDFNFDMDARRNKQQRIKEEGE

>XP_032945909.1 tumor protein 63 isoform X1 [Rhinolophus ferrumequinum]

MNFETSRCATLQYCPDPYIQRFVETPAHFSWKESYYRSTMSQSTQTSEFLSPEVFQHIWDFLEQPICSVQPIDLNFVDEPSENGTTNKIEISMDCIRMQDSDLSDPMWPQYTNLGLLNSMDQQIQNGSSSTSPYNTDHAQNSVTAPSPYAQPSSTFDALSPSPAIPSNTDYPGPHSFDVSFQQSSTAKSATWTYSTELKKLYCQIAKTCPIQIKVMTPPPQGAVIRAMPVYKKAEHVTEVVKRCPNHELSREFNEGQIAPPSHLIRVEGNSHAQYVEDPITGRQSVLVPYEPPQVGTEFTTVLYNFMCNSSCVGGMNRRPILIIVTLETRDGQVLGRRCFEARICACPGRDRKADEDSIRKQQVSDSTKNGDGTKRPFRQNTHGIQMTSIKKRRSPDDELLYLPVRGRETYEMLLKIKESLELMQYLPQHTIETYRQQQQQQHQHLLQKQTSMQSQSSYGNSSPPLNKMNSMNKLPSVSQLINPQQRNALTPTTIPDGMGANIPMMGTHMPMAGDMNGLSPTQALPPPLSMPSTSHCTPPPPYPTDCSLVSFLARLGCSSCLDYFTTQGLTTIYQIEHYSMDDLASLKIPEQFRHVIWKGILDHRQLHDFSSPPHLLRTPSGTSTVSVGSSETRGERVIDAVRFTLRQTISFPPRDEWNDFNFDMDARRNKQQRIKEEGE

>XP_005344823.1 tumor protein 63 isoform X1 [Microtus ochrogaster]

MNFETSRCSTLQYCPDPYFQRFVETPAHFSWKESYYRSAMSQSSQTSELFSPEVFQHIWDFLEQPICSVQPIDLNFVEEPSENGATNKIEISMDCIRMQDSDLTDPMWPQYTNLGLLNSMDQQIQNGSSSTSPYNTDHAQNSVTAPSPYAQPSSTFDALSPSPAIPSNTDYPGPHSFDVSFQQSSTAKSATWTYSTELKKLYCQIAKTCPIQIKVMTPPPQGAVIRAMPVYKKAEHVTEVVKRCPNHELSREFNEGQIAPPSHLIRVEGNSHAQYVEDPITGRQSVLVPYEPPQVGTEFTTVLYNFMCNSSCVGGMNRRPILIIVTLETRDGQVLGRRCFEARICACPGRDRKADEDSIRKQQVSDSAKNGDGTKRPFRQNTHGIQMTSIKKRRSPDDELLYLPVRGRETYEMLLKIKESLELMQYLPQHTIETYRQQQQQQHQHLLQKQTSMQSQSSYGNSSPPLSKMNSMNKLPSVSQLINPQQRNALTPTTMPEGMGANIPMMGTHMPMAGDMNGLSPTQALPPPLSMPSTSHCTPPPPYPTDCSIVSFLARLGCSSCLDYFTTQGLTTIYQIEHYSMDDLASLKIPEQFRHAIWKGILDHRQLHDFSSPPHLLRTPSGASTVSVGSSETRGERVIDAVRFTLRQTISFPPRDEWNDFNFDMDSRRNKQQRIKEEGE

>KAF6380715.1 tumor protein p63 [Myotis myotis]

MDFETSRCATLQYCPDPYIQRFVETPAHFSWKESYYRSTMSQSTQTSEFLSPEVFQHIWDFLEQPICSVQPIDLNFVDEPSENGATNKIEISMDCIRMQDSDLSDPMWPQYTNLGLLNSMDQQIQNGSSSTSPYNTDHAQNSVTAPSPYAQPSSTFDALSPSPAIPSNTDYPGPHSFDVSFQQSSTAKSATWTYSTELKKLYCQIAKTCPIQIKVMTPPPQGAVIRAMPVYKKAEHVTEVVKRCPNHELSREFNEGQIAPPSHLIRVEGNSHAQYVEDPITGRQSVLVPYEPPQVGTEFTTVLYNFMCNSSCVGGMNRRPILIIVTLETRDGQVLGRRCFEARICACPGRDRKADEDSIRKQQVSDSTKNGDGTKRPFRQNTHGIQMTSIKKRRSPDDELLYLPVRGRETYEMLLKIKESLELMQYLPQHTIETYRQQQQQQHQHLLQKQTSMQSQSSYGNSSPPLNKMNSMNKLPSVSQLINPQQRNALTPTTIPDGMGANIPMMGTHMPVAGDMNGLSPTQALPPPLSMPSTSHCTPPPPYPTDCSLVSFLARLGCSSCLDYFTTQGLTTIYQIEHYSMDDLASLKIPEQFRHAIWKGILDHRQLHDFSSPPHLLRTPSGTSTVSVGSSETRGERVIDAVRFTLRQTISFPPRDEWNDFNFDMDARRNKQQRIKEEGE

>XP_008837899.1 tumor protein 63 isoform X1 [Nannospalax galili]

MNFESSRCATLQYCPDPYIQRFVETPAHFSWKESYYRSTMSQSSQTSEFLSPEVFQHIWDFLEQPICSVQPIDLNFVDEPSENGTTNKIEISMDCIRMQDADLSDPMWPQYTNLGLLNSMDQQIQNGSSSTSPYNTDHAQNSVTAPSPYAQPSSTFDALSPSPAIPSNTDYPGPHSFDVSFQQSSTAKSATWTYSTELKKLYCQIAKTCPIQIKVMTPPPQGAVIRAMPVYKKAEHVTEVVKRCPNHELSREFNEGQIAPPSHLIRVEGNSHAQYVEDPITGRQSVLVPYEPPQVGTEFTTVLYNFMCNSSCVGGMNRRPILIIVTLETRDGQVLGRRCFEARICACPGRDRKADEDSIRKQQVSDSTKNGDGTKRPFRQNTHGIQMTSIKKRRSPDDELLYLPVRGRETYEMLLKIKESLELMQYLPQHTIETYRQQQQQQHQHLLQKQTPIQSQSSYGNSSPPLNKMNSMNKLPSVSQLINPQQRNALTPTTMPEGMGANIPMMSTHMPMAGDMNGLSPTQALPPPLSMPSTSHCTPPPPYPTDCSIVSFLARLGCSSCLDYFTTQGLTTIYQIEHYSMDDLASLKIPEQFRHAIWKGILDHRQLHDFSSPPHLLRTPSGASTVSVGSSETRGERVIDAVRFTLRQTISFPPRDEWNDFNFDMDSRRNKQQRIKEEGE

>XP_003792753.1 tumor protein 63 isoform X1 [Otolemur garnettii]

MNFETSRGATLQYCPDPYIQRFVETPAHFSWKESYYRSTMSQSTQTSEFLSPEVFQHIWDFLEQPICSVQPIDLNFVDEPSENGTTNKIEISMDCIRVQDSDLSDPMWPQYTNLGLLNSMDQQIQNGSSSTSPYNTDHAQNSVTAPSPYAQPSSTFDALSPSPAIPSNTDYPGPHSFDVSFQQSSTAKSATWTYSTELKKLYCQIAKTCPIQIKVMTPPPQGAVIRAMPVYKKAEHVTEVVKRCPNHELSREFNEGQIAPPSHLIRVEGNSHAQYVEDPITGRQSVLVPYEPPQVGTEFTTVLYNFMCNSSCVGGMNRRPILIIVTLETRDGQVLGRRCFEARICACPGRDRKADEDSIRKQQVSDSTKNGDGTKRPFRQNTHGIQMTSIKKRRSPDDELLYLPVRGRETYEMLLKIKESLELMQYLPQHTIETYRQQQQQQHQHLLQKQTSMQSQSSYGNSSPPLNKMNSMNKLPSVSQLINPQQRNALTPTTIPDGMGANIPMMGTHMPMAGDMNGLSPTQALPPPLSMPSTSHCTPPPPYPTDCSIVSFLARLGCSSCLDYFTTQGLTTIYQIEHYSMDDLASLKIPEQFRHAIWKGILDHRQLHDFSSPPHLLRTPSGASTVSVGSSETRGERVIDAVRFTLRQTISFPPRDEWNDFNFDMDARRNKQQRIKEEGE

>XP_041533931.1 tumor protein 63 isoform X1 [Microtus oregoni]

MNFETSRCSTLQYCPDPYFQRFVETPAHFSWKESYYRSTMSQSSQTSELFSPEVFQHIWDFLEQPICSVQPIDLNFVEEPSENGATNKIEISMDCIRMQDSDLTDPMWPQYTNLGLLNSMDQQIQNGSSSTSPYNTDHAQNSVTAPSPYAQPSSTFDALSPSPAIPSNTDYPGPHSFDVSFQQSSTAKSATWTYSTELKKLYCQIAKTCPIQIKVMTPPPQGAVIRAMPVYKKAEHVTEVVKRCPNHELSREFNEGQIAPPSHLIRVEGNSHAQYVEDPITGRQSVLVPYEPPQVGTEFTTVLYNFMCNSSCVGGMNRRPILIIVTLETRDGQVLGRRCFEARICACPGRDRKADEDSIRKQQVSDSAKNGDGTKRPFRQNTHGIQMTSIKKRRSPDDELLYLPVRGRETYEMLLKIKESLELMQYLPQHTIETYRQQQQQQHQHLLQKQTSMQSQSSYGNSSPPLSKMNSMNKLPSVSQLINPQQRNALTPTTMPEGMGANIPMMGTHMPMAGDMNGLSPTQALPPPLSMPSTSHCTPPPPYPTDCSIVSFLARLGCSSCLDYFTTQGLTTIYQIEHYSMDDLASLKIPEQFRHAIWKGILDHRQLHDFSSPPHLLRTPSGASTVSVGSSETRGERVIDAVRFTLRQTISFPPRDEWNDFNFDMDSRRNKQQRIKEEGE

>XP_027443488.1 tumor protein 63 isoform X3 [Zalophus californianus]

MNCETSRCATLQYCPDPYIQRFVETPAHFSWKESYYRSTMSQSTQTSEFLSPEVFQHIWDFLEQPICSVQPIDLNFVDEPSENGARNKIEISMDCIRMQDSDLSDPMWPQYTNLGLLNSMDQQIQNGSSSTSPYNTDHAQNSVTAPSPYAQPSSTFDALSPSPAIPSNTDYPGPHSFDVSFQQSSTAKSATWTYSTELKKLYCQIAKTCPIQIKVMTPPPQGAVIRAMPVYKKAEHVTEVVKRCPNHELSREFNEGQIAPPSHLIRVEGNSHAQYVEDPITGRQSVLVPYEPPQVGTEFTTVLYNFMCNSSCVGGMNRRPILIIVTLETRDGQVLGRRCFEARICACPGRDRKADEDSIRKQQVTDSAKNGDGTKRPFRQNTHGIQMTSIKKRRSPDDELLYLPVRGRETYEMLLKIKESLELMQYLPQHTIETYRQQQQQQHQHLLQKQTSMQSQSTYGNSSPPLNKMNSMNKLPSVSQLINPQQRNALTPTTIPDGMGANIPMMGTHMPMAGDMNGLSPTQALPPPLSMPSTSHCTPPPPYPTDCSLVSFLARLGCSSCLDYFTTQGLTTIYQIEHYSMDDLASLKIPEQFRHAIWKGILDHRQLHDFSSPPHLLRTPSGASTVSVGSSETRGERVIDAVRFTLRQTISFPPRDEWNDFNFDMDARRNKQQRIKEEGE

>XP_003991845.2 tumor protein 63 isoform X1 [Felis catus]

MLYNCSPLVDCQRELEEMNCETSRCATLQYCPDPYIQRFVETPAHFSWKESYYRSTMSQSTQTSEFLSPEVFQHIWDFLEQPICSVQPIDLNFVDEPSENGARNKIEISMDCIRMQDSDLSDPMWPQYTNLGLLNSMDQQIQNGSSSTSPYNTDHAQNSVTAPSPYAQPSSTFDALSPSPAIPSNTDYPGPHSFDVSFQQSSTAKSATWTYSTELKKLYCQIAKTCPIQIKVMTPPPQGAVIRAMPVYKKAEHVTEVVKRCPNHELSREFNEGQIAPPSHLIRVEGNSHAQYVEDPITGRQSVLVPYEPPQVGTEFTTVLYNFMCNSSCVGGMNRRPILIIVTLETRDGQVLGRRCFEARICACPGRDRKADEDSIRKQQVSDSAKNGDGTKRPFRQNTHGIQMTSIKKRRSPDDELLYLPVRGRETYEMLLKIKESLELMQYLPQHTIETYRQQQQQQHQHLLQKQTSMQTQSSYGNSSPPLNKMNSMNKLPSVSQLINPQQRNALTPTTIPDGMGANIPMMGTHMPMAGDMNGLSPTQALPPPLSMPSTSHCTPPPPYPTDCSLVSFLARLGCSSCLDYFTTQGLTTIYQIEHYSMDDLASLKIPEQFRHAIWKGILDHRQLHDFSSPPHLLRTPSGASTVSVGSSETRGERVIDAVRFTLRQTISFPPRDEWNDFNFDMDARRNKQQRIKEEGE

>XP_027807257.1 tumor protein 63 isoform X1 [Marmota flaviventris]

MNFETSRCATLQYRPDPYIQRFVETPAHFSWKESYYRSTMSQSTQTSEFLSPEVFQHIWDFLEQPICSVQPIDLNFVDEPSENGATNKIEISMDCIRMQDSDLSDPMWPQYTNLGLLNSMDQQIQNGSSSTSPYNTDHAQNSVTAPSPYAQPSSTFDALSPSPAIPSNTDYPGPHSFDVSFQQSSTAKSATWTYSTELKKLYCQIAKTCPIQIKVMTPPPQGAVIRAMPVYKKAEHVTEVVKRCPNHELSREFNEGQIAPPSHLIRVEGNSHAQYVEDPITGRQSVLVPYEPPQVGTEFTTVLYNFMCNSSCVGGMNRRPILIIVTLETRDGQVLGRRCFEARICACPGRDRKADEDSIRKQQVSDSTKNGDGTKRPFRQNTHGIQMTSIKKRRSPDDELLYLPVRGRETYEMLLKIKESLELMQYLPQHTIETYRQQQQQQHQHLLQKQTSMQSQTSYGNSSPPLNKMNSMNKLPSVSQLINPQQRNALTPTTIPDGMGANIPMMSTHMPMAGDMNGLSPTQALPPPLSMPSTSHCTPPPPYPTDCSIVSFLARLGCSSCLDYFTTQGLTTIYQIEHYSMDDLASLKIPEQFRHAIWKGILDHRQLHDFSSPPHLLRTPSGASTVSVGSSETRGERVIDAVRFTLRQTISFPPRDEWNDFNFDMDARRNKQQRIKEEGE

>XP_032193830.1 tumor protein 63 isoform X1 [Mustela erminea]

MCTKGEVSKLLCLIAFDPIAFSLLALLSLYTQVHVYMFSNCSPLVDYQRELKEMNCETSRCATLQYCPDPYIQRFVETPAHFSWKESYYRSTMSQSTQTNEFLSPEVFQHIWDFLEQPICSVQPIDLNFVDEPSENGTRNKIEISMDCIRMQDSDLSDPMWPQYTNLGLLNSMDQQIQNGSSSTSPYNTDHAQNSVTAPSPYAQPSSTFDALSPSPAIPSNTDYPGPHSFDVSFQQSSTAKSATWTYSTELKKLYCQIAKTCPIQIKVMTPPPQGAVIRAMPVYKKAEHVTEVVKRCPNHELSREFNEGQIAPPSHLIRVEGNSHAQYVEDPITGRQSVLVPYEPPQVGTEFTTVLYNFMCNSSCVGGMNRRPILIIVTLETRDGQVLGRRCFEARICACPGRDRKADEDSIRKQQVSDSAKNGDGTKRPFRQNTHGIQMTSIKKRRSPDDELLYLPVRGRETYEMLLKIKESLELMQYLPQHTIETYRQQQQQQHQHLLQKQTSMQSQSTYGNSSPPLNKMNSMNKLPSVSQLINPQQRNALTPTTIPDGMGANIPMMGTHMPMAGDMNGLSPTQALPPPLSMPSTSHCTPPPPYPTDCSLVSFLARLGCSSCLDYFTTQGLTTIYQIEHYSMDDLASLKIPEQFRHAIWKGILDHRQLHDFSSPPHLLRTPSGASTVSVGSSETRGERVIDAVRFTLRQTISFPPRDEWNDFNFDMDARRNKQQRIKEEGE

>XP_036987670.1 tumor protein 63 isoform X1 [Artibeus jamaicensis]

MNLETSRCATLQYCPDPYIQRFVETPAHFSWKESYYRSTMSQSTQTSEFLSPEVFQHIWDFLEQPICSVQPIDLNFVDEPSENGATNKIEISMDCIRMQDSDLSDPMWPQYTNLGLLNSMDQQIQNGSSSTSPYNTDHAQNSVTAPSPYAQPSSTFDALSPSPAIPSNTDYPGPHSFDVSFQQSSTAKSATWTYSTELKKLYCQIAKTCPIQIKVMTPPPQGAVIRAMPVYKKAEHVTEVVKRCPNHELSREFNEGQIAPPSHLIRVEGNSHAQYVEDPITGRQSVLVPYEPPQVGTEFTTVLYNFMCNSSCVGGMNRRPILIIVTLETRDGQVLGRRCFEARICACPGRDRKADEDSIRKQQVSDSTKNGDGTKRPFRQNTHGIQMTSIKKRRSPDDELLYLPVRGRETYEMLLKIKESLELMQYLPQHTIETYRQQQQQQHQHLLQKQTSMQSQTSYGNSSPPLNKMNSMNKLPSVSQLINPQQRNALTPTTIPDGMAANIPMMGTHMPMAGDMNGLSPTQALPPPLSMPSTSHCTPPPPYPTDCSLVSFLARLGCSSCLDYFTTQGLTTIYQIEHYSMDDLASLKIPEQFRHAIWKGILDHRQLHDFSSPPHLLRTPSGTSTVSVGSSETRGERVIDAVRFTLRQTISFPPRDEWNDFNFDMDARRNKQQRIKEEGE

>XP_037694249.1 tumor protein 63 isoform X1 [Choloepus didactylus]

MNFETSRWATPQYCPDPYIQRFVETPAHFSWKESYYRSSMSQSTQTSEFLSPEVFQHIWDFLEQPICSVQPIDLNFVDQPSENGATNKIEISMDCIRMQDSDLSDPMWPQYTNLGLLNSMDQQIQNGSSSTSPYNTDHAQNSVTAPSPYAQPSSTFDALSPSPAIPSNTDYPGPHSFDVSFQQSSTAKSATWTYSTELKKLYCQIAKTCPIQIKVMTPPPQGAVIRAMPVYKKAEHVTEVVKRCPNHELSREFNEGQIAPPSHLIRVEGNSHAQYVEDPITGRQSVLVPYEPPQVGTEFTTVLYNFMCNSSCVGGMNRRPILIIVTLETRDGQVLGRRCFEARICACPGRDRKADEDSIRKQQVSDSTKNGDGTKRPFRQNTHGIQMTSIKKRRSPDDELLYLPVRGRETYEMLLKIKESLELMQYLPQHTIETYRQQQQQQHQHLLQKQTSMQSQSSYGNSSPPLNKMNSMNKLPSVSQLINPQQRNALTPTTIPDGMGANIPMMGTHMPMAGDMNGLSPTQALPPPLSMPSTSHCTPPPPYPTDCSIVSFLARLGCSSCLDYFTTQGLTTIYQIEHYSMDDLASLKIPEQFRHAIWKGILDHRQLHDFSSPPHLLRTPSGASTVSVGSSETRGERVIDAVRFTLRQTISFPPRDEWNDFNFDMDARRNKQQRIKEEGE

>XP_026352343.1 tumor protein 63 isoform X1 [Ursus arctos horribilis]

MNCETSRCATLQYCPDPYIQRFVETPAHFSWKESYYRSTMSQSTQTSEFLSPEVFQHIWDFLEQPICSVQPIDLNFVDEPSENGARNKIEISMDCIRMQDSDLSDPMWPQYTNLGLLNSMDQQIQNGSSSTSPYNTDHAQNSVTAPSPYAQPSSTFDALSPSPAIPSNTDYPGPHSFDVSFQQSSTAKSATWTYSTELKKLYCQIAKTCPIQIKVMTPPPQGAVIRAMPVYKKAEHVTEVVKRCPNHELSREFNEGQIAPPSHLIRVEGNSHAQYVEDPITGRQSVLVPYEPPQVGTEFTTVLYNFMCNSSCVGGMNRRPILIIVTLETRDGQVLGRRCFEARICACPGRDRKADEDSIRKQQVSDSAKNGDGTKRPFRQNTHGIQMTSIKKRRSPDDELLYLPVRGRETYEMLLKIKESLELMQYLPQHTIETYRQQQQQQHQHLLQKQTSMQSQSTYGNSSPPLNKMNSMNKLPSVSQLINPQQRNALTPTAIPDGMGANIPMMGTHMPMAGDMNGLSPTQALPPPLSMPSTSHCTPPPPYPTDCSLVSFLARLGCSSCLDYFTTQGLTTIYQIEHYSMDDLASLKIPEQFRHAIWKGILDHRQLHDFSSPPHLLRTPSGASTVSVGSSETRGERVIDAVRFTLRQTISFPPRDEWNDFNFDMDARRNKQQRIKEEGE

>XP_034517071.1 tumor protein 63 isoform X1 [Ailuropoda melanoleuca]

MFYNCSPLVDCQRELKEMNCETSRCATLQYCPDPYIQRFVETPAHFSWKESYYRSTMSQSTQTSEFLSPEVFQHIWDFLEQPICSVQPIDLNFVDEPSENGARNKIEISMDCIRMQDSDLSDPMWPQYTNLGLLNSMDQQIQNGSSSTSPYNTDHAQNSVTAPSPYAQPSSTFDALSPSPAIPSNTDYPGPHSFDVSFQQSSTAKSATWTYSTELKKLYCQIAKTCPIQIKVMTPPPQGAVIRAMPVYKKAEHVTEVVKRCPNHELSREFNEGQIAPPSHLIRVEGNSHAQYVEDPITGRQSVLVPYEPPQVGTEFTTVLYNFMCNSSCVGGMNRRPILIIVTLETRDGQVLGRRCFEARICACPGRDRKADEDSIRKQQVSDSAKNGDGTKRPFRQNTHGIQMTSIKKRRSPDDELLYLPVRGRETYEMLLKIKESLELMQYLPQHTIETYRQQQQQQHQHLLQKQTSMQSQSTYGNSSPPLNKMNSMNKLPSVSQLINPQQRNALTPTTIPDGMGANIPMMGTHMPMAGDMNGLSPTQALPPPLSMPSTSHCTPPPPYPTDCSLVSFLARLGCSSCLDYFTTQGLTTIYQIEHYSMDDLASLKIPEQFRHAIWKGILDHRQLHDFSSPPHLLRTPSGASTVSVGSSETRGERVIDAVRFTLRQTISFPPRDEWNDFNFDMDARRNKQQRIKEEGE

>XP_006201027.1 tumor protein 63 isoform X1 [Vicugna pacos]

MNFETSRCATLQYCPDPYIQRFVETPAHFSWKESYYRSTMSQSTQTSEFLSPEVFQHIWDFLEQPICSVQPIDLNFVDEPSENGTTNKIEISMDCIRMQDSDLTDPMWPQYTNLGLLNSMDQQIQNGSSSTSPYNTEHAQNSVTAPSPYAQPSSTFDALSPSPAIPSNTDYPGPHSFDVSFQQSSTAKSATWTYSTELKKLYCQIAKTCPIQIKVMTPPPQGAVIRAMPVYKKAEHVTEVVKRCPNHELSREFNEGQIAPPSHLIRVEGNSHAQYVEDPITGRQSVLVPYEPPQVGTEFTTVLYNFMCNSSCVGGMNRRPILIIVTLETRDGQVLGRRCFEARICACPGRDRKADEDSIRKQQVSDSTKNGDGTKRPFRQNTHGIQMTSIKKRRSPDDELLYLPVRGRETYEMLLKIKESLELMQYLPQHTIETYRQQQQQQHQHLLQKQTSMQSQSSYGNSSPPLNKMNSMNKLPSVSQLINPQQRNALTPTTIPDGMGANIPMMSTHMPMAGDMNGLSPTQALPPPLSMPSTSHCTPPPPYPTDCSLVSFLARLGCSSCLDYFTTQGLTTIYQIEHYSMDDLASLKIPEQFRHAIWKGILDHRQLHDFSSPPHLLRTPSGASTVSVGSSETRGERVIDAVRFTLRQTISFPPRDEWNDFNFDMDARRNKQQRIKEEGE

>KAF6122297.1 tumor protein p63 [Phyllostomus discolor]

MNLETSRCATLQYCPDPYIQRFVETPAHFSWKESYYRSTMSQSTQTSEFLSPEVFQHIWDFLEQPICSVQPIDLNFVDEPSENGATNKIEISMDCIRMQDSDLSDPMWPQYTNLGLLNSMDQQIQNGSSSTSPYNTDHAQNSVTAPSPYAQPSSTFDALSPSPAIPSNTDYPGPHSFDVSFQQSSTAKSATWTYSTELKKLYCQIAKTCPIQIKVMTPPPQGAVIRAMPVYKKAEHVTEVVKRCPNHELSREFNEGQIAPPSHLIRVEGNSHAQYVEDPITGRQSVLVPYEPPQVGTEFTTVLYNFMCNSSCVGGMNRRPILIIVTLETRDGQVLGRRCFEARICACPGRDRKADEDSIRKQQVSDSTKNGDGTKRPFRQNTHGIQMTSIKKRRSPDDELLYLPVRGRETYEMLLKIKESLELMQYLPQHTIETYRQQQQQQHQHLLQKQTSMQSQTSYGNSSPPLNKMNSMNKLPSVSQLINPQQRNALTPTTIPDSMGANIPMMGTHMPMAGDMNGLSPTQALPPPLSMPSTSHCTPPPPYPTDCSLVSFLARLGCSSCLDYFTTQGLTTIYQIEHYSMDDLASLKIPEQFRHAIWKGILDHRQLHDFSSPPHLLRTPSGTSTVSVGSSETRGERVIDAVRFTLRQTISFPPRDEWNDFNFDMDARRNKQQRIKEEGE

>XP_006188922.1 tumor protein 63 isoform X1 [Camelus ferus]

MNFETSRCATLRYCPDPYIQRFVETPAHFSWKESYYRSTMSQSTQTSEFLSPEVFQHIWDFLEQPICSVQPIDLNFVDEPSENGTTNKIEISMDCIRMQDSDLTDPMWPQYTNLGLLNSMDQQIQNGSSSTSPYNTDHAQNSVTAPSPYAQPSSTFDALSPSPAIPSNTDYPGPHSFDVSFQQSSTAKSATWTYSTELKKLYCQIAKTCPIQIKVMTPPPQGAVIRAMPVYKKAEHVTEVVKRCPNHELSREFNEGQIAPPSHLIRVEGNSHAQYVEDPITGRQSVLVPYEPPQVGTEFTTVLYNFMCNSSCVGGMNRRPILIIVTLETRDGQVLGRRCFEARICACPGRDRKADEDSIRKQQVSDSTKNGDGTKRPFRQNTHGIQMTSIKKRRSPDDELLYLPVRGRETYEMLLKIKESLELMQYLPQHTIETYRQQQQQQHQHLLQKQTSMQSQSSYGNSSPPLNKMNSMNKLPSVSQLINPQQRNALTPTTIPDGMGANIPMMSTHMPMAGDMNGLSPTQALPPPLSMPSTSHCTPPPPYPTDCSLVSFLARLGCSSCLDYFTTQGLTTIYQIEHYSMDDLASLKIPEQFRHAIWKGILDHRQLHDFSSPPHLLRTPSGASTVSVGSSETRGERVIDAVRFTLRQTISFPPRDEWNDFNFDMDARRNKQQRIKEEGE

>XP_008703384.2 tumor protein 63 isoform X1 [Ursus maritimus]

MFYNCSPLVDCQRELKEMNCETSRCATLQYCPDPYIQRFVETPAHFSWKESYYRSTMSQSTQTSEFLSPEVFQHIWDFLEQPICSVQPIDLNFVDEPSENGARNKIEISMDCIRMQDSDLSDPMWPQYTNLGLLNSMDQQIQNGSSSTSPYNTDHAQNSVTAPSPYAQPSSTFDALSPSPAIPSNTDYPGPHSFDVSFQQSSTAKSATWTYSTELKKLYCQIAKTCPIQIKVMTPPPQGAVIRAMPVYKKAEHVTEVVKRCPNHELSREFNEGQIAPPSHLIRVEGNSHAQYVEDPITGRQSVLVPYEPPQVGTEFTTVLYNFMCNSSCVGGMNRRPILIIVTLETRDGQVLGRRCFEARICACPGRDRKADEDSIRKQQVSDSAKNGDGTKRPFRQNTHGIQMTSIKKRRSPDDELLYLPVRGRETYEMLLKIKESLELMQYLPQHTIETYRQQQQQQHQHLLQKQTSMQSQSTYGNSSPPLNKMNSMNKLPSVSQLINPQQRNALTPTAIPDGMGANIPMMGTHMPMAGDMNGLSPTQALPPPLSMPSTSHCTPPPPYPTDCSLVSFLARLGCSSCLDYFTTQGLTTIYQIEHYSMDDLASLKIPEQFRHAIWKGILDHRQLHDFSSPPHLLRTPSGASTVSVGSSETRGERVIDAVRFTLRQTISFPPRDEWNDFNFDMDARRNKQQRIKEEGE

>XP_004382289.1 tumor protein 63 isoform X1 [Trichechus manatus latirostris]

MNFETSRCSTLQYCPDPYIQRFVETPAHFSWKESYYRSTMSQSTQTSEFLSPEVFQHIWDFLEQPICSVQPIDLNFVDEPSENGATNKIEISMDCIRMQDSELTDPMWPQYTNLGLLNSMDQQIQNGSSSTSPYNTDHAQNSVTAPSPYAQPSSTFDALSPSPAIPSNTDYPGPHSFDVSFQQSSTAKSATWTYSTELKKLYCQIAKTCPIQIKVMTPPPQGAVIRAMPVYKKAEHVTEVVKRCPNHELSREFNEGQIAPPSHLIRVEGNSHAQYVEDPITGRQSVLVPYEPPQVGTEFTTVLYNFMCNSSCVGGMNRRPILIIVTLETRDGQVLGRRCFEARICACPGRDRKADEDSIRKQQVSDSTKNGDGTKRPFRQNTHGIQMTSIKKRRSPDDELLYLPVRGRETYEMLLKIKESLELMQYLPQHTIETYRQQQQQQHQHLLQKQTSMQSQSSYGNSSPPLNKMNSMNKLPSVSQLINPQQRNTLTPTTIPDGMGANIPMMGTHMPMAGDMNGLSPTQALPPPLAMPSTSHCTPPPPYPTDCSIVSFLARLGCSSCLDYFTTQGLTTIYQIEHYSMDDLASLKIPEQFRHAIWKGILDHRQLHDFSSPPHLLRTPSSASTVSVGSSETRGERVIDAVRFTLRQTISFPPRDEWNDFNFDMDARRNKQQRIKEEGE

>XP_022359420.1 tumor protein 63 isoform X1 [Enhydra lutris kenyoni]

MNCETSRCATLQYCPDPYIQRFVETPAHFSWKESYYRSTMSQSTQTNEFLSPEVFQHIWDFLEQPICSVQPIDLNFVDEPSENGTRNKIEISMDCIRMQDSDLSDPMWPQYTNLGLLNSMDQQIQNGSSSTSPYNTDHAQNSVTAPSPYAQPSSTFDALSPSPAIPSNTDYPGPHSFDVSFQQSSTAKSATWTYSTELKKLYCQIAKTCPIQIKVMTPPPQGAVIRAMPVYKKAEHVTEVVKRCPNHELSREFNEGQIAPPSHLIRVEGNSHAQYVEDPITGRQSVLVPYEPPQVGTEFTTVLYNFMCNSSCVGGMNRRPILIIVTLETRDGQVLGRRCFEARICACPGRDRKADEDSIRKQQVSDSAKNGDGTKRPFRQNTHGIQMTSIKKRRSPDDELLYLPVRGRETYEMLLKIKESLELMQYLPQHTIETYRQQQQQQHQHLLQKQTSMQSQSTYGNSSPPLNKMNSMNKLPSVSQLINPQQRNALTPTTIPDGMGANIPMMGTHMPMAGDMNGLSPTQALPPPLSMPSTSHCTPPPPYPTDCSLVSFLARLGCSSCLDYFTTQGLTTIYQIEHYSMDDLASLKIPEQFRHAIWKGILDHRQLHDFSSPPHLLRTPSGASTVSVGSSETRGERVIDAVRFTLRQTISFPPRDEWNDFNFDMDARRNKQQRIKEEGE

>XP_002814454.1 tumor protein 63 isoform X1 [Pongo abelii]

MNFETSRCATLQYCPDPYIQRFVETPAHFSWKESYYRSTMSQSTQTNEFLSPEVFQHIWDFLEQPICSVQPIDLNFVDEPSEGGATNKIEISMDCIRMQDSDLSDPMWPQYTNLGLLNSMDQQIQNGSSSTSPYNTDHAQNSVTAPSPYAQPSSTFDALSPSPAIPSNTDYPGPHSFDVSFQQSSTAKSATWTYSTELKKLYCQIAKTCPIQIKVMTPPPQGAVIRAMPVYKKAEHVTEVVKRCPNHELSREFNEGQIAPPSHLIRVEGNSHAQYVEDPITGRQSVLVPYEPPQVGTEFTTVLYNFMCNSSCVGGMNRRPILIIVTLETRDGQVLGRRCFEARICACPGRDRKADEDSIRKQQVSDSTKNGDGTKRPFRQNTHGIQMTSIKKRRSPDDELLYLPVRGRETYEMLLKIKESLELMQYLPQHTIETYRQQQQQQHQHLLQKQTSIQSQSSYGNSSPPLNKMNSMNKLPSVSQLINPQQRNALTPTTIPDGMGANIPMMGTHMPMAGDMNGLSPTQALPPPLSMPSTSHCTPPPPYPTDCSIVSFLARLGCSSCLDYFTTQGLTTIYQIEHYSMDDLASLKIPEQFRHAIWKGILDHRQLHEFSSPSHLLRTPSSASTVSVGSSETRGERVIDAVRFTLRQTISFPPRDEWNDFNFDMDARRNKQQRIKEEGE

>XP_036894353.1 tumor protein 63 isoform X1 [Sturnira hondurensis]

MNLETSRCATLQYCPDPYIQRFVETPAHFSWKESYYRSTMSQSTQTSEFLSPEVFQHIWDFLEQPICSVQPIDLNFVDEPSENGATNKIEISMDCIRMQDSDLSDPMWPQYTNLGLLNSMDQQIQNGSSSTSPYNTDHAQNSVTAPSPYAQPSSTFDALSPSPAIPSNTDYPGPHSFDVSFQQSSTAKSATWTYSTELKKLYCQIAKTCPIQIKVMTPPPQGAVIRAMPVYKKAEHVTEVVKRCPNHELSREFNEGQIAPPSHLIRVEGNSHAQYVEDPITGRQSVLVPYEPPQVGTEFTTVLYNFMCNSSCVGGMNRRPILIIVTLETRDGQVLGRRCFEARICACPGRDRKADEDSIRKQQVSDSTKNGDGTKRPFRQNTHGIQMTSIKKRRSPDDELLYLPVRGRETYEMLLKIKESLELMQYLPQHTIETYRQQQQQQHQHLLQKQTSMQSQTSYGNSSPPLNKMNSMNKLPSVSQLINPQQRNALTPTTIPDGMAANIPMMGTHMPMAGDMNGLSPTQALPPPLSMPSTSHCTPPPPYPTDCSLVSFLARLGCSSCLDYFTTQGLTTIYQIEHYSMDDLASLKIPEQFRHAIWKGILDHRQLHDFSSPPHLLRTPSGTSTVSVGSSEARGERVIDAVRFTLRQTISFPPRDEWNDFNFDMDARRNKQQRIKEEGE

>XP_001092093.1 tumor protein 63 isoform X1 [Macaca mulatta]

MNFETSRCATLQYCPDPYIQRFVETPAHFSWKESYYRSTMSQSTQTNEFLSPEVFQHIWDFLEQPICSVQPIDLNFVDEPSEDGATNKIEISMDCIRMQDSDLSDPMWPQYTNLGLLNSMDQQIQNGSSSTSPYNTDHAQNSVTAPSPYAQPSSTFDALSPSPAIPSNTDYPGPHSFDVSFQQSSTAKSATWTYSTELKKLYCQIAKTCPIQIKVMTPPPQGAVIRAMPVYKKAEHVTEVVKRCPNHELSREFNEGQIAPPSHLIRVEGNSHAQYVEDPITGRQSVLVPYEPPQVGTEFTTVLYNFMCNSSCVGGMNRRPILIIVTLETRDGQVLGRRCFEARICACPGRDRKADEDSIRKQQVSDSTKNGDGTKRPFRQNTHGIQMTSIKKRRSPDDELLYLPVRGRETYEMLLKIKESLELMQYLPQHTIETYRQQQQQQHQHLLQKQTSIQSQSSYGNSSPPLNKMNSMNKLPSVSQLINPQQRNALTPTTIPDGMGANIPMMGTHMPMAGDMNGLSPTQALPPPLSMPSTSHCTPPPPYPTDCSIVSFLARLGCSSCLDYFTTQGLTTIYQIEHYSMDDLASLKIPEQFRHAIWKGILDHRQLHEFSSPSHLLRTPSSASTVSVGSSETRGERVIDAVRFTLRQTISFPPRDEWNDFNFDMDARRNKQQRIKEEGE

>XP_035920522.1 tumor protein 63 isoform X1 [Halichoerus grypus]

MNCETSRCATLQYCPDPYIQRFVETPAHFSWKESYYRSTMSQSTQTSEFLSPEVFQHIWDFLEQPICSVQPIDLNFVDEPSENGARNKIEISMDCIRMQDSDLSDPMWPQYTNLGLLNSMDQQIQNGSSSTSPYNTDHAQNSVTAPSPYAQPSSTFDALSPSPAIPSNTDYPGPHSFDVSFQQSSTAKSATWTQYSTELKKLYCQIAKTCPIQIKVMTPPPQGAVIRAMPVYKKAEHVTEVVKRCPNHELSREFNEGQIAPPSHLIRVEGNSHAQYVEDPITGRQSVLVPYEPPQVGTEFTTVLYNFMCNSSCVGGMNRRPILIIVTLETRDGQVLGRRCFEARICACPGRDRKADEDSIRKQQVTDSAKNGDGTKRPFRQNTHGIQMTSIKKRRSPDDELLYLPVRGRETYEMLLKIKESLELMQYLPQHTIETYRQQQQQQHQHLLQKQTSMQSQSTYGNSSPPLNKMNSMNKLPSVSQLINPQQRNALTPTTIPDGMGANIPMMGTHMPMAGDMNGLSPTQALPPPLSMPSTSHCTPPPPYPTDCSLVSFLARLGCSSCLDYFTTQGLTTIYQIEHYSMDDLASLKIPEQFRHAIWKGILDHRQLHDFSSPPHLLRTPSGASTVSVGSSETRGERVIDAVRFTLRQTISFPPRDEWNDFNFDMDARRNKQQRIKEEGE

>XP_003477134.1 tumor protein 63 isoform X1 [Cavia porcellus]

MNFETPRCATLQYCPDPYIQRFVETPGHFSWKESYYRSTMSQSTQTSEFLSPEVFQHIWDFLEQPICSVQPIDLNFMDESSENGATNKIEISMDCIRMQDSDLRDPMWPQYTNLGLLNSMDQQIQNGSSSTSPYNTDHAQNSVTAPSPYAQPSSTFDALSPSPAIPSNTDYPGPHSFDVSFQQSSTAKSATWTYSTELKKLYCQIAKTCPIQIKVMTPPPQGAVIRAMPVYKKAEHVTEVVKRCPNHELSREFNEGQIAPPSHLIRVEGNSHAQYVEDPITGRQSVLVPYEPPQVGTEFTTVLYNFMCNSSCVGGMNRRPILIIVTLETRDGQVLGRRCFEARICACPGRDRKADEDSIRKQQVSDSTKNGDGTKRPFRQNTHGIQMTSIKKRRSPDDELLYLPVRGRETYEMLLKIKESLELMQYLPQHTIETYRQQQQQQHQHLLQKQTSMQSQSSYGNSSPPLNKMNSMNKLPSVSQLINPQQRNALTPTTIPDGMGANIPMMGTHMPMAGDMNGLSPTQTLPPPLSMPSTSHCTPPPPYPTDCSIVSFLARLGCSSCLDYFTTQGLTTIYQIEHYSMDDLASLKIPEQFRHAIWKGILDHRQLHDFSSPPHLLRTPSGASTVSVGSSETRGERVIDAVRFTLRQTISFPPRDEWNDFNFDMDARRNKQQRIKEEGE

>XP_004640955.1 tumor protein 63 isoform X1 [Octodon degus]

MNFETSRSATLQYCPDPYIQRFVETPAHFSWKESYYRSTMSQSTQTSEFLSPEVFQHIWDFLEQPLCSVQPIDLNFVDESSENGATNKIEISMDCIRMQDSDLRDPMWPQYTNLGLLNSMDQQIQNGSSSTSPYNTDHAQNSVTAPSPYAQPSSTFDALSPSPAIPSNTDYPGPHSFDVSFQQSSTAKSATWTYSTELKKLYCQIAKTCPIQIKVMTPPPQGAVIRAMPVYKKAEHVTEVVKRCPNHELSREFNEGQIAPPSHLIRVEGNSHAQYVEDPITGRQSVLVPYEPPQVGTEFTTVLYNFMCNSSCVGGMNRRPILIIVTLETRDGQVLGRRCFEARICACPGRDRKADEDSIRKQQVSDSTKNGDGTKRPFRQNTHGIQMTSIKKRRSPDDELLYLPVRGRETYEMLLKIKESLELMQYLPQHTIETYRQQQQQQHQHLLQKQTSMQSQTSYGNSSPPLNKMNSMNKLPSVSQLINPQQRNALTPTTIPDGMGSNIPMMGTHMPMAGDMNGLSPTQALPPPLSMPSTSHCTPPPPYPTDCSIVSFLARLGCSSCLDYFTTQGLTTIYQIEHYSMDDLASLKIPEQFRHAIWKGILDHRQLHDFSSPSHLLRTPSGASTVSVGSSETRGERVIDAVRFTLRQTISFPPRDEWNDFNFDMDARRNKQQRIKEEGE

>KAF6383992.1 tumor protein p63 [Pipistrellus kuhlii]

MDFETSRCATLQYCPDPYIQRFVETPAHFSWKESYYRSTMSQSTQTSEFLSPEVFQHIWDFLEQPICSVQPIDLNFVDEPSENGATNKIEISMDCIRMQDSDLSDPMWPQYTNLGLLNSMDQQIQNGSSSTSPYNTDHAQNSVTAPSPYAQPSSTFDALSPSPAIPSNTDYPGPHSFDVSFQQSSTAKSATWTYSTELKKLYCQIAKTCPIQIKVMTPPPQGAVIRAMPVYKKAEHVTEVVKRCPNHELSREFNEGQIAPPSHLIRVEGNSHAQYVEDPITGRQSVLVPYEPPQVGTEFTTVLYNFMCNSSCVGGMNRRPILIIVTLETRDGQVLGRRCFEARICACPGRDRKADEDSIRKQQVSDSTKNGDGTKRPFRQNTHGIQMTSIKKRRSPDDELLYLPVRGRETYEMLLKIKESLELMQYLPQHTIETYRQQQQQQHQHLLQKQTSMQSQSSYGNSSPPLNKMNSMNKLPSVSQLINPQQRNALTPTTIPDGMGANLPMMGTHMPVAGDMNGLSPTQTLPPPLSMPSTSHCTPPPPYPTDCSLVSFLARLGCSSCLDYFTTQGLTTIYQIEHYSMDDLASLKIPEQFRHAIWKGIQDHRQLHDFSSPPHFLRTPSGTSTVSVGSSETRGERVIDAVRFTLRQTISFPPRDEWNDFNFDMDARRNKQQRIKEEGE

>XP_025281126.1 tumor protein 63 isoform X1 [Canis lupus dingo]

MNCGTSRCATLQYCPDPYIQRFVETPAHFSWKESYYRSTMSQSTQTNEFLSPEVFQHIWDFLEQPICSVQPIDLNFADEPSENGARNKIEISMDCIRMQDSDLSDPMWPQYTNLGLLNSMDQQIQNGSSSTSPYNTDHAQNSVTAPSPYAQPSSTFDALSPSPAIPSNTDYPGPHSFDVSFQQSSTAKSATWTYSTELKKLYCQIAKTCPIQIKVMTPPPQGAVIRAMPVYKKAEHVTEVVKRCPNHELSREFNEGQIAPPSHLIRVEGNSHAQYVEDPITGRQSVLVPYEPPQVGTEFTTVLYNFMCNSSCVGGMNRRPILIIVTLETRDGQVLGRRCFEARICACPGRDRKADEDSIRKQQVSDSAKNGDGTKRPFRQNTHGIQMTSIKKRRSPDDELLYLPVRGRETYEMLLKIKESLELMQYLPQHTIETYRQQQQQQHQHLLQKQTSMQSQSTYGNSSPPLNKMNSMNKLPSVSQLINPQQRNALTPTTIPDGMGANIPMMGTHMPMAGDMNGLSPTQALPPPLSMPSTSHCTPPPPYPTDCSLVSFLARLGCSSCLDYFTTQGLTTIYQIEHYSMDDLASLKIPEQFRHAIWKGILDHRQLHDFSSPPHLLRTPSGASTVSVGSSETRGERVIDAVRFTLRQTISFPPRDEWNDFNFDMDARRNKQQRIKEEGE

>XP_034370959.1 tumor protein 63 isoform X2 [Arvicanthis niloticus]

MNFETSRCATLQYCPDPYIQRFIETPAHFSWKESYYRSAMSQSTQPSEFLSPEVFQHIWDFLEQPICSVQPIDLNFVDEPSENGATNKIEISMDCIRMQDSDLSDPMWPQYTNLGLLNSMDQQIQNGSSSTSPYNTDHAQNSVTAPSPYAQPSSTFDALSPSPAIPSNTDYPGPHSFDVSFQQSSTAKSATWTYSTELKKLYCQIAKTCPIQIKVMTPPPQGAVIRAMPVYKKAEHVTEVVKRCPNHELSREFNEGQIAPPSHLIRVEGNSHAQYVEDPITGRQSVLVPYEPPQVGTEFTTVLYNFMCNSSCVGGMNRRPILIIVTLETRDGQVLGRRCFEARICACPGRDRKADEDSIRKQQVSDNAKNGDAFRQNTHGIQMTSIKKRRSPDDELLYLPVRGRETYEMLLKIKESLELMQYLPQHTIETYRQQQQQQHQHLLQKQTSMQSQSSYGTSSPPLNKMNSMNKLPSVSQLINPQQRNALTPTTMPEGMGANIPMMGTHMPMAGDMNGLSPTQALPPPLSMPSTSHCTPPPPYPTDCSIVSFLARLGCSSCLDYFTTQGLTTIYQIEHYSMDDLASLKIPEQFRHAIWKGILDHRQLHDFSSPPHLLRTPSGASTVSVGSSETRGERVIDAVRFTLRQTISFPPRDEWNDFNFDMDSRRNKQQRIKEEGE

>XP_032027787.1 tumor protein 63 isoform X1 [Hylobates moloch]

MNFETSRCATLQYCPDPYIQRFVETPAHFSWKESYYRSTMSQSTQTNEFLSPEVFQHIWDFLEQPICSVQPIDLNFVDEPSEDGATNKIEISMDCIRMQDSDLSDPMWPQYTNLGLLNSMDQQIQNGSSSTSPYNTDHAQNSVTAPSPYAQPSSTFDALSPSPAIPSNTDYPGPHSFDVSFQQSSTAKSATWTYSTELKKLYCQIAKTCPIQIKVMTPPPQGAVIRAMPVYKKAEHVTEVVKRCPNHELSREFNEGQIAPPSHLIRVEGNSHAQYVEDPITGRQSVLVPYEPPQVGTEFTTVLYNFMCNSSCVGGMNRRPILIIVTLETRDGQVLGRRCFEARICACPGRDRKADEDSIRKQQVSDSTKNGDGTKRPFRQNTHGIQMTSIKKRRSPDDELLYLPVRGRETYEMLLKIKESLELMQYLPQHTIETYRQQQQQQHQHLLQKQTSIQSQSSYGNSSPPLNKMNSMNKLPSVSQLINPQQRNALTPTTIPDGMGANIPMMGTHMPMAGDMNGLSPTQALAPPLSMPSTSHCTPPPPYPTDCSIVSFLARLGCSSCLDYFTTQGLTTIYQIEHYSMDDLASLKIPEQFRHAIWKGILDHRQLHEFSSPSHLLRTPSSASTVSVGSSETRGERVIDAVRFTLRQTISFPPRDEWNDFNFDMDARRNKQQRIKEEGE

>XP_021527046.1 tumor protein 63 isoform X1 [Aotus nancymaae]

MNFETSRCATLQYCPDPYIQRFVETPAHFSWKESYYRSTMSQSTQTNEFLSPEVFQHIWDFLEQPICSVQPIDLNFVDEPSEDGATNKIEISMDCIRMQDSDLSDPMWPQYTNLGLLNSMDQQIQNGSSSTSPYNTDHAQNSVTAPSPYAQPSSTFDALSPSPAIPSNTDYPGPHSFDVSFQQSSTAKSATWTYSTELKKLYCQIAKTCPIQIKVMTPPPQGAVIRAMPVYKKAEHVTEVVKRCPNHELSREFNEGQIAPPSHLIRVEGNSHAQYVEDPITGRQSVLVPYEPPQVGTEFTTVLYNFMCNSSCVGGMNRRPILIIVTLETRDGQVLGRRCFEARICACPGRDRKADEDSIRKQQVSDSTKNGDGTKRPFRQNTHGIQMTSIKKRRSPDDELLYLPVRGRETYEMLLKIKESLELMQYLPQHTIETYRQQQQQQHQHLLQKQTSIQSQSSYGNNSPPLNKMNSMNKLPSVSQLINPQQRNALTPTTIPDGMGANIPMMGTHMPMTGDMNGLSPTQALPPPLSMPSTSHCTPPPPYPTDCSIVSFLARLGCSSCLDYFTTQGLTTIYQIEHYSMDDLASLKIPEQFRHAIWKGILDHRQLHEFSSPSHLLRTPSSASTVSVGSSETRGERVIDAVRFTLRQTISFPPRDEWNDFNFDMDARRNKQQRIKEEGE

>XP_017405078.1 tumor protein 63 isoform X1 [Cebus imitator]

MNFETSRCATLQYCPDPYIQRFVETPAHFSWKESYYRSTMSQSTQTNEFFSPEVFQHIWDFLEQPICSVQPIDLNFVDEPSEDGATNKIEISMDCIRMQDSDLSDPMWPQYTNLGLLNSMDQQIQNGSSSTSPYNTDHAQNSVTAPSPYAQPSSTFDALSPSPAIPSNTDYPGPHSFDVSFQQSSTAKSATWTYSTELKKLYCQIAKTCPIQIKVMTPPPQGAVIRAMPVYKKAEHVTEVVKRCPNHELSREFNEGQIAPPSHLIRVEGNSHAQYVEDPITGRQSVLVPYEPPQVGTEFTTVLYNFMCNSSCVGGMNRRPILIIVTLETRDGQVLGRRCFEARICACPGRDRKADEDSIRKQQVSDSTKNGDGTKRPFRQNTHGIQMTSIKKRRSPDDELLYLPVRGRETYEMLLKIKESLELMQYLPQHTIETYRQQQQQQHQHLLQKQTSIQSQSSYGNSSPPLNKMNSMNKLPSVSQLINPQQRNALTPTTIPDGMGANIPMMGTHMPMTGDMNGLSPTQALPPPLSMPSTSHCTPPPPYPTDCSIVSFLARLGCSSCLDYFTTQGLTTIYQIEHYSMDDLASLKIPEQFRHAIWKGILDHRQLHEFSSPSHLLRTPSSASTVSVGSSETRGERVIDAVRFTLRQTISFPPRDEWNDFNFDMDARRNKQQRIKEEGE

>XP_011721436.1 tumor protein 63 isoform X1 [Macaca nemestrina]

MNFETSRCATLQYCPDPYIQRFVETPAHFSWKESYYRSTMSQSTQTNEFLSPEVFQHIWDFLEQPICSVQPIDLNFVDEPSEDGATNKIEISMDCIRMQDSDLSDPMWPQYTNLGLLNSMDQQIQNGSSSTSPYNTDHAQNSVTAPSPYAQPSSTFDALSPSPAIPSNTDYPGPHSFDVSFQQSSTAKSATWTQYSTELKKLYCQIAKTCPIQIKVMTPPPQGAVIRAMPVYKKAEHVTEVVKRCPNHELSREFNEGQIAPPSHLIRVEGNSHAQYVEDPITGRQSVLVPYEPPQVGTEFTTVLYNFMCNSSCVGGMNRRPILIIVTLETRDGQVLGRRCFEARICACPGRDRKADEDSIRKQQVSDSTKNGDGTKRPFRQNTHGIQMTSIKKRRSPDDELLYLPVRGRETYEMLLKIKESLELMQYLPQHTIETYRQQQQQQHQHLLQKQTSIQSQSSYGNSSPPLNKMNSMNKLPSVSQLINPQQRNALTPTTIPDGMGANIPMMGTHMPMAGDMNGLSPTQALPPPLSMPSTSHCTPPPPYPTDCSIVSFLARLGCSSCLDYFTTQGLTTIYQIEHYSMDDLASLKIPEQFRHAIWKGILDHRQLHEFSSPSHLLRTPSSASTVSVGSSETRGERVIDAVRFTLRQTISFPPRDEWNDFNFDMDARRNKQQRIKEEGE

>XP_028348042.1 tumor protein 63 isoform X6 [Physeter catodon]

MNFETSRCATLQYCPDPYIQRFVETPAHFSWKESYYRSTMSQSTQTNEFLSPEVFQHIWDFLEQPICSVQPIDLNFVDEPSENGATNKIEISMDCIRMQDSDLSDPMWPQYTNLGLLNSMDQQIQNGSSSTSPYNTDHAQNSVTAPSPYAQPSSTFDALSPSPAIPSNTDYPGPHSFDVSFQQSSTAKSATWTYSTELKKLYCQIAKTCPIQIKVMTPPPQGAVIRAMPVYKKAEHVTEVVKRCPNHELSREFNEGQIAPPSHLIRVEGNSHAQYVEDPITGRQSVLVPYEPPQATSPSPFKTPKFVLNLYLVGTEFTTVLYNFMCNSSCVGGMNRRPILIIVTLETRDGQVLGRRCFEARICACPGRDRKADEDSIRKQQVSDSTKNGDGTKRPFRQNTHGIQMTSIKKRRSPDDELLYLPVRGRETYEMLLKIKESLELMQYLPQHTIETYRQQQQQQHQHLLQKQTSMQSQSSYGNSSPPLNKMNSMNKLPSVSQLINPQQRNTLTPTTIPDGMGANIPMMGTHMPMAGDMNGLSPTQALPPPLSMPSTSHCTPPPPYPTDCSLVSFLARLGCSSCLDYFTTQGLTTIYQIEHYSMDDLASLKIPEQFRHAIWKGILDHRQLHDFSSPPHLLRTPSGASTVSVGSSETRGERVIDAVRFTLRQTISFPPRDEWNDFNFDMDARRNKQQRIKEEGE

>XP_008979632.1 tumor protein 63 isoform X1 [Callithrix jacchus]

MNFETSRCATLQYCPDPYIQRYVETPAHFSWKESYYRSTMSQSTQTNEFLSPEVFQHIWDFLEQPICSVQPIDLNFVDEPSEDGATNKIEISMDCIRMQDSDLSDPMWPQYTNLGLLNSMDQQIQNGSSSTSPYNTDHAQNSVTAPSPYAQPSSTFDALSPSPAIPSNTDYPGPHSFDVSFQQSSTAKSATWTYSTELKKLYCQIAKTCPIQIKVMTPPPQGAVIRAMPVYKKAEHVTEVVKRCPNHELSREFNEGQIAPPSHLIRVEGNSHAQYVEDPITGRQSVLVPYEPPQVGTEFTTVLYNFMCNSSCVGGMNRRPILIIVTLETRDGQVLGRRCFEARICACPGRDRKADEDSIRKQQVSDSTKNGDGTKRPFRQNTHGIQMTSIKKRRSPDDELLYLPVRGRETYEMLLKIKESLELMQYLPQHTIETYRQQQQQQHQHLLQKQTSIQSQSSYGNSSPPLNKMNSMNKLPSVSQLINPQQRNALTPTTIPDGMGANIPMMGTHMPMTGDMNGLSPTQALPPPLSMPSTSHCTPPPPYPTDCSIVSFLARLGCSSCLDYFTTQGLTTIYQIEHYSMDDLASLKIPEQFRHAIWKGILDHRQLHEFSSPSHLLRTPSSASTVSVGSSETRGERVIDAVRFTLRQTISFPPRDEWNDFNFDLDARRNKQQRIKEEGE

>XP_004038246.1 tumor protein 63 isoform X1 [Gorilla gorilla gorilla]

MNFETSRCATLQYCPDPYIQRFVETPAHFSWKESYYRSTMSQSTQTNEFLSPEVFQHIWDFLEQPICSVQPIDLNFVDEPSEDGATNKIEISMDCIRMQDSDLSDPMWPQYTNLGLLNSMDQQIQNGSSSTSPYNTDHAQNSVTAPSPYAQPSSTFDALSPSPAIPSNTDYPGPHSFDVSFQQSSTAKSATWTYSTELKKLYCQIAKTCPIQIKVMTPPPQGAVIRAMPVYKKAEHVTEVVKRCPNHELSREFNEGQIAPPSHLIRVEGNSHAQYVEDPITGRQSVLVPYEPPQVGTEFTTVLYNFMCNSSCVGGMNRRPILIIVTLETRDGQVLGRRCFEARICACPGRDRKADEDSIRKQQVSDSTKNGDGTKRPFRQNTHGIQMTSIKKRRSPDDELLYLPVRGRETYEMLLKIKESLELMQYLPQHTIETYRQQQQQQHQHLLQKQTSIQSPSSYGNSSPPLSKMNSMNKLPSVSQLINPQQRNALTPTTIPDGMGANIPMMGTHMPMAGDMNGLSPTQALPPPLSMPSTSHCTPPPPYPTDCSIVSFLARLGCSSCLDYFTTQGLTTIYQIEHYSMDDLASLKIPEQFRHAIWKGILDHRQLHEFSSPSHLLRTPSSASTVSVGSSETRGERVIDAVRFTLRQTISFPPRDEWNDFNFDMDARRNKQQRIKEEGE

>KAF6478691.1 tumor protein p63 [Molossus molossus]

MNFETARCATLQYCPDPDPYIQRFVETPAHFSWKESYYRPTMSQSTQTSEFLSPEVFQHIWDFLEQPICSVQPIDLNFVDEPSENGATNKIEISMDCIHMQDSDLSDPMWPQYTNLGLLNSMDQQIQNGSSSTSPYNTDHAQNSVTAPSPYAQPSSTFDALSPSPAIPSNTDYPGPHSFDVSFQQSSTAKSATWTYSTELKKLYCQIAKTCPIQIKVMTPPPQGAVIRAMPVYKKAEHVTEVVKRCPNHELSREFNEGQIAPPSHLIRVEGNSHAQYVEDPITGRQSVLVPYEPPQVGTEFTTVLYNFMCNSSCVGGMNRRPILIIVTLETRDGQVLGRRCFEARICACPGRDRKADEDSIRKQQVSDSTKNGDGTKRPFRQNTHGIQMTSIKKRRSPDDELLYLPVRGRETYEMLLKIKESLELMQYLPQHTIETYRQQQQQQHQHLLQKQTSMQSQSSYGNSSPPLNKMNSMNKLPSVSQLINPQQRNALTPTTIPDGMGSNIPMMGTHMPVAGDMNGLSPTQALPPPLSMPSTSHCTPPPPYPTDCSLVSFLARLGCSSCLDYFTTQGLTTIYQIEHYSMDDLASLKIPEQFRHAIWKGILDHRQLHDFSSPPHLLRTPSGTSTVSVGSSETRGERVIDAVRFTLRQTISFPPRDEWNDFNFDMDARRNKQQRIKEEGE

**>NP_003713.3 tumor protein 63 isoform 1 [Homo sapiens]**

MNFETSRCATLQYCPDPYIQRFVETPAHFSWKESYYRSTMSQSTQTNEFLSPEVFQHIWDFLEQPICSVQPIDLNFVDEPSEDGATNKIEISMDCIRMQDSDLSDPMWPQYTNLGLLNSMDQQIQNGSSSTSPYNTDHAQNSVTAPSPYAQPSSTFDALSPSPAIPSNTDYPGPHSFDVSFQQSSTAKSATWTYSTELKKLYCQIAKTCPIQIKVMTPPPQGAVIRAMPVYKKAEHVTEVVKRCPNHELSREFNEGQIAPPSHLIRVEGNSHAQYVEDPITGRQSVLVPYEPPQVGTEFTTVLYNFMCNSSCVGGMNRRPILIIVTLETRDGQVLGRRCFEARICACPGRDRKADEDSIRKQQVSDSTKNGDGTKRPFRQNTHGIQMTSIKKRRSPDDELLYLPVRGRETYEMLLKIKESLELMQYLPQHTIETYRQQQQQQHQHLLQKQTSIQSPSSYGNSSPPLNKMNSMNKLPSVSQLINPQQRNALTPTTIPDGMGANIPMMGTHMPMAGDMNGLSPTQALPPPLSMPSTSHCTPPPPYPTDCSIVSFLARLGCSSCLDYFTTQGLTTIYQIEHYSMDDLASLKIPEQFRHAIWKGILDHRQLHEFSSPSHLLRTPSSASTVSVGSSETRGERVIDAVRFTLRQTISFPPRDEWNDFNFDMDARRNKQQRIKEEGE

>KAF6476646.1 tumor protein p63 [Rousettus aegyptiacus]

MNFETSRCATLQYCPDRYIQRFVETPAHFSWKESYYRSTMSQSTQTSEFLSPEVFQHIWDFLEQPICSVQPIDLNFVDEPSENGATNKIEISMDCIRMQDSDLSDPMWPQYTNLGLLNSMDQQIQNGSSSTSPYNTDHAQNSVTAPSPYAQPSSTFDALSPSPAIPSNTDYPGPHSFDVSFQQSSTAKSATWTYSTELKKLYCQIAKTCPIQIKVMTPPPQGAVIRAMPVYKKAEHVTEVVKRCPNHELSREFNEGQVAPPSHLIRVEGNSHAQYVEDPITGRQSVLVPYEPPQVGTEFTTVLYNFMCNSSCVGGMNRRPILIIVTLETRDGQVLGRRCFEARICACPGRDRKADEDSIRKQQVSDSTKNGDGTKRPFRQNTHGIQMTSIKKRRSPDDELLYLPVRGRETYEMLLKIKESLELMQYLPQHTIETYRQQQQQQHQHLLQKQTSMQSQSSYGNSSPPLNKMNSMNKLPSVSQLINPQQRNALTPTAIPDGMGANIPMMGTHMPMAGDMNGLSPTQALPPPLSMPSTSHCTPPPPYPTDCSLVSFLARLGCSSCLDYFTTQGLTTIYQIENYNMDDLASLKIPEQFRHAIWKGILDHRQLHDFSSPPHLLRTPSGTSTVSVGSSETRGERVIDAVRFTLRQTISFPPRDEWNDFNFDMDARRNKQQRIKEEGE

>XP_024624507.1 tumor protein 63 isoform X2 [Neophocaena asiaeorientalis asiaeorientalis]

MNFETSRCATLQYCPDPYIQRFVETPAHFSWKESYYRSTMSQSTQTSEFLSPEVFQHIWDFLEQPICSVQPIDLNFVDEPSENGATNKIEISMDCIRMQDSDLSDPMWPQYTNLGLLNSMDQQIQNGSSSTSPYNTDHAQNSVTAPSPYAQPSSTFDALSPSPAIPSNTDYPGPHSFDVSFQQSSTAKSATWTYSTELKKLYCQIAKTCPIQIKVMTPPPQGAVIRAMPVYKKAEHVTEVVKRCPNHELSREFNEGQIAPPSHLIRVEGNSHAQYVEDPITGRQSVLVPYEPPQVGTEFTTVLYNFMCNSSCVGGMNRRPILIIVTLETRDGQVLGRRCFEARICACPGRDRKADEDSIRKQQVSDSTKNGDAFRQNTHGIQMTSIKKRRSPDDELLYLPVRGRETYEMLLKIKESLELMQYLPQHTIETYRQQQQQQHQHLLQKQTSMQSQSSYGNSSPPLNKMNSMNKLPSVSQLINPQQRNTLTPTTIPDGMGANIPMMGTHMPMAGDMNGLSPTQALPPPLSMPSTSHCTPPPPYPTDCSLVSFLARLGCSSCLDYFTTQGLTTIYQIEHYSMDDLASLKIPEQFRHAIWKGILDHRQLHDFSSPPHLLRTPSGASTVSVGSSETRGERVIDAVRFTLRQTISFPPRDEWNDFNFDMDARRNKQQRIKEEGE

**>XP_015146847.1 tumor protein 63 isoform X1 [Gallus gallus]**

MNFEAAPFSTLQYYPDPCIPRFVETPSHFSWKESYYRSAMSQSSQPREFLSPEVIQHIWDFLEQPICSVQPIDLNFIDDPSENGPTNKIEISMDCVRLQDTELSDPMWPQYTNLGLLNSMDQQIQNGSSSTSPYNTEHAQNSVTAPSPYAQPSSTFDALSPSPAIPSNTDYPGPHSFDVSFQQSSTAKSATWTYSTELKKLYCQIAKTCPIQIKVMTPPPQGAVIRAMPVYKKAEHVTEVVKRCPNHELSREFNEGQIAPPSHLIRVEGNSHAQYVEDPITGRQSVLVPYEPPQVGTEFTTVLYNFMCNSSCVGGMNRRPILIIVTLETRDGQVLGRRCFEARICACPGRDRKADEDSIRKQQVSDSTKNGDGTKRPFRQGTHGIQMTSIKKRRSPDDELLYLPVRGRETYEMLLKIKESLELMQYLPQHTIETYRQQQQQQHQHLLQKQTSIQSQSSYGSNSPPLSKMNSMNKLPSVSQLINPQQRNALTPTTIPDGMGTNIPMMGTHMAMTGDMNGLSPTQALPPPLSMPSTSHCTPPPPYPTDCSIVSFLARLGCSSCVDYFTTQGLTTIYQIEHYSMDDLVSLKIPEQFRHAIWKGILDHRQLHDFSSPPHLLRTPSGASTVSVGSSETRGERVIDAVRFTLRQTISFPPRDEWNDFNFDMDARRNKQQRIKEEGE

>XP_015727397.1 tumor protein 63 isoform X1 [Coturnix japonica]

MNFEAAPFSTLQYYPEPCIPRFVETPSHFSWKESYYRSAMSQSSQPREFLSPEVIQHIWDFLEQPICSVQPIDLNFIDDPSENGPTNKIEISMDCVRLQDTELSDPMWPQYTNLGLLNSMDQQIQNGSSSTSPYNTEHAQNSVTAPSPYAQPSSTFDALSPSPAIPSNTDYPGPHSFDVSFQQSSTAKSATWTYSTELKKLYCQIAKTCPIQIKVMTPPPQGAVIRAMPVYKKAEHVTEVVKRCPNHELSREFNEGQIAPPSHLIRVEGNSHAQYVEDPITGRQSVLVPYEPPQVGTEFTTVLYNFMCNSSCVGGMNRRPILIIVTLETRDGQVLGRRCFEARICACPGRDRKADEDSIRKQQVSDSTKNGDGTKRPFRQGTHGIQMTSIKKRRSPDDELLYLPVRGRETYEMLLKIKESLELMQYLPQHTIETYRQQQQQQHQHLLQKQTSIQSQSSYGSNSPPLSKMNSMNKLPSVSQLINPQQRNALTPTTIPDGMGTNIPMMGTHMSMTGDMNGLSPTQALPPPLSMPSTSHCTPPPPYPTDCSIVSFLARLGCSSCVDYFTTQGLTTIYQIEHYSMDDLVSLKIPEQFRHAIWKGILDHRQLHDFSSPPHLLRTPSGASTVSVGSSETRGERVIDAVRFTLRQTISFPPRDEWNDFNFDMDARRNKQQRIKEEGE

>XP_010715046.1 tumor protein 63 isoform X1 [Meleagris gallopavo]

MNFEAAPFSTLQYYPDPCIPRFVETPSHFSWKESYYRSAMSQSSQPREFLSPEVIQHIWDFLEQPICSVQPIDLNFIDDPSENGPTNKIEISMDCVRLQDTELTDPMWPQYTNLGLLNSMDQQIQNGSSSTSPYNTEHAQNSVTAPSPYAQPSSTFDALSPSPAIPSNTDYPGPHSFDVSFQQSSTAKSATWTYSTELKKLYCQIAKTCPIQIKVMTPPPQGAVIRAMPVYKKAEHVTEVVKRCPNHELSREFNEGQIAPPSHLIRVEGNSHAQYVEDPITGRQSVLVPYEPPQVGTEFTTVLYNFMCNSSCVGGMNRRPILIIVTLETRDGQVLGRRCFEARICACPGRDRKADEDSIRKQQVSDSTKNGDGTKRPFRQGTHGIQMTSIKKRRSPDDELLYLPVRGRETYEMLLKIKESLELMQYLPQHTIETYRQQQQQQHQHLLQKQTSIQSQSSYGSNSPPLSKMNSMNKLPSVSQLINPQQRNALTPTTIPDGMGTNIPMMGTHMAMTSDMNGLSPTQALPPPLSMPSTSHCTPPPPYPTDCSIVSFLARLGCSSCVDYFTTQGLTTIYQIEHYSMDDLVSLKIPEQFRHAIWKGILDHRQLHDFSSPPHLLRTPSGASTVSVGSSETRGERVIDAVRFTLRQTISFPPRDEWNDFNFDMDARRNKQQRIKEEGE

>NXL83524.1 P63 protein [Alectura lathami]

MNFDAAPFTTLQYYPDPCIPRFVETPSHFSWKESYYRSAMSQSSQPREFLSPEVIQHIWDFLEQPICSVQPIDLNFIDDPSANGPTNKIEISMDCVRLQDTELSDPMWPQYTNLGLLNSMDQQIQNGSSSTSPYNTEHAQNSVTAPSPYAQPSSTFDALSPSPAIPSNTDYPGPHSFDVSFQQSSTAKSATWTYSTELKKLYCQIAKTCPIQIKVMTPPPQGAVIRAMPVYKKAEHVTEVVKRCPNHELSREFNEGQIAPPSHLIRVEGNSHAQYVEDPITGRQSVLVPYEPPQVGTEFTTVLYNFMCNSSCVGGMNRRPILIIVTLETRDGQVLGRRCFEARICACPGRDRKADEDSIRKQQVSDSTKNGDGTKRPFRQGTHGIQMTSIKKRRSPDDELLYLPVRGRETYEMLLKIKESLELMQYLPQHTIETYRQQQQQQHQHLLQKQTSIQSQSSYGSSSPPLSKMNSMNKLPSVSQLINPQQRNALTPTTIPDGMGTNIPMMGTHMAMTGDMNGLSPTQALPPPLSMPSTSHCTPPPPYPTDCSIVSFLARLGCSSCVDYFTTQGLTTIYQIEHYSMDDLVSLKIPEQFRHAIWKGILDHRQLHDFSSPPHLLRTPSGASTVSVGSSETRGERVIDAVRFTLRQTISFPPRDEWNDFNFDMDARRNKQQRIKEEGE

>NXI68095.1 P63 protein [Anseranas semipalmata]

MNFEAAPFTTLQYYPDPCIQRFVETPSHFSWKESYYRSTMSQSSQPREFLSPEVIQHIWDFLEQPICSVQPIDLNFIDDPSENGPTNKIEISMDCVRLQDTDLSDPMWPQYTNLGLLNSMDQQIQNGSSSTSPYNTEHAQNSVTAPSPYAQPSSTFDALSPSPAIPSNTDYPGPHSFDVSFQQSSTAKSATWTYSTELKKLYCQIAKTCPIQIKVMTPPPQGAVIRAMPVYKKAEHVTEVVKRCPNHELSREFNEGQIAPPSHLIRVEGNSHAQYVEDPITGRQSVLVPYEPPQVGTEFTTVLYNFMCNSSCVGGMNRRPILIIVTLETRDGQVLGRRCFEARICACPGRDRKADEDSIRKQQVSDSTKNGDGTKRPFRQGTHGIQMTSIKKRRSPDDELLYLPVRGRETYEMLLKIKESLELMQYLPQHTIETYRQQQQQQHQHLLQKQTSMQSQSSYGSNSPPLSKMNSMNKLPSVSQLINPQQRNALTPTTIPDGMGTNIPMMGTHMAMTGDMNGLSPTQALPPPLSMPSTSHCTPPPPYPTDCSIVSFLARLGCSSCVDYFTTQGLTTIYQIEHYSMDDLVSLKIPEQFRHAIWKGILDHRQLHDFSSPPHLLRTPSGASTVSVGSSETRGERVIDAVRFTLRQTISFPPRDEWNDFNFDMDARRNKQQRIKEEGE

>NXW39660.1 P63 protein [Nyctiprogne leucopyga]

MNFEPAPFTTLQYYPDPCIQRFVETPSHFSWKESYYRSAMSQSSQPREFLSPEVLQHIWDFLEQPICSVQPIDLNFIDDPSENGPTNKIEISMDCVRVQDTELNDPMWPQYTNLGLLNSMDQQIQNGSSSTSPYNTEHAQNSVTAPSPYAQPSSTFDALSPSPAIPSNTDYPGPHSFDVSFQQSSTAKSATWTYSTELKKLYCQIAKTCPIQIKVMTPPPQGAVIRAMPVYKKAEHVTEVVKRCPNHELSREFNEGQIAPPSHLIRVEGNSHAQYVEDPITGRQSVLVPYEPPQVGTEFTTVLYNFMCNSSCVGGMNRRPILIIVTLETRDGQVLGRRCFEARICACPGRDRKADEDSIRKQQVSDSTKNGDGTKRPFRQGTHGIQMTSIKKRRSPDDELLYLPVRGRETYEMLLKIKESLELMQYLPQHTIETYRQQQQQQHQHLLQKQTSMQSQSSYGSNSPPLSKMNSMNKLPSVSQLINPQQRNALTPTTIPDGMGTNIPMMGTHMAMTGDMNGLSPTQALPPPLSMPSTSHCTPPPPYPTDCSIVSFLARLGCSSCVDYFTTQGLTTIYQIEHYSMDDLVSLKIPEQFRHAIWKGILDHRQLHDFSSPPHLLRTPSGASTVSVGSSETRGERVIDAVRFTLRQTISFPPRDEWNDFNFDMDARRNKQQRIKEEGE

>XP_025901079.1 tumor protein 63 isoform X2 [Nothoprocta perdicaria]

MNFEAAPFTTLQYYPDPCIQRFVETPSHFSWKESYYRSAMSQNSQPREFLSPEVLQHIWDFLEQPICSVQPIDLNFIDDPSENGPTNKIEISMDCVRVQDSELSDPMWPQYTNLGLLNSMDQQIQNGSSSTSPYNTEHAQNSVTAPSPYAQPSSTFDALSPSPAIPSNTDYPGPHSFDVSFQQSSTAKSATWTYSTELKKLYCQIAKTCPIQIKVMTPPPQGAVIRAMPVYKKAEHVTEVVKRCPNHELSREFNEGQIAPPSHLIRVEGNSHAQYVEDPITGRQSVLVPYEPPQVGTEFTTVLYNFMCNSSCVGGMNRRPILIIVTLETRDGQVLGRRCFEARICACPGRDRKADEDSIRKQQVSDSTKNGDGTKRPFRQGTHGIQMTSIKKRRSPDDELLYLPVRGRETYEMLLKIKESLELMQYLPQHTIETYRQQQQQQHQHLLQKQTSMQSQSSYGSNSPPLSKMNSMNKLPSVSQLMNPQQRNALTPTTIPDGMGTNIPMMGTHMAMTSDMNGLSPTQALPPPLSMPSTSHCTPPPPYPTDCSIVSFLARLGCSSCVDYFTTQGLTTIYQIEHYSMDDLVSLKIPEQFRHAIWKGILDHRQLHDFSSPPHLLRTPSGASTVSVGSSETRGERVIDAVRFTLRQTISFPPRDEWNDFNFDMDARRNKQQRIKEEGE

>XP_025949300.1 tumor protein 63 isoform X3 [Dromaius novaehollandiae]

MNFEAAPFTTLQYYPDPCIQRFVETPSHFSWKESYYRSAMSQNSQPRDFLSPEVLQHIWDFLEQPICSVQPIDLNFIDDPSENGPTNKIEISMDCVRVQDTELSDPMWPQYTNLGLLNSMDQQIQNGSSSTSPYNTEHAQNSVTAPSPYAQPSSTFDALSPSPAIPSNTDYPGPHSFDVSFQQSSTAKSATWTYSTELKKLYCQIAKTCPIQIKVMTPPPQGAVIRAMPVYKKAEHVTEVVKRCPNHELSREFNEGQIAPPSHLIRVEGNSHAQYVEDPITGRQSVLVPYEPPQVGTEFTTVLYNFMCNSSCVGGMNRRPILIIVTLETRDGQVLGRRCFEARICACPGRDRKADEDSIRKQQVSDSTKNGDGTKRPFRQGTHGIQMTSIKKRRSPDDELLYLPVRGRETYEMLLKIKESLELMQYLPQHTIETYRQQQQQQHQHLLQKQTSMQSQSSYGSNSPPLSKMNSMNKLPSVSQLINPQQRSALTPTTIPDGMGTNIPMMGTHMAMTSDMNGLSPTQALPPPLSMPSTSHCTPPPPYPTDCSIVSFLARLGCSSCVDYFTTQGLTTIYQIEHYSMDDLVSLKIPEQFRHAIWKGILDHRQLHDFSSPPHLLRTPSGASTVSVGSSETRGERVIDAVRFTLRQTISFPPRDEWNDFNFDMDARRNKQQRIKEEGE

>NXJ33185.1 P63 protein [Ciconia maguari]

MNFEPAPFTTLQYYPDPCIQRFVETPSHFSWKESYYRSAMSHSSQPREFLSPEVLQHIWDFLEQPICSVQPIDLNFIDGPSENGPTNKIEISMDCVRVQDTELNDPMWPQYTNLGLLNSMDQQIQNGSSSTSPYNTEHAQNSVTAPSPYAQPSSTFDALSPSPAIPSNTDYPGPHSFDVSFQQSSTAKSATWTYSTELKKLYCQIAKTCPIQIKVMTPPPQGAVIRAMPVYKKAEHVTEVVKRCPNHELSREFNEGQIAPPSHLIRVEGNSHAQYVEDPITGRQSVLVPYEPPQVGTEFTTVLYNFMCNSSCVGGMNRRPILIIVTLETRDGQVLGRRCFEARICACPGRDRKADEDSIRKQQVSDSTKNGDGTKRPFRQGTHGIQMTSIKKRRSPDDELLYLPVRGRETYEMLLKIKESLELMQYLPQHTIETYRQQQQQQHQHLLQKQTSMQSQSSYGSNSPPLSKMNSMNKLPSVSQLINPQQRNALTPTTIPDGMGTNIPMMGTHMAMTGDMNGLSPTQALPPPLSMPSTSHCTPPPPYPTDCSIVSFLARLGCSSCVDYFTTQGLTTIYQIEHYSMDDLVSLKIPEQFRHAIWKGILDHRQLHDFSSPPHLLRTPSGASTVSVGSSETRGERVIDAVRFTLRQTISFPPRDEWNDFNFDMDARRNKQQRIKEEGE

>XP_008491544.1 tumor protein 63 [Calypte anna]

MNFEAAPFTTLQYYPDPCIQRFVETPSHFSWKESYYRSAMSQSSQPRDFLSPEMLQHIWDFLEQPICSVQPIDLNFIDGPSENGSTNKIEISMDCVRVQDTELNDPMWPQYTNLGLLNSMDQQIQNGSSSTSPYNTEHAQNSVTAPSPYAQPSSTFDALSPSPAIPSNTDYPGPHSFDVSFQQSSTAKSATWTYSTELKKLYCQIAKTCPIQIKVMTPPPQGAVIRAMPVYKKAEHVTEVVKRCPNHELSREFNEGQIAPPSHLIRVEGNSHAQYVEDPITGRQSVLVPYEPPQVGTEFTTVLYNFMCNSSCVGGMNRRPILIIVTLETRDGQVLGRRCFEARICACPGRDRKADEDSIRKQQVSDSTKNGDGTKRPFRQGTHGIQMTSIKKRRSPDDELLYLPVRGRETYEMLLKIKESLELMQYLPQHTIETYRQQQQQQHQHLLQKQTSMQSQSSYGSNSPPLSKMNSMNKLPSVSQLINPQQRNALTPTTIPDGMGTNIPMMGTHMAMTGDMNGLSPTQALPPPLSMPSTSHCTPPPPYPTDCSIVSFLARLGCSSCVDYFTTQGLTTIYQIEHYSMDDLVSLKIPEQFRHAIWKGILDHRQLHDFSSPPHLLRTPSGASTVSVGSSETRGERVIDAVRFTLRQTISFPPRDEWNDFNFDMDARRNKQQRIKEEGE

>NXS91673.1 P63 protein [Jacana jacana]

MNFEPAPFTTLQYYPDPCIQRFVETPSHFSWKESYYRSAMSQSSQPREFLSPEVLQHIWDFLEQPICSVQPIDLNFIDGPSENGSTNKIEISMDCVRVQDTELNDPMWPQYTNLGLLNSMDQQIQNGSSSTSPYNTEHAQNSVTAPSPYAQPSSTFDALSPSPAIPSNTDYPGPHSFDVSFQQSSTAKSATWTYSTELKKLYCQIAKTCPIQIKVMTPPPQGAVIRAMPVYKKAEHVTEVVKRCPNHELSREFNEGQIAPPSHLIRVEGNSHAQYVEDPITGRQSVLVPYEPPQVGTEFTTVLYNFMCNSSCVGGMNRRPILIIVTLETRDGQVLGRRCFEARICACPGRDRKADEDSIRKQQVSDSTKNGDGTKRPFRQGTHGIQMTSIKKRRSPDDELLYLPVRGRETYEMLLKIKESLELMQYLPQHTIETYRQQQQQQHQHLLQKQTSMQSQSSYGSNSPPLSKMNSMNKLPSVSQLINPQQRNALTPTTIPDGMGTNIPMMGTHMAMTGDMNGLSPTQALPPPLSMPSTSHCTPPPPYPTDCSIVSFLARLGCSSCVDYFTTQGLTTIYQIEHYSMDDLVSLKIPEQFRHAIWKGILDHRQLHDFSSPPHLLRTPSGASTVSVGSSETRGERVIDAVRFTLRQTISFPPRDEWNDFNFDMDARRNKQQRIKEEGE

>NXU71318.1 P63 protein [Oreotrochilus melanogaster]

MNFEAAPFTTLQYYPDPCIQRFVETPSHFSWKESYYRSAMSQSSQPRDFLSPEMLQHIWDFLEQPICSVQPIDLNFIDGPSENGSTNKIEISMDCVRVQDTELNDPMWPQYTNLGLLNSMDQQIQNGSSSTSPYNTEHAQNSVTAPSPYAQPSSTFDALSPSPAIPSNTDYPGPHSFDVSFQQSSTAKSATWTYSTELKKLYCQIAKTCPIQIKVMTPPPQGAVIRAMPVYKKAEHVTEVVKRCPNHELSREFNEGQIAPPSHLIRVEGNSHAQYVEDPITGRQSVLVPYEPPQVGTEFTTVLYNFMCNSSCVGGMNRRPILIIVTLETRDGQVLGRRCFEARICACPGRDRKADEDSIRKQQVSDSTKNGDGTKRPFRQGTHGIQMTSIKKRRSPDDELLYLPVRGRETYEMLLKIKESLELMQYLPQHTIETYRQQQQQQHQHLLQKQTSMQSQSSYGSNSPPLSKMNSMNKLPSVSQLINPQQRNALTPTTIPDGMGTNIPMMGTHMAMTGDMNGLSPTQALPPPLSMPSTSHCTPPPPYPTDCSIVSFLARLGCSSCVDYFTTQGLTTIYQIEHYSMDDLVSLKIPEQFRHAIWKGILDHRQLHDFSSPPHLLRTPSGTSTVSVGSSETRGERVIDAVRFTLRQTISFPPRDEWNDFNFDMDARRNKQQRIKEEGE

>PKK20940.1 tumor protein p63, transcript variant X1 [Columba livia]

MNFEPAPFTTLQYYPDPCIQRFVETPSQFSWKESYYRSAMSQNSQPREFLSPEVLQHIWDFLEQPICSVQPIDLNFIDGPSENGPTNKIEISMDCVRVQDTELNDPMWPQYTNLGLLNSMDQQIQNGSSSTSPYNTEHAQNSVTAPSPYAQPSSTFDALSPSPAIPSNTDYPGPHSFDVSFQQSSTAKSATWTYSTELKKLYCQIAKTCPIQIKVMTPPPQGAVIRAMPVYKKAEHVTEVVKRCPNHELSREFNEGQIAPPSHLIRVEGNSHAQYVEDPITGRQSVLVPYEPPQVGTEFTTVLYNFMCNSSCVGGMNRRPILIIVTLETRDGQVLGRRCFEARICACPGRDRKADEDSIRKQQVSDSTKNGDGTKRPFRQGTHGIQMTSIKKRRSPDDELLYLPVRGRETYEMLLKIKESLELMQYLPQHTIETYRQQQQQQHQHLLQKQTSMQSQSSYGSNSPPLSKMNSMNKLPSVSQLINPQQRNALTPTTIPDGMGTNIPMMGTHMAMTGDMNGLSPTQALPPPLSMPSTSHCTPPPPYPTDCSIVSFLARLGCSSCVDYFTTQGLTTIYQIEHYSMDDLVSLKIPEQFRHAIWKGILDHRQLHDFSSPPHLLRTPSGASTVSVGSSETRGERVIDAVRFTLRQTISFPPRDEWNDFNFDMDARRNKQQRIKEEGE

>NXP08170.1 P63 protein [Thinocorus orbignyianus]

MNFEPVPFTTLQYYPEPCIQRFVETPSHFSWKESYYRSAMSQSSQPREFLSPEVLQHIWDFLEQPICSVQPIDLNFIDGPSENGATNKIEISMDCVRVQDTELNDPMWPQYTNLGLLNSMDQQIQNGSSSTSPYNTEHAQNSVTAPSPYAQPSSTFDALSPSPAIPSNTDYPGPHSFDVSFQQSSTAKSATWTYSTELKKLYCQIAKTCPIQIKVMTPPPQGAVIRAMPVYKKAEHVTEVVKRCPNHELSREFNEGQIAPPSHLIRVEGNSHAQYVEDPITGRQSVLVPYEPPQVGTEFTTVLYNFMCNSSCVGGMNRRPILIIVTLETRDGQVLGRRCFEARICACPGRDRKADEDSIRKQQVSDSTKNGDGTKRPFRQGTHGIQMTSIKKRRSPDDELLYLPVRGRETYEMLLKIKESLELMQYLPQHTIETYRQQQQQQHQHLLQKQTSMQSQSSYGSNSPPLSKMNSMNKLPSVSQLINPQQRNALTPTTIPDGMGTNIPMMGTHMAMTGDMNGLSPTQALPPPLSMPSTSHCTPPPPYPTDCSIVSFLARLGCSSCVDYFTTQGLTTIYQIEHYSMDDLVSLKIPEQFRHAIWKGILDHRQLHDFSSPPHLLRTPSGASTVSVGSGETRGERVIDAVRFTLRQTISFPPRDEWNDFNFDMDARRNKQQRIKEEGE

>NWT23121.1 P63 protein [Cardinalis cardinalis]

MNFEAAPFSTLPYYPDPCIPRFVETPSPFSWKESYYRSAMSQSSQPREFLSPEVIQHIWDFLEQPICSVQPIDLNFIDGPSEDGSTNKIEISMDCVRVQDTELNDPMWPQYTNLGLLNSMDQQIQNGSSSTSPYNTEHAQNSVTAPSPYAQPSSTFDALSPSPAIPSNTDYPGPHSFDVSFQQSSTAKSATWTYSTELKKLYCQIAKTCPIQIKVMTPPPQGAVIRAMPVYKKAEHVTEVVKRCPNHELSREFNEGQIAPPSHLIRVEGNSHAQYVEDPITGRQSVLVPYEPPQVGTEFTTVLYNFMCNSSCVGGMNRRPILIIVTLETRDGQVLGRRCFEARICACPGRDRKADEDSIRKQQVSDSTKNGDGTKRPFRQGTHGIQMTSIKKRRSPDDELLYLPVRGRETYEMLLKIKESLELMQFLPQHTIETYRQQQQQQHQHLLQKQTSMQSQSSYGSNSPPLSKMNSMNKLPSVSQLINPQQRNALTPTTIPDSMGTNIPMMGTHMAMTGDMNGLSPTQALPPPLSMPSTSHCTPPPPYPSDCSIVSFLARLGCSSCVDYFTTQGLTTIYQIEHYSMDDLVSLKIPEQFRHAIWKGILDHRQLHDFSSPPHLLRTPSGASTVSVGSSETRGERVIDAVRFTLRQTISFPPRDEWNDFNFDMDARRNKQQRIKEEGE

>NWH85614.1 P63 protein [Aegithalos caudatus]

MNFEAAPFTTLPYYPDPCIPRFVETPSPFSWKESYYRSAMSQSSQPREFFSPEVIQHIWDFLEQPICSVQPIDLNFIDGPSEDGSTNKIEISMDCVRVQDTELNDPMWPQYTNLGLLNSMDQQIQNGSSSTSPYNTEHAQNSVTAPSPYAQPSSTFDALSPSPAIPSNTDYPGPHSFDVSFQQSSTAKSATWTYSTELKKLYCQIAKTCPIQIKVMTPPPQGAVIRAMPVYKKAEHVTEVVKRCPNHELSREFNEGQIAPPSHLIRVEGNSHAQYVEDPITGRQSVLVPYEPPQVGTEFTTVLYNFMCNSSCVGGMNRRPILIIVTLETRDGQVLGRRCFEARICACPGRDRKADEDSIRKQQVSDSTKNGDGTKRPFRQGTHGIQMTSIKKRRSPDDELLYLPVRGRETYEMLLKIKESLELMQYLPQHTIETYRQQQQQQHQHLLQKQTSMQSQSSYGSNSPPLSKMNSMNKLPSVSQLINPQQRNALTPTTIPDSMGTNIPMMGTHMAMTGDMNGLSPTQALPPPLSMPSTSHCTPPPPYPSDCSIVSFLARLGCSSCVDYFTTQGLTTIYQIEHYSMDDLVSLKIPEQFRHAIWKGILDHRQLHDFSSPPHLLRTPSGASTVSVGSSETRGERVIDAVRFTLRQTISFPPRDEWNDFNFDMDARRNKQQRIKEEGE

>NXR49230.1 P63 protein [Hippolais icterina]

MNFEAAPFTTLPYYPDPCIQRFVETPSPFSWKESYYRSAMSQSSQPREFLSPEVIQHIWDFLEQPICSVQPIDLNFIDGPSEDGSTNKIEISMDCVRVQDTELSDPMWPQYTNLGLLNSMDQQIQNGSSSTSPYNTEHAQNSVTAPSPYAQPSSTFDALSPSPAIPSNTDYPGPHSFDVSFQQSSTAKSATWTYSTELKKLYCQIAKTCPIQIKVMTPPPQGAVIRAMPVYKKAEHVTEVVKRCPNHELSREFNEGQIAPPSHLIRVEGNSHAQYVEDPITGRQSVLVPYEPPQVGTEFTTVLYNFMCNSSCVGGMNRRPILIIVTLETRDGQVLGRRCFEARICACPGRDRKADEDSIRKQQVSDSTKNGDGTKRPFRQGTHGIQMTSIKKRRSPDDELLYLPVRGRETYEMLLKIKESLELMQYLPQHTIETYRQQQQQQHQHLLQKQTSMQSQSSYGSNSPPLSKMNSMNKLPSVSQLINPQQRNALTPTTIPDSMGTNIPMMGTHMAMTGDMNGLSPTQALPPPLSMPSTSHCTPPPPYPSDCSIVSFLARLGCSSCVDYFTTQGLTTIYQIEHYSMDDLVSLKIPEQFRHAIWKGILDHRQLHDFSSPPHLLRTPSGASTVSVGSSETRGERVIDAVRFTLRQTISFPPRDEWNDFNFDMDARRNKQQRIKEEGE

>XP_038039726.1 tumor protein 63 isoform X2 [Anas platyrhynchos]

MNFEAAPFTTLQYYPDPCIQRFVETPSHFSWKESYYRSAMSQSSQPREFLSPEVIQHIWDFLEQPICSVQPIDLNFIDDPSENGPTNKIEISMDCVRLQDTELSDPMWPQYTNLGLLNSMDQQIQNGSSSTSPYNTEHTQNSVTAPSPYAQPSSTFDALSPSPAIPSNTDYPGPHSFDVSFQQSSTAKSATWTYSTELKKLYCQIAKTCPIQIKVLTPPPQGAVIRAMPVYKKAEHVTEVVKRCPNHELSREFNEGQIAPPSHLIRVEGNSHAQYVEDPITGRQSVLVPYEPPQVGTEFTTVLYNFMCNSSCVGGMNRRPILIIVTLETRDGQVLGRRCFEARICACPGRDRKADEDSIRKQQVSDSTKNGDAFRQGTHGIQMTSIKKRRSPDDELLYLPVRGRETYEMLLKIKESLELMQYLPQHTIETYRQQQQQQHQHLLQKQTSMQSQSSYGSNSPPLSKMNSMNKLPSVSQLINPQQRNALTPTTIPDGMGTNIPMMGTHMAMTGDMNGLSPTQALPPPLSMPSTSHCTPPPPYPTDCSIVSFLARLGCSSCVDYFTTQGLTTIYQIEHYSMDDLVSLKIPEQFRHAIWKGILDHRQLHDFSSPPHLLRTPSGASTVSVGSSETRGERVIDAVRFTLRQTISFPPRDEWNDFNFDMDARRNKQQRIKEEGE

>XP_036244673.1 tumor protein 63 isoform X1 [Molothrus ater]

MNFEAAPFSTLPYYPDPCIPRFVETPSPFSWKESYYRSAMSQSSQPREFLSPEVIQHIWDFLEQPICSVQPIDLNFIDGPSEDGSTNKIEISMDCVRVQDTELNDPMWPQYTNLGLLNSMDQQIQNGSSSTSPYNTEHAQNSVTAPSPYAQPSSTFDALSPSPAIPSNTDYPGPHSFDVSFQQSSTAKSATWTYSTELKKLYCQIAKTCPIQIKVMTPPPQGAVIRAMPVYKKAEHVTEVVKRCPNHELSREFNEGQIAPPSHLIRVEGNSHAQYVEDPITGRQSVLVPYEPPQVGTEFTTVLYNFMCNSSCVGGMNRRPILIIVTLETRDGQVLGRRCFEARICACPGRDRKADEDSIRKQQVSDSTKNGDGTKRPFRQGTHGIQMTSIKKRRSPDDELLYLPVRGRETYEMLLKIKESLELMQFLPQHTIETYRQQQQQQHQHLLQKQTSMQSQSSYGSSSPPLSKMNSMNKLPSVSQLINPQQRNALTPTTIPDSMGTNIPMMGTHMAMTGDMNGLSPTQALPPPLSMPSTSHCTPPPPYPSDCSIVSFLARLGCSSCVDYFTTQGLTTIYQIEHYSMDDLVSLKIPEQFRHAIWKGILDHRQLHDFSSPPHLLRTPSGASTVSVGSSETRGERVIDAVRFTLRQTISFPPRDEWNDFNFDMDARRNKQQRIKEEGE

>NXO83005.1 P63 protein [Sitta europaea]

MNFEAAPFSTLPYYPDPCIQRFVETPSPFSWKESYYRSAMSQSSQPREFLSPEVIQHIWDFLEQPICSVQPIDLNFIDGPSEDGSTNKIEISMDCVRVQDTELNDPMWPQYTNLGLLNSMDQQIQNGSSSTSPYNTEHAQNSVTAPSPYAQPSSTFDALSPSPAIPSNTDYPGPHSFDVSFQQSSTAKSATWTYSTELKKLYCQIAKTCPIQIKVMTPPPQGAVIRAMPVYKKAEHVTEVVKRCPNHELSREFNEGQIAPPSHLIRVEGNSHAQYVEDPITGRQSVLVPYEPPQVGTEFTTVLYNFMCNSSCVGGMNRRPILIIVTLETRDGQVLGRRCFEARICACPGRDRKADEDSIRKQQVSDSTKNGDGTKRPFRQGTHGIQMTSIKKRRSPDDELLYLPVRGRETYEMLLKIKESLELMQYLPQHTIETYRQQQQQQHQHLLQKQTSMQSQSSYGSNSPPLSKMNSMNKLPSVSQLINPQQRNALTPTTIPDSMGTNIPMMGTHMAMTGDMNGLSPTQALPPPLSMPSTSHCTPPPPYPSDCSIVSFLARLGCSSCVDYFTTQGLTTIYQIEHYSMDDLVSLKIPEQFRHAIWKGILDHRQLHEFSSPPHLLRTPSGASTVSVGSSETRGERVIDAVRFTLRQTISFPPRDEWNDFNFDMDARRNKQQRIKEEGE

>NWU04218.1 P63 protein [Urocynchramus pylzowi]

MNFEAAPFTTLPYYPDPCIQRFVETPSPFSWKESYYRSAMSQSSQPREFLSPEVIQHIWDFLEQPICSVQPIDLNFIDGPSEDGSTNKIEISMDCVRVQDTELSDPMWPQYTNLGLLNSMDQQIQNGSSSTSPYNTEHAQNSVTAPSPYAQPSSTFDALSPSPAIPSNTDYPGPHSFDVSFQQSSTAKSATWTYSTELKKLYCQIAKTCPIQIKVMTPPPQGAVIRAMPVYKKAEHVTEVVKRCPNHELSREFNEGQIAPPSHLIRVEGNSHAQYVEDPITGRQSVLVPYEPPQVGTEFTTVLYNFMCNSSCVGGMNRRPILIIVTLETRDGQVLGRRCFEARICACPGRDRKADEDSIRKQQVSDSTKNGDGTKRPFRQGTHGIQMTSIKKRRSPDDELLYLPVRGRETYEMLLKIKESLELMQFLPQHTIETYRQQQQQQHQHLLQKQTSMQSQSSYGSNSPPLSKMNSMNKLPSVSQLINPQQRNALTPTTIPDSMGTNIPMMGTHMAMTGDMNGLSPTQALPPPLSMPSTSHCTPPPPYPSDCSIVSFLARLGCSSCVDYFTTQGLTTIYQIEHYSMDDLVSLKIPEQFRHAIWKGILDHRQLHDFSSPPHLLRTPSGASTVSVGSSETRGERVIDAVRFTLRQTISFPPRDEWNDFNFDMDARRNKQQRIKEEGE

>NXH03158.1 P63 protein [Loxia leucoptera]

MNFEAAPFSTLPYYPDPCIPRFVETPSPFSWKESYYRSAMSQSSQPREFLSPEVIQHIWDFLEQPICSVQPIDLNFIDGPSEDGSTNKIEISMDCVRVQDTELNDPMWPQYTNLGLLNSMDQQIQNGSSSTSPYNTEHAQNSVTAPSPYAQPSSTFDALSPSPAIPSNTDYPGPHSFDVSFQQSSTAKSATWTYSTELKKLYCQIAKTCPIQIKVMTPPPQGAVIRAMPVYKKAEHVTEVVKRCPNHELSREFNEGQIAPPSHLIRVEGNSHAQYVEDPITGRQSVLVPYEPPQVGTEFTTVLYNFMCNSSCVGGMNRRPILIIVTLETRDGQVLGRRCFEARICACPGRDRKADEDSIRKQQVSDSTKNGDGTKRPFRQGTHGIQMTSIKKRRSPDDELLYLPVRGRETYEMLLKIKESLELMQFLPQHTIETSRQQQQQQHQHLLQKQTSMQSQSSYGSNSPPLSKMNSMNKLPSVSQLINPQQRNALTPTTIPDSMGTNIPMMGTHMAMTGDMNGLSPTQALPPPLSMPSTSHCTPPPPYPSDCSIVSFLARLGCSSCVDYFTTQGLTTIYQIEHYSMDDLVSLKIPEQFRHAIWKGILDHRQLHDFSSPPHLLRTPSGASTVSVGSSETRGERVIDAVRFTLRQTISFPPRDEWNDFNFDMDARRNKQQRIKEEGE

>XP_021392473.1 tumor protein 63 isoform X1 [Lonchura striata domestica]

MHDHSSMKSGLRSLSPRRGWKAMNFEAAPFSTLPYYPDPCIQRFVETPSPFSWKESYYRSAMSQSSQPREFLSPEVIQHIWDFLEQPICSVQPIDLNFIDGPSEDGSTNKIEISMDCVRVQDTELNDPMWPQYTNLGLLNSMDQQIQNGSSSTSPYNTEHAQNSVTAPSPYAQPSSTFDALSPSPAIPSNTDYPGPHSFDVSFQQSSTAKSATWTYSTELKKLYCQIAKTCPIQIKVMTPPPQGAVIRAMPVYKKAEHVTEVVKRCPNHELSREFNEGQIAPPSHLIRVEGNSHAQYVEDPITGRQSVLVPYEPPQVGTEFTTVLYNFMCNSSCVGGMNRRPILIIVTLETRDGQVLGRRCFEARICACPGRDRKADEDSIRKQQVSDSTKNGDGTKRPFRQGTHGIQMTSIKKRRSPDDELLYLPVRGRETYEMLLKIKESLELMQFLPQHTIETYRQQQQQQHQHLLQKQTSMQSQSSYGSNSPPLSKMNSMNKLPSVSQLINPQQRNALTPTTIPDSMGTNIPMMGTHMAMTGDMNGLSPTQALPPPLSMPSTSHCTPPPPYPSDCSIVSFLARLGCSSCVDYFTTQGLTTIYQIEHYSMDDLVSLKIPEQFRHAIWKGILDHRQLHDFSSPPHLLRTPSGASTVSVGSSETRGERVIDAVRFTLRQTISFPPRDEWNDFNFDMDARRNKQQRIKEEGE

>NWV90915.1 P63 protein [Machaerirhynchus nigripectus]

MNFEAAPFTTLPYYPDPCIQRFVETPSPFSWKESYYRSTMSQSSQPREFLSPEVIQHIWDFLEQPICSVQPIDLNFIDGPSEDGSTNKIEISMDCVRVQDTELNDPMWPQYTNLGLLNSMDQQIQNGSSSTSPYNTEHAQNSVTAPSPYAQPSSTFDALSPSPAIPSNTDYPGPHSFDVSFQQSSTAKSATWTYSTELKKLYCQIAKTCPIQIKVMTPPPQGAVIRAMPVYKKAEHVTEVVKRCPNHELSREFNEGQIAPPSHLIRVEGNSHAQYVEDPITGRQSVLVPYEPPQVGTEFTTVLYNFMCNSSCVGGMNRRPILIIVTLETRDGQVLGRRCFEARICACPGRDRKADEDSIRKQQVSDSTKNGDGTKRPFRQGTHGIQMTSIKKRRSPDDELLYLPVRGRETYEMLLKIKESLELMQYLPQHTIETYRQQQQQQHQHLLQKQTSIQSQSSYGSNSPPLSKMNSMNKLPSVSQLINPQQRNALTPTTIPDSMGTNIPMMGTHMAMTGDMNGLSPTQALPPPLSMPSTSHCTPPPPYPSDCSIVSFLARLGCSSCVDYFTTQGLTTIYQIEHYSMDDLVSLKIPEQFRHAIWKGILDHRQLHDFSSPPHLLRTPSGASTVSVGSSETRGERVIDAVRFTLRQTISFPPRDEWNDFNFDMDARRNKQQRIKEEGE

>NXQ63462.1 P63 protein [Anthoscopus minutus]

MNFEAAPFTTLPYYPDPCIQRFVETPSPFSWKESYYRSAMSQSSQPREFLSPEVIQHIWDFLEQPICSVQPIDLNFIDGPAEDGSTNKIEISMDCVRVQDTELNDPMWPQYTNLGLLNSMDQQIQNGSSSTSPYNTEHAQNSVTAPSPYAQPSSTFDALSPSPAIPSNTDYPGPHSFDVSFQQSSTAKSATWTYSTELKKLYCQIAKTCPIQIKVMTPPPQGAVIRAMPVYKKAEHVTEVVKRCPNHELSREFNEGQIAPPSHLIRVEGNSHAQYVEDPITGRQSVLVPYEPPQVGTEFTTVLYNFMCNSSCVGGMNRRPILIIVTLETRDGQVLGRRCFEARICACPGRDRKADEDSIRKQQVSDSTKNGDGTKRPFRQGTHGIQMTSIKKRRSPDDELLYLPVRGRETYEMLLKIKESLELMQYLPQHTIETYRQQQQQQHQHLLQKQTSMQSQSSYGSNSPPLSKMNSMNKLPSVSQLINPQQRNALTPTTIPDSMGTNIPMMGTHMAMTGDMNGLSPTQALPPPLSMPSTSHCTPPPPYPSDCSIVSFLARLGCSSCVDYFTTQGLTTIYQIEHYSMDDLVSLKIPEQFRHAIWKGILDHRQLHDFSSPPHLLRTPSGASTVSVGSSETRGERVIDAVRFTLRQTISFPPRDEWNDFNFDMDARRNKQQRIKEEGE

>NXM11403.1 P63 protein [Ploceus nigricollis]

MNFEAAPFTTLPYYPDPCIQRFVETPSPFSWKESYYRSAMSQSSQPREFLSPEVIQHIWDFLEQPICSVQPIDLNFIDGPSEDGSTNKIEISMDCVRVQDTELNDPMWPQYTNLGLLNSMDQQIQNGSSSTSPYNTEHAQNSVTAPSPYAQPSSTFDALSPSPAIPSNTDYPGPHSFDVSFQQSSTAKSATWTYSTELKKLYCQIAKTCPIQIKVMTPPPQGAVIRAMPVYKKAEHVTEVVKRCPNHELSREFNEGQIAPPSHLIRVEGNSHAQYVEDPITGRQSVLVPYEPPQVGTEFTTVLYNFMCNSSCVGGMNRRPILIIVTLETRDGQVLGRRCFEARICACPGRDRKADEDSIRKQQVSDSTKNGDGTKRPFRQGTHGIQMTSIKKRRSPDDELLYLPVRGRETYEMLLKIKESLELMQFLPQHTIETYRQQQQQQHQHLLQKQTSMQSQSSYGSNSPPLSKMNSMNKLPSVSQLINPQQRNALTPTTIPDSMGTNIPMMGTHMAMTGDMNGLSPTQALPPPLSMPSTSHCTPPPPYPSDCSIVSFLARLGCSSCVDYFTTQGLTTIYQIEHYSMDDLVSLKIPEQFRHAIWKGILDHRQLHDFSSPPHLLRTPSGASTVSVGSSETRGERVIDAVRFTLRQTISFPPRDEWNDFNFDMDARRNKQQRIKEEGE

>NWW09946.1 P63 protein [Oreocharis arfaki]

MNFEAAPFTTLPYYPDPCIQRFVETPSPFSWKESYYRSAMSQSSQPREFLSPEVIQHIWDFLEQPICSVQPIDLNFIDGPSEDGSTNKIEISMDCVRVQDTELNDPMWPQYTNLGLLNSMDQQIQNGSSSTSPYNTEHAQNSVTAPSPYAQPSSTFDALSPSPAIPSNTDYPGPHSFDVSFQQSSTAKSATWTYSTELKKLYCQIAKTCPIQIKVMTPPPQGAVIRAMPVYKKAEHVTEVVKRCPNHELSREFNEGQIAPPSHLIRVEGNSHAQYVEDPITGRQSVLVPYEPPQVGTEFTTVLYNFMCNSSCVGGMNRRPILIIVTLETRDGQVLGRRCFEARICACPGRDRKADEDSIRKQQVSDSTKNGDGTKRPFRQGTHGIQMTSIKKRRSPDDELLYLPVRGRETYEMLLKIKESLELMQYLPQHTIETYRQQQQQQHQHLLQKQTSIQSQSSYGSSSPPLSKMNSMNKLPSVSQLINPQQRNALTPTTIPDSMGTNIPMMGTHMAMTGDMNGLSPTQALPPPLSMPSTSHCTPPPPYPSDCSIVSFLARLGCSSCVDYFTTQGLTTIYQIEHYSMDDLVSLKIPEQFRHAIWKGILDHRQLHDFSSPPHLLRTPSGASTVSVGSSETRGERVIDAVRFTLRQTISFPPRDEWNDFNFDMDARRNKQQRIKEEGE

>NXA78893.1 P63 protein [Thryothorus ludovicianus]

MNFEAAPFTTLPYYPDPCIQRFVETPSPFSWKESYYRSAMSQSSQPREFLSPEVIQHIWDFLEQPICSVQPIDLNFIDGPSEDGSTNKIEISMDCVRVQDTELNDPMWPQYTNLGLLNSMDQQIQNGSSSTSPYNTEHAQNSVTAPSPYAQPSSTFDALSPSPAIPSNTDYPGPHSFDVSFQQSSTAKSATWTYSTELKKLYCQIAKTCPIQIKVMTPPPQGAVIRAMPVYKKAEHVTEVVKRCPNHELSREFNEGQIAPPSHLIRVEGNSHAQYVEDPITGRQSVLVPYEPPQVGTEFTTVLYNFMCNSSCVGGMNRRPILIIVTLETRDGQVLGRRCFEARICACPGRDRKADEDSIRKQQVSDSTKNGDGTKRPFRQGTHGIQMTSIKKRRSPDDELLYLPVRGRETYEMLLKIKESLELMQYLPQHTIETYRQQQQQQHQHLLQKQTSMQSQSSYGSNSPPLSKMNSMNKLPSVSQLINPQQRNALTPTTIPDSMGTNIPMMGTHMAMTGDMNGLSPTQALPPPLSMPSTSHCTPPPPYPSDCSIVSFLARLGCSSCVDYFTTQGLTTIYQIEHYSMDDLVSLKIPEQFRHAIWKGILDHRQLHEFSSPPHLLRTPSGASTVSVGSSETRGERVIDAVRFTLRQTISFPPRDEWNDFNFDMDARRNKQQRIKEEGE

>NXB25779.1 P63 protein [Rhagologus leucostigma]

MNFEAAPFTTLPYYPDPCIQRFVETPSPFSWKENYYRSAMSQSSQPREFLSPEVIQHIWDFLEQPICSVQPIDLNFIDGPSEDGSTNKIEISMDCVRVQDTELNDPMWPQYTNLGLLNSMDQQIQNGSSSTSPYNTEHAQNSVTAPSPYAQPSSTFDALSPSPAIPSNTDYPGPHSFDVSFQQSSTAKSATWTYSTELKKLYCQIAKTCPIQIKVMTPPPQGAVIRAMPVYKKAEHVTEVVKRCPNHELSREFNEGQIAPPSHLIRVEGNSHAQYVEDPITGRQSVLVPYEPPQVGTEFTTVLYNFMCNSSCVGGMNRRPILIIVTLETRDGQVLGRRCFEARICACPGRDRKADEDSIRKQQVSDSTKNGDGTKRPFRQGTHGIQMTSIKKRRSPDDELLYLPVRGRETYEMLLKIKESLELMQYLPQHTIETYRQQQQQQHQHLLQKQTSMQSQSSYGSNSPPLSKMNSMNKLPSVSQLINPQQRNALTPTTIPDSMGTNIPMMGTHMAMTGDMNGLSPTQALPPPLSMPSTSHCTPPPPYPSDCSIVSFLARLGCSSCVDYFTTQGLTTIYQIEHYSMDDLVSLKIPEQFRHAIWKGILDHRQLHDFSSPPHLLRTPSGASTVSVGSSETRGERVIDAVRFTLRQTISFPPRDEWNDFNFDMDARRNKQQRIKEEGE

>NXR60690.1 P63 protein [Rhadina sibilatrix]

MNFEAAPFTALPYYPDPCIQRFVETPSPFSWKESYYRSAMSQSSQPREFLSPEVIQHIWDFLEQPICSVQPIDLNFIDGPSEDGSTNKIEISMDCVRVQDTELNDPMWPQYTNLGLLNSMDQQIQNGSSSTSPYNTEHAQNSVTAPSPYAQPSSTFDALSPSPAIPSNTDYPGPHSFDVSFQQSSTAKSATWTYSTELKKLYCQIAKTCPIQIKVMTPPPQGAVIRAMPVYKKAEHVTEVVKRCPNHELSREFNEGQIAPPSHLIRVEGNSHAQYVEDPITGRQSVLVPYEPPQVGTEFTTVLYNFMCNSSCVGGMNRRPILIIVTLETRDGQVLGRRCFEARICACPGRDRKADEDSIRKQQVSDSTKNGDGTKRPFRQGTHGIQMTSIKKRRSPDDELLYLPVRGRETYEMLLKIKESLELMQYLPQHTIETYRQQQQQQHQHLLQKQTSMQSQSSYGSNSPPLSKMNSMNKLPSVSQLINPQQRNALTPTTIPDSMGTNIPMMGTHMAMTGDMNGLSPTQALPPPLSMPSTSHCTPPPPYPSDCSIVSFLARLGCSSCVDYFTTQGLTTIYQIEHYSMDDLVSLKIPEQFRHAIWKGILDHRQLHDFSSPPHLLRTPSGASTVSVGSSETRGERVIDAVRFTLRQTISFPPRDEWNDFNFDMDARRNKQQRIKEEGE

>NXL16097.1 P63 protein [Setophaga kirtlandii]

MNFEAAPFSTLPYYPDPCIPRFVETPSPFSWKESCYRSAMSQSSQPREFLSPEVIQHIWDFLEQPICSVQPIDLNFIDGPNEDGSTNKIEISMDCVRVQDTELNDPMWPQYTNLGLLNSMDQQIQNGSSSTSPYNTEHAQNSVTAPSPYAQPSSTFDALSPSPAIPSNTDYPGPHSFDVSFQQSSTAKSATWTYSTELKKLYCQIAKTCPIQIKVMTPPPQGAVIRAMPVYKKAEHVTEVVKRCPNHELSREFNEGQIAPPSHLIRVEGNSHAQYVEDPITGRQSVLVPYEPPQVGTEFTTVLYNFMCNSSCVGGMNRRPILIIVTLETRDGQVLGRRCFEARICACPGRDRKADEDSIRKQQVSDSTKNGDGTKRPFRQGTHGIQMTSIKKRRSPDDELLYLPVRGRETYEMLLKIKESLELMQFLPQHTIETYRQQQQQQHQHLLQKQTSMQSQSSYGSNSPPLSKMNSMNKLPSVSQLINPQQRNALTPTTIPDSMGTNIPMMGTHMAMTGDMNGLSPTQALPPPLSMPSTSHCTPPPPYPSDCSIVSFLARLGCSSCVDYFTTQGLTTIYQIEHYSMDDLVSLKIPEQFRHAIWKGILDHRQLHDFSSPPHLLRTPSGASTVSVGSSETRGERVIDAVRFTLRQTISFPPRDEWNDFNFDMDARRNKQQRIKEEGE

>XP_027503362.1 tumor protein 63 isoform X1 [Corapipo altera]

MNFEPAPFTTLQYYPDPCIQRFVETPSPFSWKESYYRSAMSQGSQPREFLSPEVIQHLWDFLEQPICSVQPIDLNFIDGPSENGSTNKIEISMDCVRVQDTELNDPMWPQYTNLGLLNSMDQQIQNGSSSTSPYNTEHAQNSVTAPSPYAQPSSTFDALSPSPAIPSNTDYPGPHSFDVSFQQSSTAKSATWTYSTELKKLYCQIAKTCPIQIKVMTPPPQGAVIRAMPVYKKAEHVTEVVKRCPNHELSREFNEGQIAPPSHLIRVEGNSHAQYVEDPITGRQSVLVPYEPPQVGTEFTTVLYNFMCNSSCVGGMNRRPILIIVTLETRDGQVLGRRCFEARICACPGRDRKADEDSIRKQQVSDSTKNGDGTKRPFRQGTHGIQMTSIKKRRSPDDELLYLPVRGRETYEMLLKIKESLELMQYLPQHTIETYRQQQQQQHQHLLQKQTSMQSQSSYGSNSPPLSKMNSMNKLPSVSQLINPQQRNALTPTTIPDSMGTNIPMMGTHMAMTGDMNGLSPTQALPPPLSMPSTSHCTPPPPYPSDCSIVSFLARLGCSSCVDYFTTQGLTTIYQIEHYSMDDLVSLKIPEQFRHAIWKGILDHRQLHDFSSPPHLLRTPSGASTVSVGSSETRGERVIDAVRFTLRQTISFPPRDEWNDFNFDMDARRNKQQRIKEEGE

>XP_030136196.1 tumor protein 63 isoform X1 [Taeniopygia guttata]

MHDHSSMKSGLRSLSPRRGWKEMNFEAAPFTTLPYYPDPCIQRFVETPSPFSWKESYYRSAMSQSSQPREFLSPEVIQHIWDFLEQPICSVQPIDLNFIDGPSEDGSTNKIEISMDCVRVQDTELNDPMWPQYTNLGLLNSMDQQIQNGSSSTSPYNTEHAQNSVTAPSPYAQPSSTFDALSPSPAIPSNTDYPGPHSFDVSFQQSSTAKSATWTYSTELKKLYCQIAKTCPIQIKVMTPPPQGAVIRAMPVYKKAEHVTEVVKRCPNHELSREFNEGQIAPPSHLIRVEGNSHAQYVEDPITGRQSVLVPYEPPQVGTEFTTVLYNFMCNSSCVGGMNRRPILIIVTLETRDGQVLGRRCFEARICACPGRDRKADEDSIRKQQVSDSTKNGDGTKRPFRQGTHGIQMTSIKKRRSPDDELLYLPVRGRETYEMLLKIKESLELMQFLPQHTIETYRQQQQQQHQHLLQKQTSMQSQSSYGSNSPPLSKMNSMNKLPSVSQLINPQQRNALTPTTIPDSMGTNIPMMGTHMAMTGDMNGLSPTQALPPPLSMPSTSHCTPPPPYPSDCSIVSFLARLGCSSCVDYFTTQGLTTIYQIEHYSMDDLVSLKIPEQFRHAIWKGILDHRQLHDFSSPPHLLRTPSGASTVSVGSSETRGERVIDAVRFTLRQTISFPPRDEWNDFNFDMDARRNKQQRIKEEGE

>NXB02517.1 P63 protein [Cnemophilus loriae]

MNFEAAPFTTLPYYPDPCIQRFVETPSPFSWKESYYRSAMSQSSQPREFLSPEVIQHIWDFLEQPICSVQPIDLNFIDGPSEDGSTNKIEISMDCVRVQDTELNDPMWPQYTNLGLLNSMDQQIQNGSSSTSPYNTEHAQNSVTAPSPYAQPSSTFDALSPSPAIPSNTDYPGPHSFDVSFQQSSTAKSATWTYSTELKKLYCQIAKTCPIQIKVMTPPPQGAVIRAMPVYKKAEHVTEVVKRCPNHELSREFNEGQIAPPSHLIRVEGNSHAQYVEDPITGRQSVLVPYEPPQVGTEFTTVLYNFMCNSSCVGGMNRRPILIIVTLETRDGQVLGRRCFEARICACPGRDRKADEDSIRKQQVSDSTKNGDGTKRPFRQGTHGIQMTSIKKRRSPDDELLYLPVRGRETYEMLLKIKESLELMQYLPQHTIETYRQQQQQQHQHLLQKQTSMQSQSSYGSSSPPLSKMNSMNKLPSVSQLINPQQRNALTPTTIPDSMGTNIPMMGTHMAMTGDMNGLSPTQALPPPLSMPSTSHCTPPPPYPSDCSIVSFLARLGCSSCVDYFTTQGLTTIYQIEHYSMDDLVSLKIPEQFRHAIWKGILDHRQLHDFSSPPHLLRTPSGASTVSVGSSETRGERVIDAVRFTLRQTISFPPRDEWNDFNFDMDARRNKQQRIKEEGE

>XP_038002084.1 tumor protein 63 isoform X1 [Motacilla alba alba]

MHDHSSMKSGLRSLSPRRGWKEMNFEAAPFTTLPYYPDPCIPRFVETPSPFSWKESYYRSAMSQNSQPREFLSPEVIQHIWDFLEQPICSVQPIDLNFIDGPSEDGSTNKIEISMDCVRVQDTELNDPMWPQYTNLGLLNSMDQQIQNGSSSTSPYNTEHAQNSVTAPSPYAQPSSTFDALSPSPAIPSNTDYPGPHSFDVSFQQSSTAKSATWTYSTELKKLYCQIAKTCPIQIKVMTPPPQGAVIRAMPVYKKAEHVTEVVKRCPNHELSREFNEGQIAPPSHLIRVEGNSHAQYVEDPITGRQSVLVPYEPPQVGTEFTTVLYNFMCNSSCVGGMNRRPILIIVTLETRDGQVLGRRCFEARICACPGRDRKADEDSIRKQQVSDSTKNGDGTKRPFRQGTHGIQMTSIKKRRSPDDELLYLPVRGRETYEMLLKIKESLELMQFLPQHTIETYRQQQQQQHQHLLQKQTSMQSQSSYGSNSPPLSKMNSMNKLPSVSQLINPQQRNALTPTTIPDSMGTNIPMMSTHMAMTGDMNGLSPTQALPPPLSMPSTSHCTPPPPYPSDCSIVSFLARLGCSSCVDYFTTQGLTTIYQIEHYSMDDLVSLKIPEQFRHAIWKGILDHRQLHDFSSPPHLLRTPSGASTVSVGSSETRGERVIDAVRFTLRQTISFPPRDEWNDFNFDMDARRNKQQRIKEEGE

>NXM39090.1 P63 protein [Gymnorhina tibicen]

MNFEAAPFTTLPYYPDPCIQRFVETPSPFSWKESYYRSAMSQSSQSREFLSPEVIQHIWDFLEQPICSVQPIDLNFIDGPSEDGSTNKIEISMDCVRVQDTELNDPMWPQYTNLGLLNSMDQQIQNGSSSTSPYNTEHAQNSVTAPSPYAQPSSTFDALSPSPAIPSNTDYPGPHSFDVSFQQSSTAKSATWTYSTELKKLYCQIAKTCPIQIKVMTPPPQGAVIRAMPVYKKAEHVTEVVKRCPNHELSREFNEGQIAPPSHLIRVEGNSHAQYVEDPITGRQSVLVPYEPPQVGTEFTTVLYNFMCNSSCVGGMNRRPILIIVTLETRDGQVLGRRCFEARICACPGRDRKADEDSIRKQQVSDSTKNGDGTKRPFRQGTHGIQMTSIKKRRSPDDELLYLPVRGRETYEMLLKIKESLELMQYLPQHTIETYRQQQQQQHQHLLQKQTSMQSQSSYGSNSPPLSKMNSMNKLPSVSQLINPQQRNALTPTTIPDSMGTNIPMMGTHMAMTGDMNGLSPTQALPPPLSMPSTSHCTPPPPYPSDCSIVSFLARLGCSSCVDYFTTQGLTTIYQIEHYSMDDLVSLKIPEQFRHAIWKGILDHRQLHDFSSPPHLLRTPSGASTVSVGSSETRGERVIDAVRFTLRQTISFPPRDEWNDFNFDMDARRNKQQRIKEEGE

>NXM52023.1 P63 protein [Illadopsis cleaveri]

MNFEAAPFTTLPYYPDPCIQRFVETPSPFSWKESYYRSTMSQSSQPREFLSPEVIQHIWDFLEQPICSVQPIDLNFIDGPSEDGSTNKIEISMDCVRVQDTELSDPMWPQYTNLGLLNSMDQQIQNGSSSTSPYNTEHAQNSVTAPSPYAQPSSTFDALSPSPAIPSNTDYPGPHSFDVSFQQSSTAKSATWTYSTELKKLYCQIAKTCPIQIKVMTPPPQGAVIRAMPVYKKAEHVTEVVKRCPNHELSREFNEGQIAPPSHLIRVEGNSHAQYVEDPITGRQSVLVPYEPPQVGTEFTTVLYNFMCNSSCVGGMNRRPILIIVTLETRDGQVLGRRCFEARICACPGRDRKADEDSIRKQQVSDSTKNGDGTKRPFRQGTHGIQMTSIKKRRSPDDELLYLPVRGRETYEMLLKIKESLELMQYLPQHTIETYRQQQQQQHQHLLQKQTSMQSQSSYGSNSPPLSKMNSMNKLPSVSQLINPQQRNALTPTTIPDSMGTNIPMMGTHMAMTGDMNGLSPTQALPPPLSMPSTSHCTPPPPYPSDCSIVSFLARLGCSSCVDYFTTQGLTTIYQIEHYSMDDLVSLKIPEQFRHAIWKGILDHRQLHDFSSPPHLLRTPSGASTVSVGSGETRGERVIDAVRFTLRQTISFPPRDEWNDFNFDMDARRNKQQRIKEEGE

>NXO90484.1 P63 protein [Certhia brachydactyla]

MNFEAAPFTTLPYYPDPCIQRFVETPSPFSWKESYYRSAMSQSSQPREYLSPEVIQHIWDFLEQPICSVQPIDLNFIDGPSEDGSTNKIEISMDCVRVQDTELNDPMWPQYTNLGLLNSMDQQIQNGSSSTSPYNTEHAQNSVTAPSPYAQPSSTFDALSPSPAIPSNTDYPGPHSFDVSFQQSSTAKSATWTYSTELKKLYCQIAKTCPIQIKVMTPPPQGAVIRAMPVYKKAEHVTEVVKRCPNHELSREFNEGQIAPPSHLIRVEGNSHAQYVEDPITGRQSVLVPYEPPQVGTEFTTVLYNFMCNSSCVGGMNRRPILIIVTLETRDGQVLGRRCFEARICACPGRDRKADEDSIRKQQVSDSTKNGDGTKRPFRQGTHGIQMTSIKKRRSPDDELLYLPVRGRETYEMLLKIKESLELMQYLPQHTIETYRQQQQQQHQHLLQKQTSMQSQSSYGSNSPPLSKMNSMNKLPSVSQLINPQQRNALTPTTIPDSMGTNIPMMGTHMAMTGDMNGLSPTQALPPPLSMPSTSHCTPPPPYPSDCSIVSFLARLGCSSCVDYFTTQGLTTIYQIEHYSMDDLVSLKIPEQFRHAIWKGILDHRQLHEFSSPPHLLRTPSGASTVSVGSSETRGERVIDAVRFTLRQTISFPPRDEWNDFNFDMDARRNKQQRIKEEGE

>XP_027541515.1 tumor protein 63 isoform X1 [Neopelma chrysocephalum]

MNFEPAPFTTLQYYPDPCIQRFVETPSPFSWKESYYRSAMSQGSQPREFLSPEVIQHLWDFLEQPICSVQPIDLNFIDGPSENGSTNKIEISMDCVRVQDTELNDPMWPQYTNLGLLNSMDQQIQNGSSSTSPYNTEHAQNSVTAPSPYAQPSSTFDALSPSPAIPSNTDYPGPHSFDVSFQQSSTAKSATWTYSTELKKLYCQIAKTCPIQIKVMTPPPQGAVIRAMPVYKKAEHVTEVVKRCPNHELSREFNEGQIAPPSHLIRVEGNSHAQYVEDPITGRQSVLVPYEPPQVGTEFTTVLYNFMCNSSCVGGMNRRPILIIVTLETRDGQVLGRRCFEARICACPGRDRKADEDSIRKQQVSDSTKNGDGTKRPFRQGTHGIQMTSIKKRRSPDDELLYLPVRGRETYEMLLKIKESLELMQYLPQHTIETYRQQQQQQHQHLLQKQTSMQSQSSYGSNSPPLSKMNSMNKLPSVSQLMNPQQRNALTPTTIPDSMGTNIPMMGTHMAMTGDMNGLSPTQALPPPLSMPSTSHCTPPPPYPSDCSIVSFLARLGCSSCVDYFTTQGLTTIYQIEHYSMDDLVSLKIPEQFRHAIWKGILDHRQLHDFSSPPHLLRTPSGASTVSVGSSETRGERVIDAVRFTLRQTISFPPRDEWNDFNFDMDARRNKQQRIKEEGE

>XP_041323778.1 tumor protein 63 isoform X1 [Pyrgilauda ruficollis]

MHGHSSMKSGLRSLSPRRGWKEMNFEAAPFTTLPYYPDPCIPRFVETPSPFSWKESYYHSAMSQNSQPREFLSPEVIQHIWDFLEQPICSVQPIDLNFIDGPSEDGSTNKIEISMDCVRVQDTELNDPMWPQYTNLGLLNSMDQQIQNGSSSTSPYNTEHAQNSVTAPSPYAQPSSTFDALSPSPAIPSNTDYPGPHSFDVSFQQSSTAKSATWTYSTELKKLYCQIAKTCPIQIKVMTPPPQGAVIRAMPVYKKAEHVTEVVKRCPNHELSREFNEGQIAPPSHLIRVEGNSHAQYVEDPITGRQSVLVPYEPPQVGTEFTTVLYNFMCNSSCVGGMNRRPILIIVTLETRDGQVLGRRCFEARICACPGRDRKADEDSIRKQQVSDSTKNGDGTKRPFRQGTHGIQMTSIKKRRSPDDELLYLPVRGRETYEMLLKIKESLELMQFLPQHTIETYRQQQQQQHQHLLQKQTSMQSQSSYGSNSPPLSKMNSMNKLPSVSQLINPQQRNALTPTTIPDSMGTNIPMMGTHMAMTGDMNGLSPTQALPPPLSMPSTSHCTPPPPYPSDCSIVSFLARLGCSSCVDYFTTQGLTTIYQIEHYSMDDLVSLKIPEQFRHAIWKGILDHRQLHDFSSPPHLLRTPSGASTVSVGSSETRGERVIDAVRFTLRQTISFPPRDEWNDFNFDMDARRNKQQRIKEEGE

>XP_041261735.1 tumor protein 63 isoform X1 [Onychostruthus taczanowskii]

MLGLSPRRGWKEMNFEAAPFTTLPYYPDPCIPRFVETPSTFSWKESYYHSAMSQNSQPREFLSPEVIQHIWDFLEQPICSVQPIDLNFIDGPSEDGSTNKIEISMDCVRVQDTELNDPMWPQYTNLGLLNSMDQQIQNGSSSTSPYNTEHAQNSVTAPSPYAQPSSTFDALSPSPAIPSNTDYPGPHSFDVSFQQSSTAKSATWTYSTELKKLYCQIAKTCPIQIKVMTPPPQGAVIRAMPVYKKAEHVTEVVKRCPNHELSREFNEGQIAPPSHLIRVEGNSHAQYVEDPITGRQSVLVPYEPPQVGTEFTTVLYNFMCNSSCVGGMNRRPILIIVTLETRDGQVLGRRCFEARICACPGRDRKADEDSIRKQQVSDSTKNGDGTKRPFRQGTHGIQMTSIKKRRSPDDELLYLPVRGRETYEMLLKIKESLELMQFLPQHTIETYRQQQQQQHQHLLQKQTSMQSQSSYGSNSPPLSKMNSMNKLPSVSQLINPQQRNALTPTTIPDSMGTNIPMMGTHMAMTGDMNGLSPTQALPPPLSMPSTSHCTPPPPYPSDCSIVSFLARLGCSSCVDYFTTQGLTTIYQIEHYSMDDLVSLKIPEQFRHAIWKGILDHRQLHDFSSPPHLLRTPSGASTVSVGSSETRGERVIDAVRFTLRQTISFPPRDEWNDFNFDMDARRNKQQRIKEEGE

>XP_033372679.1 tumor protein 63 isoform X5 [Parus major]

MNFEAAPFTTLPYYPDPCIQRFVETPSPFSWKESYCRSAMSQSSQPREFLSPEVIQHIWDFLEQPICSVQPIDLNFIDGPSEDGSTNKIEISMDCVRVQDTELNDPMWPQYTNLGLLNSMDQQIQNGSSSTSPYNTEHAQNSVTAPSPYAQPSSTFDALSPSPAIPSNTDYPGPHSFDVSFQQSSTAKSATWTYSTELKKLYCQIAKTCPIQIKVMTPPPQGAVIRAMPVYKKAEHVTEVVKRCPNHELSREFNEGQIAPPSHLIRVEGNSHAQYVEDPITGRQSVLVPYEPPQVGTEFTTVLYNFMCNSSCVGGMNRRPILIIVTLETRDGQVLGRRCFEARICACPGRDRKADEDSIRKQQVSDSTKNGDGTKRPFRQGTHGIQMTSIKKRRSPDDELLYLPVRGRETYEMLLKIKESLELMQYLPQHTIETYRQQQQQQHQHLLQKQTSMQSQSSYGSNSPPLSKMNSMNKLPSVSQLINPQQRNALTPTTIPDSMGTNIPMMGTHMAMTGDMNGLSPTQALPPPLSMPSTSHCTPPPPYPSDCSIVSFLARLGCSSCVDYFTTQGLTTIYQIEHYSMDDLVSLKIPEQFRHAIWKGILDHRQLHDFSSPPHLLRTPSGASTVSVGSSETRGERVIDAVRFTLRQTISFPPRDEWNDFNFDMDARRNKQQRIKEEGE

>NWH98652.1 P63 protein [Tichodroma muraria]

MNFEAAPFTTLPYYPDPCIQRFVETPSPFSWKESYYRSTMSQSSQPREFLSPEVIQHIWDFLEQPICSVQPIDLNFIDGPSEDGSTNKIEISMDCVRVQDTELNDPMWPQYTNLGLLNSMDQQIQNGSSSTSPYNTEHAQNSVTAPSPYAQPSSTFDALSPSPAIPSNTDYPGPHSFDVSFQQSSTAKSATWTYSTELKKLYCQIAKTCPIQIKVMTPPPQGAVIRAMPVYKKAEHVTEVVKRCPNHELSREFNEGQIAPPSHLIRVEGNSHAQYVEDPITGRQSVLVPYEPPQVGTEFTTVLYNFMCNSSCVGGMNRRPILIIVTLETRDGQVLGRRCFEARICACPGRDRKADEDSIRKQQVSDSTKNGDGTKRPFRQGTHGIQMTSIKKRRSPDDELLYLPVRGRETYEMLLKIKESLELMQYLPQHTIETYRQQQQQQHQHLLQKQTSMQSQSSYGSSSPPLSKMNSMNKLPSVSQLINPQQRNALTPTTIPDSMGTNIPMMGTHMAMTGDMNGLSPTQALPPPLSMPSTSHCTPPPPYPSDCSIVSFLARLGCSSCVDYFTTQGLTTIYQIEHYSMDDLVSLKIPEQFRHAIWKGILDHRQLHDFSSPPHLLRTPSGASTVSVGSSETRGERVIDAVRFTLRQTISFPPRDEWNDFNFDMDARRNKQQRIKEEGE

>NXK37261.1 P63 protein [Piprites chloris]

MNFEPAPFTTLQYYPDPCIHRFVETPSPFSWKESYYRSAMSQGSQPREFLSPEVIQHLWDFLEQPICSVQPIDLNFIDGPSENGSTNKIEISMDCVRVQDTELNDPMWPQYTNLGLLNSMDQQIQNGSSSTSPYNTEHTQNSVTAPSPYAQPSSTFDALSPSPAIPSNTDYPGPHSFDVSFQQSSTAKSATWTYSTELKKLYCQIAKTCPIQIKVMTPPPQGAVIRAMPVYKKAEHVTEVVKRCPNHELSREFNEGQIAPPSHLIRVEGNSHAQYVEDPITGRQSVLVPYEPPQVGTEFTTVLYNFMCNSSCVGGMNRRPILIIVTLETRDGQVLGRRCFEARICACPGRDRKADEDSIRKQQVSDSTKNGDGTKRPFRQGTHGIQMTSIKKRRSPDDELLYLPVRGRETYEMLLKIKESLELMQYLPQHTIETYRQQQQQQHQHLLQKQTSMQSQSSYGSNSPPLSKMNSMNKLPSVSQLINPQQRNALTPTTIPDSMGTSIPMMGTHMAMTGDMNGLSPTQALPPPLSMPSTSHCTPPPPYPSDCSIVSFLARLGCSSCVDYFTTQGLTTIYQIEHYSMDDLVSLKIPEQFRHAIWKGILDHRQLHDFSSPPHLLRTPSGASTVSVGSSETRGERVIDAVRFTLRQTISFPPRDEWNDFNFDMDARRNKQQRIKEEGE

>XP_026647914.1 tumor protein 63 isoform X1 [Zonotrichia albicollis]

MHNPSSMKSGWRSLSPGRGWKEMNFEAAPFSTLPYYPDACIPRFVETPSPFPWKESYYRSAMSQSSQPREFLSPEVIQHIWDFLEQPICSVQPIDLNFIDGPLEDGSTNKIEISMDCVRVQDTELNDPMWPQYTNLGLLNSMDQQIQNGSSSTSPYNTEHAQNSVTAPSPYAQPSSTFDALSPSPAIPSNTDYPGPHSFDVSFQQSSTAKSATWTYSTELKKLYCQIAKTCPIQIKVMTPPPQGAVIRAMPVYKKAEHVTEVVKRCPNHELSREFNEGQIAPPSHLIRVEGNSHAQYVEDPITGRQSVLVPYEPPQVGTEFTTVLYNFMCNSSCVGGMNRRPILIIVTLETRDGQVLGRRCFEARICACPGRDRKADEDSIRKQQVSDSTKNGDGTKRPFRQGTHGIQMTSIKKRRSPDDELLYLPVRGRETYEMLLKIKESLELMQFLPQHTIETYRQQQQQQHQHLLQKQTSMQSQSSYGSNSPPLSKMNSMNKLPSVSQLINPQQRNALTPTTIPDSMGTNIPMMGTHMAMTGDMNGLSPTQALPPPLSMPSTSHCTPPPPYPSDCSIVSFLARLGCSSCVDYFTTQGLTTIYQIEHYSMDDLVSLKIPEQFRHAIWKGILDHRQLHDFSSPPHLLRTPSGASTVSVGSSETRGERVIDAVRFTLRQTISFPPRDEWNDFNFDMDARRNKQQRIKEEGE

>NWI25731.1 P63 protein [Sula dactylatra]

MNFEPAPFTTLQYYPDPCIQRHFSWKESYYRSAMSHGSQPREFLSPEVLQHIWDFLEQPICSVQPIDLNFIDDPSENGPTNKIEISMDCVRVQDTELNDPMWPQYTNLGLLNSMDQQIQNGSSSTSPYNTEHAQNSVTAPSPYAQPSSTFDALSPSPAIPSNTDYPGPHSFDVSFQQSSTAKSATWTYSTELKKLYCQIAKTCPIQIKVMTPPPQGAVIRAMPVYKKAEHVTEVVKRCPNHELSREFNEGQIAPPSHLIRVEGNSHAQYVEDPITGRQSVLVPYEPPQVGTEFTTVLYNFMCNSSCVGGMNRRPILIIVTLETRDGQVLGRRCFEARICACPGRDRKADEDSIRKQQVSDSTKNGDGTKRPFRQGTHGIQMTSIKKRRSPDDELLYLPVRGRETYEMLLKIKESLELMQYLPQHTIETYRQQQQQQHQHLLQKQTSMQSQSSYGSNSPPLSKMNSMNKLPSVSQLINPQQRNALTPTTIPDGMGTNIPMMGTHMAMTGDMNGLSPTQALPPPLSMPSTSHCTPPPPYPTDCSIVSFLARLGCSSCVDYFTTQGLTTIYQIEHYSMDDLVSLKIPEQFRHAIWKGILDHRQLHDFSSPPHLLRTPSGASTVSVGSSETRGERVIDAVRFTLRQTISFPPRDEWNDFNFDMDARRNKQQRIKEEGE

>NXI13578.1 P63 protein [Irena cyanogastra]

MNFEAAPFTTLPYYPDPCMQRFVETPSPFSWKESYYRSAMSQNSQPREFLSPEVIQHLWDFLEQPICSVQPIDLNFIDGPSEDGSTNKIEISMDCVRVQDTELNDPMWPQYTNLGLLNSMDQQIQNGSSSTSPYNTEHAQNSVTAPSPYAQPSSTFDALSPSPAIPSNTDYPGPHSFDVSFQQSSTAKSATWTYSTELKKLYCQIAKTCPIQIKVMTPPPQGAVIRAMPVYKKAEHVTEVVKRCPNHELSREFNEGQIAPPSHLIRVEGNSHAQYVEDPITGRQSVLVPYEPPQVGTEFTTVLYNFMCNSSCVGGMNRRPILIIVTLETRDGQVLGRRCFEARICACPGRDRKADEDSIRKQQVSDSTKNGDGTKRPFRQGTHGIQMTSIKKRRSPDDELLYLPVRGRETYEMLLKIKESLELMQFLPQHTIETYRQQQQQQHQHLLQKQTSMQSQSSYGSSSPPLSKMNSMNKLPSVSQLINPQQRNALTPTTIPDSMGTNIPMMGTHMAMTGDMNGLSPTQALPPPLSMPSTSHCTPPPPYPSDCSIVSFLARLGCSSCVDYFTTQGLTTIYQIEHYSMDDLVSLKIPEQFRHAIWKGILDHRQLHDFSSPPHLLRTPSGASTVSVGSSETRGERVIDAVRFTLRQTISFPPRDEWNDFNFDMDARRNKQQRIKEEGE

>NWU19604.1 P63 protein [Platysteira castanea]

MNFEAAPFTTLPYYPDPCIQRFVETPSPFSWKESYYRSTMSQSSQPREFLSPEVIQHIWDFLEQPICSVQPIDLNFIDGPSEDGSTNKIEISMDCVRVQDTELNDPMWPQYTNLGLLNSMDQQIQNGSSSTSPYNTEHAQNSVTAPSPYAQPSSTFDALSPSPAIPSNTDYPGPHSFDVSFQQSSTAKSATWTYSTELKKLYCQIAKTCPIQIKVMTPPPQGAVIRAMPVYKKAEHVTEVVKRCPNHELSREFNEGQIAPPSHLIRVEGNSHAQYVEDPITGRQSVLVPYEPPQVGTEFTTVLYNFMCNSSCVGGMNRRPILIIVTLETRDGQVLGRRCFEARICACPGRDRKADEDSIRKQQVSDSTKNGDGTKRPFRQGTHGIQMTSIKKRRSPDDELLYLPVRGRETYEMLLKIKESLELMQYLPQHTIETYRQQQQQQHQPLLQKQTSMQSQSSYGSSSPPLSKMNSMNKLPSVSQLINPQQRNALTPTTIPDSMGTNIPMMGTHMAMTGDMNGLSPTQALPPPLSMPSTSHCTPPPPYPSDCSIVSFLARLGCSSCVDYFTTQGLTTIYQIEHYSMDDLVSLKIPEQFRHAIWKGILDHRQLHDFSSPPHLLRTPSGASTVSVGSSETRGERVIDAVRFTLRQTISFPPRDEWNDFNFDMDARRNKQQRIKEEGE

>OPJ85615.1 tumor protein 63 isoform A [Patagioenas fasciata monilis]

MNFEPAPFTTLQYYPDPCIQRFVETPSQFSWKESYYRSAMSQNSQPREFLSPEVLQHIWDFLEQPICSVQPIDLNFIDGPSENGPTNKIEISMDCVRVQDTELNDPMWPQYTNLGLLNSMDQQIQNGSSSTSPYNTEHAQNSVTAPSPYAQPSSTFDALSPSPAIPSNTDYPGPHSFDVSFQQSSTAKSATWTYSTELKKLYCQIAKTCPIQIKVMTPPPQGAVIRAMPVYKKAEHVTEVVKRCPNHELSREFNEGQIAPPSHLIRVEGNSHAQYVEDPITGRQSVLVPYEPPQVGTEFTTVLYNFMCNSSCVGGMNRRPILIIVTLETRDGQVLGRRCFEARICACPGRDRKADEDSIRKQQVSDSTKNGDAFRQGTHGIQMTSIKKRRSPDDELLYLPVRGRETYEMLLKIKESLELMQYLPQHTIETYRQQQQQQHQHLLQKQTSMQSQSSYGSNSPPLSKMNSMNKLPSVSQLINPQQRNALTPTTIPDGMGTNIPMMGTHMAMTGDMNGLSPTQALPPPLSMPSTSHCTPPPPYPTDCSIVSFLARLGCSSCVDYFTTQGLTTIYQIEHYSMDDLVSLKIPEQFRHAIWKGILDHRQLHDFSSPPHLLRTPSGASTVSVGSSETRGERVIDAVRFTLRQTISFPPRDEWNDFNFDMDARRNKQQRIKEEGE

>XP_032924604.1 tumor protein 63 isoform X2 [Catharus ustulatus]

MNFEAAPFTTLPYYPDPCIQRFVETPSPFSWKESYYRSAMSQSSQPREFLSPEVIQHIWDFLEQPICSVQPIDLNFIDGPSEDGSTNKIEISMDCVRVQDTELNDPMWPQYTNLGLLNSMDQQIQNGSSSTSPYNTEHAQNSVTAPSPYAQPSSTFDALSPSPAIPSNTDYPGPHSFDVSFQQSSTAKSATWTYSTELKKLYCQIAKTCPIQIKVMTPPPQGAVIRAMPVYKKAEHVTEVVKRCPNHELSREFNEGQIAPPSHLIRVEGNSHAQYVEDPITGRQSVLVPYEPPQVGTEFTTVLYNFMCNSSCVGGMNRRPILIIVTLETRDGQVLGRRCFEARICACPGRDRKADEDSIRKQQVSDSTKNGDGTKRPFRQGTHGIQMTSIKKRRSPDDELLYLPVRGRETYEMLLKIKESLELMQYLPQHTIETYRQQQQQQHQHLLQKQTSMQSQSSYGSNSPPLSKMNSMNKLPSVSQLINPQQRNALTPTTIPDSMGTNIPMMGTHMAMTGDMNGLSPTQALPPPLSMPSTSHCTPPPPYPSDCSIVSFLARLGCSSCVDYFTTQGLTTIYQIEHYSMDDLVSLKIPEQFRHAIWKGILDHRQLHDFSPPHLLRTPSGASTVSVGSSETRGERVIDAVRFTLRQTISFPPRDDWNDFNFDMDARRNKQQRIKEEGE

>NXO21751.1 P63 protein [Cisticola juncidis]

MNFEAAPFSTLPCYPDACIQRFVETPSPFSWKESYYRSAMSQSSQPREFLSPEVIQHIWDFLEQPICSVQPIDLNFIDGPSEDGSTNKIEISMDCVRVQDTELNDPMWPQYTNLGLLNSMDQQIQNGSSSTSPYNTEHAQNSVTAPSPYAQPSSTFDALSPSPAIPSNTDYPGPHSFDVSFQQSSTAKSATWTYSTELKKLYCQIAKTCPIQIKVMTPPPQGAVIRAMPVYKKAEHVTEVVKRCPNHELSREFNEGQIAPPSHLIRVEGNSHAQYVEDPITGRQSVLVPYEPPQVGTEFTTVLYNFMCNSSCVGGMNRRPILIIVTLETRDGQVLGRRCFEARICACPGRDRKADEDSIRKQQVSDSTKNGDGTKRPFRQGTHGIQMTSIKKRRSPDDELLYLPVRGRETYEMLLKIKESLELMQYLPQHTIETYRQQQQQQHQHLLQKQTSMQSQSSYGSSSPPLSKMNSMNKLPSVSQLINPQQRNALTPTTIPDSMGTNIPMMGTHMAMTGDMNGLSPTQALPPPLSMPSTSHCTPPPPYPSDCSIVSFLARLGCSSCVDYFTTQGLTTIYQIEHYSMDDLVSLKIPEQFRHAIWKGILDHRQLHDFSSPPHLLRTPSGASTVSVGSGEARGERVIDAVRFTLRQTISFPPRDEWNDFNFDMDARRNKQQRIKEEGE

>NXI88257.1 P63 protein [Rhipidura dahli]

MNFEAAPFTTLPYYPDPCIQRFVETPSPFSWKESYCRSAMSQSSQPRESLSPEVFQHIWDFLEQPICSVQPIDLNFIDGPSEDGSTNKIEISMDCVRVQDTELNDPMWPQYTNLGLLNSMDQQIQNGSSSTSPYNTEHAQNSVTAPSPYAQPSSTFDALSPSPAIPSNTDYPGPHSFDVSFQQSSTAKSATWTYSTELKKLYCQIAKTCPIQIKVMTPPPQGAVIRAMPVYKKAEHVTEVVKRCPNHELSREFNEGQIAPPSHLIRVEGNSHAQYVEDPITGRQSVLVPYEPPQVGTEFTTVLYNFMCNSSCVGGMNRRPILIIVTLETRDGQVLGRRCFEARICACPGRDRKADEDSIRKQQVSDSTKNGDGTKRPFRQGTHGIQMTSIKKRRSPDDELLYLPVRGRETYEMLLKIKESLELMQYLPQHTIETYRQQQQQQHQHLLQKQTSMQSQSSYGSSSPPLSKMNSMNKLPSVSQLINPQQRNALTPTTIPDSMGTNIPMMGTHMAMTGDMNGLSPTQALPPPLSMPSTSHCTPPPPYPSDCSIVSFLARLGCSSCVDYFTTQGLTTIYQIEHYSMDDLVSLKIPEQFRHAIWKGILDHRQLHDFSSPPHLLRTPSGASTVSVGSSETRGERVIDAVRFTLRQTISFPPRDEWNDFNFDMDARRNKQQRIKEEGE

>XP_024058148.1 tumor protein 63 isoform X1 [Terrapene carolina triunguis]

MNFEPAPYTTLQYYPDPCMQRFVETPGHFSWTESYYRSTMSQSSQTREFLSPEVFQQIWEFLEQPICSVQPINLNFMDDPSDNGPTNKIEISMDCVRMQDADGSDPMWPQYTNLGLLNSMDQQIQNGSSSTSPYNTEHAQNSVTAPSPYAQPSSTFDALSPSPAIPSNTDYPGPHSFDVSFQQSSTAKSATWTYSTELKKLYCQIAKTCPIQVKVMTPPPQGAIIRAMPVYKKAEHVTEVVKRCPNHELSREFNEGQIAPPSHLIRVEGNSHAQYVEDPITGRQSVLVPYEPPQVGTEFTTVLYNFMCNSSCVGGMNRRPILIIVTLETRDGQVLGRRCFEARICACPGRDRKADEDSIRKQQVSDSTKNGDGTKRPFRQSTHGIQMTSIKKRRSPDDELLYLPVRGRETYEMLLKIKESLELMQYLPQHTIETYRQQQQQQHQHLLQKQTSMQSQSSYGSNSPPLSKMNSMNKLPSVSQLINPQQRNALTPTAIPDGMGTNIPMMGTHMAMTGDMNGLSPTQALPPSLSMPSTSHCTPPPPYPTDCSIVSFLARLGCSSCVDYFTTQGLTTIYQIEHYSMDDLVSLKIPEQFRHAIWKGILDHRQLHDFSSPPHLLRTPSGASTVSVGSSETRGERVIDAVRFTLRQTISFPPRDEWNDFNFDMDARRNKQQRIKEEGE

>NXJ09015.1 P63 protein [Odontophorus gujanensis]

RFVETPSHFSWKESYYRSAMSQSSQPREFLSPEVIQHIWDFLEQPICSVQPIDLNFIDDPSENGPTNKIEISMDCVRLQDTELSDPMWPQYTNLGLLNSMDQQIQNGSSSTSPYNTEHAQNSVTAPSPYAQPSSTFDALSPSPAIPSNTDYPGPHSFDVSFQQSSTAKSATWTYSTELKKLYCQIAKTCPIQIKVMTPPPQGAVIRAMPVYKKAEHVTEVVKRCPNHELSREFNEGQIAPPSHLIRVEGNSHAQYVEDPITGRQSVLVPYEPPQVGTEFTTVLYNFMCNSSCVGGMNRRPILIIVTLETRDGQVLGRRCFEARICACPGRDRKADEDSIRKQQVSDSTKNGDGTKRPFRQGTHGIQMTSIKKRRSPDDELLYLPVRGRETYEMLLKIKESLELMQYLPQHTIETYRQQQQQQHQHLLQKQTSIQSQSSYGSNSPPLSKMNSMNKLPSVSQLINPQQRNALTPTTIPDGMGTNIPMMGTHMAMTSDMNGLSPTQALPPPLSMPSTSHCTPPPPYPTDCSIVSFLARLGCSSCVDYFTTQGLTTIYQIEHYSMDDLVSLKIPEQFRHAIWKGILDHRQLHDFSSPPHLLRTPSGASTVSVGSSETRGERVIDAVRFTLRQTISFPPRDEWNDFNFDMDARRNKQQRIKEEGE

>NXC38307.1 P63 protein [Penelope pileata]

PSFVRFVETPSHFSWKESYYRSAMSQSSQPREFLSPEVIQHIWDFLEQPICSVQPIDLNFIDDPSENGPTNKIEISMDCVRLQDTELSDPMWPQYTNLGLLNSMDQQIQNGSSSTSPYNTEHAQNSVTAPSPYAQPSSTFDALSPSPAIPSNTDYPGPHSFDVSFQQSSTAKSATWTYSTELKKLYCQIAKTCPIQIKVMTPPPQGAVIRAMPVYKKAEHVTEVVKRCPNHELSREFNEGQIAPPSHLIRVEGNSHAQYVEDPITGRQSVLVPYEPPQVGTEFTTVLYNFMCNSSCVGGMNRRPILIIVTLETRDGQVLGRRCFEARICACPGRDRKADEDSIRKQQVSDSTKNGDGTKRPFRQGTHGIQMTSIKKRRSPDDELLYLPVRGRETYEMLLKIKESLELMQYLPQHTIETYRQQQQQQHQHLLQKQTSIQSQSSYGSNSPPLSKMNSMNKLPSVSQLINPQQRNALTPTTIPDGMGTNIPMMGTHMAMTSDMNGLSPTQALPPPLSMPSTSHCTPPPPYPTDCSIVSFLARLGCSSCVDYFTTQGLTTIYQIEHYSMDDLVSLKIPEQFRHAIWKGILDHRQLHDFSSPPHLLRTPSGASTVSVGSSETRGERVIDAVRFTLRQTISFPPRDEWNDFNFDMDARRNKQQRIKEEGE

>XP_035400845.1 tumor protein 63 isoform X1 [Cygnus atratus]

MCESTHWKQAADLKRYWKNSLDIEKGMTWSLKKQTSFSVAWRLEQEAKSHIRFVETPSHFSWKESYYRSAMSQSSQPREFLSPEVIQHIWDFLEQPICSVQPIDLNFIDDPSENGPTNKIEISMDCVRLQDTELSDPMWPQYTNLGLLNSMDQQIQNGSSSTSPYNTEHTQNSVTAPSPYAQPSSTFDALSPSPAIPSNTDYPGPHSFDVSFQQSSTAKSATWTYSTELKKLYCQIAKTCPIQIKVLTPPPQGAVIRAMPVYKKAEHVTEVVKRCPNHELSREFNEGQIAPPSHLIRVEGNSHAQYVEDPITGRQSVLVPYEPPQVGTEFTTVLYNFMCNSSCVGGMNRRPILIIVTLETRDGQVLGRRCFEARICACPGRDRKADEDSIRKQQVSDSTKNGDGTKRPFRQGTHGIQMTSIKKRRSPDDELLYLPVRGRETYEMLLKIKESLELMQYLPQHTIETYRQQQQQQHQHLLQKQTSMQSQSSYGSNSPPLSKMNSMNKLPSVSQLINPQQRNALTPTTIPDGMGTNIPMMGTHMAMTGDMNGLSPTQALPPPLSMPSTSHCTPPPPYPTDCSIVSFLARLGCSSCVDYFTTQGLTTIYQIEHYSMDDLVSLKIPEQFRHAIWKGILDHRQLHDFSSPPHLLRTPSGASTVSVGSSETRGERVIDAVRFTLRQTISFPPRDEWNDFNFDMDARRNKQQRIKEEGE

>NXP72921.1 P63 protein [Ramphastos sulfuratus]

CFLRFVETPSHFSWKESYYRSAMSQSSQPREFLSPEVLQHIWDFLEQPICSVQPIDLNFIDDPSENGPTNKIEISMDCVRVQDTELNDPMWPQYTNLGLLNSMDQQIQNGSSSTSPYNTEHAQNSVTAPSPYAQPSSTFDALSPSPAIPSNTDYPGPHSFDVSFQQSSTAKSATWTYSTELKKLYCQIAKTCPIQIKVMTPPPQGAVIRAMPVYKKAEHVTEVVKRCPNHELSREFNEGQIAPPSHLIRVEGNSHAQYVEDPITGRQSVLVPYEPPQVGTEFTTVLYNFMCNSSCVGGMNRRPILIIVTLETRDGQVLGRRCFEARICACPGRDRKADEDSIRKQQVSDSTKNGDGTKRPFRQGTHGIQMTSIKKRRSPDDELLYLPVRGRETYEMLLKIKESLELMQYLPQHTIETYRQQQQQQHQHLLQKQTSLQSQSSYGSNSPPLGKMNSMNKLPSVSQLINPQQRNALTPTTIPDGMGTNIPMMGTHMAMTGDMNGLSPTQALPPPLSMPSTSHCTPPPPYPTDCSIVSFLARLGCSSCVDYFTTQGLTTIYQIEHYSMDDLVSLKIPEQFRHAIWKGILDHRQLHDFSSPPHLLRTPSGASTVSVGSSETRGERVIDAVRFTLRQTISFPPRDEWNDFNFDMDARRNKQQRIKEEGE

>NXL61753.1 P63 protein [Chordeiles acutipennis]

ISFCVCVFFSLFFFFFCFLRFVETPSHFSWKESYYRSAMSQSSQPREFLSPEVLQHIWDFLEQPICSVQPIDLNFIDDPSENGPTNKIEISMDCVRVQDTELNDPMWPQYTNLGLLNSMDQQIQNGSSSTSPYNTEHAQNSVTAPSPYAQPSSTFDALSPSPAIPSNTDYPGPHSFDVSFQQSSTAKSATWTYSTELKKLYCQIAKTCPIQIKVMTPPPQGAVIRAMPVYKKAEHVTEVVKRCPNHELSREFNEGQIAPPSHLIRVEGNSHAQYVEDPITGRQSVLVPYEPPQVGTEFTTVLYNFMCNSSCVGGMNRRPILIIVTLETRDGQVLGRRCFEARICACPGRDRKADEDSIRKQQVSDSTKNGDGTKRPFRQGTHGIQMTSIKKRRSPDDELLYLPVRGRETYEMLLKIKESLELMQYLPQHTIETYRQQQQQQHQHLLQKQTSMQSQSSYGSNSPPLSKMNSMNKLPSVSQLINPQQRNALTPTTIPDGMGTNIPMMGTHMAMTGDMNGLSPTQALPPPLSMPSTSHCTPPPPYPTDCSIVSFLARLGCSSCVDYFTTQGLTTIYQIEHYSMDDLVSLKIPEQFRHAIWKGILDHRQLHDFSSPPHLLRTPSGASTVSVGSSETRGERVIDAVRFTLRQTISFPPRDEWNDFNFDMDARRNKQQRIKEEGE

>NXK46272.1 P63 protein [Chauna torquata]

CFVRFVETPSHFSWKESYYRSAMSQSSQPREFLSPEVIQHIWDFLEQPICSVQPIDLNFIDDPSENGPTNKIEISMDCVRLQDTELSDPMWPQYTNLGLLNSMDQQIQNGSSSTSPYNTEHAQNSVTAPSPYAQPSSTFDALSPSPAIPSNTDYPGPHSFDVSFQQSSTAKSATWTYSTELKKLYCQIAKTCPIQIKVMTPPPQGAVIRAMPVYKKAEHVTEVVKRCPNHELSREFNEGQIAPPSHLIRVEGNSHAQYVEDPITGRQSVLVPYEPPQVGTEFTTVLYNFMCNSSCVGGMNRRPILIIVTLETRDGQVLGRRCFEARICACPGRDRKADEDSIRKQQVSDSTKNGDGTKRPFRQGTHGIQMTSIKKRRSPDDELLYLPVRGRETYEMLLKIKESLELMQYLPQHTIETYRQQQQQQHQHLLQKQTSMQSQTSYGSNSPPLGKMNSMNKLPSVSQLINPQQRNALTPTTIPDGMGTNIPMMGTHMAMTSDMNGLSPTQALPPPLSMPSTSHCTPPPPYPTDCSIVSFLARLGCSSCVDYFTTQGLTTIYQIEHYSMDDLVSLKIPEQFRHAIWKGILDHRQLHDFSSPPHLLRTPSGASTVSVGSSETRGERVIDAVRFTLRQTISFPPRDEWNDFNFDMDARRNKQQRIKEEGE

>NXA51119.1 P63 protein [Nothocercus julius]

CFLRFVETPSHFSWKESYYRSAMSQNSQPREFLSPEVLQHIWDFLEQPICSVQPIDLNFIDDPSENGPTNKIEISMDCVRVQDTELSDPMWPQYTNLGLLNSMDQQIQNGSSSTSPYNTEHAQNSVTAPSPYAQPSSTFDALSPSPAIPSNTDYPGPHSFDVSFQQSSTAKSATWTYSTELKKLYCQIAKTCPIQIKVMTPPPQGAVIRAMPVYKKAEHVTEVVKRCPNHELSREFNEGQIAPPSHLIRVEGNSHAQYVEDPITGRQSVLVPYEPPQVGTEFTTVLYNFMCNSSCVGGMNRRPILIIVTLETRDGQVLGRRCFEARICACPGRDRKADEDSIRKQQVSDSTKNGDGTKRPFRQGTHGIQMTSIKKRRSPDDELLYLPVRGRETYEMLLKIKESLELMQYLPQHTIETYRQQQQQQHQHLLQKQTSMQSQSSYGSNSPPLSKMNSMNKLPSVSQLINPQQRNALTPTTIPDGMGTNIPMMGTHMAMTSDMNGLSPTQALPPPLSMPSTSHCTPPPPYPTDCSIVSFLARLGCSSCVDYFTTQGLTTIYQIEHYSMDDLVSLKIPEQFRHAIWKGILDHRQLHDFSSPPHLLRTPSGASTVSVGSSETRGERVIDAVRFTLRQTISFPPRDEWNDFNFDMDARRNKQQRIKEEGE

>NWH27094.1 P63 protein [Grus americana]

CFLRFVETPSHFSWKESYYRSAMSQSSQPREFLSPEVLQHIWDFLEQPICSVQPIDLNFIDGPSENGPTNKIEISMDCVRVQDTELNDPMWPQYTNLGLLNSMDQQIQNGSSSTSPYNTEHAQNSVTAPSPYAQPSSTFDALSPSPAIPSNTDYPGPHSF

DVSFQQSSTAKSATWTYSTELKKLYCQIAKTCPIQIKVMTPPPQGAVIRAMPVYKKAEHVTEVVKRCPNHELSREFNEGQIAPPSHLIRVEGNSHAQYVEDPITGRQSVLVPYEPPQVGTEFTTVLYNFMCNSSCVGGMNRRPILIIVTLETRDGQVLGRRCFEARICACPGRDRKADEDSIRKQQVSDSTKNGDGTKRPFRQGTHGIQMTSIKKRRSPDDELLYLPVRGRETYEMLLKIKESLELMQYLPQHTIETYRQQQQQQHQHLLQKQTSMQSQSSYGSNSPPLSKMNSMNKLPSVSQLINPQQRNALTPTTIPDGMGTNIPMMGTHMAMTGDMNGLSPTQALPPPLSMPSTSHCTPPPPYPTDCSIVSFLARLGCSSCVDYFTTQGLTTIYQIEHYSMDDLVSLKIPEQFRHAIWKGILDHRQLHDFSSPPHLLRTPSGASTVSVGSSETRGERVIDAVRFTLRQTISFPPRDEWNDFNFDMDARRNKQQRIKEEGE

>NWZ32253.1 P63 protein [Asarcornis scutulata]

VFFPFFFLFFYFVRFVETPSHFSWKESYYRSAMSQSSQPREFLSPEVIQHIWDFLEQPICSVQPIDLNFIDDPSENGPTNKIEISMDCVRLQDTELSDPMWPQYTNLGLLNSMDQQIQNGSSSTSPYNTEHTQNSVTAPSPYAQPSSTFDALSPSPAIPSNTDYPGPHSFDVSFQQSSTAKSATWTYSTELKKLYCQIAKTCPIQIKVLTPPPQGAVIRAMPVYKKAEHVTEVVKRCPNHELSREFNEGQIAPPSHLIRVEGNSHAQYVEDPITGRQSVLVPYEPPQVGTEFTTVLYNFMCNSSCVGGMNRRPILIIVTLETRDGQVLGRRCFEARICACPGRDRKADEDSIRKQQVSDSTKNGDGTKRPFRQGTHGIQMTSIKKRRSPDDELLYLPVRGRETYEMLLKIKESLELMQYLPQHTIETYRQQQQQQHQHLLQKQTSMQSQSSYGSNSPPLSKMNSMNKLPSVSQLINPQQRNALTPTTIPDGMGTNIPMMGTHMAMTGDMNGLSPTQALPPPLSMPSTSHCTPPPPYPTDCSIVSFLARLGCSSCVDYFTTQGLTTIYQIEHYSMDDLVSLKIPEQFRHAIWKGILDHRQLHDFSSPPHLLRTPSGASTVSVGSSETRGERVIDAVRFTLRQTISFPPRDEWNDFNFDMDARRNKQQRIKEEGE

>XP_035190617.1 tumor protein 63 isoform X1 [Oxyura jamaicensis]

MWESCCPLESCKDQRLKTVANCRFVETPSHFSWKESYYRSAMSQSSQPREFLSPEVIQHIWDFLEQPICSVQPIDLNFIDDPSENGPTNKIEISMDCVRLQDTELSDPMWPQYTNLGLLNSMDQQIQNGSSSTSPYNTEHTQNSVTAPSPYAQPSSTFDALSPSPAIPSNTDYPGPHSFDVSFQQSSTAKSATWTYSTELKKLYCQIAKTCPIQIKVLTPPPQGAVIRAMPVYKKAEHVTEVVKRCPNHELSREFNEGQIAPPSHLIRVEGNSHAQYVEDPITGRQSVLVPYEPPQVGTEFTTVLYNFMCNSSCVGGMNRRPILIIVTLETRDGQVLGRRCFEARICACPGRDRKADEDSIRKQQVSDSTKNGDGTKRPFRQGTHGIQMTSIKKRRSPDDELLYLPVRGRETYEMLLKIKESLELMQYLPQHTIETYRQQQQQQHQHLLQKQTSMQSQSSYGSNSPPLSKMNSMNKLPSVSQLINPQQRNALTPTTIPDGMGTNIPMMGTHMAMTGDMNGLSPTQALPPPLSMPSTSHCTPPPPYPTDCSIVSFLARLGCSSCVDYFTTQGLTTIYQIEHYSMDDLVSLKIPEQFRHAIWKGILDHRQLHDFSSPPHLLRTPSGASTVSVGSSETRGERVIDAVRFTLRQTISFPPRDEWNDFNFDMDARRNKQQRIKEEGE

>NWQ78825.1 P63 protein [Columbina picui]

FVCVLFSFSFFFFCFCFLRFVETPSHFSWKESYYRSAMSQNSQPREFLSPEVLQHIWDFLEQPICSVQPIDLNFIDDPSENGPTNKIEISMDCVRVQDTELNDPMWPQYTNLGLLNSMDQQIQNGSSSTSPYNTEHAQNSVTAPSPYAQPSSTFDALSPS

PAIPSNTDYPGPHSFDVSFQQSSTAKSATWTYSTELKKLYCQIAKTCPIQIKVMTPPPQGAVIRAMPVYKKAEHVTEVVKRCPNHELSREFNEGQIAPPSHLIRVEGNSHAQYVEDPITGRQSVLVPYEPPQVGTEFTTVLYNFMCNSSCVGGMNRRPILIIVTLETRDGQVLGRRCFEARICACPGRDRKADEDSIRKQQVSDSTKNGDGTKRPFRQGTHGIQMTSIKKRRSPDDELLYLPVRGRETYEMLLKIKESLELMQYLPQHTIETYRQQQQQQHQHLLQKQTSMQSQSSYGSNSPPLSKMNSMNKLPSVSQLINPQQRNALTPTTIPDGMGTNIPMMGTHMAMTGDMNGLSPTQALPPPLSMPSTSHCTPPPPYPTDCSIVSFLARLGCSSCVDYFTTQGLTTIYQIEHYSMDDLVSLKIPEQFRHAIWKGILDHRQLHDFSSPPHLLRTPSGASTVSVGSSETRGERVIDAVRFTLRQTISFPPRDEWNDFNFDMDARRNKQQRIKEEGE

**>KYO25602.1 tumor protein 63 isoform A [Alligator mississippiensis]**

MNFEPAPYPTLQYYPDPCIQRTSLSFARWFVETPGHFSWKESYYRSAMSQSSQTRDFLSPEVFQHIWDFLEQPICSMQPIDLNFIDDPSENGPTNKIEISMDCVRMQDADPGDPMWGLPEGGGVRVKTLEPEERTAALINLTASMLYLENNAQSQYSEPQYTNLGLLNSMDQQIQNGSSSTSPYNTEHAQNSVTAPSPYAQPSSTFDALSPSPAIPSNTDYPGPHSFDVSFQQSSTAKSATWTYSTELKKLYCQIAKTCPIQIKVMTPPPQGAVIRAMPVYKKAEHVTEVVKRCPNHELSREFNEGQIAPPSHLIRVEGNSHAQYVEDPITGRQSVLVPYEPPQVGTEFTTVLYNFMCNSSCVGGMNRRPILIIVTLETRDGQVLGRRCFEARICACPGRDRKADEDSIRKQQVSDSTKNGDGTKRPFRQGTHGIQMTSIKKRRSPDDELLYLPVRGRETYEMLLKIKESLELMQYLPQHTIETYRQQQQQQHQHLLQKQSSMQSQSTYGSSSPPLSKMNSMNKLPSVSQLINPQQRNALTPTTIPDGMGGNIPMMGAHMAMTGDMNGLSPTQGLPPPLSMPSTSHCTPPPPYPSDCSIVSFLARLGCSSCVDYFTTQGLTTIYQIEHYSMDDLVSLKIPEQFRHAIWKGILDHRQLHDFSSPPHLLRTPSGASTVSVGSSETRGERVIDAVRFTLRQTISFPPRDEWNDFNFDLDARRNKQQRIKEEGE

>KQK78197.1 tumor protein 63 isoform X1 [Amazona aestiva]

MNFEPAPFTTLQYYPDPCIQRFVETPSHFSWKENYYRSAMSQSSQPREFLSPEVLQHIWDILEQPIRSVQPIDLNFIDSPSENGPTNKIEISMDCVRVQDTELNDPMWRLVIRNSNYLMSWERPLSIGVEDSRWHFDRLDRGWEVFVKILEPEERTAALINLTANMLYLENNAQSQYSEPQYTNLGLLNSMDQQIQNGSSSTSPYNTEHAQNSVTAPSPYAQPSSTFDALSPSPAIPSNTDYPGPHSFDVSFQQSSTAKSATWTYSTELKKLYCQIAKTCPIQIKVMTPPPQGAVIRAMPVYKKAEHVTEVVKRCPNHELSREFNEGQIAPPSHLIRVEGNSHAQYVEDPITGRQSVLVPYEPPQVGTEFTTVLYNFMCNSSCVGGMNRRPILIIVTLETRDGQVLGRRCFEARICACPGRDRKADEDSIRKQQVSDSTKNGDGTKRPFRQGTHGIQMTSIKKRRSPDDELLYLPVRGRETYEMLLKIKESLELMQYLPQHTIETYRQQQQQQHQHLLQKQTSMQSQSSYGSNSPPLSKMNSMNKLPSVSQLINPQQRNALTPTTIPDGMGTNIPMMGTHMAMTGDMNGLSPTQALPPPLSMPSTSHCTPPPPYPTDCSIVSFLARLGCSSCVDYFTTQGLTTIYQIEHYSMDDLVSLKIPEQFRHAIWKGILDHRQLHDFSSPPHLLRTPSGASTVSVGSSETRGERVIDAVRFTLRQTISFPPRDEWNDFNFDMDARRNKQQRIKEEGE

>XP_025058517.1 tumor protein 63 isoform X2 [Alligator sinensis]

MNFEPAPYPTLQYYPDPCIQRFVETPGHFSWKESYYRSAMSQSSQTRDFLSPEVFQHIWDFLEQPICSMQPIDLNFIDDPSENGPTNKIEISMDCVRMQDADPGDPMWPQYTNLGLLNSMDQQIQNGSSSTSPYNTEHAQNSVTAPSPYAQPSSTFDALSPSPAIPSNTDYPGPHSFDVSFQQSSTAKSATWTYSTELKKLYCQIAKTCPIQIKVMTPPPQGAVIRAMPVYKKAEHVTEVVKRCPNHELSREFNEGQIAPPSHLIRVEGNSHAQYVEDPITGRQSVLVPYEPPQVGTEFTTVLYNFMCNSSCVGGMNRRPILIIVTLETRDGQVLGRRCFEARICACPGRDRKADEDSIRKQQVSDSTKNGDAFRQGTHGIQMTSIKKRRSPDDELLYLPVRGRETYEMLLKIKESLELMQYLPQHTIETYRQQQQQQHQHLLQKQSSMQSQSTYGSSSPPLSKMNSMNKLPSVSQLINPQQRNALTPTTIPDGMGGNIPMMGAHMAMTGDMNGLSPTQGLPPPLSMPSTSHCTPPPPYPSDCSIVSFLARLGCSSCVDYFTTQGLTTIYQIEHYSMDDLVSLKIPEQFRHAIWKGILDHRQLHDFSSPPHLLRTPSGASTVSVGSSETRGERVIDAVRFTLRQTISFPPRDEWNDFNFDLDARRNKQQRIKEEGE

>XP_032633220.1 tumor protein 63 isoform X1 [Chelonoidis abingdonii]

MNFEPAPYTTLQYYPDPCMQRFVETPGHFSWTESYYRSTMSQSSQTREFLSPEVFQQIWDFLEQPICSVQPINLNFMDDTSDNGPTNKIEISMDCVRMQDADGSDPMWPQYTNLGLLNSMEQQIQNGSSSTSPYNTEHAQNSVTAPSPYAQPSSTFDALSPSPAIPSNTDYPGPHSFDVSFQQSSTAKSATWTYSTELKKLYCQIAKTCPIQVKVMTPPPQGAIIRAMPVYKKAEHVTEVVKRCPNHELSREFNEGQIAPPSHLIRVEGNSHAQYVEDPITGRQSVLVPYEPPQVGTEFTTVLYNFMCNSSCVGGMNRRPILIIVTLETRDGQVLGRRCFEARICACPGRDRKADEDSIRKQQVSDSTKNGDGTKRPFRQSTHGIQMTSIKKRRSPDDELLYLPVRGRETYEMLLKIKESLELMQYLPQHTIETYRQQQQQQQQHLLQKQTSMQSQSSYGSSSPPLSKMNSMNKLPSVSQLINPQQRNALTPTAIPDGMGTNIPMMGAHMAMTGDMNGLSPTQALPPSLSMPSTSHCTPPPPYPTDCSIVSFLARLGCSSCVDYFTTQGLTTIYQIEHYSMDDLVSLKIPEQFRHAIWKGILDHRQLHDFSSPPHLLRTPSGASTVSVGSSETRGERVIDAVRFTLRQTISFPPRDEWNDFNFDMDARRNKQQRIKEEGE

>NXD76531.1 P63 protein [Halcyon senegalensis]

CFLRFVETPSHFSWKESYYRSAMSQSSQPREFLSPEVLQHIWDFLEQPICSVQPIDLNFIDDPSENGPTNKIEISMDCVRVQDTELNDPMWPQYTNLGLLNSMDQQIQNGSSSTSPYNTEHAQNSVTAPSPYAQPSSTFDALSPSPAIPSNTDYPGPHSFDVSFQQSSTAKSATWTYSTELKKLYCQIAKTCPIQIKVMTPPPQGAVIRAMPVYKKAEHVTEVVKRCPNHELSREFNEGQIAPPSHLIRVEGNSHAQYVEDPITGRQSVLVPYEPPQVGTEFTTVLYNFMCNSSCVGGMNRRPILIIVTLETRDGQVLGRRCFEARICACPGRDRKADEDSIRKQQVSDSTKNGDGTKRPFRQGTHGIQMTSIKKRRSPDDELLYLPVRGRETYEMLLKIKESLELMQYLPQHTIETYRQQQQQQHQHLLQKQTSMQSQSSYGSSSPPLGKMNSMNKLPSVSQLINPQQRNALTPTTIPDGMGTNIPMMGTHMAMTGDMNGLSPTQALPPPLSMPSTSHCTPPPPYPTDCSIVSFLARLGCSSCVDYFTTQGLTTIYQIEHYSMDDLVSLKIPEQFRHAIWKGILDHRQLHDFSSPPHLLRTPSGASTVSVGSSETRGERVIDAVRFTLRQTISFPPRDEWNDFNFDMDARRNKQQRIKEEGE

>NWR58742.1 P63 protein [Bucorvus abyssinicus]

VFFLFFPFLSFSFFFCFCFLRFVETPSHFSWKESYYRSAMSQSSQPRDFLNPEVFQHIWDFLEQPICSVQPIDLNFIDGPSENGPTNKIEISMDCVRVQDTELNDPMWPQYTNLGLLNSMDQQIQNGSSSTSPYNTEHAQNSVTAPSPYAQPSSTFDALSPSPAIPSNTDYPGPHSFDVSFQQSSTAKSATWTYSTELKKLYCQIAKTCPIQIKVMTPPPQGAVIRAMPVYKKAEHVTEVVKRCPNHELSREFNEGQIAPPSHLIRVEGNSHAQYVEDPITGRQSVLVPYEPPQVGTEFTTVLYNFMCNSSCVGGMNRRPILIIVTLETRDGQVLGRRCFEARICACPGRDRKADEDSIRKQQVSDSTKNGDGTKRPFRQGTHGIQMTSIKKRRSPDDELLYLPVRGRETYEMLLKIKESLELMQYLPQHTIETYRQQQQQQHQHLLQKQTSMQSQSSYGSNSPPLSKMNSMNKLPSVSQLINPQQRNALTPTTIPDGMGTNIPMMGTHMAMTSDMNGLSPTQALPPPLSMPSTSHCTPPPPYPTDCSIVSFLARLGCSSCVDYFTTQGLTTIYQIEHYSMDDLVSLKIPEQFRHAIWKGILDHRQLHDFSSPPHLLRTPSGASTVSVGSSETRGERVIDAVRFTLRQTISFPPRDEWNDFNFDMDARRNKQQRIKEEGE

>XP_039344729.1 tumor protein 63 isoform X1 [Mauremys reevesii]

MNFEPAPYATLQYYPDPCMQRFVETPGHFSWTESYYRSTMSQSSQTREFLSPEVFQQIWDFLEQPICSVQPINLNFMEDTSDNGPTNKIEISMDCVRMQDADGSDPMWPQYTNLGLLNSMDQQIQNGSSSTSPYNTEHAQNSVTAPSPYAQPSSTFDALSPSPAIPSNTDYPGPHSFDVSFQQSSTAKSATWTYSTELKKLYCQIAKTCPIQVKVMTPPPQGAIIRAMPVYKKAEHVTEVVKRCPNHELSREFNEGQIAPPSHLIRVEGNSHAQYVEDPITGRQSVLVPYEPPQVGTEFTTVLYNFMCNSSCVGGMNRRPILIIVTLETRDGQVLGRRCFEARICACPGRDRKADEDSIRKQQVSDSTKNGDGTKRPFRQSTHGIQMTSIKKRRSPDDELLYLPVRGRETYEMLLKIKESLELMQYLPQHTIETYRQQQQQQQQHLLQKQTSMQSQSSYGSNSPPLSKMNSMNKLPSVSQLINPQQRNALTPTAIPDGMGANIPMMGAHMAMTGDMNGLSPTQALPPSLSMPSTSHCTPPPPYPTDCSIVSFLARLGCSSCVDYFTTQGLTTIYQIEHYSMDDLVSLKIPEQFRHAIWKGILDHRQLHDFSSPPHLLRTPSGASTVSVGSSETRGERVIDAVRFTLRQTISFPPRDEWNDFNFDMDARRNKQQRIKEEGE

>XP_030432128.1 tumor protein 63 isoform X1 [Gopherus evgoodei]

MNFEPAPYATLQYYPDPCMQRFVETPGHFSWTESYYRSTMSQSSQTREFLSPEVFQQIWDFLEQPICSVQPINLNFMDDTSDNGPTNKIEISMDCVRMQDADGSDPMWPQYTNLGLLNSMEQQIQNGSSSTSPYNTEHAQNSVTAPSPYAQPSSTFDALSPSPAIPSNTDYPGPHSFDVSFQQSSTAKSATWTYSTELKKLYCQIAKTCPIQVKVMTPPPQGAIIRAMPVYKKAEHVTEVVKRCPNHELSREFNEGQIAPPSHLIRVEGNSHAQYVEDPITGRQSVLVPYEPPQVGTEFTTVLYNFMCNSSCVGGMNRRPILIIVTLETRDGQVLGRRCFEARICACPGRDRKADEDSIRKQQVSDNTKNGDGTKRPFRQSTHGIQMTSIKKRRSPDDELLYLPVRGRETYEMLLKIKESLELMQYLPQHTIETYRQQQQQQQQHLLQKQTSMQSQSSYGSNSPPLSKMNSVNKLPSVSQLINPQQRNALTPTAIPDGMGTNIPMMGAHMAMAGDMNGLSPTQALPPSLSMPSTSHCTPPPPYPTDCSIVSFLARLGCSSCVDYFTTQGLTTIYQIEHYSMDDLVSLKIPEQFRHAIWKGILDHRQLHDFSSPPHLLRTPSGASTVSVGSSETRGERVIDAVRFTLRQTISFPPRDEWNDFNFDMDARRNKQQRIKEEGE

**>XP_018117104.1 tumor protein p63 L homeolog isoform X1 [Xenopus laevis]**

MLYLENNAQAQYSEPQYTNLGLLNSMEQQIQNGSSSTSPYANDHAQNSVTAPSPYAQPSSTFDALSPSPAIPSNTDYPGPHSFDVSFQQSSTAKSATWTYSTDLKKLYCQIAKTCPIQIKVMTPPPQGAVVRAMPVYKKAEHVTEVVKRCPNHELSREFNEGQIAPPSHLIRVEGNNHAQYVEDPITGRQSVLVPYEPPQVGTEFTTVLYNFMCNSSCVGGMNRRPILIIVTLETRDGQVLGRRCFEARICACPGRDRKADEDSIRKQQVSDGTKNGEAFRQSTHGIQVTSIKKRRSPDDEVLYLPVKGREIYEMLLKIKESLELMQFIPQHTIESYRQQQQHLISKQDRSLQSNMNGPRNTGRSDTFSIQHWQKRMQNSPNYMEQTLKHSPIDQDLRDETYLNERSSLPVQSSFGSVSPPLGKMNSMNKLPSVSQLMNPQQRNSLTPNAMSDGMGANIPMMSTHMPMTNDLNGLSPSQTLPPTLSLPSTSHCTPPPPYPSDCSIASFLARLGCSSCLDYFTTQGLNTIYQIENYSIEDLASLKIPDQFRHAIWKGLMEHRQIHDFTSPSHLLRSTSSASTVSVGSNEPRGERVIDAVRFTLRQTISFPPRDDWNDFNFDLDTRRNKHQRIKEEGE

>XP_004914431.1 tumor protein 63 isoform X1 [Xenopus tropicalis]

MLYLENSAQAQYSEPQYTNLGLLNSMEQQIQNGSSSTSPYANDHAQNSVTAPSPYAQPSSTFDALSPSPAIPSNTDYPGPHSFDVSFQQSSTAKSATWTYSTDLKKLYCQIAKTCPIQVKVMTPPPQGAVVRAMPVYKKAEHVTEVVKRCPNHELSREFNEGQIAPPSHLIRVEGNSHAQYVEDPITGRQSVLVPYEPPQVGTEFTTILYNFMCNSSCVGGMNRRPILIIVTLETRDGQVLGRRCFEARICACPGRDRKADEDSIRKQQVSDSTKNGEAFRQSTHGIQMTSIKKRRSPDDEVLYLPVKGREIYEMLLKIKESLELMQFLPQHTIESYRQQQQHLLQKQDRSLQSNMNGPRNTGRSDTFSIQHWQKRMPNSPNYMEQTLKHSPIDQDLRDETYLNERSSLPGQSSFGSTSPPLGKMNNMNKLPSVSQLMNPQQRNSLTPNAMSDGMGANIPMMSTHMPMTSDLNGLSPSQTLPPSLSLPSTSHCTPPPPYPSDCSIASFLARLGCSSCLDYFTTQGLNTIYQIENYSIEDLASLKIPDQFRHAIWKGLMEHRQMHDFTSPPHLLRTTSSASTVSVGSNEPRGERVIDAVRFTLRQTISFPPRDDWNDFNFDLDTRRNKQQRIKEEGE

>XP_030071449.1 tumor protein 63 isoform X2 [Microcaecilia unicolor]

MLYLENNSQPQYSEPQYTNLGLLNSMDQQIQNGSSSTSPYNTEHAQNSVTAPSPYAQPSSTFDALSPSPAIPSNTDYPGPHSFDVSFQQSSTAKSATWTYSTDLKKLYCQIAKTCPIQIKVMTPPPQGAVIRAMPVYKKAEHVTEVVKRCPNHELSREFNEGQIAPASHLIRVEGNSHAQYVEDPITGRQSVLVPYEPPQVGTEFTTILYNFMCNSSCVGGMNRRPILIIVTLETRDGQVLGRRCFEARICACPGRDRKADEDSIRKQQVSDGTKSGDAFRQTTHGIQMTSIKKRRSPDDEVLYLPVKGREIYEMLLKIKESLELMQYLPQHTIDTYRQQATHLLQKQDRSLQSTMTGPRNTMRNDTFSIHQWQKRTQSSTNSPYVEHTVKHSPIDQEVRAENYLNERSSLQAQSSFGSNSPPLSKMNGINKLPSVSQLMNPQQRNALTPTVIPDGMGANIPLMGTHMPMTNDLNGLSPTQTLPPSLSVPSTSHCTPPPPYPTDCSISSFLARLGCSSCLDYFTTQGLTTIYQIEHYSIDDLASLKIPEQFRPLIWKGLMEHRQIQDFSSPPHLLRATSSGVSVGAESRGERVIDAVRFTLRQTISFPPRDEWNDFNFDMDARRNKQQRIKEEGEQEVRAENYLNERLLRPASGANCWSQGETLRNRVTAILKHSTPPNQSVYP

>XP_029471815.1 tumor protein 63 isoform X1 [Rhinatrema bivittatum]

MLYLENNSQPQYSEPQYTNLGLLNSMDQQIQNGSSSTSPYNTEHAQNSVTAPSPYAQPSSTFDALSPSPAIPSNTDYPGPHSFDVSFQQSSTAKSATWTYSTDLKKLYCQIAKTCPIQIKVMTPPPQGAVIRAMPVYKKAEHVTEVVKRCPNHELSREFNEGQIAPPSHLIRVEGNSHAQYVEDPITGRQSVLVPYEPPQVGTEFTTILYNFMCNSSCVGGMNRRPILIIVTLETRDGQVLGRRCFEARICACPGRDRKADEDSIRKQQVSDSTKNGDGMKRPFRQSTHGIQMTSIKKRRSPDDEVLYLPVKGREIYEMLLKIKESLELMQYLPQHTIDTYRQQQQHLLQKQDRSLQSTMAGPRNALRSDSFSIHQWQKRPQNTSNSPYMEHTLKQSPIEQDVRAENYLNERSSIQAQPSFGSSSPPLSKMNSINKLPSVSQLMNPQQRNALTPTAIPDGMGANIPLMGTHMPMTNDLNGLSPTQTLPPSLSMPSTSHCTPPPPYPTDCSISSFLARLGCSSCLDYFTTQGLTTIYQIEHYSIEDLVSLKIPDQFRAAIWKGLMEHRQIQDFSSPPHLLRATSSASSVSVGAEPRGERVIDAVRFTLRQTISFPPRDDWSDFNFDMDARRNKQQRIKEEGE

>XP_033815264.1 tumor protein 63 isoform X1 [Geotrypetes seraphini]

MLYLENNSQPQYNEPQYTNLGLLNSMDQQIQNGSSSTSPYNTEHAQNSVTAPSPYAQPSSTFDALSPSPAIPSNTDYPGPHSFDVSFQQSSTAKSATWTYSTDLKKLYCQIAKTCPIQIKVMTPPPQGAVIRAMPVYKKAEHVTEVVKRCPNHELSREFNEGQIAPPSHLIRVEGNSHAQYVEDPITGRQSVLVPYEPPQVGTEFTTILYNFMCNSSCVGGMNRRPILIIVTLETRDGQVLGRRCFEARICACPGRDRKADEDSIRKQQVSDNTKSGDGMKRPFRQTTHGIQMTSIKKRRSPDDEVLYLPVKGREIYEMLLKIKESLELMQYLPQHTIDTYRQQQQHLLQKQDRSLQSTMTGPRNTMRNDTFSIQQWQKRTQTSANSPYVEQSLHLKQSPIEQDVRAENYLNERSSLQAQASFGSSSPPLSKMNSMNKLPSVSQLMNPHQRNALTPTAIPDGMGANIPLMGTHMSMSNDLNGLSPTQTLPPSLSVPSTSHCTPPPPYPTDCSISSFLARLGCSSCLDYFTTQGLTTIYQIEHYSIDDLASLKIPEQFRPTIWKGLMEHRQIQDFSSPPHLLRASSSAVSVGAESRGERVIDAVRFTLRQTISFPPRDDWNDFNFDMDPRRNKQQRIKEEGE

>XP_029070770.1 tumor protein 63 isoform X7 [Monodon monoceros]

MLYLENNAQAQFSEPQYTNLGLLNSMDQQIQNGSSSTSPYNTDHAQNSVTAPSPYAQPSSTFDALSPSPAIPSNTDYPGPHSFDVSFQQSSTAKSATWTYSTELKKLYCQIAKTCPIQIKVMTPPPQGAVIRAMPVYKKAEHVTEVVKRCPNHELSREFNEGQIAPPSHLIRVEGNSHAQYVEDPITGRQSVLVPYEPPQVGTEFTTVLYNFMCNSSCVGGMNRRPILIIVTLETRDGQVLGRRCFEARICACPGRDRKADEDSIRKQQVSDSTKNGDAFRQNTHGIQMTSIKKRRSPDDELLYLPVRGRETYEMLLKIKESLELMQYLPQHTIETYRQQQQQQHQHLLQKQTSMQSQSSYGNSSPPLNKMNSMNKLPSVSQLINPQQRNTLTPTTIPDGMGANRFGKSKNP

>XP_034988370.1 tumor protein 63 isoform X4 [Zootoca vivipara]

MLYLENNAQSQYSEPQYTNLGLLNSMDQQIQNGSSSTSPYNTDHAQNSVTAPSPYAQPSSTFDALSPSPAIPSNTDYPGPHSFDVSFQQSSTAKSATWTYSTELKKLYCQIAKTCPIQIKVMTPPPQGAVIRAMPVYKKAEHVTEVVKRCPNHELSREFNEGQIAPPSHLIRVEGNSHAQYVEDPITGRQSVLVPYEPPQVGTEFTTVLYNFMCNSSCVGGMNRRPILIIVTLETRDGQVLGRRCFEARICACPGRDRKADEDSIRKQQVSDSTKNGDAFRQSTHGIQMTSVKKRRTPDDELLYLPVRGRETYEMLLKIKESLELMQYLPQHTIETYRQQQQQQQQHQHLLQKQNSIQSQSSYVSTSPPLGKMNSMNKLPSVSQLINPQQRNALTPTSMPDSMGANIPMMGAHMAMAGDMNGLSPTQTLPPTLSMPSTSHCTPPPPYPTDCSIVSFLARLGCSSCVDYFTAQGLTTIYQIEHYSMDDLVSLKIPEQFRHAIWKGIMDHRQIHDFSSAPHLLRTPGAASAVSVGPSETRGERVIDAVRFTLRQTISFPPRDEWSDFNFDLDARRNKQQRIKEEGE

>XP_025232997.1 tumor protein 63 isoform X10 [Theropithecus gelada]

MLYLENNAQTQFSEPQYTNLGLLNSMDQQIQNGSSSTSPYNTDHAQNSVTAPSPYAQPSSTFDALSPSPAIPSNTDYPGPHSFDVSFQQSSTAKSATWTYSTELKKLYCQIAKTCPIQIKVMTPPPQGAVIRAMPVYKKAEHVTEVVKRCPNHELSREFNEGQIAPPSHLIRVEGNSHAQYVEDPITGRQSVLVPYEPPQVGTEFTTVLYNFMCNSSCVGGMNRRPILIIVTLETRDGQVLGRRCFEARICACPGRDRKADEDSIRKQQVSDSTKNGDAFRQNTHGIQMTSIKKRRSPDDELLYLPVRGRETYEMLLKIKESLELMQYLPQHTIETYRQQQQQQHQHLLQKQTSIQSQSSYGNSSPPLNKMNSMNKLPSVSQLINPQQRNALTPTTIPDGMGANRFGKSKNP

>XP_009200201.1 tumor protein 63 isoform X5 [Papio anubis]

MYRERKRERDLSSVIFLVDSYRKGLRGGGGVGKILEPEERTAALINLTANMLYLENNAQTQFSEPQYTNLGLLNSMDQQIQNGSSSTSPYNTDHAQNSVTAPSPYAQPSSTFDALSPSPAIPSNTDYPGPHSFDVSFQQSSTAKSATWTYSTELKKLYCQIAKTCPIQIKVMTPPPQGAVIRAMPVYKKAEHVTEVVKRCPNHELSREFNEGQIAPPSHLIRVEGNSHAQYVEDPITGRQSVLVPYEPPQVGTEFTTVLYNFMCNSSCVGGMNRRPILIIVTLETRDGQVLGRRCFEARICACPGRDRKADEDSIRKQQVSDSTKNGDAFRQNTHGIQMTSIKKRRSPDDELLYLPVRGRETYEMLLKIKESLELMQYLPQHTIETYRQQQQQQHQHLLQKQTSIQSQSSYGNSSPPLNKMNSMNKLPSVSQLINPQQRNALTPTTIPDGMGANIPMMGTHMPMAGDMNGLSPTQALPPPLSMPSTSHCTPPPPYPTDCSIVSFLARLGCSSCLDYFTTQGLTTIYQIEHYSMDDLASLKIPEQFRHAIWKGILDHRQLHEFSSPSHLLRTPSSASTVSVGSSETRGERVIDAVRFTLRQTISFPPRDEWNDFNFDMDARRNKQQRIKEEGE

>XP_019801622.1 tumor protein 63 isoform X2 [Tursiops truncatus]

MLYLENNAQAQFSEPQYTNLGLLNSMDQQIQNGSSSTSPYNTDHAQNSVTAPSPYAQPSSTFDALSPSPAIPSNTDYPGPHSFDVSFQQSSTAKSATWTYSTELKKLYCQIAKTCPIQIKVMTPPPQGAVIRAMPVYKKAEHVTEVVKRCPNHELSREFNEGQIAPPSHLIRVEGNSHAQYVEDPITGRQSVLVPYEPPQVGTEFTTVLYNFMCNSSCVGGMNRRPILIIVTLETRDGQVLGRRCFEARICACPGRDRKADEDSIRKQQVSDSTKNGDAFRQNTHGIQMTSIKKRRSPDDELLYLPVRGRETYEMLLKIKESLELMQYLPQHTIETYRQQQQQQHQHLLQKQTSMQSQSSYGNSSPPLNKMNSMNKLPSVSQLINPQQRNTLTPTTIPDGMGANIPMMGTHMPMAGDMNGLSPTQALPPPLSMPSTSHCTPPPPYPTDCSLVSFLARLGCSSCLDYFTTQGLTTIYQIEHYSMDDLASLKIPEQFRHAIWKGILDHRQLHDFSSPPHLLRTPSGTSTVSVGSSETRGERVIDAVRFTLRQTISFPPRDEWNDFNFDMDARRNKQQRIKEEGE

>XP_009087388.1 tumor protein 63 isoform X2 [Serinus canaria]

MLYLENNAQSQYSEPQYTNLGLLNSMDQQIQNGSSSTSPYNTEHAQNSVTAPSPYAQPSSTFDALSPSPAIPSNTDYPGPHSFDVSFQQSSTAKSATWTYSTELKKLYCQIAKTCPIQIKVMTPPPQGAVIRAMPVYKKAEHVTEVVKRCPNHELSREFNEGQIAPPSHLIRVEGNSHAQYVEDPITGRQSVLVPYEPPQVGTEFTTVLYNFMCNSSCVGGMNRRPILIIVTLETRDGQVLGRRCFEARICACPGRDRKADEDSIRKQQVSDSTKNGDAFRQGTHGIQMTSIKKRRSPDDELLYLPVRGRETYEMLLKIKESLELMQFLPQHTIETYRQQQQQQHQHLLQKQTSMQSQSSYGSNSPPLSKMNSMNKLPSVSQLINPQQRNALTPTTIPDSMGTNIPMMGTHMAMTGDMNGLSPTQALPPPLSMPSTSHCTPPPPYPSDCSIVSFLARLGCSSCVDYFTTQGLTTIYQIEHYSMDDLVSLKIPEQFRHAIWKGILDHRQLHDFSSPPHLLRTPSGASTVSVGSSETRGERVIDAVRFTLRQTISFPPRDEWNDFNFDMDARRNKQQRIKEEGE

>XP_021040680.1 tumor protein 63 isoform X10 [Mus caroli]

MLYLENNAQTQFSEPQYTNLGLLNSMDQQIQNGSSSTSPYNTDHAQNSVTAPSPYAQPSSTFDALSPSPAIPSNTDYPGPHSFDVSFQQSSTAKSATWTYSTELKKLYCQIAKTCPIQIKVMTPPPQGAVIRAMPVYKKAEHVTEVVKRCPNHELSREFNEGQIAPPSHLIRVEGNSHAQYVEDPITGRQSVLVPYEPPQVGTEFTTVLYNFMCNSSCVGGMNRRPILIIVTLETRDGQVLGRRCFEARICACPGRDRKADEDSIRKQQVSDSAKNGDAFRQNTHGIQMTSIKKRRSPDDELLYLPVRGRETYEMLLKIKESLELMQYLPQHTIETYRQQQQQQHQHLLQKQTSMQSQSSYGNSSPPLNKMNSMNKLPSVSQLINPQQRNALTPTTMPEGMGANRFGKSEDP

>XP_039090145.1 tumor protein 63 isoform X5 [Hyaena hyaena]

MLYLENNAQTQFSEPQYTNLGLLNSMDQQIQNGSSSTSPYNTDHAQNSVTAPSPYAQPSSTFDALSPSPAIPSNTDYPGPHSFDVSFQQSSTAKSATWTYSTELKKLYCQIAKTCPIQIKVMTPPPQGAVIRAMPVYKKAEHVTEVVKRCPNHELSREFNEGQIAPPSHLIRVEGNSHAQYVEDPITGRQSVLVPYEPPQVGTEFTTVLYNFMCNSSCVGGMNRRPILIIVTLETRDGQVLGRRCFEARICACPGRDRKADEDSIRKQQVSDSAKNGDAFRQNTHGIQMTSIKKRRSPDDELLYLPVRGRETYEMLLKIKESLELMQYLPQHTIETYRQQQQQQHQHLLQKQTSMQSQSSYGNSSPPLNKMNSMNKLPSVSQLINPQQRNALTPTTIPDGMGANRFGKSKNP

>XP_023965824.1 tumor protein 63 isoform X2 [Chrysemys picta bellii]

MLYLENNAQSQYSEPQYTNLGLLNSMDQQIQNGSSSTSPYNTEHAQNSVTAPSPYAQPSSTFDALSPSPAIPSNTDYPGPHSFDVSFQQSSTAKSATWTYSTELKKLYCQIAKTCPIQVKVMTPPPQGAIIRAMPVYKKAEHVTEVVKRCPNHELSREFNEGQIAPPSHLIRVEGNSHAQYVEDPITGRQSVLVPYEPPQVGTEFTTVLYNFMCNSSCVGGMNRRPILIIVTLETRDGQVLGRRCFEARICACPGRDRKADEDSIRKQQVSDSTKNGDAFRQSTHGIQMTSIKKRRSPDDELLYLPVRGRETYEMLLKIKESLELMQYLPQHTIETYRQQQQQQHQHLLQKQTSMQSQSSYGSNSPPLSKMNSMNKLPSVSQLINPQQRNALTPTAIPDGMGTNIPMMGTHMAMTGDMNGLSPTQALPPSLSMPSTSHCTPPPPYPTDCSIVSFLARLGCSSCVDYFTTQGLTTIYQIEHYSMDDLVSLKIPEQFRHAIWKGILDHRQLHDFSSPPHLLRTPSGASTVSVGSSETRGERVIDAVRFTLRQTISFPPRDEWNDFNFDMDARRNKQQRIKEEGE

>XP_040284306.1 tumor protein 63 isoform X1 [Bufo bufo]

MLYLESSAQAQYSEPQYTNLGLLNSMEQQIQNGSSSTSPYANEHAQNSVTTPSPYAQPSSTFDALSPSPAIPSNTDYPGSHSFDVSFQQSSTAKSATWTYSTDLKKLYCQIAKTCPIQIKVMTPPPQGAVIRAMPVYKKAEHVTEVVKRCPNHELSREFNEGQMAPASHLIRVEGHSHAQYVEDPITGRQSVLVPYEPPQVGTEFTTILYNFMCNSSCVGGMNRRPILIIVTLETRDGQVLGRRCFEARICACPGRDRKADEDSIRKQQGSDSTKNGEGMKQSFRQTTHGIQMTSVKKRRLPDDEVLYLPVKGREIYETLLKIKESLELMQYIPQHTIETYRQQQQHLLQKQDRSLQSNMPGSRNTGRSDIFSRHQWQKRMQSSPNYMEHNLKYSPIEQDVRDENYLNERLIVCQQNIPVYTQWFPATNSDFMQDINDGSLDKQALQGSLQGQPSFGSASPPLSKMNNMNKLPSVSQLMNPQQRNSLTPNAMTDGMGSNLPMISAHMPMAGDLNGLSPPQTLPPSLSMTTTSHCTPPPPYPTDCSISSFLARLGCSSCLDYFTTQGLSSIYQIEHYSMEDLISLKLPEQFRHAIWKGLVEHRQIHEYSSPPHLLRTTSSASTVSVGSNESRSERVIDAVRFTLRQTISFPPRDDWNDFNFDMDARRNKQQRIKEEGE

>XP_026711026.1 tumor protein 63 isoform X3 [Athene cunicularia]

MLYLENNAQSQYSEPQYTNLGLLNSMEQQIQNGSSSTSPYNTEHAQNSVTAPSPYAQPSSTFDALSPSPAIPSNTDYPGPHSFDVSFQQSSTAKSATWTYSTELKKLYCQIAKTCPIQIKVMTPPPQGAVIRAMPVYKKAEHVTEVVKRCPNHELSREFNEGQIAPPSHLIRVEGNSHAQYVEDPITGRQSVLVPYEPPQVGTEFTTVLYNFMCNSSCVGGMNRRPILIIVTLETRDGQVLGRRCFEARICACPGRDRKADEDSIRKQQVSDSTKNGDAFRQGTHGIQMTSIKKRRSPDDELLYLPVRGRETYEMLLKIKESLELMQYLPQHTIETYRQQQQQQHQHLLQKQTSMQSQSSYGSNSPPLSKMNSMNKLPSVSQLINPQQRNALTPTTIPDGMGTNIPMMGTHMAMTGDMNGLSPTQALPPPLSMPSTSHCTPPPPYPTDCSIVSFLARLGCSSCVDYFTTQGLTTIYQIEHYSMDDLVSLKIPEQFRHAIWKGILDHRQLHDFSSPPHLLRTPSGASTVSVGSSETRGERVIDAVRFTLRQTISFPPRDEWNDFNFDMDARRNKQQRIKEEGE

>XP_024420505.1 tumor protein 63 isoform X2 [Desmodus rotundus]

MLYLENNAQTQFSEPQYTNLGLLNSMDQQIQNGSSSTSPYNTDHAQNSVTAPSPYAQPSSTFDALSPSPAIPSNTDYPGPHSFDVSFQQSSTAKSATWTYSTELKKLYCQIAKTCPIQIKVMTPPPQGAVIRAMPVYKKAEHVTEVVKRCPNHELSREFNEGQIAPPSHLIRVEGNSHAQYVEDPITGRQSVLVPYEPPQVGTEFTTVLYNFMCNSSCVGGMNRRPILIIVTLETRDGQVLGRRCFEARICACPGRDRKADEDSIRKQQVSDSTKNGDAFRQNTHGIQMTSIKKRRSPDDELLYLPVRGRETYEMLLKIKESLELMQYLPQHTIETYRQQQQQQHQHLLQKQTSMQSQTSYGNSSPPLNKMNSMNKLPSVSQLINPQQRNALTPTTIPDGMGANIPMMGTHMPMAGDMNGLSPTQALPPPLSMPSTSHCTPPPPYPTDCSLVSFLARLGCSSCLDYFTTQGLTTIYQIEHYSMDDLASLKIPEQFRHAIWKGILDHRQLHDFSSPPHLLRTPSGTSTVSVGSSETRGERVIDAVRFTLRQTISFPPRDEWNDFNFDMDARRNKQQRIKEEGE

>XP_023447812.1 tumor protein 63 isoform X2 [Dasypus novemcinctus]

MLYLENNAQTQFSEPQYTNLGLLNSMDQQIQNGSSSTSPYNTDHAQNSVTAPSPYAQPSSTFDALSPSPAIPSNTDYPGPHSFDVSFQQSSTAKSATWTYSTELKKLYCQIAKTCPIQIKVMTPPPQGAVIRAMPVYKKAEHVTEVVKRCPNHELSREFNEGQIAPPSHLIRVEGNSHAQYVEDPITGRQSVLVPYEPPQVGTEFTTVLYNFMCNSSCVGGMNRRPILIIVTLETRDGQVLGRRCFEARICACPGRDRKADEDSIRKQQVSDSTKNGDAFRQNTHGIQMTSIKKRRSPDDELLYLPVRGRETYEMLLKIKESLELMQYLPQHTIETYRQQQQQQHQHLLQKQTSMQSQASYGNSSPPLNKMNSMNKLPSVSQLINPQQRSALTPTTIPDGMGANIPMMGTHMPMAGDMNGLSPTQALPPPLSMPSTSHCTPPPPYPTDCSIVSFLARLGCSSCLDYFTTQGLTTIYQIEHYSMDDLASLKIPEQFRHAIWKGILDHRQLHDFSSPPHLLRTPSGASTVSVGSSETRGERVIDAVRFTLRQTISFPPRDEWNDFNFDMDARRNKQQRIKEEGE

>XP_008149816.2 tumor protein 63 isoform X2 [Eptesicus fuscus]

MYRERRRERDLSSVIFLVDSYCKGLRGGGGVGKILEPEERTAALINLTANMLYLENNAQTQFSEPQYTNLGLLNSMDQQIQNGSSSTSPYNTDHAQNSVTAPSPYAQPSSTFDALSPSPAIPSNTDYPGPHSFDVSFQQSSTAKSATWTYSTELKKLYCQIAKTCPIQIKVMTPPPQGAVIRAMPVYKKAEHVTEVVKRCPNHELSREFNEGQIAPPSHLIRVEGNSHAQYVEDPITGRQSVLVPYEPPQVGTEFTTVLYNFMCNSSCVGGMNRRPILIIVTLETRDGQVLGRRCFEARICACPGRDRKADEDSIRKQQVSDSTKNGDAFRQNTHGIQMTSIKKRRSPDDELLYLPVRGRETYEMLLKIKESLELMQYLPQHTIETYRQQQQQQHQHLLQKQTSMQSQSSYGNSSPPLNKMNSMNKLPSVSQLINPQQRNALTPTTIPDGMGANIPMMGTHMPVAGDMNGLSPTQTLPPPLSMPSTSHCTPPPPYPTDCSLVSFLARLGCSSCLDYFTTQGLTTIYQIEHYSMDDLASLKIPEQFRHAIWKGILDHRQLHDFSSPPHLLRTPSGTSTVSVGSSETRGERVIDAVRFTLRQTISFPPRDEWNDFNFDMDARRNKQQRIKEEGE

>XP_014307705.1 tumor protein 63 isoform X2 [Myotis lucifugus]

MYRERRRERDLSSVIFLVDSYCKGPRGGGGVGKILEPEERTAALINLTANMLYLENNAQTQFSEPQYTNLGLLNSMDQQIQNGSSSTSPYNTDHAQNSVTAPSPYAQPSSTFDALSPSPAIPSNTDYPGPHSFDVSFQQSSTAKSATWTYSTELKKLYCQIAKTCPIQIKVMTPPPQGAVIRAMPVYKKAEHVTEVVKRCPNHELSREFNEGQIAPPSHLIRVEGNSHAQYVEDPITGRQSVLVPYEPPQVGTEFTTVLYNFMCNSSCVGGMNRRPILIIVTLETRDGQVLGRRCFEARICACPGRDRKADEDSIRKQQVSDSTKNGDAFRQNTHGIQMTSIKKRRSPDDELLYLPVRGRETYEMLLKIKESLELMQYLPQHTIETYRQQQQQQHQHLLQKHSHYSPTRTSMQSQSSYGNSSPPLNKMNSMNKLPSVSQLINPQQRNALTPTTIPDGMGANIPMMGTHMPVAGDMNGLSPTQGLPPPLSMPSTSHCTPPPPYPTDCSLVSFLARLGCSSCLDYFTTQGLTTIYQIEHYSMDDLASLKIPEQFRHAIWKGILDHRQLHDFSSPPHLLRTPSGTSTVSVGSSETRGERVIDAVRFTLRQTISFPPRDEWNDFNFDMDARRNKQQRIKEEGE

**>AAI52688.1 Tumor protein p63 [Danio rerio]**

MLYLETNAPSSYSEPQYTSLGLLNSMDQNGGSTSTSPYNNDHAQNNVTAPSPYAQPSSTFEALSPSPAIPSNTDYAGPHTFDVSFQQSSTAKSATWTYSTELKKLYCQIAKTCPIQIKVLTNPPQGAVIRAMPVYKKAEHVTEVVKRCPNHELSREFNDGQIAPPSHLIRVEGNSHAQYVEDSITGRQSVLVPYEPPQVGTEFTTILYNFMCNSSCVGGMNRRPILIIVTLETRDGQVLGRRCFEARICACPGRDRKADEDSIRKQHVTDGTKSSEAFRQASSHLSQLNSIKKRRSTDEEVFCLPIKGREIYEILVKIKESLELMQFLPQQTIESYRQQHQNLLQKQSSLPPQPAFGSSSPTLGKNKLPSVSQLINPQQRNALTPSGMPGGLTDSLLQSQPFPLPSVTPPMMGGPVPMNTDLSSLSPNNPLQSQLQMVPSSHCTPPPPYPMDNSISSFLLRLGCSACLDYFTAQGLTNIYQIENYNLEDLSRLKIPTEFQHIIWKGIMEYRQTMEFSPPPHILRTSSGTSTVSVGSTEARGERVIDAVRFTLRQTISFPPRDDWTDFSFDLAPDSRRNKQQRIKEEGE

>RXN19434.1 tumor 63 isoform X1 [Labeo rohita]

MTSPYAAVQFCPERAFQRLREPAACLSWAEGSFLASMSQGQGSQGTDILGQDVLNQLLEMLDQSALHSMQPIELNFSEGSAVGSASNTIQISMDCITMRGHEEPLTPQYTSLGLLNSMDQNGGSTSTSPYNNEHAQNNVTAPSPYAQPSSTFEALSPSPAIPSNTDYAGPHTFDVSFQQSSTAKSATWTYSTELKKLYCQIAKTCPIQIKVLTNPPQGAVIRAMPVYKKAEHVTEVVKRCPNHELSREFNDGQIAPPSHLIRVEGNSHAQYVEDSITGRQSVLVPYEPPQVGTEFTTILYNFMCNSSCVGGMNRRPILIIVTLETRDGQVLGRRCFEARICACPGRDRKADEDSIRKQHVSDGTKSSEGTKRPFRQASHLSQLNSIKKRRSTDEEVFCLPIKGREIYEILVKIKESLELMQFLPQQTIESYRQQQQNLLQKQSSLPPQPTFGSSSPTLGKNKLPSVSQLINPQQRNTLTPSSMAGGLTDMTPPMMGGPVPMNTDMSALSPTNPLQPQLQMVPSSHCTPPPPYPMDNSISSFLLRLGCSACLDYFTAQGLTNIYQIENYNLEDLSRLKIPTEFQHIIWKGIMEYRQTMEFSPPPHILRTSSGTSTVSVGSTEARGERVIDAVRFTLRQTISFPPRDDWTDFSFDLAPDSRRNKQQRIKEEGE

>XP_026069545.1 tumor protein 63-like isoform X1 [Carassius auratus]

MTSPYAAVQFCPERAFQRLREPAAARLSWAEGSFLASMSQGQGSQGTDILGQDVLNQLLEMLDQSALHSMQPIEFNFSEGSAVGSASNTIQISMDCITMRGPEEPLTPQYTSLGLLNSMDQNGGSTSTSPYNNEHAQNNVTAPSPYAQPSSTFEALSPSPAIPSNTDYAGPHTFDVSFQQSSTAKSATWTYSTELKKLYCQIAKTCPIQIKVLTNPPQGAVLRAMPVYKKAEHVTEVVKRCPNHELSREFNDGQIAPPSHLIRVEGNSHAQYVEDSITGRQSVLVPYEPPQVGTEFTTVLYNFMCNSSCVGGMNRRPILIIVTLETRDGQVLGRRCFEARICACPGRDRKADEDSIRKQHVSDGSKSSEGTKRPFRQTPHLSQLNSIKKRRSTDEEVFCLPIKGREIYEILVKIKESLELMQFLPQQTIESYRQQQQNLLQKQNSLPPQPAFGSSSPTLCKNKLPSVSQLINPQQRNTLTPSSMTGGLTDSLLQSQPFPLPSVTPPMMGGPVHMNTDMNALSPTNPLQSQLQMVPSHCTPPPPYPMDNSISSFLLRLGCSACLDYFTAQGLTNIYQIENYNLEDLSRLKIPTEFQHIIWKGIMEYRQTMEFSPPPHILRTSSGTSTVSVGPTEARGERVIDAVRFTLRQTISFPPRDDWTDFSFDLAPDSRRNKQQRIKEEGE

>XP_030646922.1 tumor protein 63 isoform X1 [Chanos chanos]

MTSPYGAVQYHPERTFQRLRDPSSCLAWAESSFLTTMSQTQSSQGTDILSQDVFSQLLDMLDQSAIHSVQPIELNFSEGEVDGSPSNTIQISMDCITMRGPENSFSPQYTNLGILNSMDQNIQNGSSTSTSPYNNDHAQNNVTAPSPYAQPSSTFEALSPSPAIPSNTDYAGPHSFDVSFQQSSTAKSATWTYSTELKKLYCQIAKTCPIQIKVLTNPPQGAVIRAMPVYKKAEHVTEVVKRCPNHELSREFNDGQIAPPSHLIRVEGNSHAQYVEDSITGRQSVLVPYEPPQVGTEFTTILYNFMCNSSCVGGMNRRPILIIVTLETRDGQVLGRRCFEARICACPGRDRKADEDSIRKQHVSDGTKSSEAFRQASHGIQLSSIKKRRSTDEEVFCLPIKGREIYEILVKIKESLELMQFLPQHTIESYRQQQQSLLQKQSSMPPQPAFGSSSPTHGKVNKLPSVSQLMNPQQRNTLTPSSMPGGLTDSNPMMGGHIPMNTDMSALSPTLQPQLPMVPSSHCTPPPPYPMDSSISSFLLRIGCSACLDYFTAQGLTNIYQIENYNLEDLSRLKIPAEFQHVIWKGIMEHRQSMEFSPPPHILRTSGGASTVSVGSTEARGERVIDAVRFTLRQTISFPPRDDWTDFSFDLDSRRNKQQRIKEEGE

>XP_017553525.1 tumor protein 63 isoform X1 [Pygocentrus nattereri]

MTSPYAAVQFCPEHAFQRRLREPAACLSWAEGSFLASMSQTPSSQGTDILGQDVFNQLLEMLDQSAFHSVQPIELNFSEGSTDSPPSNTIQISMDCITMHGSEEHLSSQYTNLGLLNGMDQNGGSTSTSPYNNEHAQNNVTAPSPYAQPSSTFEALSPSPAIPSNTDYAGPHTFDVSFQQSSTAKSATWTYSTELKKLYCQIAKTCPIQIKVLTSPPQGAVIRAMPVYKKAEHVTEVVKRCPNHELSREFNDGQIAPPSHLIRVEGNSHAQYVEDSITGRQSVLVPYEPPQVGTEFTTILYNFMCNSSCVGGMNRRPILIIVTLETRDGQVLGRRCFEARICACPGRDRKADEDSIRKQHVSDGTKSSEGTKRPYRQASHGIQISSIKKRRSTDEEVFCLPIKGREIYEILVKIKESLELMQFLPQHTIESYRQQQQSLLQKQWDLSDDQNYRPCPPLCSRSPLPPQPAFGSSSPPHGKVNKLPSVSQLINPQQRNTLTPSSMAGGLTDIPPPAVTPMMSGHIPMNTDMSSLSPTHALQPQLSMVPSSHCTPPPPYPMDSSISSFLLRLGCSSCLDYFTAQGLTNIYQIENYSLEDLSRLKIPTEFQHVIWKGIMDHRQTMEFSPPPHILRTSSGASTVSVGSTEARGERVIDAVRFTLRQTISFPPRDDWTDFSFDLAPDSRRNKQQRIKEEGE

>XP_036426238.1 tumor protein 63 isoform X1 [Colossoma macropomum]

MTSPYAAVQFCPEHAFQRRLREPAACLSWAEGSFLASMSQTPSSQGTDILGQDVFNQLLEMLDQSAFHSVQPIELNFSEGSTDSPPSNTIQISMDCITMHGSEEHLSSQYTNLGLLNGMDQNGGSTSTSPYNNEHAQNNVTAPSPYAQPSSTFEALSPSPAIPSNTDYAGPHTFDVSFQQSSTAKSATWTYSTELKKLYCQIAKTCPIQIKVLTSPPQGAVIRAMPVYKKAEHVTEVVKRCPNHELSREFNDGQIAPPSHLIRVEGNSHAQYVEDSITGRQSVLVPYEPPQVGTEFTTILYNFMCNSSCVGGMNRRPILIIVTLETRDGQVLGRRCFEARICACPGRDRKADEDSIRKQHVSDGTKSSEGTKRPYRQASHGIQISSIKKRRSTDEEVFCLPIKGREIYEILVKIKESLELMQFLPQHTIESYRQQQQSLLQKQWDMSDDQNYRPCPPLCSRSPLPPQPAFGSSSPPHGKVNKLPSVSQLINPQQRNTLTPSSMAGGLTDIPPPAVTPMMSGHIPMNTDMSSLSPTHALQPQLSMVPSSHCTPPPPYPMDSSISSFLLRLGCSSCLDYFTAQGLTNIYQIENYSLEDLSRLKIPTEFQHVIWKGIMDHRQTMEFSPPPHILRTSSGASTVSVGSTEARGERVIDAVRFTLRQTISFPPRDDWTDFSFDLAPDSRRNKQQRIKEEGE

>XP_026863519.1 tumor protein 63 isoform X1 [Electrophorus electricus]

MTSPYAAVQFYPEHTFQRRLRDPAACLSWAEGSFLASMSQSPSSQGTDILSQDVFNQLLEMLDQSAFHSVQPIELNFSEGSSDSSASNTIQISMDCITMHGSDEHLTSQYTNLGLLNGMDQNGGSTSTSPYNNEHAQNNVTTPSPYAQPSSTFETLSPSPAIPSNTDYAGPHTFDVSFQQSSTAKSATWTYSTDLKKLYCQIAKTCPIQIKVLTNPPQGAVIRAMPVYKKAEHVTEVVKRCPNHELSREFNDGQIAPPSHLIRVEGNSHAQYVEDSITGRQSVLVPYEPPQVGTEFTTILYNFMCNSSCVGGMNRRPILIIVTLETRDGQVLGRRCFEARICACPGRDRKADEDSIRKQHVSDGTKNSEGTKRPFRQASHGIQISSIKKRRSTDEEVFCLPIKGREIYEILVKIKESLELMQFLPQHTIESYRQQQQNLLQKQSPLPPQPAFGSASPPHGKVNKLPSVSQLINPQQRNTLTPSSMAGGLTDMSPMMSGHIPMNTDMSSLSPTHGLQPQLPMVPSSHCTPPPPYPVDSSISSFLLRLGCSSCLDYFTAQGLTNIYQIENYNLEDLSRLKIPAEFQHIIWKGIMEHRQTMEFSPPPHILRTSSGASTVSVGSTEARGERVIDAVRFTLRQTISFPPRDDWTDFSFDLAPDSRRNKQQRIKEEGE

>XP_022531206.1 tumor protein 63 isoform X1 [Astyanax mexicanus]

MTSPYAAVQFYPEHAFQRRLREPAACLSWAEGSFLASMSQTPSSQGTDILGQDVFNQLLEMLDQSAFHSVQPIELNFSDGSNDSSPSNTIQISMDCITMHGSEEHLASQYTNLGLLNGMDQNGGSTSTSPYNNEHAQNSVAAPSPYAQPSSTFEALSPSPAIPSNTDYAGPHTFDVSFQQSSTAKSATWTYSTDLKKLYCQIAKTCPIQIKVLTNPPQGAVIRAMPVYKKAEHVTEVVKRCPNHELSREFNDGQIAPPSHLIRVEGNSHAQYVEDSITGRQSVLVPYEPPQVGTEFTTILYNFMCNSSCVGGMNRRPILIIVTLETRDGQVLGRRCFEARICACPGRDRKADEDSIRKQHVSDGTKSSEGTKRPYRQASHGIQISSIKKRRSTDEEVFCLPIKGREIYEILVKIKESLELMQFLPQHTIESYRQQQQSLLQKQWDLSDDQNYRPCPPPLCSRSPLPPQPAFGSSSPPHGKVNKLPSVSQLINPQQRNTLTPSSMAAGLTDMTPMMSAHIPMNTDLSSLSPTHALQPQLPMVPSSHCTPPPPYPVDSSISSFLLRLGCSSCLDYFTAQGLTNIYQIENYNIEDLSRLKIPTDFQHLIWKGIMEHRQNMEFSPPPHILRTSSGASTVSLGSTEARGERVIDAVRFTLRQTISFPPRDDWTDFSFDLAPDSRRNKQQRIKEEGE

>XP_022623675.1 tumor protein 63 isoform X6 [Seriola dumerili]

MNSPYTAVQYYPEFPFHRLRDPPSRLSWRESSFLTTMSQNQSAQTTDFLSQDVFNQLFDMLDQSAIHTVQPIELNFTDSPTDGSAGNTIQISMDCITMHEPDETLSSQYTNLGLLNSMDQNIQNGGSTSTSPYNNDHAQNNVTAPSPYAQPSSTFDALSPSPAIPSNTDYAGPHTFDVSFQQSSTAKSATWTYSTDLKKLYCQIAKTCPIQIKVLTTPPQGAVIRAMPVYKKAEHVTEVVKRCPNHELSREFNDGQIAPPSHLIRVEGNNHAQYVEDSITGRQSVLVPYEPPQVGTEFTTILYNFMCNSSCVGGMNRRPILIIVTLETRDGQVLGRRCFEARICACPGRDRKADEDSIRKQHVTDATKSSEAFRQVSHGIQMSTIKKRRSTDEEVFCLPIKGREIYEILVKIKESLELMQFLPQHTIESYRQQQQNLLQKQTSMPSQPSYGSSSPTHGKVNKLPSVSQLINPQQRNTLTPSSMSGGLTDMTPMMGTHIPMNADMSSLSPTHALQPQLPLVPSSHCTPPPPYPMDSSISSFLIRLGCSGCLDYFTAQGLTNIYQIENYNMEDLSRLKIPAEFQHIIWKGIMEHRQAMDFSPPPHIVRTTSGASSVSVGASEARGERVIDAVRFTLRQTISFPPRDEWSDFSFDLDSRRNKQQRIKEEGE

>XP_040896914.1 tumor protein 63 isoform X1 [Toxotes jaculatrix]

MNSPYTAVQYYPEFPFHRLRDPPSRLSCRETSFLTTMSQNQSAQTNDFFSQDVFNQLFDMLDQSAIHSVQPIELNFTDSPTDGSAGNTIQISMDCITMHEPDETLSSQYTNLGLLNSMDQNIQNGGSTSTSPYNNDHAQNNVTAPSPYAQPSSTFDALSPSPAIPSNTDYAGPHTFDVSFQQSSTAKSATWTYSTELKKLYCQIAKTCPIQIKVLTTPPQGAVIRAMPVYKKAEHVTEVVKRCPNHELSREFNDGQIAPPSHLIRVEGNSHAQYVEDSITGRQSVLVPYEPPQVGTEFTTILYNFMCNSSCVGGMNRRPILIIVTLETRDGQVLGRRCFEARICACPGRDRKADEDSIRKQHVTDATKSSEGTKRPFRQVSHGIQMSTIKKRRSTDEEVFCLPIKGREIYEILVKIKESLELMQFLPQHTIESYRQQQQNLLQKQTSMPPQPSFGSSSPTHGKVNKLPSVSQLMNPQQRNTLTPSSMSGGLTDMTPMMGTHIPMNADMSSLSPTHALQPQLPLVPSSHCTPPPPYPMDSSISSFLIRLGCAGCLDYFTAQGLTNIYQIENYNMEDLSRLKIPAEFQHIIWKGIMEHRQAMDFSPPPHIVRTTSGASTVSVGSSEARGERVIDAVRFTLRQTISFPPRDEWSDFSFDLDSRRNKQQRIKEEGE

>XP_026160611.1 tumor protein 63 isoform X4 [Mastacembelus armatus]

MNSPYTAVQCYPEFTFHRLRDPSSCLSWRESSFLTAMSQNQSAPTTDFFSQDVFNQLFDMLDQSAIHSVQPIELNFTDSPTDGSAGNTIQISMDCITMHEPDETLSSQYTNLGLLNSMDQNIQNGGSTSTSPYNNDHAQNNVTAPSPYAQPSSTFDALSPSPAIPSNTDYAGPHTFDVSFQQSSTAKSATWTYSTELKKLYCQIAKTCPIQIKVLTNPPQGAVIRAMPVYKKAEHVTEVVKRCPNHELSREFNDGQIAPPSHLIRVEGNSHAQYVEDSITGRQSVLVPYEPPQVGTEFTTILYNFMCNSSCVGGMNRRPILIIVTLETRDGQVLGRRCFEARICACPGRDRKADEDSIRKQHVTDATKSSEGTKRPFRQVSHGIQMSTIKKRRSTDEEIFCLPIKGREIYEILVKIKESLELMQFLPQHTIESYRQQQQTLLQKQTSVPSQPSFGSSSPTHGKVNKLPSVSQLINPQQRNTLTPSSMSGGLTDMTPMMGTHIPMNTDMSSLSPTHSLQPQLPLVPSSHCTPPPPYPMDSSISSFLIRLGCAGCLDYFTAQGLTNIYQIENYNMEDLSRLKIPAEFQHIIWKGIMEHRQAMDFSPPPHIVRTTSGASTVSVGSSEARGERVIDAVRFTLRQTISFPPRDEWSDFSFDLDSRRNKQQRIKEEGE

>XP_020465634.1 tumor protein 63 isoform X2 [Monopterus albus]

MSQNQSQTADFFSQDVFNQLFDMLDQSAIHSVQPIELNFTEGPTDGSAGNTIQISMDCITMHEQDEMLSSQYTNLGLLNSMDQNIQNGGSTSTSPYNNDHSQNTVTAPSPYAQPSSTFDALSPSPAIPSNTDYAGPHTFDVSFQQSSTAKSATWTYSTELKKLYCQIAKTCPIQIKVLTPPPQGAVIRAMPVYKKAEHVTEVVKRCPNHELSREFNDGQIAPPSHLIRVEGNSHAQYVEDSITGRQSVLVPYEPPQVGTEFTTILYNFMCNSSCVGGMNRRPILIIVTLETRDGQVLGRRCFEARICACPGRDRKADEDSIRKQHVTDATKSSDAFRQVSHGIQMSTIKKRRSTDEEVFCLPIKGREIYEILVKIKESLELMQFLPQHTIESYRQQQQNLLQKQTSMPSQPSFGSSSPTHGKVNKLPSVSQLINPQQRNTLTPSSMSGGLTDMTPMMGTHIPMNTDMSSLSPTHTLQPQLPLVPSSHCTPPPPYPMDSSISSFLIRLGCAGCLDYFTAQGLTNIYQIENYNMEDLSRLKIPAEFQHIIWKGIIEHRQAMDFSPPPHIVRTTSGASTVSVGASEARGERVIDAVRFTLRQTISFPPRDEWSDFSFDLDSRRNKQQRIKEEGE

>XP_039544359.1 tumor protein 63-like isoform X1 [Pimephales promelas]

MLYLETNQPTSYSEPQYTSLGLLNSMDQNGGSTSTSPYNNDHAQNNVTAPSPYAQPSSTFEALSPSPAIPSNTDYAGPHTFDVSFQQSSTAKSATWTYSTELKKLYCQIAKTCPIQIKVLTNPPQGAVIRAMPVYKKAEHVTEVVKRCPNHELSREFNDGQIAPPSHLIRVEGNSHAQYMEDSITGRQSVLVPYEPPQVGTEFTTILYNFMCNSSCVGGMNRRPILIIVTLESRDGQVLGRRCFEARICACPGRDRKADEDSIRKQHVSDGTKSSEGMKRPFRQASHLSQHPSIKKRRSTDEEVFCLPIKGREIYEILVKIKESLELMQFLPQHTIESYRQQQQNLLQKQSSLPPQPNFGSSSPTLGKNKLPSVSQLINPQQRNTLTPSSMAGGLTDSLLQSQHFPLPSVTPPMMGGPVPMNTDMSGLSPTNPLQSQLQMVPSSHCTPPPPYPMDNSISSFLLRLGCSACLDYFTAQGLTNIYQIDNYNLEDLSRLKIPTEFQHIIWKGIMEYRQTMEFSPPPHILRTSSGTSTVSVGSTEARGERVIDAVRFTLRQTISFPPRDDWTDFSFDLAPDSRRNKQQRIKEEGE

>XP_039985174.1 tumor protein 63 isoform X1 [Xiphias gladius]

MNSPYTAMQYYPEFPFHRLRDPPSRLSWRESSFLTTMSQNPSAQTTDFFSQDVFNQLFDMLDQSAIHSVQPIELNFTDSPTDGSAGNTIQISMDCITMHEPDETLSSQYTNLGLLNSMDQNIQNGGSTSTSPYNNDHAQNSVTAPSPYAQPSSTFDALSPSPAIPSNTDYAGPHTFDVSFQQSSTAKSATWTYSTELKKLYCQIAKTCPIQIKVLTTPPQGAVIRAMPVYKKAEHVTEVVKRCPNHELSREFNDGQIAPPSHLIRVEGNSHAQYLEDSITGRQSVLVPYEPPQVGTEFTTILYNFMCNSSCVGGMNRRPILIIVTLETRDGQVLGRRCFEARICACPGRDRKADEDSIRKQHVTDATKSSEGTKRPFRQVSHGIQMSTIKKRRSTDEEVFCLPIKGREIYEILVKIKESLELMQFLPQHTIESYRQQQQNLLQKQTSMPSQPSFGSSSPTHGKVNKLPSVSQLINPQQRNTLTPSSMSGGLTDMTPMMGTHIPMNADMSSLSPTHALQPQLPLVPSSHCTPPPPYPMDSSISSFLIRLGCAGCLDYFTAQGLTNIYQIENYNMEDLSRLKIPAEFQHIIWKGIMEHRQAMDFSPPPHIVRTTSGASTVSVGSSEARGERVIDAVRFTLRQTISFPPRDEWSDFSFDLDSRRNKQQRIKEEGE

>XP_035813650.1 tumor protein 63 isoform X1 [Amphiprion ocellaris]

MNSPYTAVQYYPEFPFHRLRDPSSRLSWRESSFLTTMSQNQSAQTNDFFSQDVFNQLFDMLDQSALHSVQPIELNFTEGPTDGSAGNTIQISMDCITMHEPDEVHSSQYTNLGLLNSMDQNIQNGGSTSTSPYNNDHAQNNVTAPSPYAQPSSTFDALSPSPAIPSNTDYAGPHTFDVSFQQSSTAKSATWTYSTDLKKLYCQIAKTCPIQIKVLTTPPQGAVIRAMPVYKKAEHVTEVVKRCPNHELSREFNDGQIAPPSHLIRVEGNSHAQYVEDSITGRQSVLVPYEPPQVGTEFTTILYNFMCNSSCVGGMNRRPILIIVTLETRDGQVLGRRCFEARICACPGRDRKADEDSIRKQHVTDATKSSDGTKRPYRHVSQGIQMSTIKKRRSTDEEVFCLPIKGREIYEILVKIKESLELMQFLPQHTIESYRQQQQNLLQKQTSMQSQPSFGSSSPTHGKVNKLPSVSQLINPQQRNTLTPSSMSGGLTDMTPMMGTHIPMNADMSSLSPTHALQPQLPLVPSSHCTPPPPYPMDSSISSFLIRLGCAGCLDYFTAQGLNNIYQIENYNLEDLSRLKIPAEFQHIIWKGIMEHRQAMDFSPPPHIVRTTSGASTVSVGSSEARGERVIDAVRFTLRQTISFPPRDEWSDFSFDLDSRRNKQQRIKEEGE

>XP_036969889.1 tumor protein 63 isoform X1 [Acanthopagrus latus]

MFTARFYLYIRRLNKLQQAQLCRRNDRLRDPPSRLSWREGSFLSTMSQNQSAQTTDSFSQDVFSQLFDMLDQSAIHSVQPIELNFTDSPTDGSAGNTIQISMDCITMHEPDETLSSQYTNLGLLNSMDQNIQNGGSTSTSPYNNDHAQNNVTAPSPYAQPSSTFDALSPSPAIPSNTDYAGPHTFDVSFQQSSTAKSATWTYSTELKKLYCQIAKTCPIQIKVLTNPPQGAVVRAMPVYKKAEHVTEVVKRCPNHELSREFNDGQIAPPSHLIRVEGNNHAQYLEDTITGRQSVLVPYEPPQVGTEFTTILYNFMCNSSCVGGMNRRPILIIVTLETRDGQVLGRRCFEARICACPGRDRKADEDSIRKQHVTDATKSSEGTKRPFRQVSHGIQMSTIKKRRSTDEEVFCLPIKGREIYEILVKIKESLELMQFLPQHTIESYRQQQQNLLQKQTSVPSQPSYGSCSPTHGKVNKLPSVSQLMNPQQRNTLTPSSMSGGLTDMSPMMGTHIPMNADMSSLSPTHALQSQLPLVPSSHCTPPPPYPMDSSISSFLIRLGCAGCLDYFTAQGLTNIYQIENYNMEDLSRLKIPTEFQHIIWKGIMEHRQAMDFSPPPHIVRTTSGASTVSMGASEARGERVIDAVRFTLRQTISFPPRDEWSDFSFDLDSRRNKQQRIKEEGE

>XP_034036552.1 tumor protein 63 isoform X5 [Thalassophryne amazonica]

MSQNQSAQTTDLFSQDVFNQLFDMLDQSSIHSVQPIELNFTDSLRDGSAGNTIQISMDCITMHDPDDTLSSQYTNLGLLNSMDQSMQNGGSTSTSPYNNDHAQNNVTAPSPYAQPSSTFDALSPSPAIPSNTDYAGPHTFDVSFQQSSTAKSATWTYSTELKKLYCQIAKTCPIQIKVLTTPPQGAVIRAMPIYKKAEHVTEVVKRCPNHELSREFNDGQIAPPSHLIRVEGNSHAQYVEDSITGRQSVLVPYEPPQVGTEFTTILYNFMCNSSCVGGMNRRPIVIIVTLETRDGQVLGRRCFEARICACPGRDRKADEDSIRKQHVTDATKSSDAFRQVSHGIQVSSIKKRRSTDEEVFCLPIKGREIYEILVKIKESLELMQFLPQHTIESYRQQQQNLLQKQTPMQSQPSFGSSSPTHGKVNKLPSVSQLINPQQRNTLTPSSMSGGLTDMTPMMGTHIPMSADMSSLSPTLQPQLPLVPSSHCTPPPPYPMDSSISSFLIRLGCAGCLDYFTAQGLTNIYQIENYNMEDLSRLKIPAEFQHIIWKGIMEHRQAMDFSPPPHIVRTTSGASTVSVGAAEARGERVIDAVRFTLRQTISFPPRDEWSDFSFDLDSRRNKQQRIKEEGE

>XP_019207222.1 tumor protein 63 isoform X1 [Oreochromis niloticus]

MSRACTGTGPSLRHQISANGKRLLTPPPISSATDAVDLLHRLRDPSARLSWREGSFLTTMSQNQAAQTTDLFSQDVFNQLFDMLDQSAIHSVQPIELNFSDSPADGSAGNTIQISMDCITMREPDEPLSSQYTNLGLLNGMDQNIQNGGSTSTSPYNNDHAQNNVTAPSPYAQPSSTFDALSPSPAIPSNTDYAGPHTFDVSFQQSSTAKSATWTYSTELKKLYCQIAKTCPIQIKVLTTPPQGAVIRAMPVYKKAEHVTEVVKRCPNHELSREFNDGQIAPPSHLIRVEGNSHAQYVEDSITGRQSVLVPYEPPQVGTEFTTILYNFMCNSSCVGGMNRRPILIIVTLETRDGQVLGRRCFEARICACPGRDRKADEDSIRKQHVTDATKSSEGTKRPFRQVSHGIQMSTIKKRRSTDEEVFCLPIKGREIYEILVKIKESLELMQFLPQHTIESYRQQQQNLLQKQTSMSSQPSFGSTSPTPGKVNKLPSVSQLINPQQRNTLTPSSMSGGLTDMTPMMGTHIPMNADMSSLSPTHALQPQLPLVPSSHCTPPPPYPMDSSISSFLIRLGCAGCLDYFTTQGLTNIYQIENYNMEDLSRLKIPAEFQHIIWKGIMEHRQAMDFSPPPHIVRTTGSASTVSVGSSEARGERVIDAVRFTLRQTISFPPRDEWSDFSFDLDSRRNKQQRIKEEGE

>XP_028856128.1 tumor protein 63 isoform X3 [Denticeps clupeoides]

MTSPYAAVQYCPEHAFQRRLRDPPSSCLSWAESSLLAAMSQSQSSQATDILSQDVVNHLLEILDQSAFHSVQPIEFNISETPANGSASSTIQISMDCITMRGPEDPFTSQYTNLGLLNSMDQNMQNGGSTSTSPYNNEHAQNNVTAPSPYAPPSSTFETLSPSPAIPSNTDYAGPHTFDVSFQQSSTAKSATWTYSTDLKKLYCQIAKTCPIQIKVLTNPPQGAVIRAMPVYKKAEHVTEVVKRCPNHELSREFNDGQIAPPSHLIRVEGNSHAQYVEDSITGRQSVLVPYEPPQVGTEFTTILYNFMCNSSCVGGMNRRPILIIVTLETRDGQVLGRRCFEARICACPGRDRKADEDSIRKQHVSDGTKSNEGTKRPFRQMPHSTQMSCGKKRRSTDEEVFCLPIKGREIYEILVKIKESLELMQFLPQHTIETYRQQQQSLLQKQSPIPSQPTYGSSSPPQSKNKLPSVSQLINPQQRNTLTPVSVPAGLTDMTPLMSAHLTGMNTDMSPLSPSHALQSQLPMVASSHCTPPPPYPVDSSISSFLLRLGCSACLDYFTAQGLTNIYQIENYNIEDLSRLKIPTDFQHIIWKGIMEHRQTMEFSPPPHILRTSSGASTVSVGSSEARGERVIDAVRFTLRQTISFPPRDDWTDFSFDLDSRRNKQQRIKEEGE

**>XP_029684427.1 tumor protein 63 isoform X1 [Takifugu rubripes]**

MNSPYAAVQCYPEFPFHRLRDPASRLSWRDSSFLTTMSHNQSSQTSDSFSQDVFNQLFDMLDQSAIHSVQPIELNFTDSPRDGSAGNTIQISMDCITMHEPEDTFTSQYTNLGLLNSMDQNIQNGGSTSTSPYNNDHAQNNVTAPSPYAQPSSTFDALSPSPAIPSNTDYAGPHTFDVSFQQSSTAKSATWTYSTELKKLYCQIAKTCPIQIKVLTTPPQGAVVRAMPVYKKAEHVTEVVKRCPNHELSREFNDGQMAPPSHLIRVEGNNHAQYVEDTITGRQSVLVPYEPPQVGTEFTTILYNFMCNSSCVGGMNRRPILIIVTLETRDGQVLGRRCFEARICACPGRDRKADEDSIRKQHVTDATKSSEGTKHPFRQVSHGIQMSTIKKRRSTDEEVFCLPIKGREIYEILVKIKESLELMQFLPQHTIESYRQQQQNLLQKQTSVPSQPSYGSCSPTHGKVNKLPSVSQLMNPQQRNTLTPSSMSGGLTDMSPMMGTHVPMNADMSSLSPTHALQQQLPLVPSSHCTPPPPYPMDSSISSFLLRLGCAGCLDYFTAQGLTNIYQIENYNMEDLSRLKIPSEFQHIIWKGIMEHRQAMDFSPPPHIVRTTSGASSVSVGATEARGERVIDAVRFTLRQTISFPPRDEWSDFSFDLDSRRNKQQRIKEEGE

>TWW57489.1 Tumor protein 63 [Takifugu flavidus]

MMVAVLPLVCRLRDPASRLSWRDSSFLTTMSHNQSSQTSDSFSQDVFNQLFDMLDQSAIHSVQPIELNFTDSPRDGSAGNTIQISMDCITMHEPEDTFTLRGNNSAQRLLRLQESQLSSIMEQTAAALLNLSANMLYLETGTTTSYSESQYTNLGLLNSMDQNIQNGGSTSTSPYNNDHAQNNVTAPSPYAQPSSTFDALSPSPAIPSNTDYAGPHTFDVSFQQSSTAKSATWTYSTELKKLYCQIAKTCPIQIKVLTTPPQGAVVRAMPVYKKAEHVTEVVKRCPNHELSREFNDGQMAPPSHLIRVEGNNHAQYVEDTITGRQSVLVPYEPPQVGTEFTTILYNFMCNSSCVGGMNRRPILIIVTLETRDGQVLGRRCFEARICACPGRDRKADEDSIRKQHVTDATKSSEAFRQVSHGIQMSTIKKRRSTDEEVFCLPIKGREIYEILVKIKESLELMQFLPQHTIESYRQQQQNLLQKQTSVPSQPSYGSCSPTHGKVNKLPSVSQLMNPQQRNTLTPSSMSGGLTDMSPMMGTHVPMNADMSSLSPTHALQQQLPLVPSSHCTPPPPYPMDSSISSFLLRLGCAGCLDYFTAQGLTNIYQIENYNMEDLSRLKIPSEFQHIIWKGIMEHRQAMDFSPPPHIVRTTSGASSVSVGATEARGERVIDAVRFTLRQTISFPPRDEWSDFSFDLDSRRNKQQRIKEEGE

>XP_038572596.1 tumor protein 63 isoform X1 [Micropterus salmoides]

MNSPYTAVQYYPEFPFHRLRDPPSRLSWRESSFLTTMSQNQSAQTTDSFSQDVFNQLFDMLDQSAIHSVQPIELNFTDSPIDGSAGNTIQISMDCITMHEPDETLTSQYTNLGLLNSMDQNIQNGGSTSTSPYNNDHAQNNVTAPSPYAQPSSTFDALSPSPAIPSNTDYAGPHTFDVSFQQSSTAKSATWTYSTELKKLYCQIAKTCPIQIKVLTTPPQGAVIRAMPVYKKAEHVTEVVKRCPNHELSREFNDGQIAPPSHLIRVEGNNHAQYVEDTITGRQSVLVPYEPPQVGTEFTTILYNFMCNSSCVGGMNRRPILIIVTLETRDGQVLGRRCFEARICACPGRDRKADEDSIRKQHVTDATKGNDGMKRPFRQVSHGIQMSTIKKRRSTDEEVFCLPIKGREIYEILVKIKESLELMQFLPQHTIESYRQQQQNLLQKQTSMPSQPSYGSCSPTHGKVNKLPSVSQLMNPQQRNTLTPSSMSGGLTDMTPLMGTHIPMNADMSPLSPTHALQPQLPLVPSSHCTPPPPYPMDSSISSFLIRLGCTGCLDYFTAQGLTNIYQIENYNMEDLSRLKIPAEYQHIIWKGIMEHRQAMDFSPPPHIVRTTSGASTVSVGSSEARGERVIDAVRFTLRQTISFPPRDEWSDFSFDLDSRRNKQQRIKEEGE

>XP_035508762.1 tumor protein 63 isoform X1 [Morone saxatilis]

MNSPYTAVQYYPEFPFHRLRDPSSRLSWRESSFLTTMSQNQSAQTTDSFSQDVFNQLFDMLDQSAIHSVQPIELNFTDSPADGSAGNTIQISMDCITMHEPDETLSSQYTNLGLLNSMDQNIQNGGSTSTSPYNNDHAQNNVTAPSPYAQPSSTFDALSPSPAIPSNTDYAGPHTFDVSFQQSSTAKSATWTYSTDLKKLYCQIAKTCPIQIKVLTTPPQGAVIRAMPVYKKAEHVTEVVKRCPNHELSREFNDGQIAPPSHLIRVEGNNHAQYVEDTITGRQSVLVPYEPPQVGTEFTTILYNFMCNSSCVGGMNRRPILIIVTLETRDGQVLGRRCFEARICACPGRDRKADEDSIRKQHVTDATKSSDGMKRPFRQVSHGIQMSTIKKRRSTDEEVFCLPIKGREIYEILVKIKESLELMQFLPQHTIESYRQQQQNLLQKQTSMPSQPSYGSCSPTHGKVNKLPSVSQLMNPQQRNTLTPSSMSGGLTDTPFVSAVSPMMGTHIPMNADMSSLSPTHALQPQLPMVPSSHCTPPPPYPMDSSISSFLIRLGCAGCLDYFTAQGLSNIYQIENYNMEDLSRLKIPAEFQHIIWKGIMEHRQAMDFSPPPHIVRTTSGASTVSVGSSEARGERVIDAVRFTLRQTISFPPRDEWSDFSFDLDSRRNKQQRIKEEGE

>XP_030289528.1 tumor protein 63 isoform X1 [Sparus aurata]

MNSPYTAVQYYPEFPFRRLRDPPSRLSWREGSFLSTMPQNQSAQTTDSFSQDVFSQLFDMLDQSAIHSVQPIELNFTDSPTDGSAGNTIQISMDCITMHEPDETLSSQYTNLGLLNSMDQNIQNGGSTSTSPYNNDHAQNNVTAPSPYAQPSSTFDALSPSPAIPSNTDYAGPHTFDVSFQQSSTAKSATWTYSTELKKLYCQIAKTCPIQIKVLTNPPQGAVVRAMPVYKKAEHVTEVVKRCPNHELSREFNDGQIAPPSHLIRVEGNNHAQYLEDTITGRQSVLVPYEPPQVGTEFTTILYNFMCNSSCVGGMNRRPILIIVTLETRDGQVLGRRCFEARICACPGRDRKADEDSIRKQHVTDATKSSEGTKRPFRQVSHGIQMSTIKKRRSTDEEVFCLPIKGREIYEILVKIKESLELMQFLPQHTIESYRQQQQNLLQKQWDGNGLSPPLSHPRTSVPSQPSYGSCSPTHGKVNKLPSVSQLMNPQQRNTLTPSSMSGGLTDMSPMMGTHIPMNADMSSLSPTHALQSQLPLVPSSHCTPPPPYPMDSSISSFLIRLGCAGCLDYFTAQGLTNIYQIENYNMEDLSRLKIPTEFQHIIWKGIMEHRQAMDFSPPPHIVRTTSGASTVSMGASEARGERVIDAVRFTLRQTISFPPRDEWSDFSFDLDSRRNKQQRIKEEGE

>XP_020487257.1 tumor protein 63 [Labrus bergylta]

MNSPYTAVQYYPEFAFHRLRDSSSRLSWRESSFLAAMSQNQSAQTSDYFSQDVFNQLFDMLDQSAIHSVQPIELNFTDSPTDGSTGNTIQISMDCITMHEPDESLSSQYTNLGLLNSMDQNIQNGGSTSTSPYNNDHAQNNVTAPSPYAQPSSTFDAMSPSPAIPSNTDYAGPHTFDVSFQQSSTAKSATWTYSTDLKKLYCQIAKTCPIQIKVLTTPPQGAVIRAMPVYKKAEHVTEVVKRCPNHELSREFNDGQIAPPSHLIRVEGNNHAQYLEDTITGRQSVLVPYEPPQVGTEFTTILYNFMCNSSCVGGMNRRPILIIVTLETRDGQVLGRRCFEARICACPGRDRKADEDSIRKQHVTDATKSNDAFRQVSHGIQMSAIKKRRSTDEEVFCLPIKGREIYEILVKIKESLELMQFLPQHTIESYRQQQQNLLQKQTSMPSQPPYGSSSPTHGKVNKLPSVSQLMNPQQRNTLTPSGMSGGLTDMSPMMGTHIPMNDMSSLSPTHALQQQLPLVPSSHCTPPPPYPMDSSISSFLIRLGCAGCLDYFTAQGLSNIYQIENYNMEDLSRLKIPVEFQHIIWKGIIEHRQAMDFSPPPHIVRTTSGASAVSMGSSEARGERVIDAVRFTLRQTISFPPRDEWSDFSFDLDSRRNKQQRIKEEGE

>XP_033478121.1 tumor protein 63 isoform X1 [Epinephelus lanceolatus]

MNSPYTAVQYYPEFPFHRLRDPSSRLSWRESSFLTAMSQNQSAQTSDFFSQDVFNQLFDMLDQSAIHSVQPIELNFRDSPTDGSAGNTIQISMDCITMHEADETLASQYTNLGLLNSMDQNIQNGGSTSTSPYNNDHAQNNVTAPSPYAQPSSTFDALSPSPAIPSNTDYAGPHTFDVSFQQSSTAKSATWTYSTDLKKLYCQIAKTCPIQIKVLTNPPQGAVIRAMPVYKKAEHVTEVVKRCPNHELSREFNDGQIAPPSHLIRVEGNNHAQYVEDSITGRQSVLVPYEPPQVGTEFTTILYNFMCNSSCVGGMNRRPILIIVTLETRDGQVLGRRCFEARICACPGRDRKADEDSIRKQHVTDATKSSEGTKRPFRQVSHGIQMSAIKKRRSTDEEVFCLPIKGREIYEILVKIKESLELMQFLPQHTIESYRQQQQNLLQKQTSMPSQPSYGSCSPTQPGKVNKLPSVSQLINPQQRNTLTPSSMTGGLTDMTPMMGTHIPMNDMSSLSPTHALQQQLPLVPSSHCTPPPPYPMDSSISSFLIRLGCAGCLDYFTAQGLTNIYQIENYNMEDLSRLKIPAEFQHIIWKGIMEHRQAMDFSPPPHIVRTTSGASTVSVGSSEARGERVIDAVRFTLRQTISFPPRDEWSDFSFDLDSRRNKQQRIKEEGE

>XP_019130908.1 tumor protein 63 isoform X1 [Larimichthys crocea]

MNLPYTAVQHYPEFPFHRLRDPSRLSWRESSFLTAMSQNQSQTPECFSQDVFNQLFDMLDQSAIHSVQPIELNFTDSPTDGSAGNTIQISMDCITMHEPDETLSSQYTNLGLLNSMDQNIQNGGSTSTSPYNNDHAQNNVTAPSPYAQPSSTFDALSPSPAIPSNTDYAGPHTFDVSFQQSSTAKSATWTYSTDLKKLYCQIAKTCPIQIKVLTTPPQGAVIRAMPVYKKAEHVTEVVKRCPNHELSREFNDGQIAPPSHLIRVEGNNHAQYVEDTITGRQSVLVPYEPPQVGTEFTTILYNFMCNSSCVGGMNRRPILIIVTLETRDGQVLGRRCFEARICACPGRDRKADEDSIRKQHVTDATKSSEGTKRPFRQASHGIQMSTIKKRRSTDEEVFCLPIKGREIYEILVKIKESLELMQFLPQHTIESYRQQQQNLLQKQTSMPSQPSYGSCSPTHGKVNKLPSVSQLMNPQQRNTLTPSSMSGGLTDTPFFSAVSPMMGTHLPMNADMSSLSPTHALQPQLPLVPSSHCTPPPPYPMDSSISSFLIRLGCAGCLDYFTAQGLTNIYQIENYNMEDLSRLKIPSEFQHIIWKGIMEHRQAMDFSPPPHIVRTTSGASTVSVGSSEARGERVIDAVRFTLRQTISFPPRDEWSDFSFDLDSRRNKQQRIKEEGE

>XP_034736309.1 tumor protein 63 isoform X1 [Etheostoma cragini]

MNSPYTAVQCYPEFPFHRLRDPSSRLSWKESSFLTTMSQNQSAQTTDIFSQDVFNQLFDMLDQSAIHSAQPIELNFTDSPTDVSAGNTIQISMDCITMHEPDETLTSQYTNLGLLNSMDQNIQNGGSTSTSPYNNDHAQNNVTAPSPYAQPSSTFDALSPSPAIPSNTDYAGTHTFDVSFQQSSTAKSATWTYSTDLKKLYCQIAKTCPIQIKVLTNPPQGAVIRAMPVYKKAEHVTEVVKRCPNHELSREFNDGQIAPPSHLIRVEGNNHAQYVEDSITGRQSVLVPYEPPQVGTEFTTILYNFMCNSSCVGGMNRRPILIIVTLETRDGQVLGRRCFEARICACPGRDRKADEDSIRKQHVTDGTKSNDGTKRPFRQVSHGIQMSTIKKRRSTDEEVFCLPIKGREIYEILVKIKESLELMQFLPQHTIESYRQQQQNLLQKQTSMQSQPSYGSCSPTHGKVNKLPSVSQLINPQQRNTLTPSSMAGGLTDMTPMMGTHIPMNDMSSLSPTHALQPQLPMVPSSHCTPPPPYPMDSSISSFLIRLGCAGCLDYFTAQGLTNIYQIENYNMEDLSRMKIPVEFQHIIWKGIMEHRQAMDFSPPPHIVRTTSGASTVSMGSSEARGERVIDAVRFTLRQTISFPPRDEWSDFSFDLDSRRNKQQRIKEEGE

>XP_028442849.1 tumor protein 63 isoform X1 [Perca flavescens]

MNSPYTAVQYYPEFPFHRLRDPSSRLSWRESSFLTTMSQNQSAQTTDFFSQDVFNQLFDMLDQSAIHSAQPIELNFKDSPTDVSAGNTIQISMDCITMHEPDEMLTSQYTNLGLLNSMDQNIQNGGSTSTSPYNNDHAQNTVTAPSPYAQPSSTFDALSPSPAIPSNTDYAGTHTFDVSFQQSSTAKSATWTYSTDLKKLYCQIAKTCPIQIKVLTNPPQGAVIRAMPVYKKAEHVTEVVKRCPNHELSREFNDGQIAPPSHLIRVEGNNHAQYVEDSITGRQSVLVPYEPPQVGTEFTTILYNFMCNSSCVGGMNRRPILIIVTLETRDGQVLGRRCFEARICACPGRDRKADEDSIRKQHVTDGTKSNDAFRQVSHGIQMSTIKKRRSTDEEVFCLPIKGREIYEILVKIKESLELMQFLPQHTIESYRQQQQNLLQKQTSMQSQPPYGSCSPTHGKVNKLPSVSQLINPQQRNTLTPSSMAGGLTDMTPMMGTHIPMNDMSSLSPTHALQPQLPMVPSSHCTPPPPYPMDSSISSFLIRLGCAGCLDYFTAQGLTNIYQIENYNMEDLSRMKIPVEFQHIIWKGIMEHRQAMDFSPPPHIVRTTSGASTVSMGSSEARGERVIDAVRFTLRQTISFPPRDEWSDFSFDLDSRRNKQQRIKEEGE

>XP_031167634.1 tumor protein 63 isoform X1 [Sander lucioperca]

MNSPYTAVQYYPEFPFHRLRDPSSRLSWRESSFLTTMSQNQSAQTTDFFSQDVFNQLFDMLDQSAIHSAQPIELNFKDSPTDVSAGNTIQISMDCITMHEPDETLTSQYTNLGLLNSMDQNIQNGGSTSTSPYNNDHAQNNVTAPSPYAQPSSTFDALSPSPAIPSNTDYAGTHTFDVSFQQSSTAKSATWTYSTDLKKLYCQIAKTCPIQIKVLTNPPQGAVIRAMPVYKKAEHVTEVVKRCPNHELSREFNDGQIAPPSHLIRVEGNNHAQYVEDSITGRQSVLVPYEPPQVGTEFTTILYNFMCNSSCVGGMNRRPILIIVTLETRDGQVLGRRCFEARICACPGRDRKADEDSIRKQHVTDGTKSNDGTKRPFRQVSHGIQMSTIKKRRSTDEEVFCLPIKGREIYEILVKIKESLELMQFLPQHTIESYRQQQQNLLQKQASMQSQPSYGSCSPTHGKVNKLPSVSQLINPQQRNTLTPSSMAGGLTDMTPMMGTHIPMNDMSSLSPTHALQPQLPMVPSSHCTPPPPYPMDSSISSFLIRLGCAGCLDYFTAQGLTNIYQIENYNMEDLSRMKIPVEFQHIIWKGIMEHRQAMDFSPPPHIVRTTSGASTVSMGSSEARGERVIDAVRFTLRQTISFPPRDEWSDFSFDLDSRRNKQQRIKEEGE

>XP_039667746.1 tumor protein 63 isoform X1 [Perca fluviatilis]

MNSPYTAVQYYPEFPFHRLRDPSSRLSWRESSFLTTMSQNQSAQTTDFFSQDVFNQLFDMLDQSAIHSAQPIELNFKDSPTDVSAGNTIQISMDCITMHEPDETLTSQYTNLGLLNSMDQNIQNGGSTSTSPYNNDHAQNNVTAPSPYAQPSSTFDALSPSPAIPSNTDYAGTHTFDVSFQQSSTAKSATWTYSTDLKKLYCQIAKTCPIQIKVLTNPPQGAVIRAMPVYKKAEHVTEVVKRCPNHELSREFNDGQIAPPSHLIRVEGNNHAQYVEDSITGRQSVLVPYEPPQVGTEFTTILYNFMCNSSCVGGMNRRPILIIVTLETRDGQVLGRRCFEARICACPGRDRKADEDSIRKQHVTDGTKSNDGTKRPFRQVSHGIQMSTIKKRRSTDEEVFCLPIKGREIYEILVKIKESLELMQFLPQHTIESYRQQQQNLLQKQTSMQSQPPYGSCSPTHGKVNKLPSVSQLINPQQRNTLTPSSMAGGLTDMTPMMGTHIPMNDMSSLSPTHALQPQLPMVPSSHCTPPPPYPMDSSISSFLIRLGCAGCLDYFTAQGLTNIYQIENYNMEDLSRMKIPVEFQHIIWKGIMEHRQAMDFSPPPHIVRTTSGASTVSMGSSEARGERVIDAVRFTLRQTISFPPRDEWSDFSFDLDSRRNKQQRIKEEGE

>TKS74819.1 Tumor protein 63 [Collichthys lucidus]

MRRLLNEYFKGKLKPKSVEQRQRLRDPSRLSWRESSFLTAMSQNQSQTPECFSQDVFNQLFDMLDQSAIHSVQPIELNFTDSPTDGSAGNTIQISMDCITMHEPDETLSVMEQTAAALLNLSANMLYLETGTTTPYSESQYTNLGLLNSMDQNIQNGGSTSTSPYNNDHAQNNVTAPSPYAQPSSTFDALSPSPAIPSNTDYAGPHTFDVSFQQSSTAKSATWTYSTDLKKLYCQIAKTCPIQIKVLTTPPQGAVIRAMPVYKKAEHVTEVVKRCPNHELSREFNDGQIAPPSHLIRVEGNNHAQYVEDTITGRQSVLVPYEPPQVGTEFTTILYNFMCNSSCVGGMNRRPILIIVTLETRDGQVLGRRCFEARICACPGRDRKADEDSIRKQHVTDATKSSEGTKRPFRQASHGIQMSTIKKRRSTDEEVFCLPIKGREIYEILVKIKESLELMQFLPQHTIESYRQQQQNLLQKQTSMPSQPSYGSCSPTHGKVNKLPSVSQLMNPQQRNTLTPSSMSGGLTDMSPMMGTHLPMNADMSSLSPTHALQPQLPLVPSSHCTPPPPYPMDSSISSFLIRLGCAGCLDYFTAQGLTNIYQIENYNMEDLSRLKIPSEFQHIIWKGIMEHRQAMDFSPPPHIVRTTSGASTVSVGSSEARGERVIDAVRFTLRQTISFPPRDEWSDFSFDLDSRRNKQQRIKEEGE

>XP_034438674.1 tumor protein 63 isoform X1 [Hippoglossus hippoglossus]

MNSPYPEFPLYRLRDPSSRLSWRESSFLTAMSQNQSAQTTEFFSQDVFNQLFDMLDQSAIHSVQPIELNFTDSPTDGSAGNTIQISMDCITMHKPDETLSSQYTNLGLLNSMDQSIQNGGSTSTSPYNNDHAQNNVTAPSPYAQPSSTFDALSPSPAIPSNTDYAGPHTFDVSFQQSSTAKSATWTYSTELKKLYCQIAKTCPIQIKVLTNPPQGAVIRAMPVYKKAEHVTEVVKRCPNHELSREFNDAQIAPPSHLIRVEGNSHAQYLEDTITGRQSVLVPYEPPQVGTEFTTILYNFMCNSSCVGGMNRRPILIIVTLETRDGQVLGRRCFEARICACPGRDRKADEDSIRKQHVTDATKSNDGTKRPFRQVSHGIQMSTIKKRRSTDEEVFCLPIKGREIYEILVKIKESLELMQFLPQHTIESYRQQQQNLLQKQTSMQSQPSYGSSSPTHGKVNKLPSVSQLINPQQRNTLTPSSMSGGLTDMTPMMGNHIPMNADMSSLSPTHALQQQLPLVPSSHCTPPPPYPMDSSISSFLIRLGCAGCLDYFTAQGLTNIYQIENYNMEDLSRMKIPVEFQHIIWKGIMEHRQAMDFSPPPHIVRTTSGASSVSMGSSEARGERVIDAVRFTLRQTISFPPRDEWSDFSFDLDSRRNKQQRIKEEGE

>XP_028258802.1 tumor protein 63 isoform X4 [Parambassis ranga]

MNSPYAAVQYCPEFPFHRFRDPSSRLSWRESSFLTTMSQNQSAQTNDCFSQDVFNQLFDMLDQSAIHSVQPIELNFTDSPTDGSAGNTIQISMDCITMHEQDEMLSSQYTNLGLLNSMDQSIQNGGSTSTSPYNNDHAQNNVTAPSPYAQPSSTFDALSPSPAIPSNTDYAGPHTFDVSFQQSSTAKSATWTYSTELKKLYCQIAKTCPIQIKVLTTPPQGAVIRAMPVYKKAEHVTEVVKRCPNHELSREFNDGQIAPPSHLIRVEGNSHAQYVEDTITGRQSVLVPYEPPQVGTEFTTILYNFMCNSSCVGGMNRRPILIIVTLETRDGQVLGRRCFEARICACPGRDRKADEDSIRKQNVTDVTKSSEGMKRPFRHVSQGIQMSTIKKRRSTDEEVFCLPIKGREIYEILVKIKESLELMQFLPQHTIESYRQQQQNLLQKQTSMQSQPSFGSSSPTHGKVNKLPSVSQLINPQQRNTLTPSSMSGGLTDMTPMMGTHIPMNADMSSLSPTHALQPQLPLVPSSHCTPPPPYPMDSSISSFLIRLGCAGCLDYFTAQGLTNIYQIENYNMEDLSRLKIPTDCQHIIWKGIMEHRQAMDFSPPHIVRTTSGASTVSVGTSEARGERVIDAVRFTLRQTISFPPRDEWSDFSFDLDSRRNKQQRIKEEGE

>XP_032382628.1 tumor protein 63 isoform X1 [Etheostoma spectabile]

MNSPYTAVQYYPEFPFHRLRDPSSRLSWKESSFLTTMSQNQSAQTTDIFSQDVFNQLFDMLDQSAIHSAQPIELNFKDSPTDVSAGNTIQISMDCITMHEPDETLTSQYTNLGLLNSMDQNIQNGGSTSTSPYNNDHAQNNVTAPSPYAQPSSTFDALSPSPAIPSNTDYAGTHTFDVSFQQSSTAKSATWTYSTDLKKLYCQIAKTCPIQIKVLTNPPQGAVIRAMPVYKKAEHVTEVVKRCPNHELSREFNDGQIAPPSHLIRVEGNNHAQYVEDSITGRQSVLVPYEPPQVGTEFTTILYNFMCNSSCVGGMNRRPILIIVTLETRDGQVLGRRCFEARICACPGRDRKADEDSIRKQHVTDGTKSNDGTKRPFRQVSHGIQMSTIKKRRSTDEEVFCLPIKGREIYEILVKIKESLELMQFLPQHTIESYRQQQQNLLQKQTSMQSQPSYGSCSPTHGKVNKLPSVSQLINPQQRNTLTPSSMAGGLTDMTPMMGTHIPMNDMSSLSPTHALQPQLPMVPSSHCTPPPPYPMDSSISSFLIRLGCAGCLDYFTAQGLTNIYQIENYNMEDLSRMKIPVEFQHIIWKGIMEHRQAMDFSPPPHIVRTTSGASTVSMGSSEARGERXXXXVRFTLRQTISFPPRDEWSDFSFDLDSRRNKQQRIKEEGE

>XP_034556805.1 tumor protein 63 isoform X1 [Notolabrus celidotus]

MNSPYTAVQYYPEFAFHRLRDPSSRLSWRESSFLTAMSQNQSAQTSDYYSQDVFNQLFDMLDQSAIHSVQPIELNFTESPTDRSSGNTIQISMDCITMHEPDEMRSSQYTNLGLLNSMDQSIQNSGSTSTSPYNNDHAQNNVTAPSPYAQPSSTFDALSPSPAIPSNTDYAGPHTFDVSFQQSSTAKSATWTYSTDLKKLYCQIAKTCPIQIKVLTTPPQGAVIRAMPVYKKAEHVTEVVKRCPNHELSREFNDGQIAPPSHLIRVEGNNHAQYLEDSITGRQSVLVPYEPPQVGTEFTTILYNFMCNSSCVGGMNRRPILIIVTLETRDGQVLGRRCFEARICACPGRDRKADEDSIRKQHVTDGSKSSEGTKRPFRQVSHGIQMSAIKKRRSTDEEVFCLPIKGREIYEILVKIKESLELMQFLPQHTIESYRQQQQNLLQKQTSMPSQPSYGSMSPTHGKVNKLPSVSQLMNPQQRNTLTPSGMSGGLTDNAFVSAVTPMMGSHIPMNADMSSLSPTHALQPQLPLVPSSHCTPPPPYPMDSSISSFLIRLGCAGCLDYFTAQGLTNIYQIENYNMEDLSRLKIPLEFQHMIWKGIMEHRQAMDFSPPPHIVRTTSGASAVSMGGSEARGERVIDAVRFTLRQTISFPPRDEWSDFSFDLDSRRNKQQRIKEEGE

>XP_037625660.1 tumor protein 63 isoform X1 [Sebastes umbrosus]

MTSCEDLSRCDNMNSPHTAVQYYPEFPFHRLRDTPSRLSWRESSFLTTMSQNQSAQTPDYVSQDVFNQLFEMLDQSAVHSVQPIELNFRDAPADGSPGNTIQISMDCITMHEPDQTLASQYTNLGLLNSMDQTIQNGGSTSTSPYNNDHAQNNVTAPSPYAQPSSTFDALSPSPAIPSNTDYAGTHTFDVSFQQSSTAKSATWTYSTDLKKLYCQIAKTCPIQIKVLTNPPQGAVIRAMPVYKKAEHVTEVVKRCPNHELSREFNDGQIAPPSHLIRVEGNNHAQYVEDTITGRQSVLVPYEPPQVGTEFTTILYNFMCNSSCVGGMNRRPILIIVTLETRDGQVLGRRCFEARICACPGRDRKADEDSIRKQHVTDATKSSDGMKRPFRQVSHGIQMSTIKKRRSTDEEVFCLPIKGREIYEILVKIKESLELMQYLPQHTIESYRQQQQNLLQKQTSMQSQPLYGSCSPTHGKVNKLPSVSQLINPQQRNTLTPSGMTGGLTDMTPMMGTHIPMNDMSSLSPTHVLQPQLPLMPSSHCTPPPPYPMDSSISSFLIRLGCAGCLDYFTAQGLTNIYQIENYNMEDLSRLKIPAEFQHVIWKGIMEHRQAMDFSPPPHIVRTTSGASSVSMGASEARGERVIDAVRFTLRQTISFPPRDEWSDFSFDLDSRRNKQQRIKEEGE

>XP_029355774.1 tumor protein 63 isoform X1 [Echeneis naucrates]

MFLRQLRLQRQSRLSIHQKSGAIPGSTNPHVKGSLGRVLLNDASIGRLRDRPSCLAWRESSFLTAMSQNQSGQTTDFFNQDVFNQLFDMLDQSAIHSVQPIELNFRDSPTDGSPGNTIQISMDCITMHEPDEALSSQYTNLGLLNSMDQNIQNGGSTSTSPYNNDHAQNSVTAPSPYAQPSSTFDALSPSPAIPSNTDYAGPHTFDVSFQQSSTAKSATWTYSTELKKLYCQIAKTCPIQIKVLTTPPQGAVIRAMPVYKKAEHVTEVVKRCPNHELSREFNDGQIAPASHLIRVEGNNHAQYLEDSITGRQSVLVPYEPPQVGTEFTTILYNFMCNSSCVGGMNRRPILIIVTLETRDGQVLGRRCFEARICACPGRDRKADEDSIRKQHVTDATKSSEGMKRPFRQVSHGIQMSAIKKRRSTDEEVFCLPIKGREIYEILVKIKESLELMQFLPQHTIESYRQQQQNLLQKQTSMPSQPSFGSSSPTHGKVNKLPSVSQLINPQQRNTLTPSSMSGGLTDMTPMMGTHLPMNADMSSLSPTHALQPQLPLVPSSHCTPPPPYPMDSSISSFLIRLGCAGCLDYFTAQGLTNIYQIENYNMEDLSRLKIPAEFQHIIWKGIMEHRQAMDFSPPPHIVRTTSGASTVSVGASEARGERVIDAVRFTLRQTISFPPRDEWSDFSFDLDSRRNKQQRIKEEGE

>XP_037830280.1 tumor protein 63 isoform X1 [Kryptolebias marmoratus]

MNLPFTAVQYYPEFPFHRLRDPSARLSWRESSILTAMTQNQAAQTSDFFSQDVFNQLFDMLDQSAFHSVQPIELNFTDSSTDGSAGNTIQISMDYITMHEQEQLVSSQYTNLGLLNSIEQNTGSTSTSPYNNEHTQNNVTAPSPYAQPSSTFDALSPSPAIPSNTDYAGPHTFDVSFQQSSTAKSATWTYSTDLKKLYCQIAKTCPIQIKVLTPPPQGAVIRAMPVYKKAEHVTEVVKRCPNHELSREFNDGQIAPPSHLIRVEGNSHAQYVEDSITGRQSVLVPYEPPQVGTEFTTILYNFMCNSSCVGGMNRRPILIIVTLETRDGQVLGRRCFEARICACPGRDRKADEDSIRKQHGTDATKNNEGTKRPFRQVSHGIQVSAVKKRRSTDEEVFCLPIKGREIYEILVKIKESLELMQFLPQHTIESYRQQQQNLLQKQTSMPSQPSFGSCSPTHGKVNKLPSVSHLMNPQQRNTLTPSSMSGGLTDMTPMMGSHLPMNADMSSLSPTHALQPQLPLVPSSHCTPPPPYPMDSSISSFLLRLGCAGCLDYFTAQGLTNMYQIENFNMEDLSRLKIPVEFQHIIWKGIIEHRQAMDFSPPPHIVRTTSGASTVSVGSSEARGERVIDAVRFTLRQTISFPPRDEWSDFSFDLDSRRNKQQRIKEEGE

>XP_040926158.1 tumor protein 63 isoform X1 [Betta splendens]

MTRDLPPAAHYQRALSGCSVISTFQHGSSLDDGSPAALQQFCVCSSLIRLRDPSSRLSWRESSFLTTMSQNQSAQTADLFSQDVFNQLFDMLDQSTMHSVQPIELNFTDSSTDGSAGNTIQISMDCITMHEADEMLSSQYTNLGLLNSMDQNIQNGGSTSTSPYNNDHSQNNVTAPSPYTQPSSTFDALSPSPAIPSNTDYAGPHTFDVSFQQSSTAKSATWTYSTELKKLYCQIAKTCPIQIKVLTTPPQGAVIRAMPVYKKAEHVTEVVKRCPNHELSREFNDGQIAPPSHLIRVEGNNHAQYVEDSITGRQSVLVPYEPPQVGTEFTTILYNFMCNSSCVGGMNRRPILIIVTLETRDGQVLGRRCFEARICACPGRDRKADEDSIRKQHVTDASKSSDGTKRPLRQVSHGIQMSTIKKRRSTDEEVFCLPIKGREIYEILVKIKESLELMQFLPQHTIESYRQQQQNLLQKQTSMPPQPSFGSSSPTHGKVNKLPSVSQLINPQQRNTLTPSSMSGGLTDMTPMMGTHIPMNADMSSLSPTHALQPQLPLVPSSHCTPPPPYPMDSSISSFLIRLGCAGCLDYFTAQGITNIYQIENYNMEDLSRLKIPAEFQHIIWKGIMEHRQAMDYSPPSHIVRTTSGASTVSVGSSEARGERVIDAVRFTLRQTISFPPRDEWSDFSFDLDSRRKQQRIKEEGE

>XP_023810247.1 tumor protein 63 isoform X1 [Oryzias latipes]

MNSPFQAVQYYPEFPFPHRLRDPTSRLSWRDSSFLTAMAQNQSAQSDFSSQDVFNQLFDMLDQSAIHSVQPIELNFTDSPTDGSTGNTIQISMDCITMHEPDETVSSQYTNLGLLNNMENIQSSSTSTSPYNNDHAQNNVTAPSPYAQPSSTFDALSPSPAIPSNTDYAGPHSFDVSFQQSSTAKSATWTYSTDLKKLYCQIAKTCPIQIKVLTPPPQGAVIRAMPVYKKAEHVTEVVKRCPNHELSREFNDGQIAPPSHLIRVEGNNHAQYLEDSITGRQSVLVPYEPPQVGTEFTTILYNFMCNSSCVGGMNRRPILIIVTLETRDGQVLGRRCFEARICACPGRDRKADEDSIRKQHVTDASKSSEGTKRRYRQVSHGIQMSTIKKRRSTDEEVFCLPIKGREIYEILVKIKESLELMQFLPQHTIESYRQQQQNLLQKQTSMPSQPSFGSTSPTPGKVNKLPSVSQLINPQQRNTLTPSSMTGGLTDMTPMMAPHIPMNADMSSLSPTHALQPQLPLVPSSHCTPPPPYPMDSSIASFLVRLGCAGCLDYFTAQGLSNIYQIENYNLEDLCRLKIPTEFQNIIWRGIMEHRQAMDFSPPPHIVRTTSGASTVSVGAAEARGERVIDAVRFTLRQTISFPPRDEWSDFSFDLDSRRNKQQRIKEEGE

>XP_024134201.1 tumor protein 63 isoform X1 [Oryzias melastigma]

MNSPFTAVQYYPEFPFPHRLRDPTSRLSWRDSSFLTAMAQNQSAQSDFSSQDVFNQLFDMLDQSAIHSVQPIELNFTDGPTDGSTGNTIQISMDCITMHEPDDTLSSQYTNLGLLNNMENIQNSSSTSTSPYNNDHAQNNVTAPSPYAQPSSTFDALSPSPAIPSNTDYAGPHSFDVSFQQSSTAKSATWTYSTDLKKLYCQIAKTCPIQIKVLTPPPQGAVIRAMPVYKKAEHVTEVVKRCPNHELSREFNDGQIAPPSHLIRVEGNNHAQYLEDSITGRQSVLVPYEPPQVGTEFTTILYNFMCNSSCVGGMNRRPILIIVTLETRDGQVLGRRCFEARICACPGRDRKADEDSIRKQHVTDASKSSEGTKRRYRQVSHGIQMSTIKKRRSTDEEVFCLPIKGREIYDILVKIKESLELMQFLPQHTIESYRQQQQNLLQKQNSMPSQPSFGSTSPTPGKVNKLPSVSQLINPQQRNTLTPGLTGGLTDMTPMMAPHIPMNADMSSLSPTHALQPQLPLVPSSHCTPPPPYPMDSSIASFLVRLGCAGCLDYFTAQGLTNIYQIENYNLEDLCRLKIPAEFQSIIWRGIMEHRQAMDFSPPPHIVRTTSGASTVSVGASEARGERVIDAVRFTLRQTISFPPRDEWSDFSFDLDSRRNKQQRIKEEGE

>XP_023194906.1 tumor protein 63 isoform X1 [Xiphophorus maculatus]

MNSPYAALQYYPEFPFRRLRDPSSLLSWREGSLFTTMSQPGQTADFFSQDVFNQLFDMLDQSTIHSVQPIELNFTESPTDGSAGNTIQISMDCITMHEPDDVVSSQYTNLGLLNSMEQNNGSTSTSPYNNEHAPNSVTAPSPYAQPSSTFDALSPSPAIPSNTDYAGPHTFDVSFQQSSTAKSATWTYSTDLKKLYCQIAKTCPIQIKVLTPPPQGAVIRAMPVYKKAEHVTEVVKRCPNHELSREFNDGQIAPPSHLIRVEGNSHAQYLEDTITGRQSVFVPYEPPQVGTEFTTILYNFMCNSSCVGGMNRRPILIIVTLETRDGQVLGRRCFEARICACPGRDRKADEDSIRKQHVTDNSKSSEAYRQISQSIQMSTIKKRRSTDEEVFCLPIKGREIYEMLVKIKESLELMQFLPQHTIESYRQQQQNLLQKQTSMQSPTPYGSSSPTHGKVNKLPSVSQLINPQQRNTLTPSSMTGGLTDMTPIMGSHIPMSADMSSLSPTHALQQQLPLVPSSHCTPPPPYPMDSSISSFLLRLGCAGCLDYFTAQGLTNMYQIENYNLEDLSRLKIPAEFQHIIWKGIMEHRQAMDFSPPPHIVRTTSGASTVSVGSSEARGERVIDAVRFTLRQTISFPPRDEWSDFSFDLDSRRNKQQRIKEEGE

>XP_016897404.1 tumor protein 63 isoform X1 [Cynoglossus semilaevis]

MTQHWSSMPHYHRGLWVEISEVAEEAHALWLRLRDTQSCLSWREGSFLNAMAANHTAQATDFFSQDVFHQLFDMLDQSAIHSVQPIELNFTESPVDGSAGNTIQISMDCFTMHGQDDTQSSQYTNLGLLNSMDQNIQNGGSTSTSPYNNDHAQNNVTAPSPYAQPSSTFDALSPSPAIPSNTDYAGPHTFDVSFQQSSTAKSATWTYSTDLKKLYCQIAKTCPIQIKVLTTPPQGAVVRAMPVYKKAEHVTEVVKRCPNHELSREFNDGQIAPPSHLIRVEGNSHAQYVEDSITGRQSVLVPYEPPQVGTEFTTILYNFMCNSSCVGGMNRRPILIIVTLETRDGQVLGRRCFEARICACPGRDRKADEDSIRKQHVTDATKSSDGTKRPFRQVSHGIQMSTIKKRRSTDEEVFCLPIKGREIYEMLVKIKESLELMQFLPQHTIESYRQQQQNLLQKQTSIPSQPCFGSSSPTHGKVNKLPSVSQLINPQQRNTLTPSSMSGGLTDMTPMMGTPIPMNADMSSLSPTHALQPQLPLVPSSHCTPPPPYPMDSSISSFLLRLGCAGCLDYFTAQGLTNIYQIENYNMEDLSRLKIPAEFQHLIWKGIIEHRQAMDFSPPPHIVRTTSGASTVSVGSSEARGERVIDAVRFTLRQTISFPPRDEWSDFSFDLDSRRNKQQRIKEEGE

>XP_035997439.1 tumor protein 63 isoform X2 [Fundulus heteroclitus]

MNSPYTALQYYPEFPFRRLRDPSTLLSWSESSFLTAMTQNQSAQTTDFFSQDVFNQLFDMLDQSAIHSVQPIELNFTDSPTAGSAGNTIQISMDCITMHEQDDLVSPPYTNLGLLNSMEQNNGSTSTSPYNNDHAQNSVTAPSPYAQPSSTFDALSPSPAIPSNTDYAGPHTFDVSFQQSSTAKSATWTYSTDLKKLYCQIAKTCPIQIKVLTPPPQGAVIRAMPVYKKAEHVTEVVKRCPNHELSREFNDGQIAPPSHLIRVEGNNHAQYLEDTITGRQSVLVPYEPPQVGTEFTTILYNFMCNSSCVGGMNRRPILIIVTLETRDGQVLGRRCFEARICACPGRDRKADEDSIRKQHVTDNSKSSEAYRQISQSIQMSTIKKRRSTDEEVFCLPIKGREIYEMLVKIKESLELMQFLPQHTIESYRQQQQNLLQKQTSMQSPTSFGSSSPTHGKVNKLPSVSQLINPQQRNTLTPSSMSGGLTDLTPIMGSHLPMSADMSSLSPTHPLQQQLPLVPSSHCTPPPPYPMDSSISSFLLRLGCAGCLDYFTAQGLSNMYQIENYNMEDLSRLKIPAEFQHIIWKGIMEHRQVIDFPPPPHIVRTSSGASTVSVGSSEARSERVIDAVRFTLRQTISFPPRDEWSDFSFDLDSRRNKQQRIKEEGE

>XP_038159927.1 tumor protein 63 isoform X4 [Cyprinodon tularosa]

MNSPYTALQYYPEFPFHRLREPSSLLSWSESSFLTTMTQNQSAPTTDFFSQDVFNQLFDMLDQSAIHSVQPIELNFTESPTNGSAGNTIQISMDCITMHEPEDVLSSQYTNLGLLNSMEQNNGSTSTSPYNNEHAQNSVTAPSPYAQPSSTFDALSPSPAIPSNTDYAGPHTFDVSFQQSSTAKSATWTYSTDLKKLYCQIAKTCPIQIKVLTPPPQGAVIRAMPVYKKAEHVTEVVKRCPNHELSREFNDGQIAPPSHLIRVEGNSHAQYVEDNITGRQSVLVPYEPPQVGTEFTTILYNFMCNSSCVGGMNRRPILIIVTLETRDGQVLGRRCFEARICACPGRDRKADEDSIRKQHVTDNSKSSEAYRQISQSIQMSTIKKRRSTDEEVFCLPIKGREIYEMLVKIKESLELMQFLPQHTIESYRQQQQNLLQKQPSMQSQPSFGSTSPTHGKVNKLPSVSQLINPQQRNTLTPSSMSGGLTDMTPIMGSHLPMSADMSSLSPTHALQQQLPLVPSSHCTPPPPYPMDSSISSFLLRLGCAGCLDYFTAQGLSNMYQIENYNLEDLSRLKIPAEFQHIIWKGIMEHRQAMDFSPPPHIVRTSSGASTVSLGSSEARGERVIDAVRFTLRQTISFPPRDEWSDFSFDLDSRRNKQQRIKEEGE

**>AEW46989.1 tumor protein p63 [Callorhinchus milii]**

MNSGHVECSALLTDEPTCSICGGECQRCEPLAPRWPRIPSLLVFRLADPPGRGSWLESCCHSTMSQGSQSSDLLNQDWFNQLFDLAAQPVLNVQPISLSFSDELREGFPGTRIEISMDGVRMQDSEPPDPLWPQYTTLGLLNSIDQQMPNGSSSTSPYNEHTSNNVTAPSPYAQPSSTFDTLSPSPAIPSNTDYPGPHGFDVSFQQSSTAKSATWTYSPELKKLYCQIAKTCPIQIKVMTQPPAGAVVRAMPVYKKAEHVTEVVKRCPNHELSREFNDGQVAPPSHLIRVEGNSHAQYVEDPITGRQSVMVPYEPPQVGTEFTTILYNFMCNSSCVGGMNRRPILIIVTLENRDGQVLGRRCFEARICACPGRDRKADEDSIRKQQVTEGTKNGDATKRLRHVNQGIQVASITSKKRRPGEEELFYLPVRGRETYEVLLKIKESLELMQLLPQHTIESYRQQQQHLLQKQSSLQSQQSFGTSPPPMNKMPSMGNKLPSVSQLISPQRNPITHSGMPGNLVPQMMGNPMQMNGDLNGLSPTQGLPPMPTTSHCTPPPPYPSDNSISSFLARVGCAVCLEYFTTQGLINIYQIEHFTMDDLMSLKIPEQFRHAIWKGIVEHRQSLEYAGTPQLLRNTSSASSGSLASQSETRGERVIDAVRFTLRQTISFPPRDDWNDFGFDVDGRRNKQRIKEEGE

>XP_032887579.1 tumor protein 63 isoform X1 [Amblyraja radiata]

MDQLATTSSHSISRLVDPAAHFSLLENCCHSTMSQNSQSSDLCNQDWFNQIFDMAQPVLNVQPIDLSFSDEPHEGVPGNRIEISMDMVRMQDVEPMNPMWYSPPAGQHLSTDGWKPQFTNLGLLNSMEQQIPNGSSSTSPYNNDHTPSITAPSPYAQPSSTFEALSPSPAIPSNTDYPGPHGFDVSFQQSSTAKSATWTYSPELKKLYCQIAKTCPIQIKVMTQPPQGAVIRAMPVYKKAEHVTEVVKRCPNHELSREFNDGQVAPPSHLIRVEGSSHAQYVEDPITGRQSVMVPYEPPQVGTEFTTILYNFMCNSSCVGGMNRRPILIIVTLENRDGQVLGRRCFEARICACPGRDRKADEDSIRKQQVTEGVKGVDSTKRLRHVNQGIQVASISKKRRPGEEELFYLPIKGRDTYEMLLKIKESLELMQQLPQHTIESYRQQQQLLLQKQPSMQSQQSFGPSPPPMNKMHSMGNKLPSVSQLISPQRNTLAQAAMPGNMVPQMMSIPMQMNGEMNGLSPTQGLTSPMASTSHCTPPPPYPSDNSISSFLARAGCASCLEYFTTQGLMNIYQIEHFTMDDLISLKIPDQYRHAIWKGILDHRQSLDFTNAPQLLRNSSSASTASLGSQNETRGERVIDAVRFTLRQTISFPPRDDWNDFGFDVDGRRSKQRIKEEGE

>XP_038671710.1 tumor protein 63 isoform X1 [Scyliorhinus canicula]

MACIKGSSRSAFRDKLVDPTAHFSWVESCCHSNMSQNSQSSDLLNQDWFKQLFELAHPVLNVQPIDLSFSDEPHEGVPGNRIEISMDVVRMQDMEPLDPMWPQYTNLGLLNSMEQQIQNGASSTSPYNNDHTPNVTAPSPYAQPSSTFETLSPSPAIPSNTDYPGPHGFDVSFQQSSTAKSATWTYSPELKKLYCQIAKTCPIQIKVMTQPPQGAVIRAMPVYKKAEHVTEVVKRCPNHELSREFNDGQVAPPSHLIRVEGSSHAQYVEDPITGRQSVMVPYEPPQVGTEFTTILYNFMCNSSCVGGMNRRPILIIVTLENRDGQVLGRRCFEARICACPGRDRKADEDSIRKQQVTEGTKGVDTSKRLRHVNQGIQVASITSKKRRPGDEELFYLPIRGRETYEVLLKIKESLELMQLLPQHTIESYRQQQQHLLQKQPSMQSQQSFGPSPPPMNKMATMGNKLPSVSQLISPQRNALTQAPMPANMVPQMMNMPMQMNGDMNGLSPTHGLPSPMASTSHCTPPPPYPSDNSISSFLTRAGCASCLEYFTTQGLMNIYQIEHFTMDDLISLKIPEQYRHAIWKGILDHRQTLDYTNPPQLLRNTSSASTASMGSQNETRGERVIDAVRFTLRQTISFPPRDDWNDFGFDMDGRRSKQRIKEEGE

>XP_041038631.1 tumor protein 63 isoform X1 [Carcharodon carcharias]

MLYLENAAQSQYSELGSPGHREPDLSGGITSTHKGWQKFGTPFCKWQFMKPQYTNLGLLNSMEQQIQNGSSSTSPYNNDHTPNVTAPSPYAQPSSTFETLSPSPAIPSNTDYPGPHGFDVSFQQSSTAKSATWTYSPELKKLYCQIAKTCPIQIKVMTQPPQGAVIRAMPVYKKAEHVTEVVKRCPNHELSREFNDGQVAPPSHLIRVEGSSHAQYVEDPITGRQSVMVPYEPPQVGTEFTTILYNFMCNSSCVGGMNRRPILIIVTLENRDGQVLGRRCFEARICACPGRDRKADEDSIRKQQVTEGTKGVDTSKRLRHVNQGIQVASITSKKRRPGDEELFYLPIRGRETYEVLLKIKESLELMQLLPQHTIESYRQQQQHLLQKQPSMQSQQSFGPSPPPMNKMATMGNKLPSVSQLISPQRNALAQAPMPGSMVPQMMNVPMQMNGDMNGLSPTQGLPSPMASTSHCTPPPPYPSDNSISSFLTRAGCASCLEYFTTQGLMNIYQIEHFTMDDLISLKIPEQYRHAIWKGILDHRQTLDFTNPPQLLRNTSSASTASMGSQNETRGERVIDAVRFTLRQTISFPPRDDWNDFGFDVDGRRSKQRIKEEGE

>XP_032154156.1 tumor protein 63 isoform X1 [Sapajus apella]

MEMNFETSRCATLQYCPDPYIQRFVETPAHFSWKESYYRSTMSQSTQTNEFFSPEVFQHIWDFLEQPICSVQPIDLNFVDEPSEDGATNKIEISMDCIRMQDSDLSDPMWPQYTNLGLLNSMDQQIQNGSSSTSPYNTDHAQNSVTAPSPYAQPSSTFDALSPSPAIPSNTDYPGPHSFDVSFQQSSTAKSATWTYSTELKKLYCQIAKTCPIQIKVMTPPPQGAVIRAMPVYKKAEHVTEVVKRCPNHELSREFNEGQIAPPSHLIRVEGNSHAQYVEDPITGRQSVLVPYEPPQVGTEFTTVLYNFMCNSSCVGGMNRRPILIIVTLETRDGQVLGRRCFEARICACPGRDRKADEDSIRKQQVSDSTKNGDGTKRPFRQNTHGIQMTSIKKRRSPDDELLYLPVRGRETYEMLLKIKESLELMQYLPQHTIETYRQQQQQQHQHLLQKQTSIQSQSSYGNSSPPLNKMNSMNKLPSVSQLINPQQRNALTPTTIPDGMGANIPMMGTHMPMTGDMNGLSPTQALPPPLSMPSTSHCTPPPPYPTDCSIVSFLARLGCSSCLDYFTTQGLTTIYQIEHYSMDDLASLKIPEQFRHAIWKGILDHRQLHEFSSPSHLLRTPSSASTVSVGSSETRGERVIDAVRFTLRQTISFPPRDEWNDFNFDMDARRNKQQRIKEEGE

>NWU96649.1 P63 protein [Upupa epops]

FFPCFCFLRFVDTPNHFSWKESYYRSAMSQSSQSREFLSPEVLQQIWDFLEQPICSVQPIDLNFIDDPSENGPTNKIEISMDCVRVQDTELNDPMWPQYTNLGLLNSMDQQIQNGSSSTSPYNTEHTQNSVTAPSPYAQPSSTFDALSPSPAIPSNTDYPGPHSFDVSFQQSSTAKSATWTYSTELKKLYCQIAKTCPIQIKVMTPPPQGAVIRAMPVYKKAEHVTEVVKRCPNHELSREFNEGQIAPPSHLIRVEGNSHAQYVEDPITGRQSVLVPYEPPQVGTEFTTVLYNFMCNSSCVGGMNRRPILIIVTLETRDGQVLGRRCFEARICACPGRDRKADEDSIRKQQVSDSTKNGDGTKRPFRQGTHGIQMTSIKKRRSPDDELLYLPVRGRETYEMLLKIKESLELMQYLPQHTIETYRQQQQQQHQHLLQKQTSMQSQSSYGSNSPPLSKMNSMNKLPSVSQLINPQQRNALTPTTIPDGMGTNIPMMGTHMAMTSDMNGLSPTQALPPPLSMPSTSHCTPPPPYPTDCSIVSFLARLGCSSCVDYFTTQGLTTIYQIEHYSMDDLVSLKIPEQFRHAIWKGILDHRQLHDFSSPPHLLRTPSGASTVSVGSSETRGERVIDAVRFTLRQTISFPPRDEWNDFNFDMDARRNKQQRIKEEGE

>XP_036731176.1 tumor protein 63 isoform X1 [Manis pentadactyla]

MNFETPRCATLQYCPDPYIQRFGETPTHFSWKESYYRSTMSQSTQTSEFLSPEVFQHIWDFLEQPICSVQPIDLNFVDEPSENGARNKIEISMDCIRMQDSELGDPMWPQYTNLGLLNSMDQQIQNGSSSTSPYNTDHAQNSVTAPSPYAQPSSTFDALSPSPAIPSNTDYPGPHSFDVSFQQSSTAKSATWTYSTELKKLYCQIAKTCPIQIKVMTPPPQGAVIRAMPVYKKAEHVTEVVKRCPNHELSREFNEGQIAPPSHLIRVEGNSHAQYVEDPITGRQSVLVPYEPPQVGTEFTTVLYNFMCNSSCVGGMNRRPILIIVTLETRDGQVLGRRCFEARICACPGRDRKADEDSIRKQQVTDSTKNGDGTKRPFRQNAHGIQMTSIKKRRSPDDELLYLPVRGRETYEMLLKIKESLELMQYLPQHTIETYRQQQQQQQQHLLQKQTSMQSQSSYGNSSPPLNKMNSMNKLPSVSQLINPQQRNALTPTTIPDGMGANIPMMGTHMPMAGDMNGLSPTQALPPPLSMPSTSHCTPPPPYPTDCSLVSFLARLGCSSCLDYFTTQGLTTIYQIEHYSMDDLASLKIPEQFRHAIWKGILDHRQLHDFSSPPHLLRTPSGASTVSVGSSETRGERVIDAVRFTLRQTISFPPRDEWNDFNFDMDARRNKQQRIKEEGE

>NXF30024.1 P63 protein [Nyctibius bracteatus]

RFVETPSHFSWKESYYRSAMSQSSQSREFLNPEVLQHIWDFLEQPICSVQPIDLNFIDDPSDNGPTNKIEISMDCVRVQDTELNDPMWPQYTNLGLLNSMDQQIQNGSSSTSPYNTEHAQNSVTAPSPYAQPSSTFDALSPSPAIPSNTDYPGPHSFDVSFQQSSTAKSATWTYSTELKKLYCQIAKTCPIQIKVMTQPPQGAVIRAMPVYKKAEHVTEVVKRCPNHELSREFNEGQIAPPSHLIRVEGNSHAQYVEDPITGRQSVLVPYEPPQVGTEFTTVLYNFMCNSSCVGGMNRRPILIIVTLETRDGQVLGRRCFEARICACPGRDRKADEDSIRKQQVSDSTKNGDGTKRPFRQGTHGIQMTSIKKRRSPDDELLYLPVRGRETYEMLLKIKESLELMQYLPQHTIETYRQQQQQQHQHLLQKQTSMQSQSSYGSNSPPLSKMNSMNKLPSVSQLINPQQRNALTPTTIPDGMGTNIPMMGTHMAMTGDMNGLSPTQALPPPLSMPSTSHCTPPPPYPTDCSIVSFLARLGCSSCVDYFTTQGLTTIYQIEHYSMDDLVSLKIPEQFRHAIWKGILDHRQLHDFSSPPHLLRTPSGASTVSVGSSETRGERVIDAVRFTLRQTISFPPRDEWNDFNFDMDARRNKQQRIKEEGE

>XP_010622416.1 tumor protein 63 isoform X1 [Fukomys damarensis]

MPFIQTSNHELDQQFRDASKLPTTRVATIRQGGGGGTTLEKMPSCFVETPAHFSWKESYYRSTMSQSTQTSEFLSPEVFQHIWDFLEQPICSVQPIDLNFVDETSENGATNKIEISMDCIRMQDSDLSDPMWPQYTNLGLLNSMDQQIQNGSSSTSPYNTEHAQNSVTAPSPYAQPSSTFDALSPSPAIPSNTDYPGPHSFDVSFQQSSTAKSATWTYSTELKKLYCQIAKTCPIQIKVMTPPPQGAVIRAMPVYKKAEHVTEVVKRCPNHELSREFNEGQIAPPSHLIRVEGNSHAQYVEDPITGRQSVLVPYEPPQVGTEFTTVLYNFMCNSSCVGGMNRRPILIIVTLETRDGQVLGRRCFEARICACPGRDRKADEDSIRKQQVSDSTKNGDGTKRPFRQNTHGIQMTSIKKRRSPDDELLYLPVRGRETYEMLLKIKESLELMQYLPQHTIETYRQQQQQQHQHLLQKQTSMQSQASYGNSSPPLNKMNNMNKLPSVSQLINPQQRNALTPTTIPDGMGASIPMMGTHMPMAGDMNGLSPTQALPPPLSMPSTSHCTPPPPYPTDCSIVSFLARLGCSSCLDYFTTQGLTTIYQIEHYSMDDLASLKIPEQFRHAIWKGILDHRQLHDFSSPPHLLRTPSGASTVSVGSSETRGERVIDAVRFTLRQTISFPPRDEWNDFNFDMDARRTKQQRIKEEGE

>XP_040126085.1 tumor protein 63 isoform X1 [Ictidomys tridecemlineatus]

MSSTTLSLTSFVETPAHFSWKESYYRSTMSQSTQTSEFLSPEVFQHIWDFLEQPICSVQPIDLNFVDEPSENGATNKIEISMDCIRMQDSDLSDPMWPQYTNLGLLNSMDQQIQNGSSSTSPYNTDHAQNSVTAPSPYAQPSSTFDALSPSPAIPSNTDYPGPHSFDVSFQQSSTAKSATWTYSTELKKLYCQIAKTCPIQIKVMTPPPQGAVIRAMPVYKKAEHVTEVVKRCPNHELSREFNEGQIAPPSHLIRVEGNSHAQYVEDPITGRQSVLVPYEPPQVGTEFTTVLYNFMCNSSCVGGMNRRPILIIVTLETRDGQVLGRRCFEARICACPGRDRKADEDSIRKQQVSDSTKNGDGTKRPFRQNTHGIQMTSIKKRRSPDDELLYLPVRGRETYEMLLKIKESLELMQYLPQHTIETYRQQQQQQHQHLLQKQTSMQSQSSYGNSSPPLNKMNSMNKLPSVSQLINPQQRNALTPTTIPDGMGANIPMMSTHMPMAGDMNGLSPTQALPPPLSMPSTSHCTPPPPYPTDCSIVSFLARLGCSSCLDYFTTQGLTTIYQIEHYSMDDLASLKIPEQFRHAIWKGILDHRQLHDFSSPPHLLRTPSGASTVSVGSSETRGERVIDAVRFTLRQTISFPPRDEWNDFNFDMDARRNKQQRIKEEGE

>XP_037381172.1 tumor protein 63 [Talpa occidentalis]

MPSWFVETPAHFSWKESYYRSTMSQSTQTSEFLSPEVFQHIWDFLEQPICSVQPIDLNFVDEPSENGATNKIEISMDCIRMQDSDLSDPMWPQYTNLGLLNSMDQQIQNGSSSTSPYNTDHAQNSVTAPSPYAQPSSTFDALSPSPAIPSNTDYPGPHSFDVSFQQSSTAKSATWTYSTELKKLYCQIAKTCPIQIKVMTPPPQGAVIRAMPVYKKAEHVTEVVKRCPNHELSREFNEGQIAPPSHLIRVEGNSHAQYVEDPITGRQSVLVPYEPPQVGTEFTTVLYNFMCNSSCVGGMNRRPILIIVTLETRDGQVLGRRCFEARICACPGRDRKADEDSIRKQQVSDSTKNGDGTKRPFRQNTHGIQMTSIKKRRSPDDELLYLPVRGRETYEMLLKIKESLELMQYLPQHTIETYRQQQQQQHQHLLQKQTSMQSQSSYGNSSPPLNKMNSMNKLPSVSQLINPQQRNALTPTTIPDGMGANIPMMGTHMPMAGDMNGLSPTQALPPPLSMPSTSHCTPPPPYPTDCSLVSFLARLGCSSCLDYFTTQGLTTIYQIEHYSMDDLASLKIPEQFRHAIWKGILDHRQLHDFSSPPHLLRTPSGASTVSVGSSETRGERVIDAVRFTLRQTISFPPRDEWNDFNFDMDARRNKQQRIKEEGENPTLTSGERTCLRHLMKLTCVTAPTHGSSWKMITAEPEMTQRKAQIVSKMTFPNHL

>XP_036880152.1 tumor protein 63 isoform X1 [Manis javanica]

MNFETPRCATLQYCPDPYIQRFGETPTHFSWKESYYRSTMSQSTQTSEFLSPEVFQHIWDFLEQPICSVQPIDLNFVDEPSENGARNKIEISMDCIRMQDSDLGDPMWPQYTNLGLLNSMDQQIQNGSSSTSPYNTDHAQNSVTAPSPYAQPSSTFDALSPSPAIPSNTDYPGPHSFDVSFQQSSTAKSATWTYSTELKKLYCQIAKTCPIQIKVMTPPPQGAVIRAMPVYKKAEHVTEVVKRCPNHELSREFNEGQIAPPSHLIRVEGNSHAQYVEDPITGRQSVLVPYEPPQVGTEFTTVLYNFMCNSSCVGGMNRRPILIIVTLETRDGQVLGRRCFEARICACPGRDRKADEDSIRKQQVTDSTKNGDGTKRPFRQNAHGIQMTSIKKRRSPDDELLYLPVRGRETYEMLLKIKESLELMQYLPQHTIETYRQQQQQQQQHLLQKQTSMQSQSSYGNSSPPLNKMNSMNKLPSVSQLINPQQRNALTPTTIPDGMGANIPMMGTHMPMAGDMNGLSPTQALPPPLSMPSTSHCTPPPPYPTDCSLVSFLARLGCSSCLDYFTTQGLTTIYQIEHYSMDDLASLKIPEQFRHAIWKGILDHRQLHDFSSPPHLLRTPSGASTVSVGSSETRGERVIDAVRFTLRQTISFPPRDEWNDFNFDMDARRNKQQRIKEEGE
