# Supplementary material for: Phylogenetic analysis of the MCL1 BH3 binding groove and rBH3 sequence motifs in the p53 and INK4 protein families
Source: PLoS One. 2023 Jan 25;18(1):e0277726. doi: 10.1371/journal.pone.0277726 (PMC9876281; doi:10.1371/journal.pone.0277726)
Supplement: S3 File — A total of 217 p73 sequences were used to generate the p53 family phylogenetic tree. (DOCX) [file pone.0277726.s007.docx]

**>EDL14956.1 transformation related protein 73, isoform CRA_a [Mus musculus]**

MSSPRQLDAAATALPLQATTGPPAPLPKSMDPPRAQAQPRMSGSVGEMAQTSSSSSSTFEHLWSSLEPDSTYFDLPQPSQGTSEASGSEESNMDVFHLQGMAQFNLLSSAMDQMGSRAAPASPYTPEHAASAPTHSPYAQPSSTFDTMSPAPVIPSNTDYPGPHHFEVTFQQSSTAKSATWTYSPLLKKLYCQIAKTCPIQIKVSTPPPPGTAIRAMPVYKKAEHVTDIVKRCPNHELGRDFNEGQSAPASHLIRVEGNNLAQYVDDPVTGRQSVVVPYEPPQVGTEFTTILYNFMCNSSCVGGMNRRPILVIITLETRDGQVLGRRSFEGRICACPGRDRKADEDHYREQQALNESTTKNGAASKRAFKQSPPAIPALGTNVKKRRHGDEDMFYMHVRGRENFEILMKVKESLELMELVPQPLVDSYRQQQQQQLLQRPSHLQPPSYGPVLSPMNKVHGGVNKLPSVNQLVGQPPPHSSAAGPNLGPMGSGMLNSHGHSMPANGEMNGGHSSQTMVSGSHCTPPPPYHADPSLVSFLTGLGCPNCIECFTSQGLQSIYHLQNLTIEDLGALKVPDQYRMTIWRGLQDLKQSHDCGQQLLRSSSNAATISIGGSGELQRQRVMEAVHFRVRHTITIPNRGGAGAVTGPDEWADFGFDLPDCKSRKQPIKEEFTETESH

>XP_021015217.1 tumor protein p73 isoform X1 [Mus caroli]

MSGSVGEMAQSSSSSSSTFEHLWSSLEPDSTYFDLPQPSQGTSEASGSEESNMDVFHLQGMAQFNLLSSAMDQMGSRAAPASPYTPEHAASAPTHSPYAQPSSTFDTMSPAPVIPSNTDYPGPHHFEVTFQQSSTAKSATWTYSPLLKKLYCQIAKTCPIQIKVSTPPPPGTAIRAMPVYKKAEHVTDIVKRCPNHELGRDFNEGQSAPASHLIRVEGNNLSQYVDDPVTGRQSVVVPYEPPQVGTEFTTILYNFMCNSSCVGGMNRRPILVIITLETRDGQVLGRRSFEGRICACPGRDRKADEDHYREQQALNESTTKNGAASKRAFKQSPPAIPALGTNVKKRRHGDEDMFYMHVRGRENFEILMKVKESLELMELVPQPLVDSYRQQQQQQLLQRPSHLQPPSYGPVLSPMNKVHGGVNKLPSVNQLVGQPPPHSSAAGPNLGPMGSGMLNSHSHSMPANGEMNGGHSSQTMVSGSHCTPPPPYHADPSLVSFLTGLGCPNCIECFTSQGLQSIYHLQNLTIEDLGALKVPDQYRMTIWRGLQDLKQSHDCGQQLLRSSSNAATISIGGSGELQRQRVMEAVHFRVRHTITIPNRGGAGAVTGPDEWADFGFDLPDCKSRKQPIKEEFTETESH

>XP_032743290.1 tumor protein p73 [Rattus rattus]

MSSPRQLDAYATALPLQATGPPAPLPKSTDPPRAQAQPRMSNGVGEMAQSSSSSSSSSTFEHLWSSLEPDSTYFDLPQPSQGNSEATGSEESNMDVFHLQGMTSSVMAQFNLLSSAMDQMGSRAAPASPYTPEHAASAPTHSPYAQPSSTFDTMSPAPVIPSNTDYPGPHHFEVTFQQSSTAKSATWTYSPLLKKLYCQIAKTCPIQIKVSTPPPPGTAIRAMPVYKKAEHVTDIVKRCPNHELGRDFNEGQSAPASHLIRVEGNNLSQYVDDPVTGRQSVVVPYEPPQVGTEFTTILYNFMCNSSCVGGMNRRPILVIITLETRDGQVLGRRSFEGRICACPGRDRKADEDHYREQQALNESTTKNGTASKRAFKQSPPAIPALGTNVKKRRHGDEDMFYMHVRGRENFEILMKVKESLELMELVPQPLVDSYRQQQQQQLLQRPSHLQPPSYGPVLSPMNKVHGGVNKLPSVNQLVGQPPPHSSAAGPNLGPMGSGILNSHSHSMPANGEMNGGHSSQTMVSGSHCTPPPPYHADPSLVSFLTGLGCPNCIECFTSQGLQSIYHLQNLTIEDLGALKIPDQYRMTIWRGLQDLKQSHDCGQQLLRSSSNAATISIGGSGELQRQRVMEAVHFRVRHTITIPNRGGAGGVTGPDEWADFGFDLPDCKSRKQPIKEEFTETESH

>NP_001102166.1 tumor protein p73 [Rattus norvegicus]

MSSPRQLDAYATALPLQATGPPAPLPKSTDPSRAQVQPRMSNGVGEMAQSSSSSSSSSTFEHLWSSLEPDSTYFDLPQPSQGNSEATGSEESNMDVFHLQGMTSSVMAQFNLLSSAMDQMGSRAAPASPYTPEHAASAPTHSPYAQPSSTFDTMSPAPVIPSNTDYPGPHHFEVTFQQSSTAKSATWTYSPLLKKLYCQIAKTCPIQIKVSTPPPPGTAIRAMPVYKKAEHVTDIVKRCPNHELGRDFNEGQSAPASHLIRVEGNNLSQYVDDPVTGRQSVVVPYEPPQVGTEFTTILYNFMCNSSCVGGMNRRPILVIITLETRDGQVLGRRSFEGRICACPGRDRKADEDHYREQQALNESTTKNGAASKRAFKQSPPAIPALGTNVKKRRHGDEDMFYMHVRGRENFEILMKVKESLELMELVPQPLVDSYRQQQQQQLLQRPSHLQPPSYGPVLSPMNKVHGGVNKLPSVNQLVGQPPPHSSAAGPNLGSMGSGILNSHSHSMPANGEMNGGHSSQTMVSGSHCTPPPPYHADPSLVSFLTGLGCPNCIECFTSQGLQSIYHLQNLTIEDLGALKIPDQYRMTIWRGLQDLKQSHDCGQQLLRSSSNAATISIGGSGELQRQRVMEAVHFRVRHTITIPNRGGAGGVTGPDEWADFGFDLPDCKSRKQPIKEEFTETESH

>XP_031235531.1 tumor protein p73 isoform X1 [Mastomys coucha]

MSGSVGEMAQSSSSSSSSTFEHLWSSLEPDSTYFDLPQTSQGNSEASGSEESNMDVFHLQGMAQFNLLSSAMDQMGSRAAPASPYTPEHTASAPTHSPYAQPSSTFDTMSPAPVIPSNTDYPGPHHFEVTFQQSSTAKSATWTYSPLLKKLYCQIAKTCPIQIKVSTPPPPGTAIRAMPVYKKAEHVTDIVKRCPNHELGRDFNEGQSAPASHLIRVEGNNLSQYVDDPVTGRQSVVVPYEPPQVGTEFTTILYNFMCNSSCVGGMNRRPILVIITLETRDGQVLGRRSFEGRICACPGRDRKADEDHYREQQALNESTTKNGAASKRAFKQSPPAIPALGTNVKKRRHGDEDMFYMHVRGRENFEILMKVKESLELMELVPQPLVDSYRQQQQQQLLQRPSHLQPPSYGPVLSPMNKVHGGVNKLPSVNQLVGQPPPHSSAAGPNLGPMGSGMLNSHSMPANGEMNGGHSSQTMVSGSHCTPPPPYHADPSLVSFLTGLGCPNCIECFTSQGLQSIYHLQNLTIEDLGALKIPDQYRMTIWRGLQDLKQSHDCSQQLLRSSSNAATISIGGSGELQRQRVMEAVHFRVRHTITIPNRGGAGGVTGPDEWADFGFDLPDCKSRKQPIKEEFTETESH

>XP_034358564.1 tumor protein p73 isoform X1 [Arvicanthis niloticus]

MAQSSSSSNTTFEHLWSTLEPDSTYFDLPQSSQGNSEASGSEESSMDVFQLQGMTSSVMAQFNLLSSAMDQMGSRAAPASPYTPEHAASAPTHSPYAQPSSTFDTMSPAPVIPSNTDYPGPHHFEVTFQQSSTAKSATWTYSPLLKKLYCQIAKTCPIQIKVSTPPPPGTAIRAMPVYKKAEHVTDIVKRCPNHELGRDFNEGQSAPASHLIRVEGNNLSQYVDDPVTGRQSVVVPYEPPQVGTEFTTILYNFMCNSSCVGGMNRRPILVIITLETRDGQVLGRRSFEGRICACPGRDRKADEDHYREQQALNESTTKNGAASKRAFKQSPPAIPALGTNVKKRRHGDEDTFYIHVRGRENFEILMKVKESLELMELVPQPLVDSYRQQQQQQLLQRPSHLQPPSYGPVLSPMNKVHGGVNKLPSVNQLVGQPPPHSSAAGPNLGPMGSGMLNSHAHSMPANGEMNGGHSSQTMVSGSHCTPPPPYHADPSLVSFLTGLGCPNCIECFTSQGLQSIYHLQNLTIEDLGALKIPDQYRMTIWRGLQDLKQSHDCGQQLLRSSSNAATISIGGSGELQRQRVMEAVHFRVRHTITIPNRGGAGGVTGPDEWADFGFDLPDCKSRKQPIKEEFTETESH

>XP_028613668.1 tumor protein p73 [Grammomys surdaster]

MDTSRAQAQPRMSGSVGEMAQSSSSSTTTATTFEHLWSTLEPDSTYFDLSQPSQGNSEASGSEESSMDVFQLQGLTSSVMAQFNLLSSAMDQMGSRAAPASPYTPEHAASAPTHSPYAQPSSTFDTMSPAPVIPSNTDYPGPHHFEVTFQQSSTAKSATWTYSPLLKKLYCQIAKTCPIQIKVSTPPPPGTAIRAMPVYKKAEHVTDIVKRCPNHELGRDFNEGQSAPASHLIRVEGNNLSQYVDDPVTGRQSVVVPYEPPQVGTEFTTILYNFMCNSSCVGGMNRRPILVIITLETRDGQVLGRRSFEGRICACPGRDRKADEDHYREQQALNESTTKNGAASKRAFKQSPPAIPALGTNVKKRRHGDEDTFYMHVRGRENFEILMKVKESLELMELVPQPLVDSYRQQQQQQLLQRPSHLQPPSYGPVLSPMNKVHGGVNKLPSVNQLVGQPPPHSSAAGPNLGPMGSGMLNSHSHSMPANGEINGGHSSQTMVSGSHCTPPPPYHADPSLISFLTGLGCPNCIECFTSQGLQSLYHLQNLTIEDLGALKIPDQYRMTIWRGLQDLKQSHDCGQQLLRSSSNAATISIGGSGELQRQRVMEAVHFRVRHTITIPNRGGAGGVTGPDEWADFGFDLPDCKSRKQPIKEEFTETESH

>XP_005368693.1 tumor protein p73 [Microtus ochrogaster]

MSRSVREMTQSASSSPGEGATFEHLWSSLEPDSTYFDLPQPSRGNSEAVGSEETSMDVFHLPGMAQFNLLSSAMDQMGSRAAPASPYTPEHAASAPTHSPYAQPSSTFDTMSPAPVIPSNTDYPGPHHFEVTFQQSSTAKSATWTYSPLLKKLYCQIAKTCPIQIKVSTPPPPGTAIRAMPVYKKAEHVTDIVKRCPNHELGRDFNEGQSAPASHLIRVEGNNLSQYVDDPVTGRQSVVVPYEPPQVGTEFTTILYNFMCNSSCVGGMNRRPILVIITLETRDGQVLGRRSFEGRICACPGRDRKADEDHYREQQALNESATKNGAASKRAFKQSPPAIPALGANVKKRRHGDEDMFYMHVRGRENFEILMKVKESLELMELVPQPLVDSYRQQQQQQLLQRPSHMQSSSYGPVLSPINKVHGGINKLPSVNQLVGQPPPHSSAAGPNLGPMGSGMLNSHSHTMTANGEMNGGHGSQTMVSGSHCTPPPPYHADPSLVSFLTGLGCPNCIECFTSQGLQSIYHLQNLTIEDLGALKIPDQYRMTIWRGLQDLKQSHDCGQQLLRTSSNAATISIGGSGELQRQRVMEAVHFRVRHTITIPNRGGPGGVTGPDEWADFGFDLPDCKSRKQPIKEEFTETESH

>XP_038189341.1 tumor protein p73 [Arvicola amphibius]

MSRSVGEMTQSASSSPGEGATFEHLWSSLEPDSTYFDLPQSSRGNSEAVGSEETSMDVFHLPGMAQFNLLSSAMDQMGSRAAPASPYTPEHAASAPTHSPYAQPSSTFDTMSPAPVIPSNTDYPGPHHFEVTFQQSSTAKSATWTYSPLLKKLYCQIAKTCPIQIKVSTPPPPGTAIRAMPVYKKAEHVTDIVKRCPNHELGRDFNEGQSAPASHLIRVEGNNLSQYVDDPVTGRQSVVVPYEPPQVGTEFTTILYNFMCNSSCVGGMNRRPILVIITLETRDGQVLGRRSFEGRICACPGRDRKADEDHYREQQALNESATKNGAASKRAFKQSPPAIPALGANVKKRRHGDEDMFYMHVRGRENFEILMKVKESLELMELVPQPLVDSYRQQQQQLLQRPSHLQSSSYGPVLSPINKVHSGVNKLPSVNQLVGQPPPHSSAAGPNLGPMGSGMLNSHSHTMTANGEMNGGHSSQTMVSGSHCTPPPPYHADPSLVSFLTGLGCPNCIECFTSQGLQSIYHLQNLTIEDLGALKIPDQYRMTIWRGLQDLKQSHDCGQQ

>XP_041508044.1 tumor protein p73 isoform X1 [Microtus oregoni]

MSSPRQLGRCCHCPATALLQTTGPPAPQSKSTDLPRAQAQPRMSRSVGEMTQSASSSPGEGATFEHLWSSLEPDSTYFDLPQPSRGNSEAVGSEETSMDVFHLPGMVSQIMAQFNLLSSAMDQMGSRAAPASPYTPEHAASAPTHSPYAQPSSTFDTMSPAPVIPSNTDYPGPHHFEVTFQQSSTAKSATWTYSPLLKKLYCQIAKTCPIQIKVSTPPPPGTAIRAMPVYKKAEHVTDIVKRCPNHELGRDFNEGQSAPASHLIRVEGNNLSQYVDDPVTGRQSVVVPYEPPQVGTEFTTILYNFMCNSSCVGGMNRRPILVIITLETRDGQVLGRRSFEGRICACPGRDRKADEDHYREQQALNESATKNGAASKRAFKQSPPAIPALGANVKKRRHGDEDMFYMHVRGRENFEILMKVKESLELMELVPQPLVDSYRQQQQQQLLQRPSHLQSSSYGPVLSPINKVHGGVNKLPSVNQLVGQPPPHSSAAGPNLGPMGSGMLNSHNHTMAANGEMNGGHGSQTMVSGSHCTPPPPYHADPSLVSFLTGLGCPNCIECFTSQGLQSIYHLQNLTIEDLGALKIPDQYRMTIWRGLQDLKQSHDCGQQLLRTSSNAATISIGGSGELQRQRVMEAVHFRVRHTITIPNRGGPGGVTGPDEWADFGFDLPDCKSRKQPIKEEFTETESH

>XP_028716092.2 tumor protein p73 [Peromyscus leucopus]

MELPRCSYCPPSSSRMSRSVGEMAQSPSSSPGEGATFEHLWSSLEPDSTYFDLPPPNRGNNEAVGSEETSMDVFHLPGMTSSVMAQFNLLSSAMDQMGSRAAPASPYTPEHAASAPTHSPYAQPSSTFDTMSPAPVIPSNTDYPGPHHFEVTFQQSSTAKSATWTYSPLLKKLYCQIAKTCPIQIKVSTPPPPGTAIRAMPVYKKAEHVTDIVKRCPNHELGRDFNEGQSAPASHLIRVEGNNLSQYVDDPVTGRQSVVVPYEPPQVGTEFTTILYNFMCNSSCVGGMNRRPILVIITLETRDGQVLGRRSFEGRICACPGRDRKADEDHYREQQALNESATKNGAASKRAFKQSPPAIPALGANVKKRRHGDEDMFYMHVRGRENFEILMKVKESLELMELVPQPLVDSYRQQQQQQLLQRPSHLQPSSYGPVLSPMNKIHGGVNKLPSVNQLVGQPPPHSSAAGSNLGPMGSGMLNSHSHSMPANGEMNGGHSSQTMVSGSHCTPPPPYHADPSLVSFLTGLGCPNCIECFTSQGLQSIYHLQNLTIEDLGALKIPDQYRMTIWRGLQDLKQSHDCGQQLLRSSSNAATISIGGSGELQRQRVMEAVHFRVRHTITIPNRGGPGGVAGPDEWADFGFDLPDCKSRKQPIKEEFTETESH

>XP_036034583.1 tumor protein p73 isoform X1 [Onychomys torridus]

MAQSPSSSPGEGATFEHLWSSLEPDSTYFDLPPPNRGNSEAVGSEETNMDVFHLPGMTSSVMAQFNLLSSAMDQMGSRAAPASPYTPEHAASAPTHSPYAQPSSTFDTMSPAPVIPSNTDYPGPHHFEVTFQQSSTAKSATWTYSPLLKKLYCQIAKTCPIQIKVSTPPPPGTAIRAMPVYKKAEHVTDIVKRCPNHELGRDFNEGQSAPASHLIRVEGNNLSQYVDDPVTGRQSVVVPYEPPQVGTEFTTILYNFMCNSSCVGGMNRRPILVIITLETRDGQVLGRRSFEGRICACPGRDRKADEDHYREQQALNESATKNGAASKRAFKQSPPAIPALGANVKKRRHGDEDMFYMHVRGRENFEILMKVKESLELMELVPQPLVESYRQQQQQQQQQQLLQRPSHLQPPSYGPVLSPMNKIHGGVNKLPSVNQLVGQPPPHSSAAGSNLGPMGSGMLNSHSHTIPANGEMNGGHSSQTMVSGSHCTPPPPYHADPSLVSFLTGLGCPNCIECFTSQGLQSIYHLQNLTIEDLGALKIPDQYRMTIWRGLQDLKQSHDCGQQLLRSSSNAATISIGGSGELQRQRVMEAVHFRVRHTITIPNRGGPGGVTGPDEWADFGFDLPDCKSRKQPIKEEFTETESH

>XP_040590998.1 tumor protein p73 isoform X1 [Mesocricetus auratus]

MSRSVGEMAQSPSSSPGEGATFEHLWNSLEPDSTYFDLPQPSRGNSEAVGGEETSMDVFHLPGVTSSVMAQFNLLSSAMDQMGSRAAPASPYTPEHAASAPTHSPYAQPSSTFDTMSPAPVIPSNTDYPGPHHFEVTFQQSSTAKSATWTYSPLLKKLYCQIAKTCPIQIKVSTPPPPGTAIRAMPVYKKAEHVTDIVKRCPNHELGRDFNEGQSAPASHLIRVEGNNLSQYVDDPVTGRQSVVVPYEPPQVGTEFTTILYNFMCNSSCVGGMNRRPILVIITLETRDGQVLGRRSFEGRICACPGRDRKADEDHYREQQALNESAAKNGAASKRAFKQSPPAIPALGANMKKRRHGDEDMFYMHVRGRENFEILMKVKESLELMELVPQPLVDSYRQQQQQQLLQRPGHLQPPSYGPVLSPMNKVHGGVNKLPSVNQLVGQPPPHSSATGPNLGPMGSGMLNSHSHTMPTNGEMNGGHGSQTMVSGSHCTPPPPYHADPSLVSFLTGLGCPNCIECFTSQGLQSIYHLQNLTIEDLGALKIPDQYRMTIWRGLQDLKQGHDCGQQLLRSSSNAATISIGGSGELQRQRVMEAVHFRVRHTITIPNRGGPGGVTGPDEWADFGFDLPDCKSRKQPIKEEFTETEGH

>XP_029411692.1 tumor protein p73 isoform X1 [Nannospalax galili]

MPSRASRSVGEMARSPSSSPGEGATFEHLWSSLEPDSTYFDLPQPSQGTSETVGSEETGMDVFHLPGMTSSAIAQLNLFSSAMDQMGSRAAPASPYTPEHATSAPTHSPYSQPSSTFDTMSPAPVIPSNTDYPGPHHFEVTFQQSSTAKSATWTYSPLLKKLYCQIAKTCPIQIKVSAPPPPGTAIRAMPVYKKAEHVTDIVKRCPNHELGRDFNEGQSAPASHLIRVEGNNLSQYVDDPVTGRQSVVVPYEPPQVGTEFTTILYNFMCNSSCVGGMNRRPILVIITLETRDGQVLGRRSFEGRICACPGRDRKADEDHYREQQALNESAAKNGAASKRAFKQSPPAIPTLGANVKKRRHGEEDIYYMHVRGRENFEILMKVKESLELMELVPQPLVDSYRQQQQQQLLQRPSHLQPPSYGPVLSPMSKVHGGVNKLPSVNQLVGQPPPHGSGAGPNLGPMGSGMLNSHSHAMPANGEMNGGHSSQSMVSGSHCTPPPPYHADPSLVSFLTGLGCPNCIEYFTSQGLQSIYHLQNLTIEDLGALKIPDQYRMTIWRGLQDLRQGPDCAQQLLRSSGNAATISIGSTGELQRQRVMEAVHFRVRHTITIPNRGGPSGVTGPDEWADFGFDLPDCKSRKQPIKEEFTESESH

>XP_020013952.1 tumor protein p73 isoform X2 [Castor canadensis]

MAQSTSPDEGATFEHLWSSLEPDSTYFDLPQSSRGSTEVVGSTEASPDVFHLPGMNTSAQFNLLSSTMEQMSSRAAPASPYTPEHAASVPTHSPYAQPSSTFDTMSPAPVIPSNTDYPGPHHFEVTFQQSSTAKSATWTYSPLLKKLYCQIAKTCPIQIKVSTPPPPGTAIRAMPVYKKAEHVTDIVKRCPNHELGRDFNEGQSAPASHLIRVEGNNLCQYVDDPVTGRQSVVVPYEPPQVGTEFTTILYNFMCNSSCVGGMNRRPILIIITLETRDGQVLGRRSFEGRICACPGRDRKADEDHYREQQALNESATKNGAASKRAFKQSPPAIPALGANVKKRRHGDEDIFYMHVRGRENFEILMKVKESLELMELVPQPLVDSYRQQQQQLLQRPSHLQPPSYGPVLSPMNKVHGGVNKLPSVNQLVGQPPPHSSAAGPNLGPMGPGMLNSHSHALPANGEMNGGHSSQSMVSGSHCTPPPPYHADPSLVSFLTGLGCPNCIEYFTSQGLQNIYHLQNLTIEDLGALKIPDQYRMTIWRGLQDLKQSHDCGQQLLRSSSNAATISIGGSGELQRQRVMEAVHFRVRHTITIPNRSGPGTVAGPDEWADFGFDLPDCKSHKQSIKEEFTESESH

>XP_004863818.1 tumor protein p73 isoform X2 [Heterocephalus glaber]

MAQSSSTAPDEGSTFEHLWSSLEPDSTYFDLSQARQGNTEAVGSTEAAMDVFHLQDLTTPVMAQFNLLSSTMDQMSSRAAPASPYTPEHAASVPTHSPYAQPSSTFDAMSPAPVIPSNTDYPGPHHFEVTFQQSSTAKSATWTYSPLLKKLYCQIAKTCPIQVKVSTPPPPGTAIRAMPVYKKAEHVTDVVKRCPNHELGRDFNEGQSAPASHLIRVEGNNLSQYVDDPVTGRQSVVVPYEPPQVGTEFTTILYNFMCNSSCVGGMNRRPILIIITLETRDGQVLGRRSFEGRICACPGRDRKADEDHYREQQALNENATKNGATSKRAFKQSPPAIPALGANMKKRRHGDEDMYYMHVRGRENFEILMKVKESLELMELVPQPLVDSYRQQQQQQFLQRPSHLQPPSYGPVLSPMSKAHGSVNKLPSVNQLVGQPPPHASAAGASLGPMGPSMLSSHSHAMPANGEMNGSHSSQTVVSGSHCTPPPPYHADPSLVSFLTGLGCPNCIEYFTSQGLQNIYHLQNLTIEDLGALKIPDQYRMTIWRGLQDLKQGHDCGQQLLRSSSNAATISIGGAGELQRQRVMEAVHFRVRHTITIPNRGGPGGVAGPDEWADFGFDLPDCKSRKQPIKEEFPESEGH

>XP_021055474.1 tumor protein p73 isoform X4 [Mus pahari]

MLYVGDPMRHLATAQFNLLSSAMDQMGSRAAPASPYTPEHAASAPTHSPYAQPSSTFDTMSPAPVIPSNTDYPGPHHFEVTFQQSSTAKSATWTYSPLLKKLYCQIAKTCPIQIKVSTPPPPGTAIRAMPVYKKAEHVTDIVKRCPNHELGRDFNEGQSAPASHLIRVEGNNLSQYVDDPVTGRQSVVVPYEPPQVGTEFTTILYNFMCNSSCVGGMNRRPILVIITLETRDGQVLGRRSFEGRICACPGRDRKADEDHYREQQALNESTTKNGAASKRAFKQSPPAIPALGTNVKKRRHGDEDMFYMHVRGRENFEILMKVKESLELMELVPQPLVDSYRQQQQQQLLQRPSHLQPPSYGPVLSPMNKVHGGVNKLPSVNQLVGQPPPHSSAAGPNLGPMGSGMLNSHSHSMQANGEMNGGHSSQTMVSGSHCTPPPPYHADPSLVSFLTGLGCPNCIECFTSQGLQSIYHLQNLTIEDLGALKIPDQYRMTIWRGLQDLKQSHDCGQQLLRSSSNAATISIGGSGELQRQRVMEAVHFRVRHTITIPNRGGAGGVTGPDEWADFGFDLPDCKSRKQPIKEEFTETENH

>KFO31789.1 Tumor protein p73 [Fukomys damarensis]

MHSLHWQPGNEVPRKGGCQTVVALMGRPAMCGTVEKPPALRVGIEATTGQTRETRRRGHSRPEKRHPAGESSLGEAGGSSLLPNWALPLPGSVAPSWQGHVASCLLTRPELLPASGSLALGLLGPLESRQSVSSLMASGVQDMAQASSTAPDEGSTFEHLWSSLEPDSTYFDLSQARQGNTGAVGGTEAGMDVFHLEDMTTPVMAQFNLLSSTMDQMSSRAAPASPYTPEHAASVPTHSPYAQPSSTFDTMSPAPVIPSNTDYPGPHHFEVTFQQSSTAKSATWTYSPLLKKLYCQIAKTCPIQIKVSTPPPPGTAIRAMPVYKKAEHVTDVVKRCPNHELGRDFNEGQSAPASHLIRVEGNNLSQYVDDPVTGRQSVVVPYEPPQVGTEFTTILYNFMCNSSCVGGMNRRPILIIITLETRDGQVLGRRSFEGRICACPGRDRKADEDHYREQQALNENATKNGATSKRAFKQSPPAIPALSANMKKRRHGDEDMYYMHVRGRENFEILMKVKESLELMELVPQPLVDSYRQQQQQLLQRPSHLQPPSYAPVLSPMSKVHSGVNKLPSVNQLVGQPPPHTSAAAPSLGPMAPGMLSSHSHAMPANGEMNGGHSSQTVVSGSHCTPPPPYHADPSLVSFLTGLGCPNCIEYFTSQGLQSIYHLQNLTMEDLGALKIPDQYRMTIWRGLQDLKQGHDCGQQLLRSSSNAATISIGGAGELQRQRVMEAVHFRVRHTITIPNRGGPGGVAGPDEWADFGFDLPDCKSRKQPIKEEFPESEGH

>XP_004639669.1 tumor protein p73 [Octodon degus]

MAQSSSTAPDEESTFEHLWSSLEPDSTYFDLSQPSQGNTEAAGSTEASMDVFHLQDMTTPVMAQFNLLSSTMDQMSSRAAPASPYTPEHGASVPTHSPYAQPSSTFDTMSPAPVIPSNTDYPGPHHFEVTFQQSSTAKSATWTYSPMLKKLYCQIAKTCPIQIKVSAPPPPGTAIRAMPVYKKAEHVTDVVKRCPNHELGRDFNEGQSAPASHLIRVEGNNLSQYVDDPVTGRQSVLVPYEPPQVGTEFTTILYNFMCNSSCVGGMNRRPILIIITLETRDGQVLGRRSFEGRICACPGRDRKADEDHYREQQALSENATKNGAASKRAFKQSPPTIPALGANSKKRRHGDEDMYYMHVRGRENFEILMKVKESLELMDLVPQPLVDSYRQQQQQQLLQRPSHLQPPSYGPVLSPMSKAHGSVNKLPSVNQLVGPAPPHTSATGPSLGPMAPAMLSGHSHAMPPNGELNGGHSSQTMVPGSHCTPPPPYHADPSLVSFLTGLGCPNCIEYFTSQGLQNIYHLQNLTIEDLGALKIPDQYRMTIWRGLQDLKQGHECGQQLLRSSSNAATISIGGSGELQRQRVMEAVHFRVRHTITIPNRGGPGGVAGTDEWADFGFDLPDCKSRKQPIKEEFPESEGR

>ERE82728.1 tumor protein [Cricetulus griseus]

MSRSAGDMAQSPSSPGEGATFEHLWNSLEPDSTYFDLPQPSRGNSEAVGSEETSMDVFHLPGMTSPVMVPLPTLHRRSLSADWYLVVPMCWSSPPKYQDSPAKVAWRKPVQSLDSHRASPPQSTPAGSHQSAPTPPSHPDPCLVPRHTSSSACADPHEPTMLYVGDPMRHLATAQFNLLSSAMDQMGSRAAPASPYTPEHTASAPTHSPYAQPSSTFDTMSPAPVIPSNTDYPGPHHFEVTFQQSSTAKSATWTYSPLLKKLYCQIAKTCPIQIKVSTPPPPGTAIRAMPVYKKAEHVTDIVKRCPNHELGRDFNEGQSAPASHLIRVEGNNLSQYVDDPVTGRQSVVVPYEPPQVGTEFTTILYNFMCNSSCVGGMNRRPILVIITLETRDGQVLGRRSFEGRICACPGRDRKADEDHYREQQALNESATKNGAASKRAFKQSPPAIPALGANVKKRRHGDEDVFYMHVRGRENFEILMKVKESLELMELVPQPLVDSYRQQQQQLLQRPSHLQPPSYGPVLSPMNKVHGGVNKLPSVNQLVGQPPPHSSSAGPNLGPMGSGMLNSHTMPANGEMNGGHSSQTMVSGSHCTPPPPYHADPSLVSFLTGLGCPNCIECFTSQGLQSIYHLQNLTIEDLGALKIPDQYRMTIWRGLQDLKQSHDCGQQLLRSSSNAATISIGGSGELQRQRVMEAVHFRVRHTITIPNRGGPGGVTGPDEWADFGFDLPDCKSRKQPIKEEFTETESH

>XP_032350903.1 tumor protein p73 isoform X1 [Camelus ferus]

MSQSTQSAAADEGATFQHLWSSLEPDSTYFDLPQSGQGNAEVAGGAEAGMDVFHLQGMTSSVMSQFNLLSSTMDQMSSRAAPASPYTPEHAASVPTHSPYAQPSSTFDTMSPAPVIPSNADYPGPHHFEVTFQQSSTAKSATWTYSPLLKKLYCQIAKTCPVQIKVSTPPPPGTAIRAMPVYKKAEHVTEVVKRCPNHELGRDFNEGQSAPASHLIRVEGNNLSQYVDDPVTGRQSVMVPYEPPQVGTEFTTILYNFMCNSSCVGGMNRRPILIIITLETRDGQVLGRRSFEGRICACPGRDRKADEDHYREQQALNESAAKSGAASKRAFKQSPPAIPALGTSVKKRRHGDEDVYYMHVRGRENYEILMKIKESLELVELVPQQLVDSYRQQQLLQRPSHLQPPSYGPVLSPMNKVHGAVNKLPSVNQLVGQPPPHGSAAGPNLGPMGPGILNNHGHALQADGEMNGGPGSQPVVSGSHCTPPPPYHADPSLVSFLTGLGCPNCIECFTSQGLQSVYHLQSLTMEDLGALKVPEQYRMTIWRGLQDLKQGHDYGAQQLIRSSSNAATISFGSSGELQRQRVMEAVHFRVRHTITIPNRGGPAGASGPDEWADFGFDLPDCKSRKQSIKEEFTEGEAH

>XP_031319742.1 tumor protein p73 isoform X1 [Camelus dromedarius]

MSQSTQSAAAADEGATFQHLWSSLEPDSTYFDLPQSGQGNAEVAGGAEAGMDVFHLQGMTSSVMSQFNLLSSTMDQMSSRAAPASPYTPEHAASVPTHSPYAQPSSTFDTMSPAPVIPSNADYPGPHHFEVTFQQSSTAKSATWTYSPLLKKLYCQIAKTCPVQIKVSTPPPPGTAIRAMPVYKKAEHVTEVVKRCPNHELGRDFNEGQSAPASHLIRVEGNNLSQYVDDPVTGRQSVMVPYEPPQVGTEFTTILYNFMCNSSCVGGMNRRPILIIITLETRDGQVLGRRSFEGRICACPGRDRKADEDHYREQQALNESAAKSGAASKRAFKQSPPAIPALGTSVKKRRHGDEDVYYMHVRGRENYEILMKIKESLELVELVPQQLVDSYRQQQLLQRPSHLQPPSYGPVLSPMNKVHGAVNKLPSVNQLVGQPPPHGSAAGPNLGPMGPGILNNHGHALQADGEMNGGPGSQPVVSGSHCTPPPPYHADPSLVSFLTGLGCPNCIECFTSQGLQSVYHLQSLTMEDLGALKVPEQYRMTIWRGLQDLKQGHDYGAQQLIRSSSNAATISFGSSGELQRQRVMEAVHFRVRHTITIPNRGGPAGASGPDEWADFGFDLPDCKSRKQSIKEEFTEGEAH

>XP_027803564.1 tumor protein p73 isoform X1 [Marmota flaviventris]

MAQSTSTSPDEGATFEHLWSSLEPDSTYFDLPQPSRGSNEAAVGAEPSMDVFQLPGMTTSVMAQFNLLSSTMDQMSSRPAPASPYTPEHAASVPTHSPYAQPSSTFDTMSPAPVIPSNTDYPGPHHFEVTFQQSSTAKSATWTYSPLLKKLYCQIAKTCPIQIKVSTPPPPGTAVRAMPVYKKAEHVTDIVKRCPNHELGRDFNEGQSAPASHLIRVEGNSLSQYVDDPVTGRQSVVVPYEPPQVGTEFTTILYNFMCNSSCVGGMNRRPILIIITLETRDGQVLGRRSFEGRICACPGRDRKADEDHYREQQALNESATKSVAAGKRAFKQSPPAIPTLGSSVKKRRHGDEDVYYMHVRGRENFEILMKVKESLELMELVPQPLVDSYRQQQQQLLQRPSPLQPPSYGPVLSPMSKVHAGVSKLPSVNQLVGQPPPHSSAAGPNLGPMGPGVLNSHSHTVPANGEMNGGHSSQSMVSGPHCSPPPPYHADPSLVSFLTGLGCPNCIEYFTSQGLQNMYHLQNLTIEDLGALKIPDQYRMTIWRGLQDLKQGHDCGQQLLRSSGNTATISIGGSGELQRQRVMEAVHFRVRHTITIPNRSGPGVAGPDEWADFGFDLPDCRSRKQPIKEEFTESESH

>XP_026268659.1 tumor protein p73 [Urocitellus parryii]

MAQSTSTSPDEGATFEHLWSSLEPDSTYFDLPQSSRGSNEAAVGAEPSMDVFQLPGLTTSVMAQFNLLSSTMDQMSSRPAPASPYTPEHAASVPTHSPYAQPSSTFDTMSPAPVIPSNTDYPGPHHFEVTFQQSSTAKSATWTYSPLLKKLYCQIAKTCPIQIKVSTPPPPGTAVRAMPVYKKAEHVTDIVKRCPNHELGRDFNEGQSAPASHLIRVEGNSLSQYVDDPVTGRQSVVVPYEPPQVGTEFTTILYNFMCNSSCVGGMNRRPILIIITLETRDGQVLGRRSFEGRICACPGRDRKADEDHYREQQALNESTTKSVAAGKRAFKQSPPAIPTLGSSVKKRRHGDEDVYYMHVRGRENFEILMKVKESLELMELVPQPLVDSYRQQQQQQLLQRPSPLQSPSYGPVLSPMSKVHAGVSKLPSVNQLVGQPPPHSSAAGPNLGPMGPGVLNSHSHTVPANGEMNGGHSSQSMVSGPHCSPPPPYHADPSLVSFLTGLGCPNCIEYFTSQGLQNMYHLQNLTIEDLGALKIPDQYRMTIWRGLQDLKQGHDCGQQLLRSSGNTATISIGGSGELQRQRVMEAVHFRVRHTITIPNRSGPGVAGPDEWADFGFDLPDCRSRKQSIKEEFTETESH

>KAF7463230.1 tumor protein p73 [Marmota monax]

MSSPRRPDAAATAGLRSQEPKASRPPLPGPTDSPGAPAQPRGQGRSLPHGLGPWTGGAGKGGRCPGTEEGDIIPGGGPWPWPTRVSCPWKTAQGAMAQSTSTSPDEGATFEHLWSSLEPDSTYFDLPQPSRGNNEAAVGAEPSMDVFQLPGMTTSVMAQFNLLSSTMDQMSSRPAPASPYTPEHAASVPTHSPYAQPSSTFDTMSPAPVIPSNTDYPGPHHFEVTFQQSSTAKSATWTYSPLLKKLYCQIAKTCPIQIKVSTPPPPGTAVRAMPVYKKAEHVTDIVKRCPNHELGRDFNEGQSAPASHLIRVEGNSLSQYVDDPVTGRQSVVVPYEPPQVGTEFTTILYNFMCNSSCVGGMNRRPILIIITLETRDGQVLGRRSFEGRICACPGRDRKADEDHYREQQALNESATKSVAAGKRAFKQSPPAIPTLGSSVKKRRHGDEDVYYMHVRGRENFEILMKVKESLELMELVPQPLVDSYRQQQQQLLQRPSPLQPPSYGPVLSPMSKVHAGVSKLPSVNQLVGQPPPHSSAAGPNLGPMGPGVLNSHSHTVPANGEMNGGHSSQSMVSGPHCSPPPPYHADPSLVSFLTGLGCPNCIEYFTSQGLQNMYHLQNLTIEDLGALKIPDQYRMTIWRGLQDLKQGHDCGQQLLRSSGNTATISIGGSGELQRQRVMEAVHFRVRHTITIPNRSGPGVAGPDEWADFGFDLPDCRSRKQPIKEEFTESESH

>XP_006161249.1 tumor protein p73 [Tupaia chinensis]

MAQSSSTSPDQGATFEHLWSSLEPDSTYFDLPQPSRGNNEEVGSAEASMDVFHLQGMTTSVMAQFNLLSSTMDQMSSRAASASPYTPEHTASVPTHSPYAQPSSTFDTMSPAPVIPSNTDYPGPHHFEVTFQQSSTAKSATWTYSPLLKKLYCQIAKTCPIQIKVSTPPPPGTAIRAMPVYKKAEHVTDIVKRCPNHELGRDFNEGQSAPASHLIRVEGNNLSQYVDDPVTGRQSVVVPYEPPQVGTEFTTILYNFMCNSSCVGGMNRRPILIIITLETRDGQVLGRRSFEGRICACPGRDRKADEDHYREQQALNESTAKNGSTSKRAFKQSPPAIPALGASVKKRRHGDEDTYYMHVRGRENFEILMKVKESLELMELVPQPLVDSYRQQQQLLQRPSHLQPPSYGPVLSPMNKVHGGVNKLPSVNQLVGQPPPHSSAAGPNLGPMGPGMLNNHSHTVPTNGEMNGGHGSQPMVSGSHCTPPPPYHADPSLVSFLTGLGCPNCIEYFTSQGLQNIYHLQNLTIEDLGALKIPDQYRMTIWRGLQDLKQGHDYGPQQLLRSSSNAATISIGSSGELQRQRVMEAVHFRVRHTITIPNRGGGPGGGTGPDEWADFGFDLPDCKARKQPIKEEFTESEIH

>XP_003471295.3 tumor protein p73 isoform X2 [Cavia porcellus]

MAQPSSSSAAPDEGSTFEHLWSSLEPDSTYFDLSQPSQGNTEAVGGAEAGMDVFQLQDVSSPVMAQFNLLSSTMDPMSSRAAPASPYTPEHAASVPTHSPYAQPSSTFDTMSPAPVIPSNTDYPGPHHFEVTFQQSSTAKSATWTYSPMLKKLYCQIAKTCPIQIKVSTPPPPGTVVRAMPVYKKAEHVTDVVKRCPNHELGRDFNEGQSAPASHLIRVEGNNLSQYVDDPVTGRQSVVVPYEPPQVGTEFTTILYNFMCNSSCVGGMNRRPILIIITLETRDGQVLGRRSFEGRICACPGRDRKADEDHYREQQALNESSAKNGVTSKRAFKQSPPAIPALGANMKKRRHGDEDLYYMHVRGRENFEILMKVKESLELMELVPQPLVDSYRQQQQQLLQRPSHLQPPSYGPVLSPMSKAHVGVNKLPSVNQLVGQAPPHTSAAGPSLGPMAPGMLSSHSHAMPANGEMNGGHGSQAMVSGSHCTPPPPYHADPSLVSFLTGLGCPNCIEYFTSQGLQNIYHLQSLTIEDLGALKIPDQYRMTIWRGLQDLKQGHDCGQQLLRSSGNAAATIAIGGSGELQRQRVMEAVHFRVRHTITIPNRGGPSGAASADEWADFGFDLPDCKSRKQPIKEEFPESEGH

>XP_008066928.1 tumor protein p73 isoform X1 [Carlito syrichta]

MAQSTATASEEGTTFEHLWSSLEPDSTYFDLPQSSQENSEVVGGAEPGMDVFHLQGMTTSVMAQFNLLSSTMDQMGSRAASASPYTPEHAASVPTHSPYAQPSSTFDTMSPAPVIPSNTDYPGPHHFEVTFQQSSTAKSATWTYSPMLKKLYCQIAKTCPIQIKVSTPPPPGTAIRAMPVYKKAEHVTDIVKRCPNHELGRDFNEGQSAPASHLIRVEGNNLSQYVDDPVTGRQSVVVPYEPPQVGTEFTTILYNFMCNSSCVGGMNRRPILIIITLETRDGQVLGRRSFEGRICACPGRDRKADEDHYREQQALNESSAKNGAASKRAFKQSPPAVPALGAGVKKRRHADEDTYYLHVRGRENFEVLMKLKESLELMELVPQPLVDSYRQQQQQLLQRPSHLQPPIYGPVLSPMNKVHGGVNKLPSVTQLVGQPPPHSSAAGPNLGPAGPGMLNSHGHTMPANGEMGGGHSSQSMASGSHCTPPPPYHADPSLVSFLTGLGCPNCIEYFTSQGLQNIYHLQNLTIEDLGALKIPDQYRMTIWRGLQDLKQGHDFGAQQLLRSSSNAATISIGGSGELQRQRVMEAVHFRVRHTITIPNRGGPGGGPDEWADFGFDLPDCRARKQPIKEELLEAEIH

>XP_005339273.2 tumor protein p73 isoform X1 [Ictidomys tridecemlineatus]

MSSPRRPDAAATARLRSQEPKASRPPLPGPIDSPGAPAQPRVSCPWKTAQGAMAQSTSTSPDEGATFEHLWSSLEPDSTYFDLPQSSRGSNEAAVGAEPSMDVFQLPGLTTSVMAQFNLLSSTMDQMSSRPAPASPYTPEHAASVPTHSPYAQPSSTFDTMSPAPVIPSNTDYPGPHHFEVTFQQSSTAKSATWTYSPLLKKLYCQIAKTCPIQIKVSTPPPPGTAVRAMPVYKKAEHVTDIVKRCPNHELGRDFNEGQSAPASHLIRVEGNSLSQYVDDPVTGRQSVVVPYEPPQVGTEFTTILYNFMCNSSCVGGMNRRPILIIITLETRDGQVLGRRSFEGRICACPGRDRKADEDHYREQQALNESTTKSVAAGKRAFKQSPPAIPTLGSSVKKRRHGDEDVYYMHVRGRENFEILMKVKESLELMELVPQPLVDSYRQQQQQLLQRPSPLQSPSYGPVLSPMSKVHAGVSKLPSVNQLVGQPPPHSSAAGPNLGPMGPGVLNSHSHTVPANGEMNGGHSSQPMVSGPHCSPPPPYHADPSLVSFLTGLGCPNCIEYFTSQGLQNMYHLQNLTIEDLGALKIPDQYRMTIWRGLQDLKQGHDCGQQLLRSSGNTATISIGGSGELQRQRVMEAVHFRVRHTITIPNRSGPGVAGPDEWADFGFDLPDCRSRKQPIKEEFTEESH

>XP_017398179.1 tumor protein p73 isoform X1 [Cebus imitator]

MAQSTTTSPDGGTTFEHLWSSLEPDSTYFDLPQSSRGTNEAVGGTDSSMDVFHLEGMTTSVMAQFNLLSSTMDQMGSRAASASPYTPEHAASVPTHSPYAQPSSTFDTMSPAPVIPSNTDYPGPHHFEVTFQQSSTAKSATWTYSPLLKKLYCQIAKTCPIQIKVSTPPPPGTAIRAMPVYKKAEHVTDIVKRCPNHELGRDFNEGQSAPASHLIRVEGNNLSQYVDDPVTGRQSVVVPYEPPQVGTEFTTILYNFMCNSSCVGGMNRRPILIIITLETRDGQVLGRRSFEGRICACPGRDRKADEDHYREQQALNESTAKNGAASKRAFKQSPPAVPALGASVKKRRHGDEDTYYLHVRGRENFEILMKLKESLELMELVPQPLVDSYRQQQQLLQRPSHLQPPSYGPVLSPMNKVHGGMNKLPSVNQLVGQPPPHSSAAAPNLGPVGPGMLNNHSHTAPANGEMSSGHGAQAMVSGPHCTPPPPYHADPSLISFLTGLGCPNCIEYFTSQGLQSIYHLQNLTIEDLGALKIPEQYRMTIWRGLQDLKQGHDYGAAQQLLRSSNAATISIGSSGELQRQRVMEAVHFRVRHTITIPNRGGPGGPDEWADFGFDLPDCKARKQPIKEEFAEAEIH

>XP_011765500.1 tumor protein p73 isoform X1 [Macaca nemestrina]

MAQSTTTSPDGGTTFEHLWSSLEPDSTYFDLPQSSRGNNEVVGGTDSSMDVFHLEGMTTSVMAQFNLLSSTMDQMSSRAASASPYTPEHAASVPTHSPYAQPSSTFDTMSPAPVIPSNTDYPGPHHFEVTFQQSSTAKSATWTYSPLLKKLYCQIAKTCPIQIKVSTPPPPGTAIRAMPVYKKAEHVTDIVKRCPNHELGRDFNEGQSAPASHLIRVEGNNLSQYVDDPVTGRQSVVVPYEPPQVGTEFTTILYNFMCNSSCVGGMNRRPILIIITLETRDGQVLGRRSFEGRICACPGRDRKADEDHYREQQALNESSAKNGAAGKRAFKQSPPAVPALGPGVKKRRHGDEDTYYLQVRGRENFEILMKLKESLELMELVPQPLVDSYRQQQQLLQRPSHLQPPSYGPVLSPMNKVHGGMNKLPSVNQLVGQPPPHSSAATPNLGPVGAGMLNNHGHAVPANGEMSSSHSTQSMVSGSHCTPPPPYHADPSLVSFLTGLGCPNCIEYFTSQGLQSIYHLQNLTIEDLGALKIPEQYRMTIWRGLQDLKQGHDYGAAQQLLRSSNAAAISIGGSGELQRQRVMEAVHFRVRHTITIPNRGGPGAGPDEWADFGFDLPDCKARKQPIKEEFTEAEIH

>XP_021512244.1 tumor protein p73 isoform X1 [Meriones unguiculatus]

MDQMGSRAAPASPYTPEHAASAPTHSPYAQPSSTFDTMSPAPVIPSNTDYPGPHHFEVTFQQSSTAKSATWTYSPLLKKLYCQIAKTCPIQIKVSTPPPPGTAIRAMPVYKKAEHVTDIVKRCPNHELGRDFNEGQSAPASHLIRVEGNSLSQYVDDPVTGRQSVVVPYEPPQVGTEFTTILYNFMCNSSCVGGMNRRPILVIITLETRDGQVLGRRSFEGRICACPGRDRKADEDHYREQQALNESATKNGAASKRAFKQSPPAIPALGTNVKKRRHGDEDTFYMHVRGRENFEILMKVKESLELMELVPQPLVDSYRQQQQQLLQRPSHLQPPSYGPVLSPMNKVHSGVNKLPSVNQLVGQPPPHSSAAGPNLGPMGSGMLNSHSHGMPANGEMNGGHSSQTMVSGSHCTPPPPYPADPSLISFLTGLGCPNCIECFTSQGLQSVYHLQNLTIEDLGALKIPDQYRMTIWRGLQDLKQSHDCGQQLLRSSSNSATISIGGSGELQRQRVMEAVHFRVRHTITIPNRGGPGGVTGPDEWADFGFDLPDCKSRKQPIKEEFTETESH

>XP_014993662.2 tumor protein p73 isoform X1 [Macaca mulatta]

MAQSTTTSPDGGTTFEHLWSSLEPDSTYFDLPQSSRGNNEVVGGTDSSMDVFHLEGMTTSVMAQFNLLSSTMDQMSSRAASASPYTPEHAASVPTHSPYAQPSSTFDTMSPAPVIPSNTDYPGPHHFEVTFQQSSTAKSATWTYSPLLKKLYCQIAKTCPIQIKVSTPPPPGTAIRAMPVYKKAEHVTDIVKRCPNHELGRDFNEGQSAPASHLIRVEGNNLSQYVDDPVTGRQSVVVPYEPPQVGTEFTTILYNFMCNSSCVGGMNRRPILIIITLETRDGQVLGRRSFEGRICACPGRDRKADEDHYREQQALNESSAKNGAAGKRAFKQSPPAVPALGPGVKKRRHGDEDTYYLQVRGRENFEILMKLKESLELMELVPQPLVDSYRQQQQLLQRPSHLQPPSYGPVLSPMNKVHGGMNKLPSVNQLVGQPPPHSSAATPNLGPVGAGMLNNHGHAVPANGEMSSSHSTQSMVSGSHCTPPPPYHADPSLVSFLTGLGCPNCIEYFTSQGLQSIYHLQNLTIEDLGALKIPEQYRMTIWRGLQDLKQGHDYSAAQQLLRSSNAAAISIGGSGELQRQRVMEAVHFRVRHTITIPNRGGPGAGPDEWADFGFDLPDCKARKQPIKEEFTEAEIH

>XP_007979045.2 tumor protein p73 isoform X1 [Chlorocebus sabaeus]

MAQSTTTSPDGGTTFEHLWSSLEPDSTYFDLPQSSRGNNEVVGGTDSSMDVFHLEGMTTSVMAQFNLLSSTMDQMSSRAASASPYTPEHAASVPTHSPYAQPSSTFDTMSPAPVIPSNTDYPGPHHFEVTFQQSSTAKSATWTYSPLLKKLYCQIAKTCPIQIKVSAPPPPGTAIRAMPVYKKAEHVTDIVKRCPNHELGRDFNEGQSAPASHLIRVEGNNLSQYVDDPVTGRQSVVVPYEPPQVGTEFTTILYNFMCNSSCVGGMNRRPILIIITLETRDGQVLGRRSFEGRICACPGRDRKADEDHYREQQALNESSAKNGAASKRAFKQSPPAVPALGPGVKKRRHGDEDTYYLQVRGRENFEILMKLKESLELMELVPQPLVDSYRQQQQLLQRPSHLQPPSYGPVLSPMNKVHGGMNKLPSVNQLVGQPPPHSSAATPNLGPVGSGMLNNHGHAVPANGEMTSSHGTQSMVSGSHCTPPPPYHADPSLVSFLTGLGCPNCIEYFTSQGLQSIYHLQNLTIEDLGALKIPEQYRMTIWRGLQDLKQGHDYGAAAQQLLRSSNAAAISIGGSGELQRQRVMEAVHFRVRHTITIPNRGGPGAGPDEWADFGFDLPDCKARKQPIKEEFTEAEIH

>XP_010360079.1 tumor protein p73 isoform X1 [Rhinopithecus roxellana]

MAQSTATSPDGGTTFEHLWSSLEPDSTYFDLPQSSRGNNEVVGGTDSSMDVFHLEGMTTSVMAQFNLLSSTMDQMSSRAASASPYTPEHTASVPTHSPYAQPSSTFDTMSPAPVIPSNTDYPGPHHFEVTFQQSSTAKSATWTYSPLLKKLYCQIAKTCPIQIKVSTPPPPGTAIRAMPVYKKAEHVTDIVKRCPNHELGRDFNEGQSAPASHLIRVEGNNLSQYVDDPVTGRQSVVVPYEPPQVGTEFTTILYNFMCNSSCVGGMNRRPILIIITLETRDGQVLGRRSFEGRICACPGRDRKADEDHYREQQALNESSTKNGAASKRAFKQSPPAVPALGPGVKKRRHGDEDTYYLQVRGRENFEILMKLKESLELMELVPQPLVDSYRQQQQLLQRPSHLQSPSYGPVLSPMNKVHGGMNKLPSVNQLVGQPPPHSSAATPNLGPVGPGMLNNHGHAVPANGEMSSSHGAQSMVSGSHCTPPPPYHADPSLVSFLTGLGCPNCIEYFTSQGLQSIYHLQNLTIEDLGALKIPEQYRMTIWRGLQDLKQGHDCGAAQQLLRSSNAAAISIGGSGELQRQRVMEAVHFRVRHTITIPNRGGPGAGPDEWADFGFDLPDCKARKQPIKEEFTEAEIH

>XP_032118093.1 tumor protein p73 [Sapajus apella]

MAQSTTTSPDGGTTFEHLWSSLEPDSTYFDLPQSSRGTNEAVGGTDSSMDVFHLEGMTTSVMAQFNLLSSTMDQMGSRAASASPYTPEHAASVPTHSPYAQPSSTFDTMSPAPVIPSNTDYPGPHHFEVTFQQSSTAKSATWTYSPLLKKLYCQIAKTCPIQIKVSTPPPPGTAIRAMPVYKKAEHVTDIVKRCPNHELGRDFNEGQSAPASHLIRVEGNNLSQYVDDPVTGRQSVVVPYEPPQVGTEFTTILYNFMCNSSCVGGMNRRPILIIITLETRDGQVLGRRSFEGRICACPGRDRKADEDHYREQQALNESTAKNGAASKRAFKQSPPAVPALGASVKKRRHGDEDTYYLHVRGRENFEILMKLKESLELMELVPQPLVDSYRQQQQLLQRPSHLQPPSYGPVLSPMNKVHGGMNKLPSVNQLVSQPPPHSSAAAPNLGPVGPGMLNNHSHTAPANGEMSSSHGAQAMVSGPHCTPPPPYHADPSLISFLTGLGCPNCIEYFTSQGLQSIYHLQNLTIEDLGALKIPEQYRMTIWRGLQDLKQGHDYGAAQQLLRSSNAATISIGSSGELQRQRVMEAVHFRVRHTITIPNRGGPGGPDEWADFGFDLPDCKARKQPIKEEFAEAEIH

>XP_025258128.1 tumor protein p73 isoform X1 [Theropithecus gelada]

MAQSTTTSPDGGTTFEHLWSSLEPDSTYFDLPQSSRGNNEVVGGTDSSMDVFHLEGMTTSVMAQFNLLSSTMDQMSSRAASASPYTPEHAASVPTHSPYAQPSSTFDTMSPAPVIPSNTDYPGPHHFEVTFQQSSTAKSATWTYSPLLKKLYCQIAKTCPIQIKVSTPPPPGTAIRAMPVYKKAEHVTDIVKRCPNHELGRDFNEGQSAPASHLIRVEGNNLSQYVDDPVTGRQSVVVPYEPPQVGTEFTTILYNFMCNSSCVGGMNRRPILIIITLEARDGQVLGRRSFEGRICACPGRDRKADEDHYREQQALNESSAKNGAAGKRAFKQSPPAVPALGPGVKKRRHGDEDMYYLQVRGRENFEILMKLKESLELMELVPQPLVDSYRQQQQLLQRPSHLQPPSYGPVLSPMNKVHGGMNKLPSVNQLVGQPPPHSSAATPNLGPVGPGMLNNHGHAVPANGEMSSSHSTQSMVSGSHCTPPPPYHADPSLVSFLTGLGCPNCIEYFTSQGLQSIYHLQNLTIEDLGALKIPEQYRMTIWRGLQDLKQGHDYGAAQQLLRSSNAAAISIGGSGELQRQRVMEAVHFRVRHTITIPNRGGPGAGPDEWADFGFDLPDCKARKQPIKEEFTEAEIH

>XP_038378652.1 tumor protein p73 isoform X30 [Canis lupus familiaris]

MSQSTANDEGATFEHLWSSLEPDSTYFDLPQPNQGNSEVVGGAEAGMDVFHLQGMTTPVMSQFNLLSSTMDQMSSRAASASPYTPEHAASVPTHSPYAQPSSTFDTMSPAPAIPSNTDYPGPHHFDVTFQQSSTAKSATWTYSPLLKKLYCQIAKTCPIQIKVSTPPPPGTVIRAMPVYKKAEHVTEVVKRCPNHELGRDFNEGQSAPASHLIRVEGNNLSQYVDDPVTGRQSVMVPYEPPQVGTEFTTILYNFMCNSSCVGGMNRRPILIIITLETRDGQVLGRRSFEGRICACPGRDRKADEDHYREQQALSESAAKNGAASKRAFKQSPPAVPALGANVKKRRHGDEDVYYMHVRGRENFEVLMKVKESLELMELVPQQLVDTYRQQQQQQLLQRPSHLQPASYGPVLSPMNKAHGGVNKLPSVNQLVGQPPPHGSAAGPNLGPMGPGILNNHGHALPASSEMNGSHSAQSMVSGSHCTPPPPYHADPSLVSFLTGLGCPNCIEYFTSQGLQNIYHLQNLTIEDLGALKIPDQYRMTIWRGLQDLKQSHDYGAQQLIRSSSNASTISIGSSGELQRQRVMEAVHFRVRHTITIPNRGGPGGGSGPDEWADFGFDLPDCKSRKQSIKEEFTESEIN

>XP_033051753.1 tumor protein p73 isoform X1 [Trachypithecus francoisi]

MAQSTATSPDGGTTFEHLWSSLEPDSTYFDLPQSSRGNNEVVGGTDSSMDVFHLEGMTTSVMAQFNLLSSTMDQMSSRAASASPYTPEHAASVPTHSPYAQPSSTFDTMSPAPVIPSNTDYPGPHHFEVTFQQSSTAKSATWTYSPLLKKLYCQIAKTCPIQIKVSTPPPPGTAIRAMPVYKKAEHVTDIVKRCPNHELGRDFNEGQSAPASHLIRVEGNNLSQYVDDPVTGRQSVVVPYEPPQVGTEFTTILYNFMCNSSCVGGMNRRPILIIITLETRDGQVLGRRSFEGRICACPGRDRKADEDHYREQQALNESSAKNGAASKRAFKQSPPAVPALGPGVKKRRHGDEDTYYLQVRGRENFEILMKLKESLELMELVPQPLVDSYRQQQQLLQRPSHLQPPSYGPVLSPMNKVHGGMNKLPSVNQLVGQPPPHSSAATPNLGPVGPGMLNNHGHAVPANGEMSSSHGAQSMVSGSHCTPPPPYHADPSLVSFLTGLGCPNCIEYFTSQGLQSIYHLQNLTIEDLGALKIPEQYRMTIWRGLQDLKQGHDYGAAQQLLRSSNAAAISIGGSGELQRQRVMEAVHFRVRHTITIPNRGGPGAGPDEWADFGFDLPDCKARKQPIKEEFTEAEIH

>XP_023070942.1 tumor protein p73 isoform X1 [Piliocolobus tephrosceles]

MAQSTATSPDGGTTFEHLWSSLEPDSTYFDLPQSSRGNNEVVGGTDSSMDVFHLEGMTTSVMAQFNLLSSTMDQMSSRAASASPYTPEHAASVPTHSPYAQPSSTFDTMSPAPVIPSNTDYPGPHHFEVTFQQSSTAKSATWTYSPLLKKLYCQIAKTCPIQIKVSTPPPPGTAIRAMPVYKKAEHVTDIVKRCPNHELGRDFNEGQSAPASHLIRVEGNNLSQYVDDPVTGRQSVVVPYEPPQVGTEFTTILYNFMCNSSCVGGMNRRPILIIITLETRDGQVLGRRSFEGRICACPGRDRKADEDHYREQQALNESSAKNGAASKRAFKQSPPAVPALGPGVKKRRHGDEDTYYLQVRGRENFEILMKLKESLELMELVPQPLVDSYRQQQQLLQRPSHLQPPSYGPVLSPMNKVHGGMNKLPSVNQLVGQPPPHSSAATPNLGPVGPGMLNNHGHAVPANGEMSSSHGAQSMVSGSHCTPPPPYHADPSLVSFLTGLGCSNCIEYFTSQGLQSIYHLQNLTIEDLGALKIPEQYRMTIWRGLQDLKQGHDYGAAQQLLRSSNAAAAISIGGSGELQRQRVMEAVHFRVRHTITIPNRGGPGAGPDEWADFGFDLPDCKARKQPIKEEFTEAEIH

>XP_031519653.1 tumor protein p73 isoform X1 [Papio anubis]

MAQSTTTSPDGGTTFEHLWSSLEPDSTYFDLPQSSRGNNEVVGGTDSSMDVFHLEGMTTSVMAQFNLLSSTMDQMSSRAASASPYTPEHAASVPTHSPYAQPSSTFDTMSPAPVIPSNTDYPGPHHFEVTFQQSSTAKSATWTYSPLLKKLYCQIAKTCPIQIKVSTPPPPGTAIRAMPVYKKAEHVTDIVKRCPNHELGRDFNEGQSAPASHLIRVEGNNLSQYVDDPVTGRQSVVVPYEPPQVGTEFTTILYNFMCNSSCVGGMNRRPILIIITLEARDGQVLGRRSFEGRICACPGRDRKADEDHYREQQALNESSAKNGAAGKRAFKQSPPAVPALGPGVKKRRHGDEDTYYLQVRGRENFEILMKLKESLELMELVPQPLVDSYRQQQQLLQRPSHLQPPSYGPVLSPMNKVHGGMNKLPSVNQLVGQPPPHSSAATPNLGPVGPGMLNNHGHAVPANGEMSSSHSTQSMVSGSHCTPPPPYHADPSLVSFLTGLGCPNCIEYFTSQGLQSIYHLQNLTIEDLGALKIPEQYRMTIWRGLQDLKQGHDYGAAQQLLRSSNAAAISIGGSGELQRQRVMEAVHFRVRHTITIPNRGGPGAGPDEWADFGFDLPDCKARKQPIKEEFTEAEIH

>XP_040120121.1 tumor protein p73 isoform X1 [Oryx dammah]

MSQSTQPAAADEGATFQHLWSSLEPDSTYFDLPQPGQGNEEVAGGAETGMDVFHLPGMTAPVMSQFNLLSSTMDQMSSRAASASPYTPEHAASVPTHSPYAQPSSTFDTMSPAPVIPSNTDYPGPHHFEVTFQQSSTAKSATWTYSPLLKKLYCQIAKTCPIQIKVSAPPPPGTAIRAMPVYKKAEHVTEVVKRCPNHELGRDFNEGQSAPASHLIRVEGNNLSQYVDDPVTGRQSVMVPYEPPQVGTEFTTILYNFMCNSSCVGGMNRRPILIIITLETRDGQVLGRRSFEGRICACPGRDRKADEDHYREQQALNESAAKSGAASKRAFKQSPPTVPALGTNVKKRRHGDDDVYYMHVRGRENFEILMKVKESLELMELVPQQLVDSYRQQQQQLLQRPSHLQPPSYGPVLSPMSKAHGAVNKLPSVNQLVGQPPPHGSVAGPNLGSMGPGILNNHSHTLPANGEMNGGPSSQSMVSGSHCTPPPPYHADPSLVSFLTGLGCPNCIEYFTSQGLQNIYHLQNLTIEDLGTLKIPDQYRMTIWRGLQDLKQSHDYGAQQLIRSSSNAATIAIGGSGELQRQRVMEAVHFRVRHTITIPNRGGPTGGAGPDEWADFGFDLPDCKSRKQSIKEEFTESEAN

>XP_036855689.1 tumor protein p73 isoform X1 [Manis javanica]

MSGAPTHQSELPLGPRVGKMSQAPGTDEGATFEHLWSSLEPDSTYFDLPPSSRGNSEVAGSTEASMDVFHLQGMTTSVMAQFNLLSSTMDQMSSRAASASPYTPEHTASVPTHSPYAQPSSTFDTMSPAPAIPSNADYPGPHHFDVTFQQSSTAKSATWTYSPLLKKLYCQIAKTCPIQIKVSAPPPPGTAVRAMPVYKKAEHVTEVVKRCPNHELGRDFNEGQSAPASHLIRVEGNNLSQYVDDPVTGRQSVMVPYEPPQVGTEFTTILYNFMCNSSCVGGMNRRPILIIITLEARDGQVLGRRSFEGRICACPGRDRKADEDHYREQQALNESAAKNGAASKRAFKQSPPAIPALGASVKKRRHGDEDMYYMHVRGRENFEILMKVKESLELMDLVPQQVVESYRQQQLLQRSSHLQPPTYGPVLNPMNKVHGGVNKLPSVNQLVGQPPPHSSAAGPSLGPMGPGILNSHSHTLPANGEMNGGHSSQSMISGSHCTPPPPYHADPSLVSFLTGLGCPNCIEYFTSQGLQNIYHLQNLTIEDLGALKIPDQYRMTIWRGLQDLKQGQDFIRSSSNASTISIGSSGELQRQRVMEAVHFRVRHTITIPNRGGPGGGSGPEEWADFGFDLPDCKSRKQPIKEEFTESEIH

>XP_030661757.1 tumor protein p73 [Nomascus leucogenys]

MAQSTATSPDGGTTFEHLWSSLEPDSTYFDLPQSSQGNNEVVGGTDSSMDVFHLEGMTTSVMAQFNLLSSTMDQMSSRAASASPYTPEHAASVPTHSPYAQPSSTFDTMSPAPVIPSNTDYPGPHHFEVTFQQSSTAKSATWTYSPLLKKLYCQIAKTCPIQIKVSTPPPPGTAIRAMPVYKKAEHVTDVVKRCPNHELGRDFNEGQSAPASHLIRVEGNNLSQYVDDPVTGRQSVVVPYEPPQVGTEFTTILYNFMCNSSCVGGMNRRPILIIITLETRDGQVLGRRSFEGRICACPGRDRKADEDHYREQQALNESSAKNGAASKRALKQSPPAIPALGAGVKKRRHGDEDTYYLQVRGRENFEILMKLKESLELMELVPQPLVDSYRQQQQLLQRPSHLQPPSYGPVLSPMNKVHGGMNKLPSVNQLVGQPPPHSSAATPNLGPVGPGMLNNHGHTVPANGEMSSSHGAQSIVSGSHCTPPPPYHADPSLVSFLTGLGCPNCIEYFTSQGLQSIYHLQNLTIEDLGALKIPEQYRMTIWRGLQDLKQGHDYSTAQQLLRSSNAATISIGGSGELQRQRVMEAVHFRVRHTITIPNRSGPGSGPDEWADFGFDLPDCKARKQPIKEEFTEAEIH

>XP_029803059.1 tumor protein p73 isoform X1 [Suricata suricatta]

MSQPTAAADEGATFEHLWSSLEPDSTYFDLPQPSQGSSEVVSGAEADMDVFHLQGMTTPVMPPFSLLNSAMDQMSSRAAPGSPYTPEHAASVPTHSPYAQPSSTFDTMSPAPAIPSNADYPGPHHFDVTFQQSSTAKSATWTYSPLLKKLYCQIAKTCPIQVKVSSPPPPGTVVRAMPVYKKAEHVTEVVKRCPNHELGRDFNEGQSAPASHLIRVEGNNLSQYVDDPVTGRQSVMVPYEPPQVGTEFTTILYNFMCNSSCVGGMNRRPILIIITLETRDGQVLGRRSFEGRICACPGRDRKADEDHYREQQALSESAAKNGAASKRAFKQSPPAVPALGPSVKKRRHGDEDVYYMHVRGRENFEILMKVKESLELMELVPQQLVDTYRQQQQQLLQRPSHLQPASYGPVLSPMNKAHGGVNKLPSVNQLVGQPPPHGAAAGPNLGPVGPGILNNHGHTLPANGEMNSGHTAQSMVSGSHCTPPPPYHADPSLVSFLTGLGCPNCIEYFTSQGLQNIYHLQNLTIEDFGNLKIPDQYRMTIWRGLQDLKQSHDYGAQQLIRSSSNAATISIGSSGELQRQRVMEAVHFRVRHTITIPNRGGPGAGSGPDEWADFGFDLPDCKSRKQSIKEEFTESEIN

>XP_035572412.1 tumor protein p73 isoform X8 [Canis lupus dingo]

MPPGCDRTRASRPRGQVQGRCPSPPPTDEGATFEHLWSSLEPDSTYFDLPQPNQGNSEVVGGAEAGMDVFHLQGMTTPVMSQFNLLSSTMDQMSSRAASASPYTPEHAASVPTHSPYAQPSSTFDTMSPAPAIPSNTDYPGPHHFDVTFQQSSTAKSATWTYSPLLKKLYCQIAKTCPIQIKVSTPPPPGTVIRAMPVYKKAEHVTEVVKRCPNHELGRDFNEGQSAPASHLIRVEGNNLSQYVDDPVTGRQSVMVPYEPPQVGTEFTTILYNFMCNSSCVGGMNRRPILIIITLETRDGQVLGRRSFEGRICACPGRDRKADEDHYREQQALSESAAKNGAASKRAFKQSPPAVPALGANVKKRRHGDEDVYYMHVRGRENFEVLMKVKESLELMELVPQQLVDTYRQQQQQQLLQRPSHLQPASYGPVLSPMNKAHGGVNKLPSVNQLVGQPPPHGSAAGPNLGPMGPGILNNHGHALPASSEMNGSHSAQSMVSGSHCTPPPPYHADPSLVSFLTGLGCPNCIEYFTSQGLQNIYHLQNLTIEDLGALKIPDQYRMTIWRGLQDLKQSHDYGAQQLIRSSSNASTISIGSSGELQRQRVMEAVHFRVRHTITIPNRGGPGGGSGPDEWADFGFDLPDCKSRKQSIKEEFTESEIN

>XP_031539479.1 tumor protein p73 isoform X1 [Vicugna pacos]

MSQSTQSAAADEGATFQHLWSSLEPDSTYFDLPQSGRGNAEVAGSAEASMDVFHLQGMTSSVMSQFNLLSSTMDQMSSRAAPASPYTPEHAASVPTHSPYAQPSSTFDTMSPAPVIPSNADYPGPHHFEVTFQQSSTAKSATWTYSPLLKKLYCQIAKTCPVQIKVSTPPPPGTAIRAMPVYKKAEHVTEVVKRCPNHELGRDFNEGXSAPASHLIRVEGNNLSQYVDDPVTGRQSVMVPYEPPQVGTEFTTILYNFMCNSSCVGGMNRRPILIIITLETRDGQVLGRRSFEGRICACPGRDRKADEDHYREQQALNESAAKSGAASKRTFKQSPPAIPALGTSVKKRRHGDEDVYYMHVRGRENFEILMKIKESLELMELVPQQLVDSYRQQQLLQRQSHLQPPSYGPVLSPMNKVHGAVNKLPSVNQLVGQPPPHGSAAGPNLGPMGPGILNNHGHTLQADGEMNGGPSSQPVVSGSHCTPPPAYHADPSLVSFLTGLGCPNCIECFTSQGLQSVYHLQSLTMEDLGALKVPEQYRMTIWRGLQDLKQGHDYGAQQLIRSSSNAATISFGSSGELQRQRVMEAVHFRVRHTITIPNRGGPAGASGPDEWADFGFDLPDCKSRKQSIKEEFTEGEAH

>XP_020730915.1 tumor protein p73 isoform X1 [Odocoileus virginianus texanus]

MSQSTQPAAADEGATFQHLWSSLEPDSTYFDLPQSGQGNEEVAGSAEAGMDVFHLQGISTSVMSQFNLLSSPMDQMSSRAASASPYTPEHTASVPTHSPYAQPSSTFDTMSPAPVIPSNTDYPGPHHFEVTFQQSSTAKSATWTYSPLLKKLYCQIAKTCPIQIKVSTPPPPGTAIRAMPVYKKAEHVTEVVKRCPNHELGRDFNEGQSAPASHLIRVEGNNLSQYVDDPVTGRQSVMVPYEPPQVGTEFTTILYNFMCNSSCVGGMNRRPILIIITLETRDGQVLGRRSFEGRICACPGRDRKADEDHYREQQALNESAAKSGAASKRAFKQSPPTVPALGTNVKKRRHGDDDVYYMHVRGRENFEILMKVKESLELMELVPQQLVDSYRQQQQQLLQRPSHLQPPSYGPVLSPMSKAHGAVNKLPSVNQLVGQPPPHGSAAGSNLGPMGPGILNNHGHTLPANGEMNGGPSSQSMVSGSHCTPPPPYHADPSLVSFLTGLGCPNCIEYFTSQGLQNIYHLQNLTIEDLGALKIPDQYRMTIWRGLQDLKQSHDYSTQQLIRSSSNAATIAIGGSGELQRQRVMEAVHFRVRHTITIPNRGGPAGGAGPDEWADFGFDLPDCKSRKQSIKEEFTESEAN

>XP_036738536.1 tumor protein p73 isoform X1 [Manis pentadactyla]

MSGAPTHQSELPSGPHVGKMSQAPGTDEGATFEHLWSSLEPDSTYFDLPPSSRGNNEVAGSTEASMDVFHLQGMTTSVMAQFNLLSSTMDQMSSRAASASPYTPEHTASVPTHSPYAQPSSTFDTMSPAPAIPSNADYPGPHHFDVTFQQSSTAKSATWTYSPLLKKLYCQIAKTCPIQIKVSTPPPPGTAVRAMPIYKKAEHVTEVVKRCPNHELGRDFNEGQSAPASHLIRVEGNNLSQYVDDPVTGRQSVMVPYEPPQVGTEFTTILYNFMCNSSCVGGMNRRPILIIITLEARDGQVLGRRSFEGRICACPGRDRKADEDHYREQQALNESAAKNGAASKRAFKQSPPAIPALGASVKKRRHGDEDMYYMHVRGRENFEILMKVKESLELMDLVPQQVVESYRQQQLLQRSSHLQPPTYGPVLNPMNKVHGSVNKLPSVNQLVGQPPPHSSAAGPSLGPMGPGILNSHSHTLPANGEMNGGHSSQSMISGSHCTPPPPYHADPSLVSFLTGLGCPNCIEYFTSQGLQNIYHLQNLTIEDLGALKIPDQYRMTIWRGLQDLKQGQDFIRSSSNASTISIGSSGELQRQRVMEAVHFRVRHTITIPNRGGPGGGSGPEEWADFGFDLPDCKSRKQPIKEEFTESEIH

>XP_032614599.1 tumor protein p73 [Hylobates moloch]

MAQSTATSPDGGTTFEHLWSSLEPDSTYFDLPQSSQGNNEVVGGTDSSMDVFHLEGMTTSVMAQFNLLSSTMDQMSSRAASASPYTPEHAASVPTHSPYAQPSSTFDAMSPAPVIPSNTDYPGPHHFEVTFQQSSTAKSATWTYSPLLKKLYCQIAKTCPIQIKVSTPPPPGTAIRAMPVYKKAEHVTDVVKRCPNHELGRDFNEGQSAPASHLIRVEGNNLSQYVDDPVTGRQSVVVPYEPPQVGTEFTTILYNFMCNSSCVGGMNRRPILIIITLETRDGQVLGRRSFEGRICACPGRDRKADEDHYREQQALNESSAKNGAASKRALKQSPPAIPALGAGVKKRRHGDEDTYYLQVRGRENFEILMKLKESLELMELVPQPLVDSYRQQQQLLQRPSHLQPPSYGPVLSPMNKVHGGMNKLPSVNQLVGQPPPHSSAATPNLGPVGPGMLNNHGHTVPANGEMSSSHGAQSIVSGSHCTPPPPYHADPSLVSFLTGLGCPNCIEYFTSQGLQSIYHLQNLTIEDLGALKIPEQYRMTIWRGLQDLKQGHDYSTAQQLLRSSNAATISIGGSGELQRQRVMEAVHFRVRHTITIPNRSGPGSGPDEWADFGFDLPDCKARKQPIKEEFTEAEIH

>ELR61326.1 Tumor protein p73 [Bos mutus]

MSQSTQPAAADEGATFQHLWSSLEPDSTYFDLPQPGQGNEEVAGGAEAGMDVFHLPGMTTSVMSQFNLLSSTMDQMSSRAASASPYTPEHAASVPTHSPYAQPSSTFDTMSPAPVIPSNTDYPGPHHFEVTFQQSSTAKSATWTYSPLLKKLYCQIAKTCPIQIKVSAPPPPGTAIRAMPVYKKAEHVTEVVKRCPNHELGRDFNEGQSAPASHLIRVEGNNLSQYVDDPVTGRQSVMVPYEPPQVGTEFTTILYNFMCNSSCVGGMNRRPILIIITLETRDGQVLGRRSFEGRICACPGRDRKADEDHYREQQALNESAAKSGAASKRAFKQSPPTVPALGTNVKKRRHGDDDVYYIHVRGRENFEILMKVKESLELMELVPQQLVDSYRQQQQQLLQRPSHLQPPSYGPVLSPMSKAHGAVNKLPSVNQLVGQPPPHGSAAGPNLGPMGPGILNNHGHTLPANGEMNGGPSSQSMVSGSHCTPPPPYHADPSLVSFLTGLGCPNCIEYFTSQGLQNIYHLQNLTIEDLGALKIPDQYRMTIWRGLQDLKQSHDYSAQQLIRSSSNAATIAIGGSGELQRQRVMEAVHFRVRHTITIPNRGGPAGGAGPDEWADFGFDLPDCKSRKQSIKEEFTESEAN

**>NP_005418.1 tumor protein p73 isoform a [Homo sapiens]**

MAQSTATSPDGGTTFEHLWSSLEPDSTYFDLPQSSRGNNEVVGGTDSSMDVFHLEGMTTSVMAQFNLLSSTMDQMSSRAASASPYTPEHAASVPTHSPYAQPSSTFDTMSPAPVIPSNTDYPGPHHFEVTFQQSSTAKSATWTYSPLLKKLYCQIAKTCPIQIKVSTPPPPGTAIRAMPVYKKAEHVTDVVKRCPNHELGRDFNEGQSAPASHLIRVEGNNLSQYVDDPVTGRQSVVVPYEPPQVGTEFTTILYNFMCNSSCVGGMNRRPILIIITLEMRDGQVLGRRSFEGRICACPGRDRKADEDHYREQQALNESSAKNGAASKRAFKQSPPAVPALGAGVKKRRHGDEDTYYLQVRGRENFEILMKLKESLELMELVPQPLVDSYRQQQQLLQRPSHLQPPSYGPVLSPMNKVHGGMNKLPSVNQLVGQPPPHSSAATPNLGPVGPGMLNNHGHAVPANGEMSSSHSAQSMVSGSHCTPPPPYHADPSLVSFLTGLGCPNCIEYFTSQGLQSIYHLQNLTIEDLGALKIPEQYRMTIWRGLQDLKQGHDYSTAQQLLRSSNAATISIGGSGELQRQRVMEAVHFRVRHTITIPNRGGPGGGPDEWADFGFDLPDCKARKQPIKEEFTEAEIH

>XP_034809657.1 tumor protein p73 isoform X1 [Pan paniscus]

MAQSTATSPDGGTTFEHLWSSLEPDSTYFDLPQSSRGNNEVVGGTDSSMDVFHLEGMTTSVMAQFNLLSSTMDQMSSRAASASPYTPEHAASVPTHSPYAQPSSTFDTMSPAPVIPSNTDYPGPHHFEVTFQQSSTAKSATWTYSPLLKKLYCQIAKTCPIQIKVSTPPPPGTAIRAMPVYKKAEHVTDVVKRCPNHELGRDFNEGQSAPASHLIRVEGNNLSQYVDDPVTGRQSVVVPYEPPQVGTEFTTILYNFMCNSSCVGGMNRRPILIIITLEMRDGQVLGRRSFEGRICACPGRDRKADEDHYREQQALNESSAKNGAASKRAFKQSPPAVPALGAGVKKRRHGDEDTYYLQVRGRENFEILMKLKESLELMELVPQPLVDSYRQQQQLLQRPSHLQPPSYGPVLSPMNKVHGGMNKLPSVNQLVGQPPPHSSAATPNLGPVGPGMLNNHGHAVPANGEMSSSHSAQSMVSGSHCTPPPPYHADPSLVSFLTGLGCPNCIEYFTSQGLQSIYHLQNLTIEDLGALKIPEQYRMTIWRGLQDLKQGHDYSTTQQLLRSSNAATISIGGSGELQRQRVMEAVHFRVRHTITIPNRGGPGGGPDEWADFGFDLPDCKARKQPIKEEFTEAEIH

>XP_016808303.1 tumor protein p73 isoform X1 [Pan troglodytes]

MAQSTATSPDGGTTFEHLWSSLEPDSTYFDLPQSSRGNNEVVGGTDSSMDVFHLEGMTTSVMAQFNLLSSTMDQMSSRAASASPYTPEHAASVPTHSPYAQPSSTFDTMSPAPVIPSNTDYPGPHHFEVTFQQSSTAKSATWTYSPLLKKLYCQIAKTCPIQIKVSTPPPPGTAIRAMPVYKKAEHVTDVVKRCPNHELGRDFNEGQSAPASHLIRVEGNNLSQYVDDPVTGRQSVVVPYEPPQVGTEFTTILYNFMCNSSCVGGMNRRPILIIITLEMRDGQVLGRRSFEGRICACPGRDRKADEDHYREQQALNESSAKNGAASKRAFKQSPPAVPALGAGVKKRRHGDEDTYYLQVRGRENFEILMKLKESLELMELVPQPLVDSYRQQQQLLQRPSHLQPPSYGPVLSPMNKVHGGMNKLPSVNQLVGQPPPHSSAATPNLGPVGPGMLNNHGHAVPANGEMGSSHSAQSMVSGSHCTPPPPYHADPSLVSFLTGLGCPNCIEYFTSQGLQSIYHLQNLTIEDLGALKIPEQYRMTIWRGLQDLKQGHDYSTTQQLLRSSNAATISIGGSGELQRQRVMEAVHFRVRHTITIPNRGGPGGGPDEWADFGFDLPDCKARKQPIKEEFTEAEIH

>XP_024110563.1 tumor protein p73 isoform X2 [Pongo abelii]

MAQSTATSPDGGTTFEHLWSSLEPDSTYFDLPQSSRGNNEVVGGTDSSMDVFHLEGMTTSVMAQFNLLSSTMDQMSSRAASASPYTPEHAASVPTHSPYAQPSSTFDTMSPAPVIPSNTDYPGPHHFEVTFQQSSTAKSATWTYSPLLKKLYCQIAKTCPIQIKVSTPPPPGTAIRAMPVYKKAEHVTDVVKRCPNHELGRDFNEGQSAPASHLIRVEGNNLSQYVDDPVTGRQSVVVPYEPPQVGTEFTTILYNFMCNSSCVGGMNRRPILIIITLETRDGQVLGRRSFEGRICACPGRDRKADEDHYREQQALNESSAKNGATSKRAFKQSPPAVPALGAGVKKRRHGDEDTYYLQVRGRENFEILMKLKESLELMELVPQPLVDSYRQQQQLLQRPSHLQPPSYGPVLSPMNKVHGGMNKLPSVNQLVGQPPPHSSAATPNLGPVGPGMLNNHGHAVPANGEMSSSHGAQSMVSGSHCTPPPPYHADPSLVSFLTGLGCPNCIEYFTSQGLQSIYHLQNLTIEDLGALKIPEQYRMTIWRGLQDLKQGHDYSTAQQLLRSSNAATISIGGSGELQRQRVMEAVHFRVRHTITIPNRGGPGGGPEEWADFGFDLPDCKARKQPIKEEFTEAEIH

>XP_035163368.1 tumor protein p73 isoform X1 [Callithrix jacchus]

MAQSTTTSPDGGTTFEHLWSSLEPDSTYFDLPQSSRGNNEVVGGTDSSMDVFHLEGMTTSVMAQFNLLSSTMDQMSSRAASASPYTPEHAASVPTHSPYAQPSSTFDTMSPAPVIPSNTDYPGPHHFEVTFQQSSTAKSATWTYSPLLKKLYCQIAKTCPIQIKVSTPPPPGTAIRAMPVYKKAEHVTDIVKRCPNHELGRDFNEGQSAPASHLIRVEGNNLSQYVDDPVTGRQSVVVPYEPPQVGTEFTTILYNFMCNSSCVGGMNRRPILIIITLETRDGQVLGRRSFEGRICACPGRDRKADEDHYREQQALNDSTAKNGAASKRAFKQSPPAVPALGASVKKRRHGDEDTYYLHVRGRENFEILMKLKESLELMELVPQPLVDSYRQQQQLLQRPSHLQPPSYGPVLSPMNKVHGGMNKLPSVNQLVGQPPPHSSAAAPNLGPVGPGMLNNHSHTAPTNGEMSSGHGTQAMVSGPHCTPPPPYHADPSLVSFLTGLGCPNCIEYFTSQGLQSIYHLQNLTIEDLGALKIPEQYRMTIWRGLQDLKQGHDYGTAQQLLRSSNAATISIGSSGELQRQRVMEAVHFRVRHTITIPNRGGPGGPEEWADFGFDLPDCKARKQPIKEEFSEAEIH

>XP_012316413.1 tumor protein p73 isoform X1 [Aotus nancymaae]

MAQSTTTSPDGGTTFEHLWSSLEPDSTYFDLPQSSRENNEVVGGTDSSMDIFHLEGMTTSVMAQFNLLSSTMDQMSSRAASASPYTPEHAASVPTHSPYAQPSSTFDTMSPAPVIPSNTDYPGPHHFEVTFQQSSTAKSATWTYSPLLKKLYCQIAKTCPIQIKVSTPPPPGTAIRAMPIYKKAEHVTDIVKRCPNHELGRDFNEGQSAPASHLIRVEGNNLSQYVDDPVTGRQSVVVPYEPPQVGTEFTTILYNFMCNSSCVGGMNRRPILIIITLETRDGQVLGRRSFEGRICACPGRDRKADEDHFREQQALNESTAKNGAASKRAFKQSPPAIPALGASMKKRRHGDEDTYYLHVRGRENFEILMKLKESLELMELVPQPLVDSYRQQQQLLQRPSHLQPPSYGPVLSPMNKVHGGMNKLPSVNQLVGQPPPHSSAAAPNLGPVGPGMLNNHSHTAPTNGEMSSSHGAQAMVSGPHCTPPPPYHADPSLVSFLTGLGCPNCIEYFTSQGLQSVYHLQNLTIEDLGALKIPEQYRMTIWRGLQDLKQGHDYGAAQQLLRSSNAATISIGSSGELQRQRVMEAVHFRVRHTITIPNRGGPGGPDEWADFGFDLPDCKARKQPIKEEFAESEIH

>XP_010348609.1 tumor protein p73 isoform X1 [Saimiri boliviensis boliviensis]

MAQSTTTSPDGGTTFEHLWSSLEPDSTYFDLPQSSRGNNEVVGGTDSSMDVFHLEGMTTSVMAQFNLLSSTMDQMSSRAASASPYTPEHAASVPTHSPYAQPSSTFDTMSPAPVIPSNTDYPGPHHFEVTFQQSSTAKSATWTYSPLLKKLYCQIAKTCPIQIKVSTPPPPGTAIRAMPVYKKAEHVTDIVKRCPNHELGRDFNEGQSAPASHLIRVEGNNLSQYVDDPVTGRQSVVVPYEPPQVGTEFTTILYNFMCNSSCVGGMNRRPILIIITLETRDGQVLGRRSFEGRICACPGRDRKADEDHYREQQALNESTAKNGAASKRAFKQSPPAVPALGASVKKRRHGDEDTYYLHVRGRENFEILMKLKESLELMELVPQPLVDSYRQQQQLLQRQSHLQPPSYGPVLSPVNKVHGGMTKLPSVNQLVGQPPPHSSAATPNLGPVGPGMLNNHSHTAPTNGDINSGHSAQAMLSGPHCTPPPPYHADPSLVSFLTGLGCPNCIEYFTSQGLQSIYHLQNLTIEDLGALKIPEQYRMTIWRGLQDLKQGQDYGAAQQLLRSSNAATISIGSSGELQRQRVMEAVHFRVRHTITIPNRGGPGGPDEWADFGFDLPDCKARKQPIKEEFAEAEIH

>XP_025860926.1 tumor protein p73 [Vulpes vulpes]

MSQSTANDEGATFEHLWSSLEPDSTYFDLPQPNQGNSEVVGGAEAGMDVFHLQGMTTPVMPQFNLLSSTMDQMSSRAASASPYTPEHAASVPTHSPYAQPSSTFDTMSPAPAIPSNTDYPGPHHFDVTFQQSSTAKSATWTYSPLLKKLYCQIAKTCPIQIKVSTPPPPGTVIRAMPVYKKAEHVTEVVKRCPNHELGRDFNEGQSAPASHLIRVEGNNLSQYVDDPVTGRQSVMVPYEPPQVGTEFTTILYNFMCNSSCVGGMNRRPILIIITLETRDGQVLGRRSFEGRICACPGRDRKADEDHYREQQALSESAAKNGAASKRAFKQSPPAVPALGANVKKRRHGDEDVYYMHVRGRENFEVLMKVKESLELMELVPQQLVDTYRQQQQQQLLQRPSHLQPASYGPVLSPMNKAHGGVNKLPSVNQLVGQPPPHGSAAGPNLGPMGPGILNNHGHALPASSEMNGSHSAQSMVSGSHCTPPPPYHADPSLVSFLTGLGCPNCIEYFTSQGLQNIYHLQNLTIEDLGALKIPDQYRMTIWRGLQDLKQSHDYGAQQLIRSSSNASTISIGSSGELQRQRVMEAVHFRVRHTITIPNRGGPGGGSGPDEWADFGFDLPDCKSRKQSIKEEFTESEIN

>XP_034876150.1 tumor protein p73 [Mirounga leonina]

MSQPNTVDEGATFEHLWNSLEPDSTYFDLPQSSQGNNEVVGGAEAGMDVFHLQGMTTPVMSQFNLLSSTMDQMSSRAASASPYTPEHATSVPTHSPYAQPSSTFDTMSPAPAIPSNTDYPGPHHFDVTFQQSSTAKSATWTYSPLLKKLYCQIAKTCPIQIKVSAPPPPGTIVRAMPVYKKAEHVTEVVKRCPNHELGRDFNEGQSAPASHLIRVEGNNLSQYVDDPVTGRQSVMVPYEPPQVGTEFTTILYNFMCNSSCVGGMNRRPILIIITLETRDGQVLGRRSFEGRICACPGRDRKADEDHQREQQALSESAAKHGAASKRAFKQSPPAVPALGTNVKKRRHGDEDVYYMHVRGRENFEILMKVKESLELMELVPQQLVDTYRQQQQLLQRPNHLQPASYGPVLSPMNKAHAAVNKLPSVNQLVGQPPPHSSAAGPNLGPMGPGILNNHGHALPTSGEMNSSHGAQSMVSGSHCTPPPPYHADPSLVSFLTGLGCPNCIEYFTSQGLQNIYHLQNLTIEDLGALKIPDQHRMTIWRGLQDLKQSHDYGAQQLIRSSSNASTISIGSSGELQRQRVMEAVHFRVRHTITIPNRGAPAGGAGPDEWADFGFDLPDCKSRKQSIKEEFMESEIH

>VFV32341.1 tumor protein p73 isoform a [Lynx pardinus]

MSLPRRPGRCLHRLPAQPRAALGLPAASASAPTPTHPRGPGSAKSQLPSGPGARKMSQPTAAADEGATFEHLWRSLEPDSTYFDLPQPSQGNNAVVSGSGASMDVLHLQGMTTPVMPQFNLLNSTMDQMSSRAASASPYTPEHAASVPTHSPYAQPSSTFDTMSPAPAIPSNADYPGPHHFDVTFQQSSTAKSATWTYSPLLKKLYCQIAKTCPIQIKVSTPPPPGTVIRAMPVYKKAEHVTEVVKRCPNHELGRDFNEGQSAPASHLIRVEGNNLSQYVDDPVTGRQSVMVPYEPPQVGTEFTTILYNFMCNSSCVGGMNRRPILIIITLETRDGQVLGRRSFEGRICACPGRDRKADEDHYREQQALSESAAKNGAGSKRAFKQSPPAVPALGTNVKKRRHGDEDVYYMHVRGRENFEILMKVKESLELMELVPQQLVDAYRQQQQQLQRPSHLQPASYGPVLSPMNKAHGGVNKLPSVNQLVGQPPPHGSAAGPNLGPVGPGILNNHSHALPANGEMNSGHGAQSMVSGSHCTPPPYHADPSLVSFLTGLGCPNCIEYFTSQGLQNIYHLQNLTIEDLGALKIPDQYRMTIWRGLQDLKQSHDYGAQQLIRSSSNASAIAVGSSGELQRQRVMEAVHFRVRHTITIPNRGGPGPDEWADFGFDLPDCKSRKQSIKEEFTESEIN

>XP_026916437.1 tumor protein p73 isoform X1 [Acinonyx jubatus]

MSQPTAAADEGATFEHLWRSLEPDSTYFDLPQPSQGNNAVVSGSGASMDVLHLQGMTTPVMPQFNLLNSTMDQMSSRAASASPYTPEHAASVPTHSPYAQPSSTFDTMSPAPAIPSNADYPGPHHFDVTFQQSSTAKSATWTYSPLLKKLYCQIAKTCPIQIKVSTPPPPGTVIRAMPVYKKAEHVTEVVKRCPNHELGRDFNEGQSAPASHLIRVEGNNLSQYVDDPVTGRQSVMVPYEPPQVGTEFTTILYNFMCNSSCVGGMNRRPILIIITLETRDGQVLGRRSFEGRICACPGRDRKADEDHYREQQALSESAAKNGAGSKRAFKQSPPAVPALGTNVKKRRHGDEDVYYMHVRGRENFEILMKVKESLELMELVPQQLVDAYRQQQQQLQRPSHLQPASYGPVLSPMNKAHGGVNKLPSVNQLVGQPPPHGSAAGPNLGPVGPGILNNHSHALPANGEMNSGHGAQSMVSGSHCTPPPYHADPSLVSFLTGLGCPNCIEYFTSQGLQNIYHLQNLTIEDLGALKIPDQYRMTIWRGLQDLKQSHDYGAQQLIRSSSNASAIAVGSSGELQRQRVMEAVHFRVRHTITIPNRGGPGPDEWADFGFDLPDCKSRKQSIKEEFTESEIN

>XP_030184168.1 tumor protein p73 isoform X1 [Lynx canadensis]

MSLPRRPGRCLHRLPAQPRAALGLPAASASAPTPTHPRGPGSTKSQLPSGPGARKMSQPTAAADEGATFEHLWRSLEPDSTYFDLPQPSQGNNAVVSGSGASMDVLHLQGMTTPVMPQFNLLNSTMDQMSSRAASASPYTPEHAASVPTHSPYAQPSSTFDTMSPAPAIPSNADYPGPHHFDVTFQQSSTAKSATWTYSPLLKKLYCQIAKTCPIQIKVSTPPPPGTVIRAMPVYKKAEHVTEVVKRCPNHELGRDFNEGQSAPASHLIRVEGNNLSQYVDDPVTGRQSVMVPYEPPQVGTEFTTILYNFMCNSSCVGGMNRRPILIIITLETRDGQVLGRRSFEGRICACPGRDRKADEDHYREQQALSESAAKNGAGSKRAFKQSPPAVPALGTNVKKRRHGDEDVYYMHVRGRENFEILMKVKESLELMELVPQQLVDAYRQQQQQLQRPSHLQPASYGPVLSPMNKAHGGVNKLPSVNQLVGQPPPHGSAAGPNLGPVGPGILNNHSHALPANGEMNSGHGAQSMVSGSHCTPPPYHADPSLVSFLTGLGCPNCIEYFTSQGLQNIYHLQNLTIEDLGALKIPDQYRMTIWRGLQDLKQSHDYGAQQLIRSSSNASAIAVGSSGELQRQRVMEAVHFRVRHTITIPNRGGPGPDEWADFGFDLPDCKSRKQSIKEEFTESEIN

>XP_035945452.1 tumor protein p73 isoform X2 [Halichoerus grypus]

MSAHLPPPSCLHSWTPESEPPSGPGAEKMSQPNTVDEGATFEHLWNSLEPDSTYFDLPQSSQGNNEVVGGAEAGMDVFHLQGMTTPVMSQFNLLSSTMDQMSSRAASASPYTPEHATSVPTHSPYAQPSSTFDTMSPAPAIPSNTDYPGPHHFDVTFQQSSTAKSATWTYSPLLKKLYCQIAKTCPIQIKVSAPPPPSTIVRAMPVYKKAEHVTEVVKRCPNHELGRDFNEGQSAPASHLIRVEGNNLSQYVDDPVTGRQSVMVPYEPPQVGTEFTTILYNFMCNSSCVGGMNRRPILIIITLETRDGQVLGRRSFEGRICACPGRDRKADEDHHREQQALSESAAKHGAASKRAFKQSPPAVPALGTNVKKRRHGDEDVYYMHVRGRENFEILMKVKESLELMELVPQQLVDTYRQQQQLLQRPNHLQPASYGPVLSPMNKAHVAVNKLPSVNQLVGQPPPHSSAAGPNLGPVGPGILNNHGHALPTSGEVNSSHGAQSMVSGSHCTPPPPYHADPSLVSFLTGLGCPNCIEYFTSQGLQNIYHLQNLTIEDLGALKIPDQYRMTIWRGLQDLKQSHDYGAQQLIRSSSNASTISIGSSGELQRQRVMEAVHFRVRHTITIPNRGAPAGGAGPDEWADFGFDLPDCKSRKQSIKEEFMESEIH

>XP_040315225.1 tumor protein p73 isoform X2 [Puma yagouaroundi]

MSQPTAAADEGATFEHLWRSLEPDSTYFDLPQPSQGNNAVVSGSGASMDVLHLQGMTTPVMPQFNLLNSTMDQMSSRAASASPYTPEHAASVPTHSPYAQPSSTFDTMSPAPAIPSNADYPGPHHFDVTFQQSSTAKSATWTYSPLLKKLYCQIAKTCPVQIKVSTPPPPGTVIRAMPVYKKAEHVTEVVKRCPNHELGRDFNEGQSAPASHLIRVEGNNLSQYVDDPVTGRQSVMVPYEPPQVGTEFTTILYNFMCNSSCVGGMNRRPILIIITLETRDGQVLGRRSFEGRICACPGRDRKADEDHYREQQALSESAAKNGAGSKRAFKQSPPAVPALGTNVKKRRHGDEDVYYMHVRGRENFEILMKVKESLELMELVPQQLVDAYRQQQQQLQRPSHLQPASYGPVLSPMNKAHGGVNKLPSVNQLVGQPPPHGSAAGPNLGPVGPGILNNHSHALPANGEMNSGHGAQSMVSGSHCTPPPYHADPSLVSFLTGLGCPNCIEYFTSQGLQNIYHLQNLTIEDLGALKIPDQYRMTIWRGLQDLKQSHDYGAQQLIRSSSNASAIAVGSSGELQRQRVMEAVHFRVRHTITIPNRGGPGPDEWADFGFDLPDCKSRKQSIKEEFTESEIN

>XP_003793263.1 tumor protein p73 isoform X1 [Otolemur garnettii]

MAQSTATSPDEGATFEHLWSSLEPDSTYFDLPQSSRGNNQLVGSAETSMDVFHLQGMTTSVMAQFNLLSSTMDQMSSRAAPASPYTPEHAAPVPTHSPYAQPSSTFDTMSPAPVIPSNTDYPGPHHFEVTFQQSSTAKSATWTYSPLLKKLYCQIAKTCPIQIKVSTPPPPGTAIRAMPVYKKAEHVTDIVKRCPNHELGRDFNEGQSAPASHLIRVEGNNLSQYVDDPVTGRQSVVVPYEPPQVGTEFTTILYNFMCNSSCVGGMNRRPILIIITLETRDGQVLGRRSFEGRICACPGRDRKADEDHYLEQQTLNERNEGAARSSAASKRTFKQSPPTVPALGTSVKKRRHGDEDIYYLPVRGRENFEILMKVKESLELVDLVPQPLVDSYRQQQQQLLQRPSHLQPPSYGPVLSPMNKVHGGSNKLPPVNQLVGQPPSHSSAPGPSLGPVGPGVLTNHSHALPANGEANGSHSTQPMVSGSHCTPPPPYHADPSLVSFLTGLGCPNCIEYFTSQGVQNIYHLQNLTIEDLGALKIPDQYRMTIWRGLQDLKQGHDYGAQQLLRSSSNAATISIGGSGELQRQRVMEAVHFRVRHTITIPNRGGPGGGAAPEEWADFGFDLPDCKARKQPIKEELSEGEVH

>XP_006748231.1 tumor protein p73 [Leptonychotes weddellii]

MSQPNTVDEGATFEHLWNSLEPDSTYFDLPQSSQGNNEVVGGAEAGMDVFHLQGMTTPVMSQFNLLSSTMDQMSSRAASASPYTPEHATSVPTHSPYAQPSSTFDTMSPAPAIPSNTDYPGPHHFDVTFQQSSTAKSATWTYSPLLKKLYCQIAKTCPIQIKVSAPPPPGTIVRAMPVYKKAEHVTEVVKRCPNHELGRDFNEGQSAPASHLIRVEGNNLSQYVDDPVTGRQSVMVPYEPPQVGTEFTTILYNFMCNSSCVGGMNRRPILIIITLETRDGQVLGRRSFEGRICACPGRDRKADEDHQREQQALSESAAKHGAASKRAFKQSPPAVPALGTNVKKRRHGDEDVYYMHVRGRENFEILMKVKESLELMELVPQQLVDTYRQQQQLLQRPNHLQPASYGPVLSPMNKAHTAVNKLPSVNQLVGQPLPHSSAAGPNLGPMGPGILNNHSHALPTSGEMNSSHGAQSMVSGSHCTPPPPYHADPSLVSFLTGLGCPNCIEYFTSQGLQNIYHLQNLTIEDLGALKIPDQHRMTIWRGLQDLKQSHDYGAQQLLRSSSNASTISIGSSGELQRQRVMEAVHFRVRHTITIPNRGAPAGGAGPDEWADFGFDLPDCKSRKQSIKEEFMESEIH

**>XP_025067916.1 tumor protein p73 isoform X1 [Alligator sinensis]**

MPSWAGGASCSRLPARTDGTAARAIYPLSDSPVTKMSQASPADEGTTFEHLWSTLEPDSTYFDLPTSSHSGSSEVSNHTEVTMDVFQMRNTNESVMSQFNLLNSSMDQSIGSRAASTSPYNSEHTSNVPTHSPYSQPSSTFDAMSPAPVIPSNTDYPGPHHFEVTFQQSSTAKSATWTYSPLLKKLYCQIAKTCPIQIKVSTPPPPGTIIRAMPVYKKAEHVTEVVKRCPNHELGRDFNDGQSAPASHLIRVEGNNLSQYVDDPVTGRQSVMVPYEPPQVGTEFTTILYNFMCNSSCVGGMNRRPILIIITLETRDGQVLGRRSFEGRICACPGRDRKADEDHYREQQALNESAAKNGNANKRTFKQSPQGIPALGAGVKKRRHGEEEMYYVPVRGRENFEILMKIKESLELVELVPQQLVDSYRQQQQQLLQRQSHLQSPSSYGPVLSPMNKVHGGGINKLPSVNQLVGQPPQHSSNSGPNLGPMGPGMMNSHPMQANGEMNGGHSSQSMVSGSHCTPPPPYNPDPSLVSFLTGLGCPNCIDYFTSQGLQNIYHLQNLTIEDLGALKIPEQYRMIIWRGLQELKQSHDYGAQQLIRSSSNASTISIGSSGELQRQRVMEAVHFRVRHTITIPNRSGADEWADFGFDLPDCKSRKQSIKEEFTEGEIN

>XP_032619613.1 tumor protein p73 isoform X1 [Chelonoidis abingdonii]

MEPGVIYPLSDSPSVKMSQSSPADEGTTFEHLWSTLEPDSTYFDLPPSSHSGSSEVSNQTEVTMDVFQMRSMNESVMSQFNLLNSSMDQSIGSRAASTSPYNSEHPSNVPTHSPYSQPSSTFDAMSPAPVIPSNADYPGPHHFEVTFQQSSTAKSATWTYSPLLKKLYCQIAKTCPIQIKVSTPPPPGTVIRAMPVYKKAEHVTEVVKRCPNHELGRDFNDGQSAPASHLIRVEGNNLSQYVDDPVTGRQSVMVPYEPPQVGTEFTTILYNFMCNSSCVGGMNRRPILIIITLETRDGQVLGRRSFEGRICACPGRDRKADEDHYREQQALNENAAKNGNINKRTFKQSPQGIPALGAGVKKRRHGEEEMYYVPVRGRENFEILMKIKESLELVELVPQQLVDSYRQQQQQLLQRQSHLQSPSSYGPVLSPMNKVHGGGINKLPSVNQLVGQPPQHNSSSGPNLGPMGPGMLNSHPMQSNGDMNGGHSSQSMVSGSHCTPPPPYNPDPSLVSFLTGLGCPNCIDYFTSQGLQNIYHLQNLTIEDLGALKIPEQYRMIIWRGLQELKQSHDYGAQQLIRSSSNASTISIGSSGELQRQRVMEAVHFRVRHTITIPNRSGADEWADFGFDLPDCKSRKQSIKEEFTEGEIN

>XP_030393277.1 tumor protein p73 isoform X1 [Gopherus evgoodei]

MEPGVIYPLSDSPSVKMSQSSPADEGTTFEHLWSTLEPDSTYFDLPPSSHSGSSEVSNQTEVAMDVFQMRSMNESVMSQFNLLNSSMDQSIGSRAASTSPYNSEHPSNVPTHSPYSQPSSTFDAMSPAPVIPSNADYPGPHHFEVTFQQSSTAKSATWTYSPLLKKLYCQIAKTCPIQIKVSTPPPPGTVIRAMPVYKKAEHVTEVVKRCPNHELGRDFNDGQSAPASHLIRVEGNNLSQYVDDPVTGRQSVMVPYEPPQVGTEFTTILYNFMCNSSCVGGMNRRPILIIITLETRDGQVLGRRSFEGRICACPGRDRKADEDHYREQQALNENAAKNGNINKRTFKQSPQGIPALGAGVKKRRHGEEEMYYVPVRGRENFEILMKIKESLELVELVPQQLVDSYRQQQQQLLQRQSHLQSPSSYGPVLSPMNKVHGGGINKLPSVNQLVGQPPQHNSSSGPNLGPMGPGMLNSHPMQSNGDMNGGHSSQSMVSGSHCTPPPPYNPDPSLVSFLTGLGCPNCIDYFTSQGLQNIYHLQNLTIEDLGALKIPEQYRMIIWRGLQELKQSHDYGAQQLIRSSSNASTISIGSSGELQRQRVMEAVHFRVRHTITIPNRSGADEWADFGFDLPDCKSRKQSIKEEFTEGEIN

>XP_039365362.1 tumor protein p73 isoform X1 [Mauremys reevesii]

MEPGVIYPLSDSPSVKMSQSSPADEGTTFEHLWSTLEPDSTYFDLPPSSHSGSSEVSNQTEVTMDVFQMRGMNESVMSQFNLLNSSMDQSIGSRAASTSPYNSEHPSNVPTHSPYSQPSSTFDAMSPAPVIPSNADYPGPHHFEVTFQQSSTAKSATWTYSPLLKKLYCQIAKTCPIQIKVSTPPPPGTVIRAMPVYKKAEHVTEVVKRCPNHELGRDFNDGQSAPASHLIRVEGNNLSQYVDDPVTGRQSVMVPYEPPQVGTEFTTILYNFMCNSSCVGGMNRRPILIIITLETRDGQVLGRRSFEGRICACPGRDRKADEDHYREQQALNENAAKNGNINKRTFKQSPQGIPALGAGVKKRRHGEEEMYYVPVRGRENFEILMKIKESLELVELVPQQLVDSYRQQQQQLLQRQSHLQSPSSYGPVLSPMNKVHGGGINKLPSVNQLVGQPPQHNSSSGPNLGPMGPGMLNSHPMQSNGDMNGGHSSQSMVSGSHCTPPPPYNPDPSLVSFLTGLGCPNCIDYFTSQGLQNIYHLQNLTIEDLGALKIPEQYRMIIWRGLQELKQSHDYGAQQLIRSSSNASTISIGSSGELQRQRVMEAVHFRVRHTITIPNRSGADEWADFGFDLPDCKSRKQSIKEEFTEGEIN

>XP_007070222.1 tumor protein p73 isoform X1 [Chelonia mydas]

MEPGVIYPLSDSPSVKMSQSSPADEGTTFEHLWSTLEPDSTYFDLPPSSHSGSSEVSNQTEVTMDVFQMRSMNESVMSQFNLLNSSMDQSIGTRAASTSPYNSEHPSNVPTHSPYSQPSSTFDAMSPAPVIPSNADYPGPHHFEVTFQQSSTAKSATWTYSPLLKKLYCQIAKTCPIQIKVSTPPPPGTVIRAMPVYKKAEHVTEVVKRCPNHELGRDFNDGQSAPASHLIRVEGNNLSQYVDDPVTGRQSVMVPYEPPQVGTEFTTILYNFMCNSSCVGGMNRRPILIIITLETRDGQVLGRRSFEGRICACPGRDRKADEDHYREQQALNENAAKNGNVNKRTFKQSPQGIPALGAGIKKRRHGEEEMYYVPVRGRENFEILMKIKESLELVELVPQQLVDSYRQQQQQLLQRQSHLQSPSSYGPVLSPMNKVHGGGINKLPSVNQLVGQPPQHNSSSGPNLGPMGPGMLNSHPMQSNGDMNGGHSSQSMVSGSHCTPPPPYNPDPSLVSFLTGLGCPNCIDYFTSQGLQNIYHLQNLTIEDLGALKIPEQYRMIIWRGLQELKQSHDYGAQQLIRSSSNAATISIGSSGELQRQRVMEAVHFRVRHTITIPNRSGADEWADFGFDLPDCKSRKQSIKEEFTEGEIN

>XP_025941433.1 tumor protein p73 isoform X1 [Apteryx rowi]

MEPGAIYPLSDSPSVKMSQSSPADEGTTFEHLWSTLEPDSTYFDLPPSNHTGSHEVSDRTEVTMDVFQMRSMNDSVMSQFNLLNNSMDQSIGSRAASTSPYNSEHTSNVPTHSPYSQPSSTFDAMSPAPVIPSNTDYPGPHHFEVTFQQSSTAKSATWTYSPLLKKLYCQIAKTCPIQIKVSTPPPPGTIIRAMPVYKKAEHVTEVVKRCPNHELGRDFNDGQSAPASHLIRVEGNNLSQYVDDPVTGRQSVMVPYEPPQVGTEFTTILYNFMCNSSCVGGMNRRPILIIITLETRDGQVLGRRSFEGRICACPGRDRKADEDHYREQQALNESAAKNGNANKRTFKQSPQGIPALGAGVKKRRHGEEEMYYVPVRGRENFEILMKIKESLELVELVPQQLVDSYRQQQQQLLQRQSHLQTPSSYGPVLSPMNKVHGGGINKLPSVNQLVGQPPQHSSNSAPSLGPMGPGMLNSHPMQPNGEMNGGHSSQSMVSGSHCTPPPPYNPDPSLVSFLTGLGCPNCIDYFTSQGLQNIYHLQNLSIEDLGALKIPEQYRMIIWRGLQDLKQSHDYGAQQLIRSSSNASTISIGSSGELQRQRVMEAVHFRVRHTITIPNRSGADEWADFGFDLPDCKSRKQSIKEEFTEGEIN

>XP_023960040.1 tumor protein p73 isoform X1 [Chrysemys picta bellii]

MKDWRWIPLQKVIYPLSDSPSVKMSQSSPADEGTTFEHLWSTLEPDSTYFDLPPSSHSGSSEVSNQTEVTMDVFQMRSMNESVMSQFNLLNSSMDQSIGSRAASTSPYNSEHPSNVPTHSPYSQPSSTFDAMSPAPVIPSNADYPGPHHFEVTFQQSSTAKSATWTYSPLLKKLYCQIAKTCPIQIKLSTPPPPGTVIRAMPVYKKAEHVTEVVKRCPNHELGRDFNDGQSAPASHLIRVEGNNLSQYVDDPVTGRQSVMVPYEPPQVGTEFTTILYNFMCNSSCVGGMNRRPILIIITLETRDGQVLGRRSFEGRICACPGRDRKADEDHYREQQALNENAAKNGNINKRTFKQSPPGIPALGAGVKKRRHGEEEMYYVPVRGRENFEILMKIKESLELVELVPQQLVDSYRQQQQQLLQRQSHLQSPSSYGPVLSPMNKVHGGGINKLPSVNQLVGQPPQHNSSSGPNLGPMGPGMLNSHPMQSNGDMNGGHSSQAMVSGSHCTPPPPYNPDPSLVSFLTGLGCPNCIDYFTSQGLQNIYHLQNLTIEDLGALKIPEQYRMIIWRGLQELKQSHDYGAQQLIRSSSNASTISIGSSGELQRQRVMEAVHFRVRHTITIPNRSGADEWADFGFDLPDCKSRKQSIKEEFTEGEIN

>XP_024063650.1 tumor protein p73 isoform X2 [Terrapene carolina triunguis]

MSQSSPADEGTTFEHLWSTLEPDSTYFDLPPSSHSGSSEVSNQTEVTMDVFQMRSMNESVMSQFNLLNSSMDQSIGSRAASTSPYNSEHPSNVPTHSPYSQPSSTFDAMSPAPVIPSNADYPGPHHFEVTFQQSSTAKSATWTYSPLLKKLYCQIAKTCPIQIKVSTPPPPGTVIRAMPVYKKAEHVTEVVKRCPNHELGRDFNDGQSAPASHLIRVEGNNLSQYVDDPVTGRQSVMVPYEPPQVGTEFTTILYNFMCNSSCVGGMNRRPILIIITLETRDGQVLGRRSFEGRICACPGRDRKADEDHYREQQALNENAAKNGNVNKRTFKQSPPGIPALGAGVKKRRHGEEEMYYVPVRGRENFEILMKIKESLELVELVPQQLVDSYRQQQQQLLQRQSHLQSPSSYGPVLSPMNKVHGGGINKLPSVNQLVGQPPQHNSSSGPNLGPMGPGMLNSHPMQSNGDMNGGHSSQAMVSGSHCTPPPPYNPDPSLVSFLTGLGCPNCIDYFTSQGLQNIYHLQNLTIEDLGALKIPEQYRMIIWRGLQELKQSHDYGAQQLIRSSSNASTISIGSSGELQRQRVMEAVHFRVRHTITIPNRSGADEWADFGFDLPDCKSRKQSIKEEFTEGEIN

>NXG38953.1 P73 protein [Dromaius novaehollandiae]

VKMSQSSPADEGTTFEHLWSTLEPDSTYFDLPPSNHTGSHEVSNRTEVTMDVFQMRSMNDSVMSQFNLLNNSMDQSIGSRAASTSPYSSEHTSNVPTHSPYSQPSSTFDAMSPAPVIPSNTDYPGPHHFEVTFQQSSTAKSATWTYSPLLKKLYCQIAKTCPIQIKVSPPPPPGTIVRAMPVYKKAEHVTEVVKRCPNHELGRDFNDGQSAPASHLIRVEGNNLSQYVDDPVTGRQSVMVPYEPPQVGTEFTTILYNFMCNSSCVGGMNRRPILIIITLETRDGQVLGRRSFEGRICACPGRDRKADEDHYREQQALNESAAKNGNANKRTFKQSPQGIPALGAGIKKRRHGEEEMYYVPVRGRENFEILMKIKESLELVELVPQQLVDSYRQQQQQLLQRQSHLQTPSSYGPVLSPMNKVHGGGINKLPSVNQLVGQPPQHSSGSAPSLGPMGPGMLNSHPMQPNGEMNGGHSSQSMVSGSHCTPPPPYNPDPSLVSFLTGLGCPNCIDYFTSQGLQNIYHLQNLSIEDLGALKIPEQYRMIIWRGLQDLKQSHDYGAQQLIRSSSNASTISIGSSGELQRQRVMEAVHFRVRHTITIPNRSGADEWADFGFDLPDCKSRKQSIKEEFTEGEIN

>NXU26446.1 P73 protein [Thalassarche chlororhynchos]

VKMSQSSPADEGTTFEHLWSTLEPDSTYFDLPPSNHTGSNEVSNRTEVTMDVFQMRSMNDSVMSQFNLLNNSMDQSIGSRAASTSPYSSEHTSNVPTHSPYSQPSSTFDAMSPAPVIPSNTDYPGPHHFEVTFQQSSTAKSATWTYSPLLKKLYCQIAKTCPIQIKVSTPPPPGTIIRAMPVYKKAEHVTEVVKRCPNHELGRDFNDGQSAPASHLIRVEGNNLSQYVDDPVTGRQSVMVPYEPPQVGTEFTTILYNFMCNSSCVGGMNRRPILIIITLETRDGQVLGRRSFEGRICACPGRDRKADEDHYREQQALNESAAKNGNANKRTFKQSPQGIPALGAGIKKRRHGEEEMYYVPVRGRENFEILMKIKESLELVELVPQQLVDSYRQQQQQLLQRQSQLQTPSSYGPVLSPMNKVHGGGINKLPSVNQLVGQPAQHSSSSAPSLGPMGPGMLNSHPMQPNGEMNGGHSSQSMVSGSHCTPPPPYNPDPSLVSFLTGLGCPNCIDYFTSQGLQNIYHLQNLSIEDLGALKIPEQYRMIIWRGLQELKQSHDYGAQQLIRSSSNASTISIGSSGELQRQRVMEAVHFRVRHTITIPNRGGADEWADFGFDLPDCKSRKQSIKEEFTEGEIN

>NXC75400.1 P73 protein [Anhinga anhinga]

VKMSQSSPADEGTTFEHLWSTLEPDSTYFDLPPSNHTGSSEVSNRTEVTMDIFQMRSMNDSVMSQFNLLNNSMDQSIGSRAASTSPYSSEHTSNVPTHSPYSQPSSTFDAMSPAPVIPSNTDYPGPHHFEVTFQQSSTAKSATWTYSPLLKKLYCQIAKTCPIQIKVSTPPPPGTIIRAMPVYKKAEHVTEVVKRCPNHELGRDFNDGQSAPASHLIRVEGNNLSQYVDDPVTGRQSVMVPYEPPQVGTEFTTILYNFMCNSSCVGGMNRRPILIIITLETRDGQVLGRRSFEGRICACPGRDRKADEDHYREQQALNESAAKNGNANKRTFKQSPQGIPALGAGVKKRRHGEEEMYYVPVRGRENFEILMKIKESLELVELVPQQLVDSYRQQQQQLLQRQSQLQTPSSYGPVLSPMNKVHSGGINKLPSVNQLVGQPAQHSSSSAPGLGPMGPGMLNSHPMQPNGEMNGGHSSQSMVSGSHCTPPPPYNPDPSLVSFLTGLGCPNCIDYFTSQGLQNIYHLQNLSIEDLGALKIPEQYRMIIWRGLQELKQSHDYGAQQLIRSSSNASTISIGSSGELQRQRVMEAVHFRVRHTITIPNRGGADEWADFGFDLPDCKSRKQSIKEEFTEGEIN

>NXF42587.1 P73 protein [Oceanites oceanicus]

VKMSQSSPADEGTTFEHLWSTLEPDSTYFDLPPSNHTGSNEVSNRTEVTMDVFQMRSMSDSVMSQFNLLNNSMDQSIGSRAASTSPYSSEHTSNVPTHSPYSQPSSTFDAMSPAPVIPSNTDYPGPHHFEVTFQQSSTAKSATWTYSPLLKKLYCQIAKTCPIQIKVSTPPPPGTIIRAMPVYKKAEHVTEVVKRCPNHELGRDFNDGQSAPASHLIRVEGNNLSQYVDDPVTGRQSVMVPYEPPQVGTEFTTILYNFMCNSSCVGGMNRRPILIIITLETRDGQVLGRRSFEGRICACPGRDRKADEDHYREQQALNESAAKNGNANKRTFKQSPQGIPALGAGIKKRRHGEEEMYYVPVRGRENFEILMKIKESLELVELVPQQLVDSYRQQQQQLLQRQSQLQTPSSYGPVLSPMNKVHGGGINKLPSVNQLVGQPAQHSSSSAPSLGPMGPGMLNSHPMQPNGEMNGGHSSQSMVSGSHCTPPPPYNPDPSLVSFLTGLGCPNCIDYFTSQGLQNIYHLQNLSIEDLGALKIPEQYRMIIWRGLQELKQSHDYGAQQLIRSSSNASTISIGSSGELQRQRVMEAVHFRVRHTITIPNRGGADEWADFGFDLPDCKSRKQSIKEEFTEGEIN

>NWH25045.1 P73 protein [Grus americana]

AKMSQSSPADEGTTFEHLWSTLEPDSTYFDLPPSNHPGSNEVSNRTEVTMDVFQMRSMNDSVMSQFNLLNNSMDQSIGSRAASTSPYSSEHTSNVPTHSPYSQPSSTFDAMSPAPVIPSNTDYPGPHHFEVTFQQSSTAKSATWTYSPLLKKLYCQIAKTCPIQIKVSTPPPPGTIIRAMPVYKKAEHVTEVVKRCPNHELGRDFNDGQSAPASHLIRVEGNNLSQYVDDPVTGRQSVMVPYEPPQVGTEFTTILYNFMCNSSCVGGMNRRPILIIITLETRDGQVLGRRSFEGRICACPGRDRKADEDHYREQQALNESAAKNGNANKRTFKQSPQGIPALGAGIKKRRHGEEEMYYVPVRGRENFEILMKIKESLELVELVPQQLVDSYRQQQQQLLQRQSQLQTPSSYGPVLSPMNKVHGGGINKLPSVNQLVGQPAQHSSSSAPSLGPMGPGMLNSHPMQPNGEMNGGHSSQSMVSGSHCTPPPPYNPDPSLVSFLTGLGCPNCIDYFTSQGLQNIYHLQNLSIEDLGALKIPEQYRMIIWRGLQELKQSHDYGAQQLIRSSSNASTISIGSSGELQRQRVMEAVHFRVRHTITIPNRGGADEWADFGFDLPDCKSRKQSIKEEFTEGEIN

>NXT39263.1 P73 protein [Pelecanoides urinatrix]

VKMSQSSPADEGTTFEHLWSTLEPDSTYFDLPPSNHPGSNEVSNRTEVTMDVFQMRSMNDSVMSQFNLLNNSMDQSIGSRAASTSPYSSEHTSNVPTHSPYSQPSSTFDAMSPAPVIPSNTDYPGPHHFEVTFQQSSTAKSATWTYSPLLKKLYCQIAKTCPIQIKVSTPPPPGTIIRAMPVYKKAEHVTEVVKRCPNHELGRDFNDGQSAPASHLIRVEGNNLSQYVDDPVTGRQSVMVPYEPPQVGTEFTTILYNFMCNSSCVGGMNRRPILIIITLETRDGQVLGRRSFEGRICACPGRDRKADEDHYREQQALNESAAKNGNANKRTFKQSPQGIPALGAGIKKRRHGEEEMYYVPVRGRENFEILMKIKESLELVELVPQQLVDSYRQQQQQLLQRQSQLQTPSSYGPVLSPMNKVHGGGINKLPSVNQLVGQPAQHSSSSAPSLGPMGPGMLNSHPMQPNGEMNGGHSSQSMVSGSHCTPPPPYNPDPSLVSFLTGLGCPNCIDYFTSQGLQNIYHLQNLSIEDLGALKIPEQYRMIIWRGLQELKQSHDYGAQQLIRSSSNASTISIGSSGELQRQRVMEAVHFRVRHTITIPNRGGADEWADFGFDLPDCKSRKQSIKEEFTEGEIN

>NXT89293.1 P73 protein [Anhinga rufa]

VKMSQSSPADEGTTFEHLWSTLEPDSTYFDLPPSNHTGSNEVSNRTEVTMDVFQMRSMNDSVMSQFNLLNNSMDQSIGSRAASTSPYSSEHTSNVPTHSPYSQPSSTFDAMSPAPVIPSNTDYPGPHHFEVTFQQSSTAKSATWTYSPLLKKLYCQIAKTCPIQIKVSTPPPPGTIIRAMPVYKKAEHVTEVVKRCPNHELGRDFNDGQSAPASHLIRVEGNNLSQYVDDPVTGRQSVMVPYEPPQVGTEFTTILYNFMCNSSCVGGMNRRPILIIITLETRDGQVLGRRSFEGRICACPGRDRKADEDHYREQQALNESAAKNGNANKRTFKQSPQGIPALGAGVKKRRHGEEEMYYVPVRGRENFEILMKIKESLELVELVPQQLVDSYRQQQQQLLQRQSQLQTPSSYGPVLSPMNKVHSGGINKLPSVNQLVGQPAQHSSSSAPGLGPMGPGMLNSHPMQPNGEMNGGHSSQSMVSGSHCTPPPPYNPDPSLVSFLTGLGCPNCIDYFTSQGLQNIYHLQNLSIEDLGALKIPEQYRMIIWRGLQELKQSHDYGAQQLIRSSSNASTISIGSSGELQRQRVMEAVHFRVRHTITIPNRGGADEWADFGFDLPDCKSRKQSIKEEFTEGEIN

>NXE76313.1 P73 protein [Cochlearius cochlearius]

VKMSQSSPADEGTTFEHLWSTLEPDSTYFDLPPSNHTGSNEVSNRTEVTMDVFQMRSMNDSVMSQFNLLNNSMDQSIGSRAASTSPYSSEHTSNVPTHSPYSQPSSTFDAMSPAPVIPSNTDYPGPHHFEVTFQQSSTAKSATWTYSPLLKKLYCQIAKTCPIQIKVSTPPPPGTIIRAMPVYKKAEHVTEVVKRCPNHELGRDFNDGQSAPASHLIRVEGNNLSQYVDDPVTGRQSVMVPYEPPQVGTEFTTILYNFMCNSSCVGGMNRRPILIIITLETRDGQVLGRRSFEGRICACPGRDRKADEDHYREQQALNESAAKNGNANKRTFKQSPQGIPALGAGIKKRRHGEEEMYYVPVRGRENFEILMKIKESLELVELVPQQLVDSYRQQQQQLLQRQSQLQTPSSYGPVLSPMNKVHGGGINKLPSVNQLVGQPAQHGSSSAPSLGPMGPGMLNSHPMQPNGEMNGGHSSQSMVSGSHCTPPPPYNPDPSLVSFLTGLGCPNCIDYFTSQGLQNIYHLQNLSIEDLGALKIPEQYRMIIWRGLQELKQSHDYGAQQLIRSSSNASTISIGSSGELQRQRVMEAVHFRVRHTITIPNRGGADEWADFGFDLPDCKSRKQSIKEEFTEGEIN

>NXQ93690.1 P73 protein [Sagittarius serpentarius]

KMSQSSPADEGTTFEHLWSTLEPDSTYFDLPPSNHTGSDEVSNRTEVTMDVFQMRSMNDSVMSQFNLLNNSMDQSIGSRAASTSPYSSEHTSNVPTHSPYSQPSSTFDAMSPAPVIPSNTDYPGPHHFEVTFQQSSTAKSATWTYSPLLKKLYCQIAKTCPIQIKVSTPPPPGTIIRAMPVYKKAEHVTEVVKRCPNHELGRDFNDGQSAPASHLIRVEGNNLSQYVDDPVTGRQSVMVPYEPPQVGTEFTTILYNFMCNSSCVGGMNRRPILIIITLETRDGQVLGRRSFEGRICACPGRDRKADEDHYREQQALNESAAKNGNANKRTFKQSPQGIPALGAGVKKRRHGEEEMYYVPVRGRENFEILMKIKESLELVELVPQQLVDSYRQQQQQLLQRQSQLQTPSSYGPVLSPMNKVHGGGINKLPSVNQLVGQPAQHGSSSAPSLGPMGPGMLNSHPMQPNGEMNGGHSSQSMVSGSHCTPPPPYNPDPSLVSFLTGLGCPNCIDYFTSQGLQNIYHLQNLSIEDLGALKIPEQYRMIIWRGLQELKQSHDYGAQQLIRSSSNASTISIGSSGELQRQRVMEAVHFRVRHTITIPNRGGADEWADFGFDLPDCKSRKQSIKEEFTEGEIN

>XP_040389772.1 tumor protein p73 isoform X1 [Cygnus olor]

MESNMDTGVIYPLSDSPSVKMSESSPADEGTTFEHLWSTLEPDSTYFDLPPSSHAGSSEASNRTEVTMDVFQLRGINDSVMSQFNLLNNSMDQSIGSRAASTSPYSSEHTSNVPTHSPYSQPSSTFDAMSPAPVIPSNTDYPGPHHFEVTFQQSSTAKSATWTYSPLLKKLYCQIAKTCPIQIKVSTPPPPGTIIRAMPVYKKAEHVTEVVKRCPNHELGRDFNDGQSAPASHLIRVEGNNLSQYVDDPVTGRQSVMVPYEPPQVGTEFTTILYNFMCNSSCVGGMNRRPILIIITLEMRDGQVLGRRSFEGRICACPGRDRKADEDHYREQQALNESAAKNGNANKRTFKQSPQGIPALGTGIKKRRHGEEEMYYVPVRGRENFEILMKIKESLELVELVPQQLVDSYRQQQQQLLQRHRSQLQTPSSYGPVLSPMNKAHGGGINKLPSVNQLVGQPPQHSSSSAPSLGPMGPGMLNSHPMQPNGEMNGGHSSQSMVSGSHCTPPPPYNPDPSLVSFLTGLGCPNCIDYFTSQGLQNIYHLQNLSIEDLGALKIPEQYRMIIWRGLQELKQSHDYGAQQLIRSSSNASTISIGSSGELQRQRVMEAVHFRVRHTITIPNRGGADEWADFGFDLPDCKSRKQSIKEEFTEGEIN

>XP_029874169.1 tumor protein p73 isoform X1 [Aquila chrysaetos chrysaetos]

MKDWRWIPLQKVIYPLSDSPSAKMSQSSPADEGTTFEHLWSTLEPDSTYFDLPPSNHTGSNEVSNRTEVTMDVFQMRSMNDSVMSQFNLLNNSMDQSIGSRAASTSPYSSEHTSNVPTHSPYSQPSSTFDAMSPAPVIPSNTDYPGPHHFEVTFQQSSTAKSATWTYSPLLKKLYCQIAKTCPIQIKVSTPPPPGTIIRAMPVYKKAEHVTEVVKRCPNHELGRDFNDGQSAPASHLIRVEGNNLSQYVDDPVTGRQSVMVPYEPPQVGTEFTTILYNFMCNSSCVGGMNRRPILIIITLETRDGQVLGRRSFEGRICACPGRDRKADEDHYREQQALNESAAKNGNANKRTFKQSPQGIPALGTGIKKRRHGEEEMYYVPVRGRENFEILMKIKESLELVELVPQQLVDSYRQQQQQLLQRQSQLQTPSSYGPVLSPMNKVHGGGLNKLPSVNQLVGQPAQHSSGSAPSLGPMGPGMLNSHPMQPNGEMNGGHSSQSMVSGSHCTPPPPYNPDPSLVSFLTGLGCPNCIDYFTSQGLQNIYHLQNLSIEDLGALKIPEQYRMIIWRGLQELKQSHDYGAQQLIRSSSNASTISIGSSGELQRQRVMEAVHFRVRHTITIPNRGGADEWADFGFDLPDCKSRKQSIKEEFTEGEIN

>XP_038022658.1 tumor protein p73 isoform X1 [Anas platyrhynchos]

MKDWRWIPLQKVIYPLSDSPSAKMSESSPADEGTTFEHLWSTLEPDSTYFDLPPSSHAGSSEVSNRTEVTMDVFQLRGINDSVMSQFNLLNNSMDQSIGSRAASASPYSSEHTSNVPTHSPYSQPSSTFDAMSPAPVIPSNTDYPGPHHFEVTFQQSSTAKSATWTYSPLLKKLYCQIAKTCPIQIKVSTPPPPGTIIRAMPVYKKAEHVTEVVKRCPNHELGRDFNDGQSAPASHLIRVEGNNLSQYVDDPVTGRQSVMVPYEPPQVGTEFTTILYNFMCNSSCVGGMNRRPILIIITLEMRDGQVLGRRSFEGRICACPGRDRKADEDHYREQQALNESAAKNGNANKRTFKQSPQGIPALGTGIKKRRHGEEEMYYVPVRGRENFEILMKIKESLELVELVPQQLVDSYRQQQQQLLQRQSQLQTPSSYGPVLSPMNKVHGGGINKLPSVNQLVGQPPQHSSSSAPSLGPMGPGMLNSHPMQPNGEMNGGHSSQSMVSGSHCTPPPPYNPDPSLVSFLTGLGCPNCIDYFTSQGLQNIYHLQNLSIEDLGALKIPEQYRMIIWRGLQELKQSHDYGAQQLIRSSSNASTISIGSSGELQRQRVMEAVHFRVRHTITIPNRGGADEWADFGFDLPDCKSRKQSIKEEFTEGEIN

>XP_006124749.1 tumor protein p73 isoform X1 [Pelodiscus sinensis]

MKDWRWIPLQKAIYPLSDSPSVKMSQSSPVDEGTTFEHLWSTLEPDSTYFDLPPSSHSGESEVSNQAEVTMDVFQMRSMNESVLSQFNLLNSSMDQSIGSRATSTSPYSSEHPSNVPTHSPYSQPSSTFDAMSPAPVIPSNADYPGPHHFEVTFQQSSTAKSATWTYSPLLKKLYCQIAKTCPIQIKVATPPPPGTAIRAMPVYKKAEHVTEVVKRCPNHELGRDFNDGQAAPASHLIRVEGNNLSQYVDDPVTGRQSVMVPYEPPQVGTEFTTILYNFMCNSSCVGGMNRRPILIIITLETRDGQVLGRRSFEGRICACPGRDRKADEDHFREQQALNENAAKNGNVNKRTFKQSPQGIPALGAGIKKRRHGEEEMYYVPVRGRENFEILMKIKESLELVELVPQQLVDSYRQQQQQLLQRQSHLQPPSSYGPVLSPINKVHGGGINKLPSVNQLVGQPPQHSSSSGPNLGPMGPGMLNSHSMQPNGDLNGGHSSQSMVSGSHCTPPPPYNPDPSLVSFLTGLGCPNCIDYFTSQGLQNIYHLQNLTIEDLGALKIPEQYRMIIWRGLQELKQSHDYGAQQLIRSSSNASTISIGSSGELQRQRVMEAVHFRVRHTITIPNRSGADEWSDFGFDLPDCKSRKQSIKEEFTEGEIN

>XP_012425837.3 tumor protein p73 isoform X1 [Taeniopygia guttata]

MEEGVIYPLSDSPPVKMSQSSPADEGPTFEHLWSTLEPDSTYFDLPPANPSGSNEVSNRTEVTMDVFQMRGMTDSVMSQFNLLNNSMDQSIGSRAASTSPYSSEHTSNVPTHSPYSQPSSTFDTMSPAPVIPSNTDYPGPHHFEVTFQQSSTAKSATWTYSPLLKKLYCQIAKTCPIQIKVSTSPPPGTIIRAMPVYKKAEHVTEVVKRCPNHELGRDFNDGQSAPASHLIRVEGNNLSQYVDDPVTGRQSVMVPYEPPQVGTEFTTILYNFMCNSSCVGGMNRRPILIIITLETRDGQVLGRRSFEGRICACPGRDRKADEDHFREQQALNESTAKNGNANKRTFKQSPQGIPALGTGIKKRRHGEEEMYYVPVQGRENFEILMKIKESLELVELVPQQLVDSYRQQQQQLLQRQNQLQTASSYGPVLSPMNKVHGGGINKLPSVNQLVGQPAQHGSSSAPSLGPMGPGMLNSHPMQTNGEMNGGHSSQSMVSGSHCTPPPPYNADPSLVSFLTGLGCPNCIDYFTSQGLQNIYHLQNLSIEDLAALKIPEQYRMLIWRGLQELKQSHDYGAQQLIRSSSNASTISIGSSGELQRQRVMEAVHFRVRHTITIPNRGAADDWADFGFDLPDCKSRKQSIKEEFTEGEIN

>NWW31210.1 P73 protein [Panurus biarmicus]

VKMSQSSPADEGTTFEHLWSTLEPDSTYFDLPPANPTGSNEVSNRTEVTMDVFQMRGMTDSVMSQFNLLNNSMDQSIGSRAASTSPYSSEHTSNVPTHSPYSQPSSTFDAMSPAPVIPSNTDYPGPHHFEVTFQQSSTAKSATWTYSPLLKKLYCQIAKTCPIQIKVSTSPPPGTIIRAMPVYKKAEHVTEVVKRCPNHELGRDFNDGQSAPASHLIRVEGNNLSQYVDDPVTGRQSVMVPYEPPQVGTEFTTILYNFMCNSSCVGGMNRRPILIIITLETRDGQVLGRRSFEGRICACPGRDRKADEDHFREQQALNESTAKNGNANKRTFKQSPQGIPALGTGIKKRRHGEEEMYYVPVRGRENFEILMKIKESLELVELVPQQLVDSYRQQQQQLLQRQSQLQTPSSYGPVLSPMNKVHGGGINKLPSVNQLVGQPAQHSSSSAPSLGPMGPGMLNSHPMQTNGEMNGGHSSQSMVSGSHCTPPPPYSADPSLVSFLTGLGCPNCIDYFTSQGLQNIYHLQNLSIEDLGALKIPEQYRMIIWRGLQELKQSHDYGAQQLIRSSSNASTISIGSSGELQRQRVMEAVHFRVRHTITIPNRGAADDWADFGFDLPDCKSRKQSIKEEFTEGEIN

>NXR51614.1 P73 protein [Hippolais icterina]

VKMSQSSPADEGTTFEHLWSTLEPDSTYFDLPPANPSGSSEVSNRTEVTMDVFQMRDIPDSVMSQFNLLNNSMDQSIGSRAASTSPYSSEHTSNVPTHSPYSQPSSTFDAMSPAPVIPSNTDYPGPHHFEVTFQQSSTAKSATWTYSPLLKKLYCQIAKTCPIQIKVSTSPPPGTIIRAMPVYKKAEHVTEVVKRCPNHELGRDFNDGQAAPASHLIRVEGNNLSQYVDDPVTGRQSVMVPYEPPQVGTEFTTILYNFMCNSSCVGGMNRRPILIIITLETRDGQVLGRRSFEGRICACPGRDRKADEDHFREQQALNESTAKNGNANKRTFKQSPQGIPALGTGIKKRRHGEEEMYYVPVRGRENFEILMKIKESLELVELVPQQLVDSYRQQQQQLLQRQSQLQTPSSYGPVLSPMNKVHGGGINKLPSVNQLVGQPAQHSSSSAPSLGPMGPGMLNSHPMQTNGEMNGGHSSQSMVSGSHCTPPPPYSADPSLVSFLTGLGCPNCIDYFTSQGLQNIYHLQNLSIEDLGALKIPEQYRMIIWRGLQELKQSHDYGAQQLIRSSSNASTISIGSSGELQRQRVMEAVHFRVRHTITIPNRGAADDWADFGFDLPDCKSRKQSIKEEFTEGEIN

>XP_023796165.1 tumor protein p73 [Cyanistes caeruleus]

MEEGVIYPLSDSPTVKMSQSSPADEGTTFEHLWSTLEPDSTYFDLPPANPTGSNEVSNRTEVTMDVFQMRGMTDSVMSQFNLLNNSMDQSIGSRAASTSPYSSEHTSNVPTHSPYSQPSSTFDAMSPAPVIPSNTDYPGPHHFEVTFQQSSTAKSATWTYSPLLKKLYCQIAKTCPIQIKVSTSPPPGTIIRAMPVYKKAEHVTEVVKRCPNHELGRDFNDGQSAPASHLIRVEGNNLSQYVDDPVTGRQSVMVPYEPPQVGTEFTTILYNFMCNSSCVGGMNRRPILIIITLETRDGQVLGRRSFEGRICACPGRDRKADEDHFREQQALNESAAKNGNANKRTFKQSPQGIPALGTGIKKRRHGEEEMYYVPVRGRENFEILMKIKESLELVELVPQQLVDSYRQQQQQLLQRQSQLQTPSSYGPVLSPMNKVHGGGINKLPSVNQLVGQPAQHSSSSAPSLGPMGPGMLNSHPMQTNGEMNGGHSSQSMVSGSHCTPPPPYNPDPSLVSFLTGLGCPNCIDYFTSQGLQNIYHLQNLSIEDLGALKIPEQYRMIIWRGLQELKQSHDYGAQQLIRSSSNASTISIGSSGELQRQRVMEAVHFRVRHTITIPNRGAADDWADFGFDLPDCKSRKQSIKEEFTEGEIN

>XP_021230291.1 tumor protein p73 isoform X1 [Numida meleagris]

MLPIPRAMESSMEGGVIYPLSDSPSVKMSQSSPADESTTFEHLWSTLAPDSTYFDLSPSSHTGSSEVSNRTEVTMDVFQMRSMNDSVMSQFNLLNNSMDQSIGSRAASTSPYSSEHTSNVPTHSPYSQPSSTFDAMSPAPVIPSNTDYPGPHHFEVTFQQSSTAKSATWTYSPLLKKLYCQIAKTCPIQIKVSSPPPPGTIIRAMPVYKKAEHVTEVVKRCPNHELGRDFNDGQSAPASHLIRVEGNNLSQYVDDPVTGRQSVMVPYEPPQVGTEFTTILYNFMCNSSCVGGMNRRPILIIITLETRDGQVLGRRSFEGRICACPGRDRKADEDHYREQQALNENAAKNGNANKRTFKQSPQAIPALGPGVKKRRHGEEEMYYVPVRGRENFEILMKIKESLELVELVPQQLVDSYRQQQQQLLQRQNQLQTPSSYGPVLSPMNKVHGGGINKLPSVNQLVGQPAQHSSSSAPSLGPMGPGMLNSHSMQPNGEMNGGHSSQSMVSGSHCTPPPPYNPDPSLVSFLTGLGCPNCIDYFTSQGLQNIYHLQNLSIEDLGALKIPEQYRMIIWRGLQELKQSHDYGAQQLIRSSSNASTISIGSSGELQRQRVMEAVHFRVRHTITIPNRGAADEWADFGFDLPDCKSRKQSIKEEFTEGEIN

>NXS76145.1 P73 protein [Pandion haliaetus]

AKMSQSSPADEGTTFEHLWSTLEPDSTYFDLPPSNHTGSNEVSNRTEVTMDVFQMRSMNDSVMSQFNLLNNSMDQSIGSRAASTSPYSSEHTSNVPTHSPYSQPSSTFDAMSPAPVIPSNTDYPGPHHFEVTFQQSSTAKSATWTYSPLLKKLYCQIAKTCPIQIKVSTPPPPGTIIRAMPVYKKAEHVTEVVKRCPNHELGRDFNDGQSAPASHLIRVEGNNLSQYVDDPVTGRQSVMVPYEPPQVGTEFTTILYNFMCNSSCVGGMNRRPILIIITLETRDGQVLGRRSFEGRICACPGRDRKADEDHYREQQALNESAAKNGNANKRTFKQSPQGIPALGAGIKKRRHGEEEMYYVPVRGRENFEILMKIKESLELVELVPQQLVDSYRQQQQQLLQRQSQLQTPSSYGPVLSPMNKVHGGGINKLPSVNQLVGQPAQHSSSSAPSLGPMGPGMLNSHPMQPNGEMNGGHSSQSMVSGSHCTPPPPYNPDPSLISFLTGLGCPNCIDYFTSQGLQNIYHLQNLSIEDLGALKIPEQYRMIIWRGLQELKQSHDYGAQQLIRSSSNASTISIGSSGELQRQRVMEAVHFRVRHTITIPNRGGADEWADFGFDLPDCKSRKQSIKEEFTEGEIN

>XP_015503625.1 tumor protein p73 isoform X1 [Parus major]

MKDWRWIPLQKVIYPLSDSPTVKMSQSSPADEGTTFEHLWSTLEPDSTYFDLPPANPTGSNEVSNRTEVTMDVFQMRGMTDSVMSQFNLLNNSMDQSIGSRAASTSPYSSEHTSNVPTHSPYSQPSSTFDAMSPAPVIPSNTDYPGPHHFEVTFQQSSTAKSATWTYSPLLKKLYCQIAKTCPIQIKVSTSPPPGTIIRAMPVYKKAEHVTEVVKRCPNHELGRDFNDGQSAPASHLIRVEGNNLSQYVDDPVTGRQSVMVPYEPPQVGTEFTTILYNFMCNSSCVGGMNRRPILIIITLETRDGQVLGRRSFEGRICACPGRDRKADEDHFREQQALNESTAKNGNANKRTFKQSPQGIPALGTGIKKRRHGEEEMYYVPVRGRENFEILMKIKESLELVELVPQQLVDSYRQQQQQLLQRQSQLQTPSSYGPVLSPMNKVHGGGINKLPSVNQLVGQPAQHSSSSAPSLGPMGPGMLNSHPMQTNGEMNGGHSSQSMVSGSHCTPPPPYNPDPSLVSFLTGLGCPNCIDYFTSQGLQNIYHLQNLSIEDLGALKIPEQYRMIIWRGLQELKQSHDYGAQQLIRSSSNASTISIGSSGELQRQRVMEAVHFRVRHTITIPNRGAADDWADFGFDLPDCKSRKQSIKEEFTEGEIN

>XP_035200611.1 tumor protein p73 isoform X1 [Oxyura jamaicensis]

MESNMETGVIYPLSDSPSAKMSESSPADEGTTFEHLWSTLEPDSTYFDLPPSNHAGSSEVSNRTEVTMDVFQLRGMNDSVMSQFNLLNNSMDQSIGSRAASTSPYSSEHTSNVPTHSPYSQPSSTFDAMSPAPVIPSNTDYPGPHHFEVTFQQSSTAKSATWTVSCMSSSLPRRDACCSRLGFPPACCRHYSPLLKKLYCQIAKTCPIQIKVSTPPPPGTIIRAMPVYKKAEHVTEVVKRCPNHELGRDFNDGQSAPASHLIRVEGNNLSQYVDDPVTGRQSVMVPYEPPQVGTEFTTILYNFMCNSSCVGGMNRRPILIIITLEMRDGQVLGRRSFEGRICACPGRDRKADEDHYREQQALNESAAKNGNANKRTFKQSPQGIPALGAGIKKRRHGEEEMYYVPVRGRENFEILMKIKESLELVELVPQQLVDSYRQQQQQLLQRQSQLQTPSSYGPVLSPMNKVHGGGINKLPSVNQLVGQPPQHSSSSAPSLGPMGPGMLNSHPMQTNGEMNGGHSSQSMVSGSHCTPPPPYNPDPSLVSFLTGLGCPNCIDYFTSQGLQNIYHLQNLSIEDLGALKIPEQYRMIIWRGLQELKQSHDYGAQQLIRSSSNASTISIGSSGELQRQRVMEAVHFRVRHTITIPNRGGADEWADFGFDLPDCKSRKQSIKEEFTEGEIN

>XP_031469279.1 tumor protein p73 isoform X1 [Phasianus colchicus]

MLPIPRAMENSMEAGVIYPLSDSPSVKMSQSSPADESTTFEHLWSTLAPDSTYFDLSPSSHTGNNEVSNRTEITMDVFQMRGMNDSVMSQFNLLNNSMDQSIGSRAASTSPYSSEHTSNVPTHSPYSQPSSTFDAMSPAPVIPSNTDYPGPHHFEVTFQQSSTAKSATWTYSPLLKKLYCQIAKTCPIQIKVSSPPPPGTIIRAMPVYKKAEHVTEVVKRCPNHELGRDFNDGQSAPASHLIRVEGNNLSQYVDDPVTGRQSVMVPYEPPQVGTEFTTILYNFMCNSSCVGGMNRRPILIIITLETRDGQVLGRRSFEGRICACPGRDRKADEDHYREQQALNENAAKNGNANKRTFKQSPQAIPALGPGVKKRRHGEEEMYYVPVRGRENFEILMKIKESLELVELVPQQLVDSYRQQQQQLLQRQNQLQTPSSYGPVLSPMNKVHGGGINKLPSVNQLVGQPAQHSSGSASSLGPMGPGMLNSHPMQPNGEMNGGHSSQSMVSGSHCTPPPPYNPDPSLVSFLTGLGCPNCIDYFTSQGLQNIYHLQNLSIEDLGALKIPEQYRMIIWRGLQELKQSHDYGAQQLIRSSSNASTISIGSSGELQRQRVMEAVHFRVRHTITIPNRGAADEWADFGFDLPDCKSRKQSIKEEFTEGEIN

>XP_015738031.1 tumor protein p73 isoform X1 [Coturnix japonica]

MLLIPRAMESSVEAGVIYPLSDSPSVKMSQSSPADESTTFEHLWSTLAPDSTYFDLSPSSHTGSNEVSNSTEVSMDVFQMRGMNDSVMSQFNLLNNSMDQSIGSRAASTSPYSSEHTSNVPTHSPYSQPSSTFDAMSPAPVIPSNTDYPGPHHFEVTFQQSSTAKSATWTYSPLLKKLYCQIAKTCPIQIKVSSPPPPGTIIRAMPVYKKAEHVTEVVKRCPNHELGRDFNDGQSAPASHLIRVEGNNLSQYVDDPVTGRQSVMVPYEPPQVGTEFTTILYNFMCNSSCVGGMNRRPILIIITLETRDGQVLGRRSFEGRICACPGRDRKADEDHYREQQALNENAAKNGNANKRTFKQSPQAIPALGPGVKKRRHGEEEMYYVPVRGRENFEILMKIKESLELVELVPQQLVDSYRQQQQQLLQRQNQLQTPSSYGPVLSPMNKVHGGGINKLPSVNQLVGQPAQHSSSSAPSLGPMGPGMLNSHPMQPNGEMNGGHSSQSMVSGSHCTPPPPYNPDPSLVSFLTGLGCPNCIDYFTSQGLQNIYHLQNLSMEDLGALKIPEQYRMIIWRGLQELKQSHDYGAQQLIRSSSNASTISIGSSGELQRQRVMEAVHFRVRHTITIPNRGAADEWADFGFDLPDCKSRKQSIKEEFTEGEIN

>XP_003212229.2 tumor protein p73 isoform X1 [Meleagris gallopavo]

MLPIPRAMESSMEAGVIYPLSDSPSVKMSQSSPADESTTFEHLWSTLAPDSTYFDLSPSSHTGDNEVSNRTEITMDVFQMRGMNDSVMSQFNLLNNSMDQSIGSRAASTSPYSSEHTSNVPTHSPYSQPSSTFDAMSPAPVIPSNTDYPGPHHFEVTFQQSSTAKSATWTYSPLLKKLYCQIAKTCPIQIKVSSPPPPGTIIRAMPVYKKAEHVTEVVKRCPNHELGRDFNDGQSAPASHLIRVEGNNLSQYVDDPVTGRQSVMVPYEPPQVGTEFTTILYNFMCNSSCVGGMNRRPILIIITLETRDGQVLGRRSFEGRICACPGRDRKADEDHYREQQALNESAAKNGNANKRTFKQSPQAIPALGPGVKKRRHGEEEMYYVPVRGRENFEILMKIKESLELVELVPQQLVDSYRQQQQQLLQRQNQLQTPSSYGPVLSPMNKVHGGGINKLPSVNQLVGQPAQHSSGSASSLGPMGPGMLNSHPMQPNGEMNGGHSSQSMVSGSHCTPPPPYNPDPSLVSFLTGLGCPNCIDYFTSQGLQNIYHLQNLSIEDLGALKIPEQYRMIIWRGLQELKQSHDYGAQQLIRSSSNASTISIGSSGELQRQRVMEAVHFRVRHTITIPNRGAADEWADFGFDLPDCKSRKQSIKEEFTEGEIN

>NWI26228.1 P73 protein [Sula dactylatra]

VKMSQSSPADEGTTFEHLWSTLEPDSTYFDLPPSNHTGSNEVSNRTEVTMDVFQMRSMNDSVMSQFNLLNNSMDQSIGSRAASTSPYSSEHTSNVPTHSPYSQPSSTFDAMSPAPVIPSNTDYPGPHHFEVTFQQSSTAKSATWTYSPLLKKLYCQIAKTCPIQIKVSTPPPPGTIVRAMPVYKKAEHVTEVVKRCPNHELGRDFNDGQSAPASHLIRVEGNNLSQYVDDPVTGRQSVMVPYEPPQVGTEFTTILYNFMCNSSCVGGMNRRPILIIITLETRDGQVLGRRSFEGRICACPGRDRKADEDHYREQQALNESAAKNGNANKRTFKQSPQGIPALGASVKKRRHGEEEMYYVPVRGRENFEILMKIKESLELVELVPQQLVDSYRQQQQQLLQRQSQLQTPSSYGPVLSPMNKVHGGGINKLPSVNQLVGQPAQHSSGSAPSLGPMGPGMLNSHPMQPNGEMNGGHSSQSMVSGSHCTPPPPYNPDPSLVSFLTGLGCPNCIDYFTSQGLQNIYHLQNLSIEDLGALKIPEQYRMIIWRGLQELKQSHDYGAQQLIRSSSNASTISIGSSGELQRQRVMEAVHFRVRHTITIPNRGGADEWADFGFDLPDCKSRKQSIKEEFTEGEIN

>NXW24843.1 P73 protein [Circaetus pectoralis]

AKMSQSSPADEGTTFEHLWSTLEPDSTYFDLPPSNHTGSNEVSNRTEVTMDVFQMRSMNDSVMSQFNLLNNSMDQSIGSRAASTSPYSSEHTSNVPTHSPYSQPSSTFDAMSPAPVIPSNTDYPGPHHFEVTFQQSSTAKSATWTYSPLLKKLYCQIAKTCPIQIKVSTPPPPGTIIRAMPVYKKAEHVTEVVKRCPNHELGRDFNDGQSAPASHLIRVEGNNLSQYVDDPVTGRQSVMVPYEPPQVGTEFTTILYNFMCNSSCVGGMNRRPILIIITLETRDGQVLGRRSFEGRICACPGRDRKADEDHYREQQALNESAAKNGNANKRTFKQSPQGIPALGTGIKKRRHGEEEMYYVPVRGRENFEILMKIKESLELVELVPQQLVDSYRQQQQQLLQRQSQLQTPSSYGPVLSPMNKVHGGGLNKLPSVNQLVGQPAQHSTSSAPSLGPMGPGMLNSHPMQPNGEMNGGHSSQSMVSGSHCTPPPPYNPDPSLVSFLTGLGCPNCIDYFTSQGLQNIYHLQNLSIEDLGALKIPEQYRMIIWRGLQELKQSHDYGAQQLIRSSSNASTISIGSSGELQRQRVMEAVHFRVRHTITIPNRGGADEWADFGFDLPDCKSRKQSIKEEFTEGEIN

>NWH47050.1 P73 protein [Fregata magnificens]

VKMSQSSPADEGTTFEHLWSTLEPDSTYFDLPPSNHTGSNEVSNRTEVTMDVFQMRSMNDSVMSQFNLLNNSMDQSIGSRAASTSPYSSEHTSNVPTHSPYSQPSSTFDAMSPAPVIPSNTDYPGPHHFEVTFQQSSTAKSATWTYSPLLKKLYCQIAKTCPIQIKVSTPPPPGTIIRAMPVYKKAEHVTEVVKRCPNHELGRDFNDGQSAPASHLIRVEGNNLSQYVDDPVTGRQSVMVPYEPPQVGTEFTTILYNFMCNSSCVGGMNRRPILIIITLETRDGQVLGRRSFEGRICACPGRDRKADEDHYREQQALNESAAKNGNANKRTFKQSPQGIPALGAGIKKRRHGEEEMYYVPVRGRENFEILMKIKESLELVELVPQQLVDSYRQQQQQLLQRQSQLQTPSSYGPVLSPMNKVHSGGINKLPSVNQLVGQPAQHSSSSAPSLGPMGPGMLNSHPMQPNGEMNGGHSSQSMVSGSHCTPPPPYNPDPSLVSFLTGLGCPNCIDYFTSQGLQNIYHLQNLSIEDLGALKIPEQYRMIIWRGLQELKQSHDYGAQQLIRSSSNASTISIGSSGELQRQRVMEAVHFRVRHTITIPNRGGADEWADFGFDLPDCKSRKQSIKEEFTEGEIN

>NWZ58252.1 P73 protein [Haliaeetus albicilla]

AKMSQSSPADEGTTFEHLWSTLEPDSTYFDLPPSNHTGSNEVSNRTEVTMDVFQMRSMNDSVMSQFNLLNNSMDQSIGSRAASTSPYSSEHTSNVPTHSPYSQPSSTFDAMSPAPVIPSNTDYPGPHHFEVTFQQSSTAKSATWTYSPLLKKLYCQIAKTCPIQIKVSTPPPPGTIIRAMPVYKKAEHVTEVVKRCPNHELGRDFNDGQSAPASHLIRVEGNNLSQYVDDPVTGRQSVMVPYEPPQVGTEFTTILYNFMCNSSCVGGMNRRPILIIITLETRDGQVLGRRSFEGRICACPGRDRKADEDHYREQQALNESAAKNGNANKRTFKQSPQGIPALGTGIKKRRHGEEEMYYVPVRGRENFEILMKIKESLELVELVPQQLVDSYRQQQQQLLQRQSQLQTPSSYGPVLSPMNKVHGGGLNKLPSVNQLVGQPAQHSSGSAPSLGPMGPGMLNSHPMQPNGEMNGGHSSQSMVSGSHCTPPPPYNPDPSLVSFLTGLGCPNCIDYFTSQGLQNIYHLQNLSIEDLGALKIPEQYRMIIWRGLQELKQSHDYGAQQLIRSSSNASTISIGSSGELQRQRVMEAVHFRVRHTITIPNRGGADEWADFGFDLPDCKSRKQSIKEEFTEGEIN

>NXH70420.1 P73 protein [Oceanodroma tethys]

VKMSQSSPADEGTTFEHLWSTLEPDSTYFDLPPSNHTGSNEVSNRTEVTMDVFQMRSMNDSVMSQFNLLNNSMDQSIGSRAASTSPYSSEHTSNVPTHSPYSQPSSTFDAMSPAPVIPSNTDYPGPHHFEVTFQQSSTAKSATWTYSPLLKKLYCQIAKTCPIQIKVSTPPPPGTIIRAMPVYKKAEHVTEVVKRCPNHELGRDFNDGQSAPASHLIRVEGNNLSQYVDDPVTGRQSVMVPYEPPQVGTEFTTILYNFMCNSSCVGGMNRRPILIIITLETRDGQVLGRRSFEGRICACPGRDRKADEDHYREQQALNESAAKNGNANKRTFKQSPQGIPALGAGIKKRRHGEEEMYYVPVRGRENFEILMKIKESLELVELVPQQLVDSYRQQQQQLLQRQSQLQAPSSYGPVLSPMNKVHGGGINKLPSVNQLVGQPAQHGSGSAPSLGPMGPGMLNSHPMQPNGEMNGGHSSQSMVSGSHCTPPPPYNPDPSLVSFLTGLGCPNCIDYFTSQGLQNIYHLQNLSIEDLGALKIPEQYRMIIWRGLQELKQSHDYGAQQLIRSSSNASTISIGSSGELQRQRVMEAVHFRVRHTITIPNRGGADEWADFGFDLPDCKSRKQSIKEEFTEGEIN

>NXJ55541.1 P73 protein [Spizaetus tyrannus]

AKMSQSSPADEGTTFEHLWSTLEPDSTYFDLPPSNHTGSNEVSNRTEVTMDVFQMRSMNDSVMSQFNLLNNSMDQSIGSRAASTSPYSSEHTSNVPTHSPYSQPSSTFDAMSPAPVIPSNTDYPGPHHFEVTFQQSSTAKSATWTYSPLLKKLYCQIAKTCPIQIKVSTPPPPGTIIRAMPVYKKAEHVTEVVKRCPNHELGRDFNDGQSAPASHLIRVEGNNLSQYVDDPVTGRQSVMVPYEPPQVGTEFTTILYNFMCNSSCVGGMNRRPILIIITLETRDGQVLGRRSFEGRICACPGRDRKADEDHYREQQALNESAAKNGNANKRTFKQSPQGIPALGTGIKKRRHGEEEMYYVPVRGRENFEILMKIKESLELVELVPQQLVDSYRQQQQQLLQRQSQLQTPSSYGPVLSPMNKVHGGGLNKLPSVNQLVGQPVQHSSGSAPSLGPMGPGMLNSHPMQPNGEMNGGHSSQSMVSGSHCTPPPPYNPDPSLVSFLTGLGCPNCIDYFTSQGLQNIYHLQNLSIEDLGALKIPEQYRMIIWRGLQELKQSHDYGAQQLIRSSSNASTISIGSSGELQRQRVMEAVHFRVRHTITIPNRGGADEWADFGFDLPDCKSRKQSIKEEFTEGEIN

>XP_010404147.1 tumor protein p73 isoform X3 [Corvus cornix cornix]

MEEGVIYPLSDSPALKMSQSSAADEGTTFEHLWSTLEPDSTYFDLPPANPTGSNEVSNRTEVTMDVFQMRGMTDSVMSQFNLLNNSMDQSIGSRAASTSPYSSEHTSNVPTHSPYSQPSSTFDAMSPAPVIPSNTDYPGPHHFEVTFQQSSTAKSATWTYSPLLKKLYCQIAKTCPIQIKVSTSPPPGTIIRAMPVYKKAEHVTEVVKRCPNHELGRDFNDGQSAPASHLIRVEGNNLSQYVDDPVTGRQSVMVPYEPPQVGTEFTTILYNFMCNSSCVGGMNRRPILIIITLETRDGQVLGRRSFEGRICACPGRDRKADEDHFREQQALNESAAKNGNANKRTFKQSPQGIPALGTGIKKRRHGEEEMYYVPVRGRENFEILMKIKESLELVDLVPQQLVDSYRQQQQQLLQRQSQLQTPSSYGPVLSPMNKVHGGGINKLPSVNQLVGQPAQHSSGSAPSLGPMGPGMLNSHPMQPNGEMNGGHSSQSMVSGSHCTPPPPYNPDPSLVSFLTGLGCPNCIDYFTSQGLQNIYHLQNLSIEDLGALKIPEQYRMIIWRGLQELKQSHDYGAQQLIRSSSNASTISIGSSGELQRQRVMEAVHFRVRHTITIPNRGAADDWADFGFDLPDCKSRKQSIKEEFTEGEIN

>NXL42405.1 P73 protein [Podilymbus podiceps]

VKMSQSSPADEGTTFEHLWSTLEPDSTYFDLPPSNHTGSNEDSNRTEVTMDVFQMRNMNDSVMSQFNLLNNSMDQSIGSRAASTSPYSSEHTSNVPTHSPYSQPSSTFDAMSPAPVIPSNTDYPGPHHFEVTFQQSSTAKSATWTYSPLLKKLYCQIAKTCPIQIKVSTPPPPGTIIRAMPVYKKAEHVTEVVKRCPNHELGRDFNDGQSAPASHLIRVEGNNLSQYVDDPVTGRQSVMVPYEPPQVGTEFTTILYNFMCNSSCVGGMNRRPILIIITLETRDGQVLGRRSFEGRICACPGRDRKADEDHYREQQALNESAAKNGNANKRTFKQSPQGIPALGAGVKKRRHGEEEMYYVPVRGRENFEILMKIKESLELVELVPQQLVDSYRQQQQQLLQRQSQLQTPSSYGPVLSPMNKVHGGGINKLPPVNQLVGQPAQHSSSSAPSLGPMGPGMLNSHPMQPNGEMNGGHASQSMVSGSHCTPPPPYNPDPSLVSFLTGLGCPNCIDYFTSQGLQNIYHLQNLSIEDLGALKIPEQYRMIIWRGLQELKQSHDYGAQQLIRSSSNASTISIGSSGELQRQRVMEAVHFRVRHTITIPNRGGADEWADFGFDLPDCKSRKQSIKEEFTEGEIN

>NXW53052.1 P73 protein [Eurystomus gularis]

VKMSQASPADEGTTFEHLWSTLEPDSTYFDLPPSNHTGGNEVSNRTEVTMDVFQMRSMNDSVMSQFNLLNNSMDQSIGSRAASTSPYSSEHTSNVPTHSPYSQPSSTFDAMSPAPVIPSNTDYPGPHHFEVTFQQSSTAKSATWTYSPLLKKLYCQIAKTCPIQIKVSTPPPPGTIIRAMPVYKKAEHVTEVVKRCPNHELGRDFNDGQSAPASHLIRVEGNNLSQYVDDPVTGRQSVMVPYEPPQVGTEFTTILYNFMCNSSCVGGMNRRPILIIITLETRDGQVLGRRSFEGRICACPGRDRKADEDHYREQQALNESAAKNGNANKRTFKQSPQGIPALGTGVKKRRHGEEEMYYVPVRGRENFEILMKIKESLELVELVPQQLVDSYRQQQQQLLQRQSQLQTPSSYGPVLSPMNKVHGGGINKLPSVNQLVGQPAQHSSSSAPSLGPMGPGMLNSHPMQPNGEMNGGHSSQSMVSGSHCTPPPPYNPDPSAVSFLTGLGCPNCIDYFTSQGLQNIYHLQNLSIEDLGALKIPEQYRMIIWRGLQELKQSHDYGAQQLIRSSSNASTISIGSSGELQRQRVMEAVHFRVRHTITIPNRGGADDWADFGFDLPDCKSRKQSIKEEFTEGEIN

>NXI96062.1 P73 protein [Psophia crepitans]

KMSQSSPADEGTTFEHLWSTLEPDSTYFDLPPSNHPGSNEVSNRTEVTMDVFQMRSMNDSVMSQFNLLNNSMDQSIGSRAASTSPYSSEHTSNVPTHSPYSQPSSTFDAMSPAPVIPSNTDYPGPHHFEVTFQQSSTAKSATWTYSPLLKKLYCQIAKTCPIQIKVSTPPPPGTIIRAMPVYKKAEHVTEVVKRCPNHELGRDFNDGQAAPASHLIRVEGNNLSQYVDDPVTGRQSVMVPYEPPQVGTEFTTILYNFMCNSSCVGGMNRRPILIIITLETRDGQVLGRRSFEGRICACPGRDRKADEDHYREQQALNESAAKNGNANKRTFKQSPQGIPALGAGVKKRRHGEEEMYYVPVRGRENFEILMKIKESLELMELVPQQLVDSYRQQQQQLLQRQSQLQPPSSYGPVLSPMNKVHGAGINKLPSVNQLVGQPAQHSSGSAPGLGPMGPGMLNSHPMQANGEMNGGHSSQSMVSGSHCTPPPPYNPDPSLVSFLTGLGCPNCIDYFTSQGLQNIYHLQNLSIEDLGALKIPEQYRMIIWRGLQELKQSHDYGAQQLIRSSSNASTISIGSSGELQRQRVMEAVHFRVRHTITIPNRGGADEWADFGFDLPDCKSRKQSIKEEFTEGEIN

>NXI72005.1 P73 protein [Anseranas semipalmata]

VKMSQSSPADEGTTFEHLWSTLEPDSTYFDLPPSNHTGSNEVSNRTEVTMDVFQMRGMNDSVMSQFNLLNNSMDQSIGSRAASTSPYNSEHTSNVPTHSPYSQPSSTFDAMSPAPVIPSNTDYPGPHHFEVTFQQSSTAKSATWTYSPLLKKLYCQIAKTCPIQIKVSTPPPPGTIIRAMPVYKKAEHVTEVVKRCPNHELGRDFNDGQSAPASHLIRVEGNNLSQYVDDPVTGRQSVMVPYEPPQVGTEFTTILYNFMCNSSCVGGMNRRPILIIITLEMRDGQVLGRRSFEGRICACPGRDRKADEDHYREQQALNESAAKNGNANKRTFKQSPQGIPALGTGIKKRRHGEEEMYYVPVRGRENFEILMKIKESLELVELVPQQLVDSYRQQQQQLLQRQSQLQTPSSYGPVLSPMNKVHGGGINKLPSVNQLVPGQPPQHSSSSAPSLGPMGPGMLNSHPMQPNGEMNGGHSSQSMVSGSHCTPPPPYNPDPSLVSFLTGLGCPNCIDYFTSQGLQNIYHLQNLSIEDLGALKIPEQYRMIIWRGLQELKQSHDYGAQQLIRSSSNASTISIGSSGELQRQRVMEAVHFRVRHTITIPNRGGADEWADFGFDLPDCKSRKQSIKEEFTEGEIN

**>XP_040507202.1 tumor protein p73 isoform X1 [Gallus gallus]**

MLPIPRGMESSMEEGVIYPLSDSPSVKMSQSSPADESTTFEHLWSTLAPDSTYFDLSPSSHTGSNEVSNRTEVTMDVFQMRGMNDSVMSQFNLLNNSMDQSIGSRAASTSPYSSEHTSNVPTHSPYSQPSSTFDAMSPAPVIPSNTDYPGPHHFEVTFQQSSTAKSATWTYSPLLKKLYCQIAKTCPIQIKVSSPPPPGTIIRAMPVYKKAEHVTEVVKRCPNHELGRDFNDGQSAPASHLIRVEGNNLSQYVDDPVTGRQSVMVPYEPPQVGTEFTTILYNFMCNSSCVGGMNRRPILIIITLETRDGQVLGRRSFEGRICACPGRDRKADEDHYREQQALNENAAKNGNANKRTFKQSPQAIPALGPGVKKRRHGEEEMYYVPVRGRENFEILMKIKESLELVELVPQQLVDSYRQQQQQLLQRQNQLQTPSSYGPVLSPMNKAHGGGINKLPSVNQLVGQPAQHSSGSAPSLGPMGPGMLNSHPMQPNGEMNGGHSSQSMVSGSHCTPPPPYNPDPSLVSFLTGLGCPNCIDYFTSQGLQNIYHLQNLSIEDLGALKIPEQYRMIIWRGLQELKQSHDYGAQQLIRSSSNASTISIGSSGELQRQRVMEAVHFRVRHTITIPNRGAADEWADFGFDLPDCKSRKQSIKEEFTEGEIN

>NXJ03546.1 P73 protein [Odontophorus gujanensis]

VKMSQSSPADESTTFEHLWSTLAPDSTYFDLSPSSHTGSNEVSNRTEVTMDVFQMRGMNDSVMSQFNLLNNSMDQSIGSRAASTSPYSSEHTSNVPTHSPYSQPSSTFDAMSPAPVIPSNTDYPGPHHFEVTFQQSSTAKSATWTYSPLLKKLYCQIAKTCPIQIKVSSPPPPGTIIRAMPVYKKAEHVTEVVKRCPNHELGRDFNDGQSAPASHLIRVEGNNLSQYVDDPVTGRQSVMVPYEPPQVGTEFTTILYNFMCNSSCVGGMNRRPILIIITLETRDGQVLGRRSFEGRICACPGRDRKADEDHYREQQALNENAAKNGNANKRTFKQSPQAIPALGPGIKKRRHGEEEMYYVPVRGRENFEILMKIKESLELVELVPQQLVDSYRQQQQQLLQRQNQLQTPSSYGPVLSPMNKVHGGGINKLPSVNQLVGQPAQHSSSSAPSLGPMGPGMLNNHPMQPNGEMNGGHSSQSMVSGSHCTPPPPYNPDPSLVSFLTGLGCPNCIDYFTSQGLQNIYHLQNLSIEDLGALKIPEQYRMIIWRGLQELKQSHDYGAQQLIRSSSNASTISIGSSGELQRQRVMEAVHFRVRHTITIPNRGAADEWADFGFDLPDCKSRKQSIKEEFTEGEIN

>NXC47377.1 P73 protein [Penelope pileata]

VKMSQSSPADEGTTFEHLWSTLAPDSTYFDLPPSNHTGSNEVSNRTEITMDVFQMRGMNDSVMSQFNLLNNSMDQSIGSRAASTSPYSSEHTSNVPTHSPYSQPSSTFDAMSPAPVIPSNTDYPGPHHFEVTFQQSSTAKSATWTYSPLLKKLYCQIAKTCPIQIKVSSPPPPGTIIRAMPVYKKAEHVTEVVKRCPNHELGRDFNDGQSAPASHLIRVEGNNLSQYVDDPVTGRQSVMVPYEPPQVGTEFTTILYNFMCNSSCVGGMNRRPILIIITLETRDGQVLGRRSFEGRICACPGRDRKADEDHYREQQALNENAAKNGNANKRTFKQSPQAIPALGPGVKKRRHGEEEMYYVPVRGRENFEILMKIKESLELVELVPQQLVDSYRQQQQQLLQRQNQLQTPSSYGPVLSPMNKVHGGGINKLPSVNQLVGQPAQHSSSSAPSLGPMGPGMLNSHPMQPNGEMNGGHSSQSMVSGSHCTPPPPYNPDPSLVSFLTGLGCPNCIDYFTSQGLQNIYHLQNLSIEDLGALKIPEQYRMIIWRGLQELKQSHDYGAQQLIRSSSNASTISIGSSGELQRQRVMEAVHFRVRHTITIPNRGGADEWADFGFDLPDCKSRKQSIKEEFTEGEIN

>NXS50070.1 P73 protein [Balaeniceps rex]

VKMSQSSPADEGTTFEHLWSTLEPDSTYFDLSPSDHTGSNEVSNRTEVTMDVFQMRSMNDSVMSQFNLLNNSMDQSIGSRAASTSPYSSEHTSNVPTHSPYSQPSSTFDAMSPAPVIPSNTDYPGPHHFEVTFQQSSTAKSATWTYSPLLKKLYCQIAKTCPIQIKVSTPPPPGTIIRAMPVYKKAEHVTEVVKRCPNHELGRDFNDGQSAPASHLIRVEGNNLSQYVDDPVTGRQSVMVPYEPPQVGTEFTTILYNFMCNSSCVGGMNRRPILIIITLETRDGQVLGRRSFEGRICACPGRDRKADEDHYREQQALNESAAKNGNANKRTFKQSPQGIPALGTGIKKRRHGEEEMYYVPVRGRENFEILMKIKESLELVELVPQQLVDSYRQQQQQLLQRQSQLQTPSSYGPVLSPMNKVHGGGINKLPSVNQLVGQPAQHSSGPAPSLGPMGPGMLNSHPMQPNGEMNGGHSSQSMVSGSHCTPPPPYNPDPSLVSFLTGLGCPNCIDYFTSQGLQNIYHLQNLSIEDLGALKIPEQYRMIIWRGLQELKQSHDYGAQQLIRSSSNASTISIGSSGELQRQRVMEAVHFRVRHTITIPNRGGADEWADFGFDLPDCKSRKQSIKEEFTEGEIN

>NXX58906.1 P73 protein [Scopus umbretta]

VKMSQSSPADEGTTFEHLWSTLEPDSTYFDLSPSDHTGSNEVSNRTEVTMDVFQMRSMNDSVMSQFNLLNNSMDQSIGSRAASTSPYSSEHTSNVPTHSPYSQPSSTFDAMSPAPVIPSNTDYPGPHHFEVTFQQSSTAKSATWTYSPLLKKLYCQIAKTCPIQIKVSTPPPPGTIIRAMPVYKKAEHVTEVVKRCPNHELGRDFNDGQSAPASHLIRVEGNNLSQYVDDPVTGRQSVMVPYEPPQVGTEFTTILYNFMCNSSCVGGMNRRPILIIITLETRDGQVLGRRSFEGRICACPGRDRKADEDHYREQQALNESAAKNGNANKRTFKQSPQGIPALGTGVKKRRHGDEETYYVPVRGRENFEILMKIKESLELVELVPQQLVDSYRQQQQQLLQRQSQLQTPSSYGPVLSPMNKVHGGGINKLPSVNQLVGQPAQHSSGPAPSLGPMGPGMLNSHPMQPNGEMNGGHSSQSMVSGSHCTPPPPYNPDPSLVSFLTGLGCPNCIDYFTSQGLQNIYHLQNLSIEDLGALKIPEQYRMIIWRGLQELKQSHDYGAQQLIRSSSNASTISIGSSGELQRQRVMEAVHFRVRHTITIPNRGGADEWADFGFDLPDCKSRKQSIKEEFTEGEIN

>NXK05787.1 P73 protein [Herpetotheres cachinnans]

VKMSQSSPADEGTTFEHLWSTLEPDSTYFDLPPSNHTGSNEVSNRTEVTMDVFQMRSMNDSVMSQFNLLNNSMDQSIGSRAASTSPYSSEHTSNVPTHSPYSQPSSTFDAMSPAPVIPSNTDYPGPHHFEVTFQQSSTAKSATWTYSPLLKKLYCQIAKTCPIQIKVSTPPPPGTIIRAMPVYKKAEHVTEVVKRCPNHELGRDFNDGQSAPASHLIRVEGNNLSQYVDDPVTGRQSVMVPYEPPQVGTEFTTILYNFMCNSSCVGGMNRRPILIIITLETRDGQVLGRRSFEGRICACPGRDRKADEDHYREQQALNESAAKNGTANKRTFKQSPQGIPALSTGVKKRRHGEEEMYYVPVRGRENFEILMKIKESLELVELVPQQLVDSYRQQQQQLLQRQSQLQTPSSYGPVLSPMNKVHSGGINKLPSVNQLVGQPAQHSSGSAPSLGPMGPGMLNSHPMQPNGEMNGGHSSQSMVSGSHCTPPPPYNPDPSLVSFLTGLGCPNCIDYFTSQGLQNIYHLQNLSIEDLGALKIPEQYRMIIWRGLQELKQSHDYGAQQLIRSSSNASTISIGSSGELQRQRVMEAVHFRVRHTITIPNRGGADEWADFGFDLPDCKSRKQSIKEEFTEGEIN

>NXN65651.1 P73 protein [Himantopus himantopus]

VKMSQSSPADEGTTFEHLWSTLEPDSTYFDLPPSNHTGSNEVSNRTEVTMDVFQMRSMNDSVMSQFNLLNNSMDQSIGSRAASTSPYSSEHTSNVPTHSPYSQPSSTFDAMSPAPVIPSNTDYPGPHHFEVTFQQSSTAKSATWTYSPLLKKLYCQIAKTCPIQIKVSTPPPPGTIIRAMPVYKKAEHVTEVVKRCPNHELGRDFNDGQSAPASHLIRVEGNNLSQYVDDPVTGRQSVMVPYEPPQVGTEFTTILYNFMCNSSCVGGMNRRPILIIITLETRDGQVLGRRSFEGRICACPGRDRKADEDHYREQQALNESAAKNGNGNKRTFKQSPQSIPALGAGIKKRRHGEEEMYYVPVRGRENFEILMKIKESLELVELVPQQLVDSYRQQQQQLLQRQTQLQTPSSYGPVLSPMNKVHGGGINKLPSVNQLVGQPAQHGSSSAPSLGPMGPGMLNSHPMQPNGEMNGGHSSQSMVSGSHCTPPPPYNPDPSLVSFLTGLGCPNCIDYFTSQGLQNIYHLQNLSIEDLGALKIPEQYRMIIWRGLQELKQSHDYGAQQLIRSSSNASTISIGSSGELQRQRVMEAVHFRVRHTITIPNRGGADEWADFGFDLPDCKSRKQSIKEEFTEGEIN

>NXA18734.1 P73 protein [Ibidorhyncha struthersii]

VKMSQSSPADEGTTFEHLWSTLEPDSTYFDLPPANHTGSNEVSNRTEVTMDVFQMRSMNDSVMSQFNLLNNSMDQSIGSRAASTSPYSSEHTSNVPTHSPYSQPSSTFDAMSPAPVIPSNTDYPGPHHFEVTFQQSSTAKSATWTYSPLLKKLYCQIAKTCPIQIKVSTPPPPGTIIRAMPVYKKAEHVTEVVKRCPNHELGRDFNDGQSAPASHLIRVEGNNLSQYVDDPVTGRQSVMVPYEPPQVGTEFTTILYNFMCNSSCVGGMNRRPILIIITLETRDGQVLGRRSFEGRICACPGRDRKADEDHYREQQALNESAAKNGNGNKRTFKQSPQSIPALGAGIKKRRHGEEEMYYVPVRGRENFEILMKIKESLELVELVPQQLVDSYRQQQQQLLQRQTQLQTPSSYGPVLSPMNKVHGGGINKLPSVNQLVGQPAQHGSGSAPSLGAMGPGMLNSHPMQPNGEMNGGHSSQSMVSGSHCTPPPPYNPDPSLVSFLTGLGCPNCIDYFTSQGLQNIYHLQNLSIEDLGALKIPEQYRMIIWRGLQELKQSHDYGAQQLIRSSSNASTISIGSSGELQRQRVMEAVHFRVRHTITIPNRGGADEWADFGFDLPDCKSRKQSIKEEFTEGEIN

>NWS69547.1 P73 protein [Crotophaga sulcirostris]

VKMSQSSPADEGATFEHLWSTLEPDSTYFDLPPSNHTGSNEVSNRTEVTMDVFQMRSMNDSVMSQFNLLNNSMDQSIGSRAASTSPYSSEHTSNVPTHSPYSQPSSTFDAMSPAPVIPSNTDYPGPHHFEVTFQQSSTAKSATWTYSPLLKKLYCQIAKTCPIQIKVSTPPPPGTIIRAMPVYKKAEHVTEVVKRCPNHELGRDFNDGQSAPASHLIRVEGNNLSQYVDDPVTGRQSVMVPYEPPQVGTEFTTILYNFMCNSSCVGGMNRRPILIIITLETRDGQVLGRRSFEGRICACPGRDRKADEDHYREQQALNESAAKNGNANKRTFKQSPQGIPALGAGIKKRRHGEEEMFYVPVRGRENFEILMKIKESLELVELVPQQLVDSYRQQQQQLLQRQSQLQTPSSYGPVLSPMNKVHSGGINKLPSVNQLVGQPAQHGSGSAPSLGPMGPGMLNSHPMQPNGEMNGGHSSQSMVSGSHCTPPPPYNPDPSLVSFLTGLGCPNCIDYFTSQGLQNIYHLQNLSIEDLGALKIPEQYRMIIWRGLQELKQSHDYGAQQLIRSSSNASTISIGSSGELQRQRVMEAVHFRVRHTITIPNRGGADEWADFGFDLPDCKSRKQSIKEEFTEGEIN

>NWQ91032.1 P73 protein [Burhinus bistriatus]

VKMSQSSPADEGTTFEHLWSTLEPDSTYFDLPPSNHTGSNEVSNRTEVTMDVFQMRSMNDSVMSQFNLLNNSMDQSIGSRAASTSPYSSEHTSNVPTHSPYSQPSSTFDAMSPAPVIPSNTDYPGPHHFEVTFQQSSTAKSATWTYSPLLKKLYCQIAKTCPIQIKVSTPPPPGTIIRAMPVYKKAEHVTEVVKRCPNHELGRDFNDGQSAPASHLIRVEGNNLSQYVDDPVTGRQSVMVPYEPPQVGTEFTTILYNFMCNSSCVGGMNRRPILIIITLETRDGQVLGRRSFEGRICACPGRDRKADEDHYREQQALNESAAKNGNANKRTFKQSPQSIPALGASIKKRRHGEEEMYYVPVRGRENFEILMKIKESLELVELVPQQLVDSYRQQQQQLLQRQTQLQTPSSYGPVLSPMNKVHGGGINKLPSVNQLVGQPAQHGSSSAPSLGPMGAGMLNSHPMQPNGEMNGGHSSQSMVSGSHCTPPPPYNPDPSLVSFLTGLGCPNCIDYFTSQGLQNIYHLQNLSIEDLGALKIPEQYRMIIWRGLQELKQSHDYGAQQLIRSSSNASTISIGSSGELQRQRVMEAVHFRVRHTITIPNRGGADEWADFGFDLPDCKSRKQSIKEEFTEGEIN

>NWR85231.1 P73 protein [Furnarius figulus]

KMSQSSPADEGNTFEHLWSTLEPDSTYFDLSPATHTGSNEVSNRTEVTMDVFQMRGMNDSVMSQFNLLNNSMDQSIGSRAASTSPYSSEHTSNVPTHSPYSQPSSTFDAMSPAPVIPSNTDYPGPHHFEVTFQQSSTAKSATWTYSPLLKKLYCQIAKTCPIQIKVSTPPPPGTIIRAMPVYKKAEHVTEVVKRCPNHELGRDFNDGQSAPASHLIRVEGNNLSQYVDDPVTGRQSVMVPYEPPQVGTEFTTILYNFMCNSSCVGGMNRRPILIIITLETRDGQVLGRRSFEGRICACPGRDRKADEDHFREQQALNESAAKNGNANKRTFKQSPQSIPALGTSIKKRRHGEEEMYYVPVRGRENFEILMKIKESLELVELVPQQLVDSYRQQQQQLLQRQSQLQTPSSYGPVLSPMNKVHSGGINKLPSVNQLVGQPAQHGPGSAPSLGPMGPGMLNSHPMQPNGEMNGGHSSQSMVSGSHCTPPPPYNPDPSLVSFLTGLGCPNCIDYFTSQGLQNIYHLQNLSIEDLGALKIPEQYRMIIWRGLQELKQSHDYGAQQLIRSSSNASTISIGSSGELQRQRVMEAVHFRVRHTITIPNRGAADDWADFGFDLPDCKSRKQSIKEEFTEGEIN

>NXN55151.1 P73 protein [Rynchops niger]

VKMSQSSPADEGTTFEHLWSTLEPDSTYFDLPPSNHTGSNEVSNRTEVTMDVFQMRSMNDSVMSQFNLLNNSMDQSIGSRAASTSPYSSEHTSNVPTHSPYSQPSSTFDAMSPAPVIPSNTDYPGPHHFEVTFQQSSTAKSATWTYSPLLKKLYCQIAKTCPIQIKVSTPPPPGTIIRAMPVYKKAEHVTEVVKRCPNHELGRDFNDGQSAPASHLIRVEGNNLSQYVDDPVTGRQSVMVPYEPPQVGTEFTTILYNFMCNSSCVGGMNRRPILIIITLETRDGQVLGRRSFEGRICACPGRDRKADEDHYREQQALNESAAKNGNANKRTFKQSPQGIPALGTGIKKRRHGEEEMYYVPVRGRENFEILMKIKESLELVELVPQQLVDSYRQQQQQLLQRQTQLQTPSSYGPVLSPINKVHGGGINKLPSVNQLVGQPAQHGSGSAPGLGPMGPGMLNSHPMQPNGEMNGGHSSQPMVSGSHCTPPPPYNPDPSLVSFLTGLGCPNCIDYFTSQGLQNIYHLQNLSIEDLGALKIPEQYRMIIWRGLQELKQSHDYGAQQLIRSSSNASTISIGSSGELQRQRVMEAVHFRVRHTITIPNRGGADEWADFGFDLPDCKSRKQSIKEEFTEGEIN

>NXY15437.1 P73 protein [Atrichornis clamosus]

VKMSQSSPADEGTTFEHLWSTLEPDSTYFDLSPANHTGSNEVPNRTEVTMDVFQMRGMNDSVMSQFNLLNNSMDQSIGSRAASTSPYSSEHTSNVPTHSPYSQPSSTFDAMSPAPVIPSNTDYPGPHHFEVTFQQSSTAKSATWTYSPLLKKLYCQIAKTCPIQIKVSTPPPPGTIIRAMPVYKKAEHVTEVVKRCPNHELGRDFNDGQSAPASHLIRVEGNNLSQYVDDPVTGRQSVMVPYEPPQVGTEFTTILYNFMCNSSCVGGMNRRPILIIITLETRDGQVLGRRSFEGRICACPGRDRKADEDHFREQQALNESAAKNGNANKRTFKQSPQGIPALGTGIKKRRHGEEEMYYVPVRGRENFEILMKIKESLELVELVPQQLVDSYRQQQQQLLQRQSQLQTPSSYGPVLSPMNKVHGGGINKLPSVNQLVGQPAQHSSGSTPSLGPMGPGMLNSHPMQPNGEMNGGHSSQSMVSGSHCTPPPPYNPDPSLVSFLTGLGCPNCIDYFTSQGLQNIYHLQNLSIEDLGALKIPEQYRMIIWRGLQELKQSHDYGAQQLIRSSSNASTISIGSSGELQRQRVMEAVHFRVRHTITIPNRGADDWADFGFDLPDCKSRKQSIKEEFTEGEIN

>NWT37728.1 P73 protein [Chroicocephalus maculipennis]

VKMSQSSPADEGTTFEHLWSTLEPDSTYFDLPPSNHTGSNEVSDRTEVTMDVFQMRSMNDSVMSQFNLLNNSMDQSIGSRAASTSPYSSEHTSNVPTHSPYSQPSSTFDAMSPAPVIPSNTDYPGPHHFEVTFQQSSTAKSATWTYSPLLKKLYCQIAKTCPIQIKVSTPPPPGTIIRAMPVYKKAEHVTEVVKRCPNHELGRDFNDGQSAPASHLIRVEGNNLSQYVDDPVTGRQSVMVPYEPPQVGTEFTTILYNFMCNSSCVGGMNRRPILIIITLETRDGQVLGRRSFEGRICACPGRDRKADEDHYREQQALNESAAKNGNANKRTFKQSPQGIPALGTGIKKRRHGEEEMYYVPVRGRENFEILMKIKESLELVELVPQQLVDSYRQQQQQLLQRQTQLQTPSSYGPVLSPMNKVHGGGINKLPSVNQLVGQPAQHGSGSAPGLGPMGPGMLNSHPMQPNGEMNGGHSSQPMVSGSHCTPPPPYNPDPSLVSFLTGLGCPNCIDYFTSQGLQNIYHLQNLSIEDLGALKIPEQYRMIIWRGLQELKQSHDYGAQQLIRSSSNASTISIGSSGELQRQRVMEAVHFRVRHTITIPNRGGADEWADFGFDLPDCKSRKQSIKEEFTEGEIN

>NXN42389.1 P73 protein [Rhinoptilus africanus]

VKMSQSSPADEGTTFEHLWSTLEPDSTYFDLPPSNHTGSNEVSNRTEVTMDVFQMRSMNDSVMSQFNLLNNSMDQSIGSRAASTSPYSSEHTSNVPTHSPYSQPSSTFDAMSPAPVIPSNTDYPGPHHFEVTFQQSSTAKSATWTYSPLLKKLYCQIAKTCPIQIKVSTPPPPGTVIRAMPVYKKAEHVTEVVKRCPNHELGRDFNDGQAAPASHLIRVEGNNLSQYVDDPVTGRQSVMVPYEPPQVGTEFTTILYNFMCNSSCVGGMNRRPILIIITLETRDGQVLGRRSFEGRICACPGRDRKADEDHYREQQALNESAAKNGNANKRTFKQSSQGIPALGTGVKKRRHGEEEMYYVPVRGRENFEILMKIKESLELVELVPQQLVDSYRQQQQQLLQRQTQLQTPSSYGPVLSPMNKVHGGGINKLPSVNQLVGQPAQHGSGSAPSLGPMGPGMLNSHPMQPNGEMNGGHSSQPMVSGSHCTPPPPYNPDPSLVSFLTGLGCPNCIEYFTSQGLQNIYHLQNLSIEDLGALKIPEQYRMIIWRGLQELKQSHDYGAQQLIRSSSNASTISIGSSGELQRQRVMEAVHFRVRHTITIPNRGGADEWADFGFDLPDCKSRKQSIKEEFTEGEIN

>NXT74698.1 P73 protein [Zapornia atra]

KMSQSSPADEGTTFEHLWSTLEPDSTYFDLPPSSHTGSNEVSNRTEVTMDVFQMRSMNDSVMSQFNLLNNSMDQSIGSRAASTSPYSSEHTSNVPTHSPYSQPSSTFDAMSPAPVIPSNTDYPGPHHFEVTFQQSSTAKSATWTYSPLLKKLYCQIAKTCPIQIKVSTPPPPGTIIRAMPVYKKAEHVTEVVKRCPNHELGRDFNDGQSAPASHLIRVEGNNLSQYVDDPVTGRQSVMVPYEPPQVGTEFTTILYNFMCNSSCVGGMNRRPILIIITLETRDGQVLGRRSFEGRICACPGRDRKADEDHYREQQALNESAAKNGTASKRTFKQSPQGIPALGTGVKKRRHGEEEMYYVPVRGRDNFEFLMKIKESLELMELVPQQLVDSYRQQQQQLLQRQSQLQTPSSYGPVLSPLNKVHGGGINKLPSVNQLVGQPAQHGSGSAPSLGPMGPGMLNTHPMQPNGEMNGGHSSQSMVSGSHCTPPPPYNPDPSLVSFLTGLGCPNCIDYFTSQGLQNIYHLQNLSIEDLGALKIPEQYRMIIWRGLQELKQSHDYGAQQLIRSSSNASTISIGSSGELQRQRVMEAVHFRVRHTITIPNRGAADEWADFGFDLPDCKSRKQSIKEEFTEGEIN

>NXC27586.1 P73 protein [Campylorhamphus procurvoides]

KMSQSSPADEGNTFEHLWSTLEPDSTYFDLSPATHTGSNEVSNRTEVTMDVFQMRGMNDSVMSQFNLLNNSMDQSIGSRAASTSPYSSEHTSNVPTHSPYSQPSSTFDAMSPAPVIPSNTDYPGPHHFEVTFQQSSTAKSATWTYSPLLKKLYCQIAKTCPIQIKVSTPPPPGTIIRAMPVYKKAEHVTEVVKRCPNHELGRDFNDGQSAPASHLIRVEGNNLSQYVDDPVTGRQSVMVPYEPPQVGTEFTTILYNFMCNSSCVGGMNRRPILIIITLETRDGQVLGRRSFEGRICACPGRDRKADEDHFREQQALNESAAKNGNANKRTFKQSPQSIPALGTSIKKRRHGEEEMYYVPVRGRENFEILMKIKESLELVELVPQQLVDSYRQQQQQLLQRQSQLQTPSSYGPVLSPMNKVHSGGINKLPSVNQLVGQPAQHGPGSAPSLGPMGPGMLNSHPMQPNGEMNGGHSSQSMVSGSHCTPPPPYNPDPSLVSFLTGLGCPNCIDYFTSQGLQNIYHLQNLSIEDLGALKIPEQYRMIIWRGLQELKQSHDYGAQQLIRSSSNASTISIGSSGELQRQRVMEAVHFRVRHTITIPNRGTADDWADFGFDLPDCKSRKQSIKEEFTEGEIN

>NXV70734.1 P73 protein [Atlantisia rogersi]

VKMSQSSPADEGTTFEHLWSTLEPDSTYFDLPPSSHTGSNEVSNRTEVTMDVFQMRSMNDSVMSQFNLLNSSMDQSIGSRAASTSPYSSEHTSNVPTHSPYSQPSSTFDAMSPAPVIPSNTDYPGPHHFEVTFQQSSTAKSATWTYSPLLKKLYCQIAKTCPIQIKVSTPPPPGTIIRAMPVYKKAEHVTEVVKRCPNHELGRDFNDGQSAPASHLIRVEGNNLSQYVDDPVTGRQSVMVPYEPPQVGTEFTTILYNFMCNSSCVGGMNRRPILIIITLETRDGQVLGRRSFEGRICACPGRDRKADEDHYREQQALNESAAKNGTASKRTFKQSPQGIPALGTGVKKRRHGEEEMYYVPVRGRENFEFLMKIKESLELVELVPQQLVDSYRQQQQQLLQRQSQLQTPSSYGPVLSPMNKVHGGGINKLPSVNQLVGQPAQHGSGSAPSLGPMGPGMLNTHPMQPNGEMNGGHSSQSMVSGSHCTPPPPYNPDPSLVSFLTGLGCPNCIDYFTSQGLQNIYHLQNLSIEDLGALKIPEQYRMIIWRGLQDLKQSHDYGAQQLIRSSSNASTISIGSSGELQRQRVMEAVHFRVRHTITIPNRGGADEWADFGFDLPDCKSRKQSIKEEFTEGEIN

>NXG89486.1 P73 protein [Stercorarius parasiticus]

VKMSQSSPADEGTTFEHLWSTLEPDSTYFDLPPSNHTGSSEVSNRTEVTMDVFQMRSMNDSVMSQFNLLNNSMDQSIGSRAASTSPYSSEHTSNVPTHSPYSQPSSTFDAMSPAPVIPSNTDYPGPHHFEVTFQQSSTAKSATWTYSPLLKKLYCQIAKTCPIQIKVSTPPPPGTIIRAMPVYKKAEHVTEVVKRCPNHELGRDFNDGQSAPASHLIRVEGNNLSQYVDDPVTGRQSVMVPYEPPQVGTEFTTILYNFMCNSSCVGGMNRRPILIIITLETRDGQVLGRRSFEGRICACPGRDRKADEDHYREQQALNESAAKNGNANKRTFKQSPQGIPALGTGIKKRRHGEEEMYYVPVRGRENFEILMKIKESLELVELVPQQLVDSYRQQQQQLLQRQTQLQTPSSYGPVLSPMNKVHGGGINKLPSVNQLVGQPAQHGSSSAPSLGPMGPGMLNSHPMQPNGEMNGGHSSQPMVSGSHCTPPPPYNPDPSLVSFLTGLGCPNCIDYFTSQGLQNIYHLQNLSIEDLGALKIPEQYRMVIWRGLQELKQSHDYGAQQLIRSSSNASTISIGSSGELQRQRVMEAVHFRVRHTITIPNRGGADEWADFGFDLPDCKSRKQSIKEEFTEGEIN

>NXX11768.1 P73 protein [Podargus strigoides]

VKMSQSSPADEGTTFEHLWSTLEPDSTYFDLPPSSHTGSNEVSNRTEVTMDVFQMRSMNDSVMSQFNLLNSSMDQSIGSRAASTSPYSSEHTSNVPTHSPYSQPSSTFDAMSPAPVIPSNTDYPGPHHFEVTFQQSSTAKSATWTYSPLLKKLYCQIAKTCPIQIKVSTPPPPGTIIRAMPVYKKAEHVTEVVKRCPNHELGRDFNDGQSAPASHLIRVEGNNLSQYVDDPVTGRQSVMVPYEPPQVGTEFTTILYNFMCNSSCVGGMNRRPILIIITLETRDGQVLGRRSFEGRICACPGRDRKADEDHYREQQALNESAAKNGTANKRTFKQSPQSIPALGTSVKKRRHGEEEMYYVPVRGRENFEILMKIKESLELVELVPQQLVDSYRQQQQQLLQRQTQLQTPSSYGPVLSPMNKVHGGGINKLPSVTQLVGQPAQHGSGSAPGLGPMGPGMLNSHPMQPNGEMNGGHSSQSMVSGSQCTPPPPYNPDPSLVSFLTGLGCPNCIDYFTSQGLQNIYHLQNLSIEDLGALKIPEQYRMIIWRGLQELKQSHDYGAQQLIRSSSNASTISIGSSGELQRQRVMEAVHFRVRHTITIPNRGAADEWADFGFDLPDCKSRKQSIKEEFTEGEIN

>NXP29233.1 P73 protein [Scytalopus superciliaris]

KMSQSSPADEGNTFEHLWSTLEPDSTYFDLSPATHTGSNEVSNRTEVTMDVFQMRGMNDSVMSQFNLLNNSMDQSIGSRAASTSPYSSEHASNVPTHSPYSQPSSTFDAMSPAPVIPSNTDYPGPHHFEVTFQQSSTAKSATWTYSPLLKKLYCQIAKTCPIQIKVSTPPPPGTIIRAMPVYKKAEHVTEVVKRCPNHELGRDFNDGQSAPASHLIRVEGNNLSQYVDDPVTGRQSVMVPYEPPQVGTEFTTILYNFMCNSSCVGGMNRRPILIIITLETRDGQVLGRRSFEGRICACPGRDRKADEDHFREQQALNETAAKNGNANKRTFKQSPQSIPALGTSIKKRRHGEEEMYYVPVRGRENFEILMKIKESLELVELVPQQLVDSYRQQQQQLLQRQSQLQTPSSYGPVLSPMNKVHSGGINKLPSVNQLVGQPAQHGPGSAPSLGPMGPGMLNSHPMQPNGEMNGGHSSQSMVSGSHCTPPPPYNPDPSLVSFLTGLGCPNCIDYFTSQGLQNIYHLQNLSIEDLGALKIPEQYRMIIWRGLQELKQSHDYGAQQLIRSSSNASTISIGSSGELQRQRVMEAVHFRVRHTITIPNRGAADDWADFGFDLPDCKSRKQSIKEEFTEGEIN

>NWU47375.1 P73 protein [Dromas ardeola]

VKMSQSSPADEGTTFEHLWSTLEPDSTYFDLPPSNHTGSNEVSNRTEVTMDVFQMRSMNDSVMSQFNLLNNSMDQSIGSRAASTSPYSSEHTSNVPTHSPYSQPSSTFDAMSPAPVIPSNTDYPGPHHFEVTFQQSSTAKSATWTYSPLLKKLYCQIAKTCPIQIKVSTPPPPGTIIRAMPVYKKAEHVTEVVKRCPNHELGRDFNDGQSAPASHLIRVEGNNLSQYVDDPVTGRQSVMVPYEPPQVGTEFTTILYNFMCNSSCVGGMNRRPILIIITLEMRDGQVLGRRSFEGRICACPGRDRKADEDHYREQQALNESAAKNGNANKRTFKQSSQGIPALGTGIKKRRHGEEEMYYVPVRGRENFEILMKIKESLELVELVPQQLVDSYRQQQQQLLQRQTQLQTPSSYGPVLSPMNKVHGGGINKLPSVNQLVGQTAQHSSGSAPSLGPMGPGMLNSHPMQPNGEMNGGHSSQPMVSGSHCTPPPPYNPDPSLVSFLTGLGCPNCIDYFTSQGLQNIYHLQNLSIEDLGALKIPEQYRMIIWRGLQELKQSHDYGAQQLIRSSSNASTISIGSSGELQRQRVMEAVHFRVRHTITIPNRGGADEWADFGFDLPDCKSRKQSIKEEFTEGEIN

>NXI05173.1 P73 protein [Pachycephala philippinensis]

LKMSQSSPADEGTTFEHLWSTLEPDSTYFDLPPANPTGSNEVSDRTEVTMDVFQMRGMTDSVMSQFNLLNNSMDQSIGSRAASTSPYSSEHTSNVPTHSPYSQPSSTFDAMSPAPVIPSNTDYPGPHHFEVTFQQSSTAKSATWTYSPLLKKLYCQIAKTCPIQIKVSTSPPPGTIIRAMPVYKKAEHVTEVVKRCPNHELGRDFNDGQSAPASHLIRVEGNNLSQYVDDPVTGRQSVMVPYEPPQVGTEFTTILYNFMCNSSCVGGMNRRPILIIITLETRDGQVLGRRSFEGRICACPGRDRKADEDHFREQQALNENAAKNGNANKRTFKQSPQGIPALGTGIKKRRHGEEEMYYVPVRGRENFEILMKIKESLELVELVPQQLVDSYRQQQQQLLQRQSQLQTPSSYGPVLSPMNKVHGGGINKLPSVNQLVGQPAQHGSGSAPSLGPMGPGMLNSHPMQPNGEMNGGHSSQSMVSGSHCTPPPPYNPDPSLVSFLTGLGCPNCIDYFTSQGLQNIYHLQNLSIEDLGALKIPEQYRMIIWRGLQELKQSHDYGAQQLIRSSSNASTISIGSSGELQRQRVMEAVHFRVRHTITIPNRGAADDWADFGFDLPDCKSRKQSIKEEFTEGEIN

>NXV41665.1 P73 protein [Uria aalge]

VKMSQSSPADEGTTFEHLWSTLEPDSTYFDLPPSNPTGSNEVSNRTEVTMDVFQMRSMNDSVMSQFNLLNNSMDQSIGSRAASTSPYSSEHTSNVPTHSPYSQPSSTFDAMSPAPVIPSNTDYPGPHHFEVTFQQSSTAKSATWTYSPLLKKLYCQIAKTCPIQIKVSTPPPPGTIIRAMPVYKKAEHVTEVVKRCPNHELGRDFNDGQSAPASHLIRVEGNNLSQYVDDPVTGRQSVMVPYEPPQVGTEFTTILYNFMCNSSCVGGMNRRPILIIITLETRDGQVLGRRSFEGRICACPGRDRKADEDHYREQQALNESAAKNGNANKRTFKQSPQGIPALGTGIKKRRHGEEEMYYVPVRGRENFEILMKIKESLELVELVPQQLVDSYRQQQQQLLQRQTQLQTPSSYGPVLSPMNKVHGGGINKLPPVNQLVGQPAQHGSGSAPSLGPMGPGMLNSHPMQPNGEMNGGHSSQPMVSGSHCTPPPPYNPDPSLVSFLTGLGCPNCIDYFTSQGLQNIYHLQNLSIEDLGALKIPEQYRMIIWRGLQELKQSHDYGAQQLIRSSSNASTISIGSSGELQRQRVMEAVHFRVRHTITIPNRGGADEWADFGFDLPDCKSRKQSIKEEFTEGEIN

>NXB19284.1 P73 protein [Rhagologus leucostigma]

LKMSQSSPADEGTTFEHLWSTLEPDSTYFDLPPANPTGSNEVSNRTEVTMDVFQMRGMTDSVMSQFNLLNNSMDQSIGSRAASTSPYSSEHTSNVPTHSPYSQPSSTFDAMSPAPVIPSNTDYPGPHHFEVTFQQSSTAKSATWTYSPLLKKLYCQIAKTCPIQIKVSTSPPPGTIIRAMPVYKKAEHVTEVVKRCPNHELGRDFNDGQSAPASHLIRVEGNNLSQYVDDPVTGRQSVMVPYEPPQVGTEFTTILYNFMCNSSCVGGMNRRPILIIITLETRDGQVLGRRSFEGRICACPGRDRKADEDHFREQQALNESAAKNGNANKRTFKQSPQGIPALGTGVKKRRHGEEEMYYVPVRGRENFEILMKIKESLELVELVPQQLVDSYRQQQQQLLQRQSQLQTPSSYGPVLSPMNKVHGGGINKLPSVNQLVGQPAQHGSGSAPSLGPMGPGMLNSHPMQPNGEMNGGHSSQSMVSGSHCTPPPPYNPDPSLVSFLTGLGCPNCIDYFTSQGLQNIYHLQNLSIEDLGALKIPEQYRMIIWRGLQELKQSHDYGAQQLIRSSSNASTISIGSSGELQRQRVMEAVHFRVRHTITIPNRGAADDWADFGFDLPDCKSRKQSIKEEFTEGEIN

>NXQ81578.1 P73 protein [Nyctibius grandis]

VKMSQPSPADEGTTFEHLWSTLEPDSTYFDLPPSDHTDSNEVSNRTEVTMDVFQMRSMNDSVMSQFNLLNNSMDQSIGSRAASTSPYSSEHTSNVPTHSPYSQPSSTFDAMSPAPVIPSNTDYPGPHHFEVTFQQSSTAKSATWTYSPLLKKLYCQIAKTCPIQIKVSTPPPPGTIVRAMPVYKKAEHVTEVVKRCPNHELGRDFNDGQSAPASHLIRVEGNNLSQYVDDPVTGRQSVMVPYEPPQVGTEFTTILYNFMCNSSCVGGMNRRPILIIITLETRDGQVLGRRSFEGRICACPGRDRKADEDHYREQQALNESTAKNGNANKRTFKQSPQGIPALGAGVKKRRHGEEEMYYVPVRGRENFEILMKIKESLELVELVPQQLVDSYRQQQQQLLQRQSQLQTPSSYGPVLSPMNKVHGGGINKLPSVNQLVGQPAQHGSGSAPGLGPMGPGMLNSHPMQPNGEMNGGHSSQSMVSGSHCTPPPPYNPDPSLVSFLTGLGCPNCIDYFTSQGLQNIYHLQNLSIEDLGALKIPEQYRMIIWRGLQELKQSHDYGAQQLIRSSSNASTISIGSSGELQRQRVMEAVHFRVRHTITIPNRGGADEWADFGFDLPDCKSRKQSIKEEFTEGEIN

>NWT09776.1 P73 protein [Vireo altiloquus]

LKMSQSSPADEGTTFEHLWSTLEPDSTYFDLPPANPTGSNEVSNRTEVTMDVFQMRGMTDSVMSQFNLLNNSMDQSIGSRAASTSPYSSEHTSNVPTHSPYSQPSSTFDAMSPAPVIPSNTDYPGPHHFEVTFQQSSTAKSATWTYSPLLKKLYCQIAKTCPIQIKVSTSPPPGTIIRAMPVYKKAEHVTEVVKRCPNHELGRDFNDGQSAPASHLIRVEGNNLSQYVDDPVTGRQSVMVPYEPPQVGTEFTTILYNFMCNSSCVGGMNRRPILIIITLETRDGQVLGRRSFEGRICACPGRDRKADEDHFREQQALNESAAKNGNANKRTFKQSPQGIPALGTGIKKRRHGEEEMYYVPVRGRENFEILMKIKESLELVELVPQQLVDSYRQQQQQLLQRQSQLQTPSSYGPVLSPMNKVHGGGINKLPSVNQLVGQPAQHGSGSAPSLGPMGPGMLNSHPMQPNGEMNGGHSSQSMVSGSHCTPPPPYNPDPSLVSFLTGLGCPNCIDYFTSQGLQNIYHLQNLSIEDLGALKIPEQYRMIIWRGLQELKQSHDYGAQQLIRSSSNASTISIGSSGELQRQRVMEAVHFRVRHTITIPNRGAADDWADFGFDLPDCKSRKQSIKEEFTEGEIN

>NWV77432.1 P73 protein [Dasyornis broadbenti]

VKMSQSSPADEGTTFEHLWSTLEPDSTYFDLPPANPTGSNEVSNRTEVKMDVFQMRGMTDSVMSQFNLLNNSMDQSIGSRAASTSPYSSEHTSNVPTHSPYSQPSSTFDAMSPAPVIPSNTDYPGPHHFEVTFQQSSTAKSATWTYSPLLKKLYCQIAKTCPIQIKVSTSPPPGTIIRAMPVYKKAEHVTEVVKRCPNHELGRDFNDGQSAPASHLIRVEGNNLSQYVDDPVTGRQSVMVPYEPPQVGTEFTTILYNFMCNSSCVGGMNRRPILIIITLETRDGQVLGRRSFEGRICACPGRDRKADEDHFREQQALNESAAKNGNANKRTFKQSPQGIPALGTGIKKRRHGEEEMYYVPVRGRENFEILMKIKESLELVELVPQQLVDSYRQQQQQLLQRQSQLQTPSSYGPVLSPMNKVHGGGINKLPSVNQLVGQPAQHGSGSAPSLGPMGPGMLNSHPMQPNGEMNGGHSSQSMVSGSHCTPPPPYNPDPSLVSFLTGLGCPNCIDYFTSQGLQNIYHLQNLSIEDLGALKIPEQYRMIIWRGLQELKQSHDYGAQQLIRSSSNASTISIGSSGELQRQRVMEAVHFRVRHTITIPNRGAADDWADFGFDLPDCKSRKQSIKEEFTEGEIN

>NXD99743.1 P73 protein [Chaetorhynchus papuensis]

LKMSQSSPADEGTTFEHLWSTLEPDSTYFDLPPANPTGSNEVSNRTEVTMDVFQMRGMTDSVMSQFNLLNNSMDQSIGSRAASTSPYSSEHTSNVPTHSPYSQPSSTFDAMSPAPVIPSNTDYPGPHHFEVTFQQSSTAKSATWTYSPLLKKLYCQIAKTCPIQIKVSTSPPPGTIIRAMPVYKKAEHVTEVVKRCPNHELGRDFNDGQSAPASHLIRVEGNNLSQYVDDPVTGRQSVMVPYEPPQVGTEFTTILYNFMCNSSCVGGMNRRPILIIITLETRDGQVLGRRSFEGRICACPGRDRKADEDHFREQQALNESAAKNGNANKRTFKQSPQGIPALGTGVKKRRHGEEEMYYVPVRGRENFEILMKIKESLELMELVPQQLVDSYRQQQQQLLQRQSQLQTPSSYGPVLSPMNKVHGGGINKLPSVNQLVGQPAQHGSGSAPSLGPMGPGMLNSHPMQPNGEMNGGHSSQSMVSGSHCTPPPPYNPDPSLVSFLTGLGCPNCIDYFTSQGLQNIYHLQNLSIEDLGALKIPEQYRMIIWRGLQELKQSHDYGAQQLIRSSSNASTISIGSSGELQRQRVMEAVHFRVRHTITIPNRGAADDWADFGFDLPDCKSRKQSIKEEFTEGEIN

>NXG18435.1 P73 protein [Grallaria varia]

KMSQSSPADEGNTFEHLWSTLEPDSTYFDLSPATHTGSNEVSNRTEVTMDVFQMRGMNDSVMSQFNLLNNSMDQSIGSRAASTSPYSSEHTSNVPTHSPYSQPSSTFDAMSPAPVIPSNTDYPGPHHFEVTFQQSSTAKSATWTYSPLLKKLYCQIAKTCPIQIKVSTPPPPGTIIRAMPVYKKAEHVTEVVKRCPNHELGRDFNDGQSAPASHLIRVEGNNLSQYVDDPVTGRQSVMVPYEPPQVGTEFTTILYNFMCNSSCVGGMNRRPILIIITLETRDGQVLGRRSFEGRICACPGRDRKADEDHFREQQALNESAAKNGNANKRTFKQSPQNIPALGTSIKKRRHGEEEMYYVPVRGRENFEILMKIKESLELVELVPQQLVDSYRQQQQQLLQRQSQLQTPSSYGPVLSPMNKVHSGGINKLPSVNQLVGQPAQHGPGSAPSLGPIGPGMLNSHPMQPNGEMNGGHSSQSMVSGSHCTPPPPYNPDPSLVSFLTGLGCPNCIDYFTSQGLQNIYHLQNLSIEDLGALKIPEQYRMIIWRGLQELKQSHDYGAQQLIRSSSNASTISIGSSGELQRQRVMEAVHFRVRHTITIPNRGAADDWADFGFDLPDCKSRKQSIKEEFTEGEIN

>NXB68893.1 P73 protein [Struthidea cinerea]

LKMSQSSPADEGTTFEHLWSTLEPDSTYFDLPPANPTGSNEVSNRTEVTMDVFQMRGMTDSVMSQFNLLNNSMDQSIGSRAASTSPYSSEHTSNVPTHSPYSQPSSTFDAMSPAPVIPSNTDYPGPHHFEVTFQQSSTAKSATWTYSPLLKKLYCQIAKTCPIQIKVSTSPPPGTIIRAMPVYKKAEHVTEVVKRCPNHELGRDFNDGQSAPASHLIRVEGNNLSQYVDDPVTGRQSVMVPYEPPQVGTEFTTILYNFMCNSSCVGGMNRRPILIIITLETRDGQVLGRRSFEGRICACPGRDRKADEDHFREQQALNESAAKNGNANKRTFKQSPQGIPALGTGVKKRRHGEEEMYYVPVRGRENFEILMKIKESLELVELVPQQLVDSYRQQQQQLLQRQSQLQAPSSYGPVLSPMNKVHGGGINKLPSVNQLVGQPAQHGSGSAPSLGPMGPGMLNSHPMQPNGEMNGGHSSQSMVSGSHCTPPPPYNPDPSLVSFLTGLGCPNCIDYFTSQGLQNIYHLQNLSIEDLGALKIPEQYRMIIWRGLQELKQSHDYGAQQLIRSSSNASTISIGSSGELQRQRVMEAVHFRVRHTITIPNRGAADDWADFGFDLPDCKSRKQSIKEEFTEGEIN

>NXJ80511.1 P73 protein [Trogon melanurus]

VKMSQSSPADEGTTFEHLWSTLEPDSTYFDLPPSNHTGSNEVSDRTEVTMDVFQMRGMNDSVMSQFNLLNNSMDQSIGSRAASTSPYSSEHTSNVPTHSPYSQPSSTFDAMSPAPVIPSNTDYPGPHHFEVTFQQSSTAKSATWTYSPLLKKLYCQIAKTCPIQIKVSTPPPPGTIIRAMPVYKKAEHVTEVVKRCPNHELGRDFNDGQSAPASHLIRVEGNNLSQYVDDPVTGRQSVMVPYEPPQVGTEFTTILYNFMCNSSCVGGMNRRPILIIITLETRDGQVLGRRSFEGRICACPGRDRKADEDHYREQQALNESAAKNGNANKRTFKQSPQGIPALGTGVKKRRHGEEEMYYVPVRGRENFEILMKIKESLELVELVPQQLVESYRQQQQQLLQRQSQLQTSASYGPVLSPMNKVHGTGINKLPSVNQLVGQPAQHGSGSAPGLGPMGPGMLNSHPMQPNGEMNGGHSSQSMVSGSHCTPPPPYNPDPSLVSFLTGLGCPNCIDYFTSQGLQNIYHLQNLSIEDLGALKIPEQYRMIIWRGLQELKQSHDYGAQQLIRSSSSNASTISIGSSGELQRQRVMEAVHFRVRHTITIPNRGAADEWADFGFDLPDCKSRKQSIKEEFTEGEIN

>NXE93554.1 P73 protein [Menura novaehollandiae]

VKMSQSSPADEGATFEHLWSTLEPDSTYFDLPPANHTGSNEVSNSTEVTMDVFQMRGMNDSVMSQFNLLNNSMDQSIGSRAASTSPYSSEHTSNVPTHSPYSQPSSTFDAMSPAPVIPSNTDYPGPHHFEVTFQQSSTAKSATWTYSPLLKKLYCQIAKTCPIQIKVSTPPPPGTIIRAMPVYKKAEHVTEVVKRCPNHELGRDFNDGQSAPASHLIRVEGNNLSQYVDDPVTGRQSVMVPYEPPQVGTEFTTILYNFMCNSSCVGGMNRRPILIIITLETRDGQVLGRRSFEGRICACPGRDRKADEDHFREQQALNESAAKNGNANKRTFKQSPQGIPALGTGIKKRRHGEEEMYYVPVRGRENFEILMKIKESLELVELVPQQLVDSYRQQQQQLLQRQSQLQTPSSYGPVLSPMNKVHSGGINKLPSVNQLVGQPAQHGSGSTSSLGPMGPGMLNSHPMQPNGEMNGGHSSQSMVSGSHCTPPPPYNPDPSLVSFLTGLGCPNCIDYFTSQGLQNIYHLQNLSIEDLGALKIPEQYRMIIWRGLQELKQSHDYGAQQLIRSSSNASTISIGSSGELQRQRVMEAVHFRVRHTITIPNRGAADDWADFGFDLPDCKSRKQSIKEEFTEGEIN

>NWU93816.1 P73 protein [Upupa epops]

KMSQSSPADEGTTFEHLWSTLEPDSTYFDLPPSNHTGDNEVSDRTEVTMDVFQMRSMNDSVMSQFNLLNNSMDQSIGSRAASTSPYSSEHTSNVPTHSPYSQPSSTFDAMSPAPVIPSNTDYPGPHHFEVTFQQSSTAKSATWTYSPLLKKLYCQIAKTCPIQIKVSTPPPPGTIIRAMPVYKKAEHVTEVVKRCPNHELGRDFNDGQSAPASHLIRVEGNNLSQYVDDPVTGRQSVMVPYEPPQVGTEFTTILYNFMCNSSCVGGMNRRPILIIITLETRDGQVLGRRSFEGRICACPGRDRKADEDHYREQQALNESAAKNGSASKRTFKQSPQGIPALGTGIKKRRHGEEEMYYVPVRGRENFEILMKIKESLELVELVPQQLVDSYRQQQQQLLQRQSQLQSPSSYGPVLSPMNKVHGGGINKLPSVNQLVGQPAQHGSSSAPSLGPMGPGMLNSHPMQPNGEMNGGHSSQSMVSGSHCTPPPPYNPDPSLVSFLTGLGCPNCIDYFTSQGLQNIYHLQNLSIEDLGALKIPEQYRMIIWRGLQELKQSHDYGAQQLIRSSSNASTISIGSSGELQRQRVMEAVHFRVRHTITIPNRGAADDWADFGFDLPDCKSRKQSIKEEFTEGEIN

>NXB37644.1 P73 protein [Eulacestoma nigropectus]

VKMSQSSPADEGTTFEHLWSTLEPDSTYFDLPPANPTGGNEVSNRTEVTMDVFQMRGVTDSVMSQFNLLNNSMDQSIGSRAASTSPYSSEHTSNVPTHSPYSQPSSTFDAMSPAPVIPSNTDYPGPHHFEVTFQQSSTAKSATWTYSPLLKKLYCQIAKTCPIQIKVSTSPPPGTIIRAMPVYKKAEHVTEVVKRCPNHELGRDFNDGQSAPASHLIRVEGNNLSQYVDDPVTGRQSVMVPYEPPQVGTEFTTILYNFMCNSSCVGGMNRRPILIIITLETRDGQVLGRRSFEGRICACPGRDRKADEDHFREQQALNESAAKNGNANKRTFKQSPQGIPALGPGIKKRRHGEEEMYYVPVRGRENFEILMKIKESLELVELVPQQLVDSYRQQQQQLLQRQSQLQTPSSYGPVLSPMNKVHSGGINKLPSVNQLVGQPAQHGSGSAPSLGPMGPGMLNSHPMQPNGEMNGGHSSQSMVSGSHCTPPPPYNPDPSLVSFLTGLGCPNCIDYFTSQGLQNIYHLQNLSIEDLGALKIPEQYRMIIWRGLQELKQSHDYGAQQLIRSSSNASTISIGSSGELQRQRVMEAVHFRVRHTITIPNRGAADDWADFGFDLPDCKSRKQSIKEEFTEGEIN

>NXI58028.1 P73 protein [Chloroceryle aenea]

VKMSQASPADEGTTFEHLWSTLEPDSTYFDLPPSNHPGGNEVSNRTEVTMDVFQMRSMNDSVMSQFNLLNNSMDQSIGSRAASTSPYSSEHTSNVPTHSPYSQPSSTFDAMSPAPVIPSNTDYPGPHHFEVTFQQSSTAKSATWTYSPLLKKLYCQIAKTCPIQIKVSTPPPPGTIIRAMPVYKKAEHVTEVVKRCPNHELGRDFNDGQSAPASHLIRVEGNNLSQYVEDPVTGRQSVMVPYEPPQVGTEFTTILYNFMCNSSCVGGMNRRPILIIITLETRDGQVLGRRSFEGRICACPGRDRKADEDHYREQQALNESAAKNGNANKRTFKQSPQGIPALGTGVKKRRHGEEEMYYVPVRGRENFEILMKIKESLELVELVPQQLVDSYRQQQQQQLLQRQNQLQTPSSYGPVLSPMNQGHSGGINKLPSVNQLVGQPAQHSSGSAPSLGPMGPGMLNSHPMQPNGEMNGGHSSQSMVSGSHCTPPPPYNPDPSLVSFLTGLGCPNCIDYFTSQGLQNIYHLQNLSIEDLGALKIPEQYRMIIWRGLQELKQSHDYGAQQLIRSSSNASTISIGSSGELQRQRVMEAVHFRVRHTITIPNRGGADEWADFGFDLPDCKSRKQSIKEEFTEGEIN

**>XP_002933901.3 tumor protein p73 isoform X1 [Xenopus tropicalis]**

MQATLIKQRAVQGAISTDWHFRVIYPLPESPILKMSQSSGADEGTTFEHLWSTLEPDSTYFELPQSSHSNNTEASNRTDVNMDVYQMRTMNESIMSQFNLLNNTMDQSIGSRAASTSPYNPDHTSNVPTHSPYSQPSSTFDAMSPAPVIPSNTDYPGTHNFEVTFQQSSTAKSATWTYSPLLKKLYCQIAKTCPIQIKLSNPPPPGSVIRAMPVFKKAEHVTEVVKRCPNHELGRDFNDGQAAPASHLIRVEGNNLSQYVDDPVTGRQSVMVPYEPPQVGTEFTTILYNFMCNSSCVGGMNRRPILIIITLETRDGQVLGRRSFEGRICACPGRDRKADEDHFREQAALNETAAKNGNANKRTFKQSPPSVPSMGSNIKKRRHGEDEIFYIPVRGRENFEILMKIKESLELVELVPQQLVDSYRQQQQQLLQRQTHLQSTSSYGPVLSPMNKLHGGINKLPSVNQLVGQPNQHNSNAGPNMGPMGPSMLNSHPLQTNGEMNGAHSSQSMVSGSHCTPPPPYNADPSLVSFLTGLGCPNCIEYFTSQGLQNIYHLQNLTMEDLGALKIPEHYKSMIWRGIQELNKSHEYGAQQLVRSSSNASTISIGSSGELQRQRVMEAVHFRVRHTITIPNRGGADEWADFGFDLPDCKSRKQSIKEEYENSDIN

>XP_018083483.1 tumor protein p73 isoform X1 [Xenopus laevis]

MQATLIKQRTVQGTIISTDWHFRVIYPLPESPSVKMSQSTGADEGTTFEHLWSTLEPDSTYFELPQSSHSNNTEASNRTDVNMDVYQMRSMNESIMSQFNLLNNTMDQSIGSRAASTSPYNPDHTSNVPTHSPYSQPSSTFEAMSPAPIIPSNTDYPGTHNFEVTFQQSSTAKSATWTYSPLLKKLYCQIAKTCPIQIKLSNPSPPGSVIRAMPVFKKAEHVTEVVKRCPNHELGRDFNDGQAAPASHLIRVEGNNLSQYVDDPVTGRQSVMVPYEPPQVGTECTTILYNFMCNSSCVGGMNRRPILIIITLETRDGQVLGRRSFEGRICACPGRDRKADEDHFREQAALNETAAKNGNANKRTFKQSPPSVPTVGSNIKKRRHGEDEIFYIPVRGRENFEILMKIKESLELVELVPQQLVDSYRQQQQQLLQRQTHMQSTSSYGPGLSPMNKHHGGINKLPSVNQLVGQPNQHNSNAGPNLGPMGPGMLNSHPLQTNGEMNGAHSSQSMVSGSHCTPPPPYSADPSLVSFLTGLGCPNCIEYFTSQGLQNIYHLQNLTMEDLGALKIPEHYKAVIWRGIQELNKSHDYGAQQHVRSSSNASTISIGSSGELQRQRVMEAVHFRVRHTITIPNRGGADEWADFGFDLPDCKSHKQSIKEEYENSDIN

>XP_040182394.1 tumor protein p73 isoform X1 [Rana temporaria]

MPVTPTRQRAVQGTIRTDWDLRVIYPLSDSPSVKMSHSSPADEGTTFEHLWSTLEPDSNYFDLPQSTTHSNTEVTNRTEVNMDVYQMRNMNQSIMSQFNLLNNTMDQSIGSRAASTSPYNPEHNSNVPTHSPYSQPSSTFDAMSPAPVIPSNTDYPGPHNFEVTFQQSSTAKSATWTYSPLLKKLYCQIAKTCPIQIKLSSPPPPGTVIRAMPVYKKAEHVTEVVKRCPNHELGRDFNEGQSAPASHLIRVEGSNLSQYVDDPVTGRQSVMVPFEPPQVGTEYTTILYNFMCNSSCVGGMNRRPILIIITLETRDGQVLGRRSFEGRICACPGRDRKADEDHFREQAALNENASKNGNSNKRSECTITACKQSPPGVTSIGANMKKRRHGEEEIYYVPVRGRENFEILMKIKESLELVELVPQQLVDSYRQQQQQLLQRHAHIQSQSSYGPVLSPMNKVHGGMNKLPSVNQLVGHPNQHNSNNAPGMMQMGSGMVNNHSMQSNGEMNGAHSSQSMVSGSHCTPPPPYNADPSLVSFLTGLGCPNCIEYFTSQGLQNIYHLQNLTMEDLGALKIPEHYKAIIWRGIQELNKGHDYGGQQLIRSSSNASTISIGSSGELQRQRVMEAVHFRVRHTITIPNRNGADEWADFGFDLPDCKARKHSIKEEFAESDIN

>NXI13646.1 P73 protein [Irena cyanogastra]

VKMSQSSPADEGTTFEHLWSTLEPDSTYFDLPGSNPSGSSEVSNRTEVTMDVFQMRGMTDSVMSQFNLLNNSMDQSIGSRAASTSPYSSEHTSNVPTHSPYSQPSSTFDAMSPAPVIPSNTDYPGPHHFEVTFQQSSTAKSATWTYSPLLKKLYCQIAKTCPIQIKVSTSPPPGTIIRAMPVYKKAEHVTEVVKRCPNHELGRDFNDGQSAPASHLIRVEGNNLSQYVDDPVTGRQSVMVPYEPPQVGTEFTTILYNFMCNSSCVGGMNRRPILIIITLETRDGQVLGRRSFEGRICACPGRDRKADEDHFREQQALNESAAKNGNANKRTFKQSPQGIPALGTGIKKRRHGEEEMYYVPVRGRENFEILMKIKESLELVELVPQQLVDSYRQQQQQLLQRQSQLQTPSSYGPVLSPMNKAHGGGINKLPSVNQLVGQPAQHGSSSAPSLGPMGPGMLNSHPMQSNGEMNGGHSSQSMVSGSHCTPPPPYNADPSLVSFLTGLGCPNCIDYFTSQGLQNIYHLQNLSIEDLAALKIPEQYRMIIWRGLQELKQSHDYGAQQLIRSSSNASTISIGSSGELQRQRVMEAVHFRVRHTITIPNRGTADDWADFGFDLPDCKSRKQSIKEEFTEGEIN

>NXL72740.1 P73 protein [Leptocoma aspasia]

VKMSQSSPADEGTTFEHLWSTLEPDSTYFDLPAANPSGSSEVSNRTEVTMDVFQMRGMTDSVMSQFNLLNNSMDQSIGSRAASTSPYSSEHTSNVPTHSPYSQPSSTFDAMSPAPVIPSNTDYPGPHHFEVTFQQSSTAKSATWTYSPLLKKLYCQIAKTCPIQIKVSTSPPPGTIIRAMPVYKKAEHVTEVVKRCPNHELGRDFNDGQSAPASHLIRVEGNNLSQYVDDPVTGRQSVMVPYEPPQVGTEFTTILYNFMCNSSCVGGMNRRPILIIITLETRDGQVLGRRSFEGRICACPGRDRKADEDHFREQQALNESTAKNGNANKRTFKQSPQGIPALGTGIKKRRHGEEEMYYVPVRGRENFEILMKIKESLELVELVPQQLVDSYRQQQQQLLQRQSQLQTPPSYGPVLSPMNKVHGGGINKLPSVNQLVGQPAQHGSSSAPSLGPMGPGMLNSHPMQTNGEMNGGHSSQSMVSGSHCTPPPPYNADPSLVSFLTGLGCPNCIDYFTSQGLQNIYHLQNLSIEDLAALKIPEQYRMIIWRGLQELKQSHDYGAQQLIRSSSNASTISIGSSGELQRQRVMEAVHFRVRHTITIPNRGAADDWADFGFDLPDCKSRKQSIKEEFTEGEIN

>NXO20074.1 P73 protein [Cisticola juncidis]

VKMSQSSPADEGTTFEHLWSTLEPDSTYFDLPPANPTGSSEVSNSTEVTMDVFQMRGIPDSVMSQFNLLNNSMDQSIGSRAASTSPYSSEHTSNVPTHSPYSQPSSTFDAMSPAPVIPSNTDYPGSHHFEVTFQQSSTAKSATWTYSPLLKKLYCQIAKTCPIQIKVSSSPPPGTIIRAMPVYKKAEHVTEVVKRCPNHELGRDFNDGQSAPASHLIRVEGNNLSQYVDDPVTGRQSVMVPYEPPQVGTEFTTILYNFMCNSSCVGGMNRRPILIIITLETRDGQVLGRRSFEGRICACPGRDRKADEDHFREQQALNESTAKNGNANKRTFKQSPQGIPALGTGVKKRRHGEEEVYYVPVRGRENFEILMKIKESLELVELVPQQLVDSYRQQQQQLLQRQSQLQTPSSYGPVLSPMNKVHGGGINKLPSVNQLVGQPAQHSSSSAPSLGPMGPGMLNSHPMQTNGEMNGGHSSQSMVSGSHCTPPPPYNADPSLVSFLTGLGCPNCIDYFTSQGLQNIYHLQNLSIEDLGALKIPEQYRMLIWRGLQELKQSHDYGAQQLIRSSSNASTISIGSSGELQRQRVMEAVHFRVRHTITIPNRGNADDWADFGFDLPDCKSRKQSIKEEFTEGEIN

>XP_029433784.1 tumor protein p73 isoform X2 [Rhinatrema bivittatum]

MSRSSSTDEGTTFEHLWSTLEPDSTYFDLPQSSHSGNSEVSNRTEVNMDVFQMRSMSDSVMSQFNLLNSSMDQSVGSRAASTSPYSSELASNVPSHSPYSQPSSTFDAMSPAPVIPSNTDYPGPHNFEVTFQQSSTAKSATWTYSPLLKKLYCQIAKTCPIQIKVSTPPPPGTGIRAMPVYKKAEHVTEVVKRCPNHELGRDFNDGQAAPASHLIRVEGNNLSQYVDDPVTGRQSVMVPYEPPQVGTEFTTILYNFMCNSSCVGGMNRRPILIIITLETRDGQVLGRRSFEGRICACPGRDRKADEDHYREQQALNESAAKNGNANKRTFKQSPQGIPTLNANIKKRRHGEEEIYYLPVRGRENFEFLMKIKESLELVELVPQQLVDSYRQQQQQLLQRQSHLQSSSSYGPVLSPMSKVHSGINKLPSVNQLVGQPPQHTSSTGSNLGPMGPGMLNSHPMQPNGEMNGAHSSQSMVSTSHCTPPPPYNPDPGLVSFLTGLGCPNCIEYFTSQGLQNIYHLQNLTMEDLGALKIPEQYRMTIWRGLQELKQSHDYGAQQLIRSSSNASTISIGSSGELQRQRVMEAVHFRVRHTITIPNRSGADEWADFGFDLPDCKSRKQSIKEEFTEGEI

>XP_033777653.1 tumor protein p73 isoform X2 [Geotrypetes seraphini]

MSRSSSNDEGTTFEHLWGALEPDSTYFDLPQSSHPGNNEVSNQTEVNMDVFQMRNMNDSVMSQFSLLNNSMDQNISSRAASTSPYSSELASNVPTHSPYSQPSSTFDAMSPAPVIPSNTDYPGPHNFEVTFQQSSTAKSATWTYSPLLKKLYCQIAKTCPIQIKLSTPPPPGTGIRAMPVYKKAEHVTEVVKRCPNHELGRDFNDGQAAPASHLIRVEGNNLSQYVDDPVTGRQSVMVPYEPPQVGTEFTTILYNFMCNSSCVGGMNRRPILIIITLETRDGQVLGRRSFEGRICACPGRDRKADEDHYREQQALNESAAKNGNANKRTFKQSPPGIPTLNANIKKRRHGEEELYYLPVRGRENFEILMKVKESLELVELVPQQLVDSYRQQQQQLLQRQSHLQSPSSYGPVLSPMNKVHGGVNKLPSVNQLVGQPPQHSSNTGPNLGHMGCGMLNSHPMQSNGEMNGAHSSQSMVSTSHCTPPPPYNPDPGLVSFLTGLGCPNCIEYFTSQGLQNIYHLQNLTMEDLGALKIPEQYRVTIWRGLQELKQSHDYGTQQLIRSSSNASTISIGSSGELQRQRVMEAVHFRVRHTITIPNRSGADEWADFGFDLPDCKSRKQSIKEEFTEAELN

>XP_030041693.1 tumor protein p73 isoform X1 [Microcaecilia unicolor]

MPVKVVALYSLGFFAAELDYRVIYPSSNSPLVKMSRSSSTDEGTTFEHLWSTLEPDSTYFDLPQSSHSGNNEVSNQTEVNMDVFQMRSMNDSVMSQFSLLNNSMDQNISSRAASTSPYSSELASNVPTHSPYSQPSSTFDAMSPAPVIPSNTDYPGPHNFEVTFQQSSTAKSATWTYSPLLKKLYCQIAKTCPIQIKVSTPPPPGTGIRAMPVYKKAEHVTEVVKRCPNHELGRDFNDDCVSSGQAAPASHLIRVEGNNLSQYVDDPVTGRQSVMVPYEPPQVGTEFTTILYNFMCNSSCVGGMNRRPILIIITLETRDGQVLGRRSFEGRICACPGRDRKADEDHYREQQALNESAAKNGNANKRNFKQSPPGIPTLNANIKKRRHGEEEIYYLPVRGRENFEILMKVKESLELVELVPQQLVDSYRQQQQQLLQRQSHLQSPSSYGPVLSPMNKVHCSVNKLPSVNQLVGQPPQHSSNTGPNLGPMGSGMLNSHPMQSNGEMNGAHSSQSMVSTSHCTPPPPYNPDPGLVSFLTGLGCPNCIEYFTSQGLQNIYHLQNLTMEDLGALKIPEQYRVIIWRGLQELKQSHDYGTQQLIRSSSNASTISIGSSGELQRQRVMEAVHFRVRHTITIPNRSGADEWADFGFDLPDCKSRKQSIKEEFTEAELN

>XP_040285897.1 tumor protein p73 isoform X4 [Bufo bufo]

MSNSSPADEGTTFEHLWSTLEPDSNYFDLPQSSHSSNSEVSNQTEVNMDAYQMRNMNESIMSQFSLLNNTMDQSIGSRAASTSPYNPDHNSNVPTHSPYSQPSSTFDAMSPAPVIPSNTDYPGPHNFEVTFQQSSTAKSATWTYSPLLKKLYCQIAKTCPIQIKLSCPPPPGTVIRAMPVYKKAEHVTEVVKRCPNHELGRDFNDGQAAPASHLIRVEGNNLSQYVDDPVTGRQSVMVPFEPPQVGTEFTTILYNFMCNSSCVGGMNRRPILIIITLETRDGQVLGRRSFEGRICACPGRDRKADEDHFREQAALNETASKNGNANKRTCKQSPQGIPALGPNIKKRRNGEEEIYYVPVRGRENFEILMKIKESLELVELVPQQLVDSYRQQQQQLLQRHAHLQSQSSYGPLISPMNKVHGGINKLPSVNQLVGQPNQHSANTAPGMMPMGPGMLNNHSMHLNGEMNGAHSSQSMVSGSHCTPPPPYNADPSLVSFLTGLGCPNCIEYFTSQGLQNIYHLQNLTMEDLGALKIPEHYKGLIWRGIQELNKSHDYGTQQLIRSSSNASSISIGSSGELQRQRVMEAVHFRVRHTITIPNRSGADDWADFGFDLPDCKARKQSIKEEFAESDIN

>XP_032863541.1 tumor protein p73 [Tyto alba alba]

MSQSSPADEGTTFEHLWSTLEPDSTYFDLPPSNHTGSNEVSNRTEVTMDVFQMRSMNDSVMSQFNLLNNSMDQSIGSRAASTSPYSSEHTSNVPTHSPYSQPSSTFDAMSPAPVIPSNTDYPGPHHFEVTFQQSSTAKSATWTYSPLLKKLYCQIAKTCPIQMKVSTPPPPGTIIRAMPVYKKAEHVTEVVKRCPNHELGRDFNDGQAAPASHLIRVEGNNLSQYVDDPVTGRQSVMVPYEPPQVGTEFTTILYNFMCNSSCVGGMNRRPILIIITLETRDGQVLGRRSFEGRICACPGRDRKADEDHYREQQALNESAAKNGNANKRTFKQSPQGIPALGTSIKKRRHGEEEMYYVPVRGRENFEILMKIKESLELVELVPQQLVDSYRQQQQQLLQRQSQLQTPSSYGPVLSPMNKVHGGGGINKLPSVSQLVGQPAQHSSSSTPSLGPVGPGMLNSHPMQPNGEMNGGHSSQSMVSGSHCTPPPPYNPDPSLVSFLTGLGCPNCIDYFTSQGLQNIYHLQNLSIEDLGALKIPEQYRMIIWRGLQELKQSHDYGAQQLIRSSSNASTISIGSSGELQRQRVMEAVHFRVRHTITIPNRGGADEWADFGFDLPDCKSRKQSIKEEFTEGEIN

**>NP_899183.1 tumor protein p73 [Danio rerio]**

MSQSSTADEGPTFEHLWSTLEPDSTYFELPQAGHSGDRASSSLPGNRAEVCMDVYHMRDMRDMNDNVMSQYSLLSSSMDQGLGNRAASTSPYSSETTSNVPTPSPYSQPNSTFEAMSPAPAIPSNTDYPGPHNFEVTFQQSSTAKSATWTYSPLLKKLYCQIAKTCPIQIKLASSPPNGSVIRAMPIYKKAEHVTEVVKRCPNHKLGRDFNESQTAPASHLIRVEGNNLCQYVDDPVTGRQSVLVPYESPQVGTEFTTILYNFMCNSSCVGGMNRRPILIIITLETRDGQVLGRRSFEGRICACPGRDRKADEDHFREQQALNESVAKNGNANKRNFKQTPTNITGPSINIKKRRHGEEEMYYIPVRGRENFDILMKIKDSLELVEFVPQQLVDSYRQQQQQLLQRQNHVASPSSYGTLNNMNKIHGPISKLPSVNQLVTQQTQQSAGPSASLSHMGANMLGGHHMQSNGDVNGAHQSQSIVSTSHCTPPPPYNPDPSLVSFLTSLGCQNCIDYFTSQGLQSVYHLQTLTMEDLGALKIPEQFRLAIWRGLQEMKQGHDYGQQLIRSSSNMATMAIGPSGELQRQRVMEAVHFRVRHTITIPNRGPANGPEEWPDFGFDMPDCRLHKHSIKEEFAEGDVH

>RXN12647.1 tumor p73 isoform X1 [Labeo rohita]

MSQSSTADEGTTFEHLWSTLEPDSTYFELPQAGHSGDRVASSSLPSNRAEVCMDVYHMRDMRDMNDNVMSQYSLLSSSMEQGLGNRAASTSPYSSETTSNVPTPSPYSQPNSTFEAMSPAPAIPSNTDYPGPHNFEVTFQQSSTAKSATWTYSPLLKKLYCQIAKTCPIQIKLAASPPNGSVIRAMPIYKKAEHVTEVVKRCPNHELGRDFNESQTAPASHLIRVEGNNLCQYVDDPVTGRQSVLVPYEAPQVGTEFTTILYNFMCNSSCVGGMNRRPILIIITLETRDGQVLGRRSFEGRICACPGRDRKADEDHFREQQALNESVAKNGNANKRNFKQTPSNITGPSINIKKRRHGEEEMYYIPVRGRENFDILMKIKDSLELVEFVPQQLVDSYRQQQQQLLQRQSHVASPSPYGTLNNMNKIHGPINKLPSVNQLVTQQTQQNAGPSASMSHMGTNMLGGHHMQSNGDVNGAHASQSIVSTSHCTPPPPYNPDPSLVSFLTSLGCQNCIDYFTSQGLQSVYHLQTLSMEDLGALKIPEQFRLAIWRGLQDMKQGHDYGQQLIRSSSNMATMAIGPGGELQRQRVMEAVHFRVRHTITIPNRGAPTGAEEWADFGFDMPDCRIHKHSIKEEFAEECQKETVPVNPSMGNHQRSETSLNGSGRHSATKGIVIAMICTFFDAFNVPVFWPILVMYFIMLFCITMKRQIKHMIKYRYLPFTHGKRTYRGKEDTGKTFAS

>AAD27752.1 p73 [Barbus barbus]

MSQSSTADEGTTFEHLWSTLEPDSTYFELPQAGHSGDRVASSSLPSNRAEVCMDVYHMRDMRDMNDNVMSQYSLLSSSMEQGLGNRAASTSPYSSETTSNVPTPSPYSQPNSTFEAMSPAPAIPSNTDYPGPHNFEVTFQQSSTAKSATWTYSPLLKKLYCQIAKTCPIQIKLASSPPNGSVIRAMPIYKKAEHVTEVVKRCPNHELGRDFNESQTAPASHLIRVEGNNLSQYVDDPVTGRQSALVPYEAPQVGTEFTTILYNFMCNSSCVGGMNRRPILIIITLETRDGQVLGRRSFEGRICACPGRDRKADEDHFREQQALNESVAKNGNANKRNFKQTPGNITGPSINIKKGRHGEEEMYYIPVRGRENFDILMKIKDSLELVEYVPQHFVDSYRQQQQQLLQRQSHVASPSPYGTLNNMNKIHGSISKLPSVNQLVTQQTQQSAGPSASMSHMGTNMLGGHHMQSNGDVNGAHASQSLVSTSHCTPPPPYNPDPSLVSFLTSLGCQNCIDYFTSQGLQSVYHLQTLSMEDLGALKIPEQFRMAIWRGLQDMKQGHDYGQQLIRSSSNMATMAIGGGGELQRQRVMEAVHFRVRHTITIPNRGAPTGAEEWADFGFDMPDCRIHKHSIKEEFAESDVH

>XP_026126345.1 tumor protein p73 isoform X1 [Carassius auratus]

MIFSVICPALDRPTSRMSQSSTADEGTTFEHLWSTLEPDSTYFELPQAGHSGDRVASSSNRAEVCMDLYHMRDMRDMNDNVMSQYSLLSSSMEQGLGNRAASSSPYSSETASNVPTPSPYSQPNSTFEAMSPAPAIPSNTDYPGPHNFEVTFQQSSTAKSATWTYSPLLKKLYCQIAKTCPIQIKLASSPPNGSVIRAMPIYKKAEHVTEVVKRCPNHELGRDFNESQTAPASHLIRVEGNNLCQYVDDPATGRQSALVPYEAPQVGTEFTTILYNFMCNSSCVGGMNRRPILIIITLETRDGQVLGRRSFEGRICACPGRDRKADEDHFREQQALNESVAKNGNANKRNFKQTPANIAGPSVNIKKRRHGEEEMYYIPVRGRENFDILMKIKDSLELVEFVPQQLVDSYRQQQQQLLQRQSHVASPCSYGTLNNMNKIQGPISKLPSVNQLVTQQTQQSAGPSASMTHMGTNMLGGHHMQSNGDVNGAHASQSIVSTSHCTPPPPYNPDPSLVSFLTSLGCQNCIDYFTSQGLQSVYHLQTLSMEDLGALKIPEQFRMAIWRGLQDMRQGHDYGQQLIRSSSNMASMAIGAGGELQRQRVMEAVHFRVRHTITIPNRSAPAGAEEWADFGFDMPDCRIHKHSIKEEFAESDAR

>XP_039525791.1 tumor protein p73 isoform X1 [Pimephales promelas]

MSQSSTADEGTTFEHLWSTLEPDSTYFELPQAGHSGDRASSSLPSNRAEVCMDVYHMRDMNDNVMSQYSLLSSSMEQGLGNRPASTSPYSSETTSNVPTPSPYSQPNSTFEAMSPAPAIPSNTDYPGPHNFEVTFQQSSTAKSATWTYSPLLKKLYCQIAKTCPIQIKLASSPPNGSVIRAMPIYKKAEHVTEVVKRCPNHELGRDFNESQTAPASHLIRVEGNNLSQYVDDPVTGRQSVLVPYENPQVGTEFTTILYNFMCNSSCVGGMNRRPILIIITLETRDGQVLGRRSFEGRICACPGRDRKADEDHFREQQALNDSVAKNGNANKRNFKQNPANITGPSINIKKRRHGEEEMYYIPVRGRENFDILMKIKDSLELVEFVPQQLVDSYRQQQQQLLQRQSHITSPSPYGTLNNMNKMHGSISKLPSVNQLVTQQTQQSAGPSASMSHMGTNMLGGHHMQSNGDVNGAHPSQSIVSTSHCTPPPPYNPDPSLVSFLTSLGCQNCIDYFTSQGLQSVYHLQTLSMEDLGALKIPEQFRLAIWRGLQDMKQGHDYIRSSSNMATMAIGPGGELQRQRVMEAVHFRVRHTITIPNRSGATGGDEWADFGFDMPDCRIHKHSIKEEFAESDLR

>KAA0713690.1 Tumor protein p73 [Triplophysa tibetana]

MSQSSTADEGTTFEHLWSTLEPDSTYYELPQPGHSGDRVATSSLPSNRAEVCMGVYHMRDINDNVMSQYSLLSSSMDQSLGNRAASTSPYSSENTSNVPTPSPYSQPNSTFEAMSPAPAIPSNTDYPGPHNFEVTFQQSSTAKSATWTYSPLLKKLYCQIAKTCPIQIKLASSPPNGSVIRAMPIYKKAEHVTEVVKRCPNHELGRDFNESQTAPASHLIRVEGSNLCQYVDDPVTGRQSVLVPFEAPQVGTEFTTILYNFMCNSSCVGGMNRRPILIIITLETRDGQVLGRRSFEGRICACPGRDRKADEDHFREQQALNESVAKNGNANKRNFKQTPANLTGPSINIKKRRHGEEEMYYIPVRGRENFDILMKIKDSLELVDFVPQQLVDSYRQQQQQILQRQGQVASASTYGTLNNMNMNKIHGTMGKLPSVNQLVSQQNQQNAGPSASMSHMGTNMLGGHHMQTNGDLNGGHSSQSIVSTSHCTPPPPYNPDPSLVSFLTNLGCQNCIDYFTSQGVQSVYHLQTLTMEDLGALKIPEQFRVAIWRGLQDMKQGQDYGQQLIRSSSNMATMSIGPGGELQRQRVMEAVHFRVRHTITIPNRGGSTGGEEWGDFGFDMPDCRIHKHSIKEEFAESNVH

>XP_037392143.1 tumor protein p73 isoform X1 [Pygocentrus nattereri]

MSKMSQSSTSDEGTTFEHLWSSLEPDSTYFELPQTSHPGDRGPSSSLPNNRAEVCMDVYQMRDLNDNVMSQYSLLSSSMEQGLGNRAASTSPYSSETASNVPTPSPYSQPNSTFEAMSPAPAIPSNTDYPGPHSFEVTFQQSSTAKSATWTYSPLLKKLYCQIAKTCPIQVKLASAPPHGSVVRAMPIYKKAEHVTEVVKRCPNHELGREFNESQSAPASHLIRVEGNNLCQYVDDPVTGRQSVLVPYEAPQVGTEFTTILYNFMCNSSCVGGMNRRPILIIITLETRDGQVLGRRSFEGRICACPGRDRKADEDHFREQQALNESVAKNGNANKRNFRQTPPNIPGTPINIKKRRHGEEEMYYIPVRGRENFDLLMKIKDSLELVELVPQQLVDSYRQQQQQLLHRQGHVTSPSSYGTPLSNMNKVHGNISKLPSVNQIVAHQTQQNAGPSSMAHMGANMLGGHHMQSNGDVNGAHSAQAMVSASHCTPPPPYNPDPSLVSFLTSLGCQNCIDYFTSQGLQSIYHLQTLSMEDLGALKIPEQFRLAIWRGLQDMKQSHDYGQQLIRSSSNMATMAIGPSGELQRQRVMEAVHFRVRHTITIPNRGGPTGGEEWADFGFDMPDCKTRKHSIKEEFAEGDIR

>XP_026867091.1 tumor protein p73 [Electrophorus electricus]

MSKMSQSSTSDEGTTFEHLWSSLEPDSTYFELPQTSHPGDRGPSSSLPNSRAEVCMDVYQMRDLNDNVMSQYSLLSSSMEQGLGNRAASASPYSSETASNVPTPSPYSQPNSTFEAMSPAPAIPSNTDYPGPHGFEVTFQQSSTAKSATWTYSPLLKKLYCQIAKTCPIQIKLASAPPHGTAVRAMPIFKKAEHVTEVVKRCPNHELGRDFNESQSAPASHLIRVEGNNLCQYVDDPVTGRQSVLVPYETPQVGTEFTTILYNFMCNSSCVGGMNRRPILIIITLETRDGQVLGRRSFEGRICACPGRDRKADEDHFREQQALNESVAKNGTANKRNFRQSPPNIPGPSINVKKRRHGDEEMFYIPVRGRENFDLLMKIKDSLELVELVPQQLVDSYRQQQQLMHRQGHVTSPSSYGPALGNMSKAHSNISKLPSVNQIVAHQSQQNAGPSASMAHMGANMLGSHHMQSNGDVNGAHPVQSMVSASHCTPPPPYNPDPNLVSFLTSVGCQNCIDYFTSQGLQSVYHLQSLSMEDLGALKIPEQFRVAIWRGLQDMKQGHHDYGQQLIRSSSNLATMAIGPGGELQRQRVMEAVHFRVRHTITIPNRGGPASGEEWPDFGFDMPDCKARKHSVKEEFAESDVR

>XP_027022700.1 tumor protein p73 isoform X4 [Tachysurus fulvidraco]

MSKMSQSSTSDEGTTFEHLWSSLEPDSTYFELPQTSHPERGASSAMPNNRAEVCMDVYQMRDLNDNVMSQYSLLSSSMEQGLGNRAASTSPYSSETASNVPTPSSYSQPNSTFEAMSPAPAIPSNTDYPGPHGFDVTFQQSSTAKSATWTYSPLLKKLYCQIAKTCPIQVKLASTPPHGSVVRAMPIYKKAEHVTEVVKRCPNHELGRDFNESQSAPASHLIRVEGNNLCQYVDDPVTGRQSVLVPYEAPQVGTEFTTILYNFMCNSSCVGGMNRRPILIIITLETRDGQVLGRRSFEGRICACPGRDRKADEDHFREQQALNESVAKNGNANKRNFRQTPPNITGPPLNIKKRRHGDEEMYYIPVRGRENFDILMKIKDSLELVELVPQQIVDSYRQQQQQLLHRHGHVTSPSPYATPLSNMNKVHSNINKLPSVNQIVAHQSHQNAGPSASMVHMGANMLGGHHMQSNGDVNGAHTAQSMVSASHCTPPPPYNPDPSLISFLTSLGCQNCIDYFTSQGLQSVYHLQTLSMEDLGALKIPEQFRLAIWRGLQDMKQGHDYGQQLIRSSSNMATMAIGPGGELQRQRVMEAVHFRVRHTITIPNRSNTAGADEWPDFGFDMPDCKTRKNSIKEEFAESDMH

>XP_022526406.1 tumor protein p73 isoform X1 [Astyanax mexicanus]

MKDWRWLPLQRVIYPSVEHSMSKMSQSSTNDEGTTFEHLWSSLEPDSTYFELPQTGHPGDRGPSNSLPNNRAEVCMDVYQMRDLNDNVMSQYSLLSSSMEQGLGNRAASTSPYSSETASNVPTPSPYSQPNSTFEAMSPAPAIPSNTDYPGPHSFEVTFQQSSTAKSATWTYSPLLKKLYCQIAKTCPIQVKLASAPPHGSVVRAMPIYKKAEHVTEVVKRCPNHELGREFNESQSAPASHLIRVEGNNLSQYVDDPVTGRQSVFVPYETPQVGTEFTTILYNFMCNSSCVGGMNRRPILIIITLETRDGQVLGRRSFEGRICACPGRDRKADEDHFREQQALNESVAKNGNANKRNFRQTPPNIPGPPINIKKRRHGEEEMYYIPVRGRENFDLLMKIKDSLELVELVPQQLVDTYRQQQQQLLHRQGHVTSPSSYGTPMSNMNKVHGNISKLPSVNQIVAHQTQQGAGPSSMAHMGANMLGGHHMQTNGDVNGAHSGQSMVSASHCTPPPPYNPDPSLVSFLTSLGCQNCIDYFTSQGLQSIYHLQTLTMEDLGALKIPEQYRLAIWRGLQDMKQSHDYGQQLIRSSSNMATMAIGPSGELQRQRVMEAVHFRVRHTITIPNRGGPTGGEDWADFGFDMPDCKTRKHSIKEEYAEGDVH

>XP_030639382.1 tumor protein p73 isoform X1 [Chanos chanos]

MKDWRWLPLQRVIYPSVDYPTAKMSQSSTSDETPTFEHLWSSLEPDSTYFELPQTSHSGERVVSTSLPSNRAEVCMDVYHMRDLNDNVMSQYSLLSNSMEHSLANRAASTSPYSSETASNVPTPSPYSQPNSTFDAMSPAPAIPSNADYPGPHNFEVTFQQSSTAKSATWTYSPLLKKLYCQIAKTCPIQIKLSSAPPHGSAIRAMPIYKKAEHVTEVVKRCPNHELGRDFNDSQAAPASHLIRVEGNNLCQYVDDPVTGRQSVLVPYESPQVGTEFTTILYNFMCNSSCVGGMNRRPILIIITLETRDGQVLGRRSFEGRICACPGRDRKADEDHHREQQALNESVAKNSNANKRNFKQTPPNIPGPSVNIKKRRHGEDELYYVPVRGRENFDILMKIKDSLELVELVPQQIVESYRQQQQQLLQRQSQVTSPSAYSNPLSNMNKIHGGVGKLPSVNQLVPHQSQQSAGPSAVMFFIMSGPNMLSGHHMQTNGDLNGTHPAQSMVSASHCTPPPPYNPDPSLVSFLTSLGCQNCIDYFTSQGLQSVYHLQTLSMEDLGALKIPEQFRLAIWRGLQDMKQGHDYGQQLIRSSSNMATMAIGPSGELQRQRVMEAVHFRVRHTITIPNRGGPAGADDWADFGFDMPDCKTTRRQNSIKEEFTESDVH

>XP_018592106.1 tumor protein p73 isoform X1 [Scleropages formosus]

MSQSSSADEGSTFEHLWSTLEPDSTYFELPQGSHPGNHETPSSLPSNCAEVCMDVFHMRDMNETVMSQYSLLSSSMEPTLTGRAAPGSPYGSEHASSVPTPSPYSQPNSTFDAMSPAPAIPSNTDYPGPHNFEVTFQQSSTAKSATWTYSPLLKKLYCQIAKTCPIQIKLSSAPPPGSVIRAMPVYKKAEHVTEVVKRCPNHELGRDFNDGQSAPASHLIRVEGNNLSQYVDDPVTGRQSVLVPYETPQVGTEFTTILYNFMCNSSCVGGMNRRPILIIITLETRDGQVLGRRSFEGRICACPGRDRKADEDHFREQQALNDSVAKNGNANKRTFKQSPPNIPGPAINIKKRRHGEEEIYYIPVRGRENFDILMKIKDSLELVELVPQQLVDSYRQQQQQLLQRQSHVPSPSSYGSPMGGMSKIHGGMSKLPSVNQLVGQQSQQSPGPMPGLSHMGSNMVNSHHMQANGDMNGGHSSQPMASASHCTPPPPYNPDPSLVSFLTSLGCQNCIDYFTSQGLQSVYHLQTLSMEDLGALKIPEQFRLAIWRGLQDMKQGHDYGQQLIRSSSNTATMAIGPSGELQRQRVMEAVHFRVRHTITIPNRGGPGAAEDWADFGFDMPDCKARKHSIKEEFTESDVH

>XP_028848396.1 tumor protein p73 isoform X1 [Denticeps clupeoides]

MSQSSVADEGPTFEHLWSSLEPDSTYFEMPAGSHAGSQGTSSSLPNNRAEVCMDVFHMRDMNDNVMSQYNLLSSSMEQGLGSRAASTSPFSSENASSVPTPSPYSQPNSTFDAMSPAPAIPSNADYPGPHNFEVTFQQSSTAKSATWTYSPILKKLYCQIAKTCPIQIKLSSPPPHGSGIRAMPIYKKAEHVTEVVKRCPNHELGRDFNDGQAAPASHLIRVEGNNMSQYVDDPVTGRQSVMVPYESPQVGTEFTTILYNFMCNSSCVGGMNRRPILIIITLETRDGQVLGRRSFEGRICACPGRDRKADEDHFREQQAVNETVAKNGNANKRNFKQTSPTTSGPSINIKKRRHGDEELFYIPVRGRENFDILMKIKDSLELMELVPQQLVESYRQQQQQLLHRQSHVASPSSYGCPLSNMNKLHSKLPPVNQLVGHQPQQTAGPSPSMAHMGANMLGGHHMQTNGDLNGGHPTQTMVSASHCTPPPPYNPDPSLVSFLTSLGCQNYIDYFASQGLQSIYHLQALSMEDLGTLKIPEQFRLAIWRGLQDMKQSHDYGQQLIRSSSNMAAMSIGPGGELQRQRVMEAVHFRVRHTITIPNRGGGPAPEEWADFGFDMPDCKTRRHSIKEEFMESDMH

>MBN3317952.1 P73 protein [Atractosteus spatula]

RVIYPSVDYALAKMAQSSSTDEGTTFEHLWSTLEPDSTYFDLPQNSHSGSNEASTSLPTDRAEICMDVFHMRDMNETVMSQYNLLSSTMDQNLGTRAASTSPYSSEHTSNVPTPSPYSQPNSTFDAMSPAPAIPSNTDYPGPHNFEVTFQQSSTAKSATWTYSPLLKKLYCQIAKTCPIQIKLSSPPPPGSLIRAMPVYKKAEHVTEVVKRCPNHELGRDFNDGQAAPASHLIRVEGNNLSQYVDDAVTGRQSVIVPYESPQVGTEFTTILYNFMCNSSCVGGMNRRPILIIITLETRDGQVLGRRSFEGRICACPGRDRKADEDHFREQQAMTENVAKNGNANKRTFKQTPPSIPGPGINTKKRRHGEEEIYYIPVRGRENFEILMKIKDSLELVELVPQQLVDSYRQQQQQLLQRQSHISSPSSYGSVLPNMNKVHGGISKLPSVNQLVGQQSQQSPNTVPNMGPMGSSMLNSHHMQTNGDINGGHSTQTMVSAPHCTPPPPYNPDPSLVSFLTSLGCQNCIEYFTSQGLQSMYHLQSLSMEDLGALKIPEQFRLVIWRGLQEMKQGHDYGQQLIRSSSNTSTMAIGPSGELQRQRVMEAVHFRVRHTITIPNRAGPAAAEDWADFGFDMPDCKARKHSIKEEFTDSDIN

>XP_035237252.1 tumor protein p73 isoform X1 [Anguilla anguilla]

MKDWRLLPLQRVICPSVDCPLAKMSQPSAADEGTTFEHLWSTLEPDSTYYELPPGGHRGDSEASSSLPSDRAEVCMDVFHMRDMNESVMSQYSLLSSSMDQTLAGRAPSTSPYSSEHASTVPTPSPYSQPNSTFDAMSPAPAIPSNTDYPGPHNFEVTFQQSSTAKSATWTYSPLLKKLYCQIAKTCPIQIKLASTPPHGSVIRAMPVYKKAEHVTEVVKRCPNHELGRDFNDGQAAPASHLIRVEGNNLSQYVDDPVTGRQSVMVPYESPQVGTEFTTVLYNFMCNSSCVGGMNRRPILIIITLETRDGQVLGRRSFEGRICACPGRDRKADEDHFREQQALNENVAKNGNANKRTFKQTPPHIPGPAINIKKRRHGEEELYYIPVRGRENFDILMKIKDSLELVELVPQQLVDSYRQQQQQLLQRQSHLSSPSSYGSPLPNMNKIHGGMNKLPSVNQLVGQQPQPSPGPPPGMGHMGSNMLNSHHMQSNGDMNGGHSGQPMVPASHCTPPPPYNPDPSLVSFLTSLGCQNCIDYFTSQGLQSVYHLQTLSMEDLGALKIPEQFRLAIWRGLQDMKQGHDYSQQLIRSSSNTATMAIGSTGELQRQRVMEAVHFRVRHTITIPNRAGPGGTGEDWSDFGFDMPDCKAQKQSIKEEFMDSDVH

>XP_028663285.1 tumor protein p73 isoform X1 [Erpetoichthys calabaricus]

MSQSSPADEGSTFEHLWSTLESDSTYFVLPPNGHSGTDEASTSLPSSRAEVCMDVYRMRGMNETIMSQYNLLSSGMDQNLSSRAASTSPYSSEHTSNVPTPSPYSQPNSTFDAMSPAPAIPSNTDYPGPHNFEVTFQQSSTAKSATWTYSPLLKKLYCQIAKTCPIQIKLSSPAPPGSVIRAMPVYKKAEHVTEVVKRCPNHELGRDFNDSQVAPASHLIRVEGNNLCQYIDDPVTGRQSVIVPYESPQVGTEFTTILYNFMCNSSCVGGMNRRPILIIITLETRDGQVLGRRSFEGRICACPGRDRKADEDHFREQQAMNDTVAKNGNANKRTFKQTPQNIPGPGITIKKRRHGEEEIYYIPVRGRENFEILMKIKDSLELVELVPQQLVDSYRQQQQQLLQRQNHMHSPSSYGPVMPNLNKVHGGINKLPSVNQLVGQTSQQSPNTATSMGPMGPSLLNSHHMHNGDMNMAHTTQSMVSASHCTPPPSYNPDPSLISFLSSLGCQNCVDYFTSQGLQSMYHLQNLTMEDLAILKIPEQYRLVIWRGLQELKQNHDYNGQQLIRSTSSNTSTMAIGPTGELQRQRVMEAVHFRVRHTITIPNRGGAGAGGSEDWSDFGFDMPDCKSRKQSIKEEFTEDFILNLGEDDKTIPLWTGHQ

>XP_039612035.1 tumor protein p73 isoform X1 [Polypterus senegalus]

MSQSSPADEGSTFEHLWNTLESDSTYFVLPPNGHSGNDEASTSLPSSRAEVCMDVYRMRGMNETIMSQYNLLSSSMDQNLSSRAASTSPYSSEHTSNVPTPSSYSQPNSTFDAMSPAPAIPSNTDYPGPHNFEVTFQQSSTAKSATWTYSPLLKKLYCQIAKTCPIQIKLSSPAPPGSVIRAMPVYKKAEHVTEVVKRCPNHELGRDFNDSQVAPASHLIRVEGNNLCQYIDDPVTGRQSVIVPYESPQVGTEFTTILYNFMCNSSCVGGMNRRPILIIITLETRELPLCSGQVLGRRSFEGRICACPGRDRKADEDHFREQQAMNDTVAKNGNANKRTFKQTPQNIPGPGITIKKRRHGEEEIYYIPVRGRENFEILMKIKDSLELVELVPQQLVDSYRQQQQQLLQRQNHMHSPSSYGPVMSNLNKVHGGINKLPSVNQLVGQTSQQSPNTATSMGPMGPSLINSHHMHNGDMNVAHTTQSMVSASHCTPPPSYNPDPSLISFLSSLGCQNCVDYFTSQGLQSMYHLQNLTMEDLAILKIPEQYRLVIWRALQELKQNHDYNSQQLIRSTSSNTSTMAIGPTGELQRQRVMEAVHFRVRHTITIPNRAGAGGSEDWSDFGFDMPDCKSRKQSIKEEFTEDSILNLGEDDKTMPLWTGQQ

>XP_041130403.1 tumor protein p73-like isoform X1 [Polyodon spathula]

MAQSSPADEGTTFEHLWSSLEPDSTYFDLSQNSHSGNNEAAASLPTNRAEVCMDVFQMRGMNETVMSQYNLLNSSMDQNLGSRAASASPYSSEHTSNVPTPSPYSQPNSTFDAMSPAPAIPSNTDYPGPHSFEVTFQQSSTAKSATWTYSPLLKKLYCQIAKTCPIQIKLSSLAPPGSVIRAMPVYKKAEHVTEVVKRCPNHELGRDFNDGQVAPASHLIRVEGNNLSQYVDDPVTGRQSVMVPYESPQVGTECTTILYNFMCNSSCVGGMNRRPILIIITLETRDGQVLGRRSFEGRICACPGRDRKADEDHFREQQAMNDSVNKNGNANKRTFKQTPPNIPGPGVNMKKRRHGEEEIYYIPVRGRENFEILMKIKDSLELVELVPQQMVDSYRQQQQQLLQRQNPVQPPTSYGSVLPNMNKMHGGINKLPSVNQLVGQQPQQNQSAAPNMGPMGPGMMNSHHMQQNGDMNGGHSAQSMVSASHCTPPPQYNPDPSLVSFLTSLGCQNCIEYFTSQGLQNLYHLQNLTMEDLVALKIPEQLRLVIWRGLQDLKQGHDYGGQQLIRSSSSNTSTMAIGPTGELQRQRVMEAVHFRVRHTITIPNRTSTGTSEDWPDFGFDVPDCKSRKQSIKEEFTENEIN

>XP_023697324.1 tumor protein p73 isoform X1 [Paramormyrops kingsleyae]

MKDCRWLPLQRVIYPSVDYPSAKMSQSTAADEGTTFEHLWSTLEPDSTYFELPQNSHPGSHDATSGTPSSRAEVCMDVFHMRDMNESVMSQYSLLSSSMEPSLGGRAASASPYSSEHASSAPAPSPYSQPNSTFDAMSPAPAIPSNADYPGPHGFEVTFQQSSTAKSATWTYSPLLKKLYCQIAKTCPIQIKLSTAPPPGSVIRAMPVYKKAEHVTEVVKRCPNHELGRDFNDGQVAPASHLIRVEGNNLCQYVDDAVTGRQSVLVPYESPQVGTEFTTILYNFMCNSSCVGGMNRRPILIIITLEMRDGQVLGRRSFEGRICACPGRDRKADEDHFREQQALNDNVAKNSNTSKRTFKQTPASIPGPAVNFKKRRHGEEEIYYIPVRGRENFDILMKIKDSLELVELVPQQLVESYRQQQQLLQRQSHVSAPPSSSYGSPMPNMNKLHGGIGKLPAVNHLVGQSQQSPGPLPGMTHMGSNMLNSHHMQANGDMNGGHSAQTMASASHCTPPPPYNPDPSLVSFLTSLGCQNCIDYFTSQGLQSVYHLQTLSMEDLGALKIPEQFRLAIWRGLQDMKQGHDYGQQLIRSSSNAGIGPSGELQRQRVMEAVHFRVRHTITIPNRAGTGEDWADFGFDMPDCKVRKHSIKEEFAESDIH

>XP_036434928.1 tumor protein p73 isoform X2 [Colossoma macropomum]

MEQGLGNRAASTSPYSSETASNVPTPSPYSQPNSTFEAMSPAPAIPSNTDYPGPHSFEVTFQQSSTAKSATWTYSPLLKKLYCQIAKTCPIQIKLASAPPHGSVVRAMPIYKKAEHVTEVVKRCPNHELGREFNESQSAPASHLIRVEGNNLCQYVDDPVTGRQSVLVPYEAPQVGTEFTTILYNFMCNSSCVGGMNRRPILIIITLETRDGQVLGRRSFEGRICACPGRDRKADEDHFREQQALNESVAKNGNANKRNFRQTPPNIPGPSINIKKRRHGEEEMYYIPVRGRENFDLLMKIKDSLELVELVPQQLVDSYRQQQQQLLHRQGHVTSPSSYGTSLSNMNKVHGNISKLPSVNQIVAHQTQQSAGPSSMAHMGANMLGGHHMQSNGDVNGAHSAQSMVSASHCTPPPPYNPDPSLVSFLTSLGCQNCIDYFTSQGLQSIYHLQTLSMEDLGALKIPEQFRLAIWRGLQDMKQSHDYGQQLIRSSSNMATMAIGPGGELQRQRVMEAVHFRVRHTITIPNRGGPTGGEEWADFGFDMPDCKTRKHSIKEEFAEGDVR

>RXM96242.1 Tumor protein p73 [Acipenser ruthenus]

MAQSSPADEGTTFEHLWSSLVIYPSVDYPTAKMAQSSPADEGTTFEHLWSSLEPDSTYFDLPPNSHSGNNEATASLSTNRSEVCMDVFQMRGMNETVMAMFAQCRPLEDVLLQAMFAQCRPLEDVLLQSQYNLLNSSMDQSLGSRAASASPYSSEHTSNVPTPSPYSQPNSTFDAMSPAPAIPSNTDYPGPHSFEVTFQQSSTAKSATWTYSPLLKKLYCQIAKTCPIQIKLSSLAPPGSVIRAMPVYKKAEHVTEVVKRCPNHELGRDFNDGQVAPASHLIRVEGNNLSQYIDDPVTGRQSVMVPYESPQVGTECTTILYNFMCNSSCVGGMNRRPILIIITLETRDGQVLGRRSFEGRICACPGRDRKADEDHFREQQAMNDSVNKNGNANKRTFKQTPPNIPGPSINMKKRRHGEEEIYYIPVRGRENFEILMKIKDSLELVELVPQQLVDSYRQQQQQLLQRQNPIQSPTSYGSVLPNMSKMHGGINKLPSVNQLVGQQPQQSQSAAPSMGPMGPGMMNSHHMQQNGDMNGGHSAQSMVSASHCTPPPQYNPDPSLVSFLSSLGCQNCIEYFTSQGLQNLYHLQNLTMEDLVALKIPEQLRLVIWRGLQDLKQGHDYGGQQLIRSSSSNTSTMAIGPTGELQRQRVMEAVHFRVRHTITIPNRTGTGTSEDWPDFGFDVPDCKSRKQSIKEEFTENEIN

>XP_019112267.1 tumor protein p73 isoform X1 [Larimichthys crocea]

MSQSTVAEEGGTFEHLWSSLEPDSTYFELPPGSQPGERPMPSTSTPGSHRNAAEGSMDVYHLRDMNDNVMSQYNLLSSSMESLGSRATSASPYSSENASSSAVPTPSPYSQPNSTFEGLSPAPAIPSNTDYPGLHSFQVSFQQSSTAKSATWTYSPLLKKLYCQIAKTCPIQIKLSSSPPHGSIIRAMPVYKKAEHVTEVVKRCPNHELGRDFNDGQTAPASHLIRVEGSNLCQYVDDPVTGRQSVFVPYEAPQVGTEFTTILYNFMCNSSCVGGMNRRPILIIITLETRDGQVLGRRSFEGRICACPGRDRKADEDHFREQQALNDSVVKNGGANKRNFKQSPPNIPSPNINMRKRRHGEEEIYYIPVRGRENFELLMKIKDSLELVELVPQSLVDSYRQQTQQQLLQRPSHIASPTSYSPLSNMNKLHAHGGLSKPPSVNQLVGQQPQQHATAPTSIAHMGSNMLNNHHMQANGDMNGGHSSQSIVSASHCSPPPYNPDPSLVSFLTSLGCQNFIEYFTSQGLQSIYHLQTLSMEDLAALKIPEQSRLAIWRGLQDMKQGSSLQLPAPTHHHDYHGQQLLRSSSNMAASAMAAIGATGGELQRQRVMEAVHFRVRHTITIPNRSGVVGTGGMAAATDEWADFGFDMPDCKLSRSKHSIKEEFMESDVH

>XP_036388640.1 tumor protein p73 [Megalops cyprinoides]

MYYVKKKSQYNLLSSSMDQSLASRAASTSPYSSEHTSNVPTPSPYSQPNSTFDAMSPAPAIPSNTDYPGPHNFEVTFQQSSTAKSATWTYSPLLKKLYCQIAKTCPIQIKLSSAPPHGSVIRAMPVYKKAEHVTEVVKRCPNHELGRDFNDGQAAPASHLIRVEGNNLAQYVDDPVTGRQSVMVPYESPQVGTEYTTVLYNFMCNSSCVGGMNRRPILIIITLETRDGQVLGRRSFEGRICACPGRDRKADEDHFREQQTLNENVAKNGNANKRTFKQTPPHIPGPGINIKKRRHGEEELYYIPVRGRENFDILMKIKDSLELVELVPQQLVDSYRQQQQQLLQRQSHIPSPSSYGSPLPNMNKIHGGINKLPSVNQLVGQQPQPSPGPAPSLAHMGSNMLNSHHMQTNGDINGGHTTQPMVSTSHCTPPPPYNPDPSLVSFLTSLGCQNCIDYFTSQGLQSVYHLQTLSMEDLGALKIPEQFRVAIWRGLQDMKQGHDYGQQLIRSSSNTATMAIGPSGELQRQRVMEAVHFRVRHTITIPNRAGPGTGEDWADFGFDMPDCKTRKHSIKEEFTESDVH

>XP_012696373.1 tumor protein p73 [Clupea harengus]

MKDWRWLPLQRVICPSVDCPSAKMSQSATADDGTTFENLWSSLEPDSTYYEMPPGGHPGGHHGDRGTPSNMPGNRAEVCMDVYHMRDMNDNVMSQYSLLSNSMESSLGPQRASSTSPFGSDNASSVPTPSPYAQPNSTFEAASPAPAIPSNTDYPGPHAFQVTFQQSSTAKSATWTYSPLLKKLYCQIAKTCPIQIKLASGPPPGGIIRAMPIYKKAEHVTEVVKRCPNHELGRDFNDGQAAPASHLIRVEGNNVAQYVDDPATGRQSVMVPYESPQVGTEFTTILYNFMCNSSCVGGMNRRPILIIITLETRDGQVLGRRSFEGRICACPGRDRKADEDHFREQQAINDSVNKNGNANKRNFKQTPATMPGPSLNMKKRRHGDEEMFYIPVRGRENFDILMKIKDSLELMTLVPQQVVDSYRQQQQELLQRQTPMASTSPYSSSLSNMNKLHAGINKLPSVNQLVGHHSQQGAGPSPSMGHMGGNMLSGHHMQTNGDMNGSHTQQPMVSASHCTPPPPYNPDPSLVSFLTSVGCQSCIDYFTSQGLQSIYHLQTLTMEDLIALKIPEQFRLAIWRGLQDMKQNHDYGQQLIRSSSNMSAATMAIGPSGELQRQRVMEAVHFRVRHTITIPNRGGAGNGVGVGVGAPHEDWADLGFDMPDCKSSRRHAAIKEEFTENHVH

>MBN3306744.1 P73 protein [Amia calva]

MDVFHMRDMNETVMSQYNLLSSSMDQSLGSRAASTSPYSSEHTSNVPTPSPYSQPNSTFDAMSPAPAIPSNTDYPGPHNFEVTFQQSSTAKSATWTYSPLLKKLYCQIAKTCPIQVKLSSVAPPGSVIRAMPVYKKAEHVTEVVKRCPNHELGRDFNDGQAAPASHLIRVEGNNLSQYVDDPVTGRQSVMVPYEPPQVGTEFTTILYNFMCNSSCVGGMNRRPILIIITLETRDGQVLGRRSFEGRICACPGRDRKADEDHFREQQAMNENVAKNGNANKRTFKQTPPNIPGPGINIKKRRQGEEEIYYIPVRGRENFEILMKIKDSLELVELVPQQLIDSYRQQQQQLLQRQNHVPSPSSYGSVLPNMNKIHGGINKLPSVNQLVGQQSQHSPGTAPNMGPMGSSMMNSHHMQTNGDVNGGHSGQPMVSASHCTPPPPYNPDPSLVSFLTSLGCQNCIDYFTSQGLQSMYHLQSLSMEDLSALKVPEQFRMAIWRGLQEMKQSHDYGQQLIRSASSNTATMAIGPSGELQRQRVMEAVHFRVRHTITIPNRAGPGPAEDWPDFGFDVPDCKRKHSIKEEFTDGDLN

>XP_028439150.1 tumor protein p73 isoform X1 [Perca flavescens]

MLSNQHASAVFCLLGNVVHYCVSLHVLVICPSPDYPSSKMSQSTVTEEGGTFEHLWSSLEPDSTYFELPPGSRPGERPVPSTSITGSHRSAAEVSMDVYQMRDINDNVMSQYNLLSSSMESLGSRATSASPYSSENASSSAVPTPSPYSQPNSTFEGLSPAPAIPSNTDYPGLHNFQVSFQQSSTAKSATWTYSPLLKKLYCQIAKTCPIQIKLSSSPPHGSIIRAMPVYKKAEHVTEVVKRCPNHELGRDFNDGQAAPASHLIRVEGNNLCQYVDDPVTGRQSVFLPYESPQVGTEFTTILYNFMCNSSCVGGMNRRPILIIITLETRDGQVLGRRSFEGRICACPGRDRKADEDHFREQQALNDSVAKNGSANKRNFKQSPPNIPSPNINMRKRRHGEEEIYYIPVRGRENFELLMKIKDSLELVELVPQPLVDSYRQQTQQQLLQRPNHIASPSSYSPLSNMNKLHAHGGLNKVPSVNQLVGQQPQQHHTAPTSMAHMGSNMLNSHHMQGNGDMNGGHSSQTIVSASHCSPPPPYNPDPSLVSFLTSLGCQNFIEYFTSQGLQSVYHLQTLSMEDLGALKIPEQFRLAIWRGLQDMKQVTPLQLPASTHHHDYHGQPLLRSSSNMTASAMAAIGAASGELQRQRVMEAVHFRVRHTITIPNRSGVVGTSGMAGATDEWADFGFDMPDCKLSRSKHSIKEEFMESDVH

>XP_035761614.1 tumor protein p73 isoform X1 [Neolamprologus brichardi]

MSQSTVTEEGGTFEHLWSSLEPDSTYFELPPGSQPGEQPVPSTSNPGNHHSTAEVSMDVYHMRDMNENVMSQYNLLSSSMESLGSRATSTSPYSSENASSSAVPTPSPYSQPNSTFEGLSPAPAIPSNTDYPGPHAFQVSFQQSSTAKSATWTYSPLLKKLYCQIAKTCPIQIKLSSSPPHGSIIRAMPIYKKAEHVTEVVKRCPNHELGRDFNDGQAAPASHLIRVEGNNLCQYVDDPVTGRQSVIVPYEAPQVGTEFTTILYNFMCNSSCVGGMNRRPILIIITLETRDGQVLGRRSFEGRICACPGRDRKADEDHFREQQALNESVAKNGSANKRNFKQSPTNIPSPNINMRKRRHGEEEVYYIPVRGRENFELLMKIKDSLELVELVPQQLVDSYRQQAQQQLLQRPSHVASPSSYSPLSNMNKLHSHGGLSKPPSVNQLAGQQPQQHHTAPSSMAHMGDYLCPNMLNSNHMQANGEMNGGHNSQNIVSASHCSPPPPYNPDPSLVSFLTSLGCQNFIEYFTSQGLQSIYHLQTLSMEDLGALKIPEQSRLIIWRGLQDMKQGASLQLPASSHHHDYHGQQLLRSSSNMTASAMAAIGAAGGELQRQRVMEAVHFRVRHTITIPNRSSVVGTSGMTAADEWADFGFDMPDCKVSRSKHSIKEEFMESDIH

>XP_004554165.1 tumor protein p73 isoform X2 [Maylandia zebra]

MSQSTVTEEGGTFEHLWSSLEPDSTYFELPPGSQPGEQPVPSTSNPGNHHSTAEVSMDVYHMRDMNENVMSQYNLLSSSMESLGSRATSTSPYSSENASSSAVPTPSPYSQPNSTFEGLSPAPAIPSNTDYPGPHAFQVSFQQSSTAKSATWTYSPLLKKLYCQIAKTCPIQIKLSSSPPHGSIIRAMPIYKKAEHVTEVVKRCPNHELGRDFNDGQAAPASHLIRVEGNNLCQYVDDPVTGRQSVIVPYEAPQVGTEFTTILYNFMCNSSCVGGMNRRPILIIITLETRDGQVLGRRSFEGRICACPGRDRKADEDHFREQQALNESVAKNGSANKRNFKQSPTNIPSPNINMRKRRHGEEEVYYIPVRGRENFELLMKIKDSLELVELVPQQLVDSYRQQAQQQLLQRPSHVASPSSYSPLSNMNKLHSHGGLSKPPSVNQLAGQQPQQHHTAPSSMAHMGDYLCPNMLNSNHMQANGEMNGGHNSQNIVSASHCSPPPPYNPDPSLVSFLTSLGCQNFIEYFTSQGLQSIYHLQTLSMEDLGALKIPEQSRLIIWRGLQDMKQGAPLQLPASSHHHDYHGQQLLRSSSNMTASAMAAIGAAGGELQRQRVMEAVHFRVRHTITIPNRSSVVGTSGMTAADEWADFGFDMPDCKVSRSKHSIKEEFMESDIH

>XP_005478336.1 tumor protein p73 isoform X1 [Oreochromis niloticus]

MSQSTVTEEGGTFEHLWSSLEPDSTYFELPPGSQPGEQPVPSTSNPGNHHSTAEVSMDVYHMRDMNENVMSQYNLLSSSMESLGSRATSTSPYSSENASSSAVPTPSPYSQPNSTFEGLSPAPAIPSNTDYPGPHAFQVSFQQSSTAKSATWTYSPLLKKLYCQIAKTCPIQIKLSSSPPHGSIIRAMPVYKKAEHVTEVVKRCPNHELGRDFNDGQAAPASHLIRVEGNNLCQYVDDPVTGRQSVIVPYEAPQVGTEFTTILYNFMCNSSCVGGMNRRPILIIITLETRDGQVLGRRSFEGRICACPGRDRKADEDHFREQQALNESVAKNGSANKRNFKQSPTNIPSPNINMRKRRHGEEEVYYIPVRGRENFELLMKIKDSLELVELVPQQLVDSYRQQAQQQLLQRPSHVASPSSYSPLSNMSKLHSHGGLSKPPSVNQLAGQQPQQHHTAPSSMAHMGDYLCPNMLNSNHMQANGEMNGGHNSQNIVSASHCSPPPPYNPDPSLVSFLTSLGCQNFIEYFTSQGLQSIYHLQTLSMEDLGALKIPEQSRLIIWRGLQDMKQGAPLQLPASSHHHDYHGQQLLRSSSNMTASAMAAIGAAGGELQRQRVMEAVHFRVRHTITIPNRSSVVGTSGMTAPDEWADFGFDMPDCKVSRSKHSIKEEFMESDIH

>XP_039656075.1 tumor protein p73 isoform X1 [Perca fluviatilis]

MKDWRWIPLQRVICPSPDYPSSKMSQSVVTEEGGTFEHLWSSLEPDSTYFELPPGSRPGERPVPSTSITGSHRSAAEVSMDVYQMRDINDNVMSQYNLLSSSMESLGSRATSASPYSSENASSSAVPTPSPYSQPNSTFEGLSPAPAIPSNTDYPGLHNFQVSFQQSSTAKSATWTYSPLLKKLYCQIAKTCPIQIKLSSSPPHGSIIRAMPVYKKAEHVTEVVKRCPNHELGRDFNDGQAAPASHLIRVEGNNLCQYVDDPVTGRQSVFLPYESPQVGTEFTTILYNFMCNSSCVGGMNRRPILIIITLETRDGQVLGRRSFEGRICACPGRDRKADEDHFREQQALNDSVAKNGSANKRNFKQSPPNIPSPNINMRKRRHGEEEIYYIPVRGRENFELLMKIKDSLELVELVPQPLVDSYRQQTQQQLLQRPNHIASPSSYSPLSNMNKLHAHGGLNKVPSVNQLVGQQPQQHHTAPTSMAHMGSNMLNSHHMQGNGDMNGGHSSQTIVSASHCSPPPPYNPDPSLVSFLTSLGCQNFIEYFTSQGLQSVYHLQTLSMEDLGALKIPEQFRLAIWRGLQDMKQVTPLQLPASSHHHDYHGQPLLRSSSNMTASAMAAIGAASGELQRQRVMEAVHFRVRHTITIPNRSGVVGTSGMAGATDEWADFGFDMPDCKLSRSKHSIKEEFMESDVH

>XP_039903683.1 tumor protein p73 isoform X1 [Simochromis diagramma]

MSQSTVTEEGGTFEHLWSSLEPDSTYFELPPGSQPGEQPVPSTSNPGNHHSTAEVSMDVYHMRDMNENVMSQYNLLSSSMESLGSRATSTSPYSSENASSSAVPTPSPYSQPNSTFEGLSPAPAIPSNTDYPGPHAFQVSFQQSSTAKSATWTYSPLLKKLYCQIAKTCPIQIKLSSSPPHGSIIRAMPIYKKAEHVTEVVKRCPNHELGRDFNDGQAAPASHLIRVEGNNLCQYVDDPVTGRQSVIVPYEAPQVGTEFTTILYNFMCNSSCVGGMNRRPILIIITLETRDGQVLGRRSFEGRICACPGRDRKADEDHFREQQALNESVAKNGSANKRNFKQSPTNIPSPNINMRKRRHGEEEVYYIPVRGRENFELLMKIKDSLELVELVPQQLVDSYRQQAQQQLLQRPSHVASPSSYSPLSNMNKLHSHGGLSKPPSVNQLTGQQPQQHHTAPSSMAHMGDYLCPNMLNSNHMQANGEMNGGHNSQNIVSASHCSPPPPYNPDPSLVSFLTSLGCQNFIEYFTSQGLQSIYHLQTLSMEDLGALKIPEQSRLIIWRGLQDMKQGAPLQLPASSHHHDYHGQQLLRSSSNMTASAMAAIGAAGGELQRQRVMEAVHFRVRHTITIPNRSSVVGTSGMTAADEWADFGFDMPDCKVSRSKHSIKEEFMESDIH

>XP_031142804.1 tumor protein p73 isoform X2 [Sander lucioperca]

MNVEMRVICPSPDYPSSKMSQSAVTEEGGTFEHLWSSLEPDSTYFELPPGSRPGERPVPSTSITGSHHSAAEVSMDVYQMRDINDNVMSQYNLLSSSMESLGSRATSASPYSSENASSSAVPTPSPYSQPNSTFEGLSPAPAIPSNTDYPGLHNFQVSFQQSSTAKSATWTYSPLLKKLYCQIAKTCPIQIKLSSSPPHGSIIRAMPVYKKAEHVTEVVKRCPNHELGRDFNDGQAAPASHLIRVEGNNLCQYLDDPVTGRQSVFLPYESPQVGTEFTTILYNFMCNSSCVGGMNRRPILIIITLETRDGQVLGRRSFEGRICACPGRDRKADEDHFREQQALNDSVAKNGSANKRNFKQSPPNIPSPNINMRKRRHGEEEIYYIPVRGRENFELLMKIKDSLELVELVPQPLVDSYRQQTQQQLLQRPNHIASPSSYSPLSNMNKLHAHGGLNKVPSVNQLVGQQPQQHHTAPTSIAHMGSNMLNSHHMQGNGDMNGGHSSQTIVSASHCSPPPPYNPDPSLVSFLTSLGCQNFIEYFTSQGLQSVYHLQTLSMEDLGALKIPEQFRLAIWRGLQDMKQVAPLQLPASTHHHDYHGQPLLRSSSNMTASAMAAIGAASGELQRQRVMEAVHFRVRHTITIPNRSGVVGTSGMTGATDEWADFGFDMPDCKLSRSKHSIKEEFMESDVH

>NXU44289.1 P73 protein [Drymodes brunneopygia]

VKMSQSSPADEGTTFEHLWSTLEPDSTYFDLPAATPSGSNEVSNRTEVTMDVFQMRGMTDSVMSQFNLLNNSMDQSIGSRAASTSPYSSEHTSNVPTHSPYSQPSSTFDAMSPAPVIPSNTDYPGPHHFEVTFQQSSTAKSATWTYSPLLKKLYCQIAKTCPIQIKVSTSPPPGTIIRAMPVYKKAEHVTEVVKRCPNHELGRDFNDGQAAPASHLIRVEGNNLSQYVDDPVTGRQSVMVPYEPPQVGTEFTTILYNFMCNSSCVGGMNRRPILIIITLETRDGQVLGRRSFEGRICACPGRDRKADEDHFREQQALNESAAKNGNANKRTFKQSPQGIPALGTGIKKRRHGEEEMYYVPVRGRENFEILMKIKESLELVELVPQQLVDSYRQQQQQLLQRQSQLQTPSSYGPVLSPMNKVHGGGINKLPSVNQLVGQPAQHGSSSAPSLGPMGAGMLNSHPMQPNGEMNGGHSSQSMVSGSHCTPPPPYNPDPSLVSFLTGLGCPNCIDYFTSQGLQNIYHLQNLSIEDLGALKIPEQYRMIIWRGLQELKQGHDYGAQQLIRSSSNASTISIGSSGELQRQRVMEAVHFRVRHTITIPNRGTADDWADFGFDLPDCKSRKQSIKEEFTEGEIN

>XP_040904398.1 tumor protein p73 isoform X2 [Toxotes jaculatrix]

MSQSTVTEEGGTFEHLWSSLEPDSTYFELPPGSQPGERPVPSTSTPGSHRSAAEVSMDVYHMRDMNDNVMSQYNLLSSSMESLGSRATSASPYSSENASSSAVPTPSPYSQPNSTFEGLSPAPAIPSNTDYPGPHTFQVSFQQSSTAKSATWTYSPLLKKLYCQIAKTCPIQIKLSSSPPHGSIIRAMPVYKKAEHVTEVVKRCPNHELGRDFNDGQAAPASHLIRVEGNNLCQYVDDPVTGRQSVFVPYEAPQVGTEFTTILYNFMCNSSCVGGMNRRPILIIITLETRDGQVLGRRSFEGRICACPGRDRKADEDHFREQQALNESVAKNGSASKRNFKQSPPNIPSPNINMRKRRHGEEEIYYIPVRGRENFELLMKIKDSLELVELVPQPLVDSYRQQTQQQLLQRPSHIASPSSYSPLSNMNKLHAHGGLSKPPSVNQLVGQQPQQHPTAPTSIAHMGSNMLHSHHMQANGDMNGGHSSQTIVSASHCSPPPPYNPDPSLVSFLTSLGCQNFIEYFTSQGLQSVYHLQTLSMEDLGALKIPEQSRLAIWRGLQDMKQGAPIQLPTSTHHHDYHGQQLLRSSSNMAASAMAAIGAAGGELQRQRVMEAVHFRVRHTITIPNRSGVVGTTGMAAAADEWADFGFDMPDCKMSRSKHSIKEEFMESDIH

>NWR59683.1 P73 protein [Bucorvus abyssinicus]

LKMSQSSPADEGTTFEHLWSTLEPDSTYFDLPPSNHTGDNEVSNRTEVTMDVFQMRSMNDSVMSQFNLLNNSMDQSIGSRAASTSPYSSEHTSNVPTHSPYSQPSSTFDAMSPAPVIPSNTDYPGPHHFEVTFQQSSTAKSATWTYSPLLKKLYCQIAKTCPIQIKVSTPPPPGTIIRAMPVYKKAEHVTEVVKRCPNHELGRDFNDGQSAPASHLIRVEGNNLSQYVDDPVTGRQSVMVPYEPPQVGTEFTTILYNFMCNSSCVGGMNRRPILIIITLETRDGQVLGRRSFEGRICACPGRDRKADEDHYREQQALNESAAKNGNANKRTFKQSPQGIPALGTGVKKRRHGEEEMYYVPVRGRENFEILMKIKESLELVELVPQQLVESYRQQQQQLLQRQSQLQAPSSYGPVLSPINKVHGGGINKLPSVNQLVGQPAQHSASSAPSLAPMGPGMLNSHPMQPNGEINGGHSSQSMVSGSHCTPPPPYNPDPSLVSFLTGLGCPNCIDYFTSQGLQNIYHLQNLSIEDLGALKIPEQYRMIIWRGLQELKQSHDYGAQQLIRSSSNASTISIGSSGELQRQRVMEAVHFRVRHTITIPNRGGADDWADFGFDLPDCKSRKQSIKEEFTEGEIN

>XP_026009455.1 tumor protein p73 isoform X1 [Astatotilapia calliptera]

MKDWHWIPFQRVVCPSPDYPSSKMSQSTVTEEGGTFEHLWSSLEPDSTYFELPPGSQPGEQPVPSTSNPGNHHSTAEVSMDVYHMRDMNENVMSQYNLLSSSMESLGSRATSTSPYSSENASSSAVPTPSPYSQPNSTFEGLSPAPAIPSNTDYPGPHAFQVSFQQSSTAKSATWTYSPLLKKLYCQIAKTCPIQIKLSSSPPHGSIIRAMPIYKKAEHVTEVVKRCPNHELGRDFNDGQAAPASHLIRVEGNNLCQYVDDPVTGRQSVIVPYEAPQVGTEFTTILYNFMCNSSCVGGMNRRPILIIITLETRDGQVLGRRSFEGRICACPGRDRKADEDHFREQQALNESVAKNGSANKRNFKQSPTNIPSPNINMRKRRHGEEEVYYIPVRGRENFELLMKIKDSLELVELVPQQLVDSYRQQAQQQLLQRPSHVASPSSYSPLSNMNKLHSHGGLSKPPSVNQLAGQQPQQHHTAPSSMAHMGDYLYPNMLNSNHMQANGEMNGGHNSQNIVSASHCSPPPPYNPDPSLVSFLTSLGCQNFIEYFTSQGLQSIYHLQTLSMEDLGALKIPEQSRLIIWRGLQDMKQGAPLQLPASSHHHDYHGQQLLRSSSNMTASAMAAIGAAGGELQRQRVMEAVHFRVRHTITIPNRSSVVGTSGMTAADEWADFGFDMPDCKVSRSKHSIKEEFMESDIH

**>NP_001279335.1 tumor protein p73 [Callorhinchus milii]**

MAQSSPADEGHTFENLWSSLEPDSTYFDIPQTNLSGNNEAVPNLPQNRTEVCMDVFQMRDMNESVMSQFNLLNSTMDQSISNRASSGSPYNSEHTSNVPTPSPYSQPSSTFDTMSPAPAIPSNTDYPGPHLFEVTFQQSSTAKSATWTYSPLLKKLYCQIAKTCPIQAKVTSLPPSGSVVRAMPVYKKAEHVTEVVKRCPNHELGRDFNDGQSAPASHLIRVEGNNLAQYVDDPVTGRQSVMVPYEPPQVGTEFTTILYNFMCNSSCVGGMNRRPILIIITLETRDGQVLGRRSFEGRICACPGRDRKADEDHYREQQAINENAAKNANGSKRPFKQTPPGAQGPTAGIKKRRHGDEEFFYVPVRGRENFEILMKIKESLELMELVPQQLIDSYRQQQQQQLLQRQQQASSAYGAVNPPMNKIPNVNKLPSVNQLVGQPSQHSPSTSVNLGPMGPTLMNNHHHHHHYMPPNGNMNGGNPSQSTAMGPTSHCTPPPPYNADPALVSFLTGLGCPNCLEYFTSQGLQTMYHLQNLSMEDLGALKIPEQYRLLIWRGLQEFKPGHDYNSPQLLRSSNNNPSVGGELQRQRVMEAVHFRVRHTITIPNRGDDWGDFGFDVPDCKTRKQPIKEEFTENELN

>XP_038677838.1 tumor protein p73 isoform X1 [Scyliorhinus canicula]

MAQSSPADEGPTFEHLWSSLEPDSTYFDIPQTNLSGSNEAVPNLPQNRTEVCMDVFQMRDMNESVMSQFNLLNSSMDQSITNRASSASPYNSEHASNVPTPSPYSQPSSTFDTMSPAPAIPSNTDYPGPHLFEVTFQQSSTAKSATWTYSPLLKKLYCQIAKTCPIQVKLTSPPPPGSVVRAMPVYKKAEHVTEVVKRCPNHELGRDFNDGQSAPASHLIRVEGNNLAQYIDDAVTGRQSVLVPFEPPQVGTEFTTILYNFMCNSSCVGGMNRRPILIIITLETRDGQVLGRRSFEGRICACPGRDRKADEDHYREQQAINESAAKNGNGNKRTFKQSPQGVQGPNAGIKKRRHGDEEFFYVPVRGRENFEILMKIKESLELMELVPQQLIDSYRQQQQQLLQRQQQASSSYGTVVPPTNKIPNMMNKLPSVNQLVGQPSQHSPSTSTNLGHMGPTIMNNHHHHHHHHIPTNGNMNGGNSSQSAGMGPTSHCTPPPPYTADPALVSFLTGLGCPNCLEYFTSQGLQTMYHLQNLSLEDLGALKIPEQYRLLIWRGLQEFKQGHDYSSQQLIRSNNNNTGTISGELQRQRVMEAVHFRVRHTITIPNRGDDWGDFGFDVPDCKTRKQPIKEEFTESELN

>XP_032904389.1 tumor protein p73 isoform X1 [Amblyraja radiata]

MAQSSPADEGPTFEHLWSSLEPDSTYFDIPQTNLSSNNEAVPNLPQNRTEVCMDVFQMRDMNESVMSQFNLLNSSMDQSITNRASSASPYNSEHTSNVPTPSPYSQPSSTFDTMSPAPAIPSNTDYPGPHLFEVTFQQSSTAKSATWTHSPLLKKLYCQIAKTCPIQVKLASPPPPGSVVRAMPVYKKAEHVTEVVKRCPNHELGRDFNDGQAAPASHLIRVEGNNLAQYVDDAVTGRQSVMVPFEPPQVGTEFTTILYNFMCNSSCVGGMNRRPILIIITLETRDGQVLGRRSFEGRICACPGRDRKADEDHYREQQAINDSAAKNGNGNKRTFKQTPQTTQGPNAGIKKRRHGDEEFFYIPVRGRENFEILMKIKESLELMELVPQQLVDSYRQQQQQMVQRQQQASSCYGTVIPPTNKVPNMMNKLPSVNQLVGQPSQHSPSTSASHGHIGPSIMNNHHHHHHHHIPTNGNMNGGNSSQPAGMGPASHCTPPPPYNADPGLVSFLTGLGCLNCLEYFTSQGLQTMYHLQNLSIEDLGALKIPDQYRLLIWRGLQEFKQGHDYSSQQLIRSGNNTATMSGELQRQRVMEAVHFRVRHTITIPNRADDWGDFGFDLPDCKTRKPPIKEEFTESELN

>XP_041061693.1 tumor protein p73 isoform X1 [Carcharodon carcharias]

MAQSSPADEGPTFEHLWSSLEPDSTYFDIPQTNLSGSNEAVPQNRTEVCMDVFHMRDMNESVMSQFNLLNSSMDQSITNRASSASPYNSEHASNVPTPSPYSQPSSTFDTMSPAPAIPSNTDYPGPHLFEVTFQQSSTAKSATWTYSPLLKKLYCQIAKTCPIQVKLTSPPPPGSVVRAMPVYKKAEHVTEVVKRCPNHELGRDFNDGQAAPASHLIRVEGNNLAQYIDDAVTGRQSVLVPFEPPQVGTEFTTILYNFMCNSSCVGGMNRRPILIIITLETRDGQVLGRRSFEGRICACPGRDRKADEDHYREQQAINESAAKNGNGNKRTFKQTPQGVQGPNAGIKKRRHGDEEFFYVPVRGRENFEILMKIKESLELMELVPQQLIDSYRQQQQQLLQRQQQASSSYGAVIPPTNKVPNMMNKLPSVNQLVGQPSQHSPSTSANLGHMGPTIMNNHHHHHHHHIPTNGNVNGGNSSQSAGMGPTSHCTPPPPYAADPALVSFLTGLGCPNCLEYFTSQGLQTMYHLQNLSLEDLGALKIPEQYRLLIWRGLQEFKQGHDYSSQQLIRSSNNTGTISGELQRQRVMEAVHFRVRHTITIPNRGDDWGDFGFDVPDCKTRKQPIKEEFTESELN

>NXM21824.1 P73 protein [Ploceus nigricollis]

VKMSQSSPADEGPTFEHLWSTLEPDSTYFDLPPANPSGSSEVSNSTEVTMDVFQMRGMTDSVMSQFNLLNNSMDQSIGSRAASTSPYSSEHTSNVPTHSPYSQPSSTFDAMSPAPVIPSNTDYPGPHHFEVTFQQSSTAKSATWTYSPLLKKLYCQIAKTCPIQIKVSTSPPPGTIIRAMPVYKKAEHVTEVVKRCPNHELGRDFNDGQSAPASHLIRVEGNNLSQYVDDPVTGRQSVMVPYEPPQVGTEFTTILYNFMCNSSCVGGMNRRPILIIITLETRDGQVLGRRSFEGRICACPGRDRKADEDHFREQQALNESTAKNGNANKRTFKQSPQGIPALGTGIKKRRHGEEEMYYVPVRGRENFEILMKIKESLELVELVPQQLVDSYRQQQQQQLLQRQSQLQTPSSYGPVLSPMNKVHGGGINKLPSVNQLVGQPAQHGSSSAPSLGPMGPGMLNSHPMQTNGEMNGGHSSQSMVSGSHCTPPPPYNADPSLVSFLTGLGCPNCIDYFTSQGLQNIYHLQNLSIEDLAALKIPEQYRMIIWRGLQELKQSHDYGAQQLIRSSSNASTISIGSSGELQRQRVMEAVHFRVRHTITIPNRGAADDWADFGFDLPDCKSRKQSIKEEFTEGEIN

>NXG54945.1 P73 protein [Hemiprocne comata]

VKMSQSSPADEGTTFEHLWSTLEPDSTYFDLPPSNHAGSNEVSNRTEVTMDVFQMRSMNDSVMSQFNLLNNSMDQSIGSRAASTSPYSSEHTSNVPTHSPYSQPSSTFDAMSPAPVIPSNTDYPGPHHFEVTFQQSSTAKSATWTYSPMLKKLYCQIAKTCPIQIKVSSPPPPGTIIRAMPVYKKAEHVTEVVKRCPNHELGRDFNDGQSAPASHLIRVEGNNLSQYVDDPVTGRQSVMVPYEPPQVGTEFTTILYNFMCNSSCVGGMNRRPILIIITLETRDGQVLGRRSFEGRICACPGRDRKADEDHYREQQALNESAAKNSNGNKRTFKQSPQGIPALGTGVKKRRHGEEEVYYVPVRGRENFEILMKIKESLELVELVPQQLVDSYRQQQQQLLQRQSQLQTPSSYGPVLSPMNKVHGGGINKLPSVNQLVGQPAQHSSSTTPGLGPMGPGMLNSHPMQPNGEMNGGHSSQSMVSGSHCTPPPPYNPDPSLVSFLTGLGCPNCIDYFTSQGLQNIYHLQNLSIEDLGALKIPEQYRMIIWRGLQELKQSHDYGAQQLIRSSSNASTISIGSSGELQRQRVMEAVHFRVRHTITIPNRADEWADFGFDLPDCKSRKQSIKEEFTEGEIN

>NXQ28607.1 P73 protein [Alaudala cheleensis]

VKMSQSSPADEGTTFEHLWSTLEPDSTYFDLPPANPTGSNEVSNRTEVTMDVFQMRGMTDSVMSQFNLLNNSMDQSIGSRAASTSPYSSEHTSNVPTHSPYSQPSSTFDAMSPAPVIPSNTDYPGPHHFEVTFQQSSTAKSATWTYSPLLKKLYCQIAKTCPIQIKVSTSPPPGTIIRAMPVYKKAEHVTEVVKRCPNHELGRDFNDGQSAPASHLIRVEGNNLSQYVDDPVTGRQSVMVPYEPPQVGTEFTTILYNFMCNSSCVGGMNRRPILIIITLETRDGQVLGRRSFEGRICACPGRDRKADEDHFREQQALNESTAKNGNANKRTFKQSPQGIPALGTGIKKRRHGEEEMYYVPVRGRENFEILMKIKESLELVELVPQQLVDSYRQQQQQLLQRQSQLQTPSSYGPVLSPMNKVHGGGINKLPSVNQLVGQPAQHSSSSAPNLGPMGPGMLNSHPMQSNGEMNGGHSSQSMVSGSHCTPPPPYSADPSLVSFLTGLGCPNCIDYFTSQGLQNIYHLQNLSIEDLGALKIPEQYRMIIWRGLQELKQGHDYGAQQLIRSSSNASSISIGSSGELQRQRVMEAVHFRVRHTITIPNRGAADDWADFGFDLPDCKSRKQSIKEEFTEGEIN

>NWT99035.1 P73 protein [Urocynchramus pylzowi]

VKMSQSSPADEGNTFEHLWSSLEPDSTYFDLPPANPSSSNEVSNRTEVTMDVFQMRGMTDSVMSQFNLLNNSMDQSIGSRAASTSPYSSEHTSNVPTHSPYSQPSSTFDAMSPAPVIPSNTDYPGPHHFEVTFQQSSTAKSATWTYSPLLKKLYCQIAKTCPIQIKVSTSPPPGTIIRAMPVYKKAEHVTEVVKRCPNHELGRDFNDGQSAPASHLIRVEGNNLSQYVDDPVTGRQSVMVPYEPPQVGTEFTTILYNFMCNSSCVGGMNRRPILIIITLETRDGQVLGRRSFEGRICACPGRDRKADEDHFREQQALNESTAKNGNANKRTFKQSPQGIPALGTGIKKRRHGEEEMYYVPVRGRENFEILMKIKESLELVELVPQQLVDSYRQQQQQLLQRQSQLQTSSSYGPVLSPMNKVHGGGINKLPSVNQLVGQPAQHGSSSAPSLGPMGPGMLNSHPMQTNGEMNGGHSSQSMVSGSHCTPPPPYSADPSLVSFLTGLGCPNCIDYFTSQGLQNIYHLQNLSIEDLAALKIPEQYRMIIWRGLQELKQSHDYGAQQLIRSSSSNASTISIGSSGELQRQRVMEAVHFRVRHTITIPNRGAADDWADFGFDLPDCKSRKQSIKEEFTEGEIN

>NWH90318.1 P73 protein [Aegithalos caudatus]

VKMSQSSPADEGTTFEHLWSTLEPDSTYFDLPAANPAGSNEVSNSTEVTMDVFQMRGIPDSVMSQFNLLNNSMDQSIGSRAASTSPYSSEHTSNVPTHSPYSQPSSTFDAMSPAPVIPSNTDYPGPHHFEVTFQQSSTAKSATWTYSPLLKKLYCQIAKTCPIQIKVSSSPPPGTIIRAMPVYKKAEHVTEVVKRCPNHELGRDFNDGQSAPASHLIRVEGNNLSQYVDDPVTGRQSVMVPYEPPQVGTEFTTILYNFMCNSSCVGGMNRRPILIIITLETRDGQVLGRRSFEGRICACPGRDRKADEDHFREQQALNESTAKNGNANKRTFKQSPQGIPALGTGIKKRRHGEEEMYYVPVRGRENFEILMKIKESLELVELVPQQLVDSYRQQQQQLLQRQSQLQTPSSYGPVLSPMNKVHAGGINKLPSVNQLVGQPAQHSSSSAPSLGPMGPGMLNSHPMQSNGEMNGGHSSQSMVSGSHCTPPPPYNADPSLVSFLTGLGCPNCIDYFTSQGLQNIYHLQNLSIEDLGALKIPEQYRMLIWRGLQDLKQSHDYGAQQLIRSSSNASSISIGSSGELQRQRVMEAVHFRVRHTITIPNRGTADDWADFGFDLPDCKSRKQSIKEEFTEGEIN

>NWS00988.1 P73 protein [Motacilla alba]

VKMSQSSPADEGPTFEHLWSTLEPDSTYFDLPPANPSSSNEVSNSTEVTMDVFQMRGMTDSVMSQFNLLNNSMDQSIGSRAASTSPYSSEHTSNVPTHSPYSQPSSTFDAMSPAPVIPSNTDYPGPHHFEVTFQQSSTAKSATWTYSPLLKKLYCQIAKTCPIQIKVSTSPPPGTIIRAMPVYKKAEHVTEVVKRCPNHELGRDFNDGQSAPASHLIRVEGNNLSQYVDDPVTGRQSVMVPYEPPQVGTEFTTILYNFMCNSSCVGGMNRRPILIIITLETRDGQVLGRRSFEGRICACPGRDRKADEDHFREQQALNESTAKNGNANKRTFKQSPQGIPALGTGIKKRRHGEEEMYYVPVRGRENFEILMKIKESLELVELVPQQLVDSYRQQQQQLLQRQSQLQTPSSYGPVLSPMNKVHGGGINKLPSVNQLVGQPAQHGSSSAPSLGPMGPGMLNSHPMQTNGEMNGGHSSQSMVSGSHCTPPPPYNADPSLVSFLTGLGCPNCIDYFTSQGLQNIYHLQNLSIEDLAALKIPEQYRMVIWRGLQELKQGHDYGAQQLIRSSSSNASTISIGSSGELQRQRVMEAVHFRVRHTITIPNRGAADDWADFGFDLPDCKSRKQSIKEEFTEGEIN

>NWT86628.1 P73 protein [Lanius ludovicianus]

LKMSQSSPADEGTTFEHLWSTLEPDSTYFDLPPANPTSSNEVSNRTEVTMDVFQMRGMTDSVMSQFNLLNNSMDQSIGSRAASTSPYSSEHTSNVPTHSPYSQPSSTFDAMSPAPVIPSNTDYPGPHHFEVTFQQSSTAKSATWTYSPLLKKLYCQIAKTCPIQIKVSTSPPPGTIIRAMPVYKKAEHVTEVVKRCPNHELGRDFNDGQSAPASHLIRVEGNNLSQYVDDPVTGRQSVMVPYEPPQVGTEFTTILYNFMCNSSCVGGMNRRPILIIITLETRDGQVLGRRSFEGRICACPGRDRKADEDHFREQQALNESAAKNGNANKRTFKQSPQGIQALGTGIKKRRHGEEEMYYVPVRGRENFEILMKIKESLELVELVPQQLVDSYRQQQQQLLQRQSQLQTPSSYGPVLSPMNKVHGGGINKLPSVNQLVGQPAQHSSSSAPSLGPMGPGMLNSHPMQPNGEMNGGHSSQSMVSGSHCTPPPPYNPDPSLVSFLTGLGCPNCIDYFTSQGLQNIYHLQNLSIEDLGALKIPEQYRMIIWRGLQELKQSHDYGAQQLIRSSSNASTISIGSSGELQRQRVMEAVHFRVRHTITIPNRGAADDWADFGFDLPDCKSRKQSIKEEFTEGEIN

>NWS43798.1 P73 protein [Probosciger aterrimus]

VKMSQSSPADEGTTFEHLWSTLEPDSTYFDLPPSNHTGSNEVSNRTEVTMDIFQMRNMNDSVMSQFNLLNNSMDQSIGSRAASTSPYSSEHTSNVPTHSPYSQPSSTFDAMSPAPVIPSNTDYPGPHHFEVTFQQSSTAKSATWTYSPLLKKLYCQIAKTCPIQIKVSTPPPPGTIIRAMPVYKKAEHVTEVVKRCPNHELGRDFNDGQAAPASHLIRVEGNNLSQYVDDPVTGRQSVMVPYEPPQVGTEFTTILYNFMCNSSCVGGMNRRPILIIITLETRDGQVLGRRSFEGRICACPGRDRKADEDHYREQQALNDSAAKNGSTNKRTFKQSPQGIPALGAGIKKRRHGEEEMYYVPVRGRENFEILMKIKESLELVELVPQQLVDSYRQQQQQLLQRQTQLQTSSSYGPVLSPMNKVHSGGINKLPSVNQLVGQPAQHGASSAPGLAPMGPGMLNSHPMQPNGEMNGGHASQSMVSGSHCTPPPPYNPDPSLVSFLTGLGCPNCIDYFTSQGLQSIYHLQNLSIEDLGALKIPEQYRMVIWRGLQELKQSHDYGAQQLLRSSSNASTISIGSSGELQRQRVMEAVHFRVRHTITIPNRGAADDWADFGFDLPDCKSRKQSIKEEFTEGEIN

>NXY61793.1 P73 protein [Callaeas wilsoni]

VKMSQSSPADEGTTFEHLWSTLEPDSTYFDLPPANPTGSNEVSNRTEVTMDVFQMRGMTDSVMSQFNLLNNSMDQSIGSRAASTSPYSSEHTSNVPTHSPYSQPSSTFDAMSPAPVIPSNTDYPGPHHFEVTFQQSSTAKSATWTYSPLLKKLYCQIAKTCPIQIKVSTSPPPGTIIRAMPVYKKAEHVTEVVKRCPNHELGRDFNDGQSAPASHLIRVEGNNLSQYVDDPVTGRQSVMVPYEPPQVGTEFTTILYNFMCNSSCVGGMNRRPILIIITLETRDGQVLGRRSFEGRICACPGRDRKADEDHFREQQALNESAAKNGSANKRTFKQTPQGIPALGTGIKKRRHGEEEMYYVPVRGRENFEILMKIKESLELVELVPQQLVDSYRQQQQQLLQRQSQLQTPSSYGPVLSPMNKVHSGGINKLPSVNQLVGQPAQHSSSSAPSLGPMGPGMLNSHPMQPNGEMNGGHSSQSMVSGSHCTPPPPYNPDPSLVSFLTGLGCPNCIDYFTSQGLQNIYHLQNLSIEDLGALKIPEQYRMIIWRGLQELKQSHDYGAQQLIRSSSNASTISIGSSGELQRQRVMEAVHFRVRHTITIPNRGAADDWADFGFDLPDCKSRKQSIKEEFTEGEIN

>XP_030364026.1 tumor protein p73 isoform X1 [Strigops habroptila]

MSQSSPADEGTTFEHLWSTLEPDSTYFDLPPSNHTGSNEVSNRTEVTMDVFQMRSMNDSVMSQFNLLNNSMDQSIGSRAASTSPYSSEHTSNVPTHSPYSQPSSTFDAMSPAPVIPSNTDYPGPHHFEVTFQQSSTAKSATWTYSPLLKKLYCQIAKTCPIQIKVSTPPPPGTIIRAMPVYKKAEHVTEVVKRCPNHELGRDFNDGQAAPASHLIRVEGNNLSQYVDDPVTGRQSVMVPYEPPQVGTEFTTILYNFMCNSSCVGGMNRRPILIIITLETRDGQVLGRRSFEGRICACPGRDRKADEDHYREQQALNESAAKNGSTNKRTFKQSPQGIPALGAGIKKRRHGEEEMYYVPVRGRENFEILMKIKESLELVELVPQQLVDSYRQQQQQLLQRQTQLQPPSSYGPVLSPMNKVHSAGINKLPSVNQLVGQPAQHSASSAPGLAPMGPGMLNSHPMQPNGEMNGGHASQSMVSGSHCTPPPPYNPDPSLVSFLTGLGCPNCIDYFTSQGLQNIYHLQNLSIEDLGALKIPEQYRMVIWRGLQELKQSHDYGAQQLLRSSSNASTISIGSSGELQRQRVMEAVHFRVRHTITIPNRGAADDWADFGFDLPDCKSRKQSIKEEFTEGEIN

>NXA88390.1 P73 protein [Melanocharis versteri]

VKMSQPSPADEGTTFEHLWGALEPDSTYFDLPAANPTGSNEVSNRTEVTMDVFQMRGMTDTVMSQFNLLNNSMDQSIGSRAASTSPYSSEHTSNVPTHSPYSQPSSTFDAMSPAPVIPSNTDYPGPHHFEVTFQQSSTAKSATWTYSPLLKKLYCQIAKTCPIQIKVSTSPPPGTIIRAMPVYKKAEHVTEVVKRCPNHELGRDFNDGQSAPASHLIRVEGNNLSQYVDDPVTGRQSVMVPYEPPQVGTEFTTILYNFMCNSSCVGGMNRRPILIIITLETRDGQVLGRRSFEGRICACPGRDRKADEDHFREQQALNESAAKNGNANKRTFKQSPQGIPALGAGIKKRRHGDEEMYYVPVRGRENFEILMKIKESLELVELVPQQLVDSYRQQQQQLXSQLQTPSSYGPVLSPMNKVHGGGINKLPSVNQLVGQPAQHGSSSAPSLGPMGPGMLNSHPMQPNGEMNGGHSSQSMVSGSHCTPPPPYNPDPSLVSFLTGLGCPNCIDYFTSQGLQNIYHLQNLSIEDLGALKIPEQYRMIIWRGLQELKQSHDYGAQQLIRSSSNASTISIGSSGELQRQRVMEAVHFRVRHTITIPNRGAADDWADFGFDLPDCKSRKQSIKEEFTEGEIN

>NXK63246.1 P73 protein [Sylvietta virens]

VKMSQSPPADEGTTFEHLWSTLEPDSTYFDLPPANPSGSNEVSNRTEVTMDVFQMRGMADSVMSQFNLLNNSMDQSIGSRAASTSPYSSEHTSNVPTHSPYSQPSSTFDAMSPAPVIPSNTDYPGPHHFEVTFQQSSTAKSATWTYSPLLKKLYCQIAKTCPIQIKVSTSPPPGTIIRAMPVYKKAEHVTEVVKRCPNHELGRDFNDGQSAPASHLIRVEGNNLSQYVDDPVTGRQSVMVPYEPPQVGTEFTTILYNFMCNSSCVGGMNRRPILIIITLETRDGQVLGRRSFEGRICACPGRDRKADEDHFREQQALNESTAKNGNANKRTFKQSPQGIPALGTGIKKRRHGEEEMYYVPVRGRENFEILMKIKESLELVELVPQQLVDSYRQQQQQLLQRQSQLQTPSSYGPVLSPMNKVHGGGINKLPSVNQLVGQPAQHGSSSAPSLGPMGPGMLNSHPMQANGEMNGGHSSQSLVSGSHCTPPPPYSADPSLVSFLTGLGCPNCIDYFTSQGLQNIYHLQNLSIEDLGALKIPEQYRMVIWRGLQELKQSHDYGAQQLIRSSSNASSISIGSSGELQRQRVMEAVHFRVRHTITIPNRGAPDDWADFGFDLPDCKSRKQSIKEEFTEGEIN

>NXW42424.1 P73 protein [Nyctiprogne leucopyga]

VKMSQSSPDDEGTTFEHLWSTLEPDSTYFNLPPSNHAGSNEVSNRTEVTMDIFQMRSMNDSVMSQFNLLNNSMDQSIGSRAASTSPYSSEHTSNVPTHSPYSQPSSTFDAMSPAPVIPSNTDYPGPHHFEVTFQQSSTAKSATWTYSPLLKKLYCQIAKT

CPIQIKVSTPPPPGTIIRAMPVYKKAEHVTEVVKRCPNHELGRDFNDGQSAPASHLIRVEGNNLSQYVDDPVTGRQSVMVPYEPPQVGTEFTTILYNFMCNSSCVGGMNRRPILIIITLETRDGQVLGRRSFEGRICACPGRDRKADEDHYREQQALNESAAKNGNGTKRAFKQSPQGIPALGAGVKKRRHGEEEIYYVPVRGRENFEILMKIKESLELVELVPQQLVDSYRQQQQQLLQRQSQLQTPSSYGPVLSPMNKVHGGGINKLPSVNQLVGQPVQHSSSSTPGLGPMGPGMLNSHPMQPNGEMNGGHSSQPMVSGSHCTPPPPYNPDPSLVSFLTGLGCPNCIDYFTSQGLQNIYHLQNLSIEDLGALKIPEQYRMIIWRGLQELKQSHDYGAQQLIRSSSNASTISIGSSGELQRQRVMEAVHFRVRHTITIPNRGTADDWADFGFDLPDCKSRKQSIKEEFTEGEIN

>NXG99898.1 P73 protein [Loxia leucoptera]

VKMSQPSPADEGPTFEHLWSSLEPDSTYFDLPPANPSSSNEVSNSTEVTMDVFQMRGMTDSVMSQFNLLNNSMDQSIGSRAASTSPYSSEHTSNVPTHSPYSQPSSTFDAMSPAPVIPSNTDYPGPHHFEVTFQQSSTAKSATWTYSPLLKKLYCQIAKTCPIQIKVSTSPPPGTIIRAMPVYKKAEHVTEVVKRCPNHELGRDFNDGQSAPASHLIRVEGNNLSQYVDDPVTGRQSVMVPYEPPQVGTEFTTILYNFMCNSSCVGGMNRRPILIIITLETRDGQVLGRRSFEGRICACPGRDRKADEDHFREQQALNESTAKNGNANKRTFKQSPQGIPALGTGIKKRRHGEEEMYYVPVRGRENFEILMKIKESLELVELVPQQLVDSYRQQQQQQLHQRQSQLQTPSSYGPVLSPMNKVHGGGINKLPSVNQLVGQPAQHGSSSAPSLGPMGPGMLNSHSMQTNGEMNGGHSSQSMVSGSHCTPPPPYNADPSLVSFLTGLGCPNCIDYFTSQGLQNIYHLQNLSIEDLAALKIPEQYRMIIWRGLQELKQSHDYGAQQLIRSSSSNASTISIGSSGELQRQRVMEAVHFRVRHTITIPNRGTADDWADFGFDLPDCKSRKQSIKEEFTEGEIN

>NXI45420.1 P73 protein [Galbula dea]

VKMSQSSPADEGTTFEHLWSTLEPDSTYFDLPPSNHTGGNEVSDRTEVTMDVFQMRSMNDSVMSQFNLLNNSMDQSIGSRAASTSPYSSEHTSNVPTHSPYSQPSSTFDAMSPAPVIPSNTDYPGPHHFEVTFQQSSTAKSATWTYSPLLKKLYCQIAKTCPIQIKVSTPPPPGTIIRAMPVYKKAEHVTEVVKRCPNHELGRDFNDGQSAPASHLIRVEGNNLSQYVDDPVTGRQSVMVPYEPPQVGTEFTTILYNFMCNSSCVGGMNRRPILIIITLETRDGQVLGRRSFEGRICACPGRDRKADEDHYREQQALNESAAKNGNGNKRTFKQSPQGIPALGAGVKKRRHGEEEMYYVPVRGRENFEILMKIKESLELVELVPQQLVDSYRQQQQQLLQRQSQMQTPSSYGPVVSPMNKVHSGGINKLPSVNQLVGQPAQHSSSSAPSLGPMGAGMLNSHPMQPNGEMNGGHSSQSMVSGSHCTPPPPYNPDPSLVSFLTGLGCPNCIDYFTSQGLQNIYHLQNLSIEDLGALKIPEQYRMIIWRGLQELKQSHDYGAQQLIRSSSNASTISIGSSGELQRQRVMEAVHFRVRHTITIPNPGRGAADEWADFGFDLPDCKARKQAIKEEFTEGEIN

>NXK36285.1 P73 protein [Piprites chloris]

KMSQSSPADEGNTFAHLWSTLEPDSTYFDLPPATHTGSNEVSNQTEVTMDVFQMRGMNDSVMSQFNLLNNSMDQSIGSRAASTSPYSSEHTSNVPTHSPYSQPSSTFDAMSPAPVIPSNTDYPGPHHFEVTFQQSSTAKSATWTYSPLLKKLYCQIAKTCPIQIKVSTPPPPGTIIRAMPVYKKAEHVTEVVKRCPNHELGRDFNDGQSAPASHLIRVEGNNLSQYVDDPVTGRQSVMVPYEPPQVGTEFTTILYNFMCNSSCVGGMNRRPILIIITLETRDGQVLGRRSFEGRICACPGRDRKADEDHFREQQALNESAAKNGNASKRTFKQSPQSIPALGTGIKKRRHGEEEMYYVPVRGRENFEILMKIKESLELVELVPQQLVDSYRQQQQQLLQRQSQLQAPSSYGPVLSPMNKVHSGGINKLPSVNQLVGQPAQHGPSAAPSLGPMGPGMLNSHPMQPNGEMNGGHSSQSMVSGSHCTPPPPYNPDPSLVSFLTGLGCPNCIDYFTSQGLQNIYHLQNLSIEDLGALKIPEQYRMIIWRGLQELKQSHDYGAQQLIRSSSNASTISIGSSGELQRQRVMEAVHFRVRHTITIPNRGTADDWADFGFDLPDCKSRKQSIKEEFTEGEIN

>NXB01091.1 P73 protein [Cnemophilus loriae]

VKMSQSSPADEGTTFEHLWSTLEPDSTYFDLPPANPTGSNEVSNRTEVTMDVFQIRGMTDSVMSQFNLLNNSMDQSIGSRAASTSPYSSEHTSNVPTHSPYSQPSSTFDAMSPAPVIPSNTDYPGPHHFEVTFQQSSTAKSATWTYSPLLKKLYCQIAKTCPIQIKVSTSPPPGTIIRAMPVYKKAEHVTEVVKRCPNHELGRDFNDGQSAPASHLIRVEGNNLSQYVDDPVTGRQSVMVPYEPPQVGTEFTTILYNFMCNSSCVGGMNRRPILIIITLETRDGQVLGRRSFEGRICACPGRDRKADEDHFREQQALNESAAKNGNANKRTFKQSPQGIPALGTGVKKRRHGEEEMYYVPVRGRENFEILMKIKESLELVELVPQQLVDSYRQQQQQLLQRQSQLQTPSSYGPVLSPMNKVHGGGINKLPSVNQLVGQPAQHGSSSAPSLGPMGPGMLNSHPMQPNGEMNGGHSSQSMVSGSHCTPPPPYNPDPSLVSFLTGLGCPNCIDYFTSQGLQNIYHLQNLSIEDLGALKIPEQYRMIIWRGLQELKQSHDYGAQQLIRSSSNASTISIGSSGELQRQRVMEAVHFRVRHTITIPNRGTADDWADFGFDLPDCKSRKQSIKEEFTEGEIN

>NWV09353.1 P73 protein [Ptilonorhynchus violaceus]

LKMSQSSPADEGTTFEHLWSTLEPDSTYFDLPPANPTGSNEVSNRTEVTMDVFQMRGMTDSVMSQFNLLNNSMDQSIGSRAASTSPYSSEHTSNVPTHSPYSQPSSTFDAMSPAPVIPSNTDYPGPHHFEVTFQQSSTAKSATWTYSPLLKKLYCQIAKTCPIQIKVSTSPPPGTIIRAMPVYKKAEHVTEVVKRCPNHELGRDFNDGQSAPASHLIRVEGNNLSQYVDDPVTGRQSVMVPYEPPQVGTEFTTILYNFMCNSSCVGGMNRRPILIIITLETRDGQVLGRRSFEGRICACPGRDRKADEDHFREQQALNESAAKNGSANKRTFKQSPQGIPALGTGVKKRRHGEEEMYYVPVRGRENFEILMKIKESLELVELVPQQLVDSYRQQQQQLLQRQSQLQTPSSYGPVLSPMNKVHGGGINKLPSVNQLVGQPAQHGSSSTPSLGPMGPGMLNSHPMQPNGEMNGGHSSQSMVSGSHCTPPPPYNPDPSLVSFLTGLGCPNCIDYFTSQGLQNIYHLQNLSIEDLGALKIPEQYRMIIWRGLQELKQSHDYGAQQLIRSSSNASTISIGSSGELQRQRVMEAVHFRVRHTITIPNRGAADDWADFGFDLPDCKSRKQSIKEEFTEGEIN

>NWZ85036.1 P73 protein [Poecile atricapillus]

VKMSQSSPADEGTTFEHLWSTLEPDSTYFDLPPANPTGSNEVSNRTEVTMDVFQMRGMTDSVMSQFNLLNNSMDQSIGSRAASTSPYSSEHTSNVPTHSPYSQPSSTFDAMSPAPVIPSNTDYPGPHHFEVTFQQSSTAKSATWTYSPLLKKLYCQIAKTCPIQIKVSTSPPPGTIIRAMPVYKKAEHVTEVVKRCPNHELGRDFNDGQSAPASHLIRVEGNNLSQYVDDPVTGRQSVMVPYEPPQVGTEFTTILYNFMCNSSCVGGMNRRPILIIITLETRDGQVLGRRSFEGRICACPGRDRKADEDHFREQQALNESTAKNGNANKRTFKQSPQGIPALGTGIKKRRHGEEEMYYVPVRGRENFEILMKIKESLELVELVPQQLVDSYRQQQQQLLQRQSQLQTPSSYGPVLSPMNKVHGGGINKLPSVNQLVGQPAQHSSSSAPSLGPMGPGMLNSHPMQTNGEMNGGHSSQSMVSGSHCTPPPPYNPDPSLVSFLTGLGCPNCIDYFTSQGLQNIYHLQNLSIEDLGALKIPEQYRMIIWRGLQELKQSHDYGAQQLIRSSSNASTISIGSSGELQRQRVMEAVHFRVRHTITIPNRGAADDWADFGFDLPDCKSRKQSIKEEFTEGEIN

>NWV27813.1 P73 protein [Origma solitaria]

VKMSQSSPADEGTTFEHLWSTLEPDSTYFDLPPANPTVSNEVSNRTEVTMDVFQMRGMTDSVMSQFNLLNNSMDQSIGSRAASTSPYSSEHTSNVPTHSPYSQPSSTFDTMSPAPVIPSNTDYPGPHHFEVTFQQSSTAKSATWTYSPLLKKLYCQIAKTCPIQIKVSTSPPPGTIIRAMPVYKKAEHVTEVVKRCPNHELGRDFNDGQSAPASHLIRVEGNNLSQYVDDPVTGRQSVMVPYEPPQVGTEFTTILYNFMCNSSCVGGMNRRPILIIITLETRDGQVLGRRSFEGRICACPGRDRKADEDHFREQQALNESAAKNGNANKRTFKQSPQGIPALGTGIKKRRHGEEEMYYVPVRGRENFEILMKIKESLELVELVPQQLVDSYRQQQQQLLQRQSQLQTPSSYGPVLSPMNKVHSGGINKLPSVNQLVGQPAQHGSGSAPSLGPMGPGMLNSHPMQPNGEMNGGHSSQSMVSGSHCTPPPPYNPDPSLVSFLTGLGCPNCIDYFTSQGLQNIYHLQNLSIEDLGALKIPEQYRMIIWRGLQELKQSHDYGAQQLIRSSSNASTISIGSSGELQRQRVMEAVHFRVRHTITIPNRGAADDWADFGFDLPDCKSRKQSIKEEFTEGEIN
