# Supplementary material for: Phylogenetic analysis of the MCL1 BH3 binding groove and rBH3 sequence motifs in the p53 and INK4 protein families
Source: PLoS One. 2023 Jan 25;18(1):e0277726. doi: 10.1371/journal.pone.0277726 (PMC9876281; doi:10.1371/journal.pone.0277726)
Supplement: S6 File — A total of 316 p15 or p16 sequences were used to generate the INK4 family phylogenetic tree. (DOCX) [file pone.0277726.s010.docx]

**>NP_000068.1 cyclin-dependent kinase inhibitor 2A isoform p16INK4a [Homo sapiens]**

MEPAAGSSMEPSADWLATAAARGRVEEVRALLEAGALPNAPNSYGRRPIQVMMMGSARVAELLLLHGAEPNCADPATLTRPVHDAAREGFLDTLVVLHRAGARLDVRDAWGRLPVDLAEELGHRDVARYLRAAAGGTRGSNHARIDAAEGPSDIPD

>NP_001139762.1 cyclin-dependent kinase inhibitor 2A [Pan troglodytes]

MEPAAGSSMEPSADWLATAAARGRVEEVRALLEAGALPNAPNSYGRRPIQVMMMGSARVAELLLLHGAEPNCADPATLTRPVHDAAREGFLDTLVVLHRAGARLDVRDAWGRLPVDLAEELGHRDVARYLRAAAGGTRGSNHARIDAAEGPLDIPD

>XP_004047926.2 cyclin-dependent kinase inhibitor 2A isoform X1 [Gorilla gorilla gorilla]

MELAAGSSMEPSADWLATAAARGRVEEVRALLEAGALPNAPNSYGRRPIQVMMMGSARVAELLLLHGAEPNCADPATLTRPVHDAAREGFLDTLVVLHRAGARLDVRDAWGRLPVDLAEELGHRDVARYLRAAAGGTRGSNHARIDAAEGPSDIPD

>XP_009242657.1 cyclin-dependent kinase inhibitor 2A [Pongo abelii]

MEPSADWLATAAARGRVDEVRALLEAGALPNAPNSYGRRPIQVMMMGSARVAELLLLHGAEPNCADPATLTRPVHDAAREGFLDTLAVLHRAGARLDVRDAWGRLPVDLAEELGHRDVARYLRAAAGGTRGSNHARIDAAEGPSDIPD

>XP_012356230.1 cyclin-dependent kinase inhibitor 2A isoform X1 [Nomascus leucogenys]

MEPSADWLATAAARGRVEEVRALLEAGALPNAPNSYGRRPIQVMMMGCARVAELLLLHGAEPNCADPATLTRPVHDAAREGFLDTLAVLHRAGARLDVRDAWGRLPVDLAEERGHRDVSRYLRAAAGGTRGSNHARRDAAEGPSDIPD

>XP_033093388.1 cyclin-dependent kinase inhibitor 2A isoform X2 [Trachypithecus francoisi]

MEPSADWLATAAARGRVEEVRALLEAGALPNAPNSYGRRPIQVMMMGSARVAELLLLHGAEPNCADPATLTRPVHDAARQGFLDTLAVLHRAGARLDVRDAWGRLPVDLAEEWGHRDVARYLRAAAGGTRGSTHARIDAAEGPSGIPD

>XP_031993926.1 cyclin-dependent kinase inhibitor 2A isoform X1 [Hylobates moloch]

MEPSADWLATAAARGRVEEVRALLEAGALPNAPNSYGRRPIQVMMMGCARVAELLLLHGAEPNCADPATLTRPVHDAAREGFLDTLAVLHRAGARLDVRDAWGRLPVDLAEERGHRDVSRYLRAAAGGTRGSNHARRDAAEGPSDIPE

>XP_010351690.1 cyclin-dependent kinase inhibitor 2A isoform X1 [Rhinopithecus roxellana]

MEPSADWLATAAARGRVEEVRALLEAGALPNAPNSYGRRPIQVMMMGSAHVAELLLLHGAEPNCADPATLTRPVHDAAREGFLDTLAVLHRAGARLDVRDAWGRLPVDLAEERGHRDVARYLRAAAGGTRGSSHARIDAAEGPSGIPD

>XP_023059648.1 cyclin-dependent kinase inhibitor 2A isoform X3 [Piliocolobus tephrosceles]

MEPSADWLATAAARGRVEEVRALLEAGALPNAPNSYGRRPIQVMMMGSARVAELLLLHGAEPNCADPATLTRPVHDAAREGFLDTLAVLHRAGAQLDVRDAWGRLPVDLAEERGHRDVARYLRAAAGGTRGSSHGRIDAAEGPSGIPD

>XP_011768776.1 cyclin-dependent kinase inhibitor 2A [Macaca nemestrina]

MEPSADWLATAAARGRVEEVRALLEAGALPNAPNSYGRRPIQVMMMSSARVAELLLLHGAEPNCADPATLTRPVHDAAREGFLDTLAVLHRAGARLDVHDAWGRLPVDLAEERGHRDVARYLRAAAGGTRGSSHARTDAAEGPSGIPD

>XP_003928239.2 cyclin-dependent kinase inhibitor 2A isoform X1 [Saimiri boliviensis boliviensis]

MEPSADWLATAAARGRVEEVRALLEAGAPPNAPNSYGRRPIQVMMMGSARVAELLLLHGAEPNCADPATLTRPVHDAAREGFLDTLVVLHRAGARLDVRDAWGRLPVDLAEERGHCDVAGYLRAAAGGTTGSSPARADAAEGPSDVPD

>XP_037600745.1 cyclin-dependent kinase inhibitor 2A isoform X1 [Cebus imitator]

MEPSADWLATAAARGRVEEVRALLEAGAPPNAPNSYGRRPIQVMMMGSARVAELLLLHGAEPNCADPATLTRPVHDAAREGFLDTLVVLHRAGARLDVRDAWGRLPVDLAEERSHCDVAGYLRAAAGGTTGSSPARTDAAEGPSDVPD

>XP_032155951.1 cyclin-dependent kinase inhibitor 2A isoform X1 [Sapajus apella]

MEPSADWLATAAARGRVEEVRALLEAGAPPNAPNSYGRRPIQVMMMGSARVAELLLLHGAEPNCADPATLTRPVHDAAREGFLDTLVVLHRAGAPLDVRDAWGRLPVDLAEERGHCDVAGYLRAAAGGTTGSSPARTDAAEGPSDVPD

>XP_008996005.1 cyclin-dependent kinase inhibitor 2A isoform X1 [Callithrix jacchus]

MEPSADWLATAAARGRVEEVRALLEAGAPPNAPNSYGRRPIQVMMMGSARVAELLLLHGAEPNCADPATLTRPVHDAAREGFLDTLVALHRAGARLDVRDAWGRLPVDLAEERGHCHVAGYLRAAAGGTTGSSPTRTDAAGGPSDVPD

>XP_012305433.1 cyclin-dependent kinase inhibitor 2A isoform X2 [Aotus nancymaae]

MEPSADWLATAAARGRVEEVRALLEAGAPPNAPNSYGRRPIQVMMMGSARVAELLLLHGADPNCADPATLTRPVHDAAREGFLDTLVVLHRAGARLDVRDAWGRLPVDLAEERGHCDVAGYLRAAEGGITGSSPARTDAAEGPSEMIGSHLWVCRRRHA

>XP_023988689.1 cyclin-dependent kinase inhibitor 2A-like isoform X1 [Physeter catodon]

METSADWLATAAARGRADEVRALLEAGAPAHAPNRYGRSPIQVMMMGSARVAELLLLHGADPNCADPATLTRPVHDAAREGFLDTLVALHRAGARLDVRDAWGRLPVDLAEEQGHRDVARYLRAAAGGTEGGSHARADSAEGPADSPALKNP

>XP_033713909.1 cyclin-dependent kinase inhibitor 2A isoform X1 [Tursiops truncatus]

METSADWLATAAARGRADEVRALLEAGAPAHAPNRYGRSPIQVMMMGSARVAELLLLHGADPNCADPATLTRPVHDAAREGFLDTLVALHRAGARLDVRDAWGRLPVDLAEERGHRDVARYLRAAAGGTEGGSHARADSAEGPADSPALKNP

>XP_012390375.1 cyclin-dependent kinase inhibitor 2A isoform X1 [Orcinus orca]

METSADWLATAAARGRADEVRALLEAGAPAHAPNRYGRSPIQVMMMGSARVAELLLLHGADPNCADPATLTRPVHDAAREGFLDTLVALHRAGARLDVRDAWGRLPVDLAEERGHRDVARYLRAAAGGTEGGSHARADSAEGPADNPALKNP

>XP_030686181.1 cyclin-dependent kinase inhibitor 2A-like isoform X1 [Globicephala melas]

METSADWLATAAARGRADEVRALLEAGAPAHAPNRYGRSPIQVMMMGSARVAELLLLHGADPNCADPATLTRPVHDAAREGFLDTLVALHRAGARLDVRDAWGRLPVDLAEERGHRDVARYLRSAAGGTEGGSHARADSAEGPADSPALKNP

>XP_022407374.1 cyclin-dependent kinase inhibitor 2A-like isoform X1 [Delphinapterus leucas]

METSADWLATAAARGRADEVRALLEAGAPAHAPNRYGRSPIQVMMMGSARVAELLLLHGADPNCADPATLTRPVHDAAREGFLDTLVALHRAGAQLDVRDAWGRLPVDLAEERGHRDVARYLRAAAGGTEGGSHARADSAEGPADSPALKNP

>XP_029059044.1 cyclin-dependent kinase inhibitor 2A-like isoform X2 [Monodon monoceros]

METSADWLATAAARGQADEVRALLEAGAPAHAPNRYGRSPIQVMMMGSARVAELLLLHGADPNCADPATLTRPVHDAAREGFLDTLVALHRAGAQLDVRDAWGRLPVDLAEERGHRDVARYLRAAAGGTEGGSHARADSAEGPADSPALKNP

>XP_026944715.1 cyclin-dependent kinase inhibitor 2A-like isoform X1 [Lagenorhynchus obliquidens]

METSADWLATAAARGRADEVRALLEAGAPAHAPNRYGRSPIQVMMMGSARVAELLLLHGADPNCADPATLTRPVHDAAREGFLDTLVALHRAGARLDVRDAWGRLPVDLAEERGHRDVARYLRAAAGGTEGGSHALADSAEGPADSPALKNP

>XP_036710646.1 cyclin-dependent kinase inhibitor 2A-like isoform X1 [Balaenoptera musculus]

METWADWLATAAARGRADEVRALLEAGAPAHAPNRYGRSPIQVMMMGSARVAELLLLHGADPNCADPATLTRPVHDAAREGFLDTLVALHRAGARLDVRDAWGRLPVDLAEERGHRDVARYLRAAAGGTEGGSHARADSAEGPADSPALKNH

>XP_032492349.1 cyclin-dependent kinase inhibitor 2A isoform X1 [Phocoena sinus]

METSADWLATAAARGRADEVRALLEAGAPAHAPNRYGRSPIQVMMMGSARVAELLLLHGADPNCADPATLTRPVHDAAREGFLDTLVALHRAGARLDVRDAWGRLPVDLAEERGHRDVARYLRAAAGGTEGGSHARAVSAEGPADSPALKNP

>XP_008055780.1 cyclin-dependent kinase inhibitor 2A [Carlito syrichta]

MEPSADRLATAAARGRAEEVRALLEAGAPPDAPNRHGRTPIQVMMMGSARVAELLLLHGAEPNCADPATLTRPVHDAAREGFLDTLVALHRAGARLDVRDAWDRLPVDLAEELGHRDVARYLQAAAGDTGGGSHARSDAAEDPSEILDLKNN

>XP_039736645.1 cyclin-dependent kinase inhibitor 2A-like isoform X4 [Pteropus giganteus]

MEPSADWLSAAAARGRADEVRALLEEGAPPNARNSHGRSPIQVMMMGSARVAELLLLHGADPNCADPATLTRPVHDAAREGFLDTVVALHRAGARLDVRDAWGRLPVDLAEERGHREVAGYLRAASGGREGGSHAGTDAAEGPTDTPIEDQRFFKGS

>XP_036876351.1 cyclin-dependent kinase inhibitor 2A-like isoform X1 [Manis javanica]

MEPTADRLATAAARGREDEVRALLEAGAPPNAPNRHGRSPIQVMMMGSARVAELLLLHGADPNCTDPTTLTRPVHDAAREGFLDTVVALHRAGAQLDVRDAWGRLPADLAEERGHRDVALYLRAAAGGIEGGSHLGTDAAEGPADSPDV

>XP_012625237.1 cyclin-dependent kinase inhibitor 2A [Microcebus murinus]

MEPTADWLAAAAARGRCEEVRALLEAGAPPNAPNRYGRTPIQVMMMGNARVAQLLLLHGAEPNCADPATLTRPVHDAAREGFLDTLVALHRAGARLDVRDAWGRLPVDLAEERGHRDVARYLRAALGDTEGGNQAAEGPPDSPDLKNL

>XP_036743499.1 cyclin-dependent kinase inhibitor 2A-like isoform X1 [Manis pentadactyla]

MEPTADRLATAAARGREDEVRALLEAGAPPNAPNRHGRSPIQVMMMGSARVAELLLLHGADPNCTDPTTLTRPVHDAAREGFLDTVVALHRAGARLDVRDAWGRLPADLAEERGHRDVALHLRAAAGGIEGGSHLGTDAAEGPADSPDV

>XP_039077187.1 cyclin-dependent kinase inhibitor 2A isoform X1 [Hyaena hyaena]

MEPSADWLATAAARGRAEEVRALLAAGAPPNARNRLGRSPIQVMMMGSTRVAELLLLHGADPNCADPATLTRPVHDAAREGFLDTLVVLHRAGARLDVRDAWGRLPVDLAEERGHRDVVRYLRAAMRGTGSGSHAGTEGAEGPADSWT

>XP_029777064.1 cyclin-dependent kinase inhibitor 2A-like isoform X2 [Suricata suricatta]

MEPSADRLATAAARGRAEEVRALLEAGVPPNARNRLGRSPIQVMMMGSARVAELLLLHGADPNCADPATLTRPVHDAAREGFLDTLVVLHRAGARLDVRDAWGRLPVDLAEERGHRHVARYLRAAMGGTGSGSHTPIDGVEGPADSWT

>XP_036094723.1 cyclin-dependent kinase inhibitor 2A isoform X2 [Rousettus aegyptiacus]

MEPSADWLSAAAARGRADEVRALLEEGAPPNAPNSHGRSPIQVMMMGSARVAELLLLHGADPNCADPVTLTRPVHDAAREGFLDTLVALHRAGARLDVRDAWGRLPVDLAEERGHREVSEYLRAASGGTEGGSHAAEGPTGEEANLRI

>XP_025137218.1 cyclin-dependent kinase inhibitor 2A-like isoform X1 [Bubalus bubalis]

MSCSLGAQGASWKSAWSALPGCRDSAGGVEGPSLGAASANPGGGKRRGWLLTSCLGGPPALDAGGRKGGSEGRALGSSMETSADSLAAAAARGRTDEVRALLEAGASANAPNRYGRSAIQVMMMGSARVAELLLFHGADANCADPATLTRPVHDAAREGFLDTLVALHRAGARLDVRDAWGRLPVDLAEERGHRDVARYLRAAAEDTEGGSHARADSAEGPADSSDLKKD

>XP_034519869.1 cyclin-dependent kinase inhibitor 2A isoform X1 [Ailuropoda melanoleuca]

MEPSADRLATAAARGRADEVRALLAAGVPPNARNRQGRSPIQVMMMGSTRVAELLLLHGADPNCADPTTLTRPAHDAAREGFLDTLVVLHRAGARLDVRDAWGRLPVDLAEERGHRAVAGYLRAAAGGTEAGSHASMEGAEGPADSPDLKND

>XP_005335984.2 cyclin-dependent kinase inhibitor 2A [Ictidomys tridecemlineatus]

MESSADWLASAAARGRAQEVRALLEAGAPPNAPNRYGRSPIQVMMMGNTRVARLLLLHGAEPNCADPATLTRPVHDAAREGFLDTLVALHLAGARLDVRDVWGRLPVDLAEERGHRDVAQYLRAAAGDTESGSPACTAGGEGPPDNPDLKNH

>XP_025214698.1 cyclin-dependent kinase inhibitor 2A isoform X2 [Theropithecus gelada]

MEPSADWLATAAARGRVEEVRALLEAGALPNAPNSYGRRPIQVMMMGSARVAELLLLHGAEPNCADPATLTRPVHDAAREGFLDTLAVLHRAGARLDVHDAWGRLPVDLAEERGHRDVARHPRLKDPPRL

>XP_037023895.1 cyclin-dependent kinase inhibitor 2A isoform X4 [Artibeus jamaicensis]

MEPSADWLATAAARGRAEEVRALLRAGAPPNAPNSHGRTPIQVMMMGSTRVAKLLLLHGADPNCADPATLATPAHDAAREGFLDTLVALHRAGARLDVRDAWGRLPVDLAEERGHQDVARYLRAAAAGTEGAGHSATDAAEGPADPDFDNQ

>XP_032164689.1 cyclin-dependent kinase inhibitor 2A-like isoform X4 [Mustela erminea]

MEPSADRLATAAARGRADEVRALLAAGVQPNALNRQGRRPIQVMMMGSTRVAELLLLHGADPNCADPVTLTRPVHDAAREGFLDTLVVLHRAGARLDVRDAWGRLPVDLAEERGHHAVAQYLGAASGATEGGSHSHSEVAEGPADSQDFKND

>XP_032249512.1 cyclin-dependent kinase inhibitor 2A-like isoform X2 [Phoca vitulina]

MEPSADRLATAAAQGRADEVRALLAAGVPPNARNRQGRSPIQVMMMGSTRVAELLLCHGAEPNCADPTTLTRPVHDAAREGFLDTLVLLHRAGARLDVRDAWGRLPVDLAEERGHRAVAGYLRAAAGGTEGGSHALTEVAEGPGRQPGLKE

>XP_030897373.1 cyclin-dependent kinase inhibitor 2A isoform X1 [Leptonychotes weddellii]

MEPSADRLATAAAQGRADEVRALLVAGVPPNARNRQGRSPIQVMMMGSTRVAELLLCHGAEPNCADPTTLTRPVHDAARDGFLDTLVVLHRAGARLDVRDAWGRLPVDLAEERGHRAVAGYLRAAAGGTEGGSHALMEVAEGPGRQPGLKE

>XP_035877431.1 cyclin-dependent kinase inhibitor 2A isoform X1 [Phyllostomus discolor]

MEPSADWLATAAARGREEEVQALLQAGAPPNAPNSYGRTPIQVMMMGSTRVAKLLLRHGADPNCADPATLATPVHDAAREGFLDTLVALHRAGAQLDVRDAWGRLPVDLAEERGHRDVARYLRAAAAAGTEGDSHRATDAAEDRADPDLLNQ

>XP_040487413.1 cyclin-dependent kinase inhibitor 2A-like isoform X3 [Ursus maritimus]

MESFADRLATAAARGRADEVRTLLAAGVPPNARNRQGRSPIQVMMMGSTRVAELLLLHGADPNCADPTTLTRPAHDAAREGFLDTLVVLHRAGARLDVRDAWGRLPVDLAEERGHRAVAGYLRAAAGGTEAGSHASMEGAEGPADSPDLKND

>XP_004373618.2 cyclin-dependent kinase inhibitor 2A [Trichechus manatus latirostris]

MEPTADSLATAAVRGRAQEVRELLQAGAQPNAPNRYGRRPIQVMMMGSTPVAELLLLHGAEPNCADPVTLTRPVHDAAREGFLDTLMVLHRAGARLDVRDAWGRLPVDLAEERGHRDVARYLRAAAGETEGRGHAHADAAEGAAAGRRQPQL

>XP_015104082.1 cyclin-dependent kinase 4 inhibitor B isoform X1 [Vicugna pacos]

MLSGGGGDARLANAAARGQVETVRQLLEAGADPNGLNCFGRRPIQVMMMGSVRVAELLLLHGADPNCADPATLTRPVHDAAREGFLDTLVALHRAGARLDVRDAWGRLPVDLAEERGHRDVVRYLRAAARDTGGGSHAHADAAEGPADSPNLKHH

>XP_040094185.1 cyclin-dependent kinase inhibitor 2A-like isoform X1 [Oryx dammah]

METSADSLAAAAARGRADEVRALLEAGESANAPNRYGRSAIQVMMMGSARVAELLLLHGADPNCADPATLTRPVHDAAREGFLDTLVALHRAGARLDVRDAWGRLPVDLAEERGHRDVTRYLRAAAEGTEGGRHARADSAEGPADSSDLKNY

>XP_021536720.1 cyclin-dependent kinase inhibitor 2A-like [Neomonachus schauinslandi]

METSADRLATAAAQGRADEVRALLAAGVPPNARNRQGRSPIQVMMMGSTRVAELLLCHGAEPNCADPTTLTRPVHDAARDGFLDTLVVLHRAGARLDVRDAWGRLPVDLAEERGHRAVAGYLHAAAGGTEGGSHALTEVAEGSADSPDLKND

>XP_026362230.1 cyclin-dependent kinase inhibitor 2A-like isoform X3 [Ursus arctos horribilis]

MESFADRLATAAARGRADEVRTLLAAGVPPNARNRQGRSPIQVMMMGSTRVAELLLLHGADPNCADPTTLTRPAHDAAREGFLDTLVVLHRAGARLDVRDTWGRLPVDLAEERGHRAVAGYLRAAAGGTEAGSHASMEGAEGPADSPDLKND

>XP_025708719.1 cyclin-dependent kinase inhibitor 2A-like isoform X2 [Callorhinus ursinus]

MEPSADRLATAAAQGLADEVRALLSAGVPPNARNRQGRSPIQVMMMGSTRVAELLLCHGAEPNCADPTTLTRPVHDAAREGFLDTLVVLHRAGARLDVRDAWGRLPVDLAEERGHRAVAGYLRAAAGGTEGGSHTLTEVAEGPGRQPGLKE

>XP_027786839.1 cyclin-dependent kinase 4 inhibitor B-like isoform X1 [Marmota flaviventris]

MESSADWLASAAARGRAQEVRALLEAGAPPNALNRYGRSPIQVMMMGNTRVARLLLLHGAEPNCADPATLTRPVHDAAREGFLDTLVALHLAGARLDVRDVWGRLPVDLAEERGHRDVAQYLRAAAGDTESGSPACKAGGEGPPGNPDLKNH

>XP_034872574.1 cyclin-dependent kinase inhibitor 2A-like isoform X2 [Mirounga leonina]

MEPSADRLATAAAQGRADEVRALLAAGVPPNARNRQGRSPIQVMMMGSTRVAELLLCHGAEPNCADPTTLTRPVHDAARDGFLDTLVVLHRAGARLDVRDAWGRLPVDLAEERGHGAVAGYLRAAAGGTEGGSHALTEVAEGPGRQPGLKE

>XP_032333484.1 cyclin-dependent kinase inhibitor 2A isoform X1 [Camelus ferus]

MEPSADWLTAAAARGRAEEVRALLEAGAPANAPNRYGRSPIQVMMMGSVRVAELLLLHGADPNCADPATLTRPVHDAAREGFLDTLVALHRAGARLDVRDAWGRLPVDLAEERGHRDVVRYLRVAARDTGGGSHAHADAAEGPAESPNLKHH

>XP_027970993.1 cyclin-dependent kinase inhibitor 2A-like isoform X3 [Eumetopias jubatus]

MEPSADRLATAAAQGLADEVRALLAAGVPPNARNRQGRSPIQVMMMGSTRVAELLLCHGAEPNCADPTTLTRPVHDAAREGFLDTLVVLHRAGARLDVRDAWGRLPVDLAEERGHRAVAGYLRAASGGTEGGSHTLTEVAEGPGRQPGLKE

>NP_001277177.1 cyclin-dependent kinase inhibitor 2A [Felis catus]

MEPLADRLATAAARGRAEEVRALLAAGAQPNAPNRLGRSPIQVMMMGSARVAELLLLHGADPNCADPATLTRPVHDAAREGFLDTLVVLHRAGARLDVRDAWGRLPVDLAEERGHRDIVRYLRAATGGTGSGSHTGTDGAEGVADSRT

>KAF7486255.1 cyclin-dependent kinase inhibitor 2A-like [Marmota monax]

MESSADWLASAAARGRAQEVRALLEAGAPPNALNRYGRSPIQVMMMGNTRVARLLLLHGAEPNCADPATLTRPVHDAAREGFLDTLVALHLAGARLDVRDVWGRLPVDLAEERGHRDVAQYLRAAAGDTESGSPACTAGGEGPPGEDRWTTST

>XP_012668933.1 cyclin-dependent kinase inhibitor 2A isoform X1 [Otolemur garnettii]

MEPTADWLSTASALGRAEEVRALLEAGAPPNAPNRQGRTPIQVMMMGSIRVAELLLLHGADPNRADPVTLTRPVHDAAREGFLDTLMALHQAGAQLDVRDVWGRLPVDLAEERGHCDVARYLRQASGDTEGRSQVGTNAAESPADGPE

>XP_040337315.1 cyclin-dependent kinase inhibitor 2A-like [Puma yagouaroundi]

MEPLADRLATAAARGRAEEVRALLAAGAQPNAPNRLGRSPIQVMMMGSARVAELLLLHGADPNCADPATLTRPVHDAAREGFLDTLVVLHRAGARLDVRDAWGRLPVDLAEERGHRDIVRYLRAATGGTGTGSHTGTDGAEGVADSRT

>XP_023392085.1 cyclin-dependent kinase 4 inhibitor B isoform X1 [Pteropus vampyrus]

MREEDKGVLSGGGGDAGLASAAARGQVEMVRQLLEAGADPNKVNCFGRSPIQVMMMGSARVAELLLLHGADPNCADPATLTRPVHDAAREGFLDTVVALHRAGARLDVRDAWRLPVDLAEERGHREVAGYLRAASGGREGGSHAGTDAAEGPTVGCAAALSLPSAWRARSAEAVGEPSTASRTVDACGISEARKCGDSRQGRDSRNRSLR

>XP_010806061.2 cyclin-dependent kinase inhibitor 2A isoform X2 [Bos taurus]

METSADLLAAAAALGWAEEVRALLEAGASANAPNRYGRSAIQVMMMGSARVAELLLLHGADPNCADPATLTRPVHDAAREGFLDTLVALHRAGAQLDVRDAWGRLPVDLAEERGHRDVARYLRAAAEDTEGGSHASADSAEGPADSSDLKKD

>XP_020764590.1 cyclin-dependent kinase inhibitor 2A-like isoform X1 [Odocoileus virginianus texanus]

METSADSLAAAAARGRADEVRALLEAGVSANAPNRYGRSAIQVMMMGSARVAELLLLHGADPNCADPATLTRPVHDAAREGFLDTLVALHRAGARLDVRDAWGRLPVDLAEERGHRDIAGYLRAAAEGTEGGSQARADPAEGPADSSDLKNH

**>NP_001279028.1 cyclin-dependent kinase inhibitor 2A/B (p15, inhibits CDK4) [Callorhinchus milii]**

MANNDELANAAAQGDMHRMRELLDQGADPNGINSFDRTPIQVMKMGVPAVAQLLLERGADPNRQDSMGMALIHDVAREGFLDTLQVLVGFGHANPSLRNASNHLPVELASQSGHTDVVRYLDNLPKAE

>XP_038660562.1 cyclin-dependent kinase 4 inhibitor B [Scyliorhinus canicula]

MANNDELANAAAQGNIGKVEQLLNNGADPNGINSFDRTPIQVMKMGCPSLCQLLLRHGADPNRQDNQQIAIIHDVAREGFLDTLQILVEIGNADTNLRDRNNRLPIDLAMQNNHGDVVTYLSNLTQSD

>XP_041041550.1 cyclin-dependent kinase 4 inhibitor B-like isoform X1 [Carcharodon carcharias]

MANNDKLANAAAQGDIGKVIQLLNNGADPNGISSFGRTPIQVMKMGCPAVCRLLLEHGADPNRQDNQQIALIHDVAREGFLDTLKVLVEFGMADTNLRDKNNCLPIDLAKQNNHWEVVKYLGNLKQGD

>KAF7244571.1 Cyclin-dependent kinase inhibitor 2A [Varanus komodoensis]

MDPCGNNDLLANAAARGDTGAVERLLDSGADPNALNSFGRTPLQVMMMGNPRVAELLLQRGADPNRPDPSTGCLPAHDVACEGFLDTLQVLHGGGARFDLPNHQGRLPIDLAKNGGHFFVVHYLQELSG

>XP_007065501.1 cyclin-dependent kinase 4 inhibitor B [Chelonia mydas]

MGQPGANDMANAAALGNLGKVRELLDQGADPNAVNSYDRTPIQVMMMGNTQVAELLLQRGADPNRPDPRTGSLPVHDAAREGFLDTLVALHRGGARLDLRDTWGRLPIDLAVESGHQQVVSYLRAQPARGAAPPQA

>XP_005294794.1 cyclin-dependent kinase 4 inhibitor B-like isoform X1 [Chrysemys picta bellii]

MDQSGANDMASAAALGNLEKVRELLDQRADPNAVNSYDRTPIQVMMMGNTQVAELLLQRGADPNRPDPRTGSLPVHDAAREGFLDTLVALHRGGARLDLRDKSGRLPIDLAVESGHQQVVSYLRAQPARNAAPPQA

>XP_024067399.2 cyclin-dependent kinase 4 inhibitor B-like [Terrapene carolina triunguis]

MGQPGANDMASAAALGNLEKVRELLDQRADPNAVNSYDRTPIQVMMMGNTQVAELLLQRGADPNRPDPRTGSLPVHDAARDGFLDTLVALHRGGARLDLRDKSGRLPIDLAAESGHQQVVSYLRAQPARNAAPPQA

>XP_034629946.1 cyclin-dependent kinase 4 inhibitor B-like [Trachemys scripta elegans]

MGQSGANDMASAAALGDLEKVRELLDLRADPNAVNSYDRTPIQVMMMGNTQVAELLLQRGADPNRPDPRTGSLPVHDAAREGFLDTLVALHRGGARLDLRDKSGRLPIDLAVESGHQQVVSYLRAQPARNAGRVASSLRGSALLSHTKH

>XP_028568881.1 cyclin-dependent kinase 4 inhibitor B-like [Podarcis muralis]

MDPAPGSDIDGGGGRGEADELANAAARGDLETLRRLLDGGADPNAVNRFGRAPIQVMMMGNPGVAQLLLQRGADPNRPDPSTGTFPVHDAARGGFLDTLRVLRRGSARFDVRDRWGRRPLDLAEESGQSRVVSFLQEASG

>XP_032639132.1 cyclin-dependent kinase 4 inhibitor B-like [Chelonoidis abingdonii]

MQAGAVAAGGERDLLANVAAQGDVQRLRLLLEAGMNPNTVNSFGRTPIQVMMMGNTRVAELLLERGADPNRPDPRTGSLPVHDAAREGFLDTLVALHRGGARLDLGDESGRLPIDLAVESGHQQVVSYLRAQPAGNAAPPQA

>XP_039399220.1 cyclin-dependent kinase 4 inhibitor B [Mauremys reevesii]

MQPGAVAAGGERDLLANAAAQGDVQRLRLLLDAGMNPNTVNSFGRTPIQVMMMGNTRVAELLLQRGADPNRPDPRTGSLPVHDAAREGFLNTLVALHRGGARLDLGDRSGRLPIDLAVESGHRQVVSYLRAQPAGNAAPPQA

>XP_005807179.1 cyclin-dependent kinase 4 inhibitor B [Xiphophorus maculatus]

MTVEDELTTAAAKGHTAEVEALLLQGAPVNGVNSFGRRAIQVMMMGSSEVARLLLTRGADPNVTDKSTGATPLHDAARTGFLDTVQLLVKAGADPQARDKDNCLPIDLARQNGHTDVVAVLETL

>XP_022518819.1 cyclin-dependent kinase 4 inhibitor B-like isoform X1 [Astyanax mexicanus]

MNNNTSDAARERRNSEARAGMQDELATAAANGDTARTRELLDNGADVNGTNRFGRTPIQVMMMGSTPVARLLLSRGADPNVPDLDTGATPLHDAARAGFLDTVRALLEFNANPELKDAQHKTPAETAREHGHEPIAECLNKITSSTQQPNNP

>TFK10370.1 Cyclin-dependent kinase 4 inhibitor B [Platysternon megacephalum]

MQAGAVAAGGERDLLANAAAQGDVQGVRRLLEAGMNPNTVNSFGRTPIQVMMMGNTQVAELLLHRGADPNRPDPRTGSLPVHDAAREGFLDTLMALHRGGARLDLRDKWGRLPIDLAGESGHQQVVSYLRAQPARNAAPPQA

>XP_027873615.1 cyclin-dependent kinase 4 inhibitor B [Xiphophorus couchianus]

MTLEDELTTAAAKGHTAEVEALLLQGAPVNGVNSFGRRAIQVMMMGSSEVARLLLTRGADPNVTDKSTGATPLHDAARTGFLDTVQLLVKAGADPQARDKDNCLPIDLARQNGHTDVVAVLETL

>XP_032417726.1 cyclin-dependent kinase 4 inhibitor B [Xiphophorus hellerii]

MTLEDELTTAAAKGHTAEVEALLLQGAPVNGVNSFGRRAIQVMMMGSSEVARLLLTRGADPNVTDKSTGATPLHDAARTGFLDTVQLLVEAGADPQARDKDNCLPIDLARQNGHTDVVAVLETL

>XP_003452283.1 cyclin-dependent kinase 4 inhibitor B [Oreochromis niloticus]

MTLQDELTTAAAKGNTAAVKALLDRGAQVNGTNSFGRTALQVMMMGSTSVAQLLLEHGANPNVGDSSTGASPLHDAARTGFVDTVHLLVQHHADPQARDKLNRLPVDLARQHGHGDVVDFLESLQNP

>XP_005294793.1 cyclin-dependent kinase 4 inhibitor B-like [Chrysemys picta bellii]

MQAGAVAADGGRDLLANAAAQGDVQGVRLLLEAGMNPDTVNSFGRTPLQVMMMGNTQVAELLLQRGADPNRPDPRTGSLPVHDAAREGFLDTLVALHRGGARLDLRDKSGRLPIDLAVESGHQQVVSYLRAQPARNAAPPQA

>XP_038160696.1 cyclin-dependent kinase 4 inhibitor B [Cyprinodon tularosa]

MTLEDELTTASAKGDTAEVKRLLLKGAPVNGLNGFGRTALQVMMMGSSPVAQLLLEHGADPNVADSSTGSTPLHDAARAGFLDTVRLLVEAGANLQARDKADCLPIDLARQSGHSDVVSFLETL

>XP_030206751.1 cyclin-dependent kinase 4 inhibitor B [Gadus morhua]

MTLVDDLASAAATGNTAGVEDLLRRGADVNGVNKYGRTPLQVMMMGSTPVARLLLARGSRPNVADSSTGTTPLHDAAREGFLDTLRVLLEFKADPGARDHFNSRPVDLARQSGHDDVVEFLKLLEEAN

>XP_031608070.1 cyclin-dependent kinase 4 inhibitor B [Oreochromis aureus]

MTLQDELTTAATKGNTAAVKALLDRGAQVNGTNSFGRTALQVMMMGSTSVAQLLLEHGANPNVGDSSTGASPLHDAARTGFVDTVRLLVQHHADPQARDKLNRLPVDLARQHGHGDVVDFLESLQNP

>XP_029605413.1 cyclin-dependent kinase 4 inhibitor B-like [Salmo trutta]

MTMPLEDELASAAATGNTDRVKILLGRGVDVNGVNCFGRTPLQVMMMGSSPVAQLLLMQGADPNIADRHTGTTPLHDAARMGFLDTVEILVQFHADPNSRDNRNCRPIDLAIESGHHNVVAFLQAL

>XP_027740489.1 cyclin-dependent kinase 4 inhibitor B-like [Empidonax traillii]

MARRAGGSVADELANAAARGDLQRLRELLDGEADPNAVNSYGRTPIQVMMLGSTRVAELLLQRGADPNRPDPSTGCLPVHDAVRAGFLETLVALHRAGARLDLPDGRGHLPLDVAAGGPHGAVGRYLRHPPPRA

>XP_041077471.1 cyclin-dependent kinase inhibitor 2A [Polyodon spathula]

MNADKLTEAAANGDTERVQFLLENGIAPNAVNRFGRTPIQVMMMGNTKIAQLLLLYGANPNISDSSTGLTPVHDAAREGFLDTVMVLVRNSANTNVRDGRNLLPIDLARENGHGDVIAFLESNNF

>XP_022049407.1 cyclin-dependent kinase 4 inhibitor B-like [Acanthochromis polyacanthus]

MSLQDELTTAAAKGNTADVEDLLRAGAPVNGVNKFGRTAVQVMMMGSSPVAQALLEHGADPNVADPSTGSTPLHDAARTGFVDTARLLVKFLADPQARDHANRLPIDLAAQNGHTDVVAFLETL

>XP_027519474.1 cyclin-dependent kinase 4 inhibitor B [Corapipo altera]

MARRAGGSVADELANAAARGDLQRLRELLDGAADPNAVNSYGRTPIQVMMLGSPRVAELLLQRGADPNRPDPSTGCLPAHDAARAGFLETLAALHRAGARLDQPDGRGRLPLDVAAGGPHGAVGRYLRHPPPRV

>XP_020042100.1 cyclin-dependent kinase 4 inhibitor B [Castor canadensis]

MREDDKGMLGGGASDAGLANAAARGQVEKVRQLLEAGADPNGVNRFGRRPIQVMMMGSARVAELLLLYGAEPNCADSATLTRPVHDAAREGFLDTLVALHGAGARLDVRDAWGRLPVDLAEERGHRHVARYLHAAVGD

>XP_041082318.1 cyclin-dependent kinase 4 inhibitor B-like [Polyodon spathula]

MNADKLTKAVATGDTERVRFLLENGIDPNGVNRFGRTPIQVMMMGNTRIAELLLLHGANPNTVDSSTGLTPIHDAAREGFLDTVVMLVRNNADTNVRDKRDLLPLDLARENGHEELVAYLESLE

>XP_035418918.1 cyclin-dependent kinase 4 inhibitor B-like [Cygnus atratus]

MAQRAGSAAADELSNAAARGDVQRVRELLDGAADPNAVNSFGRTPIQVMMLGSPRVAELLLQRGADPNRPDPRTGCFPAHDAARAGFLDTLAALHRAGARLDLPDGRGRLPLDVAAGGPHGPVGRFLRQPADGPLP

>NP_001134648.1 Cyclin-dependent kinase 4 inhibitor B [Salmo salar]

MTMPLEDDLASAAATGNTDRVKILLGRGVDVNGVNCFGRTPLQVMMMGSSPVAQLLLMQGADPNIADRHTGTTPLHDAARMGFLDTVEILVQFHADPNSRDNRNCRPIDLAIESGHRNVVAFLQAL

>XP_039879987.1 cyclin-dependent kinase 4 inhibitor B [Simochromis diagramma]

MDVTMTLQDELTTAAAKGNTAAVEALLDRGAQVNGTNSFGRTALQVMMMGSTSVAQLLLEHGANSNVGDSSTGASPLHDAARTGFLDTVHLLVQHHADPQARDKLNRLPVDLARQHGHRDVVDFLESLQNP

>XP_038257782.1 cyclin-dependent kinase 4 inhibitor B-like [Dermochelys coriacea]

MQAGAVAAGGERDLLANAAAQGDGRKVRLLLEAGVNPNTVNAFGRTPIQVMMMGNTQVAELLLQRGADPNRPDPLTGSLPVHDAAREGFLDTLVALHRGGARLDLRDTWGRLPIDLAVENRHQQVVSYLRAQPARGAAPPQA

>ACO08438.1 Cyclin-dependent kinase 4 inhibitor B [Oncorhynchus mykiss]

MTMPLEDDLASAAATGNTNRVRILLQSGVDVNGVNCFGRTPLQVMMMGSSPVAQLLLMQGADPNIADRHTGTTPLHDAARMGFLDTVEILVQFLADPNSRDNRNCRPIDLAIESGHSNVVAFLKAL

>XP_036034694.1 cyclin-dependent kinase inhibitor 2A-like isoform X2 [Onychomys torridus]

MEPSADRLARAAAQGREHEVRALLEAGASPNAPNCFGRTPIQVMMMGNAQVASLLLLYGAEPNREDPATLSRPVHDAAREGFLDTLVVLHQAGARLDVRDAWGRLPVDLARERGHRDVVRYLRAAGSAPQGSGPAGIASAQAPPDAPDFADHP

>XP_037653945.1 cyclin-dependent kinase 4 inhibitor B [Choloepus didactylus]

MREEDKGMLGGDGDDVGLANAAARGQVETVRHLLEAGADPNGVNCYGRRPIQVMMMGSARVAELLLLHGAEPNCADPVTLTRPVHDAAREGFLDTLLALHRAGARLDVRDAWGRLPVDLAEESGHCDIVRYLHAAAGD

>XP_023147531.1 cyclin-dependent kinase 4 inhibitor B [Amphiprion ocellaris]

MSLQDKLTTAAAKGNTADVEDLLRAGAPVNGVNKFGRTAVQVMMMGSSSVAQALLEHGADPNVADRSTGSTPLHDAARTGFLDTARLLVKFLADPRARDHANRLPIDLAAQNGHRDVVAFLETL

>XP_032069719.1 cyclin-dependent kinase 4 inhibitor B-like [Thamnophis elegans]

MEPTCRNTPSERLSRAAAEGNVAELRRLLDGGADPNGLNVYGRTAIQVMKLGNPQVAELLLERGANPNVPDRSTGSLPVHDAAREGFLDTLQVLIAGGARLDLPNYYDRLPLDEAAENGQNHVVHFLTQRQGDNA

>XP_033871745.1 cyclin-dependent kinase inhibitor 2A-like [Acipenser ruthenus]

MNADKLTEAAAKGNTERVQFLLENGIAPNAVNCFGRTAIQVMMMGNTRIAQLLLLYGANPNISDSSTGLTPVHDAAREGFLDTVKMLVQNYANTNVRDGRDLLPIDLARENGHKDVIAYLESNNV

>XP_026026987.1 cyclin-dependent kinase 4 inhibitor B-like [Astatotilapia calliptera]

MDVTMTLQDELTTAAAKGNTAAVEALLERGAQVNGTNSFGRTALQVMMMGSTSVAQLLLEHGANSNVGDSSTGASPLHDAARAGFLDTVHLLVQHHADPQARDKLNRLPVDLARQHGHRDVVDFLESLQNP

**>NP_001006920.1 cyclin-dependent kinase 4 inhibitor B [Xenopus tropicalis]**

MAFNANTLCSACARGDVDLARQMLQSGIPVNATNSHGRTPIQVMMMGSPKMAQLLLDHGADPKLPDPCTGACPVHDAAREGFLDTLLVLLNNGASLYEPRDNFGQRPIDLAPPHLRAQLLLLGYN

>NP_001090330.1 cyclin-dependent kinase inhibitor 2B L homeolog [Xenopus laevis]

MDFNANTLCCASARGDVNLARQMLQSGIPVNAPNSHGRTPIQVMMMGSPQMAELLLDYGADPSVPDPSTGTCPTHDAAREGFLDTLLVLLRNGANLYEPRDNYGQRPIDLASPDLKAKLLLLGYR

>XP_018099263.1 cyclin-dependent kinase inhibitor 2A [Xenopus laevis]

MDFNANTLCCASARGDVDLARQILQSGIPVNAPNSHGRTPIQVMMMGSPQMAELLLDQGADPNVPDPSTGTCPAHDAAREGFLDTLLVLLRNGASLYAPLDNYGQRPIDLASPYLKSKLLLLGYH

>XP_040276266.1 cyclin-dependent kinase inhibitor 2A-like isoform X1 [Bufo bufo]

MEELLTMAAAQGDVQLVRAFLENGANPNSSNSHGRTAIQQVMMMGSPHLAQLLIDHGADPTIPDPTTGTCPAHDAIREGFVDTLVVLINGGASIDGPPDNFGQRPIDLASPLVLEKLKALGLAGN

>XP_040214868.1 cyclin-dependent kinase 4 inhibitor B-like [Rana temporaria]

MAEDLLATAAATGNVQRVSEKLNEGVDPNAPNSLGRTPIQVMKMGCPQIARLLIEHGADPLIPDPSTGTCPAHDAIREGFVDTLLELVKGGASLYKPEDNFGQRPIDLASASVRERLIQLGILPH

>XP_029462655.1 cyclin-dependent kinase inhibitor 2A-like [Rhinatrema bivittatum]

MSTARADALCRAAAGGDLSLVRLLLEEGTNPNDKNSYNRSAIQVMKLGNPRLAELLLRYGADPNLPDPTTATCPAHDAAREGFLDTLRVLLRAGASLERRDRWGRTPLDLVPEHLRAGLLDSLPQR

>XP_036594605.1 cyclin-dependent kinase inhibitor 2A-like isoform X2 [Trichosurus vulpecula]

MHRRHSPEKSMNLSVESLTEAAARGQTEIVRELLESGTDPNVVNRFGRSAIQVMMMGSVRVAELLLQHGADPNTPDPTTLALPVHDAAREGFLDTLMLLHRAGARLDVRDSLGRLPVDLAEEQGHHLVVTYLREVVKGGLKSTIQHVE

>XP_030050079.1 cyclin-dependent kinase inhibitor 2A-like [Microcaecilia unicolor]

MSRAGADALTRADALTRAAARGDLLLAQSLLEDGTDPNGMNSFHRTAIQVMKLGNPMLAELLLRYGADPNVPDPTTGTCPAHDAAREGFLDTLQVLVTGGARLDQCDTWGQTPIDLVPRHLVDALLKNLPHQ

>XP_037653946.1 cyclin-dependent kinase inhibitor 2A-like [Choloepus didactylus]

MEQLADRLCAAAARGRVEEVRELLQAGAQPNAPNRFGRCPIQVMMMGSARVAELLLLHGAEPNCADPVTLARPVHDAAREGFLDTLLALHRAGARLDVRDAWGRLPVDLAEERGHHHVVGYLRAAVGAPEAV

>XP_007434878.1 cyclin-dependent kinase 4 inhibitor B [Python bivittatus]

MDRSGDRDRANQLTSAAARGDLESVGRLLESGADPNATNAFGRSPIQVMMMGSSKMAELLLQRGADPNRPDPSTGATPAHDVAWGGFLDTLKILYQWGARFDQVDKWGRCPLDLAKENGQNHVVDYLQELSG

>XP_032716183.1 cyclin-dependent kinase inhibitor 2A-like isoform X3 [Lontra canadensis]

MEPSADRLATAAARGRADEVRALLEAGVQPNAVNRQGRSPIQVMMMGNTRVAELLLLHGADPNRADPITLTRPVHDAAREGFLDTLLVLHRAGARLDVRDAWGRLPVDLAEERGHRAVARYLGAAAGATEGGSHSHTEVAEGPADSQDFKND

>XP_030824973.1 cyclin-dependent kinase 4 inhibitor B-like [Camarhynchus parvulus]

MEGSPRSDGDSLCSAAARGDHEEVRRLLQAGVDPNGTNRFGRTPLQVMMLGSPRVAELLLRHGADPSRPDPRTGCLPVHDAARAGFVETLAALHRAGARLDIPDGRGRLPLDVAAGGPNGVVGRYLRDPPPLPGAGEAAEKAAR

>XP_033775353.1 cyclin-dependent kinase inhibitor 2A-like [Geotrypetes seraphini]

MSRERADELTGAAARGDLLLVQSLLERGSDPNATNSFHRTAIQVMKLGNSKLAELLLRYGADPNVPDPSTGTYPAHDAAREGFLNTLQVLVMGGARLDHCDKWGQTPIDLVPQHLLAALPENFPQSRRP

>XP_036259132.1 cyclin-dependent kinase 4 inhibitor B-like [Molothrus ater]

MEGSPRSDGDRLCSAAARGDHEEVRRLLQVGVDPNGTNRFGRTPLQVMMLGSPRVAELLLRHGADPNRPDPRTGCLPVHDAARAGFLETLAALHRAGARLDLPDGRGRLPLDVAAGGPHGAVGRYLRDPLPLPGAGGAAEKAAR

>XP_037981488.1 cyclin-dependent kinase 4 inhibitor B-like isoform X2 [Motacilla alba alba]

MEGSPRSDGDRLCSAAARGDHEEVRRLLQAGVDPNGTNRFGRTPLQVMMLGSPRVAELLLRHGADPNRPDPRTGCLPVHDAARAGFLGTLAALHRAGARLDLPDGRGRLPLDVAAGGPHGAVGRYLRDPPPLPGAGGAAEKAAR

>XP_014431942.1 cyclin-dependent kinase 4 inhibitor B-like [Pelodiscus sinensis]

MDYGREWSGGSRSEGGAQLATAAAQGDVARVRQLLEVAGVNPNIVNSFGRTPIQVMMLGSTKVAELLLQSGADPNRPDPSTGSLPVHDAARGGFLDTLVALHRGGARLDLRDGWGRLPVDLAVESGQQQVVRYLRAQNVAPPRE

>XP_033928916.1 cyclin-dependent kinase 4 inhibitor B-like [Melopsittacus undulatus]

MQASLRGDCSGDRLCSAAARGDREEVRKLLEAGADPNATNSFGRTPLQVMMLGSPRVAELLLQRGADPNRPDPRTGCLPAHDAARAGFLETLAVLHRAGARLDLPDGSGRLPLDVAAGGPHGPVARFLRHLPPLSPAAQVAGLVAEGVKR

>XP_008152306.1 cyclin-dependent kinase 4 inhibitor B [Eptesicus fuscus]

MWEEEKGKLGGSGCDAGLASAAARGQVETVRQLLQAGADPNGVNGFGRRPIQVMMMGCTRVAELLLTHGADPNRPDPATLTRPVHDAAREGFLDTLVALHRAGARLDVRDAWGRLPADLAEERGHRDLVGYLCATAGD

>XP_015470793.1 cyclin-dependent kinase 4 inhibitor B isoform X2 [Parus major]

MEGPPRGDVDRLCSAAARGDHEEVKKLLDSGVDPNGTNAFGRTPLQVMMLGSPRVAELLLRRGADPNRPDPRTGCLPVHDAARAGFLETLAVLHRAGAHLHLPDGRGRLPLDVAAGGPHGAVGRYLRDPPPLPGAAGAAEKAER

>XP_022378231.1 cyclin-dependent kinase inhibitor 2A-like isoform X2 [Enhydra lutris kenyoni]

MEPSADRLATAAARGRADEVRALLEAGVQPNALNRQGRSPIQVMMMGSTRVAELLLLHGANPNCADPITLTRPVHDAAREGFLDTLLVLHRAGAQLDVRDAWGRLPVDLAEERGHRAVARYLGAAAGATEGGSHSHTEVAESPADSQDFKND

>XP_027526069.1 cyclin-dependent kinase 4 inhibitor B-like [Neopelma chrysocephalum]

MEGSPRGDGDRLCTAAARGDREEVRKLLDAGVDPNATNRFGRTPLQVMMLGSTRVAELLLQRGADPNRPDPSTGCLPAHDAARAGFLETLVALHRAGARLDQPDGFGHLPLDVAAGGPHGAVGRYLRHPPPLPGAGRAAEGAAR

>NP_001268468.1 cyclin-dependent kinase 4 inhibitor B [Mesocricetus auratus]

MLGGGSDAGLATAAARGQVETVRQLLEAGVDPNAVNRFGRRPIQVMMMGSTQVAELLLLHGAEPNCADPNTLTRPVHDAAREGFLDTLVVLHRAGARLDVRDTWGRLPVDLAEEMGHHDVAVYLHAATGD

>XP_027256203.1 cyclin-dependent kinase 4 inhibitor B [Cricetulus griseus]

MLGGGSDAGLATAAARGQVETVRQLLEAGVDPNAVNRFGRRPIQVMMMGSTQVAELLLLHGAEPNCADPNTLTRPVHDAAREGFLDTLVVLHRAGARLDVRDTWGRLPVDLAEEMGHHDIAIYLHAATGD

>XP_036187174.1 cyclin-dependent kinase 4 inhibitor B [Myotis myotis]

MWEEEKVKLGGNGSDARLASAAARGQVETVRQLLQAGADPNGVNGFGRRPIQVMMMGSTRVAELLLIHGADPNCADPATLTRPVHDAAREGFLDTLVALHRAGARLDVRDAWGRLPVDLAEERSHRDIVRYLCATAGD

>XP_041254326.1 cyclin-dependent kinase 4 inhibitor B-like [Onychostruthus taczanowskii]

MEVSPRSDGDRLCSAAARGDCEEVRRLLHAGVDPNGTNAFGRTPLQVMMLGSPRVAELLLRHGADPNRPDPRTGCLPAHDAARAGFLETLAALHRAGARLDIPDGRGRLPLDVAAGGPHGAVGRYLRDPPPLPGAGGTAEKAAR

>XP_002194838.2 cyclin-dependent kinase inhibitor 2A-like [Taeniopygia guttata]

MGRGWGRKAAAPLPSPPSPSALRHPHPLSPREPLALEAGMKPGCPPAVSEGPAAATLRSFCRPAGAPRPGRMRALYLSRWQPRRGGADTAAPLRWRGARSGRDGRGRRGRGCEGAVAAPEKGAGSQRRARSQPGERESQRRPCASGRRGGMEGSPRSDGDRLCSAAARGDHEEVRKLLQAGVDPNGTNAFGRTPIQVMMLGSPRVAELLLRHGADPNRPDPRTGCLPAHDAARSGFLETLAALHRAGARLDLPDGRGRLPLDVAAGGPHGAVRRYLRDPPAFLGAGGVAEKPAP

>KAF6433079.1 cyclin dependent kinase inhibitor 2B [Molossus molossus]

MWEEDKIMPGGGSSDAGLASAAARGQVETVRQLLEAGADPNGVNGFGRRPIQVMMMGSTRVAELLLLHGADPNCADPATLTRPVHDAAREGFLDTLVVLHQAGARLDVRDAWGRLPVDLAEEQGHRDLARYLCTAAGD

>KQK82854.1 cyclin-dependent kinase 4 inhibitor B-like protein [Amazona aestiva]

MEASLRDDASGDRLCSAAARGDREEVRKLLDAGADPNATNSFGRTPLQVMMLGSPRVAELLLQRGADPNRPDPRTGCFPAHDAARAGFLETLAVLHRAGARLDLPDGSGSLPFDVAAGGPQGAVARFLRHPPPLPATAQVAGMAAGGAKR

>XP_032531650.1 cyclin-dependent kinase 4 inhibitor B-like [Chiroxiphia lanceolata]

MEGSPRGDGDRLCTAAARGDREEVRRLLDAGVDPNATNCFGRTPLQVMMLGSPRVAELLLQRGADPNRPDPSTGCLPAHDAARAGFLETLAALHRAGARLDQPDGRGRLPLDVAAGGPHGAVGRYLRHPPPLPGAGRSAEGAAR

**>XP_026574384.1 cyclin-dependent kinase 4 inhibitor B-like [Pseudonaja textilis]**

MEAPNGDGCQANQLANAAARGDLETAARLLESGADPNALNVFGRSPIQVMMLGSSQMAELLLRRGADPNRPDPSTGATPAHDVAQEGFLDTLKLLHHWGAHFDHLDRWGRSPLDLARQNGQNHVVDYLQELPG

>XP_026528057.1 cyclin-dependent kinase 4 inhibitor B-like [Notechis scutatus]

MEAPNGDGCQANQLANAAARGDVETAARLLESGADPNATNVFGRSPIQVMMVGSSKMAELLLRRGADPNRPDPSTGATPAHDVAQEGFLDTLKLLHHWGAHLDHLDRWGRSPLDLARKKGQNHVVDYLQELSG

>XP_039212974.1 cyclin-dependent kinase 4 inhibitor B-like [Crotalus tigris]

MHRLGARPRDTAESNMEAPNRDNCQANQLANAAARGDLETVAGLLESGADPNAINGFGRTPIQVMMMGSARMAELLLQRGADPNRPDPSTGATPAHDLAQEGFLDTLMILHHWGARFDQLDRWGHSPLDLARQNGQNHVVDYLQELSG

>XP_034266413.1 cyclin-dependent kinase 4 inhibitor B-like [Pantherophis guttatus]

MEAPNGDEWQANQLANAAARGDLETATRLLESGADPNATNVFGRSPIQVMMMGNSKMAELLLQRGADPNQPDPSTGAMPAHDVSREGFLDTLKLLHHWGARFDHLDRWDHSPLDLARMNGHNHVVDYLQELSG

>XP_029141552.1 cyclin-dependent kinase inhibitor 2A-like [Protobothrops mucrosquamatus]

MMMGSARMAELLLQRGADPNRPDPSTGATPAHDLAQEGFLDTLMILHHWGARFDQLDRWGHSPLDLARQNGQNHVVDYLQELSG

>XP_038190584.1 cyclin-dependent kinase 4 inhibitor B [Arvicola amphibius]

MLGGGSDEGLATAAARGQVETVRRLLEAGADPNAVNRFGRRPIQVMMMGSAQVAELLLLHGAEPNCADPTTLTRPVHDAAREGFLDTLVMLHKAGAWLDVCDAWGRLPVDLAEEQGHRDIARYLHAASGD

>XP_032757997.1 cyclin-dependent kinase 4 inhibitor B [Rattus rattus]

MLGGGSDAGLATAAARGQVETVRQLLEAGADPNAVNRFGRRPIQVMMMGSAQVAELLLLHGAEPNCADPATLTRPVHDAAREGFLDTLVVLHKAGARLDVCDAWGRLPVDLAEEQGHSDIARYLHAATGD

>XP_004456128.1 cyclin-dependent kinase 4 inhibitor B [Dasypus novemcinctus]

MDSQGRAASGGCGMREEDKGMLRGGGDDVGLANAAARGQVETVRQLLEAGADPNAVNCYGRRPIQVMMMGNARVAELLLLHGADPNCADPATFTRPVHDAAREGFLDTVLELHRAGAQLDVRDAWGRLPVDLAEERGYHEVVQYLRAAAGD

>XP_036034695.1 cyclin-dependent kinase 4 inhibitor B [Onychomys torridus]

MLGGGSDSGLATAAARGQVETVRQLLEAGADPNAINRFGRRPIQVMMMGSAQVAELLLLHGAEPNCADPATLTRPVHDAAREGFLDTLVVLHRAGARLDVCDAWGRLPVDLAEEQGHRDIARYLHAATGD

>XP_005352780.1 cyclin-dependent kinase 4 inhibitor B [Microtus ochrogaster]

MLGGGSDAGLATAAARGQVETVRQLLEAGADPNAVNRFGRRPIQVMMMGSAQVAELLLLHGAEPNCADPATLTRPVHDAAREGFLDTLVMLHKAGARLDVCDAWGRLPVDLAEEQGHRDIARYLHAASGD

>XP_021056635.1 cyclin-dependent kinase 4 inhibitor B [Mus pahari]

MLGGSSDAGLATAAARGQVETVRQLLEAGADPNAVNRFGRRPIQVMMMGSTQVAELLLLHGAEPNCADPATLTRPVHDAAREGFLDTLVVLHRAGARLDVCDAWGRLPVDLAEEQGHRDIARYLHAATGD

>NP_570825.1 cyclin-dependent kinase 4 inhibitor B [Rattus norvegicus]

MLGGGSDAGLATAAARGQVETVRQLLEAGADPNAVNRFGRRPIQVMMMGSAQVAELLLLHGAEPNCADPATLTRPVHDAAREGFLDTLMVLHKAGARLDVCDAWGRLPVDLAEEQGHRDIARYLHAATGD

>XP_021016070.2 cyclin-dependent kinase 4 inhibitor B [Mus caroli]

MLGGSSDAGLATAAARGQVETVRQLLEAGADPNAVNRFGRRPIQVMMMGSAQVAELLLLHGAEPNCADPATLTRPVHDAAREGFLDTLVVLHRAGARLDVCDAWGRLPVDLAEEQGHRDIARYLHAATGD

>XP_028629188.1 cyclin-dependent kinase 4 inhibitor B [Grammomys surdaster]

MLGGGSDAGLATAAARGQVETVRQLLEAGADPNAVNRFGRRPIQVMMMGSAQVAELLLLHGAEPNCADPATLTRPVHDAAREGFLDTLVVLHRAGARLDVCDAWGRLPVDLAEEQGHRDIARYLHAATGD

>XP_004838408.1 cyclin-dependent kinase 4 inhibitor B [Heterocephalus glaber]

MREEDKSMLSGGGCDVDLANAAARGQVETVRHLLDAGADPNAVNRFGRRPIQVMMMGSAHMAELLLLHGAEPNCADPATLTRPVHDAAREGFIDTLVALHRAGARLDVRDAWGRLPVDLAEEQGHRDIAGYLRAASGD

>XP_037023887.1 cyclin-dependent kinase 4 inhibitor B [Artibeus jamaicensis]

MWEEDKILLGGGSSDAGLTSAAARGQVETVRQLLEAGADPNGVNCFGRRPIQVMMMGSTHIAELLLLHGADPNCADPTTLTRPVHDAAREGFLDTLVALHRAGAHLDVRDAWGRLPVDLAEQRGHLDVARYLCAAAGD

>XP_017829391.1 cyclin-dependent kinase 4 inhibitor B [Callithrix jacchus]

MREENKGMPSGGGSDEDLASAAARGQVEKVRQLLEAGADPNGVNRFGRSAIQVMMMGSARVAELLLLHGAEPNCADPATLTRPVHDAAREGFLDTLVMLHRAGARLDVRDAWGRLPLDLAEERGHCDVAGYLRAAAGD

>XP_039077191.1 cyclin-dependent kinase 4 inhibitor B [Hyaena hyaena]

MREEDKDMLRGGGDGAGLANASARGQVDTVQQLLEAGADPNGVNRFGRRAIQVMMMGSTRVAELLLLHGADPNCADPATLTRPVHDAAREGFLDTLVVLHRAGARLDVRDAWGRLPVDLAEERGHRDVVRYLRAAAGD

>XP_026254322.1 cyclin-dependent kinase 4 inhibitor B [Urocitellus parryii]

MREEDKGMLGGSSSDAGLANAAARGQLEKVRQLLEAGADPNGVNRFGRRPIQVMMMGSAHMAELLLLHGAEPNCADPATLTRPVHDAAREGFLDTLVALHRAGARLDVRDAWGRLPVDLAEELGHREVAEYLRAAAGD

>XP_015993024.1 cyclin-dependent kinase 4 inhibitor B [Rousettus aegyptiacus]

MREEDKGVLGGGSGDAGLASAAARGQVETVRQLLEAGADPNKVNCFGRSPIQVMMMGSARVAELLLLHGADPNCADSATLTRPVHDAAREGFLDTLVALHRAGARLDVRDAWGRLPVDLAEERGHRVVARYLRVAAGD

>XP_041342348.1 cyclin-dependent kinase 4 inhibitor B-like [Pyrgilauda ruficollis]

MGALADELANAAARGDLQRLRELLDGAADPNAANSYGRTPIQVMMLGSPRVAELLLRHGADPNRPDPSTGCLPAHDAARAGFLETLAALHRAGARLDIPDGRGRLPLDVAVGGPHGAVGRYLRDPPPCAQPLPGPSSPQTYCPPPARGTPSGTVLMRCCE

>XP_006908415.1 cyclin-dependent kinase 4 inhibitor B [Pteropus alecto]

MREEDKGVLSGGGGDAGLASAAARGQVEMVRQLLEAGADPNKVNCFGRSPIQVMMMGSARVAELLLLHGADPNCADPATLTRPVHDAAREGFLDTVVALHRAGARLDVRDAWGRLPVDLAEERGHRVVARYLRVAAGD

>XP_030686180.1 cyclin-dependent kinase 4 inhibitor B [Globicephala melas]

MLRSSRPAVDYPGHFAPGGCEVSEEDKGMLSGGGGDAALSNAAARGQVEAVRQLLEAGADPNRLNRFGRRPIQVMMMGSARVAELLLLHGADPNCADPATLTRPVHDAAREGFLDTLVTLHRAGARLDVRDAWGRLPVDLAEERGHRDVARYLRAAEGD

>XP_003995565.1 cyclin-dependent kinase 4 inhibitor B [Felis catus]

MREEDKGMLRGGGDGAGLANASARGQVDTVQQLLEAGADPNGVNRFGRRPIQVMMMGSARVAELLLLHGADPNCADPATLTRPVHDAAREGFLDTLVVLHRAGARLDVRDAWGRLPVDLAEERGHRDVARYLRAAAGD

>XP_010982934.1 cyclin-dependent kinase 4 inhibitor B [Camelus dromedarius]

MLSGGGGDARLANAAARGQVETVRQLLEAGADPNGLSRFGRRPIQVMMMGSVRVAELLLLHGADPNCADPATLTRPVHDAAREGFLDTLVALHRAGARLDVRDAWGRLPVDLAEERGHRDVARYLHTAAGD

>XP_006098184.1 cyclin-dependent kinase 4 inhibitor B [Myotis lucifugus]

MWEEDKVKLGGSGSDAGLASAAARGQVETVRQLLQAGADPNGVNGFGRRPIQVMMMGSTRVAEQLLIHGADPNCADPATLTRPVHDAAREGFLDTLVALHRAGAQLDVRDAWGRLPVDLAEERGHRDIVGYLRATAGD

**>XP_028568980.1 cyclin-dependent kinase inhibitor 2A-like [Podarcis muralis]**

MPEPAQPPGAKENPRALSLPTDPSQLRPRSDGPDGHPRQMHHPLRQAQEAAASPQAPSGEAHPRSPEEPGEEKAAPPFLGADRAEEAMPARRAWNLGPEQVMRMGDPRVAELLLRWGADPNVPDPSTGSCPAHDAAREGFLDTLRVLRSGGARLDLPDRRGRLPVDVAEENGHRHVVRYLARLAIGEPWR

>XP_033029199.1 inversin-B-like isoform X1 [Lacerta agilis]

MPERAQPPGAKENPRARSLPFQRTPLQLRPRRDGPYGHPRQMHHPLRQAQEAAASPQAPSGEAHRRSPEEPGQEKAAPPFLGAYRAEEVVPARRAWILGPEQVMRMGDPHVAQLLLERGADPNVPDPSTGSYPAHDAAHQGFLDTLRMLCSGGARVDLPDRWGRLPVDVAEENGHRHVARYLARLEIWEIWR

>XP_034267152.1 cyclin-dependent kinase inhibitor 2A-like [Pantherophis guttatus]

MTPSDRLSRAAAEGNVAELRRLLDGGSDPNGLNVYGRSAIQVMKLGNPKVAELLLERKANPNVPDPSTGSLPVHDAAREGFLDTLQVLISGGARLDLPNYYGRLPLDEAAENGQNRVVQFLARRQRDNA

>XP_030422749.1 cyclin-dependent kinase 4 inhibitor B-like [Gopherus evgoodei]

MQAGAVAAGGERDLLANAAARGDVQRLRLLLEAGMNPNTVNSFGRTPIQVMMMGNTQVAELLLQRGADPNRPDPRTGSLPVHDAAREGFLDTLVALHRGGARLDLGDKSGRLPIDLAIESGHQQVVSYLRAQPAGNAAPPQA

>XP_028910528.1 cyclin-dependent kinase 4 inhibitor B-like [Ornithorhynchus anatinus]

MQSTKNPSVTAEKLAAAAARGRAKEVRELLEAGADPNVRSRFGRSPIQVMQMGSTEVAELLLQHGAEPNCPDPTTLTQPAHDAAREGFLDTLVLLHEAGARLDVPDAWGRLPVDLALEQGHHHVARYLRAAAGGTADPALNKGPALGSGKEPEGALSFLETRLFQRK

>XP_038625864.1 cyclin-dependent kinase 4 inhibitor B-like [Tachyglossus aculeatus]

MQSTKNLNVTAEKLAAAAARGRAKEVRELLEAGADPNVRSRFGRSPIQVMQMGNTEVAELLLQHGAEPNSPDPTTLTQPAHDAAREGFLDTLVLLHGAGARLDVPDAWGRLPVDLALEQGHHHVARYLRAAAGGTADPVLNKGPALGSGKEPEGALSFLETRVFQRK

>XP_020645991.1 cyclin-dependent kinase 4 inhibitor B-like [Pogona vitticeps]

MLRRAGIDRANGLSSAAARGNVDEARRLLEGGADPNGMNSHGRSAIQVMMMGNPQMAELLLQRGADPNLRDPTTGSFPAHDAARGGFLDTLRVLCQWGARFDLPDYRGRLPLDLAVENGWTQVVAYLSDGRQQGNPAKEEEERRPGV

>XP_029777063.1 cyclin-dependent kinase 4 inhibitor B-like isoform X1 [Suricata suricatta]

MTLLFLSFALLNNPVWRNGRSLARGARIKEGGAGELGRERAGLEAAERQRGGSCLRPCQVMMMGSARVAELLLLHGADPNCADPATLTRPVHDAAREGFLDTLVVLHRAGARLDVRDAWGRLPVDLAEERGHRHVARYLRAAMGGTGSGSHTPIDGVEGPADSWT

>XP_032979295.1 cyclin-dependent kinase 4 inhibitor B-like isoform X3 [Rhinolophus ferrumequinum]

MEDPQQGTRGMKEGKQESGEQKPRKHRGSLLVTSQVMMMGSARVAELLLLHGADPNCADPATLTRPVHDAAREGFLDTLAVLHRAGARLDVRDARGRLPVDLAEERGHRDVARYLREAAGGTEGGGHAHTGRRDDPDLEDHGEPETVSSS

>XP_032979292.1 cyclin-dependent kinase inhibitor 2A-like isoform X1 [Rhinolophus ferrumequinum]

MLSAICKKHGNLMASEKGTDAEQRHFREHRTGLSKSEKEHSFILDTGVFREMEDPQQGTRGMKEGKQESGEQKPRKHRGSLLVTSQVMMMGSARVAELLLLHGADPNCADPATLTRPVHDAAREGFLDTLAVLHRAGARLDVRDARGRLPVDLAEERGHR

DVARYLREAAGGTEGGGHAHTGRRDDPDLEDHGEPETVSSS

>XP_002916935.1 cyclin-dependent kinase 4 inhibitor B [Ailuropoda melanoleuca]

MREEDKGMLGGGGDDAGLANASAQGQVETVRQLLEAGADPNGVNRFGRRPIQVMMMGSTRVAELLLLHGADPNCADPTTLTRPAHDAAREGFLDTLVVLHRAGARLDVRDAWGRLPVDLAEERGHRAVARYLRAAAGD

>XP_026574382.1 cyclin-dependent kinase inhibitor 2A-like [Pseudonaja textilis]

MSGRVKCRVRGVRGERLPRFLQRCLLKVIGKVVRTRPLPHTLVLVVKNRRSPSSRPLQDAEEVMQMSSLDKYPLEPTVRRAFQLQAPCSSSEQPPGFQQIGLAMFDKVVMNLGNPRVAALLLERGANPNVPDPSTGSLPVHDAAREGFLDTLEVLVSGGARLDLRNCYGRLPLDEAAERGQNQVVHFLARRQKDDA

>XP_026528056.1 cyclin-dependent kinase inhibitor 2A-like [Notechis scutatus]

MGGRVKCRVRGVRGERLPRFLQRCLLKVIGKVVRTRPLPHTLELVVKNRRSQTSRPPQDAEEVMQMSSLDKYPLEPTVRRAFQLQAPCSSSEQPPGFQQIGLAMFDKVVMKLGNPRVAALLLERGANPNVPDPSTGSLPVHDAAREGFLDTLQVLVSGGARLDLRNNYGRLPLDEAAEGGQSQVVHFLRRRQRDDA

>XP_031305476.1 cyclin-dependent kinase inhibitor 2A-like isoform X1 [Camelus dromedarius]

MSSPIQVMMMGSVRVAELLLLHGADPNCADPATLTRPVHDAAREGFLDTLVALHRAGARLDVRDAWGRLPVDLAEERGHRDVVRYLRAAARDTGGGSHAHADAAEGPAESPSLKHH

>XP_036187173.1 cyclin-dependent kinase inhibitor 2A-like isoform X3 [Myotis myotis]

MERSADRLAAAAAGGRAEEVQALLQAGASPNAPNSHGRTPIQVMMMGSTRVAELLLIHGADPNCADPATLTRPVHDAAREGFLDTLVALHRAGARLDVRDAWGRLPVDLAEERGHRYVVGYLRREGSAETRG

>NP_999289.1 cyclin-dependent kinase 4 inhibitor B [Sus scrofa]

MLSGGGGDAGLANAAARGQVETVRQLLEAGADPNGLNHFGRRPIQVMMMGSARVAELLLLHGADPNCADPATLTRPVHDAAREGFLDTLVALRRAGARLDVQDAWGRLPVDLAEERGHRDVARFLRAAAGD

>XP_040337316.1 cyclin-dependent kinase 4 inhibitor B [Puma yagouaroundi]

MREEDKGMLRGGGNGAGLANASARGQVDTVQQLLEAGADPNGVNRFGRRPIQVMMMGSARVAELLLLHGADPNCADPATLTRPVHDAAREGFLDTLVVLHRAGARLDVRDAWGRLPVDLAEERGHRDVARYLRAAAGD

>XP_004321986.3 cyclin-dependent kinase 4 inhibitor B [Tursiops truncatus]

MLSGGGGDAALSNAAARGQVEAVRQLLEAGADPNRLNRFGRRPIQVMMMGSARVAELLLLHGADPNCADPATLTRPVHDAAREGFLDTLVALHRAGARLDLRDAWGRLPVDLAEERGHRDVARYLRAAEGD

>XP_025789568.1 cyclin-dependent kinase 4 inhibitor B [Puma concolor]

MREEDKGMLRGGGDGAGLANASARGQVDTVQQLLEAGADPNGVNRFGRRPIQVMMMGSARVAELLLLHGADPNCADPATLTRPVHDAAREGFLDTLVVLHRAGARLDVRDAWGRLPVDLAEERGHRDIARYLRAAAGD

**>XP_031460639.1 cyclin-dependent kinase 4 inhibitor B-like, partial [Phasianus colchicus]**

MAQRAARTAADELANAAARGDLQRLRELLDGAADPNAVNSFGRTPIQVMMLGSPRVAELLLQRGADPNRPDPRTGCRPAHDAARAGFLDTLAALHRAGARLDLP

>XP_027302288.1 cyclin-dependent kinase 4 inhibitor B [Anas platyrhynchos]

MAQRAGSAAADELANAAARGDVQRVRELLDGAADPNAVNSFGRTPIQVMMLGSPRVAELLLQRGADPNRPDPRTGCLPAHDAARAGFLDTLAALHRAGARLDLPDGRGRLPLDVAAGGPHGPVGRFLRQPADGPVS

>XP_021236162.1 cyclin-dependent kinase 4 inhibitor B-like [Numida meleagris]

MAQRAARTAADELANAAARGDLQRVRELLDGAADPNAVNSFGRTPIQVMMLGSPRVAELLLQRGADPNRPDPLTGCRPAHDAARAGFLDTLAALHRAGARLDLPDGRGRLPLDVAAGGPHGPVGCYLRRPPPLPRAPPP

>XP_035169272.1 cyclin-dependent kinase 4 inhibitor B-like [Oxyura jamaicensis]

MAQRAGSAAADELANAAARGDVQRVRELLDGAADPNAVNSFGRTPIQVMMLGSPRVAELLLQRGADPNRPDPRTGCLPAHDAARAGFLDTLAALHRAGARLDLPDGRGRLPLDVAAGGPHGPVGRFLRQPADGPFP

>NP_989764.1 cyclin-dependent kinase 4 inhibitor B [Gallus gallus]

MAQRAASTAADELANAAARGDLLRVKELLDGAADPNAVNSFGRTPIQVMMLGSPRVAELLLQRGADPNRPDPRTGCRPAHDAARAGFLDTLAALHRAGARLDLPDGRGRLPIDVAAGGPHGPVGCYLRRLPALPRAPLP

>XP_015705521.1 cyclin-dependent kinase 4 inhibitor B [Coturnix japonica]

MAQRAARTAADELANAAARGDLQRVRELLDGEADPNAINSFGRTPIQVMMLGSPRVAELLLQRGADPNRPDPRTGCRPAHDAARAGFLDTLETLHRAGARLDLPDSSGRLPLDVAAGGPHGPIGCYLRRLPALPRAPLP

>XP_030325739.1 cyclin-dependent kinase 4 inhibitor B-like [Strigops habroptila]

MEVSLRGDGSGDRLCTAAARGDPQEVRRLLDAGADPNATNSFGRTPLQVMMLGSPRVAELLLQRGADPNRPDPRTGCLPAHDAARAGFLETLAALHRAGARLDLPDGSGRLPVDVAAGGPHGPVARFLRHPPPLPAAAPGAGPAAEGVKR

>XP_032062216.1 cyclin-dependent kinase 4 inhibitor B-like [Aythya fuligula]

MQGPPRGSPLGDRLCSAAARGDLAEVRELLEAGADPNGTNSFGRTPLQVMMLGSPRVAELLLQRGADPNRPDPRTGCLPAHDAARAGFLDTLAALHRAGARLDLPDGRGRLPLDVAAGGPHGPVGLFLRQPPGDPQERDAER

**>XP_023813064.1 cyclin-dependent kinase 4 inhibitor B [Oryzias latipes]**

MHHPFNSNKSRHKPSVCGTSRGCSPRSGSRGRGRPSSRTPPRTRLSELDLGRRTRPGGGESVSGSKLAPSDSSHKHRRAADVLAAAGFGTIMTLQDDLTTAAAKGNAAEVERCLSAGAEVNGRNRFGRTAVQVMMMGSTAVAQVLLRHGADPNVADSSTGATPLHDAARTGFLDTVQLLVEAGADPQAEDREGRSPGDVARQNGHADVAAFLSPGLLNRPEAAHKLN

>XP_024145719.1 cyclin-dependent kinase 4 inhibitor B [Oryzias melastigma]

MTLEDELTTAAAKGNAAEVEERLAAGAQVNGRNRFGRTALQVMMMGSAAVAQVLLRHGADPNVADQTTGATPLHDAARTGFLDTVRLLVQAGADPQARDKAGRSPGDVARQDGHADVAAFLQRLTAPAD

>XP_029950286.1 cyclin-dependent kinase 4 inhibitor B [Salarias fasciatus]

MAATLEDDLTSAAAKGNAADVEDLLLAGAEVNGANRFGRTALQVMMMGSTPVAQVLLKHGADPNVADRSTGATPLHDAARTGFLDTVRLLVHFGADPRARDHANDVPADLAAQHGHEDVASFLQTL

>XP_039653727.1 cyclin-dependent kinase 4 inhibitor B [Perca fluviatilis]

MTLEDDLTTAAAMGNTADVEYLLRAGAEVNGVNCFGRTALQVMMMGSTPVAQLLLKHGADPNVADTSTGTTPLHDAARTGFLDTVRLLVQFLADPQTKDNTNNRAIDLARQNGHLDVVDFLQSL

>XP_029994441.1 cyclin-dependent kinase 4 inhibitor B [Sphaeramia orbicularis]

MPLEDDLTTAAANGDTARVESLLQTGAQVNGLNRFGRTAVQVMMMGSSSVAQVLLKHGADPNVADPRTGSTPLHDAARTGFLDTVRLLVEYTADPEATDNTNCRPVDVAKDNGHADVVAFLESL

>XP_028452333.1 cyclin-dependent kinase 4 inhibitor B-like [Perca flavescens]

MTLEDDLTTAAAMGNTADVEYLLRAGAEVNGVNCFGRTALQVMMMGSTPVAQLLLKHGADPNVADRSTGTTPLHDAARTGFLDTVRLLVQFLADPKTKDKTNNRAIDLARQNGHLDVVDFLQSR

>XP_032385257.1 cyclin-dependent kinase 4 inhibitor B [Etheostoma spectabile]

MTLEDDLTTAAARGNTADVEYLLRAGAEVNGENCFGRTALQVMMMGSTPVAQLLLKHGADPNVGDTSTGATPLHDAARAGFLDTVRLLVQFLADPQTKDNTNNRAIDLARENGHEDVVDFLQSLEK

>ACB30547.1 cell cycle inhibitor p15 [Nothobranchius kuhntae]

MTMVTLQDDLTTAAAKGNTADVESLLRRGAAVNGVNRFGRTAVQVMMMGSWPVARMLLQHGADPNVADRSTRTTPLHDAARTGFVDTVRLLVAAQADPQARDNQNRLPVDLARENDHAEVVAFLETLN

>XP_031132698.1 cyclin-dependent kinase 4 inhibitor B [Sander lucioperca]

MTLEDDLTTAAAMGNTADVEYLLRAGAEVNGVNCFGRTALQVMMMGSTPVAQLLLKHGADPNVADTSTGTTPLHDAARTGFVDTVRLLVKFQADPQTRDNTNNRAIDLARENGHRDVIDFLQSL

>XP_022621127.1 cyclin-dependent kinase 4 inhibitor D-like [Seriola dumerili]

MTLVDELATAAAKGNTADVEYLLQEGAQVNGVNCFGRTALQVMMMGSTPVAKVLLKYGADPNVADTSTGTTPLHDAARTGFVDTVRLLVEYLADPQARDNTNCRPIDLARANGHEDVVAFLESL

>XP_010739241.2 cyclin-dependent kinase 4 inhibitor B [Larimichthys crocea]

MTLEDDLSTAAAKGNREDVEDLLQAGAQVNGVNCFGRTALQVMMMGSWPVAQLLLKHGADPNLADCSTGTTPLHDAARTGFLETVQLLVQHMADPQARDKTNCRPVDLARDNGHTEVVAFLDSL

>TKS68292.1 Cyclin-dependent kinase 4 inhibitor B [Collichthys lucidus]

MTLEDDLSTAAAKGNREDVEDLLRAGAEVNGLNCYGRTALQVMMMGSWPVAQLLLKHGADPNVPDCSTGTTPLHDAARAGFLETVRLLVEYMADPQARDKTNCQPVDLARDNGHTEVVAFLESL

>XP_037830664.1 cyclin-dependent kinase 4 inhibitor B [Kryptolebias marmoratus]

MTLQDDLTTAAAKGDAAGVEALLRKGADVNVENSFGRRAIQVMMMGSTAVASILLRHQADPNVADRSTGTSPLHDAAREGFTDTVKLLLQAQADPRARDSNGRLPADLALENGHTDIAELLQSV

>XP_020457704.1 cyclin-dependent kinase 4 inhibitor B-like [Monopterus albus]

MTLEDELTSAAAQGHTAEVSRLLQAGAQVNGVNRFGRSAVQVMMMGSTPVAEVLLRHGADPNVADRRTGATPLHDAARAGFLDTVRLLVNYKADPRAKDKANCQPIDLARKSGLTDVVAYLECVLSGNSGL

>XP_034750158.1 cyclin-dependent kinase 4 inhibitor B [Etheostoma cragini]

MTLEDDLTTAAAMGNTADVEYLLRAGAEVNGENCFGRTALQVMMMGSTPVAHLLLKHGADPNVGDPSTGTTPLHDAARAGFLDTVRLLVQFLADPQTKDNANNRAIDLARENGHEDVVDFLQSL

>XP_040002608.1 cyclin-dependent kinase inhibitor 2A [Xiphias gladius]

MTLGDDLTTAAAKGNTADVECLLQAGAEVNGVNCFGQTALQVMMMGSTPVAKVLLKYGGDPNVADRSTGATPLHDAARAGFLDTVRLLVEYLADPEARDNTNCRPIDVARGNGHEGVVAFLESL

>XP_023282883.1 cyclin-dependent kinase 4 inhibitor D-like [Seriola lalandi dorsalis]

MILADELATAAAKGNTADVECLLQEGAQVNGVNCFGRTALQVMMMGSTPVAKVLLKYGADPNVADTSTGTTPLHDAARTGFVDTVRLLVEYQAEPQAKDNTNCRPIDLARANGHEDVVAFLESL

>XP_038569207.1 cyclin-dependent kinase 4 inhibitor B [Micropterus salmoides]

MTLQDALTTAAANGQAAEVKNLLQAGAQVNGVNRFGRTALQVMMMASTPVAQVLLEHGADPNVADRSTGATPLHDAARTGFLDTVRLLVEKQADPWARDNAKCLAVDLARNEGNTDVVAFLESLKPPL

>XP_040891512.1 cyclin-dependent kinase 4 inhibitor B [Toxotes jaculatrix]

MTLEDDLTTAAAKGNTEEVETLLGKGAQVNGLNCFERTALQVMMMGSTPVAEVLLKHGADPNLPDKNTGATPLHDAAMTGFLDTVRLLVACGANPQATDNTNCRPIDLARKNGHGEVVDFLESL

>XP_026159489.1 cyclin-dependent kinase 4 inhibitor B-like [Mastacembelus armatus]

MTMTLEDDLTKAAANGNTADVEDLLKAGAEVNGVNCFGCTALQVMMMGSTPVAQLLLKHGGDPNVADRHTGTTPLHDAARTGFLDTVRLLVNYRADPQARDNKNCQPIDLARKNDHTNVVAFLESLQ

>XP_029290706.1 cyclin-dependent kinase 4 inhibitor B-like [Cottoperca gobio]

MNLEDDLTKAAAMGNTAAVEDLLRAGAEVNGVNSLGRTALQVMMMGSTSVAQVLLKHGADPNVADRSTGGTPLHDAARTGFLDTVQLLVQFLADPHARDNKNNRPIDLARDKGNTDVVDFLEAIKHDAVICMC

>XP_035516165.1 cyclin-dependent kinase 4 inhibitor B [Morone saxatilis]

MTLEDDLTAAAAKGHTAGVKSLLQAGAEVNGGNCFGRSAIQVMMMGSTPVAQVLLEHGADPNVADPSTGTTPLHDAARTGFLDTVRLLVRYKADPQARDNTNCLPIDLARDNGHRYVVDFLECIN

>XP_030594233.1 cyclin-dependent kinase 4 inhibitor B-like [Archocentrus centrarchus]

MTLEDELTSAAAKGNTADVRALLEKGAQVNGNNRFGQSALQVMMMGSTSVAQLLLDHGADPNVADNSTGTTPLHDAAREGFLDTVRLLERHHADPQAKDKLNRLPIDLARLRGHGDVVNFLESLQNGYNP

>XP_036957368.1 cyclin-dependent kinase 4 inhibitor B [Acanthopagrus latus]

MTLEDDLTTAAATGNTADVENLLRAGAEVNGPNCFGRTALQVMMMGSTSVAKVLLNHGADPNVADSSTGTTPLHDAARTGYLDTVRLLVEYQADPQARDNANCRPVDLAGEHGHADVVAFLESV

>XP_034086070.1 cyclin-dependent kinase 4 inhibitor B [Gymnodraco acuticeps]

MTLEDELTKAAANGNTATVEDLLRAGAQVNGVNSLGHTALQVMMMGSSPVAQLLLRHGADPAVSDRSTGSSPLHDAARTGFVDTVRLLVKNRADPQARDNKDNRPVDLARQHGHTEVEDYLQSLPDTE

>XP_033986063.1 cyclin-dependent kinase 4 inhibitor B [Trematomus bernacchii]

MTLEDELTKAAATGNTATVEDLLRAGAQVNGVNSLGHTALQVMMMGSSSVAQLLLRHGADPAVRDRSTGSSPLHDAARTGFVDTVRLLVKNRADPQARDNKDNRPVDLARQHGHTEVEGYLQSLPDTE

>XP_035493112.1 cyclin-dependent kinase 4 inhibitor B [Scophthalmus maximus]

METTPPPPPPTTTTATTMTVADELTSAAAKGRTADVELLLRGGAEVNGLNRFGRTALQVMMMGSTPVARVLLKHGADPNVADGRTGSSPLHDAAREGFVDTARLLVEHLADPQARDNAKCRPVDLARDNGHDDVVAFLESL

>XP_030289212.1 cyclin-dependent kinase 4 inhibitor B [Sparus aurata]

MTLEDDLTTAAATGNTADVEDLLRAGAEVNGPNCFGRTALQVMMMGSTPVAKVLLNHGADPNVADSSTGTTPLHDAARTGYLDTVRLLVEYQADPQARDNANCRPVDLAAEHGHADVVAFLECV

>XP_029913838.1 cyclin-dependent kinase 4 inhibitor B [Myripristis murdjan]

MTMTLEDDLASAAARGNTEEVEDLLRRGVDVNRLNSFGRTPLQVMMMGSTPVAQLLLRRGADPNVADRCTGATPLHDAARTGFVDTVRLLVQHNGDPQARDNKNCRPVDLAREQGHKDVVAFLESL

>XP_006787175.1 cyclin-dependent kinase 4 inhibitor B [Neolamprologus brichardi]

MDVTMTLQDELTTAAAKGNTAAAEALLNRGAQVNGTNSFGRTALQVMMMGSTSVAQLLLEHGANPNVGDSSTGASPLHDAARTGFLDTVHLLVQHHADPQARDKLNRLPVDLARQHGHGDVVDFLENLQNP

>XP_034043987.1 cyclin-dependent kinase 4 inhibitor B [Thalassophryne amazonica]

MTSEDDLTAAAATGQREEVERLLRAGARVNGPNRFGRTAIQVVMMGSTSVVRFLLESGADPNVADRSTGTTPLHDAARTGFVDTVRLLVQFGADPQATDNHHHRPVDLAREHGRVEVVDFLQSL

>XP_012737657.2 cyclin-dependent kinase 4 inhibitor B [Fundulus heteroclitus]

MTPQDELTGAAARGDAASVKALLQSGAPVNGENCFGRTPLQVMMMGSSRVARLLLELGADPNVADRSTKATPLHDAARAGFLDTVRLLVEAGADPRARDNADCLPVDLARQNGHSDVLAFLETL

>XP_028260587.1 cyclin-dependent kinase 4 inhibitor B [Parambassis ranga]

MTLEDELTAAAARGDAAGVEDLLRAGAPVNGVNCFGRTALQVMMMGSARVARILLRHGADPNVSDSTGATPLHDAARTGFLDTVRLLLEFQADPQARDNADRLPVDLARQNGHADVVAFLETL

>XP_033943227.1 cyclin-dependent kinase 4 inhibitor B [Pseudochaenichthys georgianus]

MHDTHTHTHTHTHAHTPTYTETTCGVDTHIIRGTSTHQVLFTRPVHYTGVFWNRYLSEPASDRSTDGVAAGFRTMTLEDELTKAAANGNTATVENLLRAGAQVNGVNSLGYTALQVMMMGSSSVAQLLLRHGADPAVRDRSTGSSPLHDAARTGFVDTVRLLVKNRADPQARDNKDNRPVDLARQHGHTEVEDYLQSLPDTE

>RXN30151.1 Cyclin-dependent kinase 4 inhibitor B [Labeo rohita]

MWDAKFFSGLIRKMMHPEDELTKAAATGNTYHVQFLLSNGVNVNGVNKFGRTPIQVMMMGNTPLAHLLLEYGADPNVADPGTGSTPLHDAARTGFMDTVQLLIRFNADPNATDHYNLRPLDVARQTGHVDVVEFLSRI

>XP_020504082.1 cyclin-dependent kinase 4 inhibitor B-like [Labrus bergylta]

MTMTLEDELTAAAAKGNTADVEDLLRKGAQVNGLNHFGRSALQVMMMGSTPVARLLLTHGANPNVADRSTEHTPLHDAARMGFLDTVKLLVEFKANPQARDKDNLRPRDLAGKYGHTNVVDFLDSLE

>XP_037128435.1 cyclin-dependent kinase 4 inhibitor B [Syngnathus acus]

MGILEDELTSAAATGNTAEVKRLLRAGADVNGANRLGYTALQVMMMGSTPVAHLLLEGGGDPNVADSSTGSTPLHDAARTGFSDTARLLVRFAASPQARDHSNCRPVDVARRYGHEDVVAFLKSL

>XP_029368745.1 cyclin-dependent kinase 4 inhibitor D-like [Echeneis naucrates]

MTPVDELTTAAANGNRADVERLLREKVQVNGVNCFGRTALQVMMMGSTPVAQLLLEQGADPNVADVRTRTTPLHDAARTGFLDTVRLLVEYKADPQARDLANCRPVDLARQNHHKSVVDFLESLEN

>XP_029004798.1 cyclin-dependent kinase 4 inhibitor B [Betta splendens]

MSVDDLTAAAATGRTADVERLLRAGAEVNGANRFGRTALQVMMMGSSPVARVLLSHGANPNVADRSTGTTPLHDAARTGFLDTVRLLVQHGADPRASDKTGCRPVDLARQNHHNEVLDFLKSLENPV

>XP_026060223.1 cyclin-dependent kinase inhibitor 2A-like [Carassius auratus]

MRKMMHVEDELTKAAATGNTYRVQFLLSNGVNVNGVNKFGRTPIQVMMMGNTPLAHLLLEYGADPNVPDPGTGSTPLHDAARTGFMDTVQLLIRFNADPNSTDHSNLRPVDVARRTGHMDVAEFLSRI

>XP_040043194.1 cyclin-dependent kinase 4 inhibitor B [Gasterosteus aculeatus aculeatus]

MTLEDELATAAATGNTADVEDLLQAGASVNGANRFGRTALQVMMMGSTPVALLLLKHGADPNAADRSTGATPLHDAARAGFLDTVRLLVQYRAEPQARDNRARQPVDLAGENNHVDVVNFLQSLPN

>XP_034389970.1 cyclin-dependent kinase inhibitor 2A [Cyclopterus lumpus]

MDMTLQDKLTTAAATGNTAEVEDLLRAGAQVDGENCFGRTPLQVMMMGSTPVARVLLNHGAAPNVRDRSTGTTPLHDAARAGFVDTVRLLVKCGADPQARDNTNYQPIDLARENGHVDVVVFLQCL

>XP_037327316.1 cyclin-dependent kinase 4 inhibitor B [Pungitius pungitius]

MHTVTHPNRDELRPGRTGRAHGTRSDRFPDVDAAGSLDMTLEDELTTAAATGNTAEVTTLLQDGASVNGANRFGRTALQVMMMGSSQVAQVLLKHGADPNVTDRSSGATPLHDAARTGFLDTVRLLVEFGADPRARDNRQCQPVDLARANNDVDVVNFLQSLSNGQ

>XP_037619996.1 cyclin-dependent kinase 4 inhibitor B [Sebastes umbrosus]

MTLEDKLTTAAATGNTAEVEDLLRAGAPVNGLNCYGRTALQVMMMGSSPVAQVLLTHGADPSVTDGSTGATPLHDAARTGFLDTVRLLVQFLADPQAKDNTNNRPIDLARDSGHTDVVDFLQAL

>XP_026215616.1 cyclin-dependent kinase 4 inhibitor B [Anabas testudineus]

MSVADKLTTAAAKGSAAAVEELLRAGAEVNGLNCFGRTALQVMMMGSTPVALLLLKHGANPNVAEGGTGTTPLHDAARTGFLDTVRLLVEHGADPQARDSRNRRPVDLATENDHGDVEDFLRSLENTV

>XP_034448264.1 cyclin-dependent kinase 4 inhibitor B [Hippoglossus hippoglossus]

MVLEDELSTAAANGRTAAVDGLLQAGADVNGLNRFGRRALQVMMMGSEAVAQLLLGHGADPNVADSSTGSTPLHDAARTGHVGTVRLLVEHQADPEARDNKQRRAVDLAEEYGNKDVVAFLENL

>XP_031727286.1 cyclin-dependent kinase 4 inhibitor D-like [Anarrhichthys ocellatus]

MTLQDKLTTAAATGKTADVEELLREGAQVNGVNSFGRTALQVMMMGSTPVAQLLLEHGADPNVTDRSTGTTPLHDAAGTGFLDTARLLVQFRADPQATDNNSCQPIDLARENGHVEVVDFLQSQ

>XP_035272764.1 cyclin-dependent kinase 4 inhibitor B [Anguilla anguilla]

MIREDELASAAATGNVDHVGILLQAGVDVNAVNRFGRTPLQVMMMGSTRVAQLLLKHGANPNLSDESTGDTPLHDAARGGFLDTVKMLIEFNADIRAQDYRSRRPVDLARENGHLEVVTFLESL

>XP_003972435.1 cyclin-dependent kinase 4 inhibitor B [Takifugu rubripes]

MPLEDELTAAAAKGDAAQVRSLLGAGAQVNGVNCFGRTALQVMMMGSTRVAQILLDHGADPNVADGTTGATPLHDAARSGFLDTVRLLVRFTADPNARDQADRRPVDLARDECHTDVVAFLESL

>XP_026852078.1 cyclin-dependent kinase 4 inhibitor B [Electrophorus electricus]

MHDKDKLATAAANGNVELVESLLLNGADVNGVNRFGRTPIQVMMMGSTPVARVLLAHGADPNVVDAHAGATPLHDAARTGFLDTVKILIQFGANPDVGDNNNYRPVDLAQQNGHQEKTSKVSRVALFRSFAHYPVIPAFIPSYLK

>XP_033828553.1 cyclin-dependent kinase 4 inhibitor D [Periophthalmus magnuspinnatus]

MTLEDDLTTAAATGNAAAVEYLLETGAHVNGLNRFGRTALQVMMMGSLSVAQTLLKHGADPNVRDRSTGATPLHDAVRTGFVDTARLLIEFQADPNLRDNKNLRPIDIAKLGGHEEVFAFLETE

>TWW58701.1 Cyclin-dependent kinase 4 inhibitor B [Takifugu flavidus]

MPLEDELTAAAAKGDAAQVRSLLGAGAQVNGVNCFGRTALQVMMMGSTRVAQILLDHGADPNVADGTTGTTPLHDAARSGFLDTVRLLVRFTADPNARDQADRRPVDLARDECHTDVVAFLESL

>XP_030644032.1 cyclin-dependent kinase 4 inhibitor B-like [Chanos chanos]

MHPEDELSSAAATGNTERVRLLLQNGANVNSINTFGRTPIQVMMMGSTPVAQLLLDHGADPNVSDESTGATPLHDTARLGFLETVRILVQHHADPNATDNRNCRPADLARENGFLDVAEFLDNV

>KAA0722300.1 Cyclin-dependent kinase inhibitor 2A [Triplophysa tibetana]

MMYDAEELTKAAATGNTVRVQFLLSEGVNVNSFNRFGRTPIQVMMMGNTPLAHLLLEHGADPNLPDPGTGSTPLHDAARTGFLDTLKLLIHFSADPKATDNNNLRPVDVAEQTGNLDVVEFLNRF

>XP_024000147.1 cyclin-dependent kinase 4 inhibitor B-like [Salvelinus alpinus]

MTMPLEDDLASAAATGNTDRVKILLQSGVDVNGVNCFGRTPLQVMMMGSSPVAQALLMKGADPNIADRHTGTTPLHDAARMGFLDTVEILVQFLADPNSRDNRNCRPIDLARESGHHNVVAFLQAL

>XP_029106443.1 cyclin-dependent kinase 4 inhibitor B-like [Scleropages formosus]

MDPADELTSAAATGNAELVRTLLESGVAANGLNRFGRTALQVMMMGSVPVARLLLSHGADPNLGDPLTGSTPLHDAARLGFLDTVRLLVRFGARVHATDRNIQRPVDLAQQNGHVDVEAFLRSCRM

>NP_001297888.1 cyclin-dependent kinase inhibitor 2A/B (p15, inhibits CDK4) [Esox lucius]

MPLEDDLASAAATGNTDRVKMLLERGMDVNGANCFGRTPLQVMMMGSTTVARLLLQQGADPNVADRHTGTMPLHDAARMGFLDTVKILVQCNANPNTLDNWNRRPIDLAKESGHQNVVEFLQTL

>XP_038837633.1 cyclin-dependent kinase 4 inhibitor B [Salvelinus namaycush]

MTMPLEDDLASAAATGNTDRVKILLQSGVDVNGVNCFGRTPLQVMMMGSSPVAKLLLMKGADPNIADRHTGTTPLHDAARMGFLDTVEILVQFLADPNSRDNRNCRPIDLARESGHHNVVAFLQAL

>XP_033479439.1 cyclin-dependent kinase 4 inhibitor D [Epinephelus lanceolatus]

MLEEIVPTVDDLTTAAARGDSNRVDTLLWAGVDPNGCNVFGRTALQVMMMGSTTVARSLLRAGADPNLKDISTGATPLHDAARTGFLDTVKALVEHGADIQARDNADRRPIDLATENEHSSTADYLTSLENPQ

**>ETE67547.1 Cyclin-dependent kinase inhibitor 2A, partial [Ophiophagus hannah]**

MKLEIGSRGPCVESCLTTPLPSFSSLQVMKLGNPRVATLLLERGANPNVPDPSTGSLPVHDAAREGFLDTLQVLVSGGARLDLPNYYGRLPLDEAAETTEGRRLEADSAGRCWSGTSRAASHFQLLRFSAALRCAALAPSHNFLKMRVNSIRQLEISSAC

>XP_034953283.1 cyclin-dependent kinase 4 inhibitor B-like isoform X1 [Zootoca vivipara]

MHHPLRQAQEAAASPQAPSGEAHPRSPEEPGQEKAAPPFLGADRAEEAMPARRAWILGPEQVMKLGDPRVAELLLERGADPNMPDPSTGSCPAHDAAREGFLDTLRVLRSGGARFDLPDRGGRLPVDVAEENGHRHVTRYLARPCGRSGKPGDNPATI

>XP_033775341.1 cyclin-dependent kinase 4 inhibitor B-like [Geotrypetes seraphini]

MEDEAVNDNRADALTRAAACGDLLRVQSLLESGTDPNETNSFHRTAIQVMKLGNPKLAELLLLYGADPNVPDPTTNTYPVHDAAREGFLDTLQVLVRGGACLDQCDRWGQLPIDVALYPNQLRNLLDQGCQTQSH

>XP_030323879.1 cyclin-dependent kinase 4 inhibitor B-like isoform X1 [Calypte anna]

MEGFPWGEGPSDRLCCAAARGDREEVRRLLEARVDPNGTNSFGRTPLQVMMLGSPRVAELLLQRGADPNRPDPRTGCLPVHDAARTGFLETLAVLHRAGARLDLPDGRGRLPLDVAAGGPHGPVGRYLRQPPPLPAAPPRAAGPPAEGDGC

>XP_029861536.1 cyclin-dependent kinase 4 inhibitor B-like isoform X2 [Aquila chrysaetos chrysaetos]

MQGSPRSEGSGDRLCSAAACGDREEVRKLLDAGADPNGTNSFGRTPLQVMMLGSPRVAELLLQRGADPNRPDPRTGSLPAHDAARAGFLETLAALHRAGARLDLPDGRGRLPLDVAAGGPHGPVGRYLRHPPPVPAAAPGAGTAAEAAAP

>XP_032940852.1 cyclin-dependent kinase 4 inhibitor B-like [Catharus ustulatus]

MEGSLRSDGDRLCSAAARGDHEEVRRLLDAGVDPNGTNAFGRTPLQVMMLGSPRVAELLLRRGADPNRPDPHTGCLPVHDAARAGFLETLAALHRAGARLDLPDGRGRLPLDVAAGGPDGAVGRYLRHPPPLPGAEKGGPEKAVR

**>ETE67546.1 Cyclin-dependent kinase 4 inhibitor B [Ophiophagus hannah]**

MEAPNGDGCQANQLANAAARGDLETAARLLENGADPNATNAFGRSPIQVMMMGSSKMAELLLQRGADPNRPDPSTGAMPAHDVAQEGFLDTLKVLHHWGARFDHLDRWGRSPLDLARKNGQNHVVDYLQELPG

>XP_040436649.1 cyclin-dependent kinase 4 inhibitor B-like isoform X3 [Falco naumanni]

MEASPRGGGCAGDRLCSAAARGDREEVRKLLDAGADPNGTNCFGRTSLQVMMLGSPRVAELLLQRGADPNRPDPSTGCFPVHDAASAGFLETLEALHRAGARLDLPDGFGRLPLDVAAGGPHGPVGRYLRHPPPLHAAAPGDGPAAEGAVHWHARGSRPCWPPSPPSRAAPPEAPVAAIPRQDLLPPCRARISAEGTGQVLPAVEAPSER

**>NP_031696.1 cyclin-dependent kinase 4 inhibitor B [Mus musculus]**

MLGGSSDAGLATAAARGQVETVRQLLEAGADPNALNRFGRRPIQVMMMGSAQVAELLLLHGAEPNCADPATLTRPVHDAAREGFLDTLVVLHRAGARLDVCDAWGRLPVDLAEEQGHRDIARYLHAATGD

>XP_031233693.1 cyclin-dependent kinase 4 inhibitor B isoform X2 [Mastomys coucha]

MLGGGSDAGLATAAARGQVETVRQLLEAGADPNAVNRFGRRPIQVMMMGSAQVAELLLLHGAEPNCADPATLTRPVHDAAREGFLDTLVVLHRAGARLDVCDAWGRLPVDLAEEQGHRDIASPI

>XP_006167080.1 cyclin-dependent kinase 4 inhibitor B [Tupaia chinensis]

MREEDKSMLGGGGSDAGLASAAARGQVEKVRQLLEVGADPNGVNRFGRRPIQVMMMGSTRVAELLLLHGAEPNCADPATLTQPVHDAAREGFLDTRVALHRAGARLDVRDAWGRLPVDLAEERGHRDVARYLHAATGD

>XP_007124394.1 cyclin-dependent kinase 4 inhibitor B isoform X1 [Physeter catodon]

MLSGGGGDAGLSNAAARGQVEAVRQLLEAGADPNRLNRFGRRPIQVMMMGSARVAELLLLHGADPNCADPATLTRPVHDAAREGFLDTLVALHRAGARLDVRDAWGRLPVDLAEERGHRDVARYLRAAEGD

>XP_027786841.1 cyclin-dependent kinase 4 inhibitor B [Marmota flaviventris]

MREEDKGMLGGSSIDAGLANAAARGQLEKVRQLLEAGADPNGVNRFGRRPIQVMMMGSAHVAELLLLHGAEPNCADPATLTRPVHDAAREGFLDTLVALHRAGARLDVRDAWGRLPVDLAEELGHREVAEYLRAAAGD

>XP_007176885.1 cyclin-dependent kinase 4 inhibitor B isoform X1 [Balaenoptera acutorostrata scammoni]

MLSGGGGDAGLSNAAARGQVEAVRQLLEAGADPNRLNRFGRRPIQVMMMGSARVAELLLLHGADPNCADPATLTRPVHDAAREGFLDTLAALHRAGARLDVRDAWGRLPVDLAEERGHRDVARYLRAAEGD

>XP_024414159.1 cyclin-dependent kinase 4 inhibitor B [Desmodus rotundus]

MWEEDKILLGGGSSDAGLTSAAARGQVETVRQLLEAGADPNGVNRFGRRPIQVMMMGSTRIAELLLLHGADPNCADPATLNRPVHDAAREGFLDTLVALHRAGARLDVRDAWGRLPMDLAEQQGHLDVARYLRAAAGD

>XP_026944791.1 cyclin-dependent kinase 4 inhibitor B [Lagenorhynchus obliquidens]

MLSGGGGDAALSNAAARGQVEAVRQLLEAGADPNRLNRFGRRPIQVMMMGSARVAELLLLHGADPNCADPATLTRPVHDAAREGFLDTLVTLHRAGARLDVRDAWGRLPVDLAEERGHRDVARYLRAAEGD

>XP_004275096.1 cyclin-dependent kinase 4 inhibitor B [Orcinus orca]

MLSGGGGDAALSNAAARGQVEAVRQLLEAGADPNRLNRFGRRPIQVMMMGSARVAELLLLHGADPNCADPATLTRPVHDAAREGFLDTLVALHRAGARLDVRDAWGRLPVDLAEERGHRDVARYLRAAEGD

>XP_003260408.1 cyclin-dependent kinase 4 inhibitor B isoform X1 [Nomascus leucogenys]

MREENKGMPSGGGSDEGLASAAARGLVEKVRQLLEAGADPNGVNRFGRRAIQVMMMGSARVAELLLLHGAEPNCADPATLTRPVHDAAREGFLDTLVVLHRAGARLDVRDAWGRLPVDLAEERGHRDVAGYLRAATGD

>XP_005335975.1 cyclin-dependent kinase 4 inhibitor B [Ictidomys tridecemlineatus]

MREEDKGMLGGNSSDAGLANAAARGQLEKVRQLLEAGADPNGVNRFGRRPIQVMMMGSTHVAELLLLHGAEPNCADPATLTRPVHDAAREGFLDTLVALHRAGARLDVRDAWGRLPVDLAEELGHREVAEYLRAAAGD

>XP_036710651.1 cyclin-dependent kinase 4 inhibitor B isoform X2 [Balaenoptera musculus]

MLSGGGGDVGLSNAAARGQVEAVRQLLEAGADPNRLNRFGRRPIQVMMMGSARVAELLLLHGADPNCADPATLTRPVHDAAREGFLDTLAALHRAGARLDVRDAWGRLPVDLAEERGHRDVARYLRAAEGD

>XP_008055781.1 cyclin-dependent kinase 4 inhibitor B [Carlito syrichta]

MREGSKGMLRGGGGETGLANAAARGQVEKVRQLLEAGADPNGVNRFGRRAIQVMMMGSARVAELLLLHGAEPNCADPATLTRPVHDAAREGFLDTLVALHRAGARLDVRDAWDRLPVDLAEERGHRDVARYLHAAAGD

>XP_001107263.1 cyclin-dependent kinase 4 inhibitor B [Macaca mulatta]

MREENKGMPSGGGSDEGLTSAAARGLVEKVRQLLEAGADPNGVNRFGRRAIQVMMMGSARVAELLLLHGAEPNCADPATLTRPVHDAAREGFLDTLVVLHRAGARLDVRDAWGRLPVDLAEERGHRDVAGYLRAATGD

>XP_031993930.1 cyclin-dependent kinase 4 inhibitor B [Hylobates moloch]

MREENKGMPSGGGSDEGLASAAARGLVEKVRQLLEAGADPNGVNRFGRRAIQVMMMGSTRVAELLLLHGAEPNCADPATLTRPVHDAAREGFLDTLVVLHRAGARLDVRDAWGRLPVDLAEERGHRDVAGYLRAATGD

>XP_010351689.1 cyclin-dependent kinase 4 inhibitor B [Rhinopithecus roxellana]

MREENKGMPSGGDSDEGLTSAAARGLVEKVRQLLEAGADPNGVNRFGRRAIQVMMMGSARVAELLLLHGAEPNCADPATLTRPVHDAAREGFLDTLVVLHRAGARLDVRDAWGRLPVDLAEERGHRDVAGYLRAATGD

>XP_024623596.1 cyclin-dependent kinase 4 inhibitor B [Neophocaena asiaeorientalis asiaeorientalis]

MLSGGGGDAALSNAAARGQVEAVRQLLEAGADPNRLNRFGRRPIQVMMMGSARVAELLLLHGADPNCADPATLTRPVHDAAREGFLDTLVALHRAGARLDVRDAWGRLPVDLAEERGHRDVARYLRTAEGD

>XP_012625236.1 cyclin-dependent kinase 4 inhibitor B [Microcebus murinus]

MREENKGMPSGGGGDAGLASAAARGQVEKVRQLLEAGADPNGVNRFGRRAIQVMMMGSVRVAELLLLHGAEPNCADPATLTRPVHDAAREGFLDTLGALHRAGARLDVRDAWGRLPVDLAEERGHRDVARYLRAAAGD

>XP_033093379.1 cyclin-dependent kinase 4 inhibitor B [Trachypithecus francoisi]

MREENKGMPSGGGSDEGLTSAAARGLVEKVRQLLEAGADPNGVNRFGRRAIQVMMMGSARVAELLLLHGAEPNCADPATLTRPVHDAAREGFLDTLMVLHRAGARLDVRDAWGRLPVDLAEERGHRDVAGYLRAATGD

>XP_002819780.1 cyclin-dependent kinase 4 inhibitor B [Pongo abelii]

MREENKGMPSGGGDEGLASAAARGLVEKVRQLLEAGADPNGVNRFGRRAIQVMMMGSARVAELLLLHGAEPNCADPATLTRPVHDAAREGFLDTLVVLHRAGARLDVRDAWGRLPVDLAEERGHRDVAGYLRAATGD

>XP_030150305.1 cyclin-dependent kinase 4 inhibitor B [Lynx canadensis]

MREEDKGMLRGGGDGAGLANASARGQVDTVQQLLEAGADPNGVNRFGRRPIQVMMMGSARVAELLLLHGADPNCADPATLTRPVHDAAREGFLDTLVVLHRAGARLDVRDAWGRLPVALAEERGHRDVARYLRAAAGD

>XP_004373581.1 cyclin-dependent kinase 4 inhibitor B [Trichechus manatus latirostris]

MREEDKGMLGDGSGDAGLANAAARGQVETVRQLLEAGADANRVNCFGRRPIQVMMMGSTPVAELLLLHGAEPNCADPVTLTRPVHDAAREGFLDMLMVLHRAGARLDVRDAWGRLPVDLAEERGHRDVARYLRAATGD

>XP_004047927.1 cyclin-dependent kinase 4 inhibitor B [Gorilla gorilla gorilla]

MREENKGMPSGGGSDEGLASAAARGLVEKVRQLLEAGADPNGVNRFGRRAIQVMMMGSARVAELLLLHGAEPNCADPATLTRPVHDAAREGFLDTLVVLHRAGARLDVRDAWGRLPVDLAEERGHHDVAGYLRTATGD

>NP_001069362.1 cyclin-dependent kinase 4 inhibitor B [Bos taurus]

MLSGGGGDADLANAAARGQVEAVRQLLEAGVDPNRLNRFGRRPIQVMMMGSARVAELLLLHGADPNCADPATLTRPVHDAAREGFLDTLVALHRAGGRLDVRDAWGRLPVDLAEERGHRDVARYLRATAGD

>NP_001270542.1 cyclin-dependent kinase 4 inhibitor B [Macaca fascicularis]

MREENKGMPSGGGSDEGLTSAAARGLVEKVRQLLEAGADPNGVNRFGRRAIQVMMMGSARVAELLLLHGAEPNCADPATLTRPVHDAAREGFLDTLVVLHRAGARLDVRDAWGRLPVILAEERGHRDVAGYLRAATGD

>XP_036897659.1 cyclin-dependent kinase 4 inhibitor B [Sturnira hondurensis]

MWEEDKILPGGGSSDAGLTSAAARGQVETVRQLLEAGADPNGVNCFGRRPIQVMMMGSTHIAELLLLHGADPNCADPATLTRPVHDAAREGFLDTLVALHRAGARLDVRDAWGRLPVDLAEQRGHLDVARYLCAAAGD

>XP_006067118.1 cyclin-dependent kinase 4 inhibitor B [Bubalus bubalis]

MLSGGGGDADLANAAARGQVEAVRQLLEAGVDPNRLNRFGRRPIQVMMMGSARVAELLLLHGADPNCADPATLTRPVHDAAREGFLDTLVALHRAGGRLDVRDAWGRLPVDLAEERGHRDVARYLREAAGD

>XP_032491277.1 cyclin-dependent kinase 4 inhibitor B [Phocoena sinus]

MLSGGGGDAALSNAAARGQVEAVRQLLEAGADPNRLNRFGRCPIQVMMMGSARVAELLLLHGADPNCADPATLTRPVHDAAREGFLDTLVALHRAGARLDVRDAWGRLPVDLAEERGHRDVARYLRTAEGD

>XP_003782895.1 cyclin-dependent kinase 4 inhibitor B [Otolemur garnettii]

MREENKGMFSGGGSDAGLANAAARGQVEKVQQLLEAGADPNGINRFGRRAIQVMMMGSVPVAELLLLHGAEPNCADPTTLTRPVHDAAREGFLDTLVVLHRAGARLDVRDAWGRLPVDLAEERGHFDVVRYLRMAAGD

>XP_041508789.1 cyclin-dependent kinase 4 inhibitor B isoform X2 [Microtus oregoni]

MLGGGSDAGLGTAAARGQVETVRQLLEAGADPNAVNRFGRRPIQVMMMGNAQVARLLLLYGAEPNCEDPTTLSRPVHDAAREGFLDTLVVLHQAGAQLDVPDAWGRLPIDLALEQGHGDVVSYLLAAQNDPQGSGPASIASAQAPPDHP

>XP_020764558.1 cyclin-dependent kinase 4 inhibitor B [Odocoileus virginianus texanus]

MLSGGGGDADLANAAARGQVEAVRQLLEAGVDPNRLNRFGRRPIQVMMMGSARVAELLLLHGADPNCADPATLTRPVHDAAREGFLDTLVALHRAGARLDVRDAWGRQPVDLAEERGHRDVAGYLRAAAGD

>XP_003928163.1 cyclin-dependent kinase 4 inhibitor B [Saimiri boliviensis boliviensis]

MREENKGMPSEGGSDEGLASAAARGQVEKVRQLLEAGADPNGINRFGRRAIQVMMMGSARVAELLLLHGAEPNCADPATLTRPVHDAAREGFLDTLVMLHRAGARLDVRDAWGRLPLDLAEERGHCDVAGYLRAAAGD

>XP_004638619.1 cyclin-dependent kinase 4 inhibitor B [Octodon degus]

MRDEVKGALGDGGGSGGDSDLANAAARGQVEKVRQLLDAGADPNAVNRFGRRPIQVMMMGSAPMAELLLLHGAEPNCADPATLTRPIHDAAREGFMDTLVALHRAGAQLDVRDAWGRLPVDLAEEQGHRDVARYLRAASGD

>XP_028380571.1 cyclin-dependent kinase 4 inhibitor B [Phyllostomus discolor]

MWEEDKILLVGSSDAGLTSAAARGQVETVRQLLEAGADPNGVNCFGRRPIQVMMMGSTRIAELLLLHGADPNCADPITLTRPVHDAAREGFLDTLVALHRAGARLDVRDAWGRLPVDLAEQRGHLDVAQYLCAAAGD

>XP_012305436.1 cyclin-dependent kinase 4 inhibitor B [Aotus nancymaae]

MREENKGMPSGGGSDEGLASAAARGQVEKVRQLLEAGADPNGVNRFGRRAIQVMMMGCARVAELLLLHGAEPNCADPATLTRPVHDAAREGFLDTLVMLHRAGARLDVRDAWGRLPLDLAEERGHCDVAGYLRAAAGD

>XP_003407378.1 cyclin-dependent kinase 4 inhibitor B [Loxodonta africana]

MREEDKGMLGSGSGDGGLASAAARGQVETVRQLLEAGADPNRVNRFGRRPIQVMMMGSARVAELLLLHGAEPNCADPATLTRPVHDAAREGFLDTLMVLQRAGARLDVRDAWGRLPVDLAEERGHRDVALYLRAAAGD

**>NP_001035744.1 cyclin-dependent kinase inhibitor 2A p16INK4a [Mus musculus]**

MESAADRLARAAAQGRVHDVRALLEAGVSPNAPNSFGRTPIQVMMMGNVHVAALLLNYGADSNCEDPTTFSRPVHDAAREGFLDTLVVLHGSGARLDVRDAWGRLPLDLAQERGHQDIVRYLRSAGCSLCSAGWSLCTAGNVAQTDGHSFSSSTPRALELRGQSQEQS

>XP_021016643.1 cyclin-dependent kinase inhibitor 2A [Mus caroli]

MESAADRLARAAAQGRVQDVRALLEAGVSPNAPNSFGRTPIQVMMMGNVHVAALLLNYGADSNCEDPTTLSRPVHDAAREGFLDTLVVLHASGAWLDVRDAWGRLPLDLAQERGHQDVVRYLRSAGWSLCPAGWSLCTAGNVAQTDGHSFSSSTPRALGLRGQSQEQS

>XP_021056477.1 cyclin-dependent kinase inhibitor 2A [Mus pahari]

MESAADRLARAAAQGRVHEVRALLEAGVSPNAPNSFGRTPIQVMMMGNVHVAALLLFYGGDSNCEDPTTLSRPVHDAAREGFLDTLVLLHRSGARLDVRDAWGRLPLDLAQERGHQDVVRYLCSAGWYLRSAGWYLSTAGNVAQADGHNFSSSTPRCLGLRGQSQEQS

>XP_034359152.1 cyclin-dependent kinase inhibitor 2A-like [Arvicanthis niloticus]

MESSADRLARAAAQGREHEVRALLEAGASPNAPNSFGRTPIQVMMMGNVQVAALLLFYGAESNCADPTTLSRPVHDAAREGFLDTLVVLHQAGARLDVRDAWGRLPLDLAEERGHHDVVRYLSSAGNVAQADRHSVCSSTPRYLGLRGQSQEQS

>XP_032757987.1 cyclin-dependent kinase inhibitor 2A [Rattus rattus]

MESSADRLARAAAQGREHEVRALLEAGASPNAPNTFGRTPIQVMMMGNVKVAALLLSYGADSNCEDPTTLSRPVHDAAREGFLDTLAVLHEAGARLDVRDAWGRLPLDLALERGHHDVVRYLRYLLSSAGNVSRVTDRHNFCSSTPRCLGLRGQPREQL

>XP_028629214.1 cyclin-dependent kinase inhibitor 2A-like [Grammomys surdaster]

MEPSADRLARAAAQGREHEVRALLEAGASPNAPNSFGRTPIQVMMMGNVQVAALLLFYGAESNCADPTTLSRPVHDAAREGFLDTLVVLHQAGARLDVRDAWGRLPLDLAVERGHHDVVRYLSSAENVAQVDRHSFCLGTPRYLGLRGQSQE

>NP_113738.1 cyclin-dependent kinase inhibitor 2A [Rattus norvegicus]

MESSADRLARAAALGREHEVRALLEAGASPNAPNTFGRTPIQVMMMGNVKVAALLLSYGADSNCEDPTTLSRPVHDAAREGFLDTLVVLHQAGARLDVRDAWGRLPLDLALERGHHDVVRYLRYLLSSAGNVSRVTDRHNFCSSTPRCLGLRGQPPKQR

>XP_031233691.1 cyclin-dependent kinase inhibitor 2A-like [Mastomys coucha]

MESCADRLARAAAQGRVQEVRALLEAGISPNAPNSFGHTPIQVMMMGNVHVAALLLLYGADSNCADATTLSRPVHDAAREGFLDTLVVLHRAGARLDVRDAWGRLPLDLAQERGHQDVVRYLSAAGNVAPADRHSFC

>XP_038190885.1 cyclin-dependent kinase inhibitor 2A-like isoform X2 [Arvicola amphibius]

MELSADRLARAAAQGREHEVRALLEAGALPNVPNCFGRTPIQVMMMGNAQVARLLLLYGAEPNCEDPATLSRPVHDAAREGFLDTLVVLHQAGARLDVRDAWGRLPIDLALERGHRDVVRYLRAAQNDPQGNGPASLTSAQAPPDQP

>XP_021502696.1 cyclin-dependent kinase 4 inhibitor B-like [Meriones unguiculatus]

MEPSADRLSRAAAQGRVLEVRVLLEAGVSPNARNSFGRTPIQVMMMGSVQVASLLLFHGADPNCEDPATLSRPVHDAAREGFLDTLVVLHQAGARLDVRDAWGRLPLDLAQEQGHHSVLQYLAGSTPQFHSMGSAQAPPGECRSGPVEVKVFTVDSRIRAAGTKREARVFGGLIRAARAQPLSPDLCAAQRGRASWTP

>ERE86202.1 cyclin-dependent kinase inhibitor 2A, isoforms 1/2/3-like protein [Cricetulus griseus]

MEPSADRLARAAAQGREQEVRALLEAGVSPNTPNCFGRTPIQVMMMGNTQVARLLLLYGAEPNCEDPATLSRPVHDAAREGFLETLKILHQAGARLDVHDARGHLPIDLAQERGHLDVVQYLCAAESTPKAAGQQA

>NP_001268314.1 cyclin-dependent kinase inhibitor 2A [Mesocricetus auratus]

MEPSADGLARAAAQGREQEVRALLEAGVSPNAPNCFGRTPIQVMMMGNTQVARLLLLYGAEPNCEDPATLSRPVHDAAREGFLETLAILHQAGARLDVLDARGRLPVDLALERGHCDVVQYLRAAGNTPQGSEPAGVTSAQTPPEVSDFADHPLGPY

>XP_013203697.1 cyclin-dependent kinase inhibitor 2A-like [Microtus ochrogaster]

MELWADRLARAAAQGREHEVLALLEAGALPNVPNCFGRTPIQVMMMGNAKVAKLLLLYGAEPNCEDPATLSRPVHDAAREGFLDTLVVLHQAGARLDVRDAWGHLPIDLALEQGHHDVVSYLLAAQNDSQGSGPASIASAQAPPDHP

>XP_030150115.1 cyclin-dependent kinase inhibitor 2A-like isoform X2 [Lynx canadensis]

MEPLADRLATAAARGRAEEVRALLAAGAQPNAPNRLGRSPIQVMMMGSARVAELLLLHGADPNCADPATLTRPVHDAAREGFLDTLVVLHRAGARLDVRDAWGRLPVALAEERGHRDTVRYLRAATGGTGSGSHTGTDGAEGVADSRT

>XP_003478906.2 cyclin-dependent kinase inhibitor 2A [Cavia porcellus]

MDSAGEKLATAAARGRVEEVRELLEAGAPPDAPNRFGRRPIQVMMMGSTQVARLLLLHGADPNCADPVTLARPVHDAAREGFLDTLVELHQAGARLDVRDAWGRLPVDLAEEQGHRDVAQYLRNASGAV

**>OWK53514.1 Cyclin-dependent kinase inhibitor 2A, isoforms 1/2/3 [Lonchura striata domestica]**

MFKCSTIYIKKLFIVSETLLISAYLSIEAVAKCNDEEFGIGHYSFRNMPDTARHRTSAVMMLGSPRVAELLLRHGADPNRPDPRTGCLPAHDAARAGFLETLAALHRAGARLDLPDGRGRRPLDVAAGGPHGAGGGGGMEGSPRSDGDRLCSAAARGDQEEVRKLLQAGVDPNGTNAFGRTPIQVRNTSVYLELEGIRTDNRKRLSQRMQVMTTRSRPSRITSRRRSQSESSVKL

>XP_031951793.1 cyclin-dependent kinase inhibitor 2A-like isoform X2 [Corvus moneduloides]

MARSEVMMLGSPRVAELLLRRGADPNRPDPRTGCLPAHDAARAGFLETLAALHRAGARLDLPDGRGRLPLDVAAGGPHGAVGRYLRDLPHLPGAGGGRREGCALTRPRSSRRVPVPSSPAARIRAAESPASHRGWRRSRCPTAGKSLPLCRYPQGSSRTPRALRLPLLQEDTYYSIS

>XP_031951792.1 cyclin-dependent kinase 4 inhibitor B-like isoform X1 [Corvus moneduloides]

MEGSPRSDGDHLCSAAARGDHEEVRRLLDAGVDPNGTNSLGRTPLQVMMLGSPRVAELLLRRGADPNRPDPRTGCLPAHDAARAGFLETLAALHRAGARLDLPDGRGRLPLDVAAGGPHGAVGRYLRDLPHLPGAGGGRREGCALTRPRSSRRVPVPSSPAARIRAAESPASHRGWRRSRCPTAGKSLPLCRYPQGSSRTPRALRLPLLQEDTYYSIS

>XP_037981487.1 cyclin-dependent kinase inhibitor 2A-like isoform X1 [Motacilla alba alba]

MEGERADSPVGPHWGGICSVTRPRLLRTHDPADKMHHPLSARPQPAPGLGAGLAHPPPRFRGAGTALAAAPCPAAGPAAEAPRELPAQVMMLGSPRVAELLLRHGADPNRPDPRTGCLPVHDAARAGFLGTLAALHRAGARLDLPDGRGRLPLDVAAGGPHGAVGRYLRDPPPLPGAGGAAEKAAR

>XP_039946951.1 cyclin-dependent kinase 4 inhibitor B-like [Hirundo rustica]

MPDTARYRISPVEAASATALRGNLWAEAGAAGGTGRVFPWARRSPRAGIAAGSAGRPNGGRGAVRGAAGRAADELANAAARGDLQRLGELLGGAADPNAVNSCGRTPIQVMMLGSPRVAELLLRRGADPNRPDPRTGCFPVHDAARAGFLETLAALHRAGARLDLPDGRGRLPLDVAAGGPHGAVGRYLRDPPPLPSLFDFG

>XP_039422898.1 cyclin-dependent kinase 4 inhibitor B-like [Corvus cornix cornix]

MEGSPRSDGDHLCSAAARGDHEEVRRLLDAGVDPNGTNSLGRTPLQVMMLGSPRVAELLLRRGADPNRPDPRTGCLPAHDAARAGFLETLAALHRAGARLDLPDGRGRLPLDVAAGGPRGAVGRYLRDPPHLPGAGGAAEKAAR

**>NP_004927.2 cyclin-dependent kinase 4 inhibitor B isoform 1 [Homo sapiens]**

MREENKGMPSGGGSDEGLASAAARGLVEKVRQLLEAGADPNGVNRFGRRAIQVMMMGSARVAELLLLHGAEPNCADPATLTRPVHDAAREGFLDTLVVLHRAGARLDVRDAWGRLPVDLAEERGHRDVAGYLRTATGD

>XP_039736646.1 cyclin-dependent kinase 4 inhibitor B [Pteropus giganteus]

MREEDKGVLSGGGGDAGLASAAARGQVEMVRQLLEAGADPNKVNCFGRSPIQVMMMGSARVAELLLLHGADPNCADPATLTRPVHDAAREGFLDTVVALHRAGARLDVRDAWGRLPVDVAEERGHRVVARYLRVAAGD

>XP_025708689.1 cyclin-dependent kinase 4 inhibitor B isoform X1 [Callorhinus ursinus]

MREEDKGMLGGGGDDAGLANASAQGQVETVRQLLETGADPNGVNRFGRRPIQVMMMGSTRVAELLLCHGAEPNCADPTTLTRPVHDAAREGFLDTLVVLHRAGARLDVRDAWGRLPVDLAEERGHRAVAGYLRAAAGD

>XP_026362227.1 cyclin-dependent kinase 4 inhibitor B [Ursus arctos horribilis]

MREEDKGMLGGGGDDAGLANAAAQGQVETVRQLLEAGADPNGVNRFGRRPIQVMMMGSTRVAELLLLHGAEPNCADEATLTRPVHDAAREGFLDTLVVLHRAGARLDVRDAWGRLPVDLAEERGHRAVARYLRAAAGD

>XP_032249514.1 cyclin-dependent kinase 4 inhibitor B [Phoca vitulina]

MREEDKGMLGGGGDDAGLANASAQGQVETVRQLLETGADPNGVNRFGRRPIQVMMMGSTRVAELLLCHGAEPNCADPTTLTRPVHDAAREGFLDTLVLLHRAGARLDVRDAWGRLPVDLAEERGHRAVAGYLRAAAGD

>XP_006745528.1 cyclin-dependent kinase 4 inhibitor B [Leptonychotes weddellii]

MREEDKGMLGGGGDDAGLANASAQGQVETVRQLLETGADPNGVNRFGRRPIQVMMMGSTRVAELLLCHGAEPNCADPTTLTRPVHDAARDGFLDTLVVLHRAGARLDVRDAWGRLPVDLAEERGHRAVAGYLRAAAGD

**>XP_002660514.1 cyclin-dependent kinase inhibitor 2A-like [Danio rerio]**

MMNVEDELTTAAATGNISHVQFLLSNGVNANVVNKFRRTPIQVMMMGNAPLALVLLEQGADPNVPDPDTGSTPLHDAARTGFIDTVRLLIRFGADPNTKDHCDLRPVDVAQQTGNVDVVELLNRV

>XP_039550224.1 cyclin-dependent kinase inhibitor 2A [Pimephales promelas]

MMKRMHAEDELTKAAATGNTCRVQFLLYNKVNVNNVNKFGRTPIQVMMMGNTPLAHLLLKYGADPNVPDPGTGSTPLHDAARAGFMDTVRLLILFNADPNATDHCNLRPVDVARQADHVDVVEFLSRF

>XP_026126779.1 cyclin-dependent kinase 4 inhibitor B-like [Carassius auratus]

MMKMMKMHDAEELTKAAATGSTERVRALLCTGASVNGVNRFGRTALQVMMMGNTAVARLLLEHGADPNVSDPGTGSTPLHDAARSGFTDTVRLLLRFEADPSAVDHRGMRAVEVARHTGHLDVAQLLDSI

>XP_024263092.1 cyclin-dependent kinase 4 inhibitor B-like [Oncorhynchus tshawytscha]

MTMPLEDDLASAAATGNTNRVKILLQSGVDVNGVNCFGRTPLQVMMMGSSPVAQLLLMQGADPNIADRHTGTTPLHDAARMGFLDTVEILVQFLADPNSRDNRNCRPIDLAIESGHNNVVAFLKAL

>XP_020326053.2 cyclin-dependent kinase 4 inhibitor B-like [Oncorhynchus kisutch]

MTMPLEDDLASAAATGNTNRVKILLQSGVDVNGVNCFGRTPLQVMMMGSSPVAQLLLMQGADPNIADRHTGTTPLHDAARMGFLDTVEILVQFLADPNCRDNRNCRPIDLAIESGHHNVVAFLKAL

>XP_028833291.1 cyclin-dependent kinase 4 inhibitor B-like [Denticeps clupeoides]

MSLAAGRMRPEDELTSAAATGDARRVQSLLQSGADPNRVNKFGRTPLQVMMAGSAAVALLLLQHGGDPNLPDRSTGDTPLHDAARGGFLDTVRALVEFHADPRITDNRGRRPLDVAERAGHDEVAAFLRPRSG

>XP_036407586.1 cyclin-dependent kinase 4 inhibitor B-like [Megalops cyprinoides]

MFREDELASASATGNTERVGILLQTGTDVNAVNRFGRTPLQVMMMGSTAVARLLLEHGANPNVSDSHTGATPLHDAARGGFLETVAILIQYQADVNARDYNDRRPIDLARTNGHLDVVAFLESL

>XP_023697549.1 cyclin-dependent kinase 4 inhibitor B-like [Paramormyrops kingsleyae]

MDRVDVLTSAAATGNHENVQALLQAGVDANAVNRFGRTALQVMMMGSTAVARVLLQYGADPSLRDRDTGGTPLHDASRGGFLDTVKVLVAHGAEVDARDYGDQRPIDLARENGYRDVVDFLELHLH
