# Supplementary material for: Phylogenetic analysis of the MCL1 BH3 binding groove and rBH3 sequence motifs in the p53 and INK4 protein families
Source: PLoS One. 2023 Jan 25;18(1):e0277726. doi: 10.1371/journal.pone.0277726 (PMC9876281; doi:10.1371/journal.pone.0277726)
Supplement: S7 File — A total of 150 p18 sequences were used to generate the INK4 family phylogenetic tree. (DOCX) [file pone.0277726.s011.docx]

>XP_030428986.1 cyclin-dependent kinase 4 inhibitor C [Gopherus evgoodei]

MAEPLGNELASAAARGDLEKLTNLLQTNVNVNAQNGFGRTALQVMKLGNPEIARQLLMTGANPDLKDSTGFAVIHDAARAGFLDTLQTLLEFNADVNIEDNEGNLPLHLAAQEGHLPVVEFLIKRTESKVEHRNKKGDTAYDLAQLYKRNDVVKLMEGSTLSAEATDMN

>XP_032623162.1 cyclin-dependent kinase 4 inhibitor C [Chelonoidis abingdonii]

MAEPLGNELASAAARGDLEKLTNLLQTNVNVNAQNGFGRTALQVMKLGNPEIARQLLMTGANPDLKDSTGFAVIHDAARAGFLDTLQTLLEFNADVNIEDNEGNLPLHLAAQEGHLPVVEFLIKRTESKVEHRNKKGDTAYDLAKLYKRNDVVKLMEGSTLSAEATDMN

>XP_039340096.1 cyclin-dependent kinase 4 inhibitor C [Mauremys reevesii]

MAEALGNELASAAARGDLEQLTNLLQTNVNVNAQNGFGRTALQVMKLGNPEIARQLLMTGANPDLKDSTGFAVIHDVARAGFLDTLQTLLEFNADVNIEDNEGNLPLHLAAQEGHLPVVEFLIKHTESKVQHRNKKGDTAYDLAKLYKRNDVVKLMEGSTLSAEATDMN

>XP_024063777.1 cyclin-dependent kinase 4 inhibitor C [Terrapene carolina triunguis]

MAEPLGNELASAAARGDLVQLTNLLQTNVNVNAQNGFGRTALQVMKLGNPEIARQLLITGANPDLKDSTGFAVIHDAARAGFLDTLQTLLEFNADVNIEDNEGNLPLHLAAKEGHLPVVEFLIKRTESKVGHRNKKGDTAYDLAKLYKRNDVVKLMEGSTLSAEATDMN

>XP_034633941.1 cyclin-dependent kinase 4 inhibitor C [Trachemys scripta elegans]

MAEPLGNELASAAARGDLVQLTNLLQTNVNVNAQNGFGRTALQVMKLGNPEIARQLLITGANPDLKDSTGFAVIHDAARAGFLDTLQTLLEFNADVNIEDNEGNLPLHLAAKEGHLPVVEFLIKRTESKVGHQNKKGETAYDLAKLYKRNDVVKLMEGSTLSAEATDMN

>XP_005284958.1 cyclin-dependent kinase 4 inhibitor C isoform X2 [Chrysemys picta bellii]

MAEPLGNELASAAARGDLVQLTNLLQTNVNVNAQNGFGRTALQVMKLGNPEIARQLLISGANPDLKDSTGFAVIHDAARAGFLDTLQTLLEFNADVNIEDNEGNLPLHLAAKEGHLPVVEFLIKRTESKVGHQNKKGETAYDLAKLYKRNDVVKLMEGSTLSAEATDMN

>XP_038270762.1 cyclin-dependent kinase 4 inhibitor C [Dermochelys coriacea]

MAEPLGNELASAAARGDLVQLTDLLQTNVNVNAQNGFGRTALQVMKLGNPEIARRLLITGANPDLKDSTGFAVIHDAARAGFLDTLQTLLEFNADVNIEDNDGNLPLHLAAREGHLPVVEFLIKHTASKVEHQNKKGDTAYDLAKLYKRNDVVKLMEGSSLSAEAADMN

>EMP33549.1 Cyclin-dependent kinase 4 inhibitor C [Chelonia mydas]

MAEPLGNELASAAARGDLVQLTNLLQTNVNVNAQNGFGRTALQVMKLGNPEIARRLLITGANPDLKDSTGFAVIHDAARAGFLDTLQTLLEFNADVNIEDNDGNLPLHLAAREGHLPVVEFLIKHTASKVAHQNKKGDTAYDLAKLYKRNDVVKLMESSSLRAEAADMN

>XP_006126488.1 cyclin-dependent kinase 4 inhibitor C [Pelodiscus sinensis]

MAEPLVNDLTSASAKGDLVQLTNLLQKIVNVNAQNGFGRTALQVMKLGNPEIARKLLNAGANPDLKDSTGFAVIHDAARAGFLDTVQTLLEFNADVNIEDNEGNLPLHLAAEEGHLPVVEFLIKHKVSKVDHKNKEGYTAYDLAKLYKKNDIVKLMESSSLSAEAADMN

>XP_021133069.2 cyclin-dependent kinase 4 inhibitor C [Anas platyrhynchos]

MAEPSGNELASAAAKGDLVQLTNLLQKNVNVNAQNGFGRTALQVMKLGNPEIARRLLMSGANPDLKDSTGFAVIHDVARAGFLDTLQTLLEFKADVNIEDAEGNLPLHLAAQEGHVPVVEFLLKSTASKVGHQNKRGDTAYDVAKLYKRSAVLRLLEGGRPPAATD

>XP_035393419.1 cyclin-dependent kinase 4 inhibitor C [Cygnus atratus]

MAEPSGNELASAAAKGDLVQLTNLLQKNVNVNAQNGFGRTALQVMKLGNPEIARRLLMSGANPDLKDSTGFAVIHDVARAGFLDTLQTLLEFKADVNIEDAEGNLPLHLAAQEGHVPVVEFLLKSTASKVGHQNKRGDTAYDVAKLYKRSAVLRLLEGGRPPAATE

>XP_007432337.1 cyclin-dependent kinase 4 inhibitor C isoform X1 [Python bivittatus]

MAEPLANELASAAARGDLAQVTNLLENNINVNAQNGFGRTALQVMKLGNPEIARQLLLRGADPDLKDRTGFAVLHDAARAGFLDTLQTLLEFQADVNVEDNEGNLPLHLAAQEGHLPVVAFLLERTASRVEHRNKKGATAYDLAKLYKRQAVAKLLENSRRREGALSGD

>XP_008830655.1 cyclin-dependent kinase 4 inhibitor C [Nannospalax galili]

MAEPWGNELASAAARGDLEQLTSLLQTNVNVNAQNGFGRTALQVMKLGNPEIARRLLLRGANPNLKDRTGFAVIHDAARAGFLDTVQTLLEFQADVNIEDNEGNLPLHLAAKEGHLPVVEFLVKHTASNVGHRNHKGDTAFDLARLYGRNKVISLMEENGVGGATNLQ

>XP_028589357.1 cyclin-dependent kinase 4 inhibitor C [Podarcis muralis]

MAEPFANELASAAARGDLARLKNLLEKNVNVNAQNGFGRTALQVMKLGNPEIARQLLRRGADPDLRDRTGFAVLHDAARAGFLDTLQTLLEFNADVNVEDAEGNLPLHLAAQEGHLPVVAFLLERTASRVGHRNRKGDTAYDLAQLYRRSAVAKLLEGHAGGSGRTEGADGSGD

>XP_034270953.1 cyclin-dependent kinase 4 inhibitor C [Pantherophis guttatus]

MAEPLANELASAAARGDLAQVTNLLENNINVNAQNGFGRTALQVVKLGNPEIARQLLLRGADPDLKDRTGFAVLHDAARAGFLDTLQTLLEFQADVNVEDNEGNLPLHLAAQEGHLAVVAFLLERTASRVEHRNKKGATAYDLAKLYKRQAIVKLLENSRRREGALSGD

>XP_015725955.1 cyclin-dependent kinase 4 inhibitor C [Coturnix japonica]

MAEPSGNELASAAAKGDLVQLTNLLQKNVNVNAQNGFGRTALQVMKLGNPEIARRLLMSGANPNLKDSTGFAVIHDVARAGFLDTLQTLLEFHADVNIEDAEGNLPLHLAAQEGHVRVVEFLLRRTPSRVMHQNRRGDTALDVARLYRRSAVVRLMEGGPPPAADTD

>XP_031458093.1 cyclin-dependent kinase 4 inhibitor C [Phasianus colchicus]

MAEPSGNELASAAAKGDLVQLTNLLQKNVNVNAQNGFGRTALQVMKLGNPEIARRLLMSGANPNLKDSTGFAVIHDVARAGFLDTLQTLLEFHADVNIEDAEGNLPLHLAAQEGHVRVVEFLLRRTPSRVAHQNRRGDTALDLARLYRRSAVVRLMEGGPPPAADAD

>XP_005151338.1 cyclin-dependent kinase 4 inhibitor C [Melopsittacus undulatus]

MAEPSGNELASAAAKGDLVQLTNLLQKNVNVNAQNGFGRTALQVMKLGNPEIARRLLISGANPNLKDSTGFAVIHDVAREGFLDTLQTLLEFKADVNIEDDEGNLPLHLAAREGHVRVVEVLLARGECKVGHQNKRGATAYDLAKLYKRSAVVKLLEGSSFSPAAMD

>XP_021259845.1 cyclin-dependent kinase 4 inhibitor C [Numida meleagris]

MAEPSGNELASAAAKGDLVQLTNLLQKNVNVNAQNGFGRTALQVMKLGNPEIARRLLMSGANPNLKDSTGFAVIHDVARAGFLDTLQTLLEFHADVNIEDAEGNLPLHLAAQEGHVRVVEFLLRRTPSRVAHQNRRGDTALDVARLYRRSAVVRLMEGGPPPAADAD

>XP_030352067.1 cyclin-dependent kinase 4 inhibitor C [Strigops habroptila]

MAEPSGNELASAAAKGDLVQLTNLLQKNVNVNAQNGFGRTALQVMKLGNPEIARRLLISGANPNLKDSTGFAVIHDVAREGFLDTLQTLLEFKADVNIEDDEGNLPLHLAAREGHVRVVEVLLARAECKVGHQNKRGATAYDLAKLYKRSAVVKLLEGSSFSPAAMN

>KQK85453.1 cyclin-dependent kinase 4 inhibitor C [Amazona aestiva]

MAEPSGNELASAAAKGDLVQLTNLLQKNVNVNAQNGFGRTALQVMKLGNPEIARRLLISGANPNLKDSTGFAVIHDVAREGFLDTLQTLLEFKADVNIEDDEGNLPLHLAAREGHVRVVEVLLARAECKVGHQNKRGATAYDLAKLYKRSAVVELLEGSSFSPAAMD

>XP_015666408.1 cyclin-dependent kinase 4 inhibitor C [Protobothrops mucrosquamatus]

MAEPLANELASAAARGDLAQVTNLLENNINVNAQNGFGRTALQVVKLGNPEIARQLLLRGADPDLKDRTGFAVLHDAARAGFLDTLQTLLEFQADVNVEDNEGNLPLHLAAREGHLAVVAFLLERTASRVEHRNKKGATAYDLAKLYKRQAVAKLLENSRRREGALSGD

>XP_008492169.1 cyclin-dependent kinase 4 inhibitor C isoform X1 [Calypte anna]

MAEPSGNELASAAAKGDLVQLTNLLQKNVNVNAQNGFGRTALQVMKLGNPEIARRLLMKGANPNLKDSTGFAVIHDVAREGFLDTLQTLLEFEADVNIEDNEGNLPLHLAAREGHVRVVEMLLERSECKVDHQNKRGATAYDLAKLYKRAAVVKLLEDSSLFPADMN

>PKU35534.1 cyclin-dependent kinase 4 inhibitor c [Limosa lapponica baueri]

MAEPSGNELASAAAKGDLVQLTNLLQKNVNVNAQNGFGRTALQVMKLGNPEIARRLLINGADPNLKDSTGFAVIHDVAREGFLDTLQTLVEFKADVNIEDNEGNLPLHLAAREGHVRVVEFLLERTECRVGHQNKRGATAYDLAKLYRRSDVVKLLEDSSLLPEDRN

>XP_039175835.1 cyclin-dependent kinase 4 inhibitor C [Crotalus tigris]

MAEPLANELASAAARGDLAQVTNLLENNINVNAQNGFGRTALQVVKLGNPEIARQLLLRGADPDLKDRTGFAVLHDAARAGFLDTLQILLEFQADVNVEDNEGNLPLHLAAQEGHLAVVAFLLERTASRVEHRNKKGATAYDLAKLYKRQAVAKLLENSRRREGALSGD

>XP_026563080.1 cyclin-dependent kinase 4 inhibitor C [Pseudonaja textilis]

MAEPLANELASAAARGDLAQVTNLLENDINVNAQNGFGRTALQVVKLGNPEIARQLLLRGAHPDLKDRTGFAVLHDAARAGFLDTLQTLLEFGADVNVEDNEGNLPLHLAAQEGHLAVVAFLLERTASRVEHRNKKGATAYDLAKLYKRQAVAKLLENSRRKEGALSGE

>XP_020643803.1 cyclin-dependent kinase 4 inhibitor C [Pogona vitticeps]

MAEPFANELASAAARGDLAQVTNLLEKNVNVNAQNGFGRTALQVMKLGNPEIARRLLMRGADPDLKDGTGFAVLHDVARAGFLDTLQTLLEFHADVNVEDNEGNLPLHLAAQEGHLPVVAFLLERTASRVEHRNKRGATACDLAKLYKRHAVARLFEGR

>XP_004588720.1 cyclin-dependent kinase 4 inhibitor C [Ochotona princeps]

MAEPWGNELASAAARGDLEQLTSLLQNNVNVNARNGFGRTALQVMKLGNPEIARRLLLRGANPDLKDQTGFAVIHDAAREGFLDTLQTLLEFQADVNIEDNEGNLPLHLAAKEGHFPVVEFLVKHTASNVGHRNHEGDTACDLARLYGKNEVVSLLEANGVGDPKMCSE

>XP_026710255.1 cyclin-dependent kinase 4 inhibitor C isoform X1 [Athene cunicularia]

MAEPSGNELASAAAKGDLVQLTNLLQKNVNVNAQNGFGRTALQVMKLGNPEIARRLLISGANPNLKDSTGFAVIHDVAREGFLDTLQTLLEFKADVNIEDNEGNLPLHLAAREGHVRVVELLLERAECKVGHQNKRGATAYDLAKLYKRSAVVKLLEDSSFFPAAMN

>KAF7244274.1 Cyclin-dependent kinase 4 inhibitor C [Varanus komodoensis]

MAEPFANELASAAARGDLAQVTNLLEKNVNVNAQNGFGRTALQVMKLGYPEIARRLLLRGADPDLKDGTGFAVLHDAARGGFLDTLQTLLEFQADVNVEDGEGNLPLHLAAQEGHLPVVAFLLDRTASRVEHRNKKGATAYDLAKLYKRDAVAELLEGRCGSRRREGAVSGD

>XP_009642954.1 cyclin-dependent kinase 4 inhibitor C isoform X1 [Egretta garzetta]

MAEPSGNELASAAAKGDLEQLTNLLQKNVNVNAQNGFGRTALQVMKLGKPEIAKRLLIHGANPNLKDSTGFAVIHDVAREGFLDTLQTLLEFEADVNIEDDEGNLPLHLAAREGHVRVVELLLERTECRVGHQNKRGATAYDLAKLYKRAAVVKLLEDSSFPPAATD

>XP_036612594.1 cyclin-dependent kinase 4 inhibitor C [Trichosurus vulpecula]

MAEPLGNELASAAARGDLEQLTNLLQNNVSANAENGFGRTALQVMKLGNPEIARRLLLRGANPDLKDRTGFAVLHDAARAGFLDTLQTLLEFQADVNVEDSEGNLPLHLAAREGHLPVVEFLLRHTACRVDHRNHQGDTACDVARLYRRDAVVRLLEAGRPDDAAPVPGGPAPATSAPAASAEPP

>XP_027693739.1 cyclin-dependent kinase 4 inhibitor C [Vombatus ursinus]

MAEPLGNELASAAARGDLEQLTNLLQNNVSANAENGFGRTALQVMKLGNPEIARRLLLRGANPDLKDRTGFAVLHDAARAGFLDTLQTLLEFQADVNVEDSEGNLPLHLAAREGHLPVVEFLLRHTACRVDHRNHQGDTACDVARLYRRDAVVRLLEAGRPDDAAPLPGGPAPATSAPAAAAEPP

>XP_020843394.1 cyclin-dependent kinase 4 inhibitor C [Phascolarctos cinereus]

MAEPLGNELASAAARGDLEQLTNLLQNNVSANAENGFGRTALQVMKLGNPEIARRLLLRGANPDLKDRTGFAVLHDAARAGFLDTLQTLLEFQADVNVEDSEGNLPLHLAAREGHLPVVEFLLRHTACRVDHRNHQGDTACDVARLYRRDAVVRLLEAGRPDDAAPLPGGPAPATSAPAAPAEPP

>XP_023357329.2 cyclin-dependent kinase 4 inhibitor C [Sarcophilus harrisii]

MAEPLGNELASAAARGDLEQLTNLLQNNVSANAENGFGRTALQVMKLGNPEIARRLLLSGANPDLKDRTGFAVLHDAARAGFLDTLQTLLEFQADVNVEDGEGNLPLHLAAREGHLPVVEFLLRHTACRVDHRNHHGDTACDVARLYRRDAVVRLLEAGRPDDAAPLPGGPAPAASALAAAAEPP

>XP_010161076.1 cyclin-dependent kinase 4 inhibitor C [Antrostomus carolinensis]

MAEPSGNELASAAAKGDLVQLTNLLQKNVNVNAQNGFGRTALQVMKLGNPEIARRLLMNGANPNLKDSTGFAVMHDVAREGFLDTLQTLLEFKADVNVEDAEGNLPLHLAAREGHVRVVELLLEHAECKVGHQNKRGATAYDLAKLYKRSAVVKLLEDSSFFPAATD

>XP_025961608.1 cyclin-dependent kinase 4 inhibitor C [Dromaius novaehollandiae]

MAEPSGNELASAAAKGDLVQLTNLLQKNVNVNAQNGFGRTALQVMKLGNPEIARRLLSRGANPNLKDSTGFAVIHDVARAGFLDTLQTLLEFKADVNIEDAEGNLPLHLAAQEGHAAVVEFLLRRTASRVGHQNKRGATACDLARLYKRTAVLRLLEGARPPPAPQP

>XP_006025374.1 cyclin-dependent kinase 4 inhibitor C [Alligator sinensis]

MKLGNPEIARRLLLKGADPNLRDSTGFAVIHDAARAGFLDTLQTLLEFKADVNIEDNEGNLPLHLAAQEGHARVVEFLVRSTATRVQHRNKRGHTACDLARLYKRAAVVQLLEPGASGAEAADVN

>XP_025942583.1 cyclin-dependent kinase 4 inhibitor C [Apteryx rowi]

MAEPSGNELASAAAKGDLVQLTNLLQKNVNVNAQNGFGRTALQVMKLGNPEIARRLLSRGANPNLKDSTGFAVIHDVARAGFLDTLQTLLEFHADVNIEDAEGNLPLHLAAQEGHAAVVEFLLRRTASRVGHQNKRGATACDLARLYKRTAVLRLLEGPRPPPAPEP

>XP_025909425.1 cyclin-dependent kinase 4 inhibitor C [Nothoprocta perdicaria]

MAEPSGNELASAAAKGDLVQLTNLLQKNVNVNAQNGFGRTALQVMKLGNPEIARRLLSSGADPDLKDRTGFAVIHDVARAGFLDTLQTLLEFEADVNIEDAEGNLPLHLAAQEGHAAVVEFLLRRTASRVGHQNKRGATACDLARLYKRTAVLRLLEAPPAPQP

>XP_032923936.1 cyclin-dependent kinase 4 inhibitor C [Catharus ustulatus]

MAEPSGNELASAAAKGDLVQLTNLLQKNVNVNAQNGFGRTALQVMKLGNPEIARRLLSNGANPNLRDSTGFAVIHDVAREGFLDTLQTLLEFKADVNIEDNDGNLPLHLAAQEGHVRVVELLLARSECKVGHQNKRGATACDLARLYRRAAVVELLEASSSFPPMD

>XP_008923453.1 cyclin-dependent kinase 4 inhibitor C [Manacus vitellinus]

MAEPSGNELASAAAKGDLVQLTNLLQKNVNVNAQNGFGRTALQVMKLGNPEIARRLLSKGANPNLKDSTGFAIIHDVAREGFLDTLQTLLEFKADVNIEDNEGNLPLHLAAQEGHVRVVELLLARSECKVGHQNKRGATAYDLAKLYRRAAVVELLEASSSFPANMD

>XP_027539014.1 cyclin-dependent kinase 4 inhibitor C [Neopelma chrysocephalum]

MAEPSGNELASAAAKGDLVQLTNLLQKNVNVNAQNGFGRTALQVMKLGNPEIARRLLSKGANPNLKDSTGFAIIHDVAREGFLDTLQTLLEFKADVNIEDNEGNLPLHLAAQEGHVRVVELLLARSECKVGHQNKRGATAYDLAKLYRRAAVVELLEATSSFPANMD

>NP_001253.1 cyclin-dependent kinase 4 inhibitor C [Homo sapiens]

MAEPWGNELASAAARGDLEQLTSLLQNNVNVNAQNGFGRTALQVMKLGNPEIARRLLLRGANPDLKDRTGFAVIHDAARAGFLDTLQTLLEFQADVNIEDNEGNLPLHLAAKEGHLRVVEFLVKHTASNVGHRNHKGDTACDLARLYGRNEVVSLMQANGAGGATNLQ

>XP_032016699.1 cyclin-dependent kinase 4 inhibitor C [Hylobates moloch]

MAEPWGNELASAAARGDLEQLTSLLQNNVNVNAQNGFGRTALQVMKLGNPEIARRLLLRGANPNLKDRTGFAVIHDAARAGFLDTLQTLLEFQADVNIEDNEGNLPLHLAAKEGHLRVVEFLVKHTASNVGHRNHKGDTACDLARLYGRNEVVSLMQANGAGGATNLQ

>XP_003259044.1 cyclin-dependent kinase 4 inhibitor C [Nomascus leucogenys]

MAEPWGNELASAAARGDLEQLTSLLQNNVNVNAQNGFGRTALQVMKLGNPEIARRLLLRGANPNLKDRTGFAVIHDAARAGFLDTLQTLLEFQADVNIEDNEGNLPLHLAAKEGHLRVVEFLVKHTASNVGHRNHKGDTACDLARLYGRNEVVSLMQANRAGGATNLQ

>XP_008050038.1 cyclin-dependent kinase 4 inhibitor C [Carlito syrichta]

MAEPWGNELASAAARGDLEQLTSLLQNNVNVNAQNGFGRTALQVMKLGNPEIARRLLLRGANPDLKDRTGFAVIHDAARAGFLDTLQTLLEFQADVNIEDNEGNLPLHLAAKEGHLPVVEFLVKHTASNVGHRNHKGDTACDLARLYGRNEVVSLMQANGAGGAANLQ

>XP_010347535.1 cyclin-dependent kinase 4 inhibitor C [Saimiri boliviensis boliviensis]

MAEPWGNELASAAARGDLEQLTSLLQNNVNVNAQNGFGRTALQVMKLGNPEIARRLLLRGANPDLKDRTGFAVIHDAARAGFLDTLQTLLEFQADVNIEDNEGNLPLHLAAKEGHLPVVEFLVKHTASNVGHRNHKGDTACDLARLYGRNEVVSLMQANGAGGAINLQ

>XP_012290221.1 cyclin-dependent kinase 4 inhibitor C [Aotus nancymaae]

MAEPWGNELASAAARGDLEQLTSLLQNNVNVNAQNGFGRTALQVMKLGNPEIARRLLLKGANPDLKDRTGFAVIHDAARAGFLDTLQTLLEFQADVNIEDNEGNLPLHLAAKEGHLPVVEFLVKHTASNVGHRNHKGDTACDLARLYGRNEVVSLMQANGAGGAINLQ

>XP_008999448.1 cyclin-dependent kinase 4 inhibitor C [Callithrix jacchus]

MAEPWGNELASAAARGDLQQLTSLLQNNVNVNAQNGFGRTALQVMKLGNPEIARRLLLRGANPDLKDRTGFAVIHDAARAGFLDTLQTLLEFQADVNIEDNEGNLPLHLAAKEGHLPVVEFLVKHTASNVGHRNHKGDTACDLARLYGRNEVVSLMQANGAGGAINLQ

>XP_006147317.1 cyclin-dependent kinase 4 inhibitor C [Tupaia chinensis]

MAEPWGNELASAAARGDLEQLTSLLQNNVNVNAQNGFGRTALQVMKLGNPEIARRLLLKGANPDLKDRTGFAVIHDAARAGFLDTLQTLLEFQADVNIEDNEGNLPLHLAAKEGHLPVVEFLVKHTASNVGHRNHKGDTACDLARLYGRNEVVSLMEANGVEGAENLQ

>XP_003799975.1 cyclin-dependent kinase 4 inhibitor C [Otolemur garnettii]

MAEPWGNELASAAARGDLEQLTSLLQNNVNVNAQNGFGRTALQVMKLGNPEIARRLLLRGANPDLKDRTGFAVIHDAARAGFLDTLQTLLEFQADVNIEDNEGNLPLHLAAKEGHLPVVEFLVKHTASHVGHRNHKGDTAFDLAKLYGRNEVVNLMQANGAGGASNLQ

>XP_012631624.1 cyclin-dependent kinase 4 inhibitor C [Microcebus murinus]

MAEPWGNELASAAARGDLEQLTSLLQNNVNVNAQNGFGRTALQVMKLGNPEIARRLLLRGANPDLKDRTGFAVIHDAARAGFLDTLQTLLEFKADVNIEDNEGNLPLHLAAKEGHLPVVEFLVKHTASNVGHRNHKGDTACDLARLYGRSEVINLMQANGAGGPSNLQ

>XP_020040577.1 cyclin-dependent kinase 4 inhibitor C [Castor canadensis]

MAEPWGNELASAAARGDLEQLTSLLQNNVNVNAQNGFGRTALQVMKLGNPEIARRLLLRGANPNLKDRTGFAVIHDAARAGFLDTLQTLLEFQADVNIEDNEGNLPLHLAAKEGHLPVVEFLVKHTASNVGHRNHKGDTAFDLARLYGRNEVISLMEANGVGGSTNLQ

>XP_039101314.1 cyclin-dependent kinase 4 inhibitor C [Hyaena hyaena]

MAEPWGNELASAAARGDLEQLTSLLQNNVNVNAQNGFGRTALQVMKLGNPEIARRLLLRGANPDLKDRTGFAVIHDAARAGFLDTLQTLLEFQADVNIEDNEGNLPLHLAAKEGHLPVVEFLVKHTATKVGHRNHKGDTACDLARLYRRNEVVSLMEANQAEGASNLQ

>XP_005072172.1 cyclin-dependent kinase 4 inhibitor C [Mesocricetus auratus]

MAEPWGNELASAAARGDLEQLTSLLQNNVNVNAQNGFGRTALQVMKLGNPEIARRLLLRGANPNLKDRTGFAVIHDAARAGFLDTVQALLEFQADVNIEDNEGNLPLHLAAKEGHLPVVEFLVKHTASNVGHRNHKGDTAFDLARFYGRNEVISLMEANGVGGATNLQ

>XP_004371811.1 cyclin-dependent kinase 4 inhibitor C [Trichechus manatus latirostris]

MAEPWGNELASAAARGDLEQLTSLLQNNVNVNAQNGFGRTALQVMKLGNPEIARRLLLRGANPDLKDRTGFAVIHDAARAGFLDTLQTLLEFQADVNIEDNEGNLALHLAAKEGHLPVVEFLVKHTASNVGHRNHKGDTACDLARLYRRNEVVSLMEGNGAGGAANQQ

>XP_004869329.1 cyclin-dependent kinase 4 inhibitor C [Heterocephalus glaber]

MAEPWGNELASAAARGDLEQLTSLLQNNVNVNAQNGFGRTALQVMKLGNPEIARRLLLRGANPNLKDRTGFAVIHDAARAGFLDTLQTLLEFQADVNIEDNEGNLPLHLAAKEGHLQVVEFLLKHTASNVGHQNHKGDTAFDLARLYGRNEVLSLMEANGVGGATNLR

>XP_005629070.1 cyclin-dependent kinase 4 inhibitor C [Canis lupus familiaris]

MAEPWGNELASAAARGDLEQLTSLLQNNVNVNAQNGFGRTALQVMKLGNPEIARRLLLRGANPDLKDRTGFAVIHDAARAGFLDTLQTLLEFQADVNIEDNEGNLPLHLAAKEGHLPVVEFLVKHTASKVGHRNHKGDTACDLARLYRRNEVVSLMEGNQAEGASNLQ

>XP_010608589.1 cyclin-dependent kinase 4 inhibitor C [Fukomys damarensis]

MAEPWGNELASAAARGDLEQLTSLLQNNVNVNAQNGFGRTALQVMKLGNPEIARRLLLRGANPNLKDRTGFAVIHDAARAGFLDTLQTLLEFQADVNIEDNEGNLPLHLAAKEGHLQVVEFLLKHTASNVGHQNHKGDTAFDLARLYGRNEVLSLMEANGVGGATNLH

>XP_037672740.1 cyclin-dependent kinase 4 inhibitor C [Choloepus didactylus]

MAEPWGNELASAAARGDLEQLTSLLQNNVNVNAQNGFGRTALQVMKLGNPEIARRLLLRGANPDLKDRTGFAVIHDAARAGFLDTLQTLLEFQADVNIEDNEGNLPLHLAAKEGHLPVVEFLMKHTASNVGHRNHKGDTACDLARLYRRNEVVSLMEGNPEGGAANVQ

>XP_025847644.1 cyclin-dependent kinase 4 inhibitor C [Vulpes vulpes]

MAEPWGNELASAAARGDLEQLTSLLQNNVNVNAQNGFGRTALQVMKLGNPEIARRLLLRGANPDLKDRTGFAVIHDAARAGFLDTLQTLLEFQADVNIEDNEGNLPLHLAAKEGHLPVVEFLVKHTASKVGHRNHKGDTACDLARLYRRNEVVSLMEGNQAEGASSLQ

>XP_004476541.1 cyclin-dependent kinase 4 inhibitor C [Dasypus novemcinctus]

MAEPWGNELASAAARGDLEQLTSLLQNNVNVNAQNGFGRTALQVMKLGNPEIARRLLLRGANPDLKDRTGFAVIHDAARAGFLDTLQTLLEFQADVNIEDNEGNLPLHLAAKEGHLPVVEFLVKHTASNVGHRNHKGDTACDLARLYRRNEVVNLMEGNREGGAENLQ

>XP_006934795.1 cyclin-dependent kinase 4 inhibitor C [Felis catus]

MAEPWGNELASAAARGDLEQLTSLLQNNVNVNAQNGFGRTALQVMKLGNPEIARRLLLRGANPDLKDRTGFAVIHDAARAGFLDTLQTLLEFQADVNIEDNEGNLPLHLAAKEGHLPVVEFLVKHTATKVGHRNHKGDTACDLARLYRRNEVVSLMEGNQAEGASNLQ

>XP_006732130.1 cyclin-dependent kinase 4 inhibitor C [Leptonychotes weddellii]

MAEPWGNELASAAARGDLEQLTSLLQNNVNVNAQNGFGRTALQVMKLGNPEIARRLLLRGANPDLKDRTGFAVIHDAARAGFLDTLQTLLEFQADVNIEDNEGNLPLHLAAKEGHLPVVEFLMKHTASKVGHRNHKGDTACDLARLYRRNEIVSLMEGNQAEGASNLQ

>XP_003411232.1 cyclin-dependent kinase 4 inhibitor C [Loxodonta africana]

MAEPWGNELASAAARGDLEQLTSLLQNNVNVNAQNGFGRTALQVMKLGNPEIARRLLLRGANPNLKDRTGFAVIHDAARAGFLDTLQTLLEFQADVNIEDNEGNLALHLAAKEGHLPVVEFLVKHTASNVGHRNHKGDTACDLARLYRRNEVVSLMEENGAGGAVNQQ

>XP_001492377.1 cyclin-dependent kinase 4 inhibitor C [Equus caballus]

MAEPWGNELASAAARGDLEQLTSLLQNNVNVNAQNGFGRTALQVMKLGNPEIARRLLLRGANPDLKDRTGFAVIHDAARAGFLDTLQTLLEFQADVNIEDNEGNLPLHLAAKEGHLPVVEFLVKHTASKVGHRNHKGDTACDLARLYRRNEIVSLMEGNRAEGAANLQ

>XP_006200529.1 cyclin-dependent kinase 4 inhibitor C [Vicugna pacos]

MAEPWGNELASAAARGDLEQLTSLLQNNVNVNAQNGFGRTALQVMKLGNPEIARRLLLRGANPDLKDRTGFAVIHDAARAGFLDTLQTLLEFQADVNIEDNEGNLPLHLAAKEGHLPVVEFLVKHTASKVGHRNHQGDTACDLARLYRRNEVVSLMEGNRAEGASNLQ

>XP_005353636.1 cyclin-dependent kinase 4 inhibitor C [Microtus ochrogaster]

MAEPWGNELASAAARGDLEQLTSLLQNNVNVNAQNGFGRTALQVMKLGNPEIARRLLLRGANPNLKDRTGFAVIHDAARAGFLDTVQALLEFQADVNIEDNEGNLPLHLAAKEGHLPVVEFLVKHTASNVGHRNHKGDTAFDVARFYGRNEVINLMEANGVGGATNLQ

>XP_003128028.1 cyclin-dependent kinase 4 inhibitor C [Sus scrofa]

MAEPWGNELASAAARGDLEQLTSLLQNNVNVNAQNGFGRTALQVMKLGNPEIARRLLLRGANPDLKDRTGFAVIHDAARAGFLDTLQTLLEFQADVNIEDNEGNLPLHLAAKEGHLPVVEFLVKHTASKVGHRNHQGDTACDLARLYRRNEVVSLMEGNQAEGAPNLQ

>XP_029803467.1 cyclin-dependent kinase 4 inhibitor C [Suricata suricatta]

MAEPWGNELASAAARGDLEQLTSLLQNNVNVNAQNGFGRTALQVMKLGNPEIARRLLLRGANPDLKDRTGFAVIHDAARAGFLDTLQTLLEFQADVNIEDNEGNLPLHLAAKEGHLPVVEFLVKHTATKVGHRNHKGDTACDLARLYRRNEVVSLIEGNPAEGASNLQ

>XP_006180295.1 cyclin-dependent kinase 4 inhibitor C [Camelus ferus]

MAEPWGNELASAAARGDLEQLTSLLQNNVNVNAQNGFGRTALQVMKLGNPEIARRLLLRGANPDLKDRTGFAVIHDAARAGFLDTLQTLLEFQADVNIEDNEGNLPLHLAAKEGHLPVVEFLVKHTASKVGHRNHQGDTACDLARLYRRNEVVSLLEGNRAEGASNLQ

>XP_034357775.1 cyclin-dependent kinase 4 inhibitor C [Arvicanthis niloticus]

MAEPWGNELASAAARGDLEQLTSLLQNNVNVNAQNGFGRTALQVMKLGNPEIARRLLLRGANPNLKDRTGFAVIHDAARAGFLDTVQALLEFQADVNIEDNEGNLPLHLAAKEGHLPVVEFLMKHTACNVGHRNHKGDTAFDLARFYGRNEVMSLMEANGVGGATNLQ

>XP_003463127.1 cyclin-dependent kinase 4 inhibitor C [Cavia porcellus]

MAEPWGNELASAAARGDLEQLTSLLQNNVNVNAQNGFGRTALQVMKLGNPEIARRLLLRGANPNLKDRTGFAVIHDAARAGFLDTLQTLLEFQADVNIEDNDGNLPLHLAAKEGHLQVVEFLLKHTASNVGHRNHKGDTAFDLARLYGRNEVLSLMEANGVGEATDLH

>NP_571977.1 cyclin-dependent kinase 4 inhibitor C [Rattus norvegicus]

MAEPWGNELASAAARGDLEQLTSLLQNNVNVNAQNGFGRTALQVMKLGNPEIARRLLLRGANPNLKDRTGFAVIHDAARAGFLDTVQALLEFQADVNIEDNEGNLPLHLAAKEGHLPVVEFLMKHTACNVGHRNHKGDTAFDLARFYGRNEVISLMEANGVGGATSLQ

>XP_038191347.1 cyclin-dependent kinase 4 inhibitor C [Arvicola amphibius]

MAEPWGNELASAAARGDLEQLTSLLQNNVNVNAQNGFGRTALQVMKLGNPEIARRLLLRGANPNLKDRTGFAVIHDAARAGFLDTVRALLEFQADVNIEDNEGNLPLHLAAKEGHLPVVEFLVKHTASNVGHRNHKGDTAFDVARFYGRNEVINLMEANGVGGATNLQ

>NP_001094524.1 cyclin-dependent kinase 4 inhibitor C [Bos taurus]

MAEPWGNELASAAARGDLEQLTSLLQNNVNVNAQNGFGRTALQVMKLGNPEIARRLLLRGANPDLKDRTGFAVIHDAARAGFLDTLQTLLEFQADVNIEDNEGNLPLHLAAKEGHLPVVEFLVKHTACKVGHRNHQGDTACDLARLYRRNEVVSLMEGNRAEGAANLQ

>XP_027258259.1 cyclin-dependent kinase 4 inhibitor C [Cricetulus griseus]

MAEPWGNELASAAARGDLEQLTSLLQNNVNVNAQNGFGRTALQVMKLGNPEIARRLLLRGANPNLKDRTGFAVIHDAARAGFLDTVQALLEFQADVNIEDNEGNLPLHLAAKEGHLPVVEFLVKHTASNVGHRNHKGDTAFDLARFYGRNEVISLMEANGVGEATNPQ

>XP_008137549.1 cyclin-dependent kinase 4 inhibitor C [Eptesicus fuscus]

MAEPWGNELASAAARGDLEQLTSLLQNNVNVNAQNGFGRTALQVMKLGNPEIARRLLLRGANPNLKDGTGFAVIHDAARAGFLDTLQTLLEFQADVNIEDNEGNLPLHLAAKEGHLPVVEFLVKHTASNVGHRNHNGDTACDLARLYRRNEVVSLMEGNGAEGAENLQ

>XP_031234041.1 cyclin-dependent kinase 4 inhibitor C [Mastomys coucha]

MAEPWGNELASAAARGDLEQLTSLLQNNVNVNAQNGFGRTALQVMKLGNPEIARRLLLRGANPNLKDRTGFAVIHDAARAGFLDTVQALLEFQADVNIEDNEGNLPLHLAAKEGHLPVVEFLMKHTACNVGHRNHKGDTAFDLARFYGRNEVISLMEANGAGGATSL

>XP_028643037.1 cyclin-dependent kinase 4 inhibitor C [Grammomys surdaster]

MAEPWGNELASAAARGDLEQLTSLLQNNVNVNAQNGFGRTALQVMKLGNPEIARRLLLRGANPNLKDRTGFAVIHDAARAGFLDTVQALLEFQADVNIEDNEGNLPLHLAAKEGHLPVVEFLMKHTACNVGHRNHKGDTAFDLARFYGRNEVMSLMEANGVGGATSLQ

>XP_017527945.1 cyclin-dependent kinase 4 inhibitor C [Manis javanica]

MAEPWGNELASAAARGDLEQLTSLLQNNVNVNAQNGFGRTALQVMKLGNPEIARRLLLRGANPDLKDRTGFAVIHDAARAGFLDTLQTLLEFQADVNIEDNEGNLPLHLAAKEGHLPVVEFLVKHTASKTGHRNHKGDTACDLARLYRRNEVVSLMEGIQAEGAANLQ

>XP_036033073.1 cyclin-dependent kinase 4 inhibitor C [Onychomys torridus]

MAEPWGNELASAAARGDLEQLTSLLQNNVNVNAQNGFGRTALQVMKLGNPEIARRLLLRGANPNLKDPTGFAVIHDAARAGFLDTVQALLEFQADVNIEDNEGNLPLHLAAKEGHLPVVEFLVKHTASNVGHRNHKGDTAFDVARFYGRNEVISLMEANGVGGASSLQ

>XP_032971609.1 cyclin-dependent kinase 4 inhibitor C [Rhinolophus ferrumequinum]

MAEPWGNELASAAARGDLEQLTSLLQNNVNVNAQNGFGRTALQVMKLGNPEIARRLLLRGANPNLRDGTGFAVIHDAARAGFLDTLQTLLEFQADVNLEDNEGNLPLHLAAKEGHLSVVEFLVKHTASKVGHRNHKGDTACDLARLYGRNEVVSLMEGNRAEGAENLQ

>XP_007121535.1 cyclin-dependent kinase 4 inhibitor C [Physeter catodon]

MAEPWGNELASAAARGDLEQLTSLLQNNVNVNAQNGFGRTALQVMKLGNPEIARRLLLRGANPDLRDRTGFAVIHDAARAGFLDTLQTLLEFQADVNIEDNEGNLPLHLAAKEGHLPVVEFLVKHTACKVGHRNHQGDTACDLARLYRRNEVVSLMEGNRAEGAANLQ

>XP_040087135.1 cyclin-dependent kinase 4 inhibitor C [Oryx dammah]

MAEPWGNELASAAARGDLEQLTSLLQNNVNVNAQNGFGRTALQVMKLGNPEIARRLLLRGANPDLKDRTGFAVIHDAARAGFLDTLQTLLEFQADVNIEDNEGNLPLHLAAKEGHLAVVEFLVKHTACKVGHRNHQGDTACDLARLYRRNEVVSLMEGDRAEGAANLQ

>XP_004699494.1 cyclin-dependent kinase 4 inhibitor C [Echinops telfairi]

MAEPWGNELASAAARGDLEQLTSLLQNNVNVNAQNGFGRTALQVMKLGNPEIARRLLLRGANPDLRDQTGFAVIHDAARAGFLDTLQTLLEFQADVNIEDNEGNLALHLAAKEGHLQVVEFLVKHTASNVGHRNHEGDTACDLARLYRRNEVVSLMEGCGAGGAPNPQ

>KAF7463111.1 cyclin-dependent kinase 4 inhibitor C [Marmota monax]

MAEPWGNELASAAARGDLEQLTSLLQNNVNVNAQNGFGRTALQVMKLGNPEIARRLLLRGANPNLKDRTGFAVIHDAARAGFLDTLQTLLEFQADVNIEDNEGNLPLHLAAKEGHLPVVEFLVKHTASNVGHRNHSGDTAFDLARLYGKNEVISLLEANGVGVGGAANLQ

>XP_015975944.1 cyclin-dependent kinase 4 inhibitor C [Rousettus aegyptiacus]

MAEPWGNELASAAARGDLEQLTSLLQNNVNVNAQNGFGRTALQVMKLGNPEIARRLLLRGANPNLKDGTGFAVIHDAARAGFLDTLQTLLEFQADVNIEDNEGNLPLHLAAKEGHLPVVEFLVKHTASKVGHRNHKGDTACDLARLYRRNEVVSLMEGNRAEGAENLQ

>XP_037356081.1 cyclin-dependent kinase 4 inhibitor C [Talpa occidentalis]

MAEPWGNELASAAARGDLEQLTSLLQNNVNVNAQNGFGRTALQVMKLGNPEIARKLLLSGANPDLKDRTGFAVIHDAARAGFLDTLQTLLEFRADVNIEDNEGNLPLHLAAKEGHVPVVEFLVKHTASKVGHRNHMGDTACDLARLYRRNEVVSLMEGNQAEGATNLQ

>XP_005326150.1 cyclin-dependent kinase 4 inhibitor C [Ictidomys tridecemlineatus]

MAEPWGNELASAAARGDLEQLTSLLQNNVNVNAQNGFGRTALQVMKLGNPEIARRLLLRGANPNLKDRTGFAVIHDAARAGFLDTLQTLLEFQADVNIEDNEGNLPLHLAAKEGHLPVVEFLVKHTASNVGHRNHSGDTAFDLARLYGKNEVVSLLEANGVGDGGAGNLQ

>NP_001288297.1 cyclin-dependent kinase 4 inhibitor C [Mus musculus]

MAEPWGNELASAAARGDLEQLTSLLQNNVNVNAQNGFGRTALQVMKLGNPEIARRLLLRGANPNLKDGTGFAVIHDAARAGFLDTVQALLEFQADVNIEDNEGNLPLHLAAKEGHLPVVEFLMKHTACNVGHRNHKGDTAFDLARFYGRNEVISLMEANGVGGATSLQ

>XP_021516366.1 cyclin-dependent kinase 4 inhibitor C [Meriones unguiculatus]

MAEPWGNELASAAARGDLEQLTSLLQNNVNVNAQNGFGRTALQVMKLGNPEIARRLLLRGANPNLKDQTGFAVIHDAARAGFLDTVQALLEFQADVNIEDNEGNLPLHLAAKEGHLPVVEFLLKHTACHVGHRNHQGDTACDLARFYGRSEVVSLMEANGVGGAADLQ

>XP_036303838.1 cyclin-dependent kinase 4 inhibitor C [Pipistrellus kuhlii]

MAEPWGNELASAAARGDLEQLTSLLQNNVNVNAQNGFGRTALQVMKLGNPEIARRLLLRGANPNLKDGTGFAVIHDAARAGFLDTLQTLLEFQADVNIEDNEGNLPLHLAAKEGHLPVVEFLVKHTASHVGHRNHNGDTACDLARLYRRNEVVSLMEGTGAEGAENLQ

>XP_004639197.1 cyclin-dependent kinase 4 inhibitor C [Octodon degus]

MAEPWGNELTSAAARGDLEELTSLLQNNVNVNAQNGFGRTALQVMKLGNPEIARRLLLRGANPNLKDRTGFAVIHDAARAGFLDTLQTLLEFQADVNIEDNEGNLPLHLAAKEGHLQVVEYLLKHTASNVGHRNHKGDTAFDLARLYGRNEVLNLMEANGVGRATDLH

>XP_006919240.1 cyclin-dependent kinase 4 inhibitor C [Pteropus alecto]

MAEPWGNELASAAARGDLEQLTSLLQNNVNVNAQNGFGRTALQVMKLGNPEIARRLLLRGANPNLKDGTGFAVIHDAARAGFLDTLQTLLEFQADVNIEDNEGNLPLHLAAKEGHLPVVEFLVKHTASKVGHRNHKGDTACDLARLYRRNEVVSLMEGNRAEGAENRQ

>XP_036899207.1 cyclin-dependent kinase 4 inhibitor C [Sturnira hondurensis]

MAEPWGNELASAAARGDLEQLTSLLQNNVNVNAQNGFGRTALQVMKLGNPEIARRLLLRGANPNLKDGTGFAVIHDAARAGFLDTLQTLLEFQADVNIEDNEGNLPLHLAAKEGHLPVVEFLVKHTASKVGHRNHKGYTACDLARLYRRNEVVSLMEGNPAEGAENLQ

>XP_036206933.1 cyclin-dependent kinase 4 inhibitor C [Myotis myotis]

MAEPWGNELASAAARGDLEQLTSLLQNNVNVNAQNGFGRTALQVMKLGNPEIARRLLLRGADPNLKDGTGFAVIHDAARAGFLDTLQTLLEFQADVNIEDNEGNLPLHLAAKEGHLPVVEFLVKHTTSNVGHRNHNGDTACDLARLYRRNEVVSLMEGTGAEGAENLQ

>XP_024426934.1 cyclin-dependent kinase 4 inhibitor C [Desmodus rotundus]

MAEPWGNELASAAARGDLEQLTSLLQNNVNVNAQNGFGRTALQVMKLGNPEVARRLLLRGANPNLKDGTGFAVIHDAARAGFLDTLQTLLEFQADVNIEDNEGNLPLHLAAKEGHLPVVEFLVKHTASKVGHRNHKGYTACDLARLYRRNEVVSLMEGNPAEGAENLQ

>XP_028369491.1 cyclin-dependent kinase 4 inhibitor C [Phyllostomus discolor]

MAEPWGNELASAAARGDLEQLTSLLQNNVNVNAQNGFGRTALQVMKLGNPEIARRLLLGGANPNLKDGTGFAVIHDAARAGFLDTLQTLLEFQADVNIEDNEGNLPLHLAAKEGHLPVVEFLVKHTASKVGHRNHKGYTACDLARLYRRNEVVSLMEGNPAEGAENLQ

>XP_036111920.1 cyclin-dependent kinase 4 inhibitor C [Molossus molossus]

MAEPWGNELASAAARGDLEQLTSLLQNNVNVNAQNGFGRTALQVMKLGNPEIARRLLLSGANPNLKDGTGFAVIHDAARAGFLDTLQTLLEFQADVNIEDNEGNLPLHLAAKEGHLPVVEFLLKHTASKVGHRNHKGDTAFDLARLYRRNEVVSLMEENQVEGAENLQ

>XP_026521625.1 cyclin-dependent kinase 4 inhibitor C isoform X1 [Notechis scutatus]

MGAPKSHRIPGGVGQLISWPGLVNPPATETGLTPSLLLLAVQEAATRGAKNGLHGVKSEAPALHIGNGTRGFQRSLPRGLGWTELVVKLGNPEIARQLLLRGADPDLKDRTGFAVLHDAARAGFLDTLQTLLEFGADVNVEDNEGNLPLHLAAQEGHLAVVAFLLERTASRVEHRNKKGATAYDLAKLYKRQAVAKLLENSRRREGALSGD

>XP_033008063.1 cyclin-dependent kinase 4 inhibitor C [Lacerta agilis]

MAEPFANELASAAARGDLAQLTNLLENNVNVNAQNGFGRTALQVMKLGNPEIARRLLLRGADPNLRDRTGFAVLHDAARAGFLDTLQTLLEFKADVNVEDSEGNLPLHLAAQEGHLPVVAFLLERTASRVGHRNKKGDTAYDLAQLYRRSAVAKLLEGRAGGSRRTEGADGSGD

>XP_034980132.1 cyclin-dependent kinase 4 inhibitor C [Zootoca vivipara]

MAEPFANELASAAARGDLAQLTNLLENNVNVNAQNGFGRTALQVMKLGNPEIARRLLLRGADPDLKDRTGFAVLHDAARAGFLDTLQTLLEFKADVNAEDGEGNLPLHLAAQEGHLPVVAFLLERTASRVGHRNKKGETAYDLAQLYRRSAVARLLEGRAGN

>XP_004936837.1 cyclin-dependent kinase 4 inhibitor C [Gallus gallus]

MAEPSGNELASAAAKGDLVQLTNLLQKNVNVNAQNGFGRTALQVMKLGNPEIARRLLMSGANPNLKDSTGFAVIHDVARAGFLDTLQTLLEFHADVNIEDAEGNLPLHLAAQEGHVRVVEFLLRRTPSRVAHQNRRGDTALDVARLYRRSAVVQLMEGGPPPAADAD

>XP_013150074.1 cyclin-dependent kinase 4 inhibitor C [Falco peregrinus]

MAEPSGNELASAAAKGDLVQLTNLLQKNVNVNAQNGFGRTALQVMKLGNPEIARRLLVSGANPNLKDSTGFAVIHDVAREGFLDTLQTLLEFKADVNIEDDEGNLPLHLAAREGHVRVVELLLARAECKVGHQNKRGATAYDLAKLYKRAAVVKLLEDSSFFPAAMN

>XP_006087363.1 cyclin-dependent kinase 4 inhibitor C [Myotis lucifugus]

MAEPWGNELASAAARGDLEQLTSLLQNNVNVNAQNGFGRTALQVMKLGNPEIARRLLLRGANPNLKDGTGFAVIHDAARAGFLDTLQTLLEFQADVNIEDNEGNLPLHLAAKEGHLPVVEFLVKHTTSNVGHRNHNGDTACDLARLYRRNAVVSLMEGTRAEGAENLQ

>NP_001116932.1 cyclin-dependent kinase 4 inhibitor C [Xenopus tropicalis]

MEDPMADLITTAAARGELERLEDLLKGARNVDAPNRFGRTALQVMRLGNPAVARLLLSQGADPNLRDRTGYSVLHDAARAGFQDTLKTLFDFQADANIQDNEGNLPLHLAAKEGHLQVVKFLVLHTDSQVGHRNRYGDTPCDLAKVYKREDVIQWLHCYANGQEPGGK

>XP_040263626.1 cyclin-dependent kinase 4 inhibitor C [Bufo bufo]

MAGHLVMEPVEGGWMEKVRKGDMSGWRRNLAAYGWRSRQKERERILQRKARARREGIGQMRGSACEEKGHLVMEPVEGDWVQKVRKGERSGCRRNPAGDPDRRKGRGYCRGGLEQGERGGCRNICSSRVPGGGPCFTSIYYDPPISSEPRLSACSCHALITLCPGPQPSMAEPLADLMSTAAARGDLEQLEDLLQRTTNVDAPNRFGRTALQVMRLGCPAIASLLLRRGADPNLQDRSGFSVLHDTARAGFLDTLQILLDFQADVNLKDHDGNIALHLAAMEGHLPMVQYLVLQTDTRVTQRNMNGDTPCDLARMYKREHVVLWLQSNARGQTGDGQ

>XP_040216441.1 cyclin-dependent kinase 4 inhibitor C [Rana temporaria]

MAQPLADRMTSAAARGDLHTLEDLLRIAPNVDVPNRFGRTALQVMRLGCPAVASLLLGKGADPNLQDSCGFSVVHDTARAGFCDTMRILLDFHVDVNLQDNDGNTALHLAAKEGQLHMVQLLVLHTDSRMGHKNRNGETACDLARVYSRQPVVQWLQGTARGQP

>XP_033771151.1 cyclin-dependent kinase 4 inhibitor C isoform X2 [Geotrypetes seraphini]

MMADPFGNELTSAAARGDQEQVEILLQNNANVNAKNEFGRTALQVMKLGNTGIARRLLYKGADPNLKDRTGFAVIHDAARAGFLDTVKTLLQFQADVNIEDNEGNLPLHLAAQEGHLGVVEFLVRHTESNVGHINHKGATAHDLAKMYKKEEVVKWMNDNAHVQATNLQ

>XP_028902443.1 cyclin-dependent kinase 4 inhibitor C [Ornithorhynchus anatinus]

MAEPQGNELATAAARGDLERLAHLLQTNVNVNAQNGFGRTALQVMRLGNPAIARLLLVGGADPDLKDGTGFAALHDAARAGYLDTLQTLLEFQADVNVEDRDGNLPLHLAAREGHLPVVRFLLNHTASKVGHRNRHGDTACDLARLYRRSDVVRFMEATAKPGQ

>XP_030064050.1 cyclin-dependent kinase 4 inhibitor C isoform X1 [Microcaecilia unicolor]

MSVSIPNEILKETNLFRSRDKIMADPFGNELTSAAARGDQEQVEILLQNNANVNAKNGFGRTALQVVKLGNPGIARRLLHEGADPNVKDRTGFAVIHDAARAGFLDTVQTLLQFQADVNIEDNEGNLPLHLAAQEGHLSVVEFLVRHTESNVGHLNHKGATAHDLAKMYKREEVVKWMDGNVRVQTANLLQ

>XP_038616614.1 cyclin-dependent kinase 4 inhibitor C [Tachyglossus aculeatus]

MAEPQGNELATAAARGDLERLTHLLQTNVNVNAQNGFGRTALQVMRLGNPAIARLLLLGGADPDLKDGTGFAALHDAARAGYLDTLQTLLEFRADVNVEDRDGNLPLHLAAREGHLPVVRFLLDHTASKVGHRNRHGDTACDLARLYRRSDVVTFMEATAKPGQ

>XP_029474340.1 cyclin-dependent kinase 4 inhibitor C [Rhinatrema bivittatum]

MADPLGNLLTSAAARGDQEQVEILLQNNANVNAENGFGRTALQVMKLGNPEIARRLLHRGADPNLRDRTGFAVMHDAARAGFLDTVQTLLQFQADVNLEDREGNLPLHLAAQEGHLGVVEALARHSRSSVGRRNHRGATARDLARLYKREEVAKWLDEHAGAQAAGL

>NP_001186923.1 cyclin-dependent kinase 4 inhibitor C [Danio rerio]

MAEDTALDRLSTAAAIGDLMEVEQTLQSNVNVNEKNKYGRTALQVMKLGCPSIAETLLQAGADPNVRDPILGLTVIHDAARDGYLDTLHVLAQNGADVNLLDNDGNLPLHLAAREGHLDVVQFLVTHCVTQPFLANAKGYTPRDLAFMHQKHRTVEWLESIASLQSSQRL

>XP_039531555.1 cyclin-dependent kinase 4 inhibitor C [Pimephales promelas]

MAEDTTIDGLSTAAAQGNLMEIEQILQSNVNVNEKNKFGRTPLQVMKLGCPRIAETLLLAGADPNARDPILGLTVSHDAARDGYLDTLRVLVENGADVNLLDNKGNLPLHLAAQGGCLDVVQYLVSYCNTQPFLRNAKGQTPLDLASLHKKYQTVEWLENIAPSQSS

>XP_036378535.1 cyclin-dependent kinase 4 inhibitor C [Megalops cyprinoides]

MADSSEANRLASASARGDLTEVEVILQNGADVNEKNAFGRTALQVMKLGNPAIAEALLKANADPNVRDPLGGLTVLHDTARDGYADTLRVLVEYGADVNMLDNEGNLPLHLAAREGNLDVVKFLIQRTTDPSRRNVKGKTAYDLATMHNRHSTAQWIEEYIKSRD

>XP_012677154.1 cyclin-dependent kinase 4 inhibitor C [Clupea harengus]

MAGRTQADKLTTASARGDLREVEMILQNGADTNERNMLNRTAIQVMKLGNPDIAEALLRANADPNVRDPVLGLTVAHDASRDGFLDTLNVLVHHGADVNLIDFGGNLPLHLAAREGHLDVVQFLTQHTSDPSQLNGTGQTPCDLAKMHNRSSTVQWLETYVLSQQN

>XP_027022257.1 cyclin-dependent kinase 4 inhibitor C [Tachysurus fulvidraco]

MAEAPDVNRLSTAAARGDLRETEQILKSNMNVNAKNIFGRTPLQVVKLGCPSVAEALLRAGADPNVRDPCGGLTVTHDAARDGHTDTLQVLLRNGADVNLQDDAGNLPLHLSAREGHQSAVELLIPLTEHPLLHNHAGLTPMDLATHHHRDNTARWLENYLFVPSQSD

>XP_026857188.1 cyclin-dependent kinase 4 inhibitor C isoform X1 [Electrophorus electricus]

MAEAPDANRLCTAAARGDLREIEMILQSNIDVNEKNIFGRTPLQVVKLGCPCAAEALLLAQADPNLRDPIGGLTITHDAARDGYVDTLQVLVNYGANVNLLDGGGNLPLHLAAREGHLDVVQFLIQHTTQPFQKNGAGLTPLELASVNNRDDTARWLETYRSTNSH

>XP_026770716.1 cyclin-dependent kinase 4 inhibitor C [Pangasianodon hypophthalmus]

MAEAPDVNRLSTAAARGDLRETEEILESNINVNAKNKFGRTALQVVKLGCPSVAEALLRAGADPNVPDPSGGLTVMHDAAREGHADTLQVLLRYGADASLQDDAGNLPLHLAAREGHQGAVELLAPHTAHPLLPNHAGLTPLQLASQHHRDDTARWLENYRHVPSQPE

>XP_030640214.1 cyclin-dependent kinase 4 inhibitor C [Chanos chanos]

MAGSTDANMLTSAAARGDIEEIETMLQRGVDVNERNEFGRTALQVMKLGNPDIARVLLTANADPNVRDPIRGLTITHDAARDGYIDTLQVLVEHGADVNILDSEDNLPLHLAAREGHLRVVRFLFQWTRHPSHINVEGHAPYDLARIHNRQSTALWLESMLQQNN

>XP_036433950.1 cyclin-dependent kinase 4 inhibitor C [Colossoma macropomum]

MAEAPDANRLCTAAARGDLRETQLILQGNVDVNERNKFGRAPLQVVKLGCPRVAEALLQANADPNARDPVKRLTITHDAARDGYVDMLEVLVTYGADVNLQDSDGNLPLHLASREGHLDAVRYLAPLTAWPFLRNREDLTPLDLALAHHRADIAQWLQTYRPPAAIEPSLD

>XP_017548914.1 cyclin-dependent kinase 4 inhibitor C [Pygocentrus nattereri]

MAEASDANRLCTAAARGDLRETQLILQGNVDVNERNKFGRAPLQVVKLGCPRVAEVLLQAKADPNARDPVKRLTIAHDAARDGYVDMLEVLVTYGADVNLQDSDGNLPLHLASREGHLNAVRYLAPLTAWPFLRNREDFTPLDTALAHHRADVAQWLQTYRPPAATAPSLD

>XP_035268343.1 cyclin-dependent kinase 4 inhibitor C [Anguilla anguilla]

MADPSGANRLTSASARGDLTELQVFLQNGADVNERNEFGRTALQVMKLGNPAIALALLQAKADPNVRDPVGGLTVLHDAARDGYADTLQVLVNHGADVNIQDNEGNLPLHLAAREGNLDVVELLIQPTAEPMRRNRGGHTAYDLAIMRSRVSTAQRIQAYMHSIE

>XP_038858456.1 cyclin-dependent kinase 4 inhibitor C [Salvelinus namaycush]

MTGEANRLSSASARGDLAKVEMLLQNGADVNANNVFGRTPLQVMKLGNPAIAEALLRANANPNVRDHVRGLTITHDAARDGYVDTLRVLIDHGADVNLLDNDGNLPLHLAAREGYLDVVQLLVGCTKDAARHNSGGHTPYDLATMNNRVSIAQYIQAHMNL

>XP_035637909.1 cyclin-dependent kinase 4 inhibitor C [Oncorhynchus keta]

MTGEANRLSSASARGDLAEVEMLLQNGADVNANNVFGRTPLQVMKLGNPAIAEALLRANANPNVRDHVRGLTITHDAARDGYVDTLRVLIDHGADVNLLDNDGNLPLHLAAREGYLDVVQLLVGCTKDAARHNSGGHTPYDLATMNNRISIALYIQAHMNL

>XP_021459712.2 cyclin-dependent kinase 4 inhibitor C [Oncorhynchus mykiss]

MTGEANRLSSASARGDLAEVEMLLQNGADVNANNVFGRTPLQVMKLGNPAIAEALLRANANPNVRDHVRGLTITHDAARDGYVDTLRVLIDHGADVNLLDNDGNLPLHLAAREGYLDVVQLLVCCTKDAARHNSGGHTPYDLATMNNRVSIALYIQAHMNL

>XP_020354712.1 cyclin-dependent kinase 4 inhibitor C [Oncorhynchus kisutch]

MTGEANRLSSASARGDLAEVEMLLQNGADVNANNVFGRTPLQVMKLGNPAIAEALLRANANPNVRDHVRGLTITHDAARDGYVDTLRVLIDHGADVNLLDNDGNLPLHLAAREGYLDVVQLLVGCTKDAARHNSGGHTPYDLATMNNRVSIALYIQAHMNL

>XP_029563113.1 cyclin-dependent kinase 4 inhibitor C [Salmo trutta]

MTGEANRLSSASARGDLAKVEMLLQNGADVNANNVFGRTPLQVMKLGNPAIAEALLRANANPNVRDHVRGLTITHDAARDGYVDTLRVLIDHGADVNLLDKDGNLPLHLAAREGYLDVVQLLVGCTKDAARHNSGGHTPYDLATMNNRVSIAQYIQAHMNL

>XP_028668291.1 cyclin-dependent kinase 4 inhibitor C [Erpetoichthys calabaricus]

MTDSTDADELANASARGDLRVVQMLLQSGVNVNQRNTYGRTPLQVMKLGYPAIAVELLRANANPNEQDISLGVTIAHDAAREGFLETLMVLTENGADVNLQDHRGNLPLHLAAREGHLDIVRHLIDLTIDPLVRNKEGHTASDLAEMHDRHLVAQWLGTRLQR

>XP_039623203.1 cyclin-dependent kinase 4 inhibitor C [Polypterus senegalus]

MTDSTDADELANASARGELRVVQILLRSGVNVNQKNTYGRTPLQVMKLGYPAIAVELLRANANPNEQDTSLGVTIAHDAAREGFLETLMVLTENGADVNLQDHQGNLPLHLAAREGHLDIVRHLIDLTSDPLVKNKEGHTASDLAEMHDRHLVAECLGNRLQR

>XP_007231730.1 cyclin-dependent kinase 4 inhibitor C [Astyanax mexicanus]

MAEATVDANTLCSAAATGQFKMVQQILQCNVDPNKKNEFNRTALQVVKMGCPAVVELLLKKGADPNLRDPVKDLTISHDVAREGHADTLAVLLTYVADVNLKDKDGNLPLHLAAREGHLDAVRLLAPCTAHLFSRNHEGLTPIELARAHRREDTARWLESYQPPQTPQQPQSLD

>XP_018582255.1 cyclin-dependent kinase 4 inhibitor C [Scleropages formosus]

MADLTETNKLTSASARGDLREVELMITNGADVNQRNEYGYTALQAMKLGCPDLATALLKANADPNVCDALGLTVTHDAARDGYLDTLRVLLEHGADVNVTDSHGNLPLHLAAREGNLDVVELLLQRTAEPMRPNCEGSTAYDLATSYNRPSVARIIRDHPGINLAQ

>XP_028857173.1 cyclin-dependent kinase 4 inhibitor C [Denticeps clupeoides]

MDDQTAAADDLSSASARGDAAEVERMLRGGADVNGRNRFGRTPIQVMKLGCPAVAELLLLRGADADVRDPVAGLTVAHDAARDGHADTLRVLVGHKADVNVADVHGNLPLHLAAQEGHARAVRVLLGPTAAPGARNKRGHTPRDLAEMNGRDEVLRVMDGVREDVAGN

>XP_023677136.1 cyclin-dependent kinase 4 inhibitor C [Paramormyrops kingsleyae]

MADLTEANKLTSASARGDLSEVETLLQSGADVNKANRFGRTALQVMKLGVPGLAEMLLEAKADPNKRDPVKDLTIAHDAARGGYLETLQVLVKYGADVNAVDSDGNLPLHLAAQAGYRDVVEFLEQCTADQGNTADDLTPTHSKEYIQGRMQSQN

>XP_038571859.1 cyclin-dependent kinase 4 inhibitor C [Micropterus salmoides]

MADRSVADKLCSASARGNLPEVLFLLQNGADVNGFNTFNRTALQVVKLGNTAVVEALLLVGADINVRDPACGLTVTHDAAREGFIDSVRVLVDRGADTNIVDEQGNLPLHLAAREGHLEVVQLLIGRTANPQTRNGLGYTARQLAFLNGRMDTVKYIDEYLSSQ

>XP_039567632.1 cyclin-dependent kinase 4 inhibitor C [Passer montanus]

MAEPSGNELASAAAKGDLVQLTNLLQKNVNVNAQNGFGRTALQVMKLGNPEIARRLLSNGANPNLKDSTGFAVIHDVAREGFLDTLQTLLEFKADVNIEDNDGNLPLHLAAQEGHVRVVQLLLARSECKVGHQNKRGATAYDLAKLYRRAAVVELLEASSSFPPSMD

>XP_039929527.1 cyclin-dependent kinase 4 inhibitor C [Hirundo rustica]

MAEPSGNELASAAAKGDLVQLTNLLQKNVNVNAQNGFGRTALQVMKLGNPEIARRLLSNGANPNLKDSTGFAVIHDVAREGFLDTLQTLLEFKADVNIEDNDGNLPLHLAAQEGHVRVVELLLARSECKVGHQNKRGATAYDLAKLYRRAAVVELLEASSSLPPSMD

>XP_009963282.1 cyclin-dependent kinase 4 inhibitor C [Tyto alba alba]

MAEPSGNELASAAAKGDLVQLTNLLQKNVNVNAQNGFGRTALQVMKLGNPEIARRLLVSGANPNLKDSTGFAVIHDVAREGFLDTLQTLLEFKADVNIEDNDGNLPLHLAAREGHVRVVELLLGHAECKVGHQNKRGATAYDLAKLYKRSAVVKLLEDSSFFPAAMN

>XP_010896098.1 cyclin-dependent kinase 4 inhibitor C isoform X1 [Esox lucius]

MAGEANILSSASARGDLADVEMLLQNGTDVNENNVFGRTPLQVMKLGNPAIAAALLKANANPNVRDQVKGLTVCHDAARDGYADTLRVLVDHGADVNLLDHDGNLPLHLAAREGHLEVVQILFCITMDVERRNSEGHTPYDLANLNHRESIAQYIQTHINL

>AFK11493.1 cyclin-dependent kinase 4 inhibitor C [Callorhinchus milii]

MSPLTDPLPVPGIPELTAKDGDKLTSAAAKGDSKEVNVLLEKGIKVCSVNKFGSTAIQVMQMGNTIIARSLLKAGAKPNQQDSNGFTPAHDAAREGFVDTLRVLVDSGADVNIENSEGNLPIHLAAQEGHTDVLIFLEEKSNLAHKNLKGQTPIDLAQMYKRTETLQWMKQSYRKNIGKHV

>XP_041064218.1 cyclin-dependent kinase 4 inhibitor C [Carcharodon carcharias]

METADGSDGDKLTSAAAKGDSKEVNTLLENGVKVGAINKFGRTALQVMQMGNTIIAKSLLKAGAKPNQQDRGGFTPAHDAAREGFLDTLKTLVDFGANVNIENSEGNLPIHLAAQEGHTDVIIFLAKKSNLTHKNKMGQTPYELARMYKRTETVQWMEQNL

>XP_020370188.1 cyclin-dependent kinase 4 inhibitor C [Rhincodon typus]

METADGSDGDKLTSAAAKGNSKEVNILLENGVKVGAINKFGRTALQVMQMGHTIIAKSLLKAGAKPNQQDRGGFTPAHDAAREGFLDTLITLVDFGANVNIENSEGNLPIHLAAQEGHTDVIIFLAKKSNLTHKNKKGQTPYELAQMYKRTETVQWMEQNL

>XP_038650211.1 cyclin-dependent kinase 4 inhibitor C [Scyliorhinus canicula]

METADASDGDKLTSAAAKGDSKEVNALLENGVKVGALNKFGRTALQVMQMGNTIIAKSLLKAGAKPNQQDPGGYAPAHDVAREGFLDTLKILVDFGANVNIKNSEGNLPIHLAAQEGHTDVIIFLAKKSNLAHKNEKGQTPFELAQMYKRTETVQWMEQNL

>XP_032884463.1 cyclin-dependent kinase 4 inhibitor C [Amblyraja radiata]

METTDGSVGDKLTSAAAKGDSKEVNSLLENGVQADAINKFGRTALQVMQMGHSIIAKSLLKAGAKPNQQDHGGFTPAHDAAREGFLDTLKTLVDFGANVNIENSEGNLPIHLAAQEGHTDVVTFLAKESNLTFKNENGQTPYELAQIYKRTETMQWMEQNL

>XP_041125632.1 cyclin-dependent kinase 4 inhibitor C-like [Polyodon spathula]

MADSSDADRLASASARGELKEVETLLQNGANVNEKNSFGRTALQVMKLGNPSIAEALLRAKANPNERDPLWGLTVAHDAARDGFEDTLRVLVDYGADVNIQDSEGNLPLHLAAKEGNLDVVKFLIERTVEPKRANRKGMTAYDLAAMHNRQSTAQWIEGHLYGHVQN

>XP_033876077.1 cyclin-dependent kinase 4 inhibitor C-like [Acipenser ruthenus]

MADSSLASASARGELEKVEMLLQNGANVNEKNAFGRTPLQVMKLGNPSIAEALLRAKANPNERDPLLGLTVAHDAARDGFEDTLRVLVDYGADVNIEDSEGNLPLHLAAKEGNLDVVKFLIERTIEPRRANRKGMTARELAAMHNRQSTAQWIEGHSYGHVQN

>XP_018116370.1 cyclin-dependent kinase 4 inhibitor C [Xenopus laevis]

MEDPMADLISTAAARGELERLEDLLKGARNVDAPNRFGRTALQVMRLGNPAIARLLLSQGADPNLRDRTGYSVLHDAARAGFQDTLETLFDFQADANIQDNEGNLPLHLAAKEGHLQVVKFLVLHTDSQLGHQNRYGDTPCDLAKVYKRQDVIQWLHCYANGQEKGGK
