# Supplementary material for: Phylogenetic analysis of the MCL1 BH3 binding groove and rBH3 sequence motifs in the p53 and INK4 protein families
Source: PLoS One. 2023 Jan 25;18(1):e0277726. doi: 10.1371/journal.pone.0277726 (PMC9876281; doi:10.1371/journal.pone.0277726)
Supplement: S8 File — A total of 188 p19 sequences were used to generate the INK4 family phylogenetic tree. (DOCX) [file pone.0277726.s012.docx]

>XP_026535449.1 cyclin-dependent kinase 4 inhibitor D **[Notechis scutatus]**

MQPGNAEIPAGDRLTGAAARGDITEVRHILHLELVHPDSHNRFGKTALQVMMFGNIFVAEELLKQGANPNIQDGSGTTPAHDAARTGFLDTLKILVEHGADVNVPDASGSLPIHVAIQEGHTDVVCFLAPQSQLQQKDSKGQTPLELAEHLGLSHIQCILEQHLSVPA

>XP_026572602.1 cyclin-dependent kinase 4 inhibitor D [Pseudonaja textilis]

MQPGNAEIRAGDRLTGAAARGDIAEVGHILHLELVHPDSHNRFGKTALQVMMFGNIFVAEELLKQGANPNIQDGSGTTPAHDAARTGFLDTLKILVEHGADVNVPDASGSLPIHVAIQEGHTDVVCFLAPQSQLQQKDSKGQTPLELAEHLGLSHIQCILEQHLSVPA

>XP_034293742.1 cyclin-dependent kinase 4 inhibitor D isoform X6 [Pantherophis guttatus]

MKLKKPQTASVMRGRGQGACAGMQPGNAEIRAGDRLTGAAARGDITEVRHLLHLELVHPDSHNRFGKTALQVMMFGNIFVAEELLKQGANPNIQDGSGTTPAHDAARTGFLDTLKILVEHGADVNVPDASGSLPIHVAIREGYTDVVCFLAPQSQLQQKDSKGRTPLELAEDLGLSHIQCILEQHLSVPA

>XP_032068316.1 cyclin-dependent kinase 4 inhibitor D [Thamnophis elegans]

MQPGNAEIRAGDRLTGAAARGDIAEVRHLLHLELVHPDSHNRFGKTALQVMMFGNIFVAEELLKQGANPNVRDGSGTTPAHDAARTGFLDTLKVLVEHGADVNVPDASGSLPIHVAIREGRTDVVCFLAPQSQLQQKDSKGQTPLELAEHLGLSHIRCILEQHLSVPA

>XP_007430265.1 cyclin-dependent kinase 4 inhibitor D [Python bivittatus]

MQPGNAEIRAGDRLTGAAARGDVAEVRHLLHLELVHPDSHNRFGKTALQVMMFGNIFVAEELLKQGANPNIQDGSGTTPAHDAARTGFLDTLKILVEHGADVNVPDASGSLPIHVAIREGHTDVVCFLAPQSQLQHKDSKGKTPLELAQHLGLSHIQGILEQHLSVPA

>XP_039220249.1 cyclin-dependent kinase 4 inhibitor D [Crotalus tigris]

MQLGNAEIRAGDRLTGAAARGDIAEVRHLLHLELVHPDSHNRFGKTALQVMMFGNIFVAEELLKQGANPNIQDGSGTTPAHDAARTGFLDTLKILVEHGADVNVPDASGSLPIHVAIREGHTDVVCFLAPQSQLQHKDYKGRTPLELAEHLGLSHIQCILEQHLFVPA

>XP_015676126.1 cyclin-dependent kinase 4 inhibitor D [Protobothrops mucrosquamatus]

MQLGNAEIQAGDRLTGAAARGDIAEVRHLLHLELVHPDSHNRFGKTALQVMMFGNIFVAEELLKQGANPNIQDGSGTTPAHDAARTGFLDTLKILVEHGADVNVPDASGSLPIHVAIREGHTDVVCFLAPQSQLQHKDYKGRTPLELAEHLGLSHIQCILEQHLFVPA

>XP_020647291.1 cyclin-dependent kinase 4 inhibitor D isoform X1 [Pogona vitticeps]

MGGRLFSGRQTLHPPFGSSRLRALQALSLFPLPPVLPGRDLLLALRGRTGQAGGGESRANLFPPEPAFESRRPAQQFCCRWPIGMGMSPASARSWPPIGCAREKGGAARERENESESRARVTMYPSSGSAGRNHKQAPRAQVPVCTGMQPGQGETRAGDRLTGAAARGDVAEVRRLLHQELVQPDSRNRFGKTALQVMMFGNIFVAQELLKEGAHPNVQDESGTTPAHDAARTGFLDTLKILVEYGADVNVPDASGSLPLHVAIWEGHADVVCFLAPQSKLQHQDSQGRTPLELAQQLGLSHIQGILEQHLSIPG

>XP_037743952.1 cyclin-dependent kinase 4 inhibitor D [Chelonia mydas]

MLLGDEISAGDRLSGAAARGDLAELRRLLHQELVHPDSHNRFGKTALQVMMFGNTFVAEELLKQGASPNIQDEAGRAPAHDAARTGFLDTLRVLVEHGADVNVPDGAGALPVHVAVREGHTEVVRYLAPESNLRHRDAEGRTPLELARHLGLSHLEAILEQHLSAPA

>XP_039370047.1 cyclin-dependent kinase 4 inhibitor D [Mauremys reevesii]

MLLGDEISAGDRLSGAAARGDLTELRRLLHQELVHPDSHNRFGKTALQVMMFGNTFVAEELLKQGASPNIQDEAGRAPAHDAARTGFLDTLRVLVEHGADVNVPDGAGALPVHVAVREGHTEVVQYLAPESNLQHRDAEGRTPLELARHLGLSHLEAILGQHLSAPA

>XP_033029471.1 cyclin-dependent kinase 4 inhibitor D isoform X1 [Lacerta agilis]

MKSGRGERNNKARGGGAGGGGGGGGGAAADCGSCPGQARLQLGVVVGMQPGSAGDRLSGAAARGDTSEVRRLLFEELVQPDARNRFGKTALQVMMFGNVFVAQELLKQGANPNVRDESGTAPAHDAARTGFLDTLKILVEYGADVNVADASGSLPLHVAVREGHADVVLFLAPQSKLQHRDSDGRTPLELAQHLGLSHIQGILEQLLSAPA

>XP_034953385.1 cyclin-dependent kinase 4 inhibitor D [Zootoca vivipara]

MQPGSAGDRLSGAAARGDTSEVRRLLFEELVQPDARNRFGKTALQVMMFGNVFVAQELLKQGANPNVRDESGTAPAHDAARTGFLDTLKILVEYGADVNVADASGSLPLHVAVREGHADVVLFLAPLSKLQHRDSDGRTPLELAQHLGLSHIQGILEQLLSAPA

>XP_038234699.1 cyclin-dependent kinase 4 inhibitor D [Dermochelys coriacea]

MLLGDEISAGDRLSGAAARGDLAELRRLLHQELVHPDSHNRFGKTALQVMMFGNTFVAEELLKQGASPNIQDEAGRAPAHDAARTGFLDTLRVLVEHGADVNVPDGAGALPVHVAVREGHMEVVRYLAPESNLRHRDAEGRTPLELARHLGLSRLEAILEQHLSAPA

>XP_032636344.1 cyclin-dependent kinase 4 inhibitor D [Chelonoidis abingdonii]

MLLGDEISAGDRLSGAAARGDLAELRRLLHQELVHPDSHNRFGKTALQVMMFGNVFVAEELLKQGASPNIQDEAGRAPAHDAARTGFLDTLRVLVEHGADVNVPDGAGALPVHVAVREGHVEVVQYLAPESNLQHRDAEGRTPLELARHLGLSHLEAILGQHLSAPV

>XP_028568610.1 cyclin-dependent kinase 4 inhibitor D isoform X1 [Podarcis muralis]

MKWGRGERNNKARGGGAGGGGGGAAADCGSCPGQARLQLGRWGGVGLAACAGFTLCTDGAAPPPGFPQQQQPRPRLASRAPRRRRRPGLGIPAPPRPRRLLCRPRVARARGRSCAWRRSGPRRRLRAAGSADRPAAVRRDRGPCGGSKGVVAGMQPGSAGDRLSGAAARGDTGEVRRLLFEELVQPDARNRFGKTALQVMMFGNVFVAQELLKQGANPNVRDESGTAPAHDAARTGFLDTLKILVEYGADVNVADASGSLPLHVAVREGHADVVLFLAPQSKLQHRDSDGRTPLELAQHLGLSHIQGILEQLLSAPA

>XP_012397378.1 cyclin-dependent kinase 4 inhibitor D [Sarcophilus harrisii]

MLLEEISAGDRLSGAAARGDVNEVRRLLHQEFVHPDSLNRFGKTALQVMMFGSSAIALELLKQGASPNVQDGSGTSPAHDAARTGFLDTLRILVEHGADVNVPDGSGALPIHLAVREGHAAVVSFLAGESDLQHRDAGGLTPLELARQCGAGQLGRILERHLPTPSL

>XP_020850557.1 cyclin-dependent kinase 4 inhibitor D [Phascolarctos cinereus]

MLLEEISAGDRLSGAAARGDVNEVRRLLHQEFVHPDSLNRFGKTALQVMMFGSSPVALELLKQGASPNVQDGSGTSPAHDAARTGFLDTLRVLVEHGADVNVPDGSGALPIHLAVREGHAAVVSFLAGESDLQHRDAGGLTPLELARQCGAGQLGRILERHLPTPSL

>XP_036596688.1 cyclin-dependent kinase 4 inhibitor D [Trichosurus vulpecula]

MLLEEISAGDRLSGAAARGDVKEVRRLLHQEFVHPDSLNRFGKTALQVMMFGSSPIALELLKQGASPNVQDGSGTSPAHDAARTGFLDTLRVLVEHGADVNVPDGSGALPIHLAVREGHAAVVSFLAGESDLQHRDAGGLTPLELARQCGAGQLGRILERHLPTPSL

>XP_029773034.1 cyclin-dependent kinase 4 inhibitor D [Suricata suricatta]

MLLEEVRAGDRLSGAAARGDVQEVRHLLHRELVHPDALNRFGKTALQVMMFGSSTIASELLKQGASPNVQDTSGTTPAHDAARTGFLDTLKVLVEHGADVNVPDGTGALPIHLAVREGHTAVVSFLATESDLHHRDARGLTPLELAQGIGAQDLMDILQGHTVVLL

>XP_027713587.1 cyclin-dependent kinase 4 inhibitor D [Vombatus ursinus]

MLLEEISAGDRLSGAAARGDVNEVRRLLHQEFVHPDALNRFGKTALQVMMFGSSPIALELLKQGASPNVQDGSGTSPAHDAARTGFLDTLRVLVEHGADVNVPDGAGALPIHLAVREGHTAVVSFLAGESDLQHQDAGGLTPLELARQCGAGQLGRILERHLPTPSL

>XP_003981908.1 cyclin-dependent kinase 4 inhibitor D [Felis catus]

MLLEEVRAGDRLSGAAARGDVQEVRRLLHRELVHPDALNRFGKTALQVMMFGSSTIALELLKQGASPNVQDTSGTTPAHDAARTGFLDTLKVLVEHGADVNVPDGTGALPIHLAVREGHTAVVSFLASESDLHHRDARGLTPLELAQGIGAQDLMDILQGHSVVPL

>XP_022382638.1 cyclin-dependent kinase 4 inhibitor D [Enhydra lutris kenyoni]

MLLEEVRAGDRLTGAAARGDVQEVRRLLHCELVHPDALNRFGKTALQVMMFGSPTIALELLKQGASPNVQDATGTTPAHDAARTGFLDTLKVLVEHGADVNVPDGTGALPIHLAVREGHTAVVSFLAVESDLHHRDARGLTPLELAQGLGAQDLMDILQGHMVVLL

>XP_036171513.1 cyclin-dependent kinase 4 inhibitor D [Myotis myotis]

MLLEEVRAGDRLSGAAARGDVREVRRLLHRELVHPDALNRFGKTALQVMMFGSPTIALELLKQGASPNVQDTSGTTPAHDAARTGFLDTLKVLVEHGADVNAPDNTGSLPIHLAVQEGHTAVVSFLAAESDLHHRDTRGLTPLELAQGRGAQDLMDILQGHMVAPL

>NP_001009719.1 cyclin-dependent kinase 4 inhibitor D [Rattus norvegicus]

MLLEEVSVGDRLSGAAARGDVQEVRRLLHRELVHPDALNRFGKTALQVMMFGSPAVALELLKQGASPNVQDASGTSPVHDAARTGFLDTLKVLVEHGADVNTLDSTGSLPIHLAIREGHSSVVSFLAPESDLHHKDASGLTPLELARQRGAQNLMDILQSHMMIPM

>XP_029073486.1 cyclin-dependent kinase 4 inhibitor D [Monodon monoceros]

MLLEEVRAGDRLSGAAARGDVQEVRRLLHRELVHPDALNRFGKTALQVMMFGSPTVALELLKQGASPNVQDASGTTPAHDAARTGFLDTLKVLVEHGADVNAPDGTGALPIHLAVREGHTAVVGFLAAESDLQHRDVRGLTPLELARGRGAKDLMDILQWHTVAPL

>XP_025772408.1 cyclin-dependent kinase 4 inhibitor D [Puma concolor]

MLLEEVRAGDRLSGAAARGDVQEVRRLLHRELVHPDALNRFGKTALQVMMFGSSTIALELLKQGASPNVQDTSGTTPAHDAARTGFLDTLKVLVEHGADVNVPDGTGALPIHLAVREGHTAVVSFLASESDLHHRDARGLTPLELAQGIGAQDLMDILQGHSVVLL

>XP_032765966.1 cyclin-dependent kinase 4 inhibitor D [Rattus rattus]

MLLEEVSVGDRLSGAAARGDVQEVRRLLHRELVHPDALNRFGKTALQVMMFGSPAVALELLKQGASPNVQDASGTSPVHDAARTGFLDTLKVLVEHGADVNTLDSTGSLPIHLAIREGHSSVVSFLAPESDLHHKDASGLTPLELARQRGAQNLMDILQGHMMIPM

>XP_032025552.1 cyclin-dependent kinase 4 inhibitor D [Hylobates moloch]

MLLEEVRAGDRLSGAAARGDLQEVRRLLHRELVHPDALNRFGKTALQVMMFGSTAIALELLKQGASPNVQDTSGTSPVHDAARTGFLDTLKVLVEHGADVNAPDGTGALPIHLAVQEGHTAVVSFLAAESDLHRRDARGLTPLELALQRGAQDLVDILQRHMVAPL

>XP_005654916.1 cyclin-dependent kinase 4 inhibitor D [Sus scrofa]

MLLEEVCAGDRLSGAAARGDVQEVRRLLHRELVHPDVLNRFGKTALQVMMFGSPTIAQELLKQGASPNVQDASGTTPAHDAARTGFLDTLKVLVEHGADVNAPDGTGALPIHLAVREGHTSVVSFLAAESDLHHRDARGLTPLELARGRGAQDLMDILQRHTVAPL

>XP_032251893.1 cyclin-dependent kinase 4 inhibitor D [Phoca vitulina]

MLLEEVRAGDRLSGAAARGDVQEVRRLLHRELVHPDALNRFGKTALQVMMFGSPTIALELLKQGASPNVQDTTGTTPAHDAARTGFLDTLKVLVEHGADVNAPDGTGALPIHLAVREGHTAVVSFLATESDLHHRDAKGLTPLELAQGIGAQDLMDILQRHTVVLL

>XP_004378404.1 cyclin-dependent kinase 4 inhibitor D [Trichechus manatus latirostris]

MLLEEVRAGDRLSGAAARGDVQEVRRLLHRELIHPDALNRFGKTALQVMMFGSPTIALELLKQGASPNVQDISGTTPAHDAARTGFLDTLKVLVEHGADVNIPDGTGALPIHLAVREGHTAVVSFLAAESDLQHRDARGLTPLELAQQRGNRDLMDILQGHTVASL

>XP_004632903.1 cyclin-dependent kinase 4 inhibitor D [Octodon degus]

MLLEEVRAGDRLSGAAARGDAQEVRRLLHRELVHPDSLNRFGKTALQVMMFGSLAIARELLKQGASPNVQDASGTSPLHDAARTGFLDTLKVLVEHGADVNAPDGNGALPIHLAVREGHAAVVSFLAPESDLHHRDAAGLTPLELAQQRGAQDLMDILQGHKVIPL

>XP_021561028.1 cyclin-dependent kinase 4 inhibitor D [Neomonachus schauinslandi]

MLLEEVRAGDRLSGAAARGDVQEVRRLLHRELVHPDALNRFGKTALQVMMFGSPTIALELLKQGASPNVQDTTGTTPAHDAARTGFLDTLKVLVEHGADVNAPDGTGALPIHLAVREGHTAVVRFLATESDLHHRDARGLTPLELAQRIGAQDLMDILQRHTVVLL

>XP_027267315.1 cyclin-dependent kinase 4 inhibitor D [Cricetulus griseus]

MLLEEVRVGDRLSGAAARGDVQEVRRLLHRELVHPDALNRFGKTALQVMMFGSPAVALELLKQGASPNVQDASGTSPVHDAARTGFLDTLKVLVEHGADVNALDGTGSLPIHLAIKEGHSSVVSFLAPESDLHHRDTSGLTPLELAQQRGDQNLMDILQRHMVIPM

>XP_024601812.1 cyclin-dependent kinase 4 inhibitor D [Neophocaena asiaeorientalis asiaeorientalis]

MLLEEVRAGDRLSGAAARGDVQEVRRLLHRELVHPDALNRFGKTALQVMMFGSPTIAVELLKQGASPNVQDASGTTPAHDAARTGFLDTLKVLVEHGADVNAPDGTGALPIHLAVREGHTAVVGFLAAESDLHHRDARGLTPLELARGRGAKDLMDILQWHTVAPL

>XP_025719090.1 cyclin-dependent kinase 4 inhibitor D [Callorhinus ursinus]

MLLEEVRAGDRLSGAAARGDVQEVRRLLHRELVHPDALNRFGKTALQVMMFGSPTIALELLKQGASPNVQDTTGTTPAHDAARTGFLDTLKVLVEHGADVNVPDGTGALPIHLAVREGHTAVVSFLATESDLHHRDARGLTPLELAQGIGAQDLMDILQGHTVVLL

>XP_009251057.2 cyclin-dependent kinase 4 inhibitor D [Pongo abelii]

MLLEEVRAGDRLSGAAARGDVQEVRRLLHRELVHPDALNRFGKTALQVMMFGSTAIALELLKQGASPNVQDTSGTSPVHDAARTGFLDTLKVLVEHGADVNAPDGTGALPIHLAVQEGHTAVVSFLAAESDLHRRDARGLTPLELALQRGAQDLVDILQGHMVAPL

>XP_025149306.1 cyclin-dependent kinase 4 inhibitor D [Bubalus bubalis]

MLLEEVHAGDRLSGAAARGDVQEVRRLLHRELVHPDVLNRFGKTALQVMMFGSPTIALELLKQGASPNVQDASGTTPAHDAARTGFLDTLKVLVEHGADVNAPDGTGALPIHLAVREGHTSVVSFLATESDLHHRDARGLTPLELARGRGAQELMDILQRHTVAPL

>XP_001491963.1 cyclin-dependent kinase 4 inhibitor D [Equus caballus]

MLLEEVRAGDRLSGAAARGDVQEVRRLLHRELVHPDALNRFGKTALQVMMFGSSAIALELLKQGANPNIQDASGTTPAHDAARTGFLDTLRVLVEHGADVNAPDGTGALPIHLAVREGHNAVVSFLAAESDLHHRDARGLTPLELARGRGAQDLMDILQGHTVAPL

>XP_022413243.1 cyclin-dependent kinase 4 inhibitor D [Delphinapterus leucas]

MLLEEVRAGDRLSGAAARGDVQEVRRLLHRELVHPDALNRFGKTALQVMMFGSPTIALELLKQGASPNVQDASGTTPAHDAARTGFLDTLKVLVEHGADVNAPDGTGALPIHLAVREGHTAVVGFLAAESDLHHRDARGLTPLELARGRGAKDLMDILQWHTVAPL

>XP_003275794.1 cyclin-dependent kinase 4 inhibitor D [Nomascus leucogenys]

MLLEEVRAGDRLSGAAARGDLQEVRRLLHRELVHPDALNRFGKTALQVMMFGSTAIALELLKQGASPNVQDTSGTSPVHDAARTGFLDTLKVLVEHGADVNAPDGTGALPIHLAVQEGHTAVVSFLAAESDLHRRDARGLTPLQLALQRGAQDLVDILQRHMVAPL

>XP_039322986.1 cyclin-dependent kinase 4 inhibitor D [Saimiri boliviensis boliviensis]

MLLEEVCAGDRLSGAAARGDVQEVRRLLHRELVHPDALNRFGKTALQVMMFGSTTIALELLKQGASPNVQDTSGTSPVHDAARTGFLDTLKVLVEHGADVNAPDGTGALPIHLAVQEGHTAVVSFLAAESDLHLRDARGLTPLELAQQRGAQDVVDILQGHMVAPL

>XP_008156279.1 cyclin-dependent kinase 4 inhibitor D [Eptesicus fuscus]

MLLEEVRAGDRLSGAAARGDVQEVRRLLHRELVHPDALNRFGKTALQVMMFGSPAIALELLKQGASPNVQDASGTTPAHDAARTGFLDTLKVLVEHGADVNAPDNTGALPIHLAVREGHTAVVRFLAAESDLHHRDTRGLTPLELAQGRGAQDLMDILQGHMLAPL

>XP_040096653.1 cyclin-dependent kinase 4 inhibitor D [Oryx dammah]

MLLEEVHAGDRLSGAAARGDVQEVRRLLHRELVHPDVLNRFGKTALQVMMFGSPTIALELLKQGASPNVQDASGTTPAHDAARTGFLDTLKVLVEHGADVNAPDGTGALPIHLAVREGHTAVVSFLAAESDLHHRDARGLTPLELARGRGAQELMDILQRHTVAPL

>XP_032739887.1 cyclin-dependent kinase 4 inhibitor D [Lontra canadensis]

MLLEEVRAGDRLTGAAARGDVQEVRRLLHREFVHPDALNRFGKTALQVMMFGSPTIALELLKQGASPNVQDATGTTPAHDAARTGFLDTLKVLVEHGADVNAPDGTGALPIHLAVREGHTAVVSFLAMESDLHHRDARGLTPLELAQGLGAQDLMDILQGHTVVLL

>XP_021504854.1 cyclin-dependent kinase 4 inhibitor D [Meriones unguiculatus]

MLLEEVCGGDRLSGAAARGDVQEVRRLLHRELVHPDALNRFGKTALQVMMFGSPAVALELLKQGASPNVQDASGTSPVHDAARTGFLDTLKVLVEHGADVNAVDSSGSLPIHLAIREGHSSVVGFLAPESDLHYRDASGLTPLELARQRGAQNLMDILQGHMMIPM

>XP_028619742.1 cyclin-dependent kinase 4 inhibitor D [Grammomys surdaster]

MLLEEVCVGDRLSGAAARGDVQEVRRLLHRELVHPDALNRFGKTALQVMMFGSPAVALELLKQGASPNVQDASGTSPVHDAARTGFLDTLKVLVEHGADVNALDSTGSLPIHLAIREGHSSVVSFLAPESDLYHRDASGLTPLELARQRGAQNLMDILQGHMMIPM

>XP_028728283.1 cyclin-dependent kinase 4 inhibitor D [Peromyscus leucopus]

MLLEEVLAGDRLSGAAARGDVQEVRRLLHRELVHPDALNRFGKTALQVMMFGSPAVALELLKQGASPNVQDASGTSPVHDAARTGFLDTLKVLVEHGADVNVLDSTGSLPIHLAIREGHSSVVSFLAAESDLYHRDASGLTPLELARQRGDQNLMDILQGHMVIPL

>NP_001253450.1 cyclin-dependent kinase 4 inhibitor D [Macaca mulatta]

MLLEEVRSGDRLSGAAARGDVQEVRRLLHRELVHPDALNRFGKTALQVMMFGSTAIALELLKQGASPNVQDTSGTSPVHDAARTGFLDTLKVLVEHGADVNAPDGTGALPIHLAVQEGHTAVVSFLAAESDLHRRDARGLTPLELALQRGAQDLVDILQGHMVAPL

>NP_034008.2 cyclin-dependent kinase 4 inhibitor D [Mus musculus]

MLLEEVCVGDRLSGAAARGDVQEVRRLLHRELVHPDALNRFGKTALQVMMFGSPAVALELLKQGASPNVQDASGTSPVHDAARTGFLDTLKVLVEHGADVNALDSTGSLPIHLAIREGHSSVVSFLAPESDLHHRDASGLTPLELARQRGAQNLMDILQGHMMIPM

>XP_025839356.1 cyclin-dependent kinase 4 inhibitor D [Vulpes vulpes]

MLLEEVRAGDRLSGAAARGDVQEVRRLLHRELVHPDALNRFGKTALQVMMFGSPTIALELLKQGASPNVQDATGTTPAHDAARTGFLDTLKVLVEHGADVNAPDGTGALPIHLAVREGHTAVVSFLATESDLHHRDARGLTPLELAQGIGAQDLMDILQGHTVVLL

>XP_005336190.1 cyclin-dependent kinase 4 inhibitor D [Ictidomys tridecemlineatus]

MLLEEVRVGDRLSGAAARGDVQEVRRLLYRELVHPDALNRFGKTALQVMMFGSPAIALELLKQGASPNVQDASGTSPVHDAARTGFLDTLKVLVEHGADVNAPDGTGALPIHLAVQEGHSAVVSFLAPESDLQHKDARGLTPLELARQRGSQDIMDILQGCTVIPL

>XP_003461276.1 cyclin-dependent kinase 4 inhibitor D [Cavia porcellus]

MLLEEVRAGDRLSGAAARGDVQEVRRLLHRELVHPDALNRFGKTALQVMMFGSLAVAQELLKQGASPNVQDASGTSPLHDAARTGFLDTVKILVEHSADVNAPDGTGALPIHLAVREGHAAVVSFLAPESDLHHRDAAGLTPLELARQRGAQDLMDILQGHKVIPL

>XP_031202072.1 cyclin-dependent kinase 4 inhibitor D [Mastomys coucha]

MLLEEVCVGDRLSGAAARGDVQEVRRLLHRELVHPDALNRFGKTALQVMMFGSPAVALELLKQGASPNVQDASGTSPVHDAARTGFLDTLKVLVEHGADVNALDSTGSLPIHLAIREGHSSVVSFLAPESDLHHRDASGLTPLELAQQRGAQNLMDILQGHIMIPM

>XP_014951314.2 cyclin-dependent kinase 4 inhibitor D [Ovis aries]

MLLEEVHAGDRLSGAAARGDVQEVRRLLHRELVHPDVLNRFGKTALQVMMFGSPTIALELLKQGASPNVQDASGTTPAHDAARTGFLDTLKVLVEHGADVNAPDGTGALPIHLAVREGHTPVVSFLAAESDLHHRDARGLTPLELARGRGAQELMDILQRHTVAPL

>XP_034853130.1 cyclin-dependent kinase 4 inhibitor D [Mirounga leonina]

MLLEEVRAGDRLSGAAARGDVQEVRRLLHHELVHPDALNRFGKTALQVMMFGSPTIALELLKQGASPNVQDTTGTTPAHDAARTGFLDTLKVLVEHGADVNAPDGTGALPIHLAVREGHTAVVSFLATESDLHHRDARGLTPLELAQGIGAQDLMDILQRHTVVLL

>NP_001039515.1 cyclin-dependent kinase 4 inhibitor D [Bos taurus]

MLLEEVHAGDRLSGAAARGDVQEVRRLLHSELVHPDVLNRFGKTALQVMMFGSPTIALELLKQGASPNVQDASGTTPAHDAARTGFLDTLKVLVEHGADVNAPDGTGALPIHLAVREGHTSVVSFLATESDLHHRDATGLTPLELARGRGAQELMDILQRHTVAPL

>XP_006741117.1 cyclin-dependent kinase 4 inhibitor D [Leptonychotes weddellii]

MLLEEVRAGDRLSGAAARGDVQEVRRLLHRELVHPDALNRFGKTALQVMMFGSPTIALELLKQGASPNVQDTTGTTPAHDAARTGFLDTLKVLVQHGADVNAPDGTGALPIHLAVREGHTAVVSFLATESDLHHRDARGLTPLELAQGIGAQDLMDILQRHTVVLL

>XP_006184913.1 cyclin-dependent kinase 4 inhibitor D [Camelus ferus]

MLLEEVRAGDRLSGAAARGDVLEVRRLLHRELVHPDALNRFGKTALQVMMFGSPTIALELLKQGASPNVQDASGTTPAHDAARTGFLDTLKVLVDHGADVNVPDGTGALPIHLAVREGHTAVVSFLAAESDLHHRDARGLTPLELARGRGAQDLMDILQGHPVAPL

>XP_039106849.1 cyclin-dependent kinase 4 inhibitor D [Hyaena hyaena]

MLLEEVRAGDRLSGAAARGDVQEVRRLLHHELVHPDALNRFGKTALQVMMFGSPTIALELLKQGASPNVQDTSGTTPAHDAARTGFLDTLRVLVEHGADVNVPDGTGALPIHLAVREGHTAVVSFLATESDLHHRDARGLTPLELAQGIGAQDLMDILQGHPVVLL

>XP_020757885.1 cyclin-dependent kinase 4 inhibitor D [Odocoileus virginianus texanus]

MLLEEVHAGDRLSGAAARGDVQEVRRLLHRELVHPDVLNRFGKTALQVMMFGSPTIALELLKQGASPNVQDASGTTPAHDAARTGFLDTLKVLVEHGADVNAPDGTGALPIHLAVSEGHTPVVSFLAAESDLHHRDARGLTPLELARGRGAQELMDILQRHTVAPL

>XP_004865564.1 cyclin-dependent kinase 4 inhibitor D isoform X1 [Heterocephalus glaber]

MMLEEVRAGDRLSGAAARGDVQEVRRLLYRELVHPDALNRFGKTALQVMMFGSLTIAQELLKQGASPNIQDASGTSPVHDAARTGFLDTLKVLVEYGADVNMPDGTGSLPIHLAVREGHASVVSFLAPESDLHHRDAAGLTPLELARQRGAQDLLDILQGHKRRALLGDRHLNGSVSSAPQGCRHDPRAER

>XP_036315363.1 cyclin-dependent kinase 4 inhibitor D [Pipistrellus kuhlii]

MLLEEVRAGDRLSGAAARGDVREVRRLLHRELVHPDALNRFGKTALQVMMFGSPTIALELLKQGASPNVQDAAGTTPAHDAARTGFLDTLKVLVEHGADVNAPDNTGALPIHLAVREGHAAVVSFLAAESDLHHRDTRGLTPLELARGRGAQDLMDILQGHMVAPL

>XP_011611390.2 cyclin-dependent kinase 4 inhibitor D [**Takifugu rubripes**]

MVIGQMDAGKALAAAAAKGRTSEVQRILEECRVPPDTRNEFGKTALQVMMLGNCKIASLLLEKGADPNVQDKHGIAPVHDAARTGFLDTLQVLVEYGASVNLPDQSGALPIHIAIREGHRDVVEFLAPRSDLKHANKSGQTAADVARASRVPDMMDLLFSHVHR

>XP_031727870.1 cyclin-dependent kinase 4 inhibitor D [Anarrhichthys ocellatus]

MVLSQMDAGKALTSAAANGNTSEVQRILEECRVHPDTLNEFGRTALQVMMMGNSKVASLLLEKGADPNVQDKHGIAPVHDAARTGFLDTLQVLVEYGASVNLPDQSGALPIHIAIREGHRDVVEFLAPRSDLKHANRSGQTAIDVARASCVPDMMDLLFAHIHS

>XP_029135867.1 cyclin-dependent kinase 4 inhibitor D [Labrus bergylta]

MVLSQMDAGRALTSAAAKGNSSEVQRILEECRVHPDTLNEFGRTALQVMMMGNSKIAGLLLEKGADPNIQDKHGIAPVHDAARTGFLDTLRVLVDYGASVNVSDQSGALPIHIAIREGHRDVVEFLAPRSDLKHANISGQTAIDVARASRVPDMMDLLFAHIHS

>XP_020452720.1 cyclin-dependent kinase 4 inhibitor D [Monopterus albus]

MVLSQMDAGKALTSAAAKGNTSEVQRILEEYRVHPDTLNEFGRTALQVMMMGNSKIASLLLEKGADPNVQDRHGIAPVHDAARTGFLDTLQVLVEYGASVNVPDQSGALPIHIAIREGHRDVVEFLAPQSDLKHANISGQTAIDVARASCVPDMIDLLFAHIHS

>XP_033938436.1 cyclin-dependent kinase 4 inhibitor D [Pseudochaenichthys georgianus]

MVLNQMDDGKALTAAAAKGDRSEVRRILEECRVHPDNLNEFGRTALQVMMMGNSKVASLLLEKGADPNVQDKHGIAPVHDAARTGFLDTLQVLVEYGASVNVPDMKGALPIHIAIKEGHRDVVEFLAPRSDLKHANISGQTAIDVARASCAPDMIELLYSHIHS

>XP_034059176.1 cyclin-dependent kinase 4 inhibitor D [Gymnodraco acuticeps]

MVLNQMDDGKALTAAAAKGDRSEVRRILEECRVHPDNLNEFGRTALQVMMMGNSKVASLLLEKGANPNVQDKHGIAPVHDAARTGFLDTLQVLVEYGASVNIPDMKGALPIHIAIKEGHRDVVEFLAPHSDLKHANISGQTAIDVARASCAPDMIELLYSHIHS

>XP_034390041.1 cyclin-dependent kinase 4 inhibitor D [Cyclopterus lumpus]

MVISQMDAGKALTAAAAKGSTSEVQRILEECRVHPDTLNEFGRTALQVMMMGNSKIASLLLEKGADPNVQDKHGIAPIHDAARTGFPDTLQVLVEYGASVNLPDQSGALPIHIAIREGHRDVVEFLAPRSDLKHANISGQTAVDVARTSCVPDMMDLLFAHIHS

>XP_022046310.1 cyclin-dependent kinase 4 inhibitor D [Acanthochromis polyacanthus]

MVLSQMDAGKALTTAAARGNTGEVQWILEECRVHPDTVNEFGRTALQVMMMGNSKIATLLLEKGAEPNIQDKHGIAPIHDAARTGFLDTLQVLVEYGASVNMPDQNGTLPIHIAIQEGHLDVVKFLAPQSDLKHANVSGQTAIDVARASCVPDMINSLFAHIHS

>XP_033478989.1 cyclin-dependent kinase 4 inhibitor D [Epinephelus lanceolatus]

MVFSQMDAGKALTAAAARGNTSEVQRILEECRVHPDTLNEFGRTALQVMMMGNSKVASLLLEKGADPNVQDKHGIAPVHDAARTGFLDTLQVLVEYGASVNIPDHSGALPIHIAIREGHRDVVEFLAPRSDLKHTNISGQTAVDVARASCVPNMIDLLFAHIHS

>XP_035515227.1 cyclin-dependent kinase 4 inhibitor D [Morone saxatilis]

MVLSQMDAGKALTAAAARGNSSEVQRILEECRLHPDTLNEFGRTALQVMMMGNSKVASLLLEKGADPNMQDKHGIAPVHDAARTGFLDTVQVLVEYGASVNIPDQSGALPIHIAIREGHRDVVEFLAPRSDLKHANVSGQTAIDVARASCVPDMIDLLFAHIHS

>XP_019129790.1 cyclin-dependent kinase 4 inhibitor D [Larimichthys crocea]

MVLSQMDAGKALTAAAAKGNTGEVQRILEECRVHPDTRNEFGRTALQVMMMGNSKVASLLLEKGADPNLQDKHGIAPVHDAARTGFLDTLQVLVEYGASVNIPDKSGALPIHIAIREGHRDVVEFLAPRSDLKHANISGQTAIDVARASCVPDMMELLFAHIHS

>TNN83618.1 Cyclin-dependent kinase 4 inhibitor D [Liparis tanakae]

MVISQMDAGKALTAAAAKGSTSEVQRILEECRVHPDTLNEFGRTALQVMMMGNSKIASLLLEKGADPNVQDKHGIAPVHDAARTGFPDTLQVLVEYGASVNLPDHSGALPIHIAIREGHRDVVEFLAPRSDLKHANISGQTAVDVARTSCVPDMMDLLFAHIHS

>XP_030288492.1 cyclin-dependent kinase 4 inhibitor D [Sparus aurata]

MVLSQMDAGKALTAAAAKGKTSEVQRILEECRVHPDTLNEFNRTALQVMMMGNSKVASLLLEKGADPNVQDKHGIAPVHDAARTGFLDTLQVLVEYGASVNIPDQSGALPIHIAIREGHRDVVEFLAPRSDLKHANISGQTAIDVARASCVPDMIDLLFAHIHS

>XP_028460267.1 cyclin-dependent kinase 4 inhibitor D [Perca flavescens]

MVLSQMDAGKALTAAAAQGNTSEVQRILDECRLHPDTRNEFGRTALQVMMMGNSKIASLLLEKGADPNVQDKHGIAPVHDAARTGFLDTLQVLVEYGASVNIPDQSGALPIHIAIREGHLDVVEFLAPRSDLKHANISGQTAIDVARASCMPAMIDLLFAHIHS

>XP_036964687.1 cyclin-dependent kinase 4 inhibitor D isoform X1 [Acanthopagrus latus]

MFVNCSLETPLCRSQRANVFSNVYKPSLFGFYLKITGLQKQRDRKIVSALWISRKRTRQAERENIMVLSQMDAGKALTAAAAKGKTSEVQRILEECRVHPDTLNEFNRTALQVMMMGNSKVASLLLEKGADPNVQDKHGIAPVHDAARTGFLDTLQVLVEYGASVNIPDQSGALPIHIAIREGHRDVVEFLAPRSDLKHANISGQTAIDVARASCVPDMIDLLFAHIHS

>XP_038569590.1 cyclin-dependent kinase 4 inhibitor D [Micropterus salmoides]

MVLSQMDAGKALTAAAAKGNTSEVLRILEECRVHPDTLNEFGRTALQVMMMGNSKIASLLLEKGADPNVQDKHGIAPIHDAARTGFLDTVQVLVEYGALVNIPDQSGALPIHIAIREGHRDVVEFLAPRSDLKHANVSGQTAIDVARASRLPDMIDLLFAHIHS

>TKS68027.1 Cyclin-dependent kinase 4 inhibitor D p19-INK4d [Collichthys lucidus]

MVLSQMDAGKALTAAAAKGNTGEVQRILEECRVHPDTRNEFGRTALQVMMMGNSKVAALLLEKGADPNLQDKHGIAPVHDAARTGFLDTLQVLVEYGASVNIPDKSGALPIHIAIREGHRDVVEFLAPQSDLKHANASGQTAIDVARASCVPDMMELLFAHIHS

>XP_030208752.1 cyclin-dependent kinase 4 inhibitor D isoform X1 [Gadus morhua]

MVLSQMDAGRALTSAAAKGNTNDVRRILEECRVPPDTVNEFGRTALQVMMMGNTKIASLLLEHGANPNAQDRHGITPAHDAAQTGFLETLQVLVEHGASVNIPDKNGTLPIHIAIREGHRDVVEFLAPRSDLKHANTSGHTAVEVARASCLPDMIDLVFAHIHS

>XP_030002667.1 cyclin-dependent kinase 4 inhibitor D [Sphaeramia orbicularis]

MVLRQMDAGKALTAAAAKGSTSEVQRILEECRVHPDTLNEFGRTALQVMMMGNSKVASLLLEKGADPNVQDKHGIAPVHDAARTGFLDTVQVLVEYGAAVNIPDQTGALPIHIAIREGHRDVVEFLAPRSDLKHANISGQTAIDVARASCVPDMIDLLFAHIHS

>XP_039671988.1 cyclin-dependent kinase 4 inhibitor D [Perca fluviatilis]

MVLSQMDAGKALTAAAAQGNTSEVQRILDECRLHPDTLNEFGRTALQVMMMGNSKIASLLLEKGADPNVQDKHGIAPVHDAARTGFLDTLQVLVEYGASVNIPDQSGALPIHIAIREGHLDVVEFLAPRSDLKHANISGQTAIDVARASCMPAMIDLLFAHIHS

>XP_008315814.1 cyclin-dependent kinase 4 inhibitor D isoform X1 [Cynoglossus semilaevis]

MFVNCAAVRIATRTIILNVNKHCVYNLNLKITGFGKSGTRISTTLLDHQKPNKASGQNIMVFIEMDAGSALTTAAAKGNTTEVQKILEERRIHPNALNQFGRTALQVMMMGNSQIARLLLEKGAQPNIQDRHGIAPVHDAARTGFLDTVGVLVEYGASVNIPDQSGALPIHIAIREGHRDVVEFLAPRSDLKHANTSGQTAIDVARACRVPDMIDLLYAHIHC

>XP_032380931.1 cyclin-dependent kinase 4 inhibitor D [Etheostoma spectabile]

MVLSQMDAGKALTAAAAKGNTSEVQRILDECRLHPDTLNEFGRTALQVMMMGNSKIASLLLEKGADPNVQDKHGIAPVHDAARTGFLDTLQVLVEYGASVNIPDQSGALPIHIAIREGHLDVVEFLAPRSDLKHANISGQTAIDVARASCTPAMIDLLFSHIHS

>XP_037619760.1 cyclin-dependent kinase 4 inhibitor D [Sebastes umbrosus]

MVLSQMDAGKALTAAAAKGSTSEVQRILEECRVHPDTLNEFGRTALQVMMMGNSKIAGLLLEKGADPNVQDKHGIAPVHDAARTGFPDTLQVLVEYGASVNIPDQRGALPIHIAIREGHRDVVEFLAPRSDLKHANISGQTAIDMARASGVPDMIDLLFAHIHS

>XP_029298294.1 cyclin-dependent kinase 4 inhibitor D [Cottoperca gobio]

MVLSQMDAGKALTAAAAKGNTSEVQRILEECRLHPDTLNDFGRTALQVMMMGNSKVASLLLEKGADPNIQDKHGIAPVHDAARTGFVDTLQVLVEYGASVNIPDQRGALPIHIAIREGHRDVVEFLAPLSDLKHANISGQTAIDVARASCVPDMIDLLFSHIHS

>XP_029910591.1 cyclin-dependent kinase 4 inhibitor D [Myripristis murdjan]

MVLSQMDAGKALTAAAANGNTSEVQRILEEYRVHPDTLNEFGRTALQVMMMGNSKVATLLLEYGADPNIQDKHGIAPVHDAARTGFLDTLRVLVEYGASVNIPDQSGALPIHIAIREGHRDVVEFLAPRSNLKHVNTSGQTAVDVARASCVPDMIDLVFAHVHS

>XP_026214739.1 cyclin-dependent kinase 4 inhibitor D [Anabas testudineus]

MVLSQMDAGKALTAAAAKGNTGEVRRILEECRVHPDTLNEFGRTALQVMMMGNSKIATLLLEKGADPNIQDKQGITPVHDAARTGFLDTVQVLVEYGASVNMPDHSGALPIHIAIREGHRDVVEFLAPRSDLKHANVSGQTAIDVARASRVPDMIDLLFAHIHS

>XP_033826815.1 cyclin-dependent kinase 4 inhibitor D [Periophthalmus magnuspinnatus]

MVLRQMDAGKCLTAAAATGNTREVQRMMEEGRVHPDTTNEYGRTALQVMMMGNPKVASLLLQKGANPNVQDKYGISPAHDAARTGFVDTLQVLVEHGASVNVADKNGALPIHIAIREGHRDVVAFLAPRSDLKHVNTSGQTAVDVARASRVPDMVDLLFAHVHS

>XP_034544492.1 cyclin-dependent kinase 4 inhibitor D [Notolabrus celidotus]

MVLSQMDAGKALTAAAAKGDSSEVQRILEDCRVHPDTLNEFGRTALQVMMMGNSKVASLLLEKGADPNIQDKHGIAPVHDAARTGFLDTLQVLVEYGASVNVSDQSGALPIHIAIHEGHRDVVEFLAPRSDLKHANISGQTAIDVARASCVPDMIDLLFAHIHS

>XP_040003332.1 cyclin-dependent kinase 4 inhibitor D [Xiphias gladius]

MVLSQMDAGKALTAAAAKGNTSEVQRILEESRVHPDTLNEFGMTALQVMMMGNSKIASLLLEKGADPNIQDKHGIAPVHDAARTGFVDTLQVLVEYGASVNLPDQSGALPIHIAIRGGHRDVVEFLAPRSDLKHATVSGQTAIDVARASCVPDMIDLLFAHIHS

>XP_030599905.1 cyclin-dependent kinase 4 inhibitor D [Archocentrus centrarchus]

MVLSQMDAGKALTAAAAKGNTSEVQRILEECRVHPDTLNEFGRTALQVMMMGNSKIASLLLEKGAEPNIQDRHGITPVHDAARTGFLDTVQVLVEYGASVNIPDQNGALPIHIAIREGHRDVVKYLAPLSDLKHANISGQTAIDVARASCVPDMIDLLFSHVHS

>XP_011472587.1 cyclin-dependent kinase 4 inhibitor D isoform X1 [Oryzias latipes]

MCFNGPTVVDFHGVRGACAVEQARTEFIMVLSQMDAGKALTAAAAKGNTSEVQRILEECRVHPDTRNEFGRTALQVMMMGNSKIASLLLEKGADANVQDKHGIAPVHDAARTGFLDTLQVLVENGASVNIPDQNGALPIHIAIWEGHRDVVQFLAPRSNLKHANQSGQTAIDVARASCVPDMMDSLFAHIHS

>XP_024146139.1 cyclin-dependent kinase 4 inhibitor D [Oryzias melastigma]

MVLSQMDAGKALTAAAAKGNASEVQRILEECRVHPDTRNEFGRTALQVMMMGNSKIAGLLLEKGADPNVQDKHGIAPVHDAARTGFLDTLQVLVENGASVNIPDQNGALPIHIAIWEGHRDVVQFLAPRSDLKHANQSGQTAIDVARASCVPHMMDSLFAHIHS

>XP_004567929.1 cyclin-dependent kinase 4 inhibitor D [Maylandia zebra]

MVLSQMDAGKALTAAAAKGNTSEVQRILEECRVHPDTPNEFSRTALQVMMMGNSKIASLLLEKGANPNVQDRHGITPVHDAARTGFLDTVEVLVEYGASVNIPDRSGALPIHIAIREGHRDVVKYLAPRSNLKHANVSGQTAVDVARASGVPDMIDLLFSHIHS

>XP_034044801.1 cyclin-dependent kinase 4 inhibitor D [Thalassophryne amazonica]

MMVFSQMEAGKALTTAAANGRTVEVQMILEELRVHPDTPNEFGRTALQVMMMGSPKIATLLLQNGADANIQDKHGIAPVHDAARTGFLDTLQVLVEFGASVNLPDHRGALPIHIAIREGHCNIVEFLAPRSDLKHANTSGQTAVDVARASCVPGMIDLLFAHIHS

>XP_031597072.1 cyclin-dependent kinase 4 inhibitor D [Oreochromis aureus]

MVLSQMDAGKALTAAAAKGNTSEVQRILEECRVHPDTPNEFSRTALQVMMMGNSKIASLLLEKGANPNVQDRHGITPVHDAARTGFLDTVEVLVEYGASVNIPDKSGALPIHIAIREGHRDVVKYLAPRSNLKHANVSGQTAIDVARASCVPDMIDLLFSHIHS

>XP_031141822.1 cyclin-dependent kinase 4 inhibitor D [Sander lucioperca]

MVLSQMDAGKALTAAAAKGNTSEVQRILDECRLHPDTLNEFGRTALQVMMMGNSKIACLLLEKGADPNVQDKHGIAPVHDAARTGFLDTLQVLVEYGASVNIPDQNGALPIHIAIREGHLDVVEFLAPRSDLKHANISGQTAIDVARASCMPAMIDLLFAHIHS

>XP_037328840.1 cyclin-dependent kinase 4 inhibitor D [Pungitius pungitius]

MVLSQMDAGKALTAAAANGSTSEVLRILEECRVHPDTLNEFGRTALQVMMMGNSKIAGLLLEKGADPNVQDKHGIAPVHDAARTGFPDTVRVLVEYGASVNLPDQSGALPIHIAIREGHRGVVEFLAPLSDLKHANVSGQTAIDVARASCVPDIMDLLFAHIHS

>XP_017285945.1 cyclin-dependent kinase 4 inhibitor D [Kryptolebias marmoratus]

MVLSQMDAGKALTAAAAKGNAEEVQRILEECRVHPDTLNEFGRTALQVMMMGNAKIASLLLEKGADPNVQDKHGIAPAHDAARTGFLDTLQVLVEHGASVNLPDQGGALPIHIAIREGHRDVVEFLAPRSDLKHATASGQTAIDVARASCVPDMMNSLFAHIHS

>XP_006787454.1 cyclin-dependent kinase 4 inhibitor D isoform X1 [Neolamprologus brichardi]

MEAQHPLGELRLASAGRQTIMVLSQMDAGKALTAAAAKGNTSEVQRILEECRVHPDTPNEFSRTALQVMMMGNSKIASLLLEKGANPNVQDRHGITPVHDAARTGFLDTVEVLVEYGASVNIPDRSGALPIHIAIREGHRDVVKYLAPRSNLKHANVSGQTAVDVARASCVPDMIDLLFSHIHS

>XP_040042662.1 cyclin-dependent kinase 4 inhibitor D [Gasterosteus aculeatus aculeatus]

MVLSQMDAGKALTAAAANGSTSEVQRILMECRVPADTLNEFGRTALQVMMMGNSKIAGLLLEKGADPNVQDNHGIAPVHDAARTGFPDTLRVLVEYGASVNLPDHSGALPIHIAIREGHRDVVEFLAPLSDLKHANLSGQTAIDVARASCAPDIMDLLFAHIHS

>XP_003447928.1 cyclin-dependent kinase 4 inhibitor D [Oreochromis niloticus]

MVLSQMDAGKALTAAAAKGNTSEVQRILEECRVHPDTPNEFSRTALQVMMMGNSKIASLLLEKGANPNVQDRHGITPVHDAVRTGFLDTVEVLVEYGASVNIPDKSGALPIHIAIREGHRDVVKYLAPRSNLKHANLSGQTAIDVARASCVPDMIDLLFSHIHS

>ACB46844.1 cyclin-dependent kinase inhibitor 2D [Nothobranchius kuhntae]

MVLSQMEAGKALTAAAAKGNTDEVQRILEECRVHPDTVNEFGRTALQVMMMGNSKIASLLLEKGADPNLQDRHGIAPIHDAARTGFLDTLQVLVEHGASVNLPDHNGALPIHIAIREGHRDVVKFLAPQSDLKHANTSGQTAVDAARASCVPDMMDSLFAHVHSS

>XP_026160042.1 cyclin-dependent kinase 4 inhibitor D [Mastacembelus armatus]

MFVNCRSETRLCMSQRANVFSNVDKPSSFGFSLKISGLWRQRARKSWRRRGSRRPEQGRHTIMVLREMDAGKALTAAAAKGNNGEVRRILEESRVHPDTLNEFGRTALQVMMMGNSKIASLLLEKGANPNVQDRHGIAPIHDAARTGFLDTVQVLVEYGASVNIPDQSGALPIHIAIREGHREVVEFLAPRSDLKHANTSGQTAIDVARASRVPDMINLLFAHIHS

>XP_023274871.1 cyclin-dependent kinase 4 inhibitor D [Seriola lalandi dorsalis]

MVLSQMDAGKALTAAAAKGNTSEVQRILEESRVHPDTLNEFGRTALQVMMMGNSKIASLLLEKGADPNVQDKHGIAPVHDAARTGFLDTLQVLVEYGASVNIPDQSGALPIHIAIREGHRDVVEFLAPQSDLKHTNISGQTAIDVARCVPDMIDLLFTDIHS

>XP_022622577.1 cyclin-dependent kinase 4 inhibitor D isoform X1 [Seriola dumerili]

MRSPWLSFSVVNCSTSPTEQAGRERIMVLSQMDAGKALTAAAAKGNTSEVQRILEESRVHPDTLNEFGRTALQVMMMGNSKIASLLLEKGADPNVQDKHGIAPVHDAARTGFLDTLRVLVEYGASVNIPDQSGALPIHIAIREGHRDVVEFLAPQSDLKHANISGQTAIDVARCVPDMIDLLFTDIHS

>XP_023128546.1 cyclin-dependent kinase 4 inhibitor D [Amphiprion ocellaris]

MVLSQMDAGKALTAAAARGNTGEVQWILEECRVHPDTVNEFGRTALQVMMMGNSKIASLLLEKGAEPNVQDKHGIAPIHDAARTGFLDTLQVLVEYGASVNIPDQNGTLPIHIAIQEGHLEVVKFLAPQSDLKHANISGQTAIDVARASCVPDMINSLFAHIHS

>KAF3687561.1 Cyclin-dependent kinase 4 inhibitor D p19-INK4d [Channa argus]

MVFSQMDAGKALTAAAAKGDAGEVQRNLEECGVHPDTLNEFGRTALQVMMMGNSKIASLLLEKGADPNIQDKHGIAPVHDAARTGFLDTLKVLVEFGASVNIPDHNGALPIHIAIREGHRDVVEFLAPRSDLKHANISGQTAIDVARASCVPDMIDLLFAHIHS

>XP_029365622.1 cyclin-dependent kinase 4 inhibitor D [Echeneis naucrates]

MMVLSQMDAGKALTAAAAKGNRTEVQRILEESRVHPDTPNEFGMTALQVMMMGNSKIASLLLEKGADPNVQDKYGIAPVHDAARTGFLDTLRVLVEYGASVNTPDQSGALPIHIAIREGHRKVVEFLAPQSNLKHANISGQTAIDVARASGAPDMIDLLFAYIHS

>XP_028261815.1 cyclin-dependent kinase 4 inhibitor D [Parambassis ranga]

MVLSQMDAGKALTAAAAKGNTSEVQRILEECRVHPDTLNEFGKTALQVMMMGNSKIASLLLEKGANPNIQDRHGITPARDAACTGFLDTLQILVEYGASVNIPDQSGTLPIHIAIQEGHRDVVKFLAPRSDLKHANVSGQTAIDVARASCEPDMIDSLFAHIHS

>XP_037530338.1 cyclin-dependent kinase 4 inhibitor D [Nematolebias whitei]

MVLSQMDAGKALTAAAAKGNPEEVQRILEELRVHPDTLNEFGRTALQVMMMGNAKIAGLLLEKGADPNVQDKHGIAPAHDAARTGFLDTLQVLVEHGASVNLPDQNGALPIHIAIREGHRDVVEFLAPQSDLKHATVSGQTAVDVARASCVPHMMDALFAHVHS

>XP_029014837.1 cyclin-dependent kinase 4 inhibitor D [Betta splendens]

MVLSQMDAGKALTAAAAKGNTGEVLRILEECRVHPDTLNEFGRTGLQVMMMGNSKVATLLLEKGADPNVQDKQGITPLHDAARTGFLDTVQVLVEYGALVNMPDHNGALPIHIAIREGHRDVVEFLAPRSDLKHTNIHGQTAIDVARSSRVPNMIDLLFAHIHS

>XP_028307248.1 cyclin-dependent kinase 4 inhibitor D [Gouania willdenowi]

MVLSQMEAGKALAAAAAKGDTGEVQRILEECRVHPDTLNEFGRTALQVMMMGNSQIATLLLEKGADPNVQDKHGVAPVHDAAQTGFLDTLQVLVEYGASVNASDQNGSLPIHIAIREGHRDVVKFLAPRSDLAHTNTSGQTVIDMARASRVPDMIDLICAHLHS

>XP_034450015.1 cyclin-dependent kinase 4 inhibitor D [Hippoglossus hippoglossus]

MFVNCSLATPQCGSHRANVFSNVDKLRLLGFHLKTRLDGLRKERDSNLCDAVDLEPKQAAADGIMVRCPMETGEDLTAAAARGSTSEVRRILEQSGVHPDTVNEFGRTALQVMMMGNTKIATMLLEKGAEPNVQDRHGIAPVHDAARTGFLDTVLALVAHGASVNIPDQSGTLPIHIAIRQGHRDVVEFLAPRSDLRHANLSGQTAIDVARASGEPDMIDLVFAHIHR

>XP_020794159.1 cyclin-dependent kinase 4 inhibitor D [Boleophthalmus pectinirostris]

MVLSQMDAGKALTAAAAKGNTSEVQRMLDECRVHPDTRNEFXXXXVQVMMMGNPKVACLLLQKGANPNLQDKHGISPAHDAARTGFLDTLRVLVEHGASVNVADNNGALPIHIAIREGHRDVVTFLAPRSDLKHANNSGQTAVDVARASRVPDMVDLLFAHIHN

>XP_005797577.1 cyclin-dependent kinase 4 inhibitor D [Xiphophorus maculatus]

MVLSQMDAGKALTAAAAKGNADEVQRILEECRVHPDTPNEFGRTALQVMMMGNSKVARLLLEKGAEPNVQDKHGIAPVHDAAQTGFLETLQVLVEHGASVNIQDQNGALPIHIAIREGHRDIVEFLAPRSDLKHANVSGQTAIDVARSLGELDMMNSLFAHIHS

>XP_012705944.1 cyclin-dependent kinase 4 inhibitor D [Fundulus heteroclitus]

MVLSQMDAGKALTAAAARGNADEVQRLLEECRVHPDTPNEFGRTALQVMMMGNSKVAGLLLEKGAEPNVQDKYGIAPVHDAARTGFLETLQVLVEHGASVNLQDQNGALPIHIAIREGHREVVEFLAPLSDLKQATVSGQTAIDMARALGELDMMNSLFAHIHS

>XP_026989323.1 cyclin-dependent kinase 4 inhibitor D [Tachysurus fulvidraco]

MVLSESDAGKSLTAAAAKGDTAEVRRLLEDRRVHPDTRNEFGKTALQVMMMGNPNVACLLLENGADPNIQDRFGITPVHDAARTGFLDTLCVLVDYGASVNIPDQSGALPIHIAIREGYRDVVEFLAPRSNLGHQDTRGDTALDIAEASCTPDMVELLKRQLESSLAFQS

>XP_026789085.1 cyclin-dependent kinase 4 inhibitor D [Pangasianodon hypophthalmus]

MVLSESDAGKSLTAAAARGDTAEVRRLLEESRVHPDTRNEFNKTALQVMMMGNTNVACLLLENGADPNIQDRFGITPAHDAARTGFLDTLCVLVDYGASVNIPDQSGALPIHIAIREGYRDVVEFLAPRSNLGHQDTRGDTALDIAKASCTPGMVELLKRQLESSLAFQS

>XP_012694315.1 cyclin-dependent kinase 4 inhibitor D [Clupea harengus]

MVLSENDAGKGLTTAAAKGNTAEVRRMLEECRVHPDTVNEFGRTALQVMMMGNTNVACLLLEHGADPNIQDRFGVTPAHDASRGGFLDTLRALVDFGASVNVPDSSGALPIHIAIREGYRDVVEYLAPRSNLSHHNTSGETALDVARASCTPDVVELLERQLESSLTFKS

>XP_030638678.1 cyclin-dependent kinase 4 inhibitor D [Chanos chanos]

MVLSENDAGKNLTAAAAKGNVAEVRRMLEECRVHPDTVNEFGKTALQVMMMGNTNVACLLLENGADPNIQDCFGVTPAHDAARTGFLDTLRALVLYGASVNVPDQSGALPIHIAIREGHMDVVEYLAPRSNLGHHDTSGDTALDVARATCPPDVVELLERQLEYSTMYQSSQ

>XP_010871084.1 cyclin-dependent kinase 4 inhibitor D [Esox lucius]

MVLSQSDAGKSLTTAAAKGNTDEVRKILEECRVPADTVNEFGKTALQVMMMGNSNVACLLLEHGANPNITDKRGISPAHDAAHTGFLDTLQVLVEFGASVNTADHSGSLPIHVAVREGYRDIVEFLAPRSNLKHPNASGETPVDLARASASCTPEVVELLERQLESQVFSQSPSLI

>XP_037130041.1 cyclin-dependent kinase 4 inhibitor D [Syngnathus acus]

MVHMHGARGLTGAAARGNLDEVRRILEECRLHPDTVNEFGRTALQVMMMGNSKVARLLLERGADPNVQDAGGTAPVHDAARTGFVDTLQVLVEHGASVNVADHGGALPIHLAIREGHRHAVEFLAPLSDLGRANAHGQTAVDVARASHRPDMIRLLFAHIHS

>XP_026869396.1 cyclin-dependent kinase 4 inhibitor D [Electrophorus electricus]

MVLSENDAGKRLTSAAAKGDVADVRRMLEECRVHPDTINEFGKTALQVMMMGNNNVACLLLENGADPNIQDNFGITPAHDAARTGFLDTLHVLVDYGASVNIPDKSGALPIHIAIREGYRDVVEYLAPRSNLGHQDTRGDTALDIAMATCTPDIVELLKRQLESTLA

>XP_036409347.1 cyclin-dependent kinase 4 inhibitor D-like [Megalops cyprinoides]

MVLSERDAGKNLSTAAARGKVQEVRRMLEEHRVHPDTVNEFGKTALQVMMMGSTNVACLLLEHGANANVQDRHGVTPAHDAARTGFVDTLRVLVEFGASVNTPDNAGALPIHIAIREGHTDVVEFLAPRSNLSHQDTSGDTALDVARAARIPHVVELLERQMESSLKFQA

>XP_017548381.1 cyclin-dependent kinase 4 inhibitor D [Pygocentrus nattereri]

MVLSEKEAGRRLTSAAARGDAAEVRRVLEDCRVHPDTVNEFGKTALQVMMMGNTSVACLLLESGANPNIQDRFGITPAHDAARTGFLDTLRVLVDFGADVNVPDQSGVLPIHLAIREGYRDVVEYLAPRSNLGHQDTRGDNALDIAKATCTPDLVELLKRQLESTLAFQS

>XP_028830394.1 cyclin-dependent kinase 4 inhibitor D [Denticeps clupeoides]

MVVSENDAGRSLTAAAARGNTAEVRRMLEERRVHPDTANEFGRTALQVMMMGNTSVASLLLEHGANPNAQDRFGVTPVHDAARTGFLNTLRALVDHGASVNSPDSWGALPLHLAIREGHKDVVEYLVPCSNLGHHNSSGQTALDLARACCMPDVVEMLERQLESSPALQS

>XP_005346944.1 cyclin-dependent kinase 4 inhibitor D [Microtus ochrogaster]

MLLEEVRVGDRLSGAAARGDVQEVRRLLHRELVHPDALNRFGKTALQVMMFGSPAVALELLKQGASPNVQDASGTSPVHDAARTGFLDTLKVLVEHGADVNALDGTGSLPIHLAIREGHASVVSFLAPESDLHHRDSSGLTPLELARQRGAQNLMDILQRHMVIPV

>XP_038179379.1 cyclin-dependent kinase 4 inhibitor D [Arvicola amphibius]

MLLEEVRVGDRLSGAAARGDVQEVRRLLHRELVHPDALNRFGKTALQVMMFGSPAVALELLKQGASPNVQDASGTSPVHDAARTGFLDTLKVLVEHGADVNALDGTGSLPIHLAIREGHTSVVSFLAPESDLHHRDASGLTPLELARQRGAQNLMDILQRHMVIPV

>XP_003413138.1 cyclin-dependent kinase 4 inhibitor D [Loxodonta africana]

MLLEEVHAGDRLSGAAARGDVQEVRRLLHRELMHPDALNRFGKTALQVMMFGSPTIALELLKQGASPNVQDVSGTTPAHDAARTGFLDTLKVLVEHGADVNTPDGTGALPIHLAVREGHAAVVSFLAAESDLHHRDARGLTPLELAQQRGARDLMDILQGHLVAPL

>XP_005078607.1 cyclin-dependent kinase 4 inhibitor D [Mesocricetus auratus]

MLLEEVRVGDRLSGAAARGDVQEVRRLLHRELVHPDALNRFGKTALQVMMFGSPAVALELLKQGASPNVQDASGTSPVHDAARTGFLDTLKVLVEHGADVNALDGTGSLPIHLAIREGHSSVVSFLAPESDLHHRDTSGLTPLDLARQRGDQNLMDILQGHMVIPM

>XP_036049086.1 cyclin-dependent kinase 4 inhibitor D [Onychomys torridus]

MLLEEVLVGDRLSGAAARGDVQEVRRLLHRELVHPDALNRFGKTALQVMMFGSPAVALELLKQGASPNVQDASGTSPVHDAARTGFLDTLKVLVEHGADVNVLDSTGSLPIHLAIREGHSSVVSFLASDSDLHHRDSSGLTPLELARQRGDQNLMDILQGHMVIPM

>XP_012646092.1 cyclin-dependent kinase 4 inhibitor D [Microcebus murinus]

MLLEEVRAGDRLSGAAARGDVQEVRRLLHRELVHPDALNRFGKTALQVMMFGSTTIALELLKQGASPNVQDISGTSPVHDAARTGFLDTLKVLVEHGADVNAPDGTGALPIHLAVREGHAAVVGFLAAESDLHHRDARGLTPLELARQRGAQGLMDILQGHMVAPL

>XP_020023338.1 cyclin-dependent kinase 4 inhibitor D [Castor canadensis]

MLLEEVRAGDRLSGAAARGDVQEVRRLLHRELVHPDALNRFGKTALQVMMFGSPTIALELLKQGASPNVQDDSGTSPIHDAARTGFLDTLKVLVEHGADVNAPDGTGALPIHLAVREGHSAVVSFLASESDLHHRDASGLTPLELARQRGAQDLLDILQGHTVIPL

>XP_016009123.1 cyclin-dependent kinase 4 inhibitor D [Rousettus aegyptiacus]

MLLEEVRAGDRLSGAAARGDVQEVRRLLHRELVHPDSLNRFGKTALQVMMFGSPAIALELLKQGASPNVQDASGTTPAHDAARTGFLDTLKVLVEHGADVNVPDDTGALPIHLAVLEGHTTVVSFLAAESDLHHRDARGLTPFELAQGRGAHDLMDVLQGHTVASL

>XP_017657926.1 cyclin-dependent kinase 4 inhibitor D isoform X1 [Nannospalax galili]

MHCSPSRGRIATPRLGNPKYFDPDPAGSPSTHLHWAWGLGRFHRPPVSTMLLEEVRVGDRLSSAAARGDVHEVRRLLHRELVHPDSVNRFGKTALQVMMFGNPTIALELLKQGASPNVQDASGTSPVHDAARTGFLDTLKVFLDHGADVNAPDGTGALPIHLAVREGHSTIVSFLAPESDLHHRDAEGLTPLELARQRGAQDLVDILQQHMVIPL

>XP_037674260.1 cyclin-dependent kinase 4 inhibitor D [Choloepus didactylus]

MLLEEVRAGDRLSGAAARGDVQEVRRLLHRELVHPDALNRFGKTALQVMMFGSPTIALELLKQGATPNIQDASGTTPAHDAARTGFLDTLKVLVEHGADVNAPDAAGALPIHLAVREGHAAVVRFLAGESDLHHRDAGGLTPVELARQTGAQDVLDILQGHAVAPL

>XP_024412717.1 cyclin-dependent kinase 4 inhibitor D [Desmodus rotundus]

MLLEEVRAGDRLSGAAARGDVEEVRRLLSRELVHPDVLNRFGKTALQVMMFGSPTIALELLKQGASPNVQDASGTTPAHDAARTGFLDTLKVLVEHGADVNTPDNTGALPIHLAVREGHASVVSFLAAESDLHHRDTRGLTPLELAQGRGAQDLMDILQGHMVAPL

>XP_027443300.1 cyclin-dependent kinase 4 inhibitor D isoform X3 [Zalophus californianus]

MLLEEVRAGDRLSGAAARGDVQEVRRLLHRELVHPDALNRFGKTALQVMMFGSPTIALELLKQGASPNVQDTTGTTPAHDAARTGFLDTLKVLVEHGADVNAPDGTGALPIHLAVREGHTAVVRFLATESDLHHRDARGLTPLELAQGIGAQDLMDILQGHTVVLL

>XP_008066188.1 cyclin-dependent kinase 4 inhibitor D [Carlito syrichta]

MLLEEVRAGDRLSGAAARGDVQEVRRLLHRELVHPDALNRFGKTALQAMMFGSTTIALELLKQGASPNVQDSTGASPVHDAARTGFLDTLKVLMEHGADVNMPDGTGALPIHLAVQEGHAAVVSFLAAESDLHHRDARGLTPLELAQQRGAQGLMDILQGHMVAPL

>XP_011378584.1 cyclin-dependent kinase 4 inhibitor D [Pteropus vampyrus]

MLLEEVRAGDRLSGAAARGDVQEVRRLLHRELVHPDSLNRFGKTALQVMMFGSPAIALELLKQGASPNVQDASGTTPAHDAARTGFLDTLKVLVEHGADVNLPDDTGALPIHLAVLEGHTTVVSFLAAESDLHHRDARGLTPFELAQGRGAHDLMDMLQGHTVASL

>XP_013977871.2 cyclin-dependent kinase 4 inhibitor D [Canis lupus familiaris]

MLLEEVRAGDRLSGAAARGEVQEVRRLLHRELVHPDALNRFGKTALQVMMFGSPTIALELLKQGASPNVQDATGTTPAHDAARTGFLDTLKVLVEHGADVNAPDGTGALPIHLAVREGHTAVVSFLATESDLHHRDARGLTPLELAQGIGAQDLMDILQGHTVVLL

>XP_021028200.1 cyclin-dependent kinase 4 inhibitor D [Mus caroli]

MLLEEVCIGDQLSGAAARGDVQEVRRLLHRELVHPDALNRFGKTALQVMMFGSPAVALELLKQGASPNVQDASGTSPVHDAARTGFLDTLKVLVEHGADVNALDSNGSLPIHLAIREGHSSVVSFLAPESDLHHRDASGLTPLELARQRGAQNLMDILQGHMMIPM

>XP_015809885.1 PREDICTED: cyclin-dependent kinase 4 inhibitor D [**Nothobranchius furzeri**]

MVLSQMEAGKALTAAAAKGNTDEVQRILEECRVHPDTVNEFGRTALQVMMMGNSKIASLLLEKGADPNLQDRHGIAPIHDAARTGFLDTLQVLVEHGASVNLPDHNGALPIHIAIREGHRDVVKFLAPRSDLKHANTSGQTAVDAARASCVPDMMDSLFAHVHSS

>XP_024252100.1 cyclin-dependent kinase 4 inhibitor D [Oncorhynchus tshawytscha]

MVLSQSDAAKRLTAAAAKGNTDEVRRMLGXECRVHPDTVNQFGKTALQVMMMGNSNVASLLLEHGADPNITDRRGVSPAHDAARTGFVDTLRVLVEFGASVNRPDHTGTLPIHIAVREGYRDVIEFLAPLSNLKHPNTSGETAVDLARASSSCTPDVVELLERQLESKVFSHSPPLPL

>XP_029505687.1 cyclin-dependent kinase 4 inhibitor D-like [Oncorhynchus nerka]

MVLSQSDAAKSLTAAAAKGNTDEVRRMLGPECRVHPDTVNQFGKTALQVMMMGNSNVASLLLEHGADPNITDRRGVSPAHDAARTGFVDTLRVLVEFGASVNRPDHTGTLPIHIAVREGYRDVIEFLAPLSNLKHPNTSGETAVDLARASSSCTPDVVELLERQLESKVFSHSPPLPL

>XP_021412421.2 cyclin-dependent kinase 4 inhibitor D [Oncorhynchus mykiss]

MVLSQSDAAKSLTAAAAKGNTDEVRRMLGPECRVHPDTVNQFGKTALQVMMMGNSNVASLLLEHGADPNITDRRGVSPAHDAARTGFVDTLRVLVEFGASVNRPDHTGTLPIHIAVREGYRDVIEFLAPLSNLKHPNTSGETAVDLARASSSCTPDVVELLERQLESRVFSHSPPLPL

>XP_029545445.1 cyclin-dependent kinase 4 inhibitor D-like [Salmo trutta]

MVLSQSDAAKSLTAAAAKGNTDEVRRMLGPECRVHPDTVNQFGKTALQVMMMGNSNVARLLLEHGADPNITDRRGVSPAHDAARTGFVDTLRVLVEFGASVNMPDHTGTLPIHIAVREGYRDVIEFLAPLSNLKHPNTSGETAVDLARASSSCTPDVVELLERQLESKVFSHSPPLPL

>XP_033778913.1 cyclin-dependent kinase 4 inhibitor D [**Geotrypetes seraphini**]

MLLEEISEGDRLTRAAARGDLLEVQRLLQEELVHPDCHNRFGKTALQVMMFGNASIARELLKQGACANVQDSYGTTPAHDAARTGFLETLQLLVEHGADINVPDSSGSLPIHVAVREGHAGIVNFLVGESNLQHQDAQGLTALDLARLEGEPHVVSLLEQYQMEQP

>XP_030052911.1 cyclin-dependent kinase 4 inhibitor D [Microcaecilia unicolor]

MLLEEISEGDRLTRAAARGDLFEVQRLLQEELVHPDSHNRFGKTALQVMMFGNTPIAQELLKQGACANVQDAYGTTPAHDAARTGFLETLQLLVEHGADVNVPDSSGSLPIHVAIREGHADAVNFLVAESNLQHQDAQGLTALDLARLGGQSQVVSLLEQYQMEQP

>QOY46841.1 cyclin-dependent kinase inhibitor 2D [Ambystoma maculatum]

MLLDGEITAGDRLTSAAARGDTLEVRRLLHDELVHPDSINRFGKTALQVMMFGSPSVAQELLKQGASVTIQDEYGTTPAHDAARTGFLQTIQLLVEHGADINVPDSHGSLPIHLAVREGHEDVVNFLSAESNLRHRDSEGLTALDLARQGAEPQLVTILEQYMSVQT

>QOY46838.1 cyclin-dependent kinase inhibitor 2D [Ambystoma mexicanum]

MLLDGEITAGDRLTSAAARGDTLEVRRLLHDELVHPDSINRFGKTALQVMMFGSPSVAQELLKQGASVNIQDEYGTTPAHDAARTGFLQTIQLLVEHGADINVPDSHGSIPIHLAIREGHEDVVHFLSAESNLRHRDSEGLTALDLARQGAEPQLVTILEQYMSAQT

>QOY46840.1 cyclin-dependent kinase inhibitor 2D [Ambystoma andersoni]

MLLDGEITAGDRLTSAAARGDTLEVRRLLHDELVHPDSINRFGKTALQVMMFGSPSVAQELLKQGASVTIQDEYGTTPAHDAARTGFLQTIQLLVEHGADINVPDSHGSLPIHLAVREGHEDVVHFLSAESNLRHRDSEGLTALDLARQGAEPQLVTILEQYMSAQT

>QOY46839.1 cyclin-dependent kinase inhibitor 2D [Ambystoma velasci]

MLLDGEITAGDRLTSAAARGDMLEVRRLLHDELVHPDSINRFGKTALQVMMFGSPSVAQELLKQGASVTIQDEYGTTPAHDAARTGFLQTIQLLVEHGADINVPDSHGSIPIHLAVREGHEDVVHFLSAESNLRHRDSEGLTALDLARQGAEPQLVTILEQYMSVQT

>XP_037360262.1 cyclin-dependent kinase 4 inhibitor D [Talpa occidentalis]

MLLEEVRAGNRLSGAAARGDVQEVRRLLHRELVHPDALNRFGKTALQVMMFGSPTIALELLKQGASPNVQDASGTTPAHDAARTGFLDTLKVLVEHGADVNAPDGTGALPIHLAVREGHTAVVNFLAAESDLHHRDARGLTPLDLAQRRGAQDLLDILQGHTVAPL

>XP_037009860.1 cyclin-dependent kinase 4 inhibitor D [Artibeus jamaicensis]

MLLEEVCAGDRLSGAAARGDVLEVRRLLSRELVHPDVLNRFGKTALQVMMFGSPIIALELLKQGASPNVQDASGTTPAHDAARTGFLDTLKVLVEHGADVNMPDNTGALPIHLAVREGHAAVVSFLAAESDLYHRDTRGLTPLELAQGRGAQDLMDILQGHMVAPL

>XP_028377366.1 cyclin-dependent kinase 4 inhibitor D [Phyllostomus discolor]

MLLEEVCAGDRLSGAAARGDVLEVRRLLSRELVHPDVLNRFGKTALQVMMFGSPTIALELLKQGASPNVQDASGTTPAHDAARTGFLDTLKVLVEHGADVNTPDNTGALPIHLAVREGHTAVVSFLAAESDLHHRDTRGLTPLELAQGRGAQDLMDILQGHMVAPL

>XP_004595801.1 cyclin-dependent kinase 4 inhibitor D [Ochotona princeps]

MFLDEVHAGDRLSGAAARGDVDGVRRLLHRELVHPDALNRFGKTALQVMMFGNPTVARELLKQGASPNVQDAWGTSPAHDAARTGFLDTLKVLVDHGADINAPDASGALPIHLAVREGHTAVVRFLAAESDLGHRDAGGRTPLELARQTGARHLLDILQGRPL

>XP_036096069.1 cyclin-dependent kinase 4 inhibitor D [Molossus molossus]

MLLEEVRAGDRLSGAAARGDVQEVRRLLYRELVHPDALNRFGKTALQVMMFGSPTIALELLKQGASPNVQDTSGTTPAHDAARTGFLDTLKVLVEHGADVNAPDNTGALPIHLAVREGHTAVVNFLAAESDLHHRDTRGLTPLELAQGRGAQDLMDILQGHMVASL

>XP_004277411.1 cyclin-dependent kinase 4 inhibitor D [Orcinus orca]

MLLEEVRAGDRLSGAAARGDVQEVRRLLHRELVHPDALNRFGKTALQVMMFGSPTIALELLKQGASPNVQDASGTTPAHDAARTGFLDTLKVLVEHGADVNAPDGTGALPIHLAVREGHTAVVGFLAAESDLHHRDARGLTPLELARGRGAKDLMDILHIPL

>XP_019788637.1 cyclin-dependent kinase 4 inhibitor D [Tursiops truncatus]

MLLEEVRAGDRLSGAAARGDVQEVRRLLHRELVHPDALNRFGKTALQVMMFGSPTIALELLKQGASPNVQDASGTTPAHDAARTGFLDTLKVLVEHGADVNAPDGTGALPIHLAVREGHRAVVGFLAAESDLHHRDARGLTPLELARGRGAKDLMDILHIPL

>XP_006161597.2 cyclin-dependent kinase 4 inhibitor D [Tupaia chinensis]

MLLEEVRAGDRLSGAAARGDVHEVRRLLYRELVHPDALNRFGKTALQVMMFGSPVIALELLKQGASPNVQDASGTSPIHDAARTGFLDTLKVLVDHGADVNVPDGSGALPIHLAIQEGHTAVVNFLAAESDLHHKDARGLTPLELARQRGSQDLMDILQGHKVAPL

>XP_032989248.1 cyclin-dependent kinase 4 inhibitor D [Rhinolophus ferrumequinum]

MLLEEVRAGDLLSGAAARGDVQEVRRLLHRELVHPDVLNRFGKTALQVMMFGSPTIALELLKQGASPNVQDASGTTPAHDAARTGFLDTLKVLVEHGADVNAPDDTGALPIHLAVLEGHRAVVSFLAAESDLHHRDARGLTPLELARRRGAQDLMDILQRHTVAPL

>NP_001791.1 cyclin-dependent kinase 4 inhibitor D [**Homo sapiens**]

MLLEEVRAGDRLSGAAARGDVQEVRRLLHRELVHPDALNRFGKTALQVMMFGSTAIALELLKQGASPNVQDTSGTSPVHDAARTGFLDTLKVLVEHGADVNVPDGTGALPIHLAVQEGHTAVVSFLAAESDLHRRDARGLTPLELALQRGAQDLVDILQGHMVAPL

>XP_025224938.1 cyclin-dependent kinase 4 inhibitor D [Theropithecus gelada]

MLLEEVRSGDRLSGAAARGDVQEVRRLLHRELVHPDALNRFGKTALQVMMFGSTAIALELLKQGASPNVQDTSGTSPVHDAARTGFLDTLKVLVEHGADVNAPDGTGALPIHLAVQEGHTAVVSFLAAESDLHRRDARGLTPLELALQRGAQDLVDILQGHMVASL

>XP_002761779.1 cyclin-dependent kinase 4 inhibitor D [Callithrix jacchus]

MLLEEVCAGDRLSGAAARGDVQEVRRLLHRELVHPDALNRFGKTALQVMMFGSTTIALELLKQGASPNVQDTSGTSPIHDAARTGFLDTLKVLVEHGADVNAPDGTGALPIHLAVQEGHTAVVNFLAAESDLHRRDARGLTPLELALQRGAQDLVDILQGHMVAPL

>XP_017355460.1 cyclin-dependent kinase 4 inhibitor D [Cebus imitator]

MLLEEVCAGDRLSGAAARGDVQEVRRLLHRELVHPDALNRFGKTALQVMMFGSTTIALELLKQGASPNVQDTTGTSPVHDAARTGFLDTLKVLVEHGADVNAPDGTGALPIHLAVQEGHTAVVNFLAAESDLHRRDARGLTPLELALQRGAQDLVDILQGHMVAPL

>XP_012291669.1 cyclin-dependent kinase 4 inhibitor D [Aotus nancymaae]

MLLEEVCAGDRLSGAAARGDVQEVRRLLHRELVHPDALNRFGKTALQVMMFGSTTIALELLKQGASPNVQDTFGTSPVHDAARTGFLDTLKVLVEHGADVNAPDGTGALPIHLAVQEGHTAVVNFLAAESDLHRRDARGLTPLELALQRGAQDLVDILQGHMVTPL

>XP_003798006.1 cyclin-dependent kinase 4 inhibitor D [Otolemur garnettii]

MLVEELYAGDRLSGAAARGDVQEVRRLLYRELVHPDVLNRFGKTALQVMMFGSTTIALELLKQGASPNIQDMSGTSPVHDAARTGFLDTLKVLVEHGADVNLPDGTGALPIHLAVQEGHITVVSFLAAESDLHHRDARGLTPLELAQQRGAQDLVDILQGHVVAPL

>XP_026255469.1 cyclin-dependent kinase 4 inhibitor D [Urocitellus parryii]

MLLEEVRVGDRLSGAAARGDVQEVRRLLYRELVHPDALNRFGKTALQVMMFGSPAIXLELLKQGASPNVQDASGTSPVHDAARTGFLDTLKVLVEHGADVNAPDGTGXLPIHLAVQEXHSAVVSFLAPESDLQHKDARGLTPLELARQRGSQDIMDILQGCTVIPL

>XP_028339539.1 cyclin-dependent kinase 4 inhibitor D [**Physeter catodon**]

MLLEEVRAGDRLSGAAARGDVQEVRRLLHRELVHPDALNRFGKTALQVMMFGSPTIALELLKQGASPNVQDASGTTPAHDAARTGFLDTLKVLVEHGADVNAPDGTGALPIHLAVREGHTAVVSFLAAESDLHHRDARGLTPLELARGRGAKDLMDILQWHTVAPL

>XP_026337176.1 cyclin-dependent kinase 4 inhibitor D isoform X1 [Ursus arctos horribilis]

MLLEEVRAGDRLSGAAARGDVQEVRRLLHRELVHPDALNRFGKTALQDPLPSAPTQVMMFGSPTIALELLKQGASPNVQDTTGTTPAHDAARTGFLDTLKVLVEHGADVNVPDGTGALPIHLAVREGHTAVVSFLATESDLHHRDARGLTPLELAQGIGAQDLMDILQGHTVVLL

>XP_011225947.1 cyclin-dependent kinase 4 inhibitor D isoform X1 [Ailuropoda melanoleuca]

MLLEEVRAGDRLSGAAARGDVQEVRRLLHRELVHPDALNRFGKTALQDPLPSAPTQVMMFGSPTIALELLKQGASPNVQDTTGTTPAHDAARTGFLDTLKVLVEHGADVNVPDGTGALPIHLAVREGHTAVVSFLATESDLHHRDARGLTPLELAQGIGAQDLMDILQGHAVVLL

>XP_030401929.1 cyclin-dependent kinase 4 inhibitor D [**Gopherus evgoodei**]

MLLGDEISAGDRLSGAAARGDLAELRCLLHQELVHPDSHNRFGKTALQVMMFGNTFVAEELLKQGASPNIQDEAGRAPAHDAARTGFLDTLRVLVEHGADINVPDGAGALPIHVAIREGHTEVVQYLAPDSNLQHRDAEGRTPLELAHLLGLSHLEAILGQHLSAPA

>XP_014461742.2 PREDICTED: cyclin-dependent kinase 4 inhibitor D [**Alligator mississippiensis]**

MLLGDELSGGDRLSSAAARGDLAEVRRLLRDELVHPDALNRFGRTALQVMMMGSMAVAQELLKQGASANVQDAGGRAPAHDAARGGFADTLRVLLEHGADANAPDGTGALPLHVAVAEGHAPAVAVLAPVSDLGRRDAHGRTPLNLAQHLGRPGIVAILEQHCPGPA

>NP_001072839.1 cyclin-dependent kinase 4 inhibitor D [**Xenopus tropicalis**]

MLLQETSAGDRLTRAAARGDLLEVKRLLHEERIHPDCLNRFGKTALQVMMFGSTPVASELLKQGASPNIQDAYGTTPAHDAARCGFLDTLQVLVQHGAEINTPDASGSLPIHLALKAGHVPVITYLALISDLQHRDREGHTPSQLAAILDPRLASIFELST

>NP_001086884.1 cyclin-dependent kinase inhibitor 2D S homeolog [Xenopus laevis]

MLLQETSAGDRLTRAAARGDLTEVKRLLHEERIHPDCLNCFGKTALQVMMFGSTPVASELLKQGATPNIQDAYGTTPAHDAARCGFLDTLQVLVQHGAEVNTPDASGSLPIHLALKAGHVPVITYLALISDLQQHDREGHTPSQLAAILDPRLASIFELST

>XP_040271766.1 cyclin-dependent kinase 4 inhibitor D [Bufo bufo]

MLLHETSAGDLLTRAAAQGDLAEVKRLLYQERIHPDCLNRFGRTALQVMMFGSTPVASELLKQGASPNIQDSYGISPAHDAARMGFLDTLQVLVQYGADVNTPDASGCLPVHLALREGHIPVIAYLASRSNLQHQDREGRTPPQLASMLDPNLAAVLELHR

>XP_040202360.1 cyclin-dependent kinase 4 inhibitor D [Rana temporaria]

MLLQETSAGDLLTKASAQGDMEEVQRLLHQERIHPDCLNHFGRTALQVMMFGSTSIASELLKQGATANIQDSHGITPAHDAARTGFLDTLQILVQYGADLNVPDVTGSLPIHLALREGHLPVVVYLASRSNLQHRDREGRTPLQLASMLNPSLAAVLEQYT

>XP_002662282.2 cyclin-dependent kinase 4 inhibitor D [**Danio rerio**]

MMVLDGLGCGKSLSAAAAQGDARAVRRILQDHRVEPDALNEFGKTALQVMMMGSSAVAAVLLDFGADPNVQDRSGVTPAHDAARTGFLETLRVLVDGGASVNVPDHSGALPLHIAVREGHWDVVEYLAPLTELRWLDGRRVEELLEHLQATGSG

>KAA0713498.1 Cyclin-dependent kinase inhibitor 2A [Triplophysa tibetana]

MVLDGFGCGKSLSAAAAQGNVVAVRRILQDHRVEPDSLNEFGKTALQVMMMGCTSVARVLLEHGADPNVQDRCGVTPAHDAARTGFLETLRALVEHSASVNIPDHSGSIPVHIAIREGHWDVVEYLAPLSDLSHRDSRGANALDVARAARAPEMVDFLEPKMN

>XP_039512500.1 cyclin-dependent kinase 4 inhibitor D [Pimephales promelas]

MMVLDGLGICGKSLSTAAAQGNVDSVRRILQDHRVEPDTLNEFGKTALQVMMMGCTSVACVLLDHGADPNVQDRCGVTPAHDAARTGFLDTVRVLVDYGASVNVPDHSGALPIHIAIRESNWDVVEFLAPLSNLGHQDNTGSKPLDVARDACAPEIVELLEHLMASSSN

>XP_026111966.1 cyclin-dependent kinase 4 inhibitor D [Carassius auratus]

MVVDGFGRGKSLSAAAARGNVDAVRRMLQDHRLQPDTLNEFGKTALQVMMMGCTSVARVLLDHGADPNVQDRCGVTPAHDAARTGFLDTLRVLVEHGASVNVPDHSGALPLHTAVREGHRDVVEYLAPLSDLGHRDGRGVTALDTARDACALEMVELLERLMGTSSH

>XP_007231367.2 cyclin-dependent kinase 4 inhibitor D [Astyanax mexicanus]

MLINGKEAGRSLTSAAARGDAAEVRLILEDCRVHPDTANEFGKTALQVMMMGNTSVACLLLEKGADPNIPDRFGITPAHDAARTGFLETLHVLVDYGASVNTPDQFGALPIHHAIREGYRDVVEYLAPCSNLAHEDKEGNNALDIANATCPPDMVELLKRQLESTLAFQS

>XP_018617054.2 cyclin-dependent kinase 4 inhibitor D [Scleropages formosus]

MQVVANEERAGRYLSAAAATGDVRALRRLLEERRVHPDTANEFGKTALQVMMMGSTGVALLLLQHGANANVQDGQGVTPVHDAARTGFVHTLRALVDFGASVNVADCSGALPIHIALREGHWDVVEFLAPLSDLRHRDACGRSALDVAHSAGSGDMVALLEHHLKSS

>XP_035253334.1 cyclin-dependent kinase 4 inhibitor D-like [Anguilla anguilla]

MVLSENDAGKNLSAAAARGNVKELRRMLEEQRVHPDTVNEFGRTALQVMMMGSTSVACLLLEHGANANVQDRLGVTPAHDAARAGFVDTLRVLVQFGASVNTPENTGALPIHIAIREGHSDVVEFLAPRSNLNHQDTSGDTALDVARAMRCPGVVELLERQSESSLKCQS

>XP_017495629.1 cyclin-dependent kinase 4 inhibitor D [Manis javanica]

MLLEEVRAGDRLSGAAARGDVQEVRRLLHHELVHPDVLNRFGKTALQVMMFGSPIIALELLKQGASPNVQDASGTTPAHDAARTGFLDTLKVLVEHGADVNVPDGTGALPIHLAVREGHTAVVSFLAPESDLLYRDARGLTPLGVGAGEEGSGSHGHT
